# Functional Analyses of *Trichoderma reesei* LAE1 Reveal Conserved and Contrasting Roles of This Regulator

**DOI:** 10.1534/g3.112.005140

**Published:** 2013-02-01

**Authors:** Razieh Karimi-Aghcheh, Jin Woo Bok, Pallavi A. Phatale, Kristina M. Smith, Scott E. Baker, Alexander Lichius, Markus Omann, Susanne Zeilinger, Bernhard Seiboth, Catherine Rhee, Nancy P. Keller, Michael Freitag, Christian P. Kubicek

**Affiliations:** * Institute of Chemical Engineering, Research Division Biotechnology and Microbiology; ** Austrian Center of Industrial Biotechnology, c/o Institute of Chemical Engineering, University of Technology, 1060 Vienna, Austria; † Department of Medical Microbiology and Immunology, University of Wisconsin-Madison, Wisconsin 53706-1521; ‡ Department of Biochemistry and Biophysics, Oregon State University, Corvallis, Oregon 97331; § Fungal Biotechnology Team, Chemical and Biological Process Development Group, Pacific Northwest National Laboratory, Richland, Washington 99352

**Keywords:** cellulase, secondary metabolites, LaeA, *Trichoderma reesei*, *Aspergillus nidulans*, ChIP-seq, transcriptome

## Abstract

The putative methyltransferase LaeA is a global regulator that affects the expression of multiple secondary metabolite gene clusters in several fungi, and it can modify heterochromatin structure in *Aspergillus nidulans*. We have recently shown that the LaeA ortholog of *Trichoderma reesei* (LAE1), a fungus that is an industrial producer of cellulase and hemicellulase enzymes, regulates the expression of cellulases and polysaccharide hydrolases. To learn more about the function of LAE1 in *T. reesei*, we assessed the effect of deletion and overexpression of *lae1* on genome-wide gene expression. We found that in addition to positively regulating 7 of 17 polyketide or nonribosomal peptide synthases, genes encoding ankyrin-proteins, iron uptake, heterokaryon incompatibility proteins, PTH11-receptors, and oxidases/monoxygenases are major gene categories also regulated by LAE1. chromatin immunoprecipitation sequencing with antibodies against histone modifications known to be associated with transcriptionally active (H3K4me2 and -me3) or silent (H3K9me3) chromatin detected 4089 genes bearing one or more of these methylation marks, of which 75 exhibited a correlation between either H3K4me2 or H3K4me3 and regulation by LAE1. Transformation of a *laeA*-null mutant of *A. nidulans* with the *T. reesei lae1* gene did not rescue sterigmatocystin formation and further impaired sexual development. LAE1 did not interact with *A. nidulans* VeA in yeast two-hybrid assays, whereas it interacted with the *T. reesei* VeA ortholog, VEL1. LAE1 was shown to be required for the expression of *vel1*, whereas the orthologs of *velB* and *VosA* are unaffected by *lae1* deletion. Our data show that the biological roles of *A. nidulans* LaeA and *T. reesei* LAE1 are much less conserved than hitherto thought. In *T. reesei*, LAE1 appears predominantly to regulate genes increasing relative fitness in its environment.

The *Aspergillus nidulans* LaeA protein, a putative *S*-adenosylmethionine-dependent (SAM) methyltransferase, was originally described as a global regulator of secondary metabolism (Bok and Keller 2004). It was later shown to be required for the biosynthesis of secondary metabolites in *Aspergillus* (Bok *et al.* 2006a; Bouhired *et al.* 2007; Perrin *et al.*, 2007; Kale *et al.* 2008; Georgianna *et al.* 2010; Oda *et al.* 2011), in the industrial fungus *Penicillium chrysogenum* (*e.g.*, penicillin) and the phytopathogenic *Fusarium fujikuroi* (*e.g.*, gibberellins) and *Cochliobolus heterostrophus*, respectively (Hoff *et al.* 2010; Wiemann *et al.* 2010; Butchko *et al.* 2012; Wu *et al.* 2012). LaeA acts in a complex with VeA, *i.e.*, Velvet, and VelB *i.e.*, Velvet-like B (Bayram *et al.* 2008a). Because LaeA was shown to control a region with discrete borders, encompassing 70 kb of the sterigmatocystin cluster in *A. nidulans*, an epigenetic control function of LaeA was postulated (Bok *et al.* 2006b), further defined as somehow counteracting H3K9 methylation in the sterigmatocystin gene cluster (Reyes-Dominguez *et al.* 2010).

More recently evidence emerged that LaeA also controls developmental events, such as conidiation in numerous fungi (Bok *et al.* 2005; Sugui *et al.* 2007; Hoff *et al.* 2010; Wiemann *et al.* 2010; Chang *et al.* 2012; Jiang *et al.* 2012; Wu *et al.* 2012), including a light-dependent effect on asexual development in *A. nidulans* (Sarikaya Bayram *et al.* 2010). Moreover, the absence of LaeA results in the formation of significantly smaller fruiting bodies in *Aspergillus flavus* (Amaike and Keller 2009) and *A. nidulans*, the latter associated with a decrease in the formation of specific globose (or “Hülle”) cells, which nurse the young fruiting body during development (Carvalho *et al.* 2002). Thus, current knowledge suggests that LaeA has a dynamic role in both fungal morphological and chemical development.

It is, however, still unknown whether these roles for Lae proteins are uniformly distributed throughout the Pezizomycota. We have recently shown that LAE1, the *Trichoderma reesei* ortholog of *Aspergillus* LaeA, controls the expression of polysaccharide hydrolytic enzymes (Seiboth *et al.* 2012). *T. reesei* is a saprophyte specialized on pre-degraded wood (Druzhinina *et al.* 2011), thus suggesting a potential nutritional and eco-physiological role of LAE1 for this species. Interestingly, the control of expression of the polysaccharide hydrolase genes by LAE1 does not appear to involve changes in H3K9 methylation (Seiboth *et al.* 2012) as it does for some secondary metabolite gene clusters in *A. nidulans* (Reyes-Dominguez *et al.* 2010). Here we have extended our earlier findings to the genome-wide scale, with the goal to identify targets of LAE1 function in *T. reesei*, and we compare known functions of *A. nidulans* LaeA and *T. reesei* LAE1.

## Materials and Methods

### Strains and growth conditions

Strains *T. reesei* QM 9414 (ATCC 26921), an early cellulase producing mutant, and the mutant strains C.P.K. 3793 (*Δlae1*) and C.P.K. 4087 (*lae1OE*) derived from it (Seiboth *et al.* 2012), *gna3*_QL_ (Schmoll *et al.* 2009), *Δpkac1*, and *Δacy1* (Schuster *et al.* 2012) were used throughout this work. They were grown in Mandels-Andreotti medium (Mandels and Andreotti 1978), using 1% (w/v) lactose as a carbon source.

*A. nidulans* strains used or created in this study are RJW33.2 (ΔlaeA::metG, wA3, trp801, pyroA4, veA1), TJW123.20 (ΔlaeA::metG, wA3, trp801, lae1::pyroA, veA1), RDIT2.1 (metG1, veA), RJW41.A (ΔlaeA::metG, veA), RDIT9.32 (veA), and RCSR4.16 (ΔlaeA::metG, lae1::pyroA, veA). Unless otherwise noted, they were grown on glucose minimal medium, or GMM (Shimizu and Keller 2001), with additional supplements for auxotrophic strains (pyrodoxin, methionine or tryptophan). All strains are maintained as glycerol stocks at −80°. *Escherichia coli* JM109 (Promega, Madison, WI) was used for plasmid construction and amplification.

### Transformation of *A. nidulans* with *T. reesei lae1*

A *lae1* fragment obtained from pRKA41617ptrA (containing a 2.8-kb genomic clone of the *T. reesei lae1* that contained 900 bp of 5′ and 500 bp of 3′ noncoding regions) was inserted into *Pst*I and *Spe*I sites of pJW53 to create pJW139.3 (Bok and Keller 2004), which places genes at the *pyroA* locus in *A. nidulans*. The recipient strain RJW33.2 was transformed with pJW139.3 as described previously (Bok and Keller 2004). Transformants were confirmed by polymerase chain reaction (PCR) and Southern blots and one correct transformant (TJW123.20) was sexually crossed with RDIT2.1 to create a prototrophic strain (RCSR4.16). Northern analysis was performed to assess *lae1* expression.

### Nucleic acid analysis

The extraction of DNA from fungi and bacteria, restriction enzyme digestion, gel electrophoresis, blotting, hybridization, and probe preparation were performed by standard methods (Ausubel *et al.* 1999). Total RNA was extracted from *Aspergillus* strains by use of Trizol reagent (Invitrogen, Carlsbad, CA) according to the manufacturer’s instructions. RNA blots were hybridized with ^32^P-labeled *lae1* DNA fragments, which were generated by PCR using gene-specific primers, lae1SpeI (5′TACTAGTCTACCTCTTTCAAGGAGC) and lae1PstI (5′TCTGCAGACGAGAGATCATATATCCG).

### Analysis of *A. nidulans* growth and development

Conidia (asexual spores) and ascospore (sexual spores) production were examined using point inoculation or overlay methods in light and/or dark conditions. In point inoculation, 1 µL of 10^6^ conidia/mL was deposited on the middle of the plate. In overlay, 5 mL of top medium (0.75% agar) with 10^6^ conidia was applied onto the bottom medium.

Point inoculated colonies were cultured by inoculating on GMM plates with 10^3^ spores and incubating in light and dark conditions at 37° for 5 d. Colony diameter was measured and 1-cm cores from the center were taken and homogenized with 3 mL of water. Conidia were counted using a hemocytometer under a light microscope. Conidia per milliliter were determined by standard calculation (counted conidia in total grid ×x dilution factor × 10^4^).

For overlay cultures, 10^6^ spores in 5 mL of CHAMPS medium (0.75% agar) (Greene *et al.* 2003) were overlaid on top of 25 mL of CHAMPS medium (20 g of glucose, 16 g of agar, 5 g of yeast, and 1 mL of trace element solution per liter of medium) and then incubated in the dark to promote sexual development at 37° for 5 d. One-cm cores were taken from the plates, homogenized in 3 mL of water, and released ascospores were counted using a hemocytometer under a light microscope.

### Statistical analysis

Spore data were statistically compared by analysis of variance using the Tukey-Kramer test for multiple comparison. Statistically significant mean values, indicated with different letters in the figures, are significant at *P* < 0.05.

### Metabolite assays

Thin-layer chromatography (TLC) was used to assess sterigmatocystin production. One-centimeter cores were punched from the center of point inoculated plates and homogenized with 3 mL of sterile double-distilled H_2_O. Three milliliters of chloroform were added, mixed well, and the samples centrifuged for 10 min. The organic layer was removed, transferred into a 3-mL glass vial, and left to dry in a fume hood overnight. Dried extracts were resuspended with 100 μL of chloroform and 5 or 10 μL were loaded onto a non-ultraviolet (UV)-coated TLC plate. Sterigmatocystin was spotted as a standard. The plates were run in chloroform/acetone (8:2) solvent and stained with 15% aluminum chloride in 95% ethanol. TLC plates were viewed under 254-nm UV light.

### Yeast two-hybrid analysis

The yeast two-hybrid plasmids pTLex3 and pGAD424 (Cho *et al.* 2003) were modified by the “quick change” technique (van den Ent and Löwe 2006) using *Pfu*Ultra II fusion HS DNA polymerase (Stratagene, Santa Clara, CA). The bait plasmid with *lae1* cDNA was constructed using two quick change primers, TlaeALexAFWD (5′CGCAACGGCGACTGGCTGGAATTCAAGCTTA TGTCTCGAAACGCTCCCAACGGGTGTG) and TlaeALexAREV2 (5′CTTGGCTGCAG GTCGACTCGAGCGGCCGTTAAGCAGAGGATTCCTCTCTTCTAGATGGC) to place *lae1* into pTLex3. *lae1*-pTLex3 was cotransformed into the *Saccharomyces cerevisiae* reporter strain L40 with either pGAD424 (empty prey vector) or with *A. nidulans veA* cDNA in pGAD424 (Bayram *et al.* 2008b). The prey plasmid with *vel1* cDNA was constructed using two Quick change primers, TveA424FWD (5′GAGATCGAATTCCCGGGGATCCGTCGAATGGCG ACGCCTTCCTCCGTGGCCTCGTC) and TveA424REV (5′GCACAGTTGAAGTGAAC TTGCGGGGTTTTTACACCTGGTATTGGTTGAAGGTGACAACG to place *vel1* into pGAD424. *ve1*-pGAD424 was cotransformed into the *S. cerevisiae* reporter strain L40 with either pTLex3 (empty bait vector), with *A. nidulans laeA* cDNA in pTLex3 (Bayram *et al.* 2008b) or with *lae1*-pTLex3. Also, *A. nidulans laeA* cDNA in pTLex3 was cotransformed with *A. nidulans veA* cDNA in pGAD424 as a positive control. Transformants were selected on -UTL (-ura, -trp, -leu) containing 2% (w/v) glucose (SD) media. Six transformants of each combination were tested for their coloration on -UTL medium containing X-Gal.

### Transcriptome analysis

Mycelia of *T. reesei* were harvested after 26 hr of growth, total RNA extracted (Chirgwin *et al.* 1979), and purified using the RNeasy MinElute Cleanup Kit (QIAGEN, Hilden, Germany). RNA quality and quantity were determined on gels and using a Nanodrop spectrophotometer. High-quality purified RNAs were submitted to Roche-NimbleGene (40 μg per 3-microarray set) where cDNAs were synthesized, amplified, and labeled and used for subsequent hybridization.

A high-density oligonucleotide microarray (Roche-NimbleGen, Inc., Madison, WI) was constructed, using 60-mer probes representing the 9143 genes of *T. reesei*. Microarray scanning, data acquisition, and identification of probe sets showing significant differences (at *P* < 0.05) in expression levels between different conditions were performed by Roche-NimbleGen (www.nimblegen.com). Values were normalized by quantile normalization (Bolstad *et al.* 2003) and the RMA algorithm (Irizarry *et al.* 2003). After elimination of transcripts that exhibited an SD >20% of the mean value within replicates, false discovery rates (Benjamini and Hochberg 1995) were used to assess the significance of values. Transcripts showing significantly different expression compared with the 18-hr control (at least twofold changes at *P* < 0.05) were grouped by k-means clustering as implemented in Array Star 3.0.1 (Array Star Inc., Madison, WI). Gene accession numbers were annotated according to version 2 of the *T. reesei* genome assembly (http://genome.jgi-psf.org/Trire2/Trire2.home.html), and ambiguous cases annotated manually.

Genes were classified according to their major annotation in the MIPS Functional Catalogue (FUNCAT) (Ruepp *et al.* 2004). To determine whether there were differences in the functional categories in each cluster, the distribution of categories within each cluster was compared with the total distribution of the same cluster within all the annotated genes using independent χ^2^ tests. The microarray data and the related protocols are available at the GEO web site (www.ncbi.nlm.nih.gov/geo/) under accession number GSE22687 (platform GPL10642).

### Chromatin immunoprecipitation (ChIP) and ChIP-sequencing

To carry out ChIP-sequencing with *T. reesei*, we adapted a protocol developed for *Neurospora crassa* (Tamaru *et al.* 2003; Smith *et al.* 2011). QM 9414, Δ*lae1*, and *tef1*::*lae1* strains were grown for 5 d in the dark on 2% PDA medium and spores harvested. Flasks with 50 mL of lactose medium were inoculated with either 1 × 10^5^ or 1 × 10^6^ spores mL^−1^ and grown in the dark for 26 hr. All further steps were as described previously (Tamaru *et al.* 2003). DNA obtained by ChIP was suspended in 30 μL and used for construction of ChIP-seq libraries (Pomraning *et al.* 2009; Pomraning *et al.* 2012). We obtained 1.4–4.8 million mapped reads (between 76% and 98% of the total reads) for the nine libraries we sequenced (three strains and three antibodies). The antibodies used were from Active Motif (H3K4me3, 39159; H3K9me3, 39161) and Millipore (H3K4me2, 07-030). We used one additional H3K9me3 antibody from Abcam (ab8898), which resulted in less enrichment than with the Active Motif antibody (data not shown).

### Real-time PCR

DNase I-treated (Fermentas) RNA (5 µg) was reverse-transcribed with the RevertAid First Strand cDNA Kit (Fermentas) according to the manufacturer’s protocol with a combination (1:1) of the provided oligo-dT and random hexamer primers. All real-time PCR experiments were performed on a Bio-Rad (Hercules, CA) iCycler IQ. For the reaction the IQ SYBR Green Supermix (Bio-Rad) was prepared for 25-µL assays with standard MgCl_2_ concentration (3 mM) and a final primer concentration of 100 nM each. All assays were carried out in 96-well plates which were covered with optical tape. Primers, amplification efficiency and R-square values are given in Supporting Information, Table S1. Measurements for *tef1* were performed with both protocols for reference calculation. Determination of the PCR efficiency was performed using triplicate reactions from a dilution series of cDNA (1; 0.1; 0.01; 0.001). Amplification efficiency was then calculated from the given slopes in the IQ5 Optical system Software v2.0. Expression ratios were calculated using REST Software (Pfaffl *et al.* 2002). All samples were analyzed in two independent experiments with three replicates in each run.

## Results

### Genome-wide analysis of LAE1 function

To identify genes that are influenced by LAE1 function in *T. reesei*, we determined the transcriptional profiles of the wild-type, *Δlae1*, and *lae1*OE strains by comparisons of relative transcript levels between *Δlae1* and *lae1*OE, respectively, *vs.* the wild-type strain. A total of 2743 genes were differentially expressed at least twofold level either between the parent and *Δlae1* or the parent and *lae*1OE (at *P* < 0.05; Table 1; the complete list of genes is shown in Table S2). Only 71 genes were down-regulated in *Δlae1* and up-regulated in *lae1*OE (Table 1), a pattern often observed for secondary metabolite cluster expression in other systems (Bok and Keller 2004, Georgianna *et al.* 2010). The majority of genes (1113) were unaffected in the *Δlae1* strain but up-regulated in the *lae1*OE strain. A set of 372 genes were significantly down-regulated in both the *Δlae1* and *lae1*OE strains. Interestingly, no transcripts were found that were up- or down-regulated in *Δlae1* but unaffected in *lae1OE*. The remaining 1188 genes included 17 genes that were up-regulated in *Δlae1* and *lae1*OE, 930 that were down-regulated in the *lae1*OE strain but unaffected in *Δlae1*, and 240 that were up-regulated in both mutant strains (see Table 1 for details).

**Table 1:** Up-regulated and down-regulated genes in *lae1* manipulated strains of *T. reesei*

*Δlae1*	*lae1*OE	Genes
Down	Up	71
Down	Down	372
Down	*^a^*	0
Up	Down	17
Up	*^a^*	0
Up	Up	240
*^a^*	Up	1113
*^a^*	Down	930
Total		2743

a Indicates expression <twofold in either direction, and is therefore considered to be unaffected by LAE1.

Of the 1556 genes whose transcriptional behavior was consistent with a positive action of LAE1 (indicated in bold in Table 1), 221 genes shared no orthologs with any other fungus for which data are available, and 588 encoded unknown proteins that were conserved in other Pezizomycota (analyses done on July 3, 2012). To identify the gene families that were significantly affected by either loss-of-function or overexpression of LAE1, we expressed them as percentage of the total number of transcripts in the respective fraction of the transcriptome (*i.e.*, the categories shown in Table 1), and compared this with the percentage of these genes in the total genome (Table 2). This analysis revealed that the 71 genes with decreased expression in *Δlae1* and up-regulated in *lae1*OE largely belonged to the glycosyl hydrolases reported recently (Seiboth *et al.* 2012); in addition, HET (heterokaryon incompatibility) genes, enzymes metabolizing molecular oxygen (FAD monooxygenases, FAD-dependent oxidases, catalases) and PTH11-type G-protein coupled receptors (Kulkarni *et al.* 2005) were significantly abundant among these 71 genes. Genes that were unaffected in the *Δlae1* strain but up-regulated in lae1OE included five of the eight class two class II hydrophobins of *T. reesei* (Druzhinina *et al.* 2012), genes for iron uptake and again PTH11 receptors. Genes encoding ankyrins [a 33-residue motif that mediates molecular recognition via protein–protein interactions and that has been shown to be involved in pathogenesis and endosymbiosis in bacteria (Breeuwer and Jacobs 1996)], enzymes involved in reactions with molecular, and also some other genes for iron uptake were abundant among those that were down-regulated in both *Δlae1* and *lae1OE* (Table 2). The PTH11 and HET genes were chosen to validate the array data, indicating good correlation between array and quantitative PCR results (Table S3).

**Table 2:** Grouping of LAE1-dependent differentially expressed genes by functional domains

	No. in Genome	%	*Down/Up*	*%*	*N/Up*	*%*	*Down/down*	*%*
Total number	9143		71		1113		372	
Ankyrins	21	0.22	0		4	0.35	6	1.6
Glycosyl hydrolases	200	2.2	27	38	28	2.5	13	3.4
Cytochrome P450	22	0.76	0		14	1.25	6	1.6
Glutathione-*S*-transferases	38	0.4	0		5	0.45	3	0.8
HET (heteroincompatibility genes)	22	0.24	2	2.8	2	0.17	1	0.26
Hydrophobins	8	0.08	0		5	0.44	0	
Iron uptake	12	0.13	0		4	0.36	2	0.53
Metabolism	1888	20.6	2	2.8	55	4.9	49	13.1
Mitochondrial biogenesis	186	2	0		12	1.1	0	
Transporters of the major facilitator superfamily	220	2.4	2	2.8	13	1.1	24	6.4
Multidrug transporters	26	0.28	0		7	0.63	4	1.1
Orphan genes	1012	11	7	9.8	71	6.3	25	6.7
Enzymes of O_2_ metabolism	75	0.82	2	2.8	12	1.1	11	2.9
PKS and NRPS	23	0.25	0		4	0.35	1	0.25
Proteases	383	4.1	1	1.4	22	1.9	4	1
PTH11*^a^*	24	0.26	1	1.4	6	1.05	1	0.25
Ribosome biogenesis	339	3.7	0		23	2	0	
Protein secretion	313	3.4	1	1.4	16	1.4	0	
Small secreted cysteine-rich proteins	174	1.9	3	4.2	52	4.6	3	0.8
Transcription factors	227	2.4	2	2.8	13	1.1	22	6.3
Unknown proteins	3535	38.7	12	16.9	445	40	111	29.8

The number of genes with polyadenylated transcripts that change in the *T. reesei* transcriptome when *lae1* expression is changed were compared with the total number of genes in the genome and ordered according to three expression patterns shown in Table 1. Percentage values printed in bold are significantly different to the percentage of these genes in the genome (*P* < 0.05); values were rounded to the nearest decimal.

a G-protein coupled receptors typified by PTH11, a cell-surface integral membrane protein required for pathogenicity in *Magnaporthe grisea* (Kulkarni *et al.* 2005).

### *Lae1* overexpression has a greater impact than *lae1* loss on secondary metabolite gene expression in *T. reesei*

**Figure 1:**
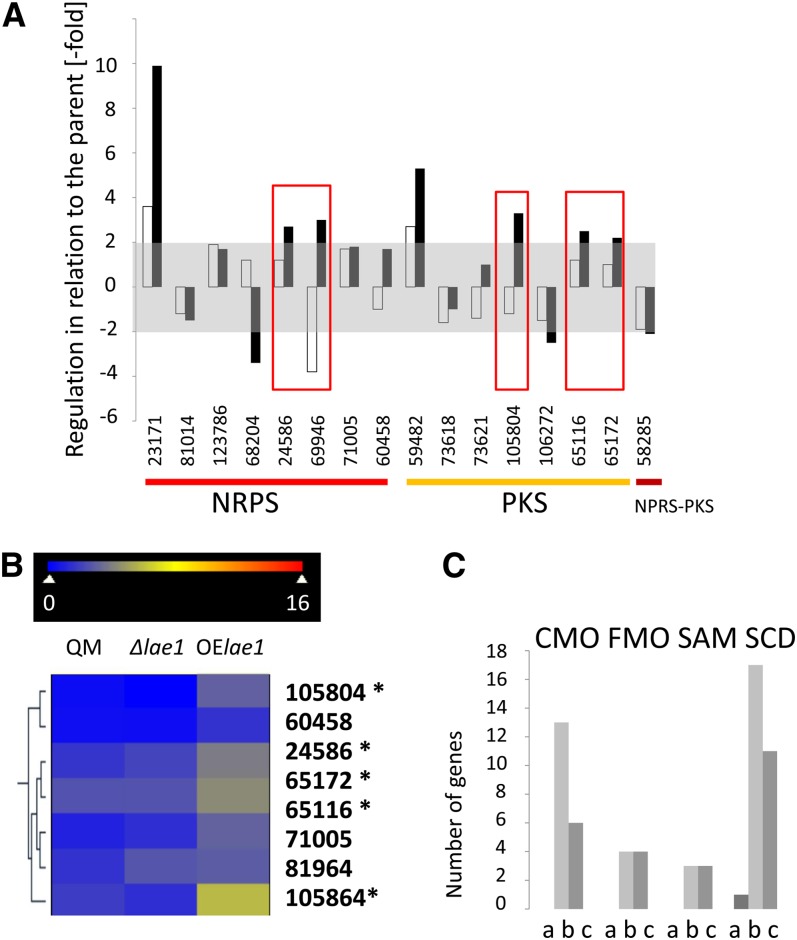
Effect of modulation of *lae1* gene expression on the expression of secondary metabolism genes. (A) Ratios of increased or decreased expression in *Δlae1* (white bars) and *lae1OE* (black bars), relative to the parent strain. The gray area indicates ± twofold, which is not considered significant. Genes are indicated by the Trire2 accession numbers (http://genome.jgi-psf.org/Trire2/Trire2.home.html) and are ordered as NRPS, PKS, and NRPS-PKS (indicated below the numbers). Only genes with *P* < 0.05 are shown. The red boxes surround genes that are likely regulated by LAE1, as described in the text. (B) Excerpt of a hierarchical cluster analysis of PKS and NRPS gene expression showing a cluster that contains all the significantly regulated genes. Data are shown as a heat map, and the color code of respective expression values (dark blue: 0; dark red: 16; numbers indicate the log_2_ of the mean expression level, n = 2) (C) Number of genes putatively involved in secondary metabolism that occur in one of the groups significantly regulated by LAE1 (a, down-regulated in *Δlae1* and up-regulated in *lae1OE*; b, unaffected in *Δlae1* but up-regulated in *lae1OE*; c, down-regulated in both). CMO, cytocrome P450cytochrome p450 monooxygenases; FMO, FAD monooxygenases; SAM, *S*-adenosyl-methionine-dependent methyltransferases; and SCD, short-chain dehydrogenases/reductases.

The *T. reesei* genome contains 10 genes for nonribosomal peptide synthases (NRPS), 11 polyketide synthase (PKS) genes, and two NRPS/PKS fusion genes (Martinez *et al.* 2008; Baker *et al.* 2012). In contrast to results from genome-wide expression analysis in *Aspergillus fumigatus* (Perrin *et al.* 2007), *A. flavus* (Georgianna *et al.* 2010), and *Fusarium verticillioides* (Butchko *et al.* 2012), that revealed decreased expression (60–80%) of PKS and NRPS genes in LaeA loss-of-function mutants, a much smaller portion of these genes is affected in *T. reesei* by LAE1 perturbation. As shown in Figure 1A, 16 of these 23 (eight NRPS and eight PKS genes) were significantly expressed under the present conditions, but only seven of them [Trire2:105804, a nonreducing PKS of clade III; Trire2:65172, a reducing PKS of the lovastatin clade, and Trire2:65116, a reducing PKS of the fumonisin clade (Baker *et al.* 2012)] and a single NRPS (Trire2:24586, encoding one of the two siderophore synthases) were up-regulated in the *lae1OE* strain. Note that these four genes were essentially not expressed in the parent and the *Δlae1* strain and thus may generate cryptic secondary metabolites (Figure 1B). In addition, the second siderophore synthase (Trire2:69946) was down-regulated in both mutants. Furthermore, one NRPS [Trire2:23171 paracelsin synthase (Neuhof *et al.* 2007)] and one PKS [Trire2:59482; a reducing t-toxin like PKS from clade III (Baker *et al.* 2012)] were significantly up-regulated but in the *lae1OE* but also *Δlae1* strain. None of the aforementioned PKS genes or their products has as yet been characterized. Other secondary metabolites and the genes involved in their synthesis pathways are not known from *T. reesei*. However, we noticed a high number of genes encoding cytochrome p450 monooxygenases, flavin-dependent monooxygenases, short chain dehydrogenase/reductases, and methyltransferases to be up-regulated in the *lae1OE* strain, and an approximately similar number of other genes for the same type of enzymes to be down-regulated in both mutant strains (Figure 1C, Table 2). These genes may be involved in secondary metabolite biosynthetic pathways.

### Perturbation of LAE1 expression does not correlate with changes in histone H3 methylation patterns

LaeA has been proposed to counteract histone H3 lysine 9 trimethylation (H3K9me3) in the sterigmatocystin gene cluster of *A. nidulans* (Reyes-Dominguez *et al.* 2010). To begin to investigate whether LAE1 influences the lysine methylation status of H3 in *T. reesei*, we performed ChIP-seq with antibodies against histone modifications known to be associated with transcriptionally active (H3K4me2 and -me3) or silent (H3K9me3) chromatin on the parental, the *Δlae1* and the *lae1*OE strain. Under the conditions used, of the 9143 predicted genes in *T. reesei*, 4089 were significantly associated with at least one of the three methylation marks in at least one of each of the three strains (Table S4). Of these, only 993 of these 4089 genes (24%) showed significant regulation as measured by the perturbation of LAE1 (Table 3). To test the default hypothesis that LAE1 would counteract H3K9 methylation, we screened the 993 genes for those that are methylated at H3K9 in the *Δlae1* strain but not in the parent and the *lae1OE* strain. Only three such genes were found (Trire2:66927, Trire2:53452, and Trire2:41942) and all encode unknown proteins. H3K9me3 constituted only a small portion of the LAE1-regulated genes (40 in total), and 31 of these were found to be methylated in all three strains. As for H3K4me2 and -me3, 557 genes showed both methylation marks in all three strains (WT, *Δlae1*, and *lae1OE*) and were thus also LAE1-independent.

**Table 3:** Summary of H3K4 di- and trimethylation patterns for genes that are not associated with methylated H3K4 in *Δlae1* but are associated with methylated H3K4 in at least one other strain

H3K4me2			H3K4me3			Regulation (*Δlae1/lae1OE*)				
WT	*Δlae1*	*lae1OE*	WT	*Δlae1*	*lae1OE*	Down/up	N/up	Down/down	N/down	Up/up
+		+	+		+		1	7	1	
+		+			+	1	1	1	2	
+		+				2	5	5	9	1
+			+		+			1		
+							1	5	3	
			+		+		6	13	12	1
		+	+		+	1				1
					+			2	2	11
		+			+	1				
			+				2	4	9	2
		+					10	6	8	11
			In total:			4*^a^*	26	44	46	27

+ indicates the presence of methylation in this strain, no symbol indicates absence; numbers specify the number of genes that show the respective pattern [up or down before the slash refers to regulation in *Δlae1 vs.* parent strain (WT), whereas right of the slash refers to regulation in the *lae1OE vs.* WT strain; N indicates no significant regulation (<twofold in either direction)]. WT, wild type.

a *lae1* not included.

We therefore screened the remaining 430 genes for those that would bear either di- or trimethylation, or both, on H3K4 in the parent and/or the *lae1OE* strain but not in the *Δlae1* strain. We found 148 genes that met these criteria (Table 3). Of these, only four genes showed the expected down-regulation in the *Δlae1* strain and up-regulation in the *lae1OE* strain if the same paradigm would hold for *T. reesei* as for *A. nidulans*. We found 26 genes that were up-regulated in the *lae1OE* strain only, and 44 genes that were down-regulated in both strains. There was no methylation pattern that clearly correlated with any of these three patterns of gene regulation. Annotation of these 74 genes showed that they comprised 17 genes of unknown function and six orphan genes known only from *T. reesei*, four genes encoding CAZymes, four genes encoding transcription factors, and three genes for permeases of the major facilitator superfamily (Table S5). None of the gene families that was enriched for LAE1 regulation (Table 2) was particularly abundant among these 74 genes. ChIP-seq results on selected targets were validated by region-specific PCR and results were similar, *i.e.*, no enrichment differences for any of the histone marks in the three different strains in any of the regions tested [data not shown (Seiboth *et al.* 2012)].

### Functional *T. reesei lae1* does not complement an *A. nidulans ΔlaeA* strain

**Figure 2:**
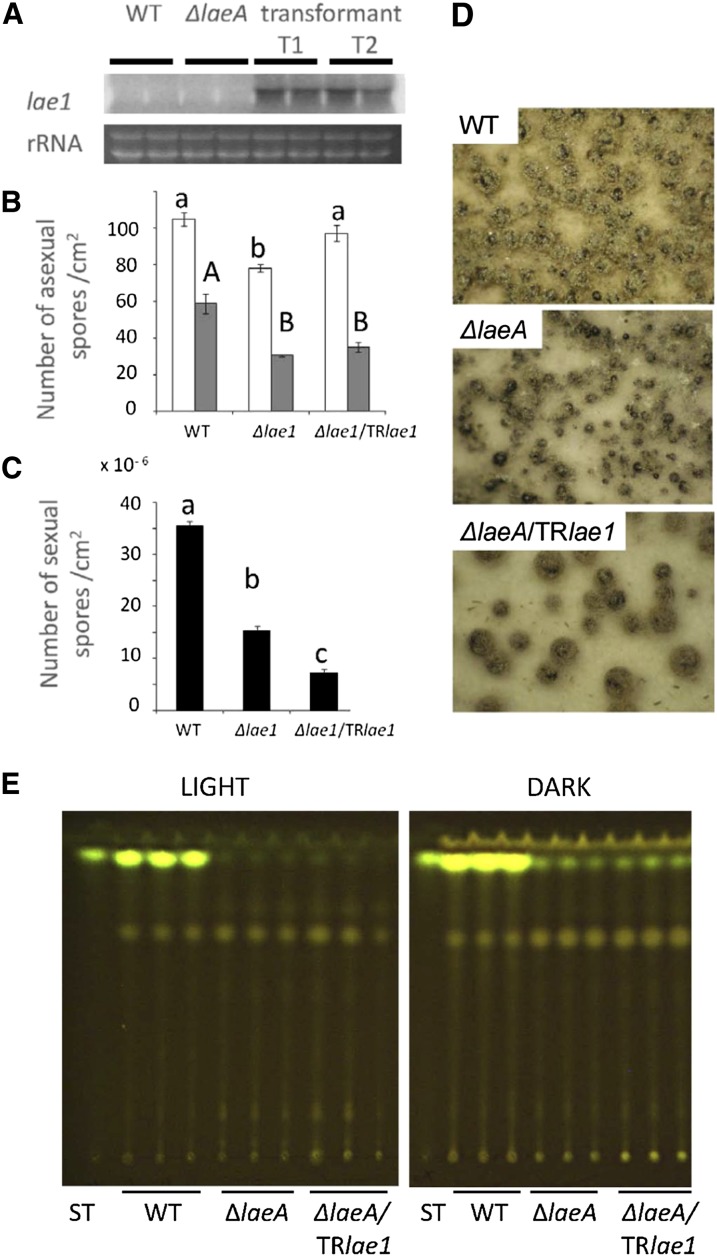
Effects of introducing *T. reesei lae1* in *A. nidulans ΔlaeA*. (A) Northern blot demonstrating expression of *Trlae1* mRNA in two *A. nidulans* transformants (T1, T2). (B) Overexpression of *lae1* leads to an impairment of asexual spore formation in a Δ*laeA* background. Light bars are strains grown in light, and dark bars are strains grown in dark. (C) Overexpression of *lae1* reinforces the impairment of sexual spore formation in a Δ*laeA* background. For both panels B and C, letters indicate statistically significant differences (*P* < 0.05) for each strain at different population levels according to the Tukey-Kramer multiple comparison test. Error bars show standard deviations of the results of three replications. (D) Sexual development of the WT, the Δ*laeA* mutant, and a *ΔlaeA*/TRlae1 strain on Champs medium in the dark for 5 d. (E) LAE1 does not complement sterigmatocystin production in a *laeA* mutant of *A. nidulans*. Wild-type (WT), the *ΔlaeA* mutant, and a *lae1* transformant *ΔlaeA*/TRlae1 were grown on GMM solid media in either light or darkness for 5 d at 37° and extracts. Extracts were separated on a TLC plate. Extraction was triplicated. Each strain was extracted twice. Norsolorinic acid (orange) and sterigmatocystin (yellow) were visualized using long-wave (254 nm) UV light. A sterigmatocystin standard (ST) was spotted on left side of the plate. WT, wild type.

A phylogeny of LaeA/LAE1 amino acid sequences resulted in a tree that resembles the species tree, indicating that LAE1 is the true ortholog of LaeA (Seiboth *et al.* 2012) and suggesting that Lae1/LAE1 is conserved among fungi. To learn whether it is also functionally conserved, we transformed an *A. nidulans ΔlaeA* mutant with the *T. reesei lae1* gene. Northern analysis showed that *lae1* was transcribed in the positive transformants (Figure 2A). Mycelial growth of *A. nidulans* was unaffected by *laeA* loss-of-function, and also the introduction of *T. reesei lae1* into the Δ*laeA* mutant had no effect (data not shown). Expression of *T. reesei lae1* in *A. nidulans* partially recovered the decrease in asexual sporulation in light but not in the dark (Figure 2B). Interestingly, *lae1* expression further decreases the formation of sexual spores (Figure 2C), which was correlated with a decrease in cleistothecia/unit area than both WT and *ΔlaeA* (Figure 2D). Those cleistothecia that were formed were abnormally large. In addition, *T. reesei lae1* was unable to restore sterigmatocystin production in *A. nidulans* (Figure 2E). Taken together, these data suggest that LAE1 cannot complement an *A. nidulans ΔlaeA* mutant and even appears to interfere with its sexual development.

### *T. reesei* LAE1 does not interact with *A. nidulans VeA*

As outlined previously, LaeA is part of the heterotrimeric velvet complex, which is assembled in the nucleus in the dark and additionally contains the VeA (velvet) protein, a regulator of morphogenesis and secondary metabolism in some filamentous fungi (Bayram and Braus 2011), and the VeA-related developmental regulator VelB (velvet-like B). VelB interacts with the N-terminus of VeA, whereas LaeA interacts with the C-terminus of VeA (Bayram *et al.* 2008a). We therefore hypothesized that the inability of LAE1 to replace LaeA may be due to a lack of interaction of *T. reesei* LAE1 with *A. nidulans* VeA.

**Figure 3:**
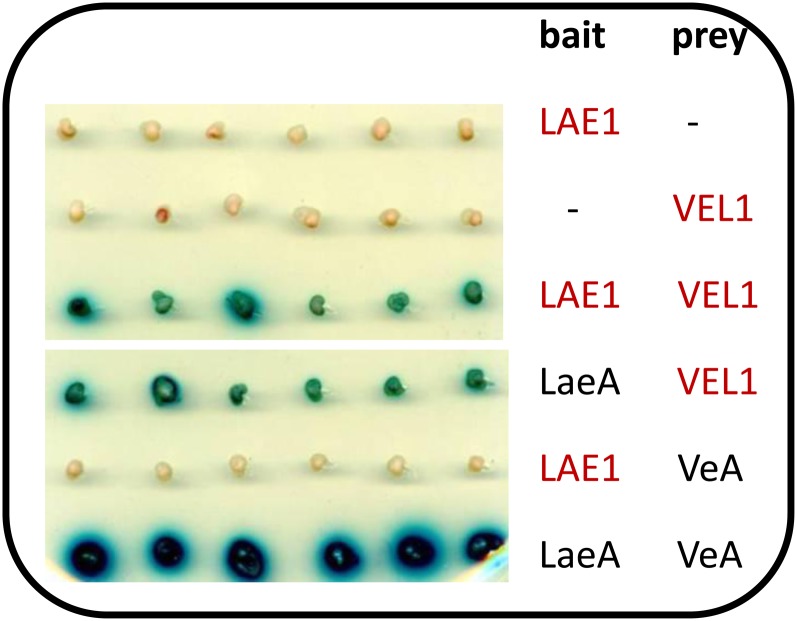
Interactions among LaeA, VeA, LAE1, and VEL1 in a yeast two-hybrid assay. Derivatives of yeast strain L40 expressing the different bait and prey fusion proteins were selected on -UTL (-leu, -trp, -ura) containing 2% (w/v) glucose (SD) media. Six transformants of each combination were tested for their coloration on -UTL medium containing X-Gal for β-galactosidase activities. *Trichoderma* proteins are marked in red.

To test this, we used a yeast two-hybrid system using the *T. reesei* and *A. nidulans* LAE1/LaeA proteins, respectively, as “bait,” and the *T. reesei/A. nidulans* VEL1/VeA proteins as “prey.” LaeA and VeA and LAE1 and VEL1 clearly interacted with each other, as did LaeA and VEL1 (Figure 3). However, in this assay *T. reesei* LAE1 was unable to interact with *A. nidulans* VeA. We therefore conclude that the inability of LAE1 to complement the Δ*laeA* strain may be caused by the inability of LAE1 to interact with the *A. nidulans* VeA protein.

### LAE1 is necessary for *vel1*—but not *vel2*—gene expression

In *A. nidulans*, expression of three velvet-like genes, *veA*, *velB*, and *vosA* are enhanced in a *ΔlaeA* mutant (Bayram and Braus 2011). To test whether this regulation is conserved in *T. reesei*, we used quantitative PCR to quantify the expression of the *veA* ortholog *vel1* (Trire2: 122284) in both wild-type and *Δlae1* strains. In contrast to *A. nidulans*, we found the transcript of *vel1* to be strongly down-regulated in *Δlae1* (29% of the control, *P* = 0.043) and to be unaffected in *lae1*OE (103%; *P* = 0.022). Inspection of the normalized microarray data revealed no effect on the putative *vel2* (Trire2:40551), and *vos1* (Trire2:102737) orthologs of *velB* and *vosA*, respectively (differences <15% in either strain, *P* < 0.05).

### Protein kinase A negatively regulates *lae1* expression

Because the aforementioned regulatory influences are different from those detected in *A. nidulans*, we also tested whether the regulation of *lae1* gene expression was similar to that reported for in *A. nidulans* (Bok and Keller 2004). In the latter fungus, *laeA* was shown to be subject to inhibition by protein kinase A (PkaA), suggesting a potential role of cyclic adenosine monophosphate (AMP). We therefore investigated the *lae1* transcript abundance in *T. reesei* strains modulated in the catalytic subunit of protein kinase A (PKA1), adenylate cyclase, and the G-protein GNA3, using *Δpka1* and *Δacy1* strains (Schuster *et al.* 2012) and a strain bearing a constitutively activated allele of *gna3*, which causes accumulation of cyclic AMP (Schmoll *et al.* 2009). Loss of function in PKA1 strongly enhances *lae1* transcript abundance (5.2 [± 1.3]-fold; *P* < 0.05), indicating similar regulation as found in *A. nidulans*. However, neither the adenylate cyclase mutant *Δacy1* nor the constitutively activated *gna3* allele showed a significant effect (0.7 [± 0.5]-fold, and 1.8 [± 0.9]-fold; respectively). We conclude that PKA1 is a negative regulator of *lae1* expression in *T. reesei*, whereas the role of cyclic-AMP is unclear. Opposite effects of *Δpka1* and *Δacy1* on expression of cellulase genes in *T. reesei* have been noted earlier (Schuster *et al.* 2012).

## Discussion

*T. reesei lae1* was originally identified by a phylogenetic approach (Seiboth *et al.* 2012), and by the synteny of its chromosomal locus in *Fusarium* and *Trichoderma* (Christian P. Kubicek, unpublished data). The latter approach was useful as many filamentous fungi express several LaeA homologs (Christian P. Kubicek, unpublished data; Jiang *et al.* 2011). We consider this important because some of the present results, such as lack of complementation of *A. nidulans*, and the small effect of *lae1* knock out on PKS and NRPS gene expression, however, may cast doubt on the identity of LAE1 as a true ortholog of the LaeA proteins characterized from other fungi, *e.g.*, *A. nidulans*, *A. fumigatus*, *A. flavus*, *P. chrysogenum*, *F. fujikuroi*, *F. verticillioides*, and *C. heterostrophus* (Bok and Keller 2004; Bok *et al.* 2005; Amaike and Keller 2009; Kosalkova *et al.* 2009; Hoff *et al.* 2010; Wiemann *et al.* 2010; Butchko *et al.* 2012; Wu *et al.* 2012). In fact, besides the loss of or enhancement of sporulation in the Δ*lae1* and *lae1*OE strains (Seiboth *et al.* 2012), respectively, which is largely conserved in many of the aforementioned taxa, and the demonstration of interaction with LAE1 with *T. reesei* VEL1, other findings seem to differ from those obtained with other fungi.

**Figure 4:**
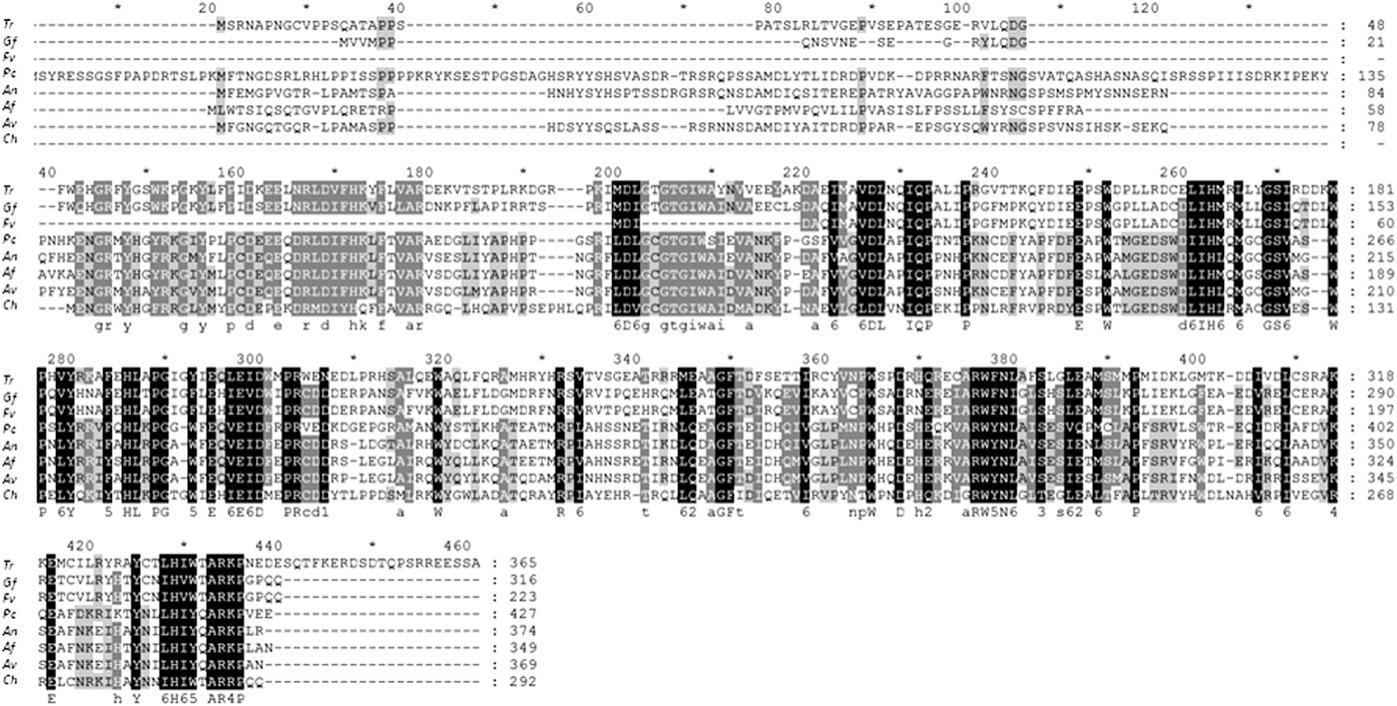
Alignment of published LaeA/LAE1 sequences. Abbreviations and accession numbers: *Tr*, *T. reesei* AFK30952.1; *Gf*, *G. fujikuroi* CBE54370.1; *Fv*, *Fusarium verticillioides* FVEG_00539 (not deposited; sequence taken from http://www.broadinstitute.org/annotation/genome/fusarium_group/MultiHome.html); *Pc*, *P. chrysogenum* ACD50375.1; *An*, *A. nidulans* AAQ95166.1; *Af*, *A. fumigatus* AAR01218.1; *Av*, *A. flavus* AAX68412.1; and *Ch*, *C. heterostrophus* AEP40318.1.

One of the most striking differences was the inability of *lae1* to complement an *A. nidulans ΔlaeA* strain. Because *laeA* was well expressed in *A. nidulans*, this could be due to incorrect translation or subtle differences in the aa structure of LaeA and LAE1. The interaction studies demonstrate that this may be due to the lack of ability of LAE1 to interact with VeA, and effects observed by LAE1 in the *A. nidulans ΔlaeA/TrLae1* transformants may thus be interpreted as being caused by an excess of unbound LAE1. This finding was puzzling because complementation of *A. nidulans ΔlaeA* was successful with Lae1 from *F. fujikuroi* (Wiemann *et al.* 2011), which is phylogenetically closer to *T. reesei* than to *A. nidulans*. An alignment of the *T. reesei* LAE1 protein with that of other published LaeA/LAE1 proteins (Figure 4) shows an overall amino acid (aa) identity of 70% with *Fusarium* spp. Since the N-terminus of LaeA has been demonstrated to be involved in binding to VeA (Bayram *et al.* 2008a) differences in its structure could interfere with this binding. However, the N-terminus is generally poorly conserved between fungi, and we could not detect specific differences that would offer an explanation for the lack of binding between VeA and LAE1.

Another possible explanation of the impact of *lae1* on *A. nidulans—*as well as its function in *T. reesei*—could be related along lines of those observed for another recently described *laeA*-like putative methyltransferase, called LlmF, in *A. nidulans* (Palmer *et al.* 2012). Using a bioinformatics approach, 10 predicted methyltransferase proteins with similarity to LaeA were characterized in *A. nidulans* with regard development and secondary metabolism. One of these proteins, LlmF, was found to exhibit an opposite phenotype to *laeA* where overexpression of *llmF* decreased secondary metabolism and sexual development and deletion of *llmF* increased secondary metabolism. LlmF was found to redirect cellular localization of VeA, thus resulting in the aforementioned phenotypes. Possibly LAE1 could have some impact on VeA function in *A. nidulans* despite negative yeast two-hybrid results. It is thus possible that other *lae1*-like proteins in *T. reesei* may impact expression of secondary metabolite gene clusters. There is also precedence for this notion from some preliminary studies on “velvet-interacting proteins” in *F. graminearum* (Jiang *et al.* 2011).

LaeA has been identified as a global transcriptional regulator of secondary metabolism in *A. nidulans* (Bok and Keller 2004) and subsequent work in other Aspergilli (Bok *et al.* 2005; Georgiianna *et al.* 2010), *P. chrysogenum* (Hoff *et al.* 2010), *F. fujikuroi* (Wiemann *et al.* 2010), *F. verticillioides* (Butchko *et al.* 2012), and *C. heterostrophus* (Wu *et al.* 2012) has lend credence to this idea. In *A. fumigatus*, expression of 13 of the 22 of the gene clusters for secondary metabolite synthesis was significantly down-regulated in *ΔlaeA* (Perrin *et al.* 2007). An even stronger effect was observed in *F. verticillioides*, where the transcription of 14 of 16 PKS genes was affected in a *lae1* mutant (Butchko *et al.* 2012). In *T. reesei*, in contrast, only five of the total 23 PKS and NRPS genes were affected in the *Δlae1* strain. Although we cannot exclude that the conditions for growth of *T. reesei* in this study were not those that best favor secondary metabolite gene expression, 10 of the 23 genes were at least moderately expressed in the parental strain. Interestingly, the seven genes that were up-regulated in the *lae1OE* strain were all characterized by a very low expression level in the parent strain, which would be compatible with assuming that overexpression of LAE1 may activate silent genes (Bok *et al.* 2006b). Nevertheless, the lower number of PKS and NRPS genes that are affected by LAE1, compared with other fungi, may suggest that regulation of their biosynthesis is not a major target of LAE1 in *T. reesei*. In *A. fumigatus*, an additional 38% of all cytochrome p450 monooxygenase genes also showed differential expression in *ΔlaeA*, and 15 of them were located in secondary metabolite clusters (Perrin *et al.* 2007). This compares well to *T. reesei*, where we detected LAE1-dependent expression of 20 from a total of 70 cytochrome P450 monooxygenases. However, it is unknown whether they are indeed involved in secondary metabolite synthesis in *T. reesei* or fulfill other functions such as detoxification (Druzhinina *et al.* 2012). Only six cytochrome P450 monooxygenases were found among the 203 genes down-regulated in the *Δlae1* mutant of *F. verticillioides* (Butchko *et al.* 2012).

The precise molecular function of LaeA/LAE1 and its mechanism of action remain enigmatic. Complex formation with VeA and VeB has been demonstrated, but its precise contribution to the regulation of secondary metabolism gene clusters is not yet known, even in *Aspergillus* spp. In *A. nidulans*, LaeA somehow counteracts trimethylation of H3K9 and binding of the homolog of Heterochromatin Protein 1 (HP1/HepA) to this repressive chromatin mark (Strauss and Reyes-Dominguez 2011). Based on results presented here, this model is unlikely to be true for *T. reesei* as among a total of 1021 genes responding to modulation of *lae1* function, H3K9me3 was absent from most genes in the Δlae*1*strain, and the methylation pattern of the 13 genes that indeed exhibited H3K9 methylation was independent of LAE1. Absence of H3K9me3 from chromatin associated with *bona fide* genes is also the default state in *N. crassa*, where heterochromatic histone modification marks are almost exclusively associated with transposon relics (Tamaru and Selker 2001; Tamaru *et al.* 2003; Lewis *et al.* 2009, Smith *et al.* 2011). Conversely, only 74 genes exhibited H3K4 methylation patterns that would be consistent with a role of LAE1 in H3K4 methylation, and the FunCat composition of the protein encoded by this gene sample was random. Based on our data, we conclude that LAE1 is not involved in the methylation of H3K4 or H3K9 in *T. reesei*. Currently, the evidence for direct histone methylation by LaeA/LAE1 is at best sparse. No true substrate has been identified yet. None of the LaeA homologs share domains found in many protein methyltransferases that specifically act on histones, *e.g.*, SET domains found in Clr4/DIM-5/Suvar3-9, or motifs found in Dot1 (Sawada *et al.* 2004; Adhvaryu *et al.* 2005). LaeA/LAE1 shares some short regions of similarity with arginine methyltransferases. Arginine methylation of histone tails can promote or prevent the docking of transcriptional effector molecules, and in this way influence the expression of gene clusters and regulons (Di Lorenzo and Bedford 2011). Two equally likely scenarios may involve the use of LaeA/LAE1 as a shuttle for methyl groups to other proteins in the velvet complex, based on the requirement of the SAM binding motif in *Aspergillus* LaeA (Bok *et al.* 2006a, Bayram and Braus 2011), or the possibility that LaeA/LAE1 methylate proteins in the velvet complex. However, we also cannot rule out other possible indirect effects of LAE1 such as on acetylation or on general nucleosome arrangements known to impact gene expression in fungi. For example, the histone acetyltransferase EsaA was recently shown to activate secondary metabolite gene clusters through H4K12 acetylation in *A. nidulans* (Soukup *et al.* 2012).

LaeA, in combination with the velvet family of related regulatory proteins, may be involved in supporting the development of progeny in Aspergilli, by controlling the production of chemicals to protect fruiting bodies and the production of nourishing cells for developing fruiting bodies (Sarikaya Bayram *et al.* 2010). Based on our current findings, we adapt this interpretation for *T. reesei*. This species is unique in the genus because it has escaped its evolutionary history as a mycoparasite to become a successful competitor in the use of predegraded wood (Druzhinina *et al.* 2011). Obviously, this lifestyle profits more from an efficient arsenal of (hemi)cellulases and proteases, and transporters for the respective hydrolysis products, than from excessive toxification of its competitors. In this study we identified LAE1-dependent expression of PTH11 G-protein-coupled receptors, heterokaryon incompatibility proteins, hydrophobins, iron uptake, and unknown proteins bearing ankyrin motifs (see Table 2). Regulation of iron uptake and hydrophobin formation by LaeA has also been detected in *A. fumigatus* (Perrin *et al.* 2007) and *A. flavus* (Georgianna *et al.* 2008, Chang *et al.* 2012), and genes encoding enzymes reacting with molecular oxygen are also present in the LaeA/LAE1-dependent transcriptomes of *A. fumigatus*, *A. flavus*, and *F. verticillioides* (Perrin *et al.* 2007; Georgianna *et al.* 2008; Butchko *et al.* 2012) but all the other gene categories are a specific response of *T. reesei*. As all these genes function in the response of the fungus to environmental stimuli and the presence of competing organism, we interpret this such that LAE1 is involved in successful establishment of *T. reesei* in its environment.

## Supplementary Material

Supporting Information
