# Supplementary material for: Functional Analyses of Trichoderma reesei LAE1 Reveal Conserved and Contrasting Roles of This Regulator
Source: G3 (Bethesda). 2013 Feb 1;3(2):369–78. doi: 10.1534/g3.112.005140 (PMC3564997; doi:10.1534/g3.112.005140)
Supplement: Supporting Information [file supp_3_2_369__index.html]

Supporting Information 

# Functional Analyses of *Trichoderma reesei* LAE1 Reveal Conserved and Contrasting Roles of This Regulator

## Supporting Information for Karimi-Aghcheh *et al.*, 2013

**Files in this Data Supplement:**

- Supporting Information - Tables S1-S5 (PDF, 4 MB)
- Table S1 - Primers used for qPCR in this study (PDF, 66 KB)
- Table S2 - Genes differentially expressed in *T. reesei* strains modulated in lae1 function (PDF, 1 MB)
- Table S3 - Comparison of expression results from microarrays and qPCR for selected genes (PDF, 61 KB)
- Table S4 - Global H3K9 and H3K4 methylation patterns in relation to regulation by LAE1 (PDF, 3 MB)
- Table S5 - A few genes are associated with H3K4 methylation, which would be consistent with a positive role of LAE1 in H3K4 methylation. (PDF, 91 KB)
