# Supplementary material for: Functional Analyses of Trichoderma reesei LAE1 Reveal Conserved and Contrasting Roles of This Regulator
Source: G3 (Bethesda). 2013 Feb 1;3(2):369–78. doi: 10.1534/g3.112.005140 (PMC3564997; doi:10.1534/g3.112.005140)
Supplement: Supporting Information [file supp_3.2.369_005140SI.pdf]

G3 Genes | Genomes | Genetics

Functional analyses of Trichoderma reesei LAE1 reveal conserved and contrasting roles of this regulator

[1]Razieh Karimi-Aghcheh, [2]Jin Woo Bok , [3]Pallavi A. Phatale, [3]Kristina M. Smith, [4]Scott E. Baker, [1]Alexander Lichius, [1]Markus Omann, [1]Susanne Zeilinger, [1,5]Bernhard Seiboth, [2]Cathy Rhee, [2]Nancy P. Keller, [3]Michael Freitag and [1,5,*]Christian P. Kubicek

1 Institute of Chemical Engineering, Research Division Biotechnology and Microbiology, TU Wien, Austria

2 Department of Medical Microbiology and Immunology, University of Wisconsin-Madison, USA.

3 Department of Biochemistry and Biophysics, Oregon State University, USA.

4 Fungal Biotechnology Team, Chemical and Biological Process Development Group, Pacific Northwest National Laboratory, Richland, WA 99352, USA.

5 Austrian Center of Industrial Biotechnology, c/o Institute of Chemical Engineering, TU Wien, Austria

* corresponding author: (tel) +43 1 58801 166 500; (fax) +43 1 58801 172 99;

ckubicek@mail.zserv.tuwien.ac.at

Table S1 Primers used for qPCR in this study

Gene	sequence (primer forward 5´→3´)	sequence (primer reverse 5´→3´)	primer efficiency (%)	Tm (°C)
Gene	primer efficiency (%)	Tm (°C)	100	57.2
vel1	AGCCTTATGTGCCTCACT	GCAGGAGCAGAGTAGTTG	100	57.2
lae1	ACTGGAGATTGACTGGATGC	TTCTGCGTCTGGTAGCCTC	100	62.9
Trire2:23171	GGTGGCTGTAGATGTGTT	TGAGAGTCGTCGTTGAAG	100	57.2
Trire2:112083	GGTGAAGAGATACGGTAT	TGAGTTGGAGTAGATGTC	100	52.6
Trire2:109101	GAATCCAGTGGCATTCTATC	CGACCCTCAATAATATCATACC	100	57.1
Trire2:69187	GCCTCTACCAATGAACTC	CCGAGACAGGACATAATC	97.2	56.1
Trire2:107071	CGATTGCCGTCTACGATAT	GCTCAGTTGTCCAGTCAT	100	56.1
Trire2:111494	ATCAACCAGACAGACAAC	AGATATAGACTTGCTCACATAG	95	52.6
Trire2:122824	CGTATGGCACCATCATTGG	CTGGGAAGACTCGTCGGAG	100	64
Trire2:39587	CTTGACAACCCGCCAAACAG	CGATGGAGTAAAAGACACCG	87.5	62
Trire2:82041	CTCATCGTCTTCCCCATTC	CATGCTTGTTGTTGTGCCCTG	100	62
Trire2:69904	CGGGAGACATTTCGCCTTTATC	CAGAAGCAGGTCGCAGAGTAG	86	64
Trire2:5647	CTACAACCCCGATAACCTC	CGTGGAAGATGCGGTAGTAC	98.5	60
Trire2:110744	CCTCTACGCCTCGTCAATC	CAATGGCGGAGAAGAAGAAGC	85	62
Trire2:110339	GATGCCGCTGATTGCCAC	CGAGATCGGTGATGATGTCG	91	65
Trire2:62462	CACGAACCCAACGCTCCAAC	CGAAGTATGCCCACTCCAG	95.5	63
Trire2:121990	CTCAAGGTAGTTGCGGTG	CGTCATCTGGTGGCTGTTG	90	60
Trire2:76763	CGGCAGAGGTTGAGCAATAC	CGGTAACACGGAGGCGTTTC	89	64
Trire2:105224	CACCGCTTGCCATCATCTTC	CAAGAGGAATCCCAAGACAC	100	61
sar1	TGGATCGTCAACTGGTTCTACGA	GCATGTGTAGCAACGTGGTCTTT	64	64

Table S2 Genes differentially expressed in T. reesei strains modulated in lae1 function*

SEQ_ID	wild-type	S.D.	*Δlae1*	S.D.	*lae1OE*	S.D.	ratio *[Δlae1]*	ratio *[ae1OE]*	p-value	F- value	
1959	6.55	0.23	6.99	0.07	8.78	0.15	1.359 up	4.687 up	0.00	166.52	Haloacid dehalogenase-like hydrolase
1977	12.71	0.03	12.57	0.04	11.58	0.14	1.106 down	2.191 down	0.00	89.50	Peptidase_M28
1993	12.52	0.05	12.44	0.14	11.27	0.09	1.057 down	2.381 down	0.00	151.74	γ-glutamyl phosphate reductase GPR
2033	4.81	0.06	3.30	0.04	5.95	0.13	2.855 down	2.205 up	0.00	404.26	unknown protein
2036	6.24	0.02	6.11	0.09	7.75	0.16	1.090 down	2.856 up	0.00	142.94	cysteine peptidase
2071	9.68	0.07	10.58	0.09	10.86	0.09	1.875 up	2.266 up	0.00	123.87	unknown protein. 5 TM
2076	9.84	0.10	6.09	0.11	6.75	0.45	13.496 down	8.504 down	0.00	67.36	malate permease
2091	12.79	0.03	12.11	0.17	11.37	0.08	1.604 down	2.676 down	0.00	136.90	6-phosphofructokinase
2096	9.25	0.02	9.96	0.15	7.47	0.31	1.637 up	3.429 down	0.00	76.37	Zn2Cys6 transcriptional regulator
2125	12.16	0.01	12.35	0.05	11.13	0.10	1.146 up	2.029 down	0.00	188.64	Fructose-2.6-bisphosphatase
2148	13.15	0.02	12.06	0.21	11.74	0.24	2.133 down	2.651 down	0.00	30.59	Zn2Cys6 transcriptional regulator
2185	5.85	0.02	6.67	0.15	8.71	0.36	1.764 up	7.251 up	0.00	76.38	GCN5-related N-acetyltransferase
2223	8.84	0.12	9.08	0.13	10.23	0.22	1.178 up	2.618 up	0.00	45.43	succinate-CoA ligase. alpha subunit
2316	12.36	0.10	10.37	0.27	10.33	0.10	3.965 down	4.082 down	0.00	141.24	unknown protein with caleosin domain
2322	9.61	0.01	9.18	0.14	8.22	0.17	1.344 down	2.612 down	0.00	65.81	unknown protein
2358	9.66	0.06	9.32	0.03	8.12	0.28	1.269 down	2.915 down	0.00	40.58	unknown protein with HIT domain
2392	14.29	0.09	14.02	0.04	12.19	0.23	1.209 down	4.288 down	0.00	117.02	peroxisomal hydratase-dehydrogenase-epimerase
2399	11.59	0.01	11.51	0.10	9.90	0.08	1.056 down	3.210 down	0.00	457.97	unknown protein
2489	6.70	0.04	5.83	0.06	8.45	0.38	1.820 down	3.356 up	0.00	59.33	Ribonuclease T2
2492	10.31	0.17	9.44	0.02	8.18	0.24	1.832 down	4.378 down	0.00	78.04	Phosphatidylinositol-4-phosphate 5-kinase
2499	7.61	0.09	7.85	0.04	5.36	0.27	1.182 up	4.741 down	0.00	122.47	unknown protein
2517	4.72	0.08	4.23	0.05	5.82	0.41	1.399 down	2.149 up	0.01	18.41	Flavoprotein monooxygenase. putative
2526	13.57	0.02	13.29	0.14	12.56	0.14	1.215 down	2.010 down	0.00	50.83	Protein kinase C
2570	3.28	0.07	3.82	0.24	4.83	0.08	1.448 up	2.929 up	0.00	105.25	short chain dehydrogenase/reductase
2687	12.56	0.07	10.54	0.17	10.90	0.10	4.071 down	3.173 down	0.00	197.49	AAA ATPase
2703	11.87	0.14	11.97	0.04	10.62	0.24	1.074 up	2.372 down	0.00	41.84	unknown protein

2730	13.28	0.05	13.24	0.00	12.16	0.20	1.024 down	2.171 down	0.00	47.38	unknown protein
2745	12.92	0.02	12.76	0.13	11.80	0.12	1.115 down	2.175 down	0.00	91.93	fumarate hydratase-like protein
2835	11.99	0.01	11.83	0.01	10.80	0.21	1.112 down	2.275 down	0.00	44.84	unknown protein
3043	3.23	0.03	3.71	0.18	5.11	0.18	1.393 up	3.664 up	0.00	105.89	SSCRP
3049	13.72	0.39	12.80	0.01	10.85	0.08	1.898 down	7.341 down	0.00	181.03	methionine aminopeptidase
3055	12.48	0.15	7.97	0.14	11.17	0.12	22.757 down	2.485 down	0.00	651.11	short chain dehydrogenase/reductase
3094	5.60	0.12	5.26	0.11	7.17	0.67	1.265 down	2.986 up	0.02	11.46	GH30 glucan endo 1.6-β-glucanase
3121	3.35	0.46	3.82	0.25	4.97	0.12	1.379 up	3.066 up	0.00	31.51	cytochrome P450 oxidoreductase OrdA-like
3196	12.47	0.11	12.28	0.05	10.80	0.16	1.138 down	3.171 down	0.00	131.28	GH38 α-mannosidase
3262	3.61	0.03	4.01	0.01	5.31	0.21	1.315 up	3.244 up	0.00	86.89	succinate semialdehyde dehydrogenase (NADP)
3267	9.12	0.02	10.14	0.06	7.30	0.31	2.019 up	3.543 down	0.00	100.17	MRSP1/expansin-like
3350	11.77	0.18	12.11	0.24	10.76	0.31	1.265 up	2.002 down	0.01	18.73	phosphopantothenate-cysteine ligase
3364	11.25	0.06	10.43	0.11	9.12	0.17	1.768 down	4.386 down	0.00	156.98	Zinc-containing alcohol dehydrogenase
3422	6.69	0.30	6.91	0.15	8.76	0.41	1.166 up	4.207 up	0.00	31.17	unknown protein with oxidoreductase domain
3462	12.11	0.15	12.22	0.06	11.06	0.24	1.083 up	2.060 down	0.00	30.58	UreF urease accessory protein
3481	10.81	0.10	10.51	0.24	9.67	0.22	1.236 down	2.211 down	0.01	23.75	unknown protein
3488	11.29	0.09	9.77	0.05	9.93	0.21	2.876 down	2.578 down	0.00	55.13	unknown protein
3501	9.18	0.12	8.88	0.13	8.15	0.24	1.227 down	2.035 down	0.01	19.64	unknown protein
3529	10.62	0.10	11.31	0.32	11.99	0.16	1.613 up	2.586 up	0.00	33.87	unknown protein
3568	10.27	0.07	7.85	0.24	8.39	0.27	5.337 down	3.682 down	0.00	62.06	phospholipase A2
3579	7.68	0.00	7.74	0.19	9.71	0.27	1.046 up	4.104 up	0.00	79.80	ATP-dependent RNA helicases.
3596	7.86	0.25	7.31	0.09	8.90	0.43	1.470 down	2.050 up	0.01	14.87	uroporphyrinogen synthase
3605	11.24	0.02	10.78	0.07	9.96	0.17	1.377 down	2.431 down	0.00	67.96	Zn2Cys6 transcriptional regulator
3835	10.40	0.27	10.40	0.01	8.73	0.33	1.002 down	3.172 down	0.00	34.15	3-ketoacyl-CoA thiolase
3889	13.56	0.12	13.42	0.01	12.31	0.33	1.099 down	2.382 down	0.01	21.19	unknown protein
3909	9.25	0.02	10.02	0.11	10.58	0.44	1.710 up	2.528 up	0.03	10.22	Mitochondrial ribosomal protein L43
3914	10.64	0.03	7.12	0.09	6.78	0.89	11.485 down	14.492 down	0.01	22.27	Endoplasmic reticulum protein EP58
4040	11.23	0.04	10.80	0.00	10.10	0.21	1.353 down	2.187 down	0.00	35.16	RNA polymerase Rpc34 subunit
4064	11.58	0.07	11.92	0.04	13.18	0.09	1.267 up	3.025 up	0.00	345.65	Threonine dehydratase
4069	11.64	0.03	11.63	0.09	10.33	0.29	1.008 down	2.480 down	0.00	32.07	AMP-dependent synthetase and ligase

4109	9.86	0.05	10.13	0.00	8.71	0.20	1.204 up	2.228 down	0.00	68.66	Aspartate/tyrosine/aromatic aminotransferase
4114	6.26	0.36	3.21	0.04	5.03	0.19	8.272 down	2.337 down	0.00	96.40	FAD linked oxidase
4124	6.34	0.08	10.60	0.10	9.84	0.11	19.177 up	11.315 up	0.00	1042.30	myb transcriptional regulator
4171	9.30	0.15	6.01	0.14	7.97	0.19	9.788 down	2.519 down	0.00	177.10	calcium transporter
4240	11.22	0.06	8.06	0.05	8.02	0.08	8.960 down	9.188 down	0.00	1420.23	stress response protein Rds1; secreted
4308	12.63	0.13	11.85	0.03	11.62	0.17	1.717 down	2.021 down	0.00	32.90	Aminopeptidase N
4428	10.26	0.15	9.95	0.05	9.19	0.25	1.234 down	2.098 down	0.01	20.69	unknown protein. TPR domain
4442	8.19	0.00	6.96	0.11	6.43	0.27	2.342 down	3.378 down	0.00	43.60	SAM-dependent methyltransferase
4479	11.44	0.06	12.11	0.22	12.65	0.27	1.597 up	2.318 up	0.01	18.26	small nuclear ribonucleoprotein Sm D2
4494	12.22	0.06	11.93	0.08	10.60	0.35	1.224 down	3.085 down	0.00	29.62	unknown protein
4517	5.19	0.02	6.20	0.13	9.04	0.17	2.027 up	14.425 up	0.00	577.36	Cytochrome P450 CYP4/CYP19/CYP26 subfamilies
4561	9.77	0.08	8.32	0.02	8.41	0.19	2.718 down	2.569 down	0.00	64.60	GT α-1.6-mannosyltransferase
4626	13.27	0.26	11.88	0.16	10.95	0.44	2.627 down	5.010 down	0.01	26.50	unknown protein. C2 domain
4677	11.12	0.05	11.06	0.06	9.69	0.18	1.045 down	2.710 down	0.00	92.34	unknown protein
4726	12.92	0.10	12.89	0.11	11.52	0.24	1.022 down	2.629 down	0.00	47.56	Cytochrome P450 monooxygenase
4737	10.89	0.06	9.58	0.02	9.10	0.23	2.471 down	3.457 down	0.00	68.07	arylamine N-acetyltransferase 1
4748	12.15	0.14	12.09	0.11	11.00	0.16	1.042 down	2.213 down	0.00	57.75	C2H2 transcription factor
4847	10.92	0.10	10.62	0.14	9.76	0.10	1.229 down	2.232 down	0.00	83.73	unknown protein
4851	11.76	0.07	4.15	0.23	9.82	0.18	194.650 down	3.832 down	0.00	1034.06	unknown protein
4875	11.39	0.14	10.78	0.01	9.28	0.23	1.521 down	4.324 down	0.00	98.04	carboxy-cis.cis-muconate cyclase
4876	12.36	0.17	11.02	0.00	10.03	0.30	2.534 down	5.033 down	0.00	61.72	catechol dioxygenase
4905	6.27	0.39	8.16	0.27	7.78	0.52	3.713 up	2.852 up	0.03	10.27	unknown protein
4921	12.88	0.17	12.46	0.04	11.80	0.25	1.338 down	2.114 down	0.01	19.91	C2H2 transcriptional regulator
4941	12.34	0.04	11.96	0.00	9.37	0.39	1.301 down	7.840 down	0.00	83.15	phenylacetyl-CoA ligase.
4945	11.80	0.15	11.97	0.02	10.80	0.07	1.121 up	2.001 down	0.00	152.39	MAPKKK mitogen activated protein kinase kinase kinase Ste11
4950	9.73	0.04	9.36	0.00	8.62	0.21	1.291 down	2.153 down	0.00	35.56	urea transporter
4952	6.59	0.24	5.49	0.12	8.11	0.17	2.137 down	2.872 up	0.00	157.24	unknown protein
4999	9.24	0.39	13.25	0.25	12.64	0.23	16.108 up	10.569 up	0.00	133.02	cytochrome P450 monooxygenase
5000	5.37	0.36	6.35	0.04	7.11	0.55	1.974 up	3.348 up	0.03	9.95	MFS permease
5026	8.75	0.02	10.14	0.03	10.74	0.12	2.634 up	3.969 up	0.00	288.54	SSCRP

5063	12.65	0.04	11.93	0.09	11.16	0.25	1.647 down	2.797 down	0.00	39.15	unknown protein
5072	8.79	0.05	9.00	0.08	9.98	0.12	1.160 up	2.286 up	0.00	113.58	unknown protein
5084	11.91	0.06	9.34	0.08	7.62	0.51	5.928 down	19.550 down	0.00	79.21	conidiation-specific protein 10
5182	12.58	0.03	9.89	0.02	10.43	0.15	6.435 down	4.438 down	0.00	311.80	iron-dependent peroxidase
5196	11.13	0.00	11.14	0.03	9.23	0.28	1.006 up	3.749 down	0.00	78.40	C-type cyclin
5275	12.75	0.11	12.79	0.03	10.89	0.06	1.024 up	3.633 down	0.00	800.28	unknown protein with SET domain
5296	10.85	0.09	10.83	0.03	9.75	0.03	1.019 down	2.155 down	0.00	510.87	DHQase_I. Type I 3-dehydroquinase
5308	5.13	0.34	5.20	0.68	6.49	0.42	1.050 up	2.558 up	0.04	7.82	unknown protein
5319	10.23	0.01	9.42	0.04	8.19	0.09	1.747 down	4.094 down	0.00	585.84	unknown protein
5324	10.79	0.14	11.26	0.10	12.02	0.24	1.384 up	2.343 up	0.01	26.97	Ribosomal protein L14b
5330	4.77	0.01	4.76	0.25	5.91	0.25	1.011 down	2.195 up	0.01	26.91	glutathione S-transferase. putative
5366	11.28	0.01	11.29	0.01	9.92	0.63	1.008 up	2.555 down	0.04	7.87	O-methyltransferase. putative
5369	3.51	0.29	3.19	0.36	6.05	1.06	1.250 down	5.811 up	0.03	10.22	Metallocarboxypeptidase. putative
5530	12.84	0.14	12.11	0.14	11.36	0.21	1.651 down	2.780 down	0.00	42.34	unknown protein
5612	9.58	0.08	8.56	0.04	8.06	0.40	2.028 down	2.858 down	0.01	15.90	MFS permease
5651	8.16	0.04	6.47	0.38	6.42	0.29	3.222 down	3.328 down	0.01	27.94	unknown protein
5656	10.62	0.09	10.70	0.15	9.39	0.25	1.057 up	2.348 down	0.00	36.99	MFS permease
5675	12.39	0.08	12.21	0.12	11.31	0.10	1.130 down	2.106 down	0.00	98.52	C2H2 transcriptional regulator
5737	10.10	0.02	10.30	0.01	8.96	0.18	1.148 up	2.194 down	0.00	78.72	tyrosine/serine protein phosphatase. putative
5776	13.87	0.02	12.85	0.02	12.56	0.18	2.029 down	2.478 down	0.00	61.23	glucose-6-phosphate isomerase
5807	8.79	0.18	6.94	0.13	7.18	0.50	3.606 down	3.055 down	0.02	13.68	GH95 α-L-fucosidase
5818	9.88	0.07	8.82	0.05	8.84	0.10	2.086 down	2.049 down	0.00	117.46	DSBA oxidoreductase
5836	10.74	0.03	12.11	0.23	11.79	0.42	2.584 up	2.073 up	0.03	9.20	GH2 β-mannosidase
5888	4.26	0.17	3.77	0.42	5.31	0.25	1.400 down	2.069 up	0.01	22.83	fatty-acid amide hydrolase. putative
5889	10.37	0.45	9.36	0.23	9.26	0.33	2.014 down	2.156 down	0.04	7.69	amidase
5890	4.07	0.13	3.83	0.26	5.61	0.06	1.177 down	2.908 up	0.00	146.41	MFS permease
5912	11.49	0.12	11.19	0.13	10.44	0.18	1.225 down	2.059 down	0.00	34.11	unknown protein
5942	11.37	0.21	10.49	0.13	9.50	0.26	1.845 down	3.665 down	0.00	46.16	unknown protein
5970	11.51	0.07	9.33	0.06	8.77	0.16	4.533 down	6.710 down	0.00	286.94	Amidase
6085	3.77	0.07	5.01	0.27	4.80	0.05	2.362 up	2.041 up	0.00	55.44	Enoyl-CoA hydratase/isomerase. putative

6103	3.80	0.02	4.04	0.32	4.94	0.11	1.179 up	2.196 up	0.00	37.99	MFS multidrug transporter. putative
21152	11.01	0.14	11.46	0.12	12.59	0.29	1.364 up	2.991 up	0.00	33.69	unknown protein
21170	13.33	0.03	13.86	0.30	14.54	0.07	1.440 up	2.300 up	0.00	47.41	Ribosomal protein S12
21221	11.39	0.17	11.45	0.21	10.36	0.08	1.044 up	2.038 down	0.00	60.93	unknown protein
21288	12.68	0.04	12.73	0.04	11.55	0.20	1.035 up	2.182 down	0.00	53.68	Vacuolar segregation protein Pep7
21342	12.35	0.12	12.43	0.08	11.21	0.18	1.056 up	2.204 down	0.00	61.79	TUL1 Golgi-localized RING-finger ubiquitin ligase (E3)
21396	11.40	0.03	10.81	0.04	10.22	0.16	1.497 down	2.263 down	0.00	61.24	sugar isomerase
21412	13.97	0.17	12.89	0.11	12.97	0.12	2.118 down	2.000 down	0.00	50.22	unknown protein
21415	12.60	0.19	11.61	0.08	10.46	0.25	1.989 down	4.412 down	0.00	71.00	unknown protein
21416	13.17	0.08	12.41	0.03	11.47	0.13	1.698 down	3.251 down	0.00	165.59	unknown protein
21422	12.80	0.02	12.78	0.03	11.15	0.08	1.013 down	3.152 down	0.00	594.42	MFS permease
21461	12.27	0.07	11.92	0.15	11.27	0.25	1.270 down	2.003 down	0.01	17.47	ATP-dependent protease La. putative
21498	11.25	0.18	12.40	0.20	12.91	0.03	2.221 up	3.163 up	0.00	120.28	unknown protein
21505	12.87	0.18	12.46	0.11	11.57	0.15	1.330 down	2.467 down	0.00	54.90	G-protein alpha subunit 3 GNA-3
21571	13.26	0.11	12.93	0.09	12.24	0.21	1.253 down	2.019 down	0.01	25.09	unknown protein
21595	10.53	0.14	7.93	0.13	8.56	0.17	6.057 down	3.914 down	0.00	150.98	MFS permease
21758	14.86	0.16	15.08	0.02	13.36	0.20	1.165 up	2.832 down	0.00	92.43	isocitrate lyase
21817	12.81	0.05	11.87	0.19	11.47	0.18	1.918 down	2.520 down	0.00	43.31	unknown protein
21836	13.96	0.01	13.13	0.01	11.84	0.16	1.775 down	4.346 down	0.00	202.44	phosphoglucomutase/phosphomannomutase
21960	11.54	0.12	11.83	0.07	13.05	0.52	1.223 up	2.842 up	0.02	11.40	phospholipase C
22030	12.46	0.02	12.51	0.09	11.17	0.14	1.033 up	2.442 down	0.00	135.52	unknown protein
22035	12.17	0.07	11.40	0.19	10.92	0.14	1.702 down	2.376 down	0.00	49.85	unknown protein
22064	13.86	0.03	13.07	0.01	12.20	0.07	1.726 down	3.163 down	0.00	553.67	DNAJ heat shock family protein
22076	11.93	0.13	12.30	0.19	13.11	0.16	1.294 up	2.266 up	0.00	40.46	lysine decarboxylase
22093	12.88	0.10	11.88	0.05	10.15	0.19	2.004 down	6.633 down	0.00	230.35	Protein farnesyltransferase. alpha subunit
22129	10.92	0.02	12.68	0.01	12.59	0.68	3.396 up	3.195 up	0.04	7.90	GH61 polysaccharide monooxygenase
22168	10.72	0.12	9.71	0.14	9.61	0.25	2.016 down	2.163 down	0.01	19.84	unknown protein
22201	11.38	0.02	11.09	0.29	9.94	0.28	1.223 down	2.717 down	0.01	26.99	unknown protein
22251	10.55	0.04	10.81	0.01	12.58	0.27	1.199 up	4.105 up	0.00	84.11	Mitochondrial carnitine-acylcarnitine carrier protein
22277	9.52	0.03	11.02	0.31	10.63	0.27	2.830 up	2.159 up	0.01	19.53	sideroflexin-1

22283	13.00	0.24	12.76	0.10	11.91	0.39	1.179 down	2.130 down	0.03	9.31	unknown protein
22332	11.84	0.08	12.72	0.13	13.04	0.17	1.838 up	2.292 up	0.00	43.15	small nuclear ribonucleoprotein E
22351	7.22	0.12	8.32	0.21	9.24	0.30	2.142 up	4.051 up	0.00	40.89	legume-like lectin
22386	12.03	0.16	12.13	0.08	14.74	0.11	1.066 up	6.519 up	0.00	520.33	Epl1/Sm1
22388	11.31	0.04	11.39	0.04	10.28	0.19	1.051 up	2.048 down	0.00	48.99	unknown protein
22426	11.61	0.01	11.09	0.16	10.30	0.11	1.434 down	2.484 down	0.00	97.28	unknown protein
22459	11.26	0.03	12.76	0.17	12.47	0.11	2.822 up	2.322 up	0.00	106.23	carboxypeptidase A
22464	12.04	0.16	11.48	0.06	9.07	0.15	1.475 down	7.818 down	0.00	385.49	Bifunctional P-450:NADPH-P450 reductase
22512	8.85	0.04	10.54	0.16	11.74	0.18	3.243 up	7.442 up	0.00	221.40	short chain dehydrogenase/reductase
22532	14.25	0.09	14.09	0.03	13.22	0.27	1.118 down	2.049 down	0.01	20.57	BZIP transcriptional regulator
22559	13.28	0.01	12.81	0.09	12.16	0.24	1.385 down	2.173 down	0.01	24.45	unknown protein
22625	9.72	0.10	10.32	0.04	10.92	0.15	1.520 up	2.304 up	0.00	64.87	Molecular chaperone (DnaJ superfamily)
22654	12.94	0.03	12.11	0.17	10.77	0.19	1.783 down	4.500 down	0.00	119.71	monodehydroascorbate reductase
22667	9.38	0.13	9.93	0.21	10.51	0.07	1.465 up	2.189 up	0.00	57.21	pre-rRNA-processing protein IPI1. putative
22705	11.22	0.00	11.45	0.06	10.05	0.14	1.175 up	2.255 down	0.00	141.99	urease
22707	11.84	0.03	12.08	0.00	12.99	0.09	1.183 up	2.228 up	0.00	226.72	unknown protein
22718	12.11	0.20	12.57	0.01	13.13	0.05	1.375 up	2.028 up	0.00	79.01	Glutaminylpeptide cyclotransferase
22741	10.19	0.17	9.78	0.14	8.85	0.40	1.330 down	2.539 down	0.02	13.17	unknown protein
22785	12.51	0.07	12.29	0.16	10.86	0.13	1.166 down	3.150 down	0.00	153.37	C2H2 transcriptional regulator (amdA ?)
22830	11.16	0.04	10.51	0.06	9.64	0.04	1.569 down	2.876 down	0.00	893.43	Glutathione S-transferase
22841	10.29	0.13	10.40	0.15	11.65	0.34	1.082 up	2.560 up	0.01	22.40	unknown protein
22875	11.95	0.01	11.88	0.02	10.21	0.09	1.047 down	3.346 down	0.00	648.36	unknown protein
23059	12.83	0.65	13.38	0.17	14.35	0.18	1.461 up	2.879 up	0.01	15.66	Ribosomal protein L24/L26
23083	10.60	0.07	11.90	0.08	12.20	0.54	2.470 up	3.030 up	0.03	9.94	Flavoprotein monooxygenase
23115	10.04	0.06	9.14	0.22	11.80	0.38	1.866 down	3.393 up	0.00	53.89	expansin
23124	12.66	0.25	12.89	0.13	11.55	0.17	1.169 up	2.158 down	0.00	47.20	unknown protein
23146	11.72	0.00	11.73	0.01	13.04	0.20	1.007 up	2.499 up	0.00	72.73	unknown protein
23152	10.85	0.00	10.66	0.02	9.75	0.21	1.145 down	2.143 down	0.00	40.13	unknown LMBR1 domain protein
23171	10.52	0.24	12.38	0.07	13.83	0.21	3.634 up	9.894 up	0.00	191.65	NRPS
23193	12.20	0.02	11.71	0.13	13.41	0.17	1.403 down	2.313 up	0.00	110.62	importin ß KapJ

23271	12.72	0.00	13.21	0.08	14.16	0.15	1.398 up	2.713 up	0.00	105.35	ER-derived vesicles protein Erv41
23462	11.90	0.05	11.22	0.12	10.57	0.19	1.605 down	2.515 down	0.00	46.94	unknown protein
23475	13.71	0.01	13.03	0.09	12.61	0.04	1.594 down	2.142 down	0.00	306.64	Peptidase family M28 protein
24586	4.43	0.40	4.74	0.08	5.89	0.40	1.236 up	2.736 up	0.02	13.45	NRPS
25159	13.46	0.07	12.51	0.12	12.41	0.10	1.924 down	2.060 down	0.00	75.53	PTPc. Protein tyrosine phosphatases (PTP)
25739	12.09	0.10	11.93	0.02	10.89	0.10	1.120 down	2.296 down	0.00	149.52	CorA family metal ion transporter [Metarhizium anisopliae ARSEF 23]
26187	3.23	0.03	3.08	0.01	5.63	0.16	1.113 down	5.270 up	0.00	392.37	unknown protein
26255	8.74	0.01	8.55	0.12	7.47	0.42	1.141 down	2.413 down	0.02	12.86	Zn2Cys6 transcriptional regulator
26642	11.03	0.16	9.61	0.09	9.31	0.48	2.673 down	3.309 down	0.02	13.85	MFS permease
26787	11.81	0.04	11.87	0.31	10.76	0.32	1.036 up	2.073 down	0.02	14.26	ADP-ribosylation factor-binding protein Gga2
26871	10.34	0.02	9.90	0.04	9.33	0.29	1.361 down	2.021 down	0.02	14.61	Zn2Cys6 transcriptional regulator
27042	10.63	0.02	9.74	0.10	9.50	0.17	1.852 down	2.192 down	0.00	45.57	unknown protein
27181	13.84	0.08	12.39	0.04	9.65	0.59	2.740 down	18.345 down	0.00	62.51	MFS permease
27219	3.82	0.46	4.79	0.01	5.25	0.11	1.966 up	2.694 up	0.01	27.28	GH27 α-galactosidase
27422	5.33	0.09	6.43	0.15	6.40	0.18	2.149 up	2.109 up	0.00	35.03	RNA helicase DEAD/DEAH box
27697	8.96	0.01	9.13	0.03	7.37	0.38	1.122 up	3.020 down	0.00	32.84	Amidase
27770	10.59	0.03	11.12	0.02	7.86	0.41	1.439 up	6.637 down	0.00	90.90	MFS permease
27939	10.61	0.02	10.75	0.02	8.99	0.22	1.098 up	3.071 down	0.00	96.69	unknown protein
27983	3.59	0.09	3.24	0.25	5.32	0.50	1.272 down	3.331 up	0.01	22.77	PTH11 GPCR
27992	10.75	0.02	12.75	0.07	12.44	0.06	4.000 up	3.240 up	0.00	713.53	PTH11 GPCR
28036	8.49	0.09	6.27	0.35	6.63	0.10	4.652 down	3.610 down	0.00	95.29	MFS permease
29275	6.62	0.29	7.00	0.01	8.31	0.08	1.297 up	3.219 up	0.00	112.54	unknown protein
29439	10.95	0.03	10.09	0.01	9.73	0.25	1.808 down	2.325 down	0.01	25.79	unknown protein
29642	3.90	0.20	4.46	0.17	5.90	0.36	1.468 up	3.998 up	0.00	34.58	Phosphatidylserine decarboxylase
29710	10.58	0.30	10.13	0.21	8.79	0.12	1.362 down	3.454 down	0.00	72.03	unknown protein
29993	10.37	0.11	9.77	0.11	8.58	0.15	1.516 down	3.465 down	0.00	135.07	unknown protein
30084	7.89	0.03	9.33	0.18	9.10	0.20	2.706 up	2.313 up	0.00	40.89	unknown protein
30166	10.42	0.18	9.65	0.07	7.66	0.21	1.698 down	6.766 down	0.00	171.73	unknown protein
30274	8.21	0.35	8.41	0.22	9.85	0.33	1.148 up	3.126 up	0.01	24.33	unknown protein
30465	3.67	0.05	3.03	0.21	5.06	0.23	1.557 down	2.611 up	0.00	78.07	unknown protein. WD-repeats

30578	9.20	0.05	8.57	0.06	7.03	0.26	1.547 down	4.472 down	0.00	85.87	HhH-GPD family base excision DNA repair protein
30759	10.11	0.07	9.19	0.04	8.70	0.53	1.892 down	2.654 down	0.04	7.88	zinc containing alcohol dehydrogenase superfamily
30776	3.14	0.04	3.19	0.02	4.33	0.07	1.036 up	2.281 up	0.00	424.55	alpha-ketoglutarate-dependent sulfonate diounknown proteingenase
31134	12.64	0.16	12.48	0.05	9.83	0.16	1.116 down	7.005 down	0.00	363.54	isoprenylcysteine carboxyl methyltransferase
31210	11.81	0.20	11.86	0.01	9.69	0.24	1.033 up	4.325 down	0.00	103.75	unknown protein
31248	12.02	0.17	11.60	0.03	10.35	0.10	1.341 down	3.187 down	0.00	183.47	Ribonuclease. T2 family
31367	11.81	0.05	11.60	0.01	10.61	0.24	1.156 down	2.285 down	0.00	34.13	unknown protein
31447	4.70	0.12	5.60	0.09	6.22	0.23	1.867 up	2.863 up	0.00	42.40	unknown protein
31481	9.29	0.08	10.55	0.01	10.35	0.09	2.395 up	2.074 up	0.00	163.52	chaperone protein dnaJ 6
32087	5.26	0.52	5.69	0.05	7.28	0.51	1.346 up	4.040 up	0.01	15.86	short chain dehydrogenase/reductase
32212	2.69	0.23	2.47	0.35	4.08	0.05	1.166 down	2.618 up	0.00	63.62	SSCRP
32243	11.55	0.17	11.04	0.25	9.94	0.55	1.428 down	3.056 down	0.03	9.85	Alpha/beta hydrolase
32261	12.43	0.11	11.56	0.12	11.01	0.32	1.832 down	2.678 down	0.01	20.83	unknown protein
32364	12.01	0.05	11.34	0.07	9.30	0.36	1.586 down	6.507 down	0.00	72.82	GDSL lipase
32402	2.80	0.15	2.79	0.22	4.48	0.08	1.009 down	3.214 up	0.00	154.54	glutathione S-transferase GliG. putative
32478	3.09	0.05	3.22	0.01	4.80	0.25	1.093 up	3.281 up	0.00	72.21	unknown protein
32639	5.29	0.29	5.84	0.25	7.57	0.95	1.471 up	4.886 up	0.05	7.36	unknown protein
32747	3.38	0.04	3.89	0.49	4.90	0.08	1.420 up	2.856 up	0.00	32.83	unknown protein
32798	12.43	0.04	12.70	0.14	14.92	0.08	1.207 up	5.624 up	0.00	659.82	unknown protein
32849	11.61	0.02	11.06	0.15	10.24	0.17	1.465 down	2.581 down	0.00	63.16	unknown protein
33029	7.88	0.41	7.88	0.03	10.07	0.83	1.001 up	4.594 up	0.02	10.76	unknown protein
33058	9.03	0.05	10.14	0.27	10.35	0.35	2.148 up	2.491 up	0.02	13.13	unknown protein
33207	10.47	0.09	10.67	0.07	9.32	0.18	1.147 up	2.231 down	0.00	72.10	cation transport protein ChaC. putative
33342	9.37	0.13	9.91	0.17	12.29	0.10	1.461 up	7.618 up	0.00	488.74	Oligosaccharyltransferase. epsilon subunit
33359	10.04	0.17	10.85	0.20	12.06	0.51	1.743 up	4.041 up	0.01	17.30	translation initiation factor SUI1 and density-regulated protein.
33371	4.62	0.07	4.73	0.18	5.98	0.47	1.078 up	2.569 up	0.02	12.14	phosphoglycerate mutase family protein
33387	12.86	0.03	12.36	0.05	11.04	0.32	1.411 down	3.518 down	0.00	41.94	GCN5-related N-acetyltransferase. putative
33723	4.77	0.32	5.75	0.18	6.27	0.45	1.977 up	2.839 up	0.03	10.28	ankyrin repeat-containing protein. putative
33949	4.97	0.36	5.20	0.10	6.31	0.19	1.178 up	2.535 up	0.00	31.05	NADH:cytochrome b5 reductase (CBR). putative
33960	12.26	0.17	12.87	0.26	13.60	0.24	1.529 up	2.528 up	0.01	23.26	prefoldin subunit 6. putative

34183	8.03	0.18	8.59	0.12	9.05	0.38	1.467 up	2.019 up	0.05	7.22	unknown protein
34197	11.81	0.14	11.98	0.07	10.28	0.09	1.125 up	2.879 down	0.00	259.52	BolA-like protein
34252	13.07	0.11	12.27	0.04	10.55	0.16	1.742 down	5.722 down	0.00	260.43	unknown protein
34280	10.49	0.04	11.50	0.13	11.52	0.34	2.015 up	2.031 up	0.03	10.46	unknown protein
34312	4.12	0.03	4.42	0.24	6.78	0.34	1.230 up	6.305 up	0.00	78.81	conidiation protein 6
34327	11.56	0.06	12.33	0.14	12.66	0.25	1.702 up	2.138 up	0.01	18.78	NADH-ubiquinone oxidoreductase B12 subunit. putative
34413	5.09	0.44	5.85	0.66	8.10	0.59	1.686 up	8.071 up	0.01	21.48	unique protein. secreted
34493	14.80	0.13	14.87	0.15	11.84	0.23	1.048 up	7.778 down	0.00	219.01	a-type peptide pheromone precursor hpp1
34726	4.50	0.23	5.48	0.01	5.92	0.09	1.973 up	2.671 up	0.00	82.70	Guanine nucleotide exchange factor for Ras-likeGTPases (RasGEF)
35109	3.67	0.12	3.05	0.17	7.77	0.74	1.535 down	17.094 up	0.00	57.40	unknown protein. contains WD repeats
35137	3.77	0.32	3.26	0.05	4.93	0.20	1.427 down	2.234 up	0.00	46.93	unknown protein
35534	5.06	0.27	5.48	0.41	6.25	0.20	1.338 up	2.277 up	0.02	14.30	unknown protein
35726	4.52	0.09	4.68	0.14	5.99	0.23	1.121 up	2.775 up	0.00	50.94	Subtilisin like protease
35777	13.64	0.03	13.19	0.06	12.38	0.31	1.359 down	2.383 down	0.01	20.49	K(+)/H(+) antiporter 1
36006	12.77	0.10	12.10	0.24	11.38	0.15	1.591 down	2.621 down	0.00	48.86	Serine/Threonine protein kinase
36159	10.53	0.11	10.26	0.10	9.37	0.15	1.202 down	2.237 down	0.00	58.27	unknown protein
36335	10.29	0.02	10.74	0.11	7.33	0.60	1.362 up	7.762 down	0.00	47.37	Zn2Cys6 transcriptional regulator
36468	11.21	0.07	10.31	0.03	9.22	0.26	1.872 down	3.992 down	0.00	64.80	Zn2Cys6 transcriptional regulator
36530	10.15	0.11	10.03	0.08	8.99	0.26	1.084 down	2.227 down	0.01	26.71	unknown protein
36608	10.78	0.01	11.51	0.14	9.67	0.20	1.657 up	2.149 down	0.00	83.69	unknown protein
36703	10.56	0.02	10.15	0.17	9.20	0.32	1.326 down	2.569 down	0.01	21.38	Zn2Cys6 transcriptional regulator
36913	7.61	0.21	8.22	0.08	6.53	0.35	1.525 up	2.103 down	0.01	24.52	Zn2Cys6 transcriptional regulator
37316	3.48	0.27	3.46	0.09	5.23	0.08	1.014 down	3.350 up	0.00	148.72	unknown protein
37525	10.94	0.01	13.30	0.00	12.93	0.08	5.125 up	3.989 up	0.00	950.42	GPCR . contains RGS domain
37783	11.84	0.14	9.91	0.07	8.85	0.47	3.819 down	7.917 down	0.00	42.63	unknown protein
37950	2.71	0.06	2.98	0.31	4.93	0.07	1.199 up	4.659 up	0.00	188.34	unknown protein
38048	9.31	0.05	9.83	0.13	7.98	0.34	1.436 up	2.503 down	0.00	36.14	unknown protein
38080	12.06	0.15	12.08	0.00	10.74	0.21	1.013 up	2.511 down	0.00	57.90	C2H2 transcriptional regulator
38603	11.37	0.03	11.59	0.08	10.11	0.20	1.162 up	2.404 down	0.00	73.87	MFS permease
38640	12.93	0.03	13.01	0.12	11.64	0.27	1.062 up	2.448 down	0.00	37.03	unknown protein

38833	11.96	0.05	11.25	0.05	10.28	0.29	1.641 down	3.197 down	0.00	38.74	unknown protein
38863	11.04	0.02	10.71	0.12	9.97	0.08	1.257 down	2.096 down	0.00	127.44	unknown protein
39221	8.88	0.07	8.92	0.01	7.78	0.54	1.032 up	2.130 down	0.05	7.11	Zn2Cys6 transcriptional regulator
39590	8.89	0.10	9.33	0.03	10.62	0.25	1.352 up	3.305 up	0.00	60.38	Membrane protein Erj5 with DnaJ domain
39606	10.26	0.28	9.65	0.21	9.02	0.34	1.531 down	2.360 down	0.02	11.04	unknown protein
39755	11.04	0.13	10.17	0.04	9.56	0.35	1.818 down	2.781 down	0.01	19.09	GH16 glucan endo-1.3(4)-β-D-glucosidase
40290	8.98	0.12	9.34	0.19	10.29	0.35	1.281 up	2.479 up	0.01	15.93	unknown protein
40346	10.31	0.16	11.29	0.03	11.51	0.27	1.959 up	2.292 up	0.01	19.65	formamidopyrimidine-DNA glycosylase
40775	11.18	0.01	9.90	0.07	9.72	0.17	2.429 down	2.752 down	0.00	79.10	RTA1 protein. 7 TNM. responds to xenobiotic stimulus
40808	5.85	0.37	9.25	0.13	4.52	0.14	10.571 up	2.515 down	0.00	360.37	short-chain dehydrogenase/reductase
40943	11.97	0.09	11.70	0.06	10.15	0.30	1.207 down	3.540 down	0.00	52.12	unknown protein
41001	7.83	0.10	8.01	0.16	9.03	0.29	1.137 up	2.303 up	0.01	21.33	unknown protein with Nif domain
41171	10.29	0.24	10.00	0.01	8.18	0.40	1.220 down	4.325 down	0.00	36.03	unknown protein
41260	3.05	0.10	3.14	0.08	4.52	0.12	1.057 up	2.770 up	0.00	173.50	PTH11 GPCR
41325	7.93	0.16	8.63	0.05	6.86	0.35	1.625 up	2.104 down	0.00	28.80	RTA1 like protein; 7 TM
41425	8.87	0.09	8.75	0.14	7.08	0.37	1.086 down	3.458 down	0.00	34.34	unknown protein
41428	13.26	0.01	13.22	0.09	11.97	0.26	1.027 down	2.445 down	0.00	37.50	Tyrosine specific protein phosphatase and dual specificity protein phosphatase
41504	9.95	0.06	10.03	0.07	11.04	0.20	1.054 up	2.124 up	0.00	44.04	phosphopantetheinyl transferase. putative
41573	13.52	0.14	13.15	0.02	12.46	0.31	1.293 down	2.085 down	0.02	13.70	BZIP transcriptional regulator
41590	11.48	0.18	10.46	0.05	9.66	0.31	2.028 down	3.551 down	0.00	35.28	iron transporter
41617	11.82	0.06	3.28	0.04	14.25	0.38	372.860 down	5.394 up	0.00	928.13	lae1
41699	6.56	0.05	7.81	0.06	8.35	0.40	2.380 up	3.449 up	0.01	21.86	HET protein
41761	13.74	0.14	13.63	0.15	12.60	0.20	1.077 down	2.205 down	0.00	37.38	iron transporter
41768	6.05	0.27	6.28	0.16	7.87	0.65	1.172 up	3.543 up	0.03	10.68	GH16 cell wall glucanosyltransferase
41942	10.25	0.17	11.08	0.15	9.14	0.18	1.770 up	2.165 down	0.00	92.84	unknown protein
42043	2.67	0.13	2.75	0.06	4.48	0.16	1.059 up	3.502 up	0.00	163.43	unknown protein
42152	8.71	0.03	8.13	0.05	6.34	0.24	1.491 down	5.173 down	0.00	132.06	GH75 chitosanase
42181	9.67	0.13	9.90	0.02	10.99	0.35	1.175 up	2.499 up	0.01	18.87	unknown protein
42264	9.50	0.13	10.25	0.19	6.36	0.45	1.681 up	8.807 down	0.00	97.08	3'-5' exonuclease
42326	11.68	0.10	12.17	0.02	10.64	0.42	1.404 up	2.046 down	0.01	16.18	unknown protein

42355	9.31	0.05	9.83	0.13	8.20	0.21	1.434 up	2.154 down	0.00	69.25	unknown protein
42449	5.86	0.14	6.26	0.13	7.29	0.36	1.313 up	2.689 up	0.01	19.17	N-acetyltransferase activity
42571	6.46	0.02	6.20	0.08	7.51	0.27	1.196 down	2.069 up	0.00	32.21	unknown protein
42848	14.26	0.06	12.98	0.04	12.91	0.31	2.421 down	2.534 down	0.01	22.53	unknown protein
42866	12.18	0.05	10.63	0.11	10.57	0.25	2.932 down	3.059 down	0.00	45.77	thioesterase family protein
42919	13.57	0.01	13.88	0.04	14.71	0.12	1.237 up	2.199 up	0.00	118.71	ubiquitin fusion protein
42942	2.23	0.02	2.11	0.05	3.71	0.10	1.087 down	2.798 up	0.00	338.13	unknown protein
43076	12.32	0.08	12.41	0.03	10.92	0.32	1.070 up	2.626 down	0.00	33.19	unknown protein
43083	10.26	0.14	9.95	0.15	11.94	0.43	1.236 down	3.216 up	0.00	29.07	unknown protein
43115	9.52	0.01	11.05	0.12	10.65	0.14	2.885 up	2.190 up	0.00	88.85	SSCRP
43161	8.68	0.13	9.13	0.08	10.36	0.32	1.364 up	3.214 up	0.00	33.29	Carboxylesterase and related proteins
43199	4.03	0.12	4.55	0.35	5.95	0.18	1.442 up	3.792 up	0.00	61.63	unknown protein
43225	11.51	0.14	12.52	0.20	13.24	0.18	2.007 up	3.317 up	0.00	65.31	U6 small nuclear ribonucleoprotein (Lsm3). putative
43236	12.29	0.14	12.83	0.09	13.40	0.21	1.449 up	2.153 up	0.01	25.68	unknown protein
43269	9.43	0.27	11.10	0.33	10.88	0.37	3.176 up	2.723 up	0.01	14.82	bHLH transcriptional regulator
43302	3.68	0.37	3.67	0.17	6.12	0.43	1.007 down	5.446 up	0.00	42.46	unknown protein
43392	5.42	0.08	6.15	0.25	7.97	0.55	1.651 up	5.859 up	0.01	26.27	unique protein
43401	8.59	0.30	9.06	0.33	12.84	0.23	1.387 up	19.063 up	0.00	229.96	unknown protein
43427	2.69	0.17	2.94	0.07	4.25	0.18	1.188 up	2.936 up	0.00	77.73	unknown protein
43701	7.86	0.08	8.85	0.09	10.05	0.36	1.993 up	4.588 up	0.00	41.84	MFS multidrug transporter
43893	12.80	0.21	12.25	0.15	11.56	0.10	1.466 down	2.366 down	0.00	56.48	unknown protein
43974	10.19	0.05	10.87	0.21	11.31	0.26	1.608 up	2.180 up	0.01	16.78	UDP-galactose transporter
44117	13.34	0.15	12.83	0.10	12.00	0.07	1.424 down	2.529 down	0.00	145.01	Heterokaryon incompatibility protein HEC-C
44175	13.28	0.05	10.77	0.02	9.83	0.13	5.701 down	10.981 down	0.00	769.90	MFS H+ sugar transporter
44251	9.96	0.05	9.85	0.10	11.30	0.28	1.078 down	2.544 up	0.00	39.80	GTPase-activating protein Msb3 (Sec4/Rabs)
44278	15.15	0.02	13.74	0.04	12.88	0.20	2.647 down	4.813 down	0.00	140.75	Rab geranyl transferase escort protein
44362	9.48	0.01	12.44	0.06	12.54	0.12	7.751 up	8.294 up	0.00	779.09	unknown protein
44366	9.94	0.00	8.74	0.03	8.82	0.29	2.297 down	2.179 down	0.01	19.55	Glycosylphosphatidylinositol-specific phospholipase C
44459	10.42	0.09	11.47	0.02	12.13	0.14	2.076 up	3.266 up	0.00	143.38	Rfc4p (replication factor C) subunit 4
44476	11.26	0.00	8.94	0.05	9.68	0.40	4.980 down	2.986 down	0.00	30.16	MRP-type ABC transporter

44640	12.15	0.16	13.24	0.01	13.55	0.06	2.128 up	2.630 up	0.00	191.12	unknown protein
44878	12.56	0.08	12.19	0.05	11.29	0.18	1.298 down	2.413 down	0.00	59.01	unknown protein
45250	11.95	0.01	12.52	0.01	14.00	0.13	1.483 up	4.138 up	0.00	335.30	oleate-delta12-desaturase
45512	10.58	0.03	10.97	0.21	11.72	0.30	1.314 up	2.214 up	0.01	15.24	unknown protein
45573	11.23	0.08	11.64	0.03	12.32	0.09	1.329 up	2.118 up	0.00	128.61	PTH11 GPCR
45598	13.30	0.36	12.26	0.04	12.30	0.24	2.064 down	2.003 down	0.02	12.83	unknown protein
45675	12.45	0.02	11.88	0.09	10.71	0.14	1.482 down	3.326 down	0.00	164.98	unknown protein
45717	13.08	0.13	10.91	0.08	10.92	0.27	4.496 down	4.485 down	0.00	75.32	GH47 α-1.2-mannosidase
46057	11.12	0.13	11.27	0.09	12.12	0.19	1.111 up	2.011 up	0.00	33.62	unknown protein
46128	8.24	0.15	6.81	0.08	7.16	0.30	2.682 down	2.114 down	0.01	19.61	AAA ATPase
46320	11.98	0.13	12.50	0.21	13.09	0.17	1.441 up	2.162 up	0.00	29.50	small nuclear ribonucleoprotein LSM2
46443	10.34	0.00	11.04	0.11	9.05	0.38	1.623 up	2.437 down	0.00	31.96	GT α-1.6-mannosyltransferase
46446	12.63	0.09	12.41	0.01	11.49	0.06	1.162 down	2.194 down	0.00	266.99	Thioredoxin binding protein TBP-2
46545	7.01	0.05	8.22	0.21	11.21	0.38	2.305 up	18.397 up	0.00	143.43	Prephenate dehydrogenase
46763	12.42	0.12	12.78	0.05	10.56	0.27	1.281 up	3.639 down	0.00	89.64	endonuclease/exonuclease/phosphatase family protein
46816	8.71	0.07	7.63	0.28	10.41	0.23	2.115 down	3.253 up	0.00	116.09	GH3 β-glucosidase CEL3d
47077	10.59	0.12	9.60	0.11	9.58	0.15	1.986 down	2.020 down	0.00	39.58	diounknown proteingenase
47136	10.93	0.07	11.02	0.04	12.18	0.23	1.064 up	2.384 up	0.00	42.62	Glutathione peroxidase
47268	11.21	0.06	12.44	0.02	12.76	0.29	2.346 up	2.922 up	0.00	32.26	GH3 β-glucosidase BGL3i
47286	13.64	0.10	12.87	0.05	12.40	0.32	1.704 down	2.369 down	0.01	15.76	unknown protein
47315	10.91	0.01	9.74	0.14	9.62	0.27	2.264 down	2.449 down	0.01	25.47	P-type ATPase
47330	10.67	0.11	11.33	0.08	12.72	0.20	1.578 up	4.142 up	0.00	112.69	SSCRP
47479	11.08	0.18	11.38	0.08	9.37	0.37	1.229 up	3.275 down	0.00	39.96	Zn2Cys6 transcriptional regulator
47510	11.79	0.13	12.33	0.25	12.95	0.34	1.451 up	2.230 up	0.02	11.02	Complex 1 LYR protein
47603	10.71	0.13	10.28	0.08	11.77	0.34	1.351 down	2.082 up	0.01	23.36	succinyl-CoA:3-ketoacid-coenzyme A transferase subunit A
47733	11.87	0.05	13.84	0.09	14.21	0.18	3.912 up	5.034 up	0.00	178.55	SSCRP
47795	11.62	0.07	12.11	0.26	12.64	0.24	1.410 up	2.027 up	0.01	14.79	ribosomal protein S10.
47814	12.94	0.00	12.89	0.09	11.64	0.34	1.035 down	2.472 down	0.01	23.69	prenylcysteine lyase. putative
47926	9.50	0.17	10.29	0.12	11.64	0.29	1.723 up	4.383 up	0.00	57.08	ABC1 family protein
48080	11.52	0.05	11.72	0.13	7.84	0.25	1.148 up	12.864 down	0.00	341.98	unknown protein

48211	13.39	0.08	12.47	0.06	11.19	0.14	1.886 down	4.585 down	0.00	236.39	unknown protein. intracellular
48280	9.49	0.34	10.03	0.48	13.57	0.76	1.455 up	16.937 up	0.00	34.96	unknown protein
48295	10.86	0.07	11.76	0.06	12.41	0.39	1.862 up	2.929 up	0.01	17.67	unknown protein
48366	12.79	0.28	13.14	0.03	13.87	0.08	1.269 up	2.111 up	0.00	44.92	26S proteasome regulatory complex subunit Rpn12
48438	12.23	0.05	11.88	0.13	10.48	0.17	1.270 down	3.364 down	0.00	125.44	Zn2Cys6 transcriptional regulator
48792	11.02	0.05	12.15	0.27	12.80	0.15	2.193 up	3.449 up	0.00	73.33	amidase
48951	12.71	0.21	12.37	0.04	11.56	0.10	1.266 down	2.215 down	0.00	66.59	unknown protein
49048	10.91	0.13	9.81	0.07	8.30	0.24	2.152 down	6.111 down	0.00	126.53	RNA-dependent RNA-polymerase
49081	8.73	0.08	7.12	0.10	13.14	0.31	3.051 down	21.255 up	0.00	477.21	GH74 Xyloglucanase CEL74a
49112	12.14	0.10	11.14	0.04	10.64	0.17	2.004 down	2.831 down	0.00	76.38	Glutamate-1-semialdehyde aminotransferase
49149	10.33	0.01	11.20	0.27	11.93	0.26	1.817 up	3.015 up	0.00	32.01	Translocation protein Sec66
49193	12.94	0.04	13.29	0.14	13.99	0.33	1.274 up	2.066 up	0.02	11.75	GH17 glucan 1.3-β-glucosidase
49295	12.23	0.14	12.05	0.02	11.22	0.11	1.128 down	2.004 down	0.00	80.42	unknown protein
49366	12.46	0.18	9.16	0.14	10.27	0.16	9.869 down	4.569 down	0.00	223.32	unknown protein
49517	10.83	0.07	11.38	0.22	12.22	0.17	1.469 up	2.630 up	0.00	49.67	unknown protein
49589	13.23	0.17	12.76	0.00	6.17	0.46	1.387 down	133.610 down	0.00	350.57	unknown protein
49753	11.05	0.14	10.40	0.04	9.40	0.12	1.570 down	3.133 down	0.00	146.60	L-arabinitol 4-dehydrogenase
49832	11.96	0.07	11.75	0.10	10.63	0.12	1.155 down	2.510 down	0.00	137.20	QDE2. Argonaute-like protein. essential for quelling
49864	11.99	0.14	12.39	0.15	13.11	0.12	1.322 up	2.180 up	0.00	55.05	unknown protein
49928	8.73	0.03	8.54	0.07	11.65	0.27	1.144 down	7.578 up	0.00	197.05	unknown protein. only in Sordariomycetes
49976	8.40	0.20	4.56	0.03	13.34	0.39	14.409 down	30.525 up	0.00	558.57	GH5 endo-β-1.4-glucanase EGL5/CEL45a
50077	10.12	0.18	11.21	0.05	11.34	0.25	2.121 up	2.333 up	0.01	23.77	Amino acid transporters
50104	9.38	0.19	10.19	0.14	10.41	0.38	1.752 up	2.040 up	0.05	7.43	phosphatidyl synthase (A. fumigatus)
50215	11.33	0.10	8.66	0.02	12.58	0.30	6.380 down	2.374 up	0.00	186.73	GH16 endo-1.3-β-D-glucosidase/1.3-glucan binding protein
50249	13.26	0.14	12.96	0.00	12.18	0.09	1.231 down	2.103 down	0.00	104.05	peptidase family M3
50323	7.59	0.00	8.75	0.13	9.44	0.45	2.246 up	3.620 up	0.01	18.56	OOC1
50429	9.35	0.22	10.04	0.12	11.16	0.27	1.620 up	3.515 up	0.00	41.38	unknown protein
50516	12.60	0.02	13.30	0.05	13.63	0.04	1.621 up	2.032 up	0.00	505.61	ATPase. V-type
50583	10.25	0.14	9.61	0.08	8.30	0.13	1.556 down	3.869 down	0.00	196.00	Guanine deaminase (GDEase). an aminohydrolase responsible for the conversion ofGu

50593	10.16	0.18	11.63	0.10	11.67	0.04	2.758 up	2.845 up	0.00	182.36	5' nucleotidase
50616	10.40	0.42	10.56	0.19	7.36	1.34	1.118 up	8.243 down	0.04	8.71	Coenzyme A transferase
50625	13.23	0.01	12.96	0.24	12.22	0.16	1.201 down	2.018 down	0.00	30.11	unknown protein
50786	11.63	0.12	13.23	0.26	13.14	0.16	3.020 up	2.845 up	0.00	56.08	Flavin-linked sulfhydryl oxidase
50996	10.82	0.02	8.64	0.14	7.91	0.51	4.528 down	7.482 down	0.00	35.39	unknown protein. C2 domain
51130	11.66	0.13	11.57	0.22	12.78	0.36	1.063 down	2.182 up	0.01	15.22	cytochrome c
51375	11.81	0.01	12.78	0.12	13.40	0.14	1.968 up	3.018 up	0.00	118.93	unknown protein
51407	11.59	0.12	10.78	0.03	10.46	0.21	1.753 down	2.200 down	0.00	29.70	unknown protein
51415	13.00	0.11	12.12	0.07	11.76	0.40	1.841 down	2.361 down	0.03	10.32	unknown protein
51426	12.55	0.12	12.88	0.27	14.01	0.13	1.256 up	2.757 up	0.00	61.75	Ribosomal protein L30 based on homology with similar proteins in other fungi.
51558	12.02	0.05	11.60	0.12	10.63	0.19	1.335 down	2.630 down	0.00	61.53	unknown protein
51603	13.58	0.00	13.45	0.01	12.39	0.11	1.097 down	2.285 down	0.00	180.82	unknown protein
51654	8.76	0.02	8.46	0.13	7.44	0.48	1.231 down	2.496 down	0.03	10.15	unknown protein
51846	12.50	0.01	12.35	0.22	11.27	0.20	1.113 down	2.358 down	0.00	42.05	Zn2Cys6 transcriptional regulator
52073	13.53	0.22	12.28	0.03	12.07	0.18	2.362 down	2.743 down	0.00	48.70	sulfatase
52222	8.32	0.10	7.53	0.05	6.75	0.27	1.733 down	2.984 down	0.00	35.59	ankyrin repeat domain-containing protein
52315	3.50	0.18	3.45	0.42	14.38	0.06	1.038 down	1882.167 up	0.00	2729.49	copper transporter Ctr
52476	7.53	0.13	12.78	0.18	12.10	0.77	38.161 up	23.863 up	0.00	48.07	unknown protein
52499	11.43	0.00	11.38	0.21	9.95	0.13	1.035 down	2.786 down	0.00	112.08	C2H2 transcriptional regulator
52505	11.34	0.02	11.74	0.00	12.37	0.33	1.315 up	2.034 up	0.02	11.51	unknown protein
52875	12.72	0.10	12.12	0.13	10.79	0.35	1.522 down	3.806 down	0.00	35.41	Zn2Cys6 transcriptional regulator
53004	12.10	0.01	12.24	0.11	10.89	0.18	1.103 up	2.307 down	0.00	71.27	protein tyrosine phosphatase activity. M phase of mitotic cell cycle
53029	3.87	0.59	3.71	0.11	4.88	0.22	1.115 down	2.011 up	0.02	11.81	ß-lactamase-like protein
53053	10.19	0.17	12.43	0.03	12.59	0.08	4.738 up	5.307 up	0.00	420.23	unknown protein
53079	10.68	0.16	8.65	0.15	8.21	0.83	4.105 down	5.546 down	0.03	9.83	NADP/FAD dependent oxidoreductase
53153	9.26	0.01	9.14	0.04	7.89	0.23	1.092 down	2.593 down	0.00	55.17	unknown protein
53168	2.80	0.05	2.77	0.05	4.64	0.46	1.024 down	3.572 up	0.01	26.35	Cytochrome P450. putative
53186	5.71	0.05	6.10	0.05	7.82	0.51	1.317 up	4.326 up	0.01	24.10	PL20 glucuronan lyase A
53282	7.05	0.42	6.98	0.06	8.29	0.42	1.047 down	2.361 up	0.02	11.29	NAD(P)H-dependent FMN reductase LOT6. putative
53314	6.12	0.37	6.71	0.09	7.21	0.15	1.507 up	2.126 up	0.01	18.31	unknown protein

53334	8.51	0.08	9.13	0.01	9.60	0.22	1.530 up	2.122 up	0.01	26.49	unknown protein
53360	9.98	0.06	9.02	0.07	8.60	0.34	1.942 down	2.594 down	0.01	17.39	SSCRP
53366	9.94	0.15	10.38	0.03	11.05	0.04	1.353 up	2.151 up	0.00	150.61	translation initiation protein Sua5p.
53372	11.26	0.14	9.80	0.18	10.10	0.16	2.753 down	2.231 down	0.00	46.37	acyltransferase 3
53373	13.32	0.11	13.00	0.22	11.54	0.16	1.248 down	3.435 down	0.00	99.03	CoA-transferase family III
53378	3.49	0.28	4.28	0.23	5.21	0.25	1.733 up	3.298 up	0.00	32.90	siderophore transporter
53401	10.03	0.03	10.29	0.00	8.59	0.30	1.200 up	2.706 down	0.00	47.28	unknown protein
53428	9.57	0.18	9.58	0.10	6.38	0.45	1.005 up	9.099 down	0.00	77.20	SAM-dependent metyhltransferase
53452	2.22	0.03	2.32	0.14	3.99	0.09	1.070 up	3.399 up	0.00	361.91	unknown protein
53475	11.33	0.05	10.27	0.04	9.82	0.09	2.083 down	2.851 down	0.00	271.95	MFS permease
53500	10.94	0.05	10.92	0.01	8.30	0.28	1.012 down	6.223 down	0.00	146.60	unknown protein
53501	11.71	0.06	10.77	0.27	10.64	0.24	1.921 down	2.097 down	0.01	16.52	unknown protein
53525	9.00	0.07	9.30	0.09	10.09	0.14	1.224 up	2.126 up	0.00	67.53	unknown protein (tyrosin phosphatase domain)
53542	13.99	0.00	13.66	0.07	12.53	0.04	1.254 down	2.746 down	0.00	877.04	GH76 α-1.6-mannanase
53561	10.83	0.02	9.51	0.01	8.54	0.38	2.483 down	4.876 down	0.00	40.70	unknown protein
53596	9.97	0.11	10.38	0.08	11.00	0.09	1.330 up	2.044 up	0.00	89.95	unknown protein
53611	10.79	0.11	9.72	0.15	8.52	0.25	2.105 down	4.822 down	0.00	79.84	MFS permease
53615	10.94	0.01	10.47	0.03	9.55	0.18	1.383 down	2.620 down	0.00	73.72	unknown protein with WD repeats
53665	4.80	0.26	5.21	0.10	6.81	0.09	1.329 up	4.049 up	0.00	165.54	unknown protein
53672	12.28	0.01	11.49	0.04	10.83	0.17	1.729 down	2.741 down	0.00	83.44	unknown protein
53673	12.24	0.04	11.36	0.05	13.29	0.27	1.835 down	2.072 up	0.00	57.85	NADP:D-xylose dehydrogenase [Hypocrea jecorina]
53701	10.67	0.05	10.87	0.22	11.94	0.23	1.149 up	2.411 up	0.00	33.28	unknown protein
53747	8.19	0.16	8.74	0.04	6.66	0.43	1.468 up	2.879 down	0.00	29.88	unknown protein
53777	11.75	0.01	10.36	0.21	8.39	0.36	2.621 down	10.284 down	0.00	93.14	unknown protein
53824	11.12	0.08	7.92	0.11	9.00	0.62	9.191 down	4.343 down	0.01	23.12	unknown secreted protein
53859	12.24	0.04	12.01	0.04	10.56	0.14	1.176 down	3.203 down	0.00	196.84	C-4 sterol methyl oxidase
53862	3.54	0.16	4.12	0.05	5.31	0.20	1.501 up	3.430 up	0.00	79.72	unknown protein with WD repeats
53863	6.54	0.12	6.23	0.04	7.93	0.35	1.246 down	2.616 up	0.00	31.97	Flavoprotein monooxygenase
53868	9.12	0.12	8.15	0.07	7.44	0.17	1.960 down	3.207 down	0.00	86.72	NADH:flavin oxidoreductase/NADH oxidase
53888	9.70	0.14	8.72	0.09	8.29	0.08	1.981 down	2.663 down	0.00	137.85	pirin

53903	9.91	0.02	8.92	0.08	6.84	0.53	1.980 down	8.402 down	0.00	42.33	MFS permease
53939	11.24	0.09	11.55	0.22	10.17	0.02	1.238 up	2.099 down	0.00	128.68	DNA polymerase y family member involved in translesion synthesis during DNA repair
53956	12.23	0.02	12.53	0.10	11.14	0.24	1.231 up	2.129 down	0.00	43.55	unknown protein
53961	11.44	0.11	7.67	0.05	8.37	0.34	13.697 down	8.413 down	0.00	116.41	Aspartyl protease
53964	3.69	0.26	4.24	0.10	6.28	0.45	1.471 up	6.049 up	0.00	39.50	oxalate decarboxylase
53989	11.09	0.15	10.71	0.13	9.71	0.30	1.298 down	2.593 down	0.01	24.27	pfkB family kinase. putative
54048	10.23	0.05	8.90	0.19	7.82	0.34	2.510 down	5.314 down	0.00	50.05	unknown protein
54052	7.02	0.67	8.60	0.28	11.25	0.45	2.977 up	18.694 up	0.00	57.95	unknown protein
54089	5.43	0.36	5.88	0.11	7.38	0.32	1.367 up	3.852 up	0.00	34.61	unknown protein
54128	10.79	0.16	10.71	0.12	9.48	0.24	1.056 down	2.477 down	0.00	38.54	IlvA Threonine dehydratase
54144	9.46	0.05	9.33	0.10	11.11	0.08	1.097 down	3.141 up	0.00	470.57	ferric reductase
54160	3.12	0.07	2.99	0.04	4.95	0.13	1.095 down	3.539 up	0.00	298.65	GliI [Aspergillus fumigatus]
54166	12.55	0.13	12.31	0.09	11.01	0.30	1.179 down	2.905 down	0.00	34.39	Cytochrome P450
54179	4.69	0.27	4.79	0.03	6.19	0.08	1.067 up	2.827 up	0.00	119.04	glutathione S-transferase
54202	11.23	0.09	11.09	0.10	10.04	0.18	1.098 down	2.275 down	0.00	57.10	unknown protein. contains DUF155 domain
54226	10.39	0.14	9.91	0.05	11.70	0.19	1.394 down	2.478 up	0.00	96.21	unknown protein
54227	9.66	0.05	6.46	0.02	6.73	0.50	9.221 down	7.643 down	0.00	45.89	short-chain dehydrogenase/reductase
54230	9.27	0.03	8.44	0.09	7.60	0.56	1.777 down	3.192 down	0.03	10.05	NACHT domain WD40 repeat-containing protein. related to HET
54239	11.12	0.04	8.87	0.13	9.37	0.34	4.733 down	3.356 down	0.00	40.13	Multicopper oxidases
54285	11.88	0.08	11.57	0.01	10.37	0.11	1.239 down	2.840 down	0.00	219.80	unknown protein
54335	12.53	0.15	12.12	0.02	11.08	0.22	1.329 down	2.742 down	0.00	47.59	unknown protein
54349	11.44	0.02	12.16	0.12	13.12	0.31	1.637 up	3.184 up	0.00	32.35	small nuclear ribonucleoprotein F
54352	10.88	0.20	9.54	0.12	7.14	0.38	2.527 down	13.359 down	0.00	106.81	unknown protein.
54366	8.59	0.19	8.36	0.04	7.03	0.26	1.169 down	2.940 down	0.00	44.69	ceramidase. nonlysosomal
54426	6.54	0.18	6.74	0.11	7.84	0.26	1.145 up	2.457 up	0.00	29.53	unknown protein
54444	10.03	0.21	10.02	0.12	8.44	0.40	1.005 down	3.004 down	0.01	23.57	unknown protein
54461	3.62	0.44	3.54	0.08	6.57	1.36	1.056 down	7.740 up	0.04	7.78	MFS permease
54462	4.15	0.36	4.80	0.02	6.02	0.11	1.567 up	3.654 up	0.00	78.93	unknown protein
54469	11.85	0.02	11.62	0.10	10.45	0.16	1.174 down	2.646 down	0.00	100.97	Vacuolar transporter chaperone 4 (vtc4). putative
54550	9.43	0.02	9.43	0.01	8.33	0.10	1.001 down	2.149 down	0.00	185.46	short chain dehydrognease/reductase

54611	12.45	0.17	12.57	0.09	14.11	0.18	1.086 up	3.175 up	0.00	99.58	unknown protein
54622	6.21	0.10	4.86	0.06	8.44	0.37	2.553 down	4.686 up	0.00	111.46	unknown protein
54633	13.01	0.02	12.59	0.01	11.88	0.35	1.339 down	2.183 down	0.02	12.87	GT ß-glycosyltransferases
54659	12.91	0.18	12.52	0.08	11.06	0.56	1.315 down	3.606 down	0.02	14.13	unknown protein
54667	9.67	0.07	12.33	0.13	13.25	0.31	6.311 up	11.946 up	0.00	141.91	acyl-CoA synthetase
54669	4.89	1.18	5.53	0.10	8.14	0.28	1.552 up	9.480 up	0.01	26.79	Golgi matrix protein. rud3. involved in the structural organization of the cis-Golgi
54674	11.59	0.00	12.05	0.08	10.56	0.29	1.375 up	2.033 down	0.00	31.52	unknown protein
54676	9.75	0.17	9.97	0.25	10.90	0.46	1.169 up	2.232 up	0.04	7.58	COX17. cytochrome C oxidase assembly protein
54694	11.81	0.02	9.73	0.09	8.39	0.30	4.224 down	10.692 down	0.00	136.18	aryl-alcohol dehydrogenases
54703	11.98	0.01	10.57	0.00	10.65	0.21	2.647 down	2.513 down	0.00	50.71	C2H2 transcriptional regulator
54723	2.95	0.14	2.91	0.03	4.38	0.03	1.025 down	2.698 up	0.00	460.57	SSCRP
54761	5.79	0.13	3.84	0.08	7.53	1.13	3.862 down	3.324 up	0.02	11.89	quercetin 2.3-diounknown proteingenase
54768	11.25	0.09	13.93	0.07	13.34	0.17	6.432 up	4.254 up	0.00	217.38	unknown protein
54784	2.85	0.07	2.92	0.06	4.70	0.10	1.049 up	3.593 up	0.00	423.61	unknown protein
54789	4.33	0.17	4.88	0.34	5.96	0.55	1.464 up	3.105 up	0.03	9.66	short chain dehydrogenase/reductase
54790	8.87	0.16	8.39	0.00	7.16	0.25	1.395 down	3.263 down	0.00	53.39	unknown protein
54846	9.26	0.10	9.22	0.20	7.51	0.34	1.025 down	3.366 down	0.00	38.23	half-sized ABC transporter
54870	12.57	0.12	12.53	0.04	11.47	0.26	1.025 down	2.143 down	0.01	27.18	acetate—CoA ligase
54893	12.97	0.16	13.15	0.05	11.73	0.26	1.138 up	2.349 down	0.00	38.18	unknown protein
54902	7.59	0.04	7.68	0.00	5.94	0.51	1.062 up	3.137 down	0.01	18.63	unknown protein
54954	6.53	0.42	6.31	0.17	5.07	0.31	1.163 down	2.740 down	0.01	18.86	MRP-type ABC transporter
54972	8.47	0.05	6.77	0.03	5.49	0.14	3.231 down	7.847 down	0.00	508.28	MFS permease
55034	13.17	0.02	12.22	0.17	11.57	0.24	1.932 down	3.027 down	0.00	41.50	unknown protein
55036	6.89	0.06	7.93	0.17	8.88	0.29	2.055 up	3.968 up	0.00	47.40	unknown protein
55039	5.97	0.00	7.79	0.37	7.71	0.21	3.534 up	3.347 up	0.00	43.72	unknown protein
55041	10.32	0.22	10.16	0.01	9.24	0.18	1.115 down	2.113 down	0.00	33.85	unknown protein
55060	9.86	0.09	11.18	0.07	10.87	0.19	2.487 up	2.002 up	0.00	42.09	kinesin heavy chain
55105	9.04	0.07	8.08	0.16	7.75	0.13	1.935 down	2.440 down	0.00	64.85	Zn2Cys6 transcriptional regulator
55126	10.02	0.11	8.82	0.09	8.77	0.36	2.304 down	2.382 down	0.02	14.38	unknown protein
55172	9.16	0.01	9.56	0.02	10.83	0.16	1.324 up	3.179 up	0.00	141.66	unknown protein

55193	9.43	0.14	8.36	0.11	7.88	0.29	2.101 down	2.923 down	0.01	28.26	unknown protein
55213	9.00	0.04	9.09	0.08	7.70	0.35	1.066 up	2.472 down	0.01	24.21	tRNA-specific adenosine deaminase
55240	11.61	0.22	8.84	0.01	9.82	0.27	6.843 down	3.475 down	0.00	75.62	large-conductance mechanosensitive channel
55272	13.40	0.01	14.67	0.10	14.62	0.07	2.401 up	2.322 up	0.00	220.27	unknown protein
55279	11.94	0.03	11.24	0.23	10.46	0.14	1.618 down	2.786 down	0.00	67.25	unknown protein
55353	12.32	0.04	12.02	0.07	10.47	0.13	1.233 down	3.611 down	0.00	268.80	pathotenate kinase
55362	10.38	0.11	9.15	0.19	9.37	0.09	2.356 down	2.012 down	0.00	66.68	Heat shock protein 70
55374	4.87	0.02	4.49	0.28	5.94	0.07	1.298 down	2.104 up	0.00	89.51	unknown protein
55407	10.82	0.04	9.82	0.05	9.71	0.28	2.000 down	2.158 down	0.01	18.62	MFS permease
55443	9.93	0.09	11.09	0.13	11.09	0.35	2.239 up	2.233 up	0.02	12.66	unknown protein
55454	11.89	0.08	11.63	0.16	10.28	0.13	1.195 down	3.038 down	0.00	131.81	unknown protein
55566	7.77	0.00	8.12	0.06	8.83	0.27	1.272 up	2.085 up	0.01	18.95	multicopper oidase
55584	12.69	0.20	12.51	0.10	11.66	0.19	1.131 down	2.033 down	0.00	28.62	unknown protein
55589	11.34	0.08	12.27	0.22	12.87	0.19	1.915 up	2.906 up	0.00	47.10	3' exoribonuclease involved in RNA processing during translation.
55595	12.29	0.03	12.68	0.03	13.32	0.15	1.311 up	2.042 up	0.00	55.43	Ca2+ transporter
55597	12.96	0.01	12.49	0.19	11.48	0.40	1.384 down	2.787 down	0.01	16.49	unknown protein
55599	11.04	0.00	10.26	0.03	9.87	0.13	1.710 down	2.246 down	0.00	92.06	RNA 12 protein
55630	10.45	0.32	8.50	0.01	7.48	0.17	3.870 down	7.837 down	0.00	159.88	monocarboxylate transporter
55631	13.01	0.16	12.39	0.00	11.81	0.26	1.531 down	2.294 down	0.01	22.27	phospholipase
55634	7.01	0.54	6.61	0.04	5.69	0.09	1.319 down	2.506 down	0.01	21.39	MFS permease
55636	10.40	0.09	9.60	0.17	9.07	0.24	1.750 down	2.511 down	0.00	28.95	MRP-type ABC transporter
55637	9.83	0.28	9.06	0.02	8.38	0.29	1.710 down	2.742 down	0.01	21.16	Unknown protein with RNA-dependent RNA polymerase (RdRP) and DEXDc/DEAD-like
55666	3.59	0.10	3.67	0.45	5.67	0.03	1.057 up	4.246 up	0.00	96.97	unknown protein with WD repeats
55671	4.37	0.35	5.10	0.38	5.53	0.11	1.665 up	2.237 up	0.01	14.72	unknown protein
55719	5.40	0.17	6.63	0.11	6.61	0.34	2.340 up	2.304 up	0.01	14.66	Protein kinase
55782	5.69	0.49	6.05	0.27	8.13	0.31	1.283 up	5.432 up	0.00	42.42	dipeptidyl peptidase 5
55802	8.68	0.02	9.12	0.07	11.46	0.65	1.351 up	6.833 up	0.01	25.69	GH76 α-1.6-mannanase
55871	6.59	0.19	4.80	0.03	5.02	0.24	3.474 down	2.975 down	0.00	49.29	Short-chain dehydrogenase/reductase
55887	6.50	0.20	8.77	0.10	11.77	0.62	4.792 up	38.374 up	0.00	80.82	unknown protein. secreted
55999	8.14	0.04	7.07	0.10	9.36	0.25	2.100 down	2.335 up	0.00	93.12	GH27 α-galactosidase

56026	10.92	0.01	10.44	0.05	9.44	0.36	1.396 down	2.779 down	0.01	20.38	unknown protein
56064	14.14	0.12	13.91	0.12	12.81	0.11	1.179 down	2.519 down	0.00	119.36	unknown protein
56093	2.91	0.02	3.22	0.09	4.58	0.19	1.247 up	3.202 up	0.00	96.95	unknown protein
56117	12.28	0.14	12.36	0.07	10.93	0.16	1.054 up	2.548 down	0.00	92.61	unknown protein
56141	12.39	0.13	11.59	0.06	10.76	0.59	1.738 down	3.095 down	0.04	8.66	unknown protein
56211	8.69	0.12	11.09	0.18	10.98	0.17	5.270 up	4.898 up	0.00	150.26	unknown protein
56218	11.43	0.05	11.97	0.12	10.00	0.18	1.453 up	2.688 down	0.00	131.31	unknown protein
56236	6.42	0.37	6.15	0.45	7.87	0.57	1.201 down	2.746 up	0.03	9.86	SAM-dependent methyltransferases
56256	12.38	0.15	12.33	0.08	11.28	0.20	1.034 down	2.133 down	0.00	38.86	unknown protein
56259	3.29	0.24	3.79	0.37	5.09	0.24	1.422 up	3.484 up	0.00	35.31	unknown protein
56278	3.52	0.04	3.81	0.39	5.41	0.40	1.217 up	3.688 up	0.01	24.71	unknown protein. in Sordariomycetes
56289	7.17	0.07	7.66	0.06	6.05	0.49	1.404 up	2.177 down	0.02	13.76	MFS permease
56326	6.23	0.11	6.69	0.33	7.76	0.42	1.370 up	2.884 up	0.02	13.76	Zinc-containing alcohol dehydrogenase
56350	4.55	0.13	4.32	0.15	5.85	0.19	1.175 down	2.450 up	0.00	66.94	cysteine synthase. putative
56376	7.86	0.19	8.78	0.03	9.00	0.21	1.888 up	2.202 up	0.01	26.77	unknown protein
56390	13.42	0.00	12.39	0.07	11.77	0.31	2.036 down	3.134 down	0.00	31.42	unknown protein. TPR repeats
56399	12.53	0.03	11.79	0.08	10.98	0.23	1.669 down	2.925 down	0.00	50.08	unknown protein
56408	12.30	0.06	12.42	0.01	11.26	0.22	1.081 up	2.056 down	0.00	40.20	unknown protein
56426	12.17	0.01	12.01	0.23	11.13	0.14	1.116 down	2.044 down	0.00	38.92	GCPR. mPR-type
56427	9.63	0.16	7.92	0.12	8.57	0.27	3.269 down	2.089 down	0.00	30.08	Arylacetamide deacetylase
56434	5.33	0.02	4.92	0.07	7.22	0.09	1.327 down	3.728 up	0.00	790.24	FKBP-type peptidyl-prolyl isomerase. putative
56448	6.82	0.09	5.56	0.04	8.23	0.11	2.402 down	2.663 up	0.00	579.00	GH18 chitinase CHI18-11
56467	7.09	0.18	7.83	0.01	8.37	0.10	1.678 up	2.430 up	0.00	87.54	Mitochondrial deoxynucleotide carrier protein
56469	11.29	0.18	11.51	0.08	9.94	0.03	1.172 up	2.548 down	0.00	269.90	unknown protein
56499	8.67	0.23	9.48	0.11	10.24	0.41	1.754 up	2.959 up	0.01	14.90	N-acyl-phosphatidylethanolamine-hydrolyzing phospholipase D. putative
56515	9.47	0.09	8.65	0.18	6.25	0.43	1.767 down	9.316 down	0.00	67.97	Regulation of HR towards crossover
56546	10.44	0.06	12.54	0.30	12.94	0.12	4.290 up	5.674 up	0.00	158.05	unknown protein. 3TM
56572	12.24	0.02	11.77	0.10	9.98	0.23	1.383 down	4.784 down	0.00	119.48	aminotransferase. classes I and II family
56587	10.36	0.11	10.03	0.01	7.95	0.21	1.259 down	5.312 down	0.00	174.73	GCN5-related N-acetyltransferase
56625	12.23	0.08	12.37	0.13	10.90	0.24	1.101 up	2.505 down	0.00	51.32	CoA-transferase family III

56646	9.02	0.05	8.64	0.01	7.47	0.48	1.307 down	2.940 down	0.02	13.82	monoamine oxidase
56671	7.62	0.20	8.53	0.02	8.85	0.43	1.885 up	2.349 up	0.04	8.60	unknown protein
56726	9.58	0.17	9.94	0.10	11.02	0.12	1.287 up	2.717 up	0.00	103.61	Branched chain alpha-keto acid dehydrogenase complex. alpha subunit
56758	9.59	0.06	10.00	0.03	11.07	0.23	1.330 up	2.799 up	0.00	54.97	unknown protein
56819	12.28	0.08	10.93	0.05	10.51	0.40	2.546 down	3.415 down	0.01	21.51	glutathione-S-transferase
56830	11.98	0.05	10.99	0.06	10.69	0.35	1.988 down	2.437 down	0.01	15.20	dipeptidyl peptidase 5
56831	11.38	0.07	11.70	0.15	12.45	0.19	1.247 up	2.100 up	0.00	31.92	Co-chaperone Hsc20
56840	9.02	0.01	6.32	0.14	11.77	0.62	6.499 down	6.709 up	0.00	86.62	unknown protein
56853	10.35	0.02	9.27	0.15	8.38	0.58	2.107 down	3.913 down	0.02	12.60	unknown protein
56860	11.21	0.01	10.24	0.00	9.37	0.31	1.964 down	3.588 down	0.00	39.91	unknown protein
56863	13.75	0.06	11.13	0.12	12.07	0.16	6.161 down	3.218 down	0.00	178.66	unknown protein
56894	2.88	0.23	3.09	0.17	4.26	0.10	1.156 up	2.606 up	0.00	72.96	GH18 chitinase CHI18-10
56996	4.93	0.29	4.85	0.10	8.93	0.21	1.054 down	15.988 up	0.00	362.01	GH5 β-Mannanase MAN1
57002	12.41	0.18	12.07	0.24	10.88	0.24	1.266 down	2.875 down	0.00	35.74	unknown protein
57008	5.24	0.40	5.55	0.15	6.96	0.49	1.238 up	3.284 up	0.02	13.77	unknown protein
57045	7.48	0.45	6.61	0.00	6.40	0.25	1.818 down	2.101 down	0.03	9.96	unknown protein
57049	12.79	0.04	12.13	0.01	11.52	0.31	1.578 down	2.408 down	0.01	19.50	tRNA selenocysteine-associated protein 1
57101	4.84	0.35	5.41	0.36	6.73	0.15	1.475 up	3.705 up	0.00	42.76	PTH11 GPCR
57112	9.08	0.17	8.87	0.10	7.80	0.41	1.156 down	2.420 down	0.02	12.86	HET and Ankyrin domain protein
57128	12.17	0.14	11.50	0.05	10.93	0.29	1.584 down	2.356 down	0.01	18.68	GH13 glycogen debranching enyzme
57179	5.10	0.27	5.27	0.06	8.18	0.57	1.125 up	8.449 up	0.00	42.77	GH105/GH88 glycosyl hydrolase
57185	7.09	0.18	9.06	0.15	8.36	0.64	3.912 up	2.408 up	0.04	7.87	Amino acid transporters
57198	10.39	0.09	9.74	0.19	8.55	0.27	1.567 down	3.568 down	0.00	47.77	Rad10
57286	8.85	0.11	9.96	0.19	10.10	0.38	2.161 up	2.381 up	0.02	11.46	unknown protein
57322	11.88	0.01	11.87	0.00	10.31	0.48	1.005 down	2.955 down	0.01	17.36	ARO Transcriptional regulators containing a DNA-binding HTH domain and an aminotr
57357	8.69	0.11	8.86	0.07	10.10	0.29	1.124 up	2.654 up	0.00	32.22	Glycine cleavage T protein (aminomethyl transferase). putative
57383	8.82	0.01	7.94	0.14	7.53	0.19	1.846 down	2.443 down	0.00	43.71	unknown protein
57397	6.76	0.18	7.98	0.11	8.28	0.42	2.316 up	2.850 up	0.02	13.47	unknown protein
57433	3.24	0.01	3.19	0.16	4.63	0.05	1.035 down	2.620 up	0.00	299.84	Peptidase S8 and S53. subtilisin. kexin. sedolisin
57465	12.29	0.03	12.19	0.10	11.13	0.13	1.074 down	2.235 down	0.00	104.32	Vsp9 domain protein

57488	12.93	0.18	12.73	0.02	11.70	0.26	1.149 down	2.350 down	0.01	27.75	Top3 gene
57524	12.44	0.12	12.02	0.05	11.39	0.18	1.336 down	2.065 down	0.00	35.65	amidohydrolase 2
57526	9.65	0.09	6.60	0.04	7.15	0.17	8.271 down	5.647 down	0.00	291.59	GPCR. mating type pheromone G-protein coupled receptor
57527	10.16	0.18	10.12	0.01	8.47	0.22	1.029 down	3.217 down	0.00	78.91	unknown protein
57537	11.64	0.03	12.02	0.23	12.78	0.21	1.299 up	2.213 up	0.01	27.11	unknown protein
57555	9.34	0.06	7.89	0.07	7.60	0.63	2.737 down	3.347 down	0.04	8.66	cytochrome P450 monooxygenase
57568	13.46	0.09	13.41	0.01	12.24	0.33	1.042 down	2.344 down	0.01	21.32	unknown protein
57575	4.83	0.22	5.06	0.44	6.37	0.39	1.168 up	2.904 up	0.02	14.45	unknown protein
57592	11.76	0.08	11.74	0.00	10.51	0.15	1.009 down	2.373 down	0.00	100.45	unknown protein
57603	12.83	0.22	12.30	0.03	11.76	0.08	1.449 down	2.098 down	0.00	58.36	unknown protein
57643	10.95	0.09	10.15	0.22	6.73	0.26	1.743 down	18.548 down	0.00	285.83	unknown protein
57735	12.87	0.02	12.39	0.05	11.85	0.24	1.398 down	2.025 down	0.01	20.26	GATA type transcriptional regulator
57749	10.71	0.10	9.68	0.10	8.80	0.43	2.035 down	3.764 down	0.01	21.78	MFS permease
57760	11.59	0.03	11.16	0.09	10.05	0.22	1.349 down	2.915 down	0.00	61.82	Zn2Cys6 transcriptional regulator
57860	11.60	0.02	11.40	0.05	9.77	0.15	1.153 down	3.578 down	0.00	225.07	Multicopper oxidases
57865	7.75	0.20	8.10	0.14	9.24	0.21	1.281 up	2.816 up	0.00	46.53	unknown protein
57868	8.31	0.03	9.60	0.04	9.35	0.19	2.442 up	2.062 up	0.00	42.56	Delta 1-pyrroline-5-carboxylate reductase
57870	10.16	0.03	11.42	0.18	11.80	0.28	2.398 up	3.119 up	0.00	34.63	histone H3
57891	12.23	0.02	12.12	0.09	11.10	0.11	1.078 down	2.186 down	0.00	125.15	actinin actin binding and calcium-binding EF-hand domains
57914	11.52	0.03	11.73	0.17	7.31	0.29	1.158 up	18.424 down	0.00	333.80	Esterase/lipase/thioesterase
57965	12.25	0.02	12.49	0.02	11.11	0.10	1.179 up	2.203 down	0.00	258.03	DSBA family oxidoreductase. putative
57975	7.05	0.29	7.90	0.03	8.78	0.20	1.804 up	3.326 up	0.00	52.08	phospholipase C. related to Aspergillus fumigatus phosphatidylinositol phospholipase
58016	5.03	0.31	5.53	0.33	6.67	0.09	1.417 up	3.118 up	0.00	43.99	unknown protein
58026	10.53	0.13	10.41	0.25	9.10	0.28	1.086 down	2.694 down	0.00	29.50	glutathione S-transferase domain-containing protein
58051	10.50	0.06	10.03	0.08	9.12	0.30	1.387 down	2.606 down	0.01	25.60	unknown protein
58130	12.99	0.00	12.35	0.01	11.82	0.06	1.563 down	2.246 down	0.00	384.56	bHLH transcriptional regulator
58227	5.12	0.25	6.29	0.30	7.12	0.31	2.248 up	4.000 up	0.00	30.50	GCN5-related acetyltransferase
58229	11.11	0.12	12.20	0.01	12.13	0.08	2.134 up	2.030 up	0.00	120.50	Ubiquitin-like protein (HubA)
58244	11.80	0.22	10.88	0.00	9.61	0.27	1.892 down	4.551 down	0.00	62.91	unknown protein
58264	9.47	0.00	10.27	0.15	10.83	0.09	1.747 up	2.575 up	0.00	132.85	paxU orthologue ? (indole-terpene biosynthesis?)

58267	9.48	0.08	9.99	0.11	11.07	0.12	1.428 up	3.018 up	0.00	161.89	tRNA-dihydrouridine synthase.
58285	7.34	0.13	6.36	0.25	6.28	0.24	1.981 down	2.096 down	0.01	16.42	PKS-NRPS
58296	3.94	0.02	4.09	0.05	5.36	0.34	1.107 up	2.676 up	0.01	26.87	MFS permease
58333	8.23	0.13	7.63	0.13	7.04	0.20	1.519 down	2.281 down	0.00	32.69	FAD binding protein
58366	3.84	0.14	3.48	0.11	5.40	0.33	1.280 down	2.944 up	0.00	42.64	AAA+-type ATPase
58370	10.26	0.11	9.36	0.08	9.03	0.18	1.865 down	2.339 down	0.00	43.72	mtDNA repair protein
58387	11.13	0.16	10.80	0.03	9.92	0.09	1.261 down	2.312 down	0.00	108.01	proline iminopeptidase
58412	7.15	0.34	5.26	0.33	5.35	0.15	3.697 down	3.471 down	0.00	42.07	AdhP Zn-dependent alcohol dehydrogenases
58431	3.82	0.50	4.30	0.05	5.38	0.17	1.400 up	2.947 up	0.01	27.42	unknown protein
58450	4.42	0.15	4.65	0.28	5.65	0.31	1.176 up	2.349 up	0.01	16.02	GH3 β-xylosidase XYL3b
58456	11.87	0.09	8.66	0.14	9.45	0.19	9.297 down	5.379 down	0.00	210.31	Zn2Cys6 transcriptional regulator
58479	7.77	0.19	7.26	0.02	6.02	0.29	1.418 down	3.365 down	0.00	41.47	short chain dehydrogenase/reductase
58492	9.80	0.05	10.28	0.30	10.94	0.43	1.397 up	2.204 up	0.05	7.08	unknown protein
58509	9.51	0.16	9.80	0.08	8.29	0.18	1.223 up	2.335 down	0.00	72.71	DNA ligase IV involved in non-homologous end joining of double-strand DNA breaks
58511	12.34	0.15	10.58	0.09	9.00	0.31	3.383 down	10.118 down	0.00	117.99	amino acid permease (GABA)
58521	3.48	0.09	3.69	0.07	5.32	0.33	1.160 up	3.571 up	0.00	43.52	SAM (and some other nucleotide) binding motif
58525	9.95	0.05	8.46	0.05	8.21	0.36	2.797 down	3.337 down	0.01	26.49	unknown protein
58535	3.48	0.18	4.04	0.12	6.53	0.32	1.475 up	8.270 up	0.00	108.76	unknown protein
58560	10.20	0.14	9.89	0.05	9.13	0.34	1.242 down	2.103 down	0.02	12.11	MFS monocarboxylate transporter
58563	4.47	0.16	4.48	0.16	5.52	0.17	1.004 up	2.070 up	0.00	40.40	oxalate decarboxylase
58574	5.98	0.48	6.11	0.10	7.36	0.49	1.092 up	2.601 up	0.03	9.18	unknown protein
58601	3.29	0.08	2.96	0.02	5.25	0.14	1.251 down	3.900 up	0.00	345.05	unknown protein
58603	9.61	0.06	9.71	0.04	11.84	0.19	1.070 up	4.702 up	0.00	207.27	unknown protein. contains Splecktrin motif
58634	11.64	0.04	11.05	0.06	9.92	0.15	1.499 down	3.290 down	0.00	152.86	Zn2Cys6 Fungal transcriptional regulator
58639	11.60	0.07	8.20	0.08	10.23	0.26	10.563 down	2.584 down	0.00	141.20	unknown protein
58640	9.82	0.01	10.42	0.32	11.39	0.26	1.516 up	2.972 up	0.00	29.24	unknown protein
58651	8.26	0.32	8.14	0.35	9.64	0.07	1.084 down	2.597 up	0.00	43.37	adenylosuccinate synthase
58675	7.64	0.15	6.98	0.24	5.59	0.57	1.577 down	4.152 down	0.01	15.22	short chain dehydrogenase/reductase
58689	8.40	0.18	8.05	0.11	6.96	0.28	1.277 down	2.717 down	0.00	30.31	ERG5 C-22 sterol desaturase. a cytochrome P450 enzyme that catalyzes the formation
58694	10.65	0.02	9.88	0.12	9.49	0.26	1.700 down	2.232 down	0.01	21.30	unknown protein

58699	8.44	0.04	7.69	0.03	7.15	0.15	1.682 down	2.440 down	0.00	78.86	unknown protein
58734	11.64	0.12	12.11	0.05	13.27	0.10	1.390 up	3.106 up	0.00	212.09	prefoldin subunit 2
58746	11.67	0.13	11.25	0.05	10.30	0.28	1.343 down	2.596 down	0.00	28.58	unknown protein
58823	5.99	0.02	6.60	0.18	7.41	0.48	1.531 up	2.674 up	0.03	9.91	MRSP1/expansin-like
58837	11.76	0.07	11.11	0.19	10.33	0.12	1.566 down	2.698 down	0.00	84.39	unknown protein
58848	5.62	0.12	5.23	0.14	6.97	0.44	1.314 down	2.539 up	0.01	20.27	unknown protein
58853	8.05	0.04	9.62	0.11	12.30	0.11	2.977 up	19.052 up	0.00	1288.61	Homeodomain-like
58880	3.72	0.11	3.66	0.19	6.13	0.25	1.041 down	5.309 up	0.00	122.25	MRSP1/expansin-like
58887	3.38	0.12	3.19	0.16	4.86	0.11	1.134 down	2.799 up	0.00	157.84	GH78 α-L-rhamnosidase
58897	13.35	0.04	12.76	0.08	11.55	0.08	1.497 down	3.468 down	0.00	494.37	C2H2 transcriptional regulator
58952	5.80	0.25	5.17	0.09	4.73	0.12	1.547 down	2.103 down	0.00	32.45	calcium transporting ATPase. ion pump
58990	8.94	0.13	9.51	0.06	10.01	0.23	1.491 up	2.112 up	0.01	22.78	v-SNARE Bos1. ER-Golgi
59028	6.69	0.03	7.71	0.05	9.02	0.26	2.016 up	4.997 up	0.00	92.48	unknown protein
59050	3.13	0.25	2.84	0.13	6.41	0.83	1.216 down	9.714 up	0.01	27.29	Ankyrin
59053	12.54	0.20	12.33	0.05	11.34	0.16	1.156 down	2.291 down	0.00	53.14	Adenosine/AMP deaminase
59065	4.17	0.24	4.59	0.01	5.63	0.20	1.339 up	2.752 up	0.00	47.64	unknown protein
59067	9.94	0.08	10.18	0.09	11.20	0.17	1.180 up	2.397 up	0.00	65.44	Zn2Cys6 transcriptional regulator
59073	9.73	0.06	9.58	0.00	8.58	0.24	1.110 down	2.213 down	0.00	34.08	unknown protein
59081	3.17	0.11	3.27	0.00	4.83	0.13	1.066 up	3.150 up	0.00	200.10	CsdB Selenocysteine lyase
59151	14.30	0.21	10.21	0.17	12.06	0.29	17.018 down	4.712 down	0.00	128.31	SSCRP
59188	10.60	0.15	10.93	0.19	8.50	0.22	1.257 up	4.261 down	0.00	126.19	unknown protein
59190	9.99	0.16	9.30	0.16	8.51	0.23	1.614 down	2.775 down	0.00	35.46	MFS permease
59196	7.52	0.11	8.59	0.00	9.54	0.53	2.101 up	4.067 up	0.01	16.18	short chain dehydrogenase/reductase
59234	10.43	0.02	10.02	0.27	8.45	0.49	1.330 down	3.943 down	0.01	20.24	Zn2Cys6 transcriptional regulator
59244	3.98	0.21	4.09	0.05	6.00	0.54	1.079 up	4.037 up	0.01	20.86	metalloprotease. putative
59272	9.49	0.05	9.10	0.37	7.89	0.33	1.314 down	3.044 down	0.01	22.28	MFS permease
59315	7.45	0.06	8.24	0.08	6.20	0.21	1.723 up	2.386 down	0.00	105.73	PKS
59322	9.45	0.06	8.23	0.13	6.90	0.33	2.327 down	5.863 down	0.00	63.76	Zinc-binding oxidoreductase
59333	2.96	0.12	3.24	0.16	4.97	0.46	1.215 up	4.018 up	0.01	26.13	MFS permease
59351	7.22	0.24	5.80	0.07	5.29	0.20	2.679 down	3.796 down	0.00	68.48	1-aminocyclopropane-1-carboxylate synthase

59352	10.50	0.02	9.77	0.11	8.63	0.70	1.659 down	3.656 down	0.04	8.47	cytochrome P450
59353	9.10	0.12	9.39	0.01	7.82	0.36	1.228 up	2.423 down	0.01	25.14	Zn2Cys6 transcriptional regulator
59354	9.52	0.08	8.52	0.04	7.91	0.25	2.000 down	3.047 down	0.00	45.72	Zn2Cys6 transcriptional regulator
59364	13.68	0.06	9.67	0.31	9.01	0.22	16.146 down	25.526 down	0.00	306.55	Sexual differentiation process protein ISP4
59368	8.63	0.10	11.15	0.14	13.71	0.19	5.742 up	33.950 up	0.00	640.15	unknown protein. Duf341
59372	8.22	0.02	3.77	0.10	6.25	0.09	21.869 down	3.935 down	0.00	1530.25	unknown protein
59381	11.06	0.02	10.38	0.10	8.26	0.30	1.602 down	6.982 down	0.00	110.68	SAM-dependent methyltransferases
59382	11.81	0.09	11.10	0.22	8.60	0.30	1.643 down	9.290 down	0.00	126.32	unknown protein
59391	10.26	0.06	10.21	0.05	7.97	0.53	1.035 down	4.898 down	0.00	30.35	GH27 α-galactosidase
59396	5.91	0.03	5.58	0.03	7.20	0.61	1.260 down	2.444 up	0.03	9.64	unknown protein
59482	9.72	0.01	11.14	0.01	12.11	0.51	2.690 up	5.271 up	0.01	25.01	PKS
59491	8.79	0.11	8.05	0.06	6.17	0.12	1.664 down	6.146 down	0.00	452.69	unknown protein
59508	4.71	0.08	4.18	0.40	5.92	0.32	1.443 down	2.313 up	0.01	24.23	dipeptidyl peptidase 5
59515	9.24	0.02	10.57	0.06	11.04	0.25	2.504 up	3.481 up	0.00	56.31	MFS permease
59546	11.91	0.06	11.38	0.13	10.83	0.40	1.441 down	2.105 down	0.04	8.09	Zn2Cys6 transcriptional regulator
59582	3.67	0.11	4.21	0.12	5.32	0.35	1.451 up	3.142 up	0.01	26.41	unknown protein
59584	10.15	0.01	10.87	0.19	11.58	0.53	1.644 up	2.685 up	0.04	8.09	unknown protein
59597	4.17	0.03	4.47	0.16	5.69	0.09	1.231 up	2.859 up	0.00	180.21	unknown protein
59598	11.81	0.05	10.90	0.09	9.72	0.17	1.883 down	4.278 down	0.00	161.81	unknown protein
59609	12.56	0.13	12.57	0.01	11.43	0.33	1.007 up	2.188 down	0.01	18.74	unknown protein
59628	6.60	0.38	7.02	0.28	8.66	0.29	1.342 up	4.185 up	0.00	36.47	unknown protein
59642	11.29	0.09	11.01	0.15	9.51	0.39	1.211 down	3.427 down	0.01	27.73	α/ß hydrolase lipase/epoxide hydrolase
59649	8.47	0.04	8.65	0.01	6.51	0.27	1.129 up	3.892 down	0.00	95.16	Zinc-containing alcohol dehydrogenase
59665	10.19	0.06	10.14	0.10	8.87	0.32	1.031 down	2.486 down	0.01	25.76	unknown protein
59669	10.53	0.14	10.64	0.10	9.41	0.20	1.081 up	2.171 down	0.00	44.92	extracellular salicylate hydroxylase/monooxygenase. putative
59689	9.46	0.12	9.18	0.02	8.18	0.23	1.219 down	2.438 down	0.00	39.28	GH2 β-mannosidase
59690	3.08	0.31	2.91	0.24	4.86	0.13	1.119 down	3.442 up	0.00	83.95	unknown protein
59726	2.84	0.02	2.93	0.00	5.25	0.09	1.062 up	5.297 up	0.00	1077.63	DNA photolyase. N-terminal. class 1. FAD-binding
59740	9.36	0.13	8.98	0.01	7.55	0.18	1.298 down	3.502 down	0.00	114.38	transcriptional regulator. unknown
59746	9.24	0.04	9.03	0.01	7.55	0.17	1.158 down	3.233 down	0.00	148.83	oxaloacetase-like protein

59747	13.01	0.05	12.51	0.20	11.61	0.24	1.417 down	2.643 down	0.00	32.98	Rho GTPase-activating protein involved in signal transduction mechanisms
59751	11.25	0.03	10.68	0.19	10.18	0.26	1.475 down	2.093 down	0.01	16.20	Ribonucleases P/MRP protein subunit POP1 containing protein
59763	5.92	0.03	7.41	0.01	7.98	0.19	2.804 up	4.151 up	0.00	128.55	unknown protein
59770	10.49	0.12	9.88	0.12	8.59	0.16	1.525 down	3.754 down	0.00	131.69	aryl-alcohol dehydrogenases
59778	12.93	0.13	12.79	0.02	10.38	0.31	1.104 down	5.861 down	0.00	101.79	GPCR. related to A nidulans GprC
59791	2.72	0.05	2.91	0.02	5.03	0.42	1.143 up	4.978 up	0.00	46.91	GH18. chitinase CHI18-15
59796	12.70	0.05	12.21	0.02	11.57	0.09	1.407 down	2.193 down	0.00	162.81	MFS permease
59801	7.65	0.13	7.54	0.03	9.29	0.27	1.082 down	3.116 up	0.00	62.35	unknown protein
59827	8.93	0.18	9.60	0.04	11.11	0.11	1.592 up	4.546 up	0.00	256.44	unknown protein
59833	11.55	0.02	10.42	0.02	9.97	0.26	2.185 down	2.973 down	0.00	40.48	unknown protein
59843	9.38	0.07	9.54	0.22	8.05	0.31	1.113 up	2.512 down	0.00	29.40	AMP-dependent synthetase and ligase. acetoacetyl-CoA synthase-like
59876	4.91	0.56	4.48	0.20	7.32	1.08	1.341 down	5.318 up	0.03	9.07	xenobiotic compound monoounknown proteingenase. DszA family
59887	8.48	0.38	6.05	0.32	6.37	0.16	5.385 down	4.318 down	0.00	58.83	L-lactate dehydrogenase. putative
59936	9.18	0.01	9.86	0.24	10.54	0.22	1.599 up	2.570 up	0.00	30.80	unknown protein
59940	12.05	0.09	10.15	0.17	10.43	0.51	3.732 down	3.082 down	0.02	13.95	unknown protein
59952	8.96	0.08	10.04	0.25	6.58	0.28	2.117 up	5.176 down	0.00	148.78	Amino acid transporter PotE
60004	12.02	0.20	11.60	0.23	10.61	0.28	1.338 down	2.648 down	0.01	23.70	Zn2Cys6 transcriptional regulator
60028	3.54	0.28	3.37	0.13	5.22	0.24	1.122 down	3.193 up	0.00	58.73	unknown protein
60052	10.16	0.02	9.23	0.17	8.07	0.08	1.895 down	4.237 down	0.00	310.61	short chain dehydrogenase/reductase
60116	13.65	0.08	12.95	0.13	10.50	0.54	1.630 down	8.900 down	0.00	45.58	MRP-type ABC transporter
60144	3.73	0.37	3.78	0.30	6.18	0.91	1.036 up	5.498 up	0.02	11.01	Amino acid transporters
60182	3.68	0.65	2.68	0.17	4.94	0.21	1.995 down	2.394 up	0.00	30.63	unknown protein
60187	7.16	0.03	6.88	0.01	5.97	0.14	1.217 down	2.281 down	0.00	93.56	enoyl-CoA hydratase/isomerase
60194	10.74	0.02	10.07	0.27	9.62	0.23	1.588 down	2.163 down	0.01	17.54	phosphatidylinositol-specific phospholipase C (MPLC1)
60232	10.82	0.00	11.12	0.11	9.26	0.24	1.226 up	2.958 down	0.00	83.82	unknown protein
60270	12.04	0.01	11.59	0.02	10.90	0.12	1.362 down	2.202 down	0.00	114.22	unknown protein
60282	12.04	0.01	11.64	0.14	10.70	0.20	1.318 down	2.532 down	0.00	49.07	Zn2Cys6 transcriptional regulator
60328	9.38	0.09	9.00	0.32	7.77	0.18	1.299 down	3.043 down	0.00	50.46	unknown protein
60337	6.92	0.03	4.09	0.42	5.37	0.21	7.097 down	2.932 down	0.00	63.70	unknown protein
60352	9.49	0.05	7.87	0.24	7.24	0.51	3.078 down	4.788 down	0.01	20.45	NADH-dehydrogenase (ubiquinone)

60370	11.50	0.16	9.04	0.03	9.83	0.41	5.514 down	3.175 down	0.00	29.72	unknown protein
60378	7.71	0.17	9.13	0.18	6.60	0.39	2.670 up	2.164 down	0.00	42.54	AAA ATPase
60418	4.81	0.09	5.35	0.10	6.31	0.36	1.458 up	2.832 up	0.01	20.38	Aldehyde dehydrogenase
60419	4.91	0.18	4.90	0.13	6.42	0.20	1.005 down	2.848 up	0.00	65.69	acid phosphatase. putative
60422	10.55	0.13	9.08	0.14	9.02	0.32	2.764 down	2.876 down	0.01	24.60	unknown protein
60445	11.42	0.08	9.64	0.02	8.23	0.35	3.424 down	9.081 down	0.00	90.72	unknown protein
60456	11.12	0.06	11.32	0.12	10.03	0.13	1.149 up	2.137 down	0.00	105.59	unknown protein
60489	11.14	0.02	9.73	0.12	12.34	0.39	2.666 down	2.296 up	0.00	49.05	CE5 cutinase
60490	10.90	0.10	11.05	0.13	9.80	0.15	1.104 up	2.155 down	0.00	75.00	2-nitropropane dioxygenase
60557	3.26	0.11	3.42	0.16	4.72	0.12	1.123 up	2.760 up	0.00	123.01	unknown protein
60560	7.92	0.05	2.92	0.02	6.80	0.47	31.933 down	2.174 down	0.00	110.22	unknown protein
60565	9.98	0.10	10.00	0.02	7.72	0.35	1.012 up	4.780 down	0.00	69.41	unknown protein
60578	11.71	0.14	11.53	0.08	10.52	0.15	1.131 down	2.268 down	0.00	60.74	Zn2Cys6 transcriptional regulator
60616	12.42	0.13	10.18	0.06	10.42	0.48	4.708 down	3.985 down	0.01	22.81	unknown protein
60634	11.26	0.17	11.47	0.02	10.02	0.17	1.156 up	2.372 down	0.00	81.34	transcriptional regulator. unknown
60635	5.36	0.26	4.25	0.18	10.05	1.91	2.154 down	25.709 up	0.02	12.76	GH92 α-1.2-mannosidase
60638	5.49	0.09	4.42	0.58	8.04	0.16	2.099 down	5.875 up	0.00	118.79	unknown protein
60664	12.18	0.08	11.87	0.01	11.02	0.18	1.239 down	2.244 down	0.00	51.98	unknown protein with PH domain (putative phosphatidylinositol 4.5-bisphosphate pro
60743	12.33	0.10	12.96	0.12	13.40	0.22	1.540 up	2.098 up	0.01	23.27	Dolichyl-phosphate mannosyltransferase polypeptide 3
60758	11.64	0.27	7.97	0.01	9.29	0.14	12.719 down	5.079 down	0.00	265.91	SAM-dependent methyltransferase
60761	12.98	0.07	11.97	0.27	11.15	0.31	2.016 down	3.554 down	0.00	31.87	transcriptional regulator Grainyhead/CP2
60771	11.89	0.21	12.60	0.17	13.05	0.17	1.632 up	2.232 up	0.00	28.61	prefoldin chaperone
60810	13.51	0.16	9.84	0.06	11.90	0.33	12.659 down	3.051 down	0.00	94.27	unknown protein. GPR1/FUN34/yaaH protein. 6TMs
60825	3.24	0.07	3.37	0.05	5.26	0.49	1.099 up	4.054 up	0.01	25.86	Cytochrome P450. putative
60835	11.57	0.14	11.43	0.30	10.40	0.31	1.105 down	2.255 down	0.01	15.70	PutA delta-1-pyrroline-5-carboxylate dehydrogenase
60847	13.10	0.07	12.68	0.19	12.06	0.16	1.334 down	2.048 down	0.00	33.98	Mitochondrial F1F0-ATP synthase. subunit c/ATP9/proteolipid
60850	10.28	0.15	10.08	0.08	9.16	0.34	1.142 down	2.173 down	0.02	14.53	Mitochondrial initiation factor 2 (IF-2).
60890	9.90	0.01	10.19	0.12	11.60	0.27	1.224 up	3.235 up	0.00	52.23	L-galactose dehydrogenase (L-GalDH). putative
60897	11.62	0.12	11.60	0.07	10.25	0.11	1.013 down	2.589 down	0.00	158.40	Zn2Cys6 transcriptional regulator
60931	8.79	0.05	9.21	0.12	7.76	0.24	1.335 up	2.042 down	0.00	42.29	Zn2Cys6 transcriptional regulator

60945	12.91	0.23	10.66	0.13	13.97	0.60	4.781 down	2.081 up	0.00	32.27	MFS permease
60949	8.58	0.08	9.51	0.01	7.19	0.47	1.906 up	2.613 down	0.00	29.48	unknown protein
60951	12.14	0.05	11.79	0.03	10.95	0.11	1.275 down	2.289 down	0.00	147.24	unknown protein
60981	9.70	0.05	9.64	0.06	8.29	0.26	1.043 down	2.651 down	0.00	46.87	unknown protein
60987	11.60	0.12	11.73	0.01	9.87	0.47	1.100 up	3.317 down	0.01	23.91	MRP-type ABC transporter
61020	7.06	0.14	7.86	0.13	8.28	0.17	1.740 up	2.330 up	0.00	38.37	thioesterase superfamily protein
61032	11.58	0.15	11.14	0.07	10.40	0.13	1.363 down	2.274 down	0.00	64.23	unknown protein
61055	6.01	0.25	5.13	0.02	7.78	0.95	1.851 down	3.390 up	0.03	9.56	unique protein
61066	9.13	0.18	8.61	0.03	7.74	0.33	1.434 down	2.615 down	0.01	19.18	GMC oxidoreductase
61116	12.72	0.18	12.77	0.06	11.61	0.29	1.039 up	2.148 down	0.01	22.18	ferric reductase
61121	10.07	0.00	9.86	0.09	8.80	0.23	1.160 down	2.413 down	0.00	42.56	arylsulfatase. putative
61127	13.21	0.12	12.23	0.03	11.50	0.38	1.975 down	3.274 down	0.01	21.81	Serine carboxypeptidase
61134	7.27	0.04	7.78	0.12	8.41	0.34	1.429 up	2.205 up	0.02	12.31	unknown protein
61190	9.22	0.23	10.04	0.01	10.37	0.44	1.766 up	2.218 up	0.05	7.00	BolA domain-containing protein
61222	9.09	0.08	8.89	0.16	7.39	0.34	1.149 down	3.238 down	0.00	34.24	unknown protein
61278	8.56	0.03	8.33	0.11	7.47	0.32	1.175 down	2.137 down	0.01	15.29	MFS permease
61293	9.03	0.11	8.99	0.01	7.62	0.22	1.030 down	2.666 down	0.00	60.19	dipeptidyl peptidase 5
61383	11.92	0.06	11.04	0.01	10.48	0.23	1.849 down	2.716 down	0.00	41.73	unknown protein
61441	12.38	0.07	11.75	0.04	11.21	0.18	1.542 down	2.246 down	0.00	42.19	unknown protein
61476	9.73	0.17	9.23	0.11	7.79	0.25	1.409 down	3.821 down	0.00	66.49	Zn2Cys6 transcriptional regulator
61496	10.63	0.01	10.58	0.03	12.10	0.03	1.036 down	2.757 up	0.00	2729.14	MFS permease
61504	10.43	0.23	9.36	0.21	8.53	0.56	2.093 down	3.731 down	0.02	11.56	unknown protein
61526	9.20	0.04	7.93	0.00	7.44	0.15	2.413 down	3.380 down	0.00	145.06	unknown protein
61553	9.68	0.07	7.18	0.02	7.46	0.26	5.669 down	4.651 down	0.00	96.49	D-xylulose 5-phosphate/D-fructose 6-phosphate phosphoketolase
61605	5.92	0.21	9.20	0.11	7.21	0.28	9.729 up	2.443 up	0.00	94.03	Vacuolar sorting protein VPS1. dynamin. and related proteins
61618	4.46	0.13	4.71	0.15	6.16	0.29	1.187 up	3.255 up	0.00	42.56	unknown protein
61632	11.08	0.11	12.17	0.25	12.10	0.12	2.128 up	2.019 up	0.00	33.76	unknown protein
61642	13.96	0.09	13.01	0.10	12.28	0.21	1.927 down	3.207 down	0.00	66.28	unknown protein. secreted
61703	6.94	0.05	7.33	0.02	8.10	0.26	1.316 up	2.241 up	0.01	25.10	germinal center kinase. related to S. cerevisiae Kic1
61798	11.23	0.14	11.12	0.04	12.41	0.08	1.072 down	2.271 up	0.00	198.75	unknown protein. only in ascomycota

61830	4.61	0.55	4.11	0.37	6.16	0.28	1.405 down	2.945 up	0.01	24.99	SSCRP
61858	3.03	0.06	2.61	0.04	4.52	0.20	1.334 down	2.801 up	0.00	119.91	unknown protein
61907	11.25	0.04	11.25	0.13	10.21	0.23	1.002 up	2.049 down	0.00	29.21	unknown protein
61910	9.34	0.06	9.82	0.09	8.33	0.22	1.396 up	2.016 down	0.00	52.74	Aldehyde dehydrogenase
61939	11.89	0.02	11.83	0.08	13.48	0.23	1.044 down	3.007 up	0.00	77.56	unknown protein
61948	3.91	0.31	3.44	0.27	5.61	0.21	1.383 down	3.258 up	0.00	65.12	Protein disulfide isomerase 2
61987	9.69	0.10	9.53	0.16	8.65	0.21	1.122 down	2.063 down	0.01	27.90	unknown protein
61995	12.44	0.02	12.54	0.19	13.62	0.16	1.071 up	2.258 up	0.00	56.93	unknown protein
62034	10.78	0.05	10.03	0.05	8.60	0.43	1.683 down	4.546 down	0.00	32.03	unknown protein
62086	9.38	0.10	10.02	0.14	10.73	0.20	1.555 up	2.551 up	0.00	43.23	unknown protein
62114	8.20	0.32	7.22	0.30	6.32	0.27	1.978 down	3.675 down	0.00	28.93	unknown protein
62153	10.73	0.02	10.14	0.15	9.58	0.12	1.503 down	2.219 down	0.00	69.62	malate dehydrogenase
62171	8.05	0.16	7.86	0.22	10.58	0.38	1.142 down	5.770 up	0.00	68.47	MFS permease
62181	12.10	0.07	12.00	0.01	10.69	0.12	1.073 down	2.658 down	0.00	191.50	Protein kinase. Ca2+-dependent
62213	14.16	0.01	14.13	0.03	12.27	0.10	1.021 down	3.707 down	0.00	600.43	unknown protein with fasciclin domain
62255	2.78	0.22	2.90	0.31	4.59	0.25	1.092 up	3.526 up	0.00	46.53	lipase. lipocalin related
62285	11.93	0.01	11.65	0.15	10.32	0.14	1.215 down	3.054 down	0.00	136.68	unknown protein
62323	8.77	0.16	9.14	0.06	7.62	0.39	1.296 up	2.216 down	0.01	18.98	unknown protein
62333	3.87	0.01	3.88	0.40	5.81	0.23	1.003 up	3.830 up	0.00	57.41	unknown protein
62340	9.34	0.12	10.87	0.03	10.75	0.10	2.892 up	2.669 up	0.00	172.16	unknown protein
62380	13.37	0.03	9.16	0.06	11.97	0.21	18.482 down	2.628 down	0.00	327.92	MFS permease (galactose permease ?)
62401	12.21	0.11	12.02	0.04	10.91	0.20	1.136 down	2.453 down	0.00	53.69	guanine nucleotide exchange factor synembryn. putative
62424	11.09	0.24	10.87	0.09	9.39	0.24	1.165 down	3.248 down	0.00	51.37	UDP-glucose:sterol b-glucosyltransferase
62447	12.86	0.01	12.11	0.07	10.56	0.15	1.686 down	4.938 down	0.00	276.92	unknown protein
62462	11.76	0.01	11.20	0.08	13.04	0.28	1.475 down	2.438 up	0.00	53.68	PTH11 GPCR
62477	8.96	0.04	10.13	0.17	10.32	0.27	2.257 up	2.568 up	0.01	26.63	Vacuolar protein sorting-associated protein Vps28
62480	9.47	0.08	10.22	0.32	10.50	0.16	1.686 up	2.049 up	0.01	19.05	mitochondrial tricarboxylate transporter (Ctp). putative
62484	10.98	0.03	9.45	0.01	9.00	0.28	2.877 down	3.937 down	0.00	55.93	unknown protein
62488	10.02	0.01	11.62	0.09	11.50	0.18	3.022 up	2.780 up	0.00	79.12	MFS permease
62502	12.31	0.05	12.66	0.08	10.98	0.14	1.270 up	2.522 down	0.00	180.09	MFS permease

62556	10.86	0.07	9.78	0.07	9.20	0.34	2.118 down	3.153 down	0.01	25.49	unknown protein
62611	13.56	0.35	12.64	0.28	10.43	0.17	1.888 down	8.764 down	0.00	131.15	MRP-type ABC transporter
62651	12.13	0.20	11.73	0.22	9.71	0.27	1.323 down	5.377 down	0.00	81.82	long-chain fatty acid transporter.
62663	7.26	0.13	7.55	0.03	8.61	0.30	1.221 up	2.548 up	0.01	25.32	unknown protein
62693	12.91	0.13	12.96	0.04	8.73	0.29	1.031 up	18.111 down	0.00	326.74	ABC-transporter Ste6p
62704	9.85	0.03	9.60	0.00	8.73	0.07	1.191 down	2.177 down	0.00	382.90	GH18. chitinase CHI18-3
62711	13.76	0.04	13.44	0.07	12.72	0.13	1.252 down	2.050 down	0.00	67.51	amino acid transporter
62716	5.18	0.15	6.46	0.05	14.19	0.12	2.434 up	515.554 up	0.00	5318.91	Ctr copper transporter family protein
62805	10.55	0.11	11.06	0.05	9.09	0.48	1.427 up	2.758 down	0.01	21.90	C2H2 transcriptional regulator
62836	12.48	0.02	12.02	0.13	11.01	0.24	1.377 down	2.777 down	0.00	44.20	unknown protein
62872	13.50	0.04	14.52	0.00	10.24	0.17	2.024 up	9.608 down	0.00	801.65	unknown protein GPR1/FUN34/yaaH-like
62912	10.88	0.15	11.74	0.19	12.25	0.42	1.810 up	2.580 up	0.02	10.77	unknown protein. 1TM
62977	8.80	0.15	7.33	0.05	7.13	0.44	2.778 down	3.175 down	0.01	15.77	nucleoside diphosphate sugar epimerase. secreted
63007	11.14	0.03	10.48	0.02	9.22	0.27	1.582 down	3.793 down	0.00	63.31	meiotic chromosome segregation protein. putative
63152	11.92	0.01	11.75	0.02	10.65	0.16	1.122 down	2.397 down	0.00	89.81	Cut9 interacting protein Scn1. putative
63163	10.24	0.18	10.27	0.23	9.17	0.22	1.015 up	2.105 down	0.01	24.63	unknown protein
63202	11.99	0.00	11.71	0.06	9.79	0.18	1.213 down	4.578 down	0.00	213.52	alpha/beta hydrolase.
63204	13.22	0.12	12.94	0.11	12.10	0.13	1.214 down	2.179 down	0.00	68.76	flavodoxin domain containing protein
63379	9.28	0.09	10.30	0.24	10.57	0.29	2.029 up	2.439 up	0.01	17.80	RNA polymerase II transcriptional coactivator. putative
63395	11.49	0.10	12.00	0.11	10.26	0.24	1.427 up	2.347 down	0.00	60.27	Glycerophosphoryl diester phosphodiesterase
63400	12.12	0.00	11.74	0.15	11.04	0.42	1.303 down	2.106 down	0.04	7.62	mitochondrial exoribonuclease Cyt-4
63503	9.36	0.01	9.85	0.10	10.52	0.10	1.398 up	2.238 up	0.00	120.72	rrbosomal protein S2 and tRNA/rRNA methyltransferase.
63526	7.20	0.11	8.40	0.21	9.86	0.76	2.301 up	6.318 up	0.02	13.88	SSCRP
63653	8.36	0.13	11.65	0.17	10.78	0.54	9.788 up	5.352 up	0.00	32.50	ankyrin
63687	10.12	0.02	8.04	0.18	9.01	0.57	4.207 down	2.154 down	0.03	10.58	ankyrin
63692	8.79	0.06	8.36	0.14	7.34	0.12	1.343 down	2.729 down	0.00	114.60	dipeptidyl peptidase 5
63702	11.90	0.08	11.48	0.02	10.66	0.10	1.342 down	2.367 down	0.00	146.37	unknown protein
63710	3.49	0.48	3.17	0.17	4.57	0.08	1.246 down	2.107 up	0.01	28.05	MMR; HR regulation
63727	11.71	0.11	11.71	0.04	10.56	0.06	1.004 down	2.219 down	0.00	241.90	Acyl-CoA dehydrogenase. central region
63756	9.82	0.01	8.34	0.18	8.47	0.23	2.781 down	2.539 down	0.00	38.77	helicase. DEAD-box superfamily

63815	8.24	0.36	8.31	0.10	10.56	0.27	1.051 up	4.989 up	0.00	71.74	Membrane coat complex Retromer. subunit Vps26
63837	6.97	0.22	6.87	0.12	5.60	0.19	1.072 down	2.580 down	0.00	51.05	unknown protein
63868	12.27	0.17	10.83	0.29	10.74	0.23	2.728 down	2.899 down	0.00	30.82	unknown protein
63881	8.65	0.11	9.81	0.28	9.80	0.26	2.236 up	2.218 up	0.01	17.07	unknown protein
63899	7.43	0.28	8.07	0.10	9.65	0.43	1.554 up	4.639 up	0.00	29.96	unknown protein
63914	12.17	0.14	11.35	0.04	10.52	0.24	1.761 down	3.150 down	0.00	50.23	integral membrane protein. putative
63919	9.02	0.04	10.16	0.02	10.47	0.17	2.195 up	2.727 up	0.00	81.27	Cys/Met metabolism PLP-dependent enzyme
63966	4.65	0.19	4.61	0.18	6.45	0.10	1.027 down	3.494 up	0.00	172.27	high-affinity glucose transporter
63978	11.83	0.04	11.65	0.07	10.51	0.15	1.133 down	2.490 down	0.00	96.69	Zn2Cys6 transcriptional regulator
64018	13.00	0.01	13.15	0.10	11.32	0.32	1.107 up	3.214 down	0.00	48.23	GPCR. mating type pheromone G-protein coupled receptor
64029	11.06	0.14	10.75	0.02	9.77	0.22	1.233 down	2.447 down	0.00	40.91	folylpolyglutamate synthase . putative
64049	6.14	0.38	6.05	0.29	9.12	0.88	1.064 down	7.850 up	0.01	17.94	unknown protein
64066	11.95	0.08	12.21	0.02	10.50	0.15	1.196 up	2.742 down	0.00	171.56	acyltransferase 3
64172	8.33	0.03	9.00	0.11	9.77	0.32	1.590 up	2.704 up	0.01	21.85	Glutathione-dependent formaldehyde-activating.GFA
64181	6.49	0.22	7.41	0.03	7.77	0.48	1.885 up	2.427 up	0.04	7.51	SSCRP
64288	2.96	0.43	2.71	0.24	4.22	0.16	1.188 down	2.399 up	0.00	30.72	unknown protein
64295	13.49	0.01	13.17	0.06	12.03	0.09	1.242 down	2.735 down	0.00	282.73	nitrogen permease regulator Npr2. putative
64312	10.82	0.11	9.86	0.19	8.77	0.43	1.953 down	4.165 down	0.01	23.90	unknown protein. only present in fungi
64318	10.80	0.01	11.17	0.15	11.86	0.26	1.295 up	2.086 up	0.01	19.14	unknown protein
64370	11.10	0.00	9.92	0.15	8.06	0.24	2.259 down	8.211 down	0.00	165.00	calpain-like protease
64448	12.64	0.05	13.42	0.08	14.15	0.08	1.720 up	2.858 up	0.00	281.90	unknown protein
64469	12.17	0.15	11.25	0.24	11.06	0.20	1.902 down	2.170 down	0.01	22.17	Hydrolases of alpha/beta hydrolase superfamily
64545	11.63	0.04	10.44	0.03	9.74	0.15	2.286 down	3.702 down	0.00	168.21	MFS permease
64620	11.03	0.01	10.56	0.01	9.87	0.26	1.383 down	2.236 down	0.01	24.39	Alpha/beta hydrolase
64667	13.55	0.07	11.55	0.19	11.83	0.47	4.014 down	3.283 down	0.01	18.11	unknown protein
64672	9.61	0.22	9.23	0.11	7.29	0.11	1.298 down	4.986 down	0.00	235.56	Helix-turn-helix. AraC type
64676	8.24	0.07	10.19	0.25	9.52	0.26	3.856 up	2.420 up	0.00	36.17	unknown protein
64710	10.98	0.12	10.52	0.18	8.94	0.16	1.372 down	4.096 down	0.00	136.61	AAA+-type ATPase
64719	10.24	0.05	9.40	0.03	9.06	0.17	1.795 down	2.267 down	0.00	53.49	Subtilisin like protease (SUB3)
64827	11.92	0.06	11.54	0.10	10.23	0.27	1.302 down	3.227 down	0.00	49.77	GH36 raffinose synthase domain protein

64834	11.00	0.04	10.78	0.08	9.57	0.24	1.158 down	2.680 down	0.00	49.76	unknown protein
64874	13.37	0.10	13.72	0.17	12.13	0.24	1.271 up	2.365 down	0.00	48.77	MFS toxin efflux pump
64882	10.29	0.11	8.34	0.20	8.51	0.19	3.852 down	3.417 down	0.00	79.16	MFS permease
64916	11.05	0.06	11.80	0.04	9.60	0.77	1.689 up	2.720 down	0.03	10.02	unknown protein
64919	4.55	0.27	5.05	0.22	6.26	0.26	1.410 up	3.266 up	0.00	33.76	MFS permease
64925	11.35	0.10	12.38	0.07	12.56	0.17	2.051 up	2.320 up	0.00	50.56	GT32 a-glycosyltransferase
64937	7.31	0.09	7.37	0.03	8.69	0.53	1.045 up	2.616 up	0.02	10.81	unknown protein
64951	3.80	0.16	3.60	0.06	5.64	0.39	1.147 down	3.583 up	0.00	38.84	unknown protein
64956	3.21	0.29	3.66	0.18	4.89	0.21	1.370 up	3.208 up	0.00	45.55	unknown protein
64959	11.30	0.18	7.57	0.01	10.02	0.18	13.328 down	2.437 down	0.00	274.98	phosphatidyl synthase
64971	9.89	0.04	8.87	0.10	8.54	0.46	2.033 down	2.559 down	0.03	9.51	Amino acid permeases
64996	3.95	0.08	6.69	0.10	6.46	0.79	6.698 up	5.718 up	0.02	13.53	nitrilase
65021	5.65	0.13	6.06	0.20	7.25	0.41	1.331 up	3.039 up	0.01	18.30	short chain dehydrogenase/reductase
65029	12.94	0.00	12.60	0.05	11.81	0.35	1.263 down	2.182 down	0.02	13.39	2OG-Fe(II) oxygenase superfamily protein
65037	5.95	0.01	5.27	0.04	7.18	0.09	1.602 down	2.345 up	0.00	490.32	pyrophosphatase
65040	3.38	0.10	3.25	0.27	4.77	0.14	1.088 down	2.634 up	0.00	76.58	cytochrome P450. putative
65067	4.31	0.19	3.41	0.19	6.07	0.24	1.860 down	3.403 up	0.00	105.24	short chain dehydrogenase/reductase
65095	5.17	0.12	5.36	0.11	6.42	0.12	1.135 up	2.378 up	0.00	97.83	unknown protein
65096	6.81	0.24	7.26	0.14	8.09	0.42	1.360 up	2.422 up	0.03	10.00	3'-tRNA processing endoribonuclease
65098	3.78	0.06	4.25	0.01	5.88	0.11	1.379 up	4.278 up	0.00	482.28	FAD-binding domain and SignalP-predicted secretion signal. Distantly related to tre36[illegible]
65102	3.65	0.38	3.66	0.36	5.02	0.18	1.007 up	2.577 up	0.01	24.56	unknown protein
65106	4.46	0.08	4.58	0.32	9.24	1.03	1.083 up	27.491 up	0.00	33.64	CN_hydrolase
65107	11.66	0.07	11.22	0.08	10.41	0.10	1.357 down	2.374 down	0.00	142.19	cytochrome P450
65116	4.04	0.56	4.29	0.02	5.36	0.25	1.187 up	2.497 up	0.02	14.62	PKS
65117	6.15	0.40	4.67	0.43	4.87	0.16	2.782 down	2.425 down	0.01	16.37	ankyrin
65133	7.83	0.10	8.29	0.13	6.33	0.54	1.370 up	2.840 down	0.01	17.26	Zn2Cys6 transcriptional regulator
65141	6.54	0.15	6.57	0.13	7.80	0.31	1.022 up	2.394 up	0.01	23.45	cytochrome P450 monooxygenase
65142	9.07	0.05	9.92	0.05	10.32	0.26	1.800 up	2.379 up	0.01	25.89	aldehyde dehydrogenase ALDH
65153	9.45	0.10	9.48	0.13	8.43	0.33	1.019 up	2.030 down	0.01	15.53	MFS permease
65156	6.32	0.19	7.24	0.10	7.74	0.31	1.887 up	2.672 up	0.01	19.81	S1/P1 nuclease

65162	10.94	0.19	12.83	0.03	13.37	0.14	3.710 up	5.381 up	0.00	210.52	GH18 endo-N-acetyl-ß-D-glucosaminidase Endo T
65164	12.52	0.16	11.75	0.02	11.22	0.19	1.711 down	2.468 down	0.00	43.30	unknown protein
65172	5.02	0.17	5.05	0.04	6.17	0.21	1.018 up	2.221 up	0.00	38.75	PKS
65223	8.89	0.14	9.58	0.04	7.79	0.13	1.609 up	2.156 down	0.00	160.57	unknown protein
65229	9.68	0.08	11.34	0.01	10.95	0.12	3.163 up	2.411 up	0.00	147.35	unknown protein
65232	9.03	0.19	9.21	0.08	7.41	0.39	1.132 up	3.068 down	0.00	29.32	short chain dehydrogenase/reductase
65286	9.49	0.02	9.79	0.16	8.15	0.13	1.227 up	2.536 down	0.00	147.30	unknown protein
65315	12.82	0.06	8.79	0.13	9.29	0.77	16.360 down	11.572 down	0.00	29.31	bZIP transcription factor
65324	8.73	0.00	9.42	0.19	11.30	0.23	1.609 up	5.921 up	0.00	135.59	metacaspase CasA
65333	12.54	0.01	11.26	0.24	10.24	0.25	2.438 down	4.942 down	0.00	72.53	GH15 alpha-glycosidase (Glucoamylase and related glycosyl hydrolases)
65380	10.51	0.12	11.99	0.04	11.93	0.26	2.788 up	2.669 up	0.00	36.52	GH47 α-1.2-mannosidase
65494	9.71	0.06	11.70	0.13	11.08	0.58	3.957 up	2.590 up	0.03	10.24	metallopeptidase
65499	5.29	0.43	5.16	0.09	6.80	0.20	1.096 down	2.838 up	0.00	39.00	Tyrosine specific protein phosphatase and dual specificity protein phosphatase
65508	13.05	0.10	12.90	0.03	11.76	0.27	1.109 down	2.452 down	0.00	31.46	fructose-bisphosphatase
65522	11.90	0.17	8.57	0.05	9.84	0.55	10.051 down	4.155 down	0.00	29.92	unknown protein
65547	8.18	0.01	8.48	0.02	6.52	0.10	1.235 up	3.156 down	0.00	559.48	mandelate racemase/muconate lactonase-like protein
65572	11.88	0.26	11.70	0.00	10.48	0.35	1.131 down	2.638 down	0.01	19.98	unknown protein
65603	5.97	0.05	6.76	0.03	7.41	0.10	1.730 up	2.704 up	0.00	218.43	unknown protein
65607	12.60	0.06	12.50	0.17	11.41	0.17	1.071 down	2.279 down	0.00	54.57	RgsC. regulator of G-protein signaling
65640	7.63	0.06	8.39	0.00	9.44	0.17	1.692 up	3.499 up	0.00	122.56	unknown protein
65711	12.41	0.09	13.00	0.02	13.87	0.17	1.504 up	2.757 up	0.00	77.00	SAM-dependent methyltransferase
65718	14.38	0.00	13.13	0.14	13.04	0.10	2.383 down	2.523 down	0.00	127.25	unknown protein
65736	5.12	0.22	6.20	0.12	7.28	0.27	2.122 up	4.485 up	0.00	57.97	catabolic 3-dehydroquinase
65739	10.32	0.10	9.69	0.05	8.08	0.36	1.548 down	4.721 down	0.00	49.11	unknown protein
65746	11.35	0.07	11.13	0.11	9.96	0.23	1.162 down	2.609 down	0.00	47.32	Zn2Cys6 transcriptional regulator
65768	12.51	0.05	12.86	0.20	11.48	0.35	1.275 up	2.053 down	0.01	18.56	unknown protein
65771	3.32	0.15	3.49	0.21	6.02	0.25	1.125 up	6.530 up	0.00	133.71	NACHT domain WD40 repeat-containing protein. related to HET
65817	9.57	0.03	7.01	0.13	7.80	0.21	5.911 down	3.410 down	0.00	115.75	GT α-1.3-mannosyltransferase CMT1
65819	7.73	0.13	7.17	0.01	6.41	0.37	1.477 down	2.505 down	0.02	14.34	Molecular chaperone Hsp70 family
65821	11.91	0.22	11.69	0.07	10.83	0.10	1.160 down	2.113 down	0.00	57.75	vacuolar targeting protein Atg18

65873	10.68	0.14	10.13	0.08	9.49	0.03	1.464 down	2.278 down	0.00	166.62	protein kinase A. catalytic subunit
65882	10.26	0.13	9.82	0.03	8.99	0.14	1.350 down	2.411 down	0.00	77.58	dihydrodipicolinate synthase . putative
65883	10.29	0.13	8.07	0.26	8.24	0.39	4.669 down	4.131 down	0.00	30.96	D-isomer-specific 2-hydroxy acid dehydrogenase
65891	10.76	0.08	10.10	0.05	9.38	0.52	1.574 down	2.603 down	0.04	7.94	PKS
65921	7.81	0.02	7.80	0.02	9.58	0.47	1.005 down	3.414 up	0.01	23.91	Acetyl/propionyl-CoA carboxylase alpha subunit
65925	6.90	0.08	7.27	0.12	8.09	0.20	1.289 up	2.282 up	0.00	39.42	cytidine and deoxycytidylate deaminase zinc-binding region
65933	6.13	0.30	7.21	0.06	7.25	0.27	2.112 up	2.174 up	0.01	14.69	Epl1/Sm1
65949	8.32	0.13	11.21	0.09	9.47	0.36	7.410 up	2.214 up	0.00	50.95	unknown protein
65950	8.29	0.04	8.54	0.06	6.74	0.14	1.188 up	2.931 down	0.00	221.30	unknown protein
65957	9.80	0.06	9.77	0.00	8.74	0.12	1.022 down	2.086 down	0.00	111.23	unknown protein
65975	9.70	0.08	9.01	0.06	8.22	0.46	1.609 down	2.796 down	0.02	11.72	unknown protein
65986	10.77	0.12	10.22	0.16	9.33	0.42	1.463 down	2.718 down	0.02	13.15	GH27 α-galactosidase
65997	9.57	0.20	10.38	0.00	11.25	0.18	1.751 up	3.188 up	0.00	68.40	Cyclophilin type peptidyl-prolyl cis-trans isomerase
66016	12.23	0.01	10.16	0.14	10.62	0.12	4.207 down	3.062 down	0.00	202.79	dienelactone hydrolase
66023	9.71	0.09	9.67	0.03	8.31	0.22	1.028 down	2.627 down	0.00	61.18	unknown protein
66034	7.76	0.27	7.61	0.06	5.19	0.58	1.104 down	5.924 down	0.00	28.44	unknown protein
66077	3.39	0.09	3.19	0.11	5.34	0.15	1.149 down	3.847 up	0.00	238.23	ooc1-related protein
66092	11.38	0.02	8.31	0.38	9.94	0.22	8.365 down	2.706 down	0.00	81.94	unique protein
66103	12.17	0.14	13.09	0.05	13.45	0.10	1.891 up	2.429 up	0.00	99.41	WbbJ Acetyltransferase (isoleucine patch superfamily)
66210	8.39	0.02	8.94	0.04	9.54	0.18	1.461 up	2.221 up	0.00	47.52	unknown protein
66228	5.97	0.03	6.21	0.05	7.75	0.55	1.186 up	3.451 up	0.01	15.54	unknown protein
66268	13.01	0.14	12.89	0.11	11.79	0.14	1.083 down	2.323 down	0.00	75.40	unknown protein
66345	11.79	0.03	11.50	0.07	14.57	0.13	1.222 down	6.910 up	0.00	773.21	Mn superoxide dismutase
66370	4.39	0.03	4.25	0.06	6.58	0.51	1.105 down	4.544 up	0.00	32.25	unknown protein
66394	11.29	0.08	11.42	0.02	9.71	0.19	1.099 up	2.982 down	0.00	120.69	DNA polymerase family X member. Most closely related to DNA polymerase mu. an er activity.
66510	2.93	0.31	2.80	0.13	4.38	0.16	1.090 down	2.723 up	0.00	60.85	short chain dehydrognease/reductase
66521	11.51	0.19	12.70	0.07	10.42	0.23	2.283 up	2.125 down	0.00	88.69	unknown protein
66534	9.56	0.00	5.71	0.08	7.80	1.11	14.354 down	3.374 down	0.03	9.99	Cytochrome P450 CYP2 subfamily
66551	7.18	0.13	5.93	0.14	5.89	0.13	2.368 down	2.445 down	0.00	71.41	AAA ATPase

66563	10.78	0.03	11.87	0.00	12.27	0.18	2.132 up	2.812 up	0.00	73.89	unknown protein
66598	10.69	0.05	9.09	0.02	9.10	0.37	3.037 down	3.008 down	0.01	23.40	NADPH:quinone oxidoreductase
66606	9.73	0.08	10.06	0.03	8.49	0.31	1.256 up	2.361 down	0.00	33.93	Zn2Cys6 transcriptional regulator
66614	9.91	0.01	9.69	0.21	7.95	0.31	1.167 down	3.892 down	0.00	50.23	alpha/beta hydrolase
66657	9.19	0.28	8.61	0.15	7.33	0.47	1.494 down	3.626 down	0.01	16.97	MFS permease
66662	11.31	0.10	11.11	0.08	9.71	0.27	1.148 down	3.023 down	0.00	46.98	Mandelate racemase/muconate lactonizing protein
66726	8.99	0.02	5.58	0.08	7.84	1.11	10.629 down	2.210 down	0.04	8.31	FAD binding protein
66751	5.11	0.00	4.77	0.04	6.27	0.63	1.261 down	2.246 up	0.04	7.79	unknown protein
66753	6.41	0.13	6.50	0.05	8.15	0.44	1.065 up	3.341 up	0.01	23.42	unknown protein
66758	7.13	0.00	6.56	0.01	8.21	0.55	1.479 down	2.110 up	0.02	10.88	Ankyrin
66766	4.42	0.20	3.60	0.10	8.68	0.06	1.758 down	19.235 up	0.00	1788.24	Carbon-nitrogen hydrolase
66776	6.90	0.31	6.42	0.19	8.17	0.63	1.399 down	2.410 up	0.03	9.01	unknown protein
66786	12.32	0.00	10.09	0.12	10.92	0.37	4.715 down	2.649 down	0.00	30.29	unknown protein
66788	9.09	0.10	11.05	0.08	11.68	0.69	3.890 up	5.984 up	0.01	15.41	unknown protein
66804	11.01	0.08	10.55	0.13	9.65	0.29	1.376 down	2.571 down	0.01	26.01	GH69: candidate a-glycosyltransferase
66819	13.16	0.13	12.89	0.11	12.05	0.29	1.208 down	2.170 down	0.01	17.56	Amino acid transporter LysP
66827	9.66	0.03	9.34	0.04	7.57	0.34	1.251 down	4.262 down	0.00	55.20	Zinc-containing alcohol dehydrogenase
66828	13.58	0.20	14.26	0.14	12.33	0.22	1.603 up	2.380 down	0.00	69.86	Zn2Cys6 transcriptional regulator
66859	3.22	0.10	3.49	0.36	5.47	0.24	1.205 up	4.750 up	0.00	70.38	serine peptidase S28
66865	9.52	0.02	7.81	0.04	7.50	0.25	3.263 down	4.034 down	0.00	71.77	unknown protein
66877	9.95	0.05	9.82	0.00	8.95	0.12	1.094 down	2.003 down	0.00	104.02	unknown protein
66888	11.01	0.03	7.44	0.09	8.61	0.33	11.913 down	5.286 down	0.00	101.58	GT α-1.3-mannosyltransferase
66895	9.51	0.06	10.49	0.28	11.79	0.17	1.971 up	4.834 up	0.00	108.65	3' exoribonuclease
66937	12.09	0.04	11.31	0.02	10.06	0.23	1.717 down	4.094 down	0.00	95.00	FAD binding domain-containing protein
66950	9.60	0.18	9.63	0.07	8.28	0.39	1.020 up	2.504 down	0.01	17.85	serine/threonine protein kinase.
66999	5.65	0.04	5.09	0.19	8.42	1.00	1.480 down	6.807 up	0.01	15.61	AMP-dependent synthetase and ligase. putative
67024	10.50	0.16	10.01	0.03	12.38	0.24	1.411 down	3.681 up	0.00	112.75	unknown protein
67035	13.88	0.12	13.30	0.10	11.64	0.09	1.492 down	4.701 down	0.00	407.39	unknown protein
67053	12.27	0.05	12.09	0.05	11.17	0.25	1.132 down	2.145 down	0.01	27.72	Ubiquitin carboxyl-terminal hydrolase
67107	9.06	0.07	10.41	0.07	10.76	0.19	2.535 up	3.231 up	0.00	80.76	MutS-related protein involved in mismatch repair

67109	6.13	0.07	5.76	0.06	7.93	0.72	1.288 down	3.491 up	0.02	12.73	unknown protein
67133	11.34	0.20	10.12	0.26	9.95	0.40	2.323 down	2.620 down	0.02	11.39	unknown protein
67272	12.44	0.05	11.86	0.14	11.11	0.04	1.492 down	2.516 down	0.00	233.49	CRO1. required for syncytial to cellular transision. involved in sexual development
67275	11.28	0.10	12.61	0.19	12.44	0.16	2.518 up	2.241 up	0.00	46.36	RAS1
67286	10.78	0.11	10.42	0.11	9.04	0.22	1.279 down	3.345 down	0.00	72.79	unknown protein
67290	5.52	0.19	5.82	0.01	7.35	0.10	1.231 up	3.539 up	0.00	207.56	unknown protein
67418	8.16	0.04	8.64	0.23	9.75	0.14	1.399 up	3.015 up	0.00	83.32	C2H2 transcription factor
67448	10.50	0.08	10.45	0.01	8.80	0.08	1.030 down	3.245 down	0.00	529.60	AGA_beta. Aspartylglucosaminidase family
67469	13.08	0.04	11.41	0.12	11.48	0.10	3.172 down	3.022 down	0.00	221.51	MFS permease (maltose permease)
67494	14.04	0.10	13.38	0.23	11.73	0.28	1.575 down	4.957 down	0.00	70.17	Golgi GDP-mannose transporter
67538	11.44	0.03	6.73	0.08	8.64	0.24	26.140 down	6.954 down	0.00	303.94	Catalase
67579	9.78	0.27	8.67	0.02	11.32	0.96	2.166 down	2.901 up	0.03	8.79	phospholipase A2
67600	2.17	0.16	2.32	0.14	4.02	0.04	1.108 up	3.619 up	0.00	323.80	Fungal chitin synthase
67605	8.90	0.02	9.11	0.04	7.09	0.15	1.152 up	3.525 down	0.00	274.34	unknown protein
67607	4.49	0.06	4.71	0.22	6.80	0.65	1.160 up	4.956 up	0.01	18.67	unknown protein
67616	12.26	0.03	11.55	0.19	11.15	0.16	1.632 down	2.155 down	0.00	36.56	unknown protein
67627	3.25	0.03	3.30	0.06	4.66	0.09	1.034 up	2.664 up	0.00	358.06	short chain dehydrognease/reductase
67639	13.74	0.06	13.69	0.01	12.57	0.13	1.038 down	2.249 down	0.00	118.16	IlvB. Thiamine pyrophosphate-requiring enzymes
67678	4.34	0.06	4.61	0.02	6.49	0.59	1.211 up	4.457 up	0.01	19.30	chitin deacetylase
67692	7.05	0.49	5.84	0.22	5.93	0.15	2.318 down	2.175 down	0.02	13.90	MFS permease
67698	4.30	0.13	4.83	0.29	5.55	0.19	1.450 up	2.387 up	0.01	26.61	nucleotide sugar dehydrogenase (UDP-Glc?)
67699	10.94	0.14	7.00	0.09	9.04	0.37	15.411 down	3.753 down	0.00	88.88	FAD/NAD-oxidoreductase. only in Hypocreaceae
67707	4.05	0.07	4.47	0.37	5.58	0.12	1.340 up	2.879 up	0.00	48.81	unknown protein with TLC domain
67717	11.97	0.07	11.28	0.16	10.15	0.37	1.614 down	3.520 down	0.01	27.52	unknown protein. ß-lactamase HMMPfam
67761	9.80	0.19	11.09	0.19	11.77	0.26	2.447 up	3.908 up	0.00	45.72	calpain-like protease
67866	6.71	0.24	4.75	0.10	5.42	0.29	3.890 down	2.442 down	0.00	31.59	unknown protein
67958	3.71	0.22	3.50	0.33	4.90	0.19	1.153 down	2.276 up	0.00	31.06	unknown protein. only present in Magnaporthe. Chaetomium and Gibberella
67964	13.24	0.10	12.12	0.01	11.94	0.12	2.182 down	2.462 down	0.00	115.86	Cytochrome P450 CYP2 subfamily
68019	9.98	0.24	8.75	0.04	7.87	0.10	2.337 down	4.319 down	0.00	164.18	beta-lactamase superfamily
68028	8.52	0.05	8.46	0.09	7.29	0.27	1.039 down	2.340 down	0.00	32.22	unknown protein

68036	13.49	0.04	12.84	0.11	12.27	0.23	1.572 down	2.329 down	0.00	30.28	Cystathionine beta-lyases/cystathionine gamma-synthases
68068	11.60	0.10	11.38	0.00	10.53	0.27	1.157 down	2.086 down	0.01	20.00	unknown protein
68086	11.08	0.08	11.15	0.01	9.31	0.27	1.050 up	3.395 down	0.00	73.54	unknown protein
68131	8.23	0.09	7.75	0.18	6.87	0.33	1.392 down	2.566 down	0.01	18.66	heterocompatibility domain protein
68161	8.39	0.05	7.20	0.11	6.22	0.14	2.279 down	4.504 down	0.00	230.11	Arylacetamide deacetylase
68169	8.21	0.14	6.43	0.03	6.86	0.32	3.450 down	2.553 down	0.01	27.93	Calcium transporter
68204	8.67	0.13	8.95	0.01	6.92	0.25	1.209 up	3.375 down	0.00	89.00	NRPS
68279	10.54	0.02	8.52	0.10	8.02	0.26	4.046 down	5.749 down	0.00	101.46	Short-chain dehydrogenase/reductase
68336	4.58	0.03	4.81	0.18	5.90	0.10	1.171 up	2.489 up	0.00	114.61	short chain dehydrogenase/reductase
68348	8.57	0.03	10.86	0.20	12.10	0.13	4.893 up	11.521 up	0.00	466.73	SAM-dependent methyltransferase
68371	5.35	0.33	5.16	0.11	7.60	0.30	1.138 down	4.760 up	0.00	71.66	protein kinase. unusual
68401	3.11	0.06	3.37	0.01	4.86	0.10	1.199 up	3.353 up	0.00	396.73	unknown protein
68455	10.90	0.08	10.71	0.02	9.64	0.11	1.145 down	2.398 down	0.00	145.45	Zn2Cys6 transcriptional regulator
68466	4.51	0.05	4.42	0.05	5.76	0.20	1.060 down	2.389 up	0.00	66.70	glycerone kinase
68508	9.81	0.16	9.83	0.09	8.40	0.21	1.011 up	2.661 down	0.00	60.28	unknown protein
68574	9.77	0.27	6.82	0.02	8.03	0.37	7.717 down	3.343 down	0.00	45.98	unknown protein
68590	10.65	0.02	9.87	0.06	9.28	0.22	1.715 down	2.584 down	0.00	41.55	Alcohol dehydrogenase. class V
68608	14.73	0.13	14.40	0.00	13.41	0.20	1.260 down	2.507 down	0.00	50.55	Thiazole biosynthetic enzyme (Stress-inducible protein sti35)
68636	10.94	0.25	11.38	0.17	12.13	0.22	1.349 up	2.275 up	0.01	22.75	ribosomal protein S11
68640	7.38	0.20	7.79	0.10	8.43	0.40	1.331 up	2.079 up	0.04	7.47	unknown protein
68705	12.22	0.04	12.33	0.05	7.10	0.64	1.079 up	34.693 down	0.00	107.87	cytochrome P450 monooxygenase
68755	12.56	0.08	10.20	0.13	9.23	0.25	5.158 down	10.057 down	0.00	174.06	short chain dehydrogenase/reductase
68813	13.41	0.05	11.72	0.01	11.85	0.55	3.217 down	2.939 down	0.02	10.71	MFS permease
68889	11.28	0.07	10.37	0.11	9.31	0.16	1.881 down	3.905 down	0.00	143.70	PDR-type ABC transporters
68924	9.09	0.07	12.02	0.03	13.14	1.18	7.616 up	16.584 up	0.02	13.09	cyclopropane/fatty acid synthase; plant related
68925	8.24	0.13	8.16	0.01	6.94	0.08	1.051 down	2.454 down	0.00	215.72	MFS permease
68927	10.73	0.07	10.82	0.05	9.55	0.18	1.069 up	2.266 down	0.00	75.12	unknown protein
68966	9.54	0.01	9.50	0.12	8.50	0.47	1.034 down	2.063 down	0.04	7.67	flavoprotein. putative
68990	13.19	0.08	13.51	0.08	11.36	0.96	1.242 up	3.578 down	0.05	7.29	MFS permease
69026	9.36	0.19	9.05	0.16	8.30	0.38	1.237 down	2.080 down	0.04	8.71	MFS permease

69131	11.42	0.05	11.28	0.09	10.41	0.14	1.105 down	2.018 down	0.00	61.91	Unknown protein with FYVE/PHD zinc finger domain.
69171	10.08	0.14	9.29	0.10	11.73	0.46	1.728 down	3.127 up	0.00	33.51	unknown protein
69181	2.95	0.10	2.78	0.21	4.78	0.13	1.129 down	3.553 up	0.00	176.34	unknown protein
69210	3.36	0.17	3.32	0.13	5.14	0.21	1.031 down	3.440 up	0.00	92.13	FoNIIA
69228	13.23	0.02	13.10	0.11	11.83	0.14	1.094 down	2.637 down	0.00	119.53	BioA Adenosylmethionine--amino--oxononanoate aminotransferase
69245	11.63	0.00	9.06	0.20	10.44	0.21	5.937 down	2.282 down	0.00	97.99	GH2 β-mannosidase
69257	8.19	0.05	8.62	0.00	9.40	0.39	1.344 up	2.311 up	0.02	11.97	Peptidase M22. glycoprotease
69276	7.51	0.17	5.98	0.09	11.81	0.72	2.896 down	19.619 up	0.00	84.77	GH30 endo-β-1.4-xylanase
69282	10.89	0.01	9.07	0.10	8.79	0.21	3.538 down	4.297 down	0.00	109.56	monocarboxylate transporter
69287	12.44	0.09	12.84	0.12	13.83	0.13	1.318 up	2.617 up	0.00	104.55	unknown protein
69291	3.15	0.18	3.27	0.04	4.64	0.29	1.081 up	2.804 up	0.00	36.18	FoNIIA
69303	7.40	0.01	10.33	0.01	9.60	0.39	7.661 up	4.594 up	0.00	54.45	unknown protein
69362	11.97	0.06	11.86	0.19	10.34	0.30	1.078 down	3.080 down	0.00	39.09	phospholipase A2 protein family
69375	9.24	0.17	7.03	0.04	8.18	0.53	4.623 down	2.086 down	0.02	14.06	unknown protein
69384	11.42	0.00	10.75	0.01	10.04	0.17	1.593 down	2.602 down	0.00	78.94	unknown protein
69399	11.51	0.09	12.09	0.11	12.83	0.02	1.500 up	2.501 up	0.00	283.31	mitochondrial 40S ribosomal protein [Aspergillus niger]. Possible homologue of yeast
69416	11.65	0.12	11.70	0.08	10.48	0.15	1.030 up	2.247 down	0.00	85.66	unknown protein
69426	8.97	0.18	7.66	0.15	7.56	0.44	2.487 down	2.651 down	0.02	11.05	copper transporter
69479	8.65	0.09	8.88	0.02	7.57	0.30	1.180 up	2.111 down	0.01	26.18	unknown protein
69486	3.20	0.07	3.44	0.03	5.14	0.28	1.179 up	3.826 up	0.00	70.12	unknown protein
69490	4.21	0.14	4.15	0.06	6.08	0.38	1.036 down	3.654 up	0.00	40.07	CE4 chitin deacetylase
69528	8.24	0.15	9.90	0.11	9.57	0.12	3.159 up	2.513 up	0.00	107.18	unknown protein
69555	4.41	0.28	4.56	0.22	6.31	0.25	1.111 up	3.726 up	0.00	51.60	aspartyl protease. Aspergillopepsin-like
69569	4.22	0.52	5.41	0.03	5.48	0.19	2.283 up	2.392 up	0.01	15.15	unknown protein
69574	13.48	0.05	13.03	0.03	10.72	0.21	1.370 down	6.767 down	0.00	234.91	MFS permease
69648	3.99	0.43	3.45	0.17	6.15	0.54	1.449 down	4.473 up	0.01	28.16	Cytochrome P450 CYP11/CYP12/CYP24/CYP27 subfamilies
69679	3.34	0.03	3.47	0.16	5.00	0.12	1.091 up	3.165 up	0.00	183.81	ABC transporter
69688	3.08	0.14	3.24	0.10	5.09	0.17	1.121 up	4.039 up	0.00	158.03	unknown protein
69692	4.44	0.14	5.08	0.25	8.85	0.75	1.557 up	21.254 up	0.00	47.42	Quinoprotein amine dehydrogenase beta chain-like protein
69695	10.00	0.03	9.83	0.08	6.78	0.46	1.123 down	9.330 down	0.00	76.87	Zn2Cys6 transcriptional regulator

69696	5.14	0.24	5.11	0.06	7.17	0.59	1.023 down	4.071 up	0.01	19.07	coenzyme F420-dependent N5.N10-methylene tetrahydromethanopterin reductase
69712	3.16	0.15	2.75	0.29	4.24	0.14	1.328 down	2.110 up	0.00	52.12	MFS permease
69742	5.51	0.21	6.65	0.16	8.54	0.28	2.206 up	8.201 up	0.00	110.41	esterase
69751	8.28	0.00	9.16	0.14	6.72	0.59	1.838 up	2.952 down	0.01	20.92	GH16
69757	9.89	0.04	10.62	0.08	10.94	0.17	1.663 up	2.068 up	0.00	37.27	Peptidyl-tRNA hydrolase
69777	7.40	0.29	8.26	0.13	9.20	0.14	1.812 up	3.486 up	0.00	70.44	unknown protein
69805	3.91	0.04	3.84	0.12	5.16	0.13	1.046 down	2.384 up	0.00	119.57	unknown protein
69811	8.80	0.19	10.17	0.09	5.90	0.28	2.584 up	7.460 down	0.00	246.69	γ-glutamyltransferase
69816	7.98	0.01	8.20	0.02	9.29	0.11	1.168 up	2.477 up	0.00	197.99	aminoglycoside 3-N-acetyltransferase
69825	8.08	0.29	9.31	0.02	9.28	0.24	2.347 up	2.300 up	0.01	21.11	unknown protein
69841	11.97	0.06	11.96	0.01	10.38	0.22	1.006 down	3.008 down	0.00	84.58	unknown protein
69857	7.36	0.00	7.10	0.13	8.41	0.47	1.198 down	2.075 up	0.02	10.71	unknown protein
69868	5.58	0.09	6.15	0.26	7.98	0.42	1.493 up	5.306 up	0.00	39.03	GDP-mannose a-mannosyltransferases;Distant relative
69870	3.28	0.09	3.36	0.05	4.97	0.25	1.058 up	3.223 up	0.00	66.76	FAD monooxygenase
69879	10.38	0.01	11.22	0.15	11.45	0.17	1.796 up	2.098 up	0.00	36.70	unknown protein
69881	3.36	0.11	3.48	0.03	4.73	0.22	1.086 up	2.584 up	0.00	53.35	Dit1p. spore wall maturation
69883	4.12	0.33	3.48	0.40	5.28	0.21	1.557 down	2.245 up	0.00	29.84	cytochrome P450. putative
69885	10.40	0.04	8.15	0.07	9.31	0.45	4.762 down	2.134 down	0.01	20.83	initiation factor 2B
69896	3.00	0.17	2.72	0.27	4.97	0.14	1.209 down	3.938 up	0.00	143.98	alpha/beta hydrolase
69901	3.40	0.07	3.02	0.01	4.60	0.03	1.300 down	2.309 up	0.00	1125.08	unknown protein
69920	3.63	0.16	3.71	0.01	4.93	0.12	1.058 up	2.467 up	0.00	114.94	unknown protein
69928	3.47	0.08	3.63	0.13	5.25	0.11	1.113 up	3.432 up	0.00	249.27	unknown protein
69933	10.15	0.10	10.40	0.06	8.71	0.15	1.185 up	2.711 down	0.00	152.19	unknown protein
69944	6.24	0.24	4.51	0.30	7.87	0.21	3.310 down	3.091 up	0.00	136.78	GH31 α-xylosidase/α-glucosidase
69946	11.99	0.07	10.08	0.12	10.39	0.48	3.780 down	3.042 down	0.01	15.77	NRPS. siderophore synthase
69950	4.41	0.06	4.82	0.02	5.81	0.19	1.329 up	2.635 up	0.00	67.30	unknown protein
69956	8.78	0.15	5.90	0.28	7.49	0.55	7.346 down	2.442 down	0.01	20.76	Alcohol dehydrogenase. class V
69972	11.66	0.18	11.21	0.18	9.45	0.16	1.364 down	4.630 down	0.00	144.76	Zn2Cys6 transcriptional regulator
70017	10.40	0.17	10.91	0.16	12.85	0.40	1.423 up	5.487 up	0.00	46.97	mitochondrial ribosomal protein S19/S15.
70021	6.18	0.02	5.85	0.05	7.25	0.23	1.259 down	2.096 up	0.00	47.30	CE3 acetyl xylan Esterase

70025	13.16	0.04	12.65	0.04	10.78	0.19	1.429 down	5.203 down	0.00	196.82	alcohol dehydrogenase. zinc-containing. putative
70092	9.13	0.40	8.33	0.30	7.73	0.19	1.736 down	2.636 down	0.01	18.18	unknown protein
70096	9.51	0.03	10.55	0.23	10.76	0.16	2.064 up	2.380 up	0.00	40.88	Gß-WD40 domain protein
70098	11.17	0.01	10.10	0.15	7.75	0.36	2.102 down	10.727 down	0.00	109.06	amino acid permease (GABA)
70108	9.23	0.07	8.85	0.11	7.01	0.25	1.302 down	4.649 down	0.00	101.37	MFS permease
70186	5.06	0.23	4.57	0.29	6.72	0.49	1.405 down	3.155 up	0.01	21.99	GH28 polygalacturonase/xylogalacturonan hydrolase
70195	3.38	0.05	3.36	0.12	4.52	0.09	1.016 down	2.210 up	0.00	158.57	unknown protein
70197	11.15	0.01	5.60	0.18	9.84	0.19	46.788 down	2.467 down	0.00	617.37	Zn-dependent ß-lactamase
70327	5.46	0.61	5.91	0.13	7.14	0.39	1.369 up	3.210 up	0.02	13.23	unknown protein
70334	10.11	0.04	8.65	0.30	6.98	0.81	2.761 down	8.772 down	0.01	16.47	short chain dehydrogenase/reductase
70339	4.26	0.23	4.10	0.10	5.40	0.24	1.120 down	2.200 up	0.00	31.06	cytochrome P450 monoounknown proteingenase (trichothecene C-15 hydrounknown
70349	12.45	0.04	10.46	0.05	9.34	0.36	3.978 down	8.651 down	0.00	80.55	MFS permease LIZ1
70355	13.53	0.04	10.95	0.08	9.32	0.37	5.994 down	18.605 down	0.00	139.38	SAM-dependent methylransferase
70365	10.54	0.08	6.53	0.12	7.16	0.48	16.042 down	10.414 down	0.00	69.55	unknown protein
70373	12.92	0.17	12.05	0.00	10.33	0.27	1.837 down	6.019 down	0.00	98.16	unknown protein
70375	11.87	0.01	10.13	0.13	8.22	0.38	3.347 down	12.561 down	0.00	101.32	amidase
70383	11.28	0.18	10.42	0.10	9.02	0.36	1.814 down	4.805 down	0.00	43.16	succinate semialdehyde dehydrogenase. NADP
70400	4.57	0.27	4.75	0.12	6.19	0.53	1.135 up	3.069 up	0.02	12.68	unknown protein
70414	9.68	0.10	9.46	0.11	7.93	0.31	1.160 down	3.363 down	0.00	44.28	Zn2Cys6 transcriptional regulator
70439	10.54	0.06	11.54	0.13	11.87	0.25	1.997 up	2.507 up	0.00	29.12	NADH-ubiquinone oxidoreductase
70491	11.78	0.16	5.64	0.54	10.47	0.08	70.700 down	2.492 down	0.00	329.23	esterase/lipase
70570	9.59	0.10	9.65	0.07	7.89	0.22	1.047 up	3.243 down	0.00	91.82	Zn2Cys6 transcriptional regulator
70599	4.79	0.01	5.12	0.07	6.19	0.19	1.258 up	2.636 up	0.00	68.71	unknown protein
70600	7.64	0.14	3.51	0.13	4.67	0.08	17.584 down	7.834 down	0.00	814.33	catalase. large subunit type
70608	12.03	0.00	7.00	0.03	8.32	0.65	32.737 down	13.100 down	0.00	55.95	unknown protein. HHE domains
70630	13.50	0.01	13.46	0.07	12.46	0.06	1.033 down	2.060 down	0.00	358.12	homoserine acetyltransferase family protein
70631	5.02	0.30	4.79	0.05	6.21	0.19	1.175 down	2.285 up	0.00	44.04	dipeptidyl peptidase 5
70803	9.92	0.09	8.92	0.11	12.17	0.09	2.003 down	4.754 up	0.00	896.65	bifunctional catalase/peroxidase
70829	10.01	0.06	9.05	0.06	7.40	0.33	1.944 down	6.140 down	0.00	75.50	MFS multidrug transporter. putative
70830	9.31	0.01	9.67	0.10	8.10	0.20	1.280 up	2.310 down	0.00	73.93	MFS permease

70837	3.91	0.09	4.52	0.26	4.97	0.24	1.519 up	2.084 up	0.01	14.75	E3 ubiquitin ligase
70842	9.42	0.32	8.29	0.00	7.04	0.22	2.181 down	5.192 down	0.00	79.52	Cytochrome P450 CYP2 subfamily
70845	9.04	0.11	10.70	0.06	11.59	0.16	3.171 up	5.855 up	0.00	225.01	GH55 β-1.3-glucanase
70855	7.71	0.04	8.41	0.05	8.75	0.17	1.629 up	2.052 up	0.00	38.21	unknown protein
70859	12.75	0.14	10.64	0.08	10.34	0.27	4.320 down	5.344 down	0.00	82.25	amidase
70894	12.76	0.12	10.28	0.11	11.00	0.19	5.560 down	3.395 down	0.00	124.59	unknown protein
70907	10.29	0.16	12.14	0.05	12.50	0.15	3.594 up	4.621 up	0.00	169.89	Ankyrin
70910	11.55	0.25	10.54	0.12	8.04	0.39	2.008 down	11.340 down	0.00	89.78	GMC oxidoreductase
70918	4.01	0.13	3.94	0.15	7.04	0.63	1.053 down	8.118 up	0.00	37.99	unknown protein
70919	5.81	0.07	7.64	0.25	7.57	0.16	3.550 up	3.373 up	0.00	83.83	SSCRP
70921	5.99	0.14	5.56	0.06	7.92	0.40	1.347 down	3.785 up	0.00	46.34	unknown protein
70923	8.68	0.00	9.31	0.13	7.26	0.09	1.543 up	2.682 down	0.00	367.09	unknown protein. only in fungi
70927	3.16	0.10	3.31	0.00	4.89	0.14	1.116 up	3.319 up	0.00	203.65	unknown protein
70932	10.28	0.11	9.93	0.01	9.24	0.29	1.280 down	2.064 down	0.01	15.03	MFS permease
70943	10.48	0.05	10.30	0.00	9.47	0.31	1.137 down	2.022 down	0.01	14.90	Histidine kinase. part of a two component signal transduction system
70956	7.25	0.06	5.04	0.39	5.53	0.28	4.615 down	3.284 down	0.00	35.89	cytochrome P450 monooxygenase
70961	10.69	0.08	9.65	0.07	9.32	0.42	2.050 down	2.579 down	0.02	11.57	dienelactone hydrolase
70962	2.90	0.14	2.90	0.04	4.90	0.03	1.001 up	4.010 up	0.00	782.85	tripeptide peptidase
70973	3.03	0.24	2.84	0.08	4.03	0.17	1.142 down	2.000 up	0.00	39.81	4-aminobutyrate aminotransferase
70991	5.83	0.11	7.99	0.09	7.65	0.31	4.481 up	3.539 up	0.00	46.14	unknown protein. ThiJ/PfpI domain
70996	6.37	0.24	6.68	0.16	7.52	0.25	1.238 up	2.219 up	0.01	19.64	SSCRP
71010	7.98	0.08	6.89	0.16	5.82	0.14	2.126 down	4.473 down	0.00	173.68	MDR-type ABC transporters
71029	10.94	0.06	12.79	0.26	14.14	0.26	3.606 up	9.178 up	0.00	128.82	Ctr copper transporter. putative
71059	9.70	0.14	9.20	0.12	8.55	0.45	1.410 down	2.224 down	0.05	7.42	MFS permease
71072	7.07	0.36	4.76	0.11	5.14	0.20	4.958 down	3.818 down	0.00	61.33	Gluconate kinase
71076	2.98	0.31	3.11	0.04	4.72	0.24	1.093 up	3.337 up	0.00	52.85	estrase/lipase
71078	2.56	0.14	2.61	0.07	4.17	0.10	1.038 up	3.064 up	0.00	227.56	Ca/CaM-dependent kinase-1
71080	9.42	0.16	9.01	0.04	7.51	0.16	1.325 down	3.746 down	0.00	140.87	Zn2Cys6 transcriptional regulator
71092	10.34	0.02	11.78	0.18	12.80	0.22	2.724 up	5.525 up	0.00	118.89	phytase
71094	8.58	0.08	10.76	0.04	7.22	0.39	4.531 up	2.569 down	0.00	92.03	Intradiol ring-cleavage dioxygenase

71095	4.58	0.09	4.96	0.07	6.55	0.29	1.300 up	3.922 up	0.00	60.55	Aldehyde dehydrogenase
71101	5.37	0.40	5.38	0.17	7.84	0.29	1.010 up	5.575 up	0.00	67.98	enoyl-CoA hydratase/isomerase
71103	2.80	0.00	3.16	0.09	4.46	0.07	1.281 up	3.159 up	0.00	490.87	dynamin GTPase. putative
71123	7.27	0.05	8.13	0.00	10.42	0.33	1.810 up	8.852 up	0.00	121.11	unknown protein
71125	3.77	0.12	3.85	0.14	5.65	0.38	1.057 up	3.678 up	0.00	35.72	unknown protein
71146	3.35	0.04	3.51	0.60	4.94	0.38	1.114 up	3.012 up	0.02	14.48	unknown protein
71154	5.27	0.47	5.49	0.21	6.53	0.29	1.158 up	2.392 up	0.02	13.04	unknown protein
71166	3.94	0.40	4.03	0.21	6.27	0.28	1.061 up	5.009 up	0.00	59.23	unknown protein
71167	2.85	0.42	2.59	0.09	4.56	0.14	1.202 down	3.268 up	0.00	68.95	SSCRP
71173	3.53	0.14	4.15	0.04	5.26	0.12	1.536 up	3.313 up	0.00	175.88	unknown protein
71180	6.22	0.05	6.74	0.00	8.02	0.44	1.436 up	3.483 up	0.01	21.50	unknown protein
71192	3.67	0.54	3.69	0.17	6.39	0.51	1.010 up	6.576 up	0.00	32.88	unknown protein
71196	5.10	0.16	5.49	0.01	6.57	0.44	1.308 up	2.759 up	0.02	14.03	unknown protein
71212	5.47	0.23	5.54	0.23	7.16	0.34	1.050 up	3.241 up	0.00	30.06	unique protein
71245	4.32	0.20	4.74	0.04	5.50	0.43	1.341 up	2.270 up	0.04	8.63	GH18. chitinase CHI18-1
71390	5.86	0.08	5.94	0.22	7.03	0.35	1.059 up	2.255 up	0.01	15.50	MRSP1/expansin-like
71399	12.72	0.02	11.50	0.01	11.62	0.11	2.328 down	2.149 down	0.00	134.28	endo-1.3-β-glucanase
71532	12.29	0.03	8.50	0.08	10.57	0.09	13.909 down	3.294 down	0.00	1106.21	GH71 α-1 3-glucanase
71554	3.71	0.25	3.59	0.13	5.35	0.12	1.084 down	3.111 up	0.00	115.03	GH5 β-1.3-mannanase/endo-β-1.4-mannosidase
71556	7.46	0.10	5.79	0.05	5.63	0.14	3.190 down	3.546 down	0.00	168.22	unknown protein
72071	5.70	0.37	5.95	0.16	7.48	0.62	1.184 up	3.438 up	0.02	10.72	Carbohydrate-Binding Module Family 13
72072	9.59	0.17	7.82	0.04	8.56	0.28	3.402 down	2.039 down	0.00	29.57	CE1 esterase (PHB?)
72183	8.58	0.17	10.25	0.13	7.50	0.15	3.161 up	2.115 down	0.00	228.09	SSCRP
72488	9.32	0.14	9.94	0.15	8.07	0.32	1.527 up	2.387 down	0.00	37.87	GH95 α-L-fucosidase
72567	12.99	0.15	9.03	0.15	15.25	0.15	15.565 down	4.797 up	0.00	1107.20	GH6 Cellobiohydrolase CEL6A/CBH2
72581	8.82	0.34	7.71	0.09	11.40	0.21	2.158 down	5.960 up	0.00	201.13	unknown protein
72602	12.02	0.21	11.09	0.08	10.62	0.05	1.905 down	2.632 down	0.00	111.61	ribosomal protein P2.
72605	4.31	0.34	4.06	0.18	5.70	0.25	1.190 down	2.628 up	0.00	35.55	GPCR. secretin like
72611	13.77	0.12	13.30	0.08	11.31	0.40	1.386 down	5.511 down	0.00	50.81	Zn2Cys6 transcriptional regulator
72612	11.50	0.06	10.43	0.12	9.70	0.26	2.100 down	3.496 down	0.00	50.55	acetylornithine deacetylase

72627	9.44	0.11	9.83	0.07	7.01	0.32	1.309 up	5.418 down	0.00	109.17	GPCR. secretin like
72632	11.70	0.02	9.17	0.11	8.11	0.54	5.777 down	12.000 down	0.00	48.17	GH27 α-galactosidase AGL1
72896	12.26	0.03	12.61	0.14	13.46	0.09	1.277 up	2.294 up	0.00	122.74	Dolichol-phosphate (beta-D) mannosyltransferase 2
72907	7.94	0.00	7.83	0.16	6.34	0.42	1.083 down	3.047 down	0.01	21.46	unknown protein with WSC domain
72922	12.41	0.00	8.98	0.13	9.52	0.07	10.758 down	7.407 down	0.00	1110.16	amino acid permease (GABA)
73005	2.66	0.02	2.75	0.00	4.49	0.09	1.061 up	3.560 up	0.00	637.33	GH79 β-glucuronidase
73016	3.02	0.01	3.20	0.05	5.41	0.13	1.132 up	5.225 up	0.00	508.13	aryl-alcohol oxidase
73023	3.43	0.02	3.45	0.15	4.99	0.16	1.013 up	2.949 up	0.00	116.79	unknown protein
73024	4.22	0.35	4.97	0.05	6.61	0.66	1.673 up	5.217 up	0.01	15.20	unknown protein
73039	7.30	0.29	6.89	0.19	5.33	0.23	1.333 down	3.926 down	0.00	58.14	APHPhosphotransferase enzyme family aligned
73048	13.24	0.10	14.22	0.14	14.75	0.06	1.975 up	2.846 up	0.00	187.40	unknown protein
73101	4.81	0.12	4.67	0.19	6.29	0.12	1.099 down	2.796 up	0.00	131.44	glucan endo-1.3-1.4-β-D-glucosidase
73102	7.51	0.03	6.53	0.05	6.11	0.21	1.966 down	2.636 down	0.00	48.61	GH39 β-xylosidase
73103	2.42	0.18	2.56	0.21	3.90	0.03	1.100 up	2.797 up	0.00	126.63	ribosomal protein P2.
73104	10.50	0.04	9.84	0.03	9.10	0.18	1.576 down	2.634 down	0.00	67.42	Zn2Cys6 transcriptional regulator
73119	2.75	0.11	2.92	0.24	4.63	0.10	1.119 up	3.672 up	0.00	172.00	unknown protein
73173	7.12	0.07	7.70	0.24	8.87	0.55	1.500 up	3.381 up	0.02	12.09	HFB1
73179	6.99	0.07	8.66	0.06	8.78	0.43	3.182 up	3.464 up	0.01	20.78	GH71 α-1 3-glucanase
73248	7.96	0.25	6.78	0.13	6.68	0.23	2.266 down	2.413 down	0.01	24.69	GH55 exo-1.3-β-glucanase
73250	3.38	0.32	3.07	0.09	6.51	1.36	1.239 down	8.723 up	0.03	9.56	Urea transporter
73344	3.24	0.00	3.42	0.12	5.06	0.19	1.136 up	3.532 up	0.00	116.51	Cytochrome P450 CYP3/CYP5/CYP6/CYP9 subfamilies
73417	13.29	0.04	12.77	0.06	12.07	0.11	1.432 down	2.331 down	0.00	137.53	bZIP transcriptional regulator MeaB
73516	14.59	0.04	13.92	0.08	13.56	0.34	1.590 down	2.049 down	0.03	10.24	Grg1 Glucose repressible protein
73536	9.46	0.12	9.48	0.15	12.80	0.11	1.012 up	10.116 up	0.00	782.96	NADP-glutamate dehydrogenase
73594	10.58	0.11	11.36	0.23	11.80	0.16	1.712 up	2.329 up	0.00	33.93	unknown protein
73623	4.63	0.01	4.23	0.24	5.99	0.21	1.317 down	2.574 up	0.00	65.62	flavoprotein monooxygenase
73632	7.19	0.14	4.42	0.10	11.10	0.28	6.821 down	15.028 up	0.00	591.15	CE5 acetyl xylan esterase AXE1
73638	9.01	0.17	4.94	0.12	13.63	0.38	16.794 down	24.546 up	0.00	555.34	CIP1
73643	7.81	0.10	3.13	0.22	12.33	0.16	25.659 down	22.910 up	0.00	2218.87	GH61 polysaccharide monooxygenase CEL61a
73783	9.81	0.18	9.85	0.04	11.27	0.15	1.032 up	2.759 up	0.00	100.93	mitochondrial co-chaperone GrpE . putative

73792	11.63	0.02	11.83	0.06	10.43	0.05	1.145 up	2.298 down	0.00	809.35	Zn2Cys6 transcriptional regulator
73815	13.99	0.17	13.46	0.07	12.91	0.18	1.436 down	2.114 down	0.00	31.96	unknown protein
73904	9.14	0.15	11.26	0.08	10.92	0.13	4.345 up	3.426 up	0.00	181.24	unknown protein
73912	8.48	0.11	9.00	0.04	9.66	0.20	1.438 up	2.263 up	0.00	37.75	unknown protein
73924	11.14	0.11	12.47	0.07	12.48	0.09	2.517 up	2.535 up	0.00	173.36	MDR-type ABC transporters
74017	11.41	0.02	11.14	0.08	10.11	0.09	1.207 down	2.458 down	0.00	235.41	unknown protein
74054	12.28	0.03	12.31	0.04	13.38	0.13	1.020 up	2.139 up	0.00	111.43	Exocyst component Sec6
74060	7.87	1.07	8.74	0.11	11.21	0.26	1.822 up	10.096 up	0.00	32.26	SSCRP
74162	12.52	0.00	12.17	0.06	11.39	0.12	1.277 down	2.200 down	0.00	107.12	Helicase. C-terminal
74194	13.02	0.12	11.13	0.28	11.40	0.27	3.702 down	3.064 down	0.00	35.73	mannitol dehydrogenase LXR1
74214	9.95	0.20	8.46	0.00	8.51	0.27	2.813 down	2.706 down	0.00	30.94	unknown protein
74215	6.96	0.15	7.80	0.03	5.73	0.29	1.792 up	2.347 down	0.00	55.58	RTA1-like protein. 7 TM. responds to xenobiotic stimuli
74228	11.70	0.13	13.10	0.00	13.21	0.23	2.632 up	2.849 up	0.00	47.01	oleate δ-12 desaturase
74278	12.26	0.12	10.73	0.16	11.08	0.24	2.893 down	2.268 down	0.00	31.14	Pyridine nucleotide-disulphide oxidoreductase. class-II
74379	8.43	0.09	8.66	0.04	9.46	0.13	1.175 up	2.035 up	0.00	67.31	ERG2 C-8 sterol isomerase; catalyzes the isomerization of the delta-8 double bond to t
74421	10.38	0.00	10.60	0.12	11.94	0.35	1.164 up	2.954 up	0.00	28.66	unknown protein
74476	11.49	0.06	12.09	0.07	10.18	0.21	1.512 up	2.470 down	0.00	97.69	unknown protein
74486	10.91	0.02	11.51	0.02	12.03	0.13	1.513 up	2.165 up	0.00	82.02	unknown protein
74502	13.25	0.12	12.63	0.04	12.24	0.04	1.539 down	2.020 down	0.00	172.70	Aldehyde dehydrogenase
74515	13.53	0.07	12.21	0.05	12.24	0.31	2.508 down	2.449 down	0.01	21.27	unknown protein
74563	11.02	0.03	10.93	0.03	9.25	0.18	1.067 down	3.411 down	0.00	153.07	unknown protein
74576	13.56	0.11	13.52	0.07	12.22	0.03	1.033 down	2.544 down	0.00	407.74	BZIP transcriptional regulator
74622	11.27	0.03	11.37	0.08	12.49	0.20	1.068 up	2.317 up	0.00	51.00	unknown protein
74701	10.65	0.17	10.59	0.05	9.43	0.25	1.043 down	2.336 down	0.00	32.00	GTP-binding protein involved in protein synthesis
74861	9.15	0.03	9.15	0.05	7.77	0.08	1.000 up	2.614 down	0.00	457.84	unknown protein
74943	9.15	0.05	9.97	0.04	10.62	0.14	1.760 up	2.763 up	0.00	110.40	Diphthine synthase
74971	12.24	0.05	12.23	0.07	11.16	0.33	1.002 down	2.104 down	0.01	16.93	unknown protein
75027	13.85	0.10	12.58	0.05	12.52	0.07	2.399 down	2.514 down	0.00	218.64	unknown protein
75036	12.99	0.08	12.29	0.13	11.32	0.32	1.618 down	3.168 down	0.00	30.70	GH63 α-glucosidase
75156	4.13	0.04	3.53	0.17	5.94	0.19	1.519 down	3.506 up	0.00	162.97	unknown protein

75247	4.67	0.02	4.44	0.26	6.06	0.17	1.176 down	2.611 up	0.00	73.54	AAA+-type ATPase
75260	11.33	0.02	10.36	0.05	10.03	0.09	1.954 down	2.455 down	0.00	208.69	RNA polymerase H/23 kD subunit
75271	9.55	0.06	9.16	0.19	11.33	0.33	1.311 down	3.421 up	0.00	54.50	pyridoxine biosynthesis protein PDX1
75290	7.46	0.04	9.70	0.05	6.17	0.18	4.744 up	2.431 down	0.00	411.01	dynamin GTPase
75294	11.15	0.14	10.39	0.08	12.50	0.18	1.696 down	2.545 up	0.00	136.55	BCAT_beta_family
75295	13.34	0.06	12.39	0.02	11.98	0.16	1.935 down	2.580 down	0.00	79.59	Trehalose-6-phosphate synthase component TPS1 and related subunits
75361	11.71	0.07	11.14	0.05	9.61	0.19	1.483 down	4.283 down	0.00	149.15	unknown protein
75403	11.05	0.15	11.16	0.03	9.94	0.20	1.074 up	2.159 down	0.00	46.21	CsdB Selenocysteine lyase
75409	11.49	0.00	10.63	0.13	10.44	0.08	1.811 down	2.064 down	0.00	107.30	Phospholipid-translocating P-type ATPase. flippase; putative
75470	7.00	0.15	7.68	0.19	8.19	0.22	1.594 up	2.271 up	0.01	23.83	unknown protein
75609	13.14	0.03	13.37	0.10	14.26	0.12	1.178 up	2.180 up	0.00	89.17	Rpl18 gene encoding 60sRibosomal protein L18.
75704	9.58	0.10	10.48	0.16	10.64	0.09	1.867 up	2.085 up	0.00	62.22	Adenosine 5'-phosphosulfate kinase
75713	11.87	0.17	9.07	0.00	10.78	0.36	6.996 down	2.139 down	0.00	48.30	Cytochrome P450 CYP2 subfamily
75758	12.19	0.08	12.44	0.08	11.18	0.34	1.187 up	2.014 down	0.01	18.55	unknown protein
75884	10.22	0.12	8.43	0.12	7.36	0.79	3.472 down	7.271 down	0.02	14.53	unknown protein
75923	11.65	0.32	10.59	0.18	10.03	0.31	2.091 down	3.079 down	0.01	20.54	unknown protein
75985	11.16	0.10	12.43	0.16	13.10	0.24	2.407 up	3.846 up	0.00	61.91	Isochorismatase hydrolase
75995	12.90	0.26	13.24	0.29	14.04	0.26	1.266 up	2.204 up	0.02	14.42	ssDNA binding protein
76034	12.11	0.25	10.62	0.02	10.77	0.24	2.795 down	2.527 down	0.00	31.28	Iron/ascorbate family oxidoreductases
76073	12.03	0.27	12.70	0.03	13.46	0.32	1.587 up	2.692 up	0.01	18.96	U6 snRNA-associated Sm-like protein LSm6
76075	11.73	0.01	13.52	0.05	13.25	0.27	3.472 up	2.873 up	0.00	44.32	unknown protein
76136	12.16	0.21	12.02	0.18	9.83	0.28	1.097 down	4.994 down	0.00	82.07	unknown protein
76151	10.40	0.04	10.84	0.04	11.72	0.13	1.358 up	2.492 up	0.00	125.32	GT31 ß-glycosyltransferase
76200	11.85	0.07	12.04	0.06	13.12	0.27	1.138 up	2.411 up	0.00	30.42	unknown protein
76210	7.02	0.19	3.12	0.05	10.47	1.49	14.836 down	10.988 up	0.01	27.48	GH62 α-L-arabinofuranosidase ABF2
76220	13.13	0.01	12.86	0.21	12.11	0.14	1.205 down	2.030 down	0.00	38.42	Zn2Cys6 transcriptional regulator
76238	13.36	0.08	8.09	0.13	10.90	0.09	38.819 down	5.536 down	0.00	1524.02	plasma membrane H+ ATPase
76288	10.81	0.14	9.95	0.06	9.42	0.22	1.806 down	2.610 down	0.00	39.21	short chain dehydrogenase/reductase
76360	3.67	0.06	3.52	0.10	6.00	0.29	1.105 down	5.019 up	0.00	106.54	unknown protein. 1 TM domain
76366	12.29	0.10	7.39	0.04	10.51	0.16	29.853 down	3.451 down	0.00	699.15	NADH:flavin oxidoreductase/12-oxophytodienoate reductase

76421	11.00	0.06	10.53	0.10	9.77	0.31	1.382 down	2.333 down	0.01	18.06	aromatic ring-opening dioxygenase LigB subunit. putative
76453	12.40	0.13	12.63	0.09	13.52	0.34	1.175 up	2.179 up	0.02	13.71	MDR-type ABC transporters
76532	6.76	0.08	7.38	0.20	9.34	0.23	1.535 up	6.009 up	0.00	130.32	ribosomal protein (60S) P0
76543	11.28	0.10	11.20	0.05	10.27	0.08	1.052 down	2.013 down	0.00	150.60	ARF GTPase activator (Csx2). putative
76601	12.12	0.02	5.48	0.03	10.45	0.24	100.220 down	3.183 down	0.00	722.95	Sulfite oxidase. molybdopterin-binding component
76620	10.63	0.23	9.60	0.25	9.27	0.31	2.041 down	2.566 down	0.01	15.31	Glycerol-3-phosphate dehydrogenase
76633	11.50	0.02	11.62	0.15	9.29	0.49	1.083 up	4.622 down	0.00	35.25	MDR multidrug transporter
76659	7.35	0.19	7.65	0.25	9.85	0.23	1.222 up	5.631 up	0.00	108.65	unknown protein
76672	7.05	0.16	6.68	0.05	11.84	1.69	1.287 down	27.778 up	0.02	14.51	GH3 β-glucosidase BGL1/CEL3a
76677	11.37	0.10	11.19	0.12	10.05	0.21	1.132 down	2.490 down	0.00	49.36	Zn2Cys6 transcriptional regulator
76682	3.70	0.03	4.03	0.15	5.98	0.72	1.259 up	4.843 up	0.02	14.21	PDR-type ABC transporters
76705	11.75	0.11	11.87	0.00	10.54	0.18	1.082 up	2.325 down	0.00	74.16	Zn2Cys6 transcriptional regulator
76713	12.89	0.10	12.25	0.25	11.20	0.15	1.567 down	3.232 down	0.00	76.30	unknown protein
76740	10.18	0.00	10.76	0.29	11.91	0.20	1.492 up	3.307 up	0.00	53.58	zuotin
76766	12.59	0.03	11.97	0.02	11.43	0.21	1.542 down	2.240 down	0.00	35.28	xanthine/uracil permease
76822	9.21	0.06	11.86	0.09	8.00	0.29	6.287 up	2.302 down	0.00	193.82	unknown protein
76828	10.58	0.04	11.00	0.09	11.61	0.21	1.334 up	2.039 up	0.01	26.04	exosome complex exonuclease RRP40. putative
76897	14.55	0.13	14.11	0.12	13.07	0.41	1.363 down	2.796 down	0.01	15.68	MFS permease
76910	10.70	0.06	9.47	0.15	9.58	0.09	2.344 down	2.174 down	0.00	95.87	monocarboxylate transporter
76986	11.77	0.05	11.61	0.08	10.30	0.26	1.119 down	2.765 down	0.00	44.96	unknown protein
77138	12.04	0.12	12.25	0.16	13.30	0.31	1.151 up	2.395 up	0.01	20.72	unknown protein
77167	9.37	0.07	9.36	0.23	7.57	0.27	1.011 down	3.488 down	0.00	57.95	unknown protein
77202	11.22	0.03	8.48	0.12	9.03	0.11	6.662 down	4.546 down	0.00	421.43	short chain dehydrogenase/reductase
77291	9.94	0.28	10.38	0.03	8.60	0.38	1.359 up	2.532 down	0.01	24.44	Zn2Cys6 transcriptional regulator
77299	8.93	0.20	7.05	0.04	10.11	0.51	3.682 down	2.262 up	0.00	38.59	GH2 Exo-β-D-glucosaminidase GLS93
77423	12.08	0.06	10.50	0.05	9.70	0.54	2.978 down	5.195 down	0.01	21.60	Jacalin-like lectin
77495	12.71	0.15	12.95	0.25	13.83	0.12	1.182 up	2.172 up	0.00	40.03	eukaryotic translation initiation factor 3 subunit 11.
77506	13.60	0.03	13.37	0.03	12.54	0.15	1.173 down	2.078 down	0.00	66.93	GH5 β-glycosidase CEL5d
77517	10.15	0.08	10.32	0.01	11.92	0.15	1.129 up	3.423 up	0.00	195.91	MFS hexose transporter
77547	10.34	0.02	8.96	0.06	7.65	0.26	2.598 down	6.449 down	0.00	120.91	GT1 glycosyltransferase

77557	11.27	0.06	8.40	0.01	8.77	0.24	7.316 down	5.660 down	0.00	154.68	GT glycosyltransferases not yet assigned to a family. 3 TMs
77593	7.95	0.06	7.62	0.14	6.24	0.37	1.259 down	3.284 down	0.00	28.93	unknown protein
77602	14.05	0.04	13.46	0.11	12.91	0.29	1.503 down	2.212 down	0.01	16.99	A.a-trehalose-phosphate synthase
77629	8.57	0.03	9.46	0.11	10.04	0.47	1.847 up	2.756 up	0.03	10.66	unknown protein
77743	9.97	0.12	10.86	0.02	8.37	0.24	1.852 up	3.027 down	0.00	124.03	unknown protein
77878	11.32	0.04	11.19	0.00	13.11	0.14	1.096 down	3.446 up	0.00	284.36	C2H2 transcriptional regulator
77915	10.12	0.11	10.21	0.12	11.14	0.24	1.061 up	2.019 up	0.01	23.05	unknown protein
77932	10.51	0.12	11.04	0.18	12.22	0.36	1.446 up	3.282 up	0.01	25.78	Ribosomal protein L30.
77979	10.63	0.02	11.24	0.30	11.90	0.13	1.525 up	2.412 up	0.00	38.70	SmallSubunit. aps3. of the adaptor protein complex AP-3 of clathrin-coated vesicles; i
78010	12.14	0.00	12.01	0.02	10.86	0.17	1.096 down	2.423 down	0.00	89.58	ankyrin containing protein
78012	10.93	0.01	10.48	0.10	9.85	0.30	1.359 down	2.109 down	0.01	14.73	Sensory transduction histidine kinase
78037	11.34	0.01	11.05	0.03	9.73	0.25	1.220 down	3.040 down	0.00	59.78	unknown protein
78042	11.55	0.00	11.42	0.05	10.35	0.22	1.091 down	2.296 down	0.00	44.22	unknown protein
78264	10.33	0.09	11.02	0.04	11.48	0.17	1.611 up	2.211 up	0.00	45.79	Peptide methionine sulfoxide reductase
78274	8.07	0.12	7.77	0.33	9.13	0.23	1.231 down	2.084 up	0.01	27.07	phosphatidylinositol-3-phosphate 5-kinase; fab1
78301	12.93	0.03	12.63	0.25	11.75	0.14	1.228 down	2.251 down	0.00	45.19	cation diffusion facilitator family transporter
78357	13.88	0.26	13.71	0.25	12.39	0.14	1.122 down	2.796 down	0.00	50.77	NADH-quinone oxidoreductase. putative
78391	9.55	0.04	9.00	0.00	7.39	0.21	1.462 down	4.460 down	0.00	131.62	unknown protein
78465	11.54	0.01	11.95	0.04	13.06	0.17	1.324 up	2.856 up	0.00	106.76	siderophore transporter
78552	11.30	0.03	11.19	0.00	9.97	0.14	1.082 down	2.510 down	0.00	135.60	Aromatic L-amino acid decarbounknown proteinlase
78585	11.05	0.09	5.74	0.16	8.79	0.93	39.746 down	4.811 down	0.01	27.21	MFS permease
78611	13.06	0.03	12.16	0.07	11.02	0.26	1.873 down	4.125 down	0.00	70.52	phospholipase D
78639	11.45	0.01	13.71	0.19	12.98	0.13	4.778 up	2.880 up	0.00	150.16	Serine/threonine protein kinase
78679	9.74	0.03	10.17	0.21	10.78	0.15	1.345 up	2.057 up	0.00	33.79	mitochondrial carrier protein
78688	14.18	0.04	13.41	0.11	12.72	0.06	1.703 down	2.746 down	0.00	292.77	Winged helix repressor DNA-binding
78713	11.27	0.08	9.68	0.08	9.19	0.25	3.003 down	4.203 down	0.00	72.48	GH72 ß-(1-3) glucanosyltransferase
78740	9.06	0.04	9.11	0.07	10.22	0.29	1.037 up	2.234 up	0.01	24.94	v-SNARE Bet1. ER-Golgi complex
78773	13.46	0.02	13.36	0.04	12.41	0.15	1.069 down	2.061 down	0.00	69.67	long-chain-fatty-acid--CoA ligase 2
78792	10.79	0.02	9.88	0.01	8.75	0.25	1.875 down	4.111 down	0.00	81.04	unknown protein
78797	12.89	0.02	12.77	0.08	11.08	0.13	1.085 down	3.513 down	0.00	257.08	xanthine dehydrogenase

78799	11.05	0.01	10.95	0.04	9.27	0.29	1.076 down	3.434 down	0.00	60.14	Zn2Cys6 transcriptional regulator
78811	12.36	0.16	12.09	0.11	11.25	0.45	1.201 down	2.151 down	0.04	7.62	unknown protein
78828	12.00	0.13	13.51	0.12	13.57	0.47	2.840 up	2.953 up	0.02	13.12	carboxylesterase type B
78864	9.20	0.05	9.45	0.09	10.63	0.26	1.190 up	2.701 up	0.00	41.71	GcvTGlycine cleavage system T protein
78866	12.97	0.13	12.98	0.00	11.93	0.26	1.009 up	2.054 down	0.01	24.88	unknown protein
78895	11.44	0.01	11.45	0.14	9.99	0.21	1.006 up	2.724 down	0.00	68.48	Zn2Cys6 transcriptional regulator
78970	14.36	0.04	13.06	0.05	12.08	0.07	2.472 down	4.870 down	0.00	1013.94	mitochondrial (phosphate) carrier
78984	12.56	0.03	12.18	0.05	11.49	0.27	1.304 down	2.108 down	0.01	18.87	unknown protein
79034	10.12	0.05	10.67	0.00	11.60	0.04	1.463 up	2.784 up	0.00	1196.97	ABC1 family protein
79059	12.11	0.05	12.19	0.02	13.17	0.20	1.057 up	2.079 up	0.00	40.97	Myo-inositol-1-phosphate synthase. associated to cellulase signal transduction (PMID
79106	14.27	0.15	14.68	0.03	15.32	0.04	1.328 up	2.075 up	0.00	148.32	40s ribosomal protein L44e by homology to the corresponding protein of several yeas
79153	12.21	0.11	11.99	0.14	10.67	0.38	1.159 down	2.901 down	0.01	22.27	HyuA. N-methylhydantoinase A/acetone carboxylase. beta subunit
79187	10.09	0.01	11.14	0.19	11.33	0.06	2.070 up	2.371 up	0.00	111.19	DNA polymerase alpha-primase complex. polymerase-associated subunit B
79202	11.83	0.04	10.58	0.09	13.63	0.12	2.388 down	3.461 up	0.00	633.86	MFS permease. associated with cellulose signalling
79222	10.00	0.06	6.26	0.02	7.77	0.35	13.373 down	4.677 down	0.00	93.61	unknown protein
79229	11.34	0.04	11.92	0.19	12.67	0.14	1.497 up	2.508 up	0.00	65.15	elongation factor G (Tu) and the LepA membrane protein family.
79237	11.22	0.15	10.73	0.09	9.59	0.12	1.407 down	3.081 down	0.00	139.02	unknown protein
79271	13.81	0.14	13.59	0.08	12.21	0.16	1.162 down	3.028 down	0.00	110.77	malate synthase
79345	8.07	0.06	9.79	0.21	9.90	0.50	3.286 up	3.544 up	0.01	15.00	unknown protein. Mpv17/PMP22 family
79361	10.00	0.14	11.17	0.06	11.26	0.10	2.257 up	2.408 up	0.00	112.48	unknown protein
79396	14.17	0.05	12.88	0.06	13.11	0.27	2.453 down	2.093 down	0.01	22.97	GT2 ß-glycosyltransferase. related to hyaluronan synthases
79426	8.95	0.05	9.45	0.06	10.04	0.31	1.416 up	2.139 up	0.02	14.49	unknown protein
79461	12.65	0.23	11.97	0.02	11.59	0.15	1.603 down	2.087 down	0.00	30.74	unknown protein
79487	10.89	0.02	11.22	0.02	12.14	0.31	1.260 up	2.392 up	0.01	21.82	Mannose-P-dolichol utilization defect 1 protein
79565	9.72	0.15	10.62	0.08	10.92	0.28	1.870 up	2.302 up	0.01	18.15	Thioredoxin reductase TrxB
79602	10.06	0.08	10.07	0.07	7.88	0.44	1.010 up	4.513 down	0.00	40.67	GH81 endo-1.3-β-glucanase
79606	13.12	0.07	12.41	0.04	12.00	0.15	1.639 down	2.177 down	0.00	58.33	GH115 xylan-α-1.2-glucuronidase or α-(4-O-methyl)-glucuronidase
79644	9.95	0.11	9.44	0.08	11.12	0.37	1.424 down	2.247 up	0.01	24.81	metal ion transporter SMF2
79661	11.84	0.21	11.87	0.05	10.63	0.25	1.018 up	2.319 down	0.00	33.14	unknown protein
79725	10.00	0.03	9.87	0.19	8.37	0.10	1.094 down	3.115 down	0.00	179.55	Zn2Cys6 transcriptional regulator

79726	9.56	0.02	12.54	0.06	12.74	0.39	7.847 up	9.055 up	0.00	80.68	unknown protein
79741	9.16	0.12	8.30	0.09	10.30	0.36	1.807 down	2.212 up	0.00	34.55	ER-bound Farnesyl-diphosphate farnesyltransferase
79756	10.21	0.01	10.43	0.03	8.93	0.19	1.166 up	2.416 down	0.00	84.60	F-Box/WD40 repeat protein FWD-1. targets the clock component FRQ for degradation
79779	8.12	0.01	8.82	0.08	10.56	0.62	1.626 up	5.436 up	0.01	20.20	unknown protein
79807	12.64	0.03	12.51	0.12	11.58	0.38	1.096 down	2.083 down	0.02	10.82	Aspartictype endopeptidase ctsD
79816	5.84	0.06	5.57	0.28	7.72	0.46	1.212 down	3.680 up	0.01	28.23	unknown protein; secreted
79823	10.21	0.28	9.81	0.01	9.14	0.21	1.320 down	2.100 down	0.01	19.97	DCL2. Dicer-like protein. involved in quelling
79933	8.82	0.31	9.16	0.10	10.47	0.34	1.273 up	3.151 up	0.01	24.57	mitochondrial ribosomal protein MRPL6.
79947	11.67	0.13	11.34	0.00	13.01	0.14	1.257 down	2.543 up	0.00	160.60	DNA-dependent RNA polymerase
79960	10.75	0.20	12.41	0.09	12.28	0.12	3.156 up	2.894 up	0.00	107.20	GH47 α-1.2-mannosidase
80019	13.35	0.14	14.15	0.01	14.60	0.23	1.739 up	2.366 up	0.00	28.81	short-chain dehydrogenase/reductase
80026	12.50	0.20	13.64	0.02	14.16	0.39	2.194 up	3.143 up	0.01	17.98	MFS permease
80034	12.23	0.07	12.52	0.07	11.17	0.16	1.226 up	2.088 down	0.00	85.54	DadA Glycine/D-amino acid oxidases (deaminating) [Amino acid transport and metabo
80056	6.62	0.14	7.67	0.20	8.38	0.53	2.072 up	3.390 up	0.02	11.44	RNA 3'-terminal phosphate cyclase . putative
80058	11.32	0.03	7.80	0.23	9.23	0.38	11.514 down	4.257 down	0.00	64.66	MFS permease
80096	12.88	0.04	12.76	0.05	11.57	0.43	1.083 down	2.464 down	0.02	13.76	unknown protein
80105	11.80	0.04	11.81	0.10	10.68	0.23	1.011 up	2.166 down	0.00	35.59	unknown protein
80137	11.09	0.10	10.84	0.12	9.92	0.22	1.188 down	2.247 down	0.00	34.51	unknown protein
80167	12.99	0.06	13.43	0.14	14.07	0.21	1.362 up	2.118 up	0.01	27.28	histone H4 variant
80211	6.02	0.05	6.56	0.01	8.77	0.31	1.450 up	6.721 up	0.00	106.49	ribosomal protein L14.
80288	11.80	0.06	12.06	0.05	13.24	0.33	1.203 up	2.718 up	0.01	26.07	cytochrome c oxidase polypeptide V
80291	12.38	0.04	12.23	0.02	11.02	0.30	1.110 down	2.580 down	0.00	30.64	Zn2Cys6 transcriptional regulator
80340	9.94	0.11	12.27	0.04	12.59	0.16	5.037 up	6.295 up	0.00	284.96	GT α-1.6-mannosyltransferase
80365	11.98	0.02	12.13	0.15	10.84	0.19	1.112 up	2.200 down	0.00	56.01	unknown protein
80425	10.33	0.05	9.88	0.04	9.18	0.21	1.363 down	2.221 down	0.00	35.11	unknown protein
80441	11.62	0.05	12.17	0.21	12.83	0.35	1.469 up	2.322 up	0.02	12.53	unknown protein
80523	10.05	0.07	9.78	0.09	8.67	0.18	1.208 down	2.604 down	0.00	71.69	pH-response regulator protein palC
80528	10.64	0.32	12.63	0.02	11.71	0.08	3.962 up	2.091 up	0.00	80.10	Gß-WD40 protein
80553	12.91	0.04	12.80	0.11	11.73	0.12	1.083 down	2.260 down	0.00	112.98	ADP-ribosylation factor like protein
80636	12.70	0.02	12.40	0.06	11.57	0.27	1.232 down	2.196 down	0.01	22.41	unknown protein

80639	3.54	0.13	4.93	0.30	5.56	0.47	2.616 up	4.050 up	0.01	17.71	iron transporter
80645	12.86	0.02	12.85	0.05	11.01	0.48	1.004 down	3.596 down	0.01	24.23	Amidase
80654	9.34	0.00	5.79	0.09	7.39	0.12	11.701 down	3.854 down	0.00	616.24	UDP-glucuronosyl and UDP-glucosyl transferase
80659	11.38	0.03	4.77	0.32	8.84	0.29	97.770 down	5.813 down	0.00	310.61	alcohol oxidase AOX1
80668	11.31	0.12	10.49	0.06	9.62	0.19	1.769 down	3.231 down	0.00	78.97	Zn2Cys6 transcriptional regulator
80685	10.18	0.01	10.58	0.01	8.09	0.35	1.314 up	4.278 down	0.00	74.07	unknown protein
80695	11.82	0.22	11.98	0.22	10.79	0.26	1.119 up	2.036 down	0.01	20.29	half-sized ABC transporter
80714	12.33	0.16	12.56	0.15	10.94	0.35	1.174 up	2.611 down	0.01	27.37	Dolichyl-phosphate-mannose a-mannosyltransferase
80733	6.63	0.00	7.20	0.17	7.86	0.09	1.493 up	2.345 up	0.00	99.06	unknown protein
80758	12.83	0.08	12.70	0.09	11.81	0.04	1.095 down	2.026 down	0.00	235.48	serine/threonine protein kinase nrc2
80843	2.56	0.10	2.50	0.11	4.05	0.16	1.043 down	2.809 up	0.00	113.23	26S proteasome regulatory complex subunit Rpn8
80871	11.71	0.15	10.92	0.10	9.91	0.41	1.732 down	3.483 down	0.01	20.85	unknown protein
80875	10.50	0.01	11.05	0.11	8.39	0.79	1.465 up	4.312 down	0.01	15.59	MFS permease
80980	11.74	0.07	14.20	0.08	14.00	0.15	5.500 up	4.778 up	0.00	253.40	peptidyl arginine deiminase
81004	8.68	0.02	8.23	0.09	7.54	0.14	1.369 down	2.202 down	0.00	65.45	aspartyl protease
81019	11.00	0.24	10.28	0.08	8.49	0.55	1.642 down	5.665 down	0.01	25.18	Zinc-containing alcohol dehydrogenase
81022	13.01	0.06	13.08	0.10	11.24	0.18	1.047 up	3.411 down	0.00	151.70	allantoate permease. 10 TM domains
81058	9.14	0.15	8.02	0.05	7.32	0.25	2.175 down	3.544 down	0.00	53.14	peptidase family S58
81082	7.86	0.09	11.34	0.10	12.52	1.11	11.207 up	25.313 up	0.01	19.53	aquaglyceroporin
81087	10.24	0.09	6.86	0.06	11.84	0.55	10.395 down	3.035 up	0.00	88.30	aminopeptidase Y
81096	10.40	0.14	10.44	0.08	14.28	0.11	1.024 up	14.686 up	0.00	1233.48	ferric reductase
81125	13.56	0.02	12.48	0.05	12.24	0.24	2.107 down	2.495 down	0.00	33.31	Amino acid permease
81150	9.24	0.09	9.84	0.16	8.06	0.24	1.513 up	2.272 down	0.00	56.07	unknown protein
81183	11.63	0.10	11.44	0.10	10.38	0.20	1.134 down	2.366 down	0.00	48.16	unknown protein
81271	10.46	0.09	11.72	0.04	12.10	0.43	2.399 up	3.113 up	0.01	15.84	xylitol dehydrogenase XDH1
81275	12.46	0.14	11.22	0.12	10.76	0.08	2.360 down	3.247 down	0.00	179.70	phytanoyl-CoA dioxygenase family protein
81362	8.71	0.08	9.08	0.03	9.85	0.05	1.290 up	2.199 up	0.00	340.39	Lipoate synthase
81383	11.62	0.21	13.23	0.12	14.27	0.17	3.063 up	6.292 up	0.00	163.75	GPCR . contains RGS domain
81389	12.22	0.02	10.34	0.09	9.96	0.18	3.699 down	4.788 down	0.00	166.38	phosphate/H+ symporter
81420	8.72	0.07	7.19	0.09	7.31	0.44	2.875 down	2.643 down	0.02	13.01	Argonaute siRNA chaperone (ARC) complex subunit

81423	11.59	0.21	11.46	0.31	12.89	0.29	1.091 down	2.463 up	0.01	23.69	unknown protein
81454	12.15	0.10	12.12	0.14	10.70	0.31	1.024 down	2.735 down	0.00	31.87	unknown protein
81473	9.92	0.05	10.28	0.04	10.95	0.39	1.282 up	2.031 up	0.04	8.56	caffeine-induced death protein 2
81511	11.39	0.09	10.52	0.04	10.09	0.25	1.824 down	2.453 down	0.00	28.53	formamidase
81609	11.86	0.07	11.44	0.07	10.84	0.31	1.338 down	2.033 down	0.02	12.39	GH15
81623	12.73	0.04	11.84	0.11	11.43	0.08	1.859 down	2.465 down	0.00	185.24	unknown protein. only in Hypocreaceae
81646	12.64	0.16	12.04	0.04	11.47	0.04	1.513 down	2.238 down	0.00	141.24	NADPH oxidase
81659	12.31	0.04	8.98	0.08	10.44	0.17	10.047 down	3.646 down	0.00	281.55	unknown protein
81676	10.96	0.02	10.38	0.12	8.48	0.53	1.496 down	5.583 down	0.00	29.12	salicylate hydroxylase
81700	12.67	0.08	12.48	0.11	11.24	0.06	1.144 down	2.691 down	0.00	317.99	mannose-6-phosphate isomerase
81720	11.65	0.01	11.36	0.06	10.25	0.26	1.222 down	2.629 down	0.00	37.61	Ume5 gen
81730	10.84	0.02	11.13	0.16	11.91	0.23	1.224 up	2.100 up	0.01	24.99	glutaredoxin
81774	12.24	0.09	12.04	0.02	10.75	0.20	1.141 down	2.798 down	0.00	72.24	unknown protein
81843	10.80	0.03	9.34	0.12	7.49	0.46	2.746 down	9.875 down	0.00	60.40	Alkaline phosphatase
81906	12.39	0.20	12.94	0.28	13.60	0.08	1.461 up	2.310 up	0.00	36.93	unknown protein
81911	12.61	0.08	12.46	0.12	11.41	0.18	1.107 down	2.299 down	0.00	56.94	unknown protein
81920	11.55	0.07	11.72	0.05	12.78	0.26	1.128 up	2.336 up	0.00	30.11	SSCRP
81955	2.89	0.27	2.90	0.09	4.46	0.19	1.008 up	2.970 up	0.00	63.52	nitrate reductase
81979	13.00	0.03	9.76	0.07	10.67	0.13	9.476 down	5.025 down	0.00	502.20	glutathione-S-transferase
82037	12.52	0.02	12.74	0.01	11.08	0.06	1.163 up	2.719 down	0.00	1080.27	Inorganic phosphate transporter
82049	10.25	0.12	10.91	0.02	13.17	0.29	1.577 up	7.546 up	0.00	124.96	unknown protein
82095	6.16	0.06	6.23	0.01	7.49	0.51	1.055 up	2.518 up	0.02	10.85	ammonium transporter. high affinity
82105	8.91	0.26	7.45	0.09	6.81	0.40	2.755 down	4.288 down	0.01	26.34	PDR-type ABC transporters
82204	8.08	0.16	7.92	0.03	6.79	0.37	1.113 down	2.439 down	0.01	16.85	ammonium transporter. high affinity
82235	12.76	0.10	12.99	0.11	14.27	0.18	1.174 up	2.844 up	0.00	82.92	GH31 α-glucosidase
82246	11.95	0.01	12.39	0.07	13.11	0.10	1.358 up	2.245 up	0.00	134.65	GCPR. mPR-type
82286	12.36	0.02	12.93	0.02	13.39	0.29	1.478 up	2.044 up	0.02	13.92	unknown protein
82309	11.83	0.00	11.35	0.02	9.45	0.52	1.390 down	5.190 down	0.01	28.35	MFS permease
82321	11.04	0.07	4.10	0.23	9.46	0.74	122.123 down	2.981 down	0.00	81.69	Aquaporin
82327	12.55	0.05	10.27	0.00	10.04	0.28	4.846 down	5.701 down	0.00	94.75	PDR-type ABC transporters

82374	12.43	0.04	11.41	0.07	10.39	0.75	2.028 down	4.120 down	0.04	8.47	unknown protein
82403	13.55	0.08	13.28	0.07	11.61	0.14	1.198 down	3.822 down	0.00	238.93	Acyl-CoA dehydrogenase
82425	4.54	0.05	4.64	0.22	5.97	0.13	1.070 up	2.685 up	0.00	90.83	unknown protein
82523	9.32	0.16	9.12	0.13	8.22	0.17	1.146 down	2.139 down	0.00	39.93	unknown protein
82560	8.44	0.03	9.50	0.12	9.95	0.25	2.091 up	2.857 up	0.00	37.30	Ribokinase
82591	12.47	0.05	10.83	0.04	10.72	0.13	3.120 down	3.377 down	0.00	202.45	unknown protein with oxidoreductase domain
82606	11.08	0.08	11.17	0.07	12.20	0.30	1.068 up	2.174 up	0.01	20.46	Mn2+ homeostasis protein Per1
82616	12.20	0.23	9.83	0.11	10.03	0.33	5.179 down	4.507 down	0.00	48.54	GH5 membrane bound endoglucanase CEL5b
82619	12.74	0.02	12.92	0.03	9.54	0.48	1.131 up	9.196 down	0.00	78.59	arginosuccinate synthetase
82626	8.46	0.30	8.59	0.04	6.77	0.49	1.093 up	3.230 down	0.01	18.72	half-sized ABC transporter
82628	4.03	0.31	6.08	0.37	5.50	0.19	4.132 up	2.764 up	0.00	33.82	acetyltransferase SidF
82633	12.16	0.07	10.18	0.08	9.90	0.29	3.948 down	4.784 down	0.00	67.84	GH72 β-1 3-glucanosyltransferase
82662	14.11	0.09	7.87	0.06	11.87	0.20	75.475 down	4.705 down	0.00	753.52	Epl1/Sm1
102383	11.29	0.11	10.66	0.07	9.28	0.16	1.548 down	4.029 down	0.00	167.85	unknown protein
102385	2.87	0.14	2.85	0.19	4.29	0.12	1.011 down	2.671 up	0.00	105.82	unknown protein
102386	6.71	0.25	7.40	0.12	7.97	0.26	1.618 up	2.391 up	0.01	19.16	unique protein
102411	10.42	0.10	9.81	0.06	9.02	0.21	1.522 down	2.640 down	0.00	48.26	unknown protein
102416	10.58	0.12	9.48	0.13	9.46	0.10	2.132 down	2.163 down	0.00	76.63	amino acid permease PotE
102454	6.28	0.13	7.08	0.00	7.39	0.17	1.741 up	2.160 up	0.00	41.71	unknown protein
102467	4.80	0.15	4.22	0.39	6.49	0.09	1.499 down	3.218 up	0.00	101.95	unknown protein
102499	2.71	0.06	2.66	0.04	5.44	0.71	1.036 down	6.618 up	0.01	25.12	Zn2Cys6 transcriptional regulator
102500	5.32	0.02	5.12	0.17	7.26	0.27	1.148 down	3.845 up	0.00	82.90	MRSP1/expansin-like
102567	2.61	0.05	3.22	0.45	4.23	0.18	1.516 up	3.056 up	0.00	32.10	unknown protein
102593	9.10	0.21	9.73	0.10	10.44	0.18	1.550 up	2.534 up	0.00	40.50	Mitochondrial ribosomal protein L17
102637	3.06	0.09	3.95	0.08	4.92	0.11	1.847 up	3.624 up	0.00	216.12	unique protein
102680	2.42	0.01	2.34	0.11	3.67	0.02	1.058 down	2.377 up	0.00	643.98	unique protein
102738	12.18	0.00	10.17	0.11	11.09	0.52	4.027 down	2.121 down	0.02	12.30	unknown protein
102773	3.23	0.07	3.98	0.12	5.86	0.16	1.674 up	6.196 up	0.00	279.58	unknown protein
102774	4.89	0.02	5.37	0.37	7.28	0.51	1.389 up	5.248 up	0.01	25.71	unknown protein
102779	6.03	0.03	7.31	0.15	7.14	0.30	2.439 up	2.160 up	0.01	18.27	unknown protein

102780	5.79	0.19	6.21	0.21	7.84	0.55	1.340 up	4.147 up	0.01	17.45	unknown protein
102787	5.81	0.18	6.72	0.03	7.19	0.32	1.879 up	2.600 up	0.01	18.36	unique protein
102788	2.81	0.29	3.06	0.10	4.57	0.11	1.186 up	3.375 up	0.00	99.94	unique protein
102816	12.30	0.15	11.70	0.07	11.07	0.11	1.513 down	2.344 down	0.00	82.91	Zn2Cys6 transcriptional regulator
102830	12.69	0.24	10.56	0.01	11.18	0.33	4.386 down	2.858 down	0.00	31.43	unknown protein
102846	10.73	0.20	10.43	0.26	9.57	0.22	1.230 down	2.239 down	0.01	20.71	unknown protein
102850	9.00	0.04	9.04	0.03	7.85	0.05	1.029 up	2.222 down	0.00	696.54	unique protein
102851	9.23	0.12	11.30	0.17	12.54	1.01	4.207 up	9.908 up	0.02	11.70	SSCRP
102863	10.44	0.01	10.29	0.01	8.92	0.24	1.103 down	2.858 down	0.00	58.72	unknown protein
102876	6.34	0.19	6.39	0.02	5.30	0.37	1.040 up	2.045 down	0.02	12.39	unique protein
102887	4.50	0.04	5.04	0.11	6.19	0.38	1.447 up	3.225 up	0.01	24.29	unique protein
102892	13.39	0.10	13.17	0.10	12.01	0.26	1.165 down	2.607 down	0.00	36.21	unknown protein
102906	10.74	0.05	9.19	0.13	8.62	0.27	2.927 down	4.337 down	0.00	60.77	unique protein
102920	12.11	0.00	9.90	0.07	9.47	0.25	4.625 down	6.239 down	0.00	121.53	C2H2 transcriptional regulator
102927	3.52	0.04	4.01	0.33	4.89	0.08	1.412 up	2.592 up	0.00	53.72	Serine/threonine protein kinase
102933	8.32	0.02	9.19	0.08	6.35	0.23	1.831 up	3.914 down	0.00	189.43	unknown protein. only in Gibberella
102935	3.11	0.07	3.28	0.22	4.68	0.09	1.124 up	2.963 up	0.00	146.45	unknown protein
102936	5.65	0.07	5.95	0.11	7.48	0.20	1.234 up	3.549 up	0.00	104.24	unknown protein
102953	9.59	0.01	7.98	0.07	7.35	0.15	3.052 down	4.708 down	0.00	218.57	dityrosine transporter. required for spore wall synthesis
102960	10.84	0.04	11.73	0.09	12.04	0.25	1.842 up	2.297 up	0.01	25.15	unique protein. 2 TM
102976	7.64	0.16	8.31	0.24	8.91	0.25	1.588 up	2.411 up	0.01	20.09	unknown protein
102985	12.37	0.07	11.48	0.08	10.68	0.15	1.861 down	3.234 down	0.00	126.08	unknown protein
102999	6.79	0.09	9.41	0.12	10.31	0.39	6.126 up	11.491 up	0.00	85.82	unknown protein
103015	10.32	0.11	7.17	0.03	6.94	0.26	8.856 down	10.365 down	0.00	194.28	unknown protein
103016	2.73	0.10	2.65	0.12	4.16	0.13	1.057 down	2.695 up	0.00	144.94	unique protein
103032	3.25	0.11	3.53	0.14	5.02	0.16	1.208 up	3.405 up	0.00	125.26	unknown protein
103034	8.45	0.31	6.64	0.06	5.17	0.12	3.507 down	9.746 down	0.00	251.03	Zn2Cys6 transcriptional regulator
103041	5.10	0.69	2.83	0.26	7.73	0.63	4.814 down	6.212 up	0.00	48.24	acetamidase
103043	2.20	0.01	2.34	0.10	3.79	0.10	1.105 up	3.003 up	0.00	293.51	unknown protein
103044	3.35	0.08	3.55	0.38	4.83	0.32	1.148 up	2.795 up	0.01	21.05	unique protein

103049	5.31	0.24	4.06	0.41	6.62	0.24	2.382 down	2.468 up	0.00	56.32	GH28 endo-polygalacturonase
103059	6.74	0.05	5.70	0.06	7.76	0.43	2.053 down	2.031 up	0.01	25.68	unknown protein
103061	4.04	0.42	4.41	0.01	6.10	0.64	1.298 up	4.189 up	0.02	12.75	unknown protein
103063	6.61	0.16	5.82	0.40	8.55	0.27	1.738 down	3.827 up	0.00	70.38	unknown protein
103064	8.77	0.04	6.06	0.00	10.08	0.24	6.552 down	2.463 up	0.00	297.95	unknown protein
103065	6.67	0.06	7.14	0.23	8.29	0.32	1.378 up	3.061 up	0.01	27.28	unknown protein
103073	5.68	0.00	5.96	0.13	6.71	0.22	1.205 up	2.041 up	0.01	25.16	unknown protein
103112	6.88	0.01	8.23	0.09	8.92	0.27	2.549 up	4.117 up	0.00	63.11	unknown protein
103114	3.75	0.00	3.86	0.24	5.39	0.29	1.079 up	3.106 up	0.00	39.63	unknown protein
103119	4.08	0.49	4.24	0.28	6.09	0.16	1.115 up	4.041 up	0.00	47.34	membrane dipeptidase GliJ
103121	3.26	0.21	3.52	0.00	5.27	0.34	1.200 up	4.041 up	0.00	45.63	unique protein
103122	2.80	0.14	2.79	0.04	4.66	0.14	1.000 down	3.653 up	0.00	213.92	Zn2Cys6 transcriptional regulator
103129	4.98	0.24	5.13	0.43	6.21	0.20	1.111 up	2.343 up	0.01	18.78	unknown protein
103130	4.19	0.10	4.59	0.03	5.60	0.19	1.315 up	2.650 up	0.00	65.57	unique protein
103135	4.57	0.31	4.67	0.21	8.59	1.65	1.070 up	16.190 up	0.03	9.45	SSCRP
103136	3.54	0.10	4.23	0.10	4.83	0.11	1.614 up	2.455 up	0.00	107.96	Glycolate oxidase
103138	10.95	0.08	10.91	0.02	9.65	0.24	1.029 down	2.460 down	0.00	44.63	Zn2Cys6 transcriptional regulator
103145	10.29	0.18	8.54	0.05	8.48	0.31	3.357 down	3.491 down	0.00	36.17	unique protein
103147	14.36	0.03	13.55	0.10	11.78	0.35	1.749 down	5.977 down	0.00	66.35	unknown protein
103149	10.91	0.07	6.74	0.01	8.20	0.18	18.085 down	6.549 down	0.00	460.98	AAA ATPase
103155	3.21	0.06	3.42	0.30	5.07	0.22	1.153 up	3.630 up	0.00	62.78	unknown protein
103156	8.28	0.05	3.98	0.21	6.60	0.18	19.725 down	3.204 down	0.00	341.30	unknown protein
103157	11.16	0.00	11.19	0.18	8.88	0.51	1.024 up	4.853 down	0.00	32.63	unknown protein
103158	9.69	0.07	8.31	0.07	6.75	0.35	2.610 down	7.664 down	0.00	78.58	Zn2Cys6 transcriptional regulator
103172	7.16	0.15	4.60	0.01	6.14	0.20	5.905 down	2.021 down	0.00	113.95	unique protein
103174	3.05	0.09	3.06	0.11	5.05	0.15	1.007 up	4.014 up	0.00	226.43	SSCRP
103175	3.14	0.12	3.70	0.22	4.99	0.17	1.476 up	3.592 up	0.00	89.00	epoxide hydrolase. putative
103176	4.38	0.33	4.39	0.18	5.73	0.24	1.004 up	2.534 up	0.00	29.14	unknown protein
103177	4.36	0.33	5.41	0.11	5.62	0.24	2.072 up	2.395 up	0.01	18.51	glutathionine S-transferase
103186	4.26	0.17	3.81	0.29	5.34	0.17	1.367 down	2.113 up	0.00	45.58	unknown protein

103189	6.82	0.20	5.20	0.05	5.45	0.32	3.073 down	2.586 down	0.01	23.09	HET protein
103193	8.14	0.16	4.80	0.55	5.30	0.18	10.151 down	7.186 down	0.00	81.59	ankyrin
103205	6.72	0.05	9.04	0.38	8.97	0.68	5.009 up	4.748 up	0.02	12.54	unknown protein
103215	11.54	0.20	11.06	0.05	9.92	0.09	1.392 down	3.073 down	0.00	159.13	Cytochrome P450 CYP4/CYP19/CYP26 subfamilies
103222	12.94	0.12	13.54	0.10	14.12	0.16	1.518 up	2.257 up	0.00	45.27	Ribosomal protein S29 (S14 family) by homology to the corresponding protein of N. cr
103230	11.42	0.05	11.25	0.08	9.16	0.22	1.127 down	4.801 down	0.00	154.68	Zn2Cys6 transcriptional regulator
103236	9.66	0.21	9.01	0.01	11.13	0.12	1.562 down	2.773 up	0.00	195.76	unknown protein
103286	2.54	0.08	2.92	0.43	4.77	0.23	1.300 up	4.691 up	0.00	60.77	unique protein
103335	7.60	0.31	7.95	0.15	9.98	0.57	1.274 up	5.229 up	0.01	22.59	unknown protein
103356	13.29	0.01	13.12	0.07	12.17	0.16	1.124 down	2.175 down	0.00	67.08	Vacuolar transporter chaperone
103372	12.47	0.09	11.75	0.01	10.97	0.24	1.646 down	2.811 down	0.00	44.07	BZIP transcriptional regulator
103394	3.38	0.24	3.27	0.10	5.06	0.16	1.079 down	3.209 up	0.00	103.46	unknown protein
103395	3.14	0.27	3.15	0.05	4.77	0.26	1.010 up	3.104 up	0.00	47.50	unknown protein
103403	3.53	0.11	3.90	0.45	5.19	0.19	1.289 up	3.154 up	0.00	34.59	unknown protein
103423	5.98	0.19	5.85	0.09	7.37	0.44	1.092 down	2.619 up	0.01	17.14	SSCRP
103447	9.47	0.12	9.61	0.04	8.26	0.30	1.104 up	2.307 down	0.00	29.21	unknown protein
103455	9.41	0.01	11.00	0.01	11.16	0.24	3.006 up	3.367 up	0.00	63.20	unknown protein
103482	11.47	0.00	10.68	0.07	9.27	0.38	1.723 down	4.604 down	0.00	40.21	transcriptional activator with ariadne RING finger
103506	12.73	0.01	11.92	0.24	11.59	0.23	1.748 down	2.197 down	0.01	20.11	unknown protein
103537	6.21	0.19	10.65	0.01	10.18	0.33	21.752 up	15.724 up	0.00	182.26	GCN5-N-acetyltransferase
103651	3.33	0.24	3.73	0.23	4.86	0.12	1.319 up	2.880 up	0.00	58.96	unknown protein
103653	2.15	0.10	2.09	0.02	3.47	0.07	1.046 down	2.489 up	0.00	379.26	unique protein
103655	8.44	0.06	9.17	0.04	9.83	0.14	1.654 up	2.620 up	0.00	109.18	rad55
103668	9.95	0.07	9.52	0.02	8.63	0.56	1.348 down	2.508 down	0.05	7.11	unique protein
103671	11.33	0.08	10.65	0.03	9.40	0.33	1.602 down	3.810 down	0.00	42.11	unknown protein
103683	6.18	0.35	6.91	0.04	5.13	0.11	1.654 up	2.075 down	0.00	73.27	unique protein
103694	11.90	0.14	11.72	0.14	9.26	0.46	1.135 down	6.223 down	0.00	48.04	PTH11 GPCR
103715	5.65	0.21	6.10	0.17	7.62	0.41	1.364 up	3.923 up	0.01	27.50	unique protein
103778	3.65	0.07	3.59	0.07	6.40	0.40	1.045 down	6.708 up	0.00	79.82	unique protein
103798	11.55	0.16	10.22	0.02	9.72	0.32	2.520 down	3.567 down	0.00	33.46	SSCRP

103799	10.91	0.01	10.32	0.15	9.40	0.17	1.497 down	2.834 down	0.00	76.07	unique protein
103804	12.60	0.17	12.22	0.15	11.32	0.22	1.300 down	2.422 down	0.00	31.56	SWI-SNF chromatin-remodeling complex protein
103852	11.98	0.04	12.06	0.03	10.78	0.23	1.057 up	2.304 down	0.00	49.70	unknown protein
103863	4.35	0.08	4.64	0.02	5.84	0.32	1.224 up	2.825 up	0.00	30.71	unknown protein
103886	14.40	0.24	14.27	0.16	12.95	0.14	1.097 down	2.746 down	0.00	66.66	unknown protein
103898	8.04	0.16	9.09	0.30	9.25	0.31	2.071 up	2.304 up	0.02	12.59	unknown protein
103900	3.16	0.15	3.22	0.19	4.96	0.09	1.039 up	3.474 up	0.00	191.33	unknown protein
103907	6.95	0.30	7.42	0.00	5.06	0.61	1.380 up	3.721 down	0.01	19.15	unknown protein
103930	12.39	0.05	12.06	0.00	10.94	0.39	1.251 down	2.725 down	0.01	18.13	unknown protein
103949	7.71	0.11	7.94	0.15	8.96	0.35	1.170 up	2.376 up	0.01	16.06	unknown protein
103959	3.26	0.21	3.16	0.08	4.72	0.12	1.069 down	2.748 up	0.00	123.91	unique protein
103960	8.69	0.12	7.45	0.08	7.05	0.25	2.353 down	3.107 down	0.00	43.86	nitrilase
103973	5.61	0.50	6.10	0.21	7.84	0.59	1.398 up	4.665 up	0.01	15.14	unknown protein
104000	10.69	0.14	11.63	0.12	11.80	0.12	1.918 up	2.158 up	0.00	53.59	DNA repair protein rad18
104016	11.64	0.10	11.81	0.05	10.59	0.12	1.120 up	2.070 down	0.00	120.11	unknown protein
104046	3.28	0.14	3.67	0.43	4.79	0.11	1.311 up	2.853 up	0.00	37.43	unique protein
104050	4.20	0.35	3.56	0.41	8.09	0.98	1.559 down	14.834 up	0.01	28.16	SSCRP
104054	3.82	0.03	3.88	0.14	5.72	0.24	1.043 up	3.720 up	0.00	88.32	unknown protein
104059	3.45	0.37	3.36	0.28	5.30	0.15	1.067 down	3.602 up	0.00	65.02	short chain dehydrognease/reductase
104060	3.25	0.38	2.99	0.20	4.97	0.32	1.197 down	3.301 up	0.00	35.54	short chain dehydrognease/reductase
104064	3.52	0.29	3.44	0.13	5.39	0.42	1.057 down	3.660 up	0.00	29.44	unknown protein
104067	3.70	0.01	3.48	0.11	6.35	0.61	1.162 down	6.266 up	0.00	33.90	unknown protein
104071	4.47	0.05	4.44	0.39	5.83	0.28	1.019 down	2.566 up	0.01	23.71	unknown protein
104075	10.93	0.22	10.65	0.22	9.52	0.41	1.213 down	2.653 down	0.02	13.92	Zn2Cys6 transcriptional regulator
104077	10.78	0.02	10.41	0.06	7.17	0.92	1.291 down	12.210 down	0.01	23.05	Amino acid transporter PotE
104079	4.72	0.22	4.56	0.09	6.57	0.32	1.112 down	3.604 up	0.00	51.52	MRSP1/expansin-like
104081	5.55	0.13	5.51	0.20	7.12	0.73	1.026 down	2.959 up	0.04	7.62	coenzyme F420-dependent N5.N10-methylene tetrahydromethanopterin reductase
104106	4.16	0.11	4.05	0.14	5.47	0.16	1.073 down	2.488 up	0.00	90.42	ADP/ATP carrier protein
104109	4.44	0.13	4.60	0.02	5.66	0.20	1.121 up	2.331 up	0.00	47.52	unique protein
104118	3.27	0.25	3.48	0.27	5.81	0.44	1.156 up	5.813 up	0.00	41.37	unique protein

104140	9.55	0.01	10.93	0.18	10.91	0.27	2.606 up	2.557 up	0.01	26.90	3-hydroxi-isobutyrate dehydrogenase
104157	3.30	0.00	3.27	0.36	4.49	0.05	1.015 down	2.282 up	0.00	53.68	unique protein
104168	6.42	0.07	7.75	0.00	7.55	0.35	2.519 up	2.197 up	0.01	14.76	unknown protein
104173	5.53	0.02	4.90	0.05	6.84	0.21	1.554 down	2.475 up	0.00	103.48	unique protein
104175	4.33	0.13	4.61	0.13	5.71	0.15	1.211 up	2.594 up	0.00	79.90	unique protein
104180	9.55	0.14	6.27	0.46	8.29	0.13	9.686 down	2.395 down	0.00	97.31	Ankyrin
104181	4.71	0.11	6.34	0.14	6.93	0.26	3.106 up	4.664 up	0.00	69.97	SSCRP
104182	11.47	0.06	10.30	0.04	8.47	0.34	2.242 down	7.991 down	0.00	94.62	Zn2Cys6 transcriptional regulator
104200	6.26	0.08	8.24	0.32	8.67	0.47	3.939 up	5.335 up	0.01	26.30	unknown protein. F-box
104201	2.98	0.09	2.90	0.04	4.36	0.20	1.064 down	2.585 up	0.00	80.18	unknown protein
104206	3.67	0.03	3.95	0.31	4.69	0.21	1.211 up	2.035 up	0.01	18.00	SSCRP
104209	3.76	0.11	3.96	0.16	5.57	0.32	1.146 up	3.511 up	0.00	43.07	unknown protein
104211	8.37	0.09	4.41	0.21	12.97	0.28	15.509 down	24.344 up	0.00	883.18	FKBP-type peptidyl-prolyl cis-trans isomerase
104215	8.09	0.05	7.55	0.04	6.38	0.07	1.454 down	3.289 down	0.00	537.69	unknown protein
104227	4.10	0.27	3.94	0.25	8.49	1.38	1.120 down	21.005 up	0.01	17.09	SSCRP
104228	2.54	0.26	2.48	0.08	3.98	0.16	1.048 down	2.701 up	0.00	71.49	unique protein
104231	12.70	0.11	12.57	0.08	11.63	0.10	1.087 down	2.094 down	0.00	109.37	unknown protein
104260	5.98	0.21	6.37	0.06	7.87	0.28	1.303 up	3.683 up	0.00	51.47	unknown protein
104261	6.25	0.13	6.65	0.23	8.83	0.41	1.323 up	6.012 up	0.00	50.30	GCN5-related N-acetyltransferase
104276	9.57	0.08	9.91	0.22	8.16	0.20	1.269 up	2.652 down	0.00	72.16	unknown protein
104277	13.33	0.32	12.37	0.05	11.80	0.47	1.942 down	2.882 down	0.03	10.27	cell wall protein. distantly related to A. niger CwpA.
104286	3.33	0.16	3.42	0.10	4.84	0.13	1.065 up	2.845 up	0.00	121.82	unique protein
104289	4.34	0.06	4.27	0.07	5.67	0.14	1.048 down	2.528 up	0.00	145.74	unique protein
104292	7.50	0.09	8.35	0.01	8.77	0.42	1.803 up	2.411 up	0.03	10.22	mating type pheromone precursor. alpha type
104293	5.98	0.54	6.49	0.48	8.14	0.21	1.430 up	4.472 up	0.01	28.34	HFB5
104294	4.48	0.47	4.76	0.24	6.93	0.20	1.215 up	5.465 up	0.00	67.34	unknown protein
104295	7.24	0.16	4.88	0.32	9.76	0.67	5.144 down	5.720 up	0.00	56.23	unknown protein
104304	10.28	0.15	9.01	0.06	8.34	0.25	2.406 down	3.822 down	0.00	57.79	unknown protein
104334	13.09	0.07	12.88	0.07	12.04	0.32	1.156 down	2.076 down	0.02	14.20	unknown protein
104335	5.40	0.42	5.73	0.00	6.56	0.15	1.256 up	2.224 up	0.01	21.62	unknown protein

104336	3.54	0.33	3.13	0.09	5.14	0.15	1.324 down	3.040 up	0.00	91.88	unique protein
104351	6.35	0.01	7.63	0.05	7.87	0.29	2.431 up	2.871 up	0.00	30.81	unique protein
104353	4.64	0.02	5.36	0.33	5.78	0.29	1.651 up	2.210 up	0.02	11.91	unknown protein. contains F-box
104354	3.27	0.05	3.34	0.31	4.70	0.27	1.047 up	2.685 up	0.00	30.58	SSCRP
104368	5.69	0.50	5.89	0.28	7.64	0.40	1.153 up	3.884 up	0.01	21.47	unknown protein
104369	3.78	0.12	3.70	0.22	5.23	0.15	1.052 down	2.744 up	0.00	85.25	unknown protein
104373	2.93	0.12	3.22	0.09	4.96	0.20	1.217 up	4.060 up	0.00	124.78	MRSP1/expansin-like
104377	11.82	0.02	11.56	0.05	9.53	0.31	1.198 down	4.890 down	0.00	79.65	unknown protein
104380	8.36	0.13	7.27	0.00	5.84	0.30	2.130 down	5.726 down	0.00	79.63	Zn2Cys6 transcriptional regulator
104390	9.31	0.04	11.22	0.31	7.95	0.26	3.773 up	2.563 down	0.00	121.61	glutathione S-transferase
104395	10.21	0.00	10.35	0.03	8.77	0.27	1.098 up	2.721 down	0.00	51.16	unknown protein
104399	2.50	0.00	2.70	0.05	3.93	0.06	1.149 up	2.696 up	0.00	634.28	unique protein
104419	3.89	0.25	3.77	0.37	5.94	0.42	1.090 down	4.138 up	0.00	30.44	unique protein. secreted
104421	2.93	0.07	3.22	0.17	4.97	0.10	1.220 up	4.106 up	0.00	270.16	unknown protein
104422	3.22	0.13	2.73	0.10	6.41	0.62	1.407 down	9.137 up	0.00	50.18	unknown protein
104423	5.10	0.35	4.38	0.09	7.12	0.35	1.648 down	4.053 up	0.00	58.58	MRSP1/expansin-like
104437	11.49	0.15	13.25	0.05	12.81	0.11	3.382 up	2.494 up	0.00	140.96	unknown protein
104490	9.80	0.14	10.21	0.24	11.54	0.32	1.325 up	3.345 up	0.00	31.37	v-SNARE Gos1. Golgi transport
104501	2.41	0.11	2.56	0.06	4.10	0.14	1.112 up	3.236 up	0.00	166.92	unique protein
104511	8.20	0.03	8.12	0.06	9.36	0.23	1.060 down	2.225 up	0.00	43.91	unique protein
104513	10.62	0.07	10.37	0.12	9.52	0.17	1.188 down	2.135 down	0.00	46.75	Zn2Cys6 transcriptional regulator
104533	2.45	0.10	2.41	0.07	4.12	0.11	1.025 down	3.190 up	0.00	293.98	unique protein
104556	5.79	0.15	6.08	0.09	9.39	0.54	1.223 up	12.129 up	0.00	67.35	unknown protein
104557	10.32	0.08	7.52	0.14	8.49	0.06	6.959 down	3.565 down	0.00	553.15	short-chain dehydrogenase/reductase
104576	2.20	0.07	2.23	0.08	3.67	0.03	1.023 up	2.780 up	0.00	676.14	unique protein
104577	3.77	0.20	3.91	0.06	5.19	0.07	1.095 up	2.664 up	0.00	155.81	unique protein
104585	6.26	0.11	6.14	0.02	7.45	0.38	1.082 down	2.283 up	0.01	17.25	unknown protein
104592	3.89	0.30	3.76	0.34	5.36	0.15	1.093 down	2.779 up	0.00	43.21	unknown protein
104593	6.60	0.13	5.05	0.25	5.06	0.11	2.936 down	2.909 down	0.00	77.80	unknown protein with ankyrin. Leu-zipper and WD40
104595	10.68	0.08	10.86	0.06	11.81	0.36	1.134 up	2.185 up	0.02	14.04	Ribosomal protein L7Ae/L30e/S12e/Gadd45. Homologue of yeast SNU13.

104599	10.45	0.08	8.79	0.13	8.00	0.22	3.147 down	5.473 down	0.00	117.20	Mandelate racemase/muconate lactonizing enzyme
104601	8.23	0.13	8.36	0.08	7.09	0.38	1.094 up	2.201 down	0.01	15.95	unknown protein
104617	12.45	0.04	12.17	0.13	11.03	0.14	1.218 down	2.670 down	0.00	105.00	
104695	4.37	0.05	3.59	0.32	5.53	0.36	1.708 down	2.243 up	0.01	27.90	unique protein
104715	2.47	0.16	2.46	0.15	4.01	0.07	1.005 down	2.905 up	0.00	192.79	unique protein
104716	3.01	0.11	3.12	0.38	5.01	0.10	1.076 up	3.999 up	0.00	100.42	unique protein
104741	11.06	0.09	10.13	0.04	8.93	0.25	1.902 down	4.389 down	0.00	78.30	unknown protein
104744	11.34	0.11	11.49	0.15	12.53	0.41	1.111 up	2.282 up	0.02	11.59	cytochrome c oxidase assembly protein COX19. putative
104762	7.57	0.09	8.36	0.14	8.60	0.11	1.727 up	2.041 up	0.00	53.53	unknown protein
104795	7.58	0.18	8.07	0.10	9.28	0.12	1.407 up	3.245 up	0.00	128.94	unique protein
104820	9.48	0.04	9.02	0.03	8.43	0.41	1.371 down	2.073 down	0.04	7.86	unknown protein
104842	11.37	0.23	11.36	0.21	10.20	0.28	1.005 down	2.241 down	0.01	19.95	transthyretin-like protein
104911	11.79	0.01	11.65	0.00	10.72	0.14	1.099 down	2.097 down	0.00	87.85	unknown protein
104923	3.28	0.18	3.38	0.00	4.91	0.22	1.072 up	3.090 up	0.00	69.98	unique protein
104925	8.63	0.00	9.43	0.03	7.14	0.35	1.743 up	2.802 down	0.00	52.00	unknown protein
104927	7.68	0.16	9.46	0.11	9.73	0.39	3.427 up	4.123 up	0.00	29.55	unique protein
104968	12.02	0.11	11.73	0.07	10.55	0.10	1.229 down	2.768 down	0.00	188.37	unknown protein
104972	13.65	0.12	13.24	0.16	12.59	0.29	1.330 down	2.083 down	0.02	13.85	glutathione synthase
105003	11.25	0.04	8.94	0.16	9.21	0.28	4.968 down	4.113 down	0.00	66.87	unknown protein
105027	5.23	0.27	5.36	0.11	6.39	0.15	1.093 up	2.231 up	0.00	38.47	unknown protein
105106	12.96	0.02	13.28	0.03	10.36	0.42	1.255 up	6.057 down	0.00	73.35	unique protein
105133	5.90	0.02	6.45	0.08	7.06	0.23	1.460 up	2.238 up	0.01	28.04	unknown protein
105143	9.41	0.20	9.81	0.08	11.95	0.49	1.323 up	5.834 up	0.00	36.53	unique protein
105154	4.59	0.03	4.59	0.02	6.05	0.53	1.002 down	2.745 up	0.02	12.46	unknown protein
105155	4.21	0.24	4.63	0.33	6.28	0.16	1.334 up	4.191 up	0.00	72.04	unknown protein
105156	3.52	0.40	3.57	0.11	6.94	0.73	1.034 up	10.673 up	0.00	32.41	unique protein
105157	3.07	0.02	3.09	0.08	5.23	0.10	1.011 up	4.445 up	0.00	651.12	unknown protein
105158	7.00	0.06	7.48	0.20	5.78	0.10	1.390 up	2.330 down	0.00	155.15	unknown protein
105167	3.43	0.21	3.59	0.28	4.93	0.09	1.123 up	2.845 up	0.00	68.27	unique protein
105171	3.65	0.23	3.68	0.15	5.81	0.16	1.023 up	4.463 up	0.00	145.59	unknown protein

105173	2.62	0.15	2.73	0.24	4.38	0.21	1.076 up	3.365 up	0.00	69.68	unknown protein
105187	6.56	0.15	6.38	0.02	8.52	0.36	1.130 down	3.887 up	0.00	50.55	unknown protein
105206	4.55	0.31	5.03	0.00	6.34	0.36	1.399 up	3.470 up	0.01	25.91	unique protein
105220	6.40	0.39	3.96	0.35	4.59	0.09	5.424 down	3.525 down	0.00	55.86	unknown protein
105221	3.70	0.31	2.98	0.41	4.72	0.05	1.650 down	2.028 up	0.00	40.61	unknown protein
105222	3.84	0.09	3.95	0.15	5.71	0.34	1.081 up	3.648 up	0.00	43.69	unique protein
105223	8.62	0.06	8.38	0.26	6.42	0.24	1.179 down	4.591 down	0.00	87.86	unknown protein
105233	2.86	0.01	2.69	0.06	4.34	0.03	1.129 down	2.785 up	0.00	2054.93	unique protein
105239	11.56	0.15	11.34	0.13	9.98	0.14	1.161 down	2.977 down	0.00	110.89	Zn2Cys6 transcriptional regulator
105246	3.77	0.13	4.35	0.31	5.70	0.18	1.488 up	3.805 up	0.00	67.49	GH23 exo-ß-1.3-glucanase. distantly related
105247	3.19	0.23	3.44	0.17	5.19	0.06	1.196 up	4.006 up	0.00	188.44	dipeptidyl peptidase 5
105251	9.70	0.07	9.46	0.06	8.26	0.30	1.185 down	2.724 down	0.00	31.24	unknown protein
105255	9.46	0.15	8.77	0.14	7.05	0.34	1.609 down	5.298 down	0.00	57.77	Zn2Cys6 transcriptional regulator
105260	11.56	0.08	10.04	0.26	7.90	0.47	2.886 down	12.720 down	0.00	64.07	MFS permease
105263	11.69	0.19	11.24	0.00	10.00	0.12	1.364 down	3.222 down	0.00	146.52	Zn2Cys6 transcriptional regulator
105269	9.61	0.10	8.88	0.04	8.60	0.19	1.661 down	2.011 down	0.00	28.97	Zn2Cys6 transcriptional regulator
105279	4.80	0.34	3.90	0.10	5.98	0.71	1.861 down	2.277 up	0.03	9.49	Leucine aminopeptidase 1
105286	4.55	0.12	4.22	0.23	5.90	0.14	1.261 down	2.546 up	0.00	90.36	unknown protein
105290	5.58	0.15	4.88	0.43	6.81	0.53	1.631 down	2.336 up	0.02	13.16	unknown protein
105307	9.30	0.01	10.03	0.25	10.90	0.59	1.649 up	3.013 up	0.04	7.90	40s mitochodrial ribosome subunit protein Mrp17
105311	10.60	0.13	11.26	0.21	12.63	0.30	1.576 up	4.079 up	0.00	47.62	SSCRP
105330	5.23	0.38	4.48	0.06	7.23	0.47	1.687 down	3.985 up	0.00	36.96	unknown protein
105336	5.04	0.35	5.32	0.04	6.47	0.33	1.210 up	2.696 up	0.01	19.01	unknown protein
105338	3.00	0.24	3.26	0.21	5.23	0.28	1.199 up	4.693 up	0.00	66.45	unknown protein
105363	11.53	0.05	9.03	0.19	8.69	0.56	5.675 down	7.165 down	0.00	29.16	aldolase/citrate lyase family protein
105381	10.01	0.22	10.41	0.22	11.22	0.32	1.324 up	2.320 up	0.02	13.97	unknown protein
105383	3.58	0.00	3.80	0.44	5.58	0.26	1.162 up	3.994 up	0.00	44.75	unknown protein
105385	4.14	0.04	4.10	0.34	5.44	0.18	1.030 down	2.463 up	0.00	40.31	unknown protein
105386	12.27	0.12	12.15	0.11	10.81	0.23	1.081 down	2.736 down	0.00	54.56	unknown protein
105391	2.84	0.02	3.09	0.03	4.98	0.12	1.189 up	4.408 up	0.00	435.83	unknown protein

105393	6.37	0.06	5.78	0.08	7.87	0.48	1.500 down	2.832 up	0.01	23.89	unknown protein
105405	3.12	0.07	2.89	0.09	4.70	0.29	1.174 down	2.990 up	0.00	55.05	unique protein
105444	5.47	0.21	4.84	0.21	6.88	0.30	1.549 down	2.658 up	0.00	44.05	SSCRP
105445	2.81	0.05	2.88	0.02	4.45	0.13	1.048 up	3.096 up	0.00	250.64	unknown protein. only in Neurospora and A. fumigatus
105447	4.99	0.20	4.89	0.19	7.02	0.33	1.074 down	4.082 up	0.00	53.72	unknown protein
105448	3.29	0.22	3.57	0.27	5.80	0.40	1.215 up	5.686 up	0.00	47.65	CBM 13
105449	3.27	0.16	3.30	0.11	5.16	0.29	1.020 up	3.699 up	0.00	61.32	Cyclin C-dependent kinase CDK8
105455	8.79	0.04	7.04	0.24	7.48	0.37	3.385 down	2.493 down	0.01	18.56	unknown protein
105457	3.13	0.02	3.38	0.12	4.56	0.11	1.195 up	2.697 up	0.00	175.60	unknown protein
105467	3.40	0.29	3.47	0.16	5.23	0.10	1.053 up	3.546 up	0.00	115.97	unknown protein
105488	6.17	0.07	6.39	0.23	7.24	0.05	1.162 up	2.099 up	0.00	75.24	unknown protein
105514	11.01	0.12	6.45	0.03	9.52	0.66	23.554 down	2.801 down	0.00	41.87	unique protein
105515	11.50	0.17	13.05	0.11	13.90	0.13	2.915 up	5.259 up	0.00	216.53	unique protein
105518	11.91	0.04	10.56	0.10	9.27	0.16	2.552 down	6.245 down	0.00	260.59	K+ channel
105532	6.28	0.04	5.52	0.13	7.50	0.59	1.695 down	2.322 up	0.02	13.17	unique protein
105537	7.11	0.10	7.18	0.02	6.03	0.16	1.051 up	2.108 down	0.00	72.40	unknown protein
105540	5.25	0.28	5.20	0.24	6.50	0.40	1.033 down	2.368 up	0.02	12.82	unique protein
105553	6.67	0.57	7.12	0.27	9.76	0.69	1.363 up	8.518 up	0.01	22.60	unique protein
105556	3.59	0.01	4.13	0.23	5.67	0.14	1.445 up	4.205 up	0.00	157.22	unique protein
105597	11.82	0.04	11.96	0.12	10.43	0.26	1.101 up	2.615 down	0.00	50.62	unique protein
105643	12.01	0.07	11.83	0.01	10.59	0.20	1.135 down	2.688 down	0.00	70.93	myb transcriptional regulator
105652	3.74	0.15	4.09	0.06	5.51	0.06	1.273 up	3.408 up	0.00	339.50	unknown protein
105676	8.69	0.02	9.86	0.11	9.89	0.18	2.252 up	2.303 up	0.00	50.14	DNA replication licensing factor mcm7
105707	2.78	0.54	2.88	0.38	4.44	0.20	1.072 up	3.169 up	0.01	23.67	unknown protein
105718	11.10	0.22	10.12	0.09	8.21	0.29	1.979 down	7.431 down	0.00	104.61	unknown protein
105722	10.76	0.00	6.64	0.22	8.75	0.51	17.360 down	4.027 down	0.00	51.85	unique protein
105729	3.13	0.38	3.15	0.31	4.60	0.06	1.012 up	2.767 up	0.00	41.75	unknown protein
105752	2.80	0.14	2.82	0.04	6.30	1.06	1.018 up	11.330 up	0.01	18.05	C4-dicarboxylate transporter/malic acid transport protein
105763	10.20	0.24	10.93	0.14	8.12	0.26	1.649 up	4.233 down	0.00	114.04	HFBs
105765	11.39	0.11	11.41	0.13	9.36	0.36	1.009 up	4.102 down	0.00	51.05	unknown protein

105768	4.61	0.12	4.54	0.18	5.67	0.08	1.049 down	2.085 up	0.00	88.18	Cytochrome P450
105775	9.52	0.10	8.18	0.05	8.39	0.39	2.536 down	2.195 down	0.02	11.84	unknown protein
105784	8.10	0.13	7.24	0.19	6.61	0.27	1.815 down	2.811 down	0.01	28.15	Zn2Cys6 transcriptional regulator
105785	4.58	0.09	5.09	0.04	6.72	0.19	1.423 up	4.402 up	0.00	151.49	unknown protein
105798	4.02	0.15	4.18	0.38	5.43	0.10	1.120 up	2.659 up	0.00	46.35	MFS permease
105804	3.59	0.31	3.33	0.43	5.34	0.20	1.193 down	3.352 up	0.00	44.52	PKS
105805	3.32	0.07	3.67	0.42	5.53	0.17	1.272 up	4.632 up	0.00	78.10	Zn2Cys6 transcriptional regulator
105806	3.11	0.22	2.58	0.08	5.03	0.13	1.436 down	3.782 up	0.00	229.23	unknown protein
105820	2.83	0.06	3.14	0.26	4.24	0.16	1.237 up	2.642 up	0.00	54.42	unknown protein
105823	9.62	0.02	4.85	0.30	6.76	0.45	27.139 down	7.263 down	0.00	81.61	FAD binding domain protein
105832	12.66	0.10	12.18	0.10	11.38	0.23	1.403 down	2.434 down	0.00	32.27	phosphoribosylaminoimidazole carbounknown proteinlase
105834	9.00	0.02	9.15	0.28	7.39	0.24	1.110 up	3.049 down	0.00	56.63	Zn2Cys6 transcriptional regulator
105838	4.36	0.28	4.15	0.23	5.51	0.08	1.160 down	2.210 up	0.00	52.87	glyoxalase
105840	9.19	0.00	5.93	0.11	10.24	0.33	9.559 down	2.071 up	0.00	187.59	unknown protein. 2 TMs
105849	9.65	0.05	9.14	0.13	7.46	0.21	1.424 down	4.587 down	0.00	129.79	Zn2Cys6 transcriptional regulator
105851	7.73	0.00	7.43	0.17	6.49	0.48	1.232 down	2.375 down	0.03	8.81	GMC oxidoreductase family protein
105863	10.84	0.07	11.32	0.14	9.63	0.09	1.395 up	2.300 down	0.00	216.55	unknown protein
105866	11.94	0.03	11.76	0.06	10.55	0.12	1.135 down	2.615 down	0.00	177.20	unique protein
105867	3.25	0.38	3.33	0.09	5.25	0.30	1.058 up	3.986 up	0.00	44.82	unique protein
105869	5.76	0.03	6.38	0.17	7.64	0.27	1.536 up	3.676 up	0.00	52.64	HFB6
105874	7.15	0.06	7.22	0.04	5.75	0.18	1.051 up	2.636 down	0.00	105.88	FAD binding domain-containing protein
105884	3.51	0.07	3.67	0.03	5.19	0.19	1.120 up	3.214 up	0.00	109.51	short chain dehydrogenase/reductase
105888	5.67	0.03	6.55	0.30	7.75	0.30	1.847 up	4.222 up	0.00	42.05	short chain dehydrogenase/reductase
105894	5.39	0.45	5.59	0.22	8.56	0.58	1.149 up	9.027 up	0.00	37.04	unknown protein
105912	4.14	0.08	4.02	0.23	5.37	0.24	1.082 down	2.348 up	0.00	35.79	unknown protein
105977	4.17	0.04	4.64	0.09	5.27	0.19	1.379 up	2.139 up	0.00	35.64	unknown secreted protein. 3 TM
105978	9.14	0.04	9.88	0.14	8.14	0.24	1.676 up	2.003 down	0.00	57.74	ARO8. Transcriptional regulators containing a DNA-binding HTH domain and an amino
105983	3.44	0.09	3.56	0.39	4.87	0.11	1.085 up	2.706 up	0.00	48.02	unknown protein
105989	4.03	0.11	5.44	0.29	5.40	0.17	2.653 up	2.578 up	0.00	40.65	Zn2Cys6 transcriptional regulator
106009	11.46	0.12	11.28	0.18	10.42	0.23	1.133 down	2.063 down	0.01	23.03	Zn2Cys6 transcriptional regulator

106018	10.86	0.17	11.93	0.00	12.07	0.05	2.107 up	2.312 up	0.00	133.36	unknown protein
106024	7.73	0.25	8.24	0.16	9.68	0.63	1.429 up	3.875 up	0.02	11.80	unknown protein
106029	11.58	0.04	10.58	0.07	9.37	0.16	1.994 down	4.616 down	0.00	203.95	MFS permease
106041	3.37	0.00	3.65	0.14	5.00	0.16	1.216 up	3.105 up	0.00	120.31	unique protein
106043	10.09	0.06	8.15	0.07	8.00	0.25	3.842 down	4.265 down	0.00	79.56	unknown protein. GFA-domain
106067	9.56	0.72	8.94	0.11	11.16	0.22	1.540 down	3.028 up	0.01	28.11	Chaperonin Cpn10
106082	11.93	0.00	11.49	0.06	10.44	0.15	1.358 down	2.813 down	0.00	126.56	Epl1/Sm1
106089	8.48	0.01	7.61	0.14	6.13	0.18	1.823 down	5.089 down	0.00	169.85	unique protein
106107	5.31	0.09	5.83	0.23	6.65	0.10	1.429 up	2.523 up	0.00	68.42	unknown protein
106116	5.08	0.17	6.23	0.01	6.81	0.67	2.227 up	3.324 up	0.05	7.36	unknown protein
106118	10.89	0.04	10.61	0.16	9.58	0.13	1.221 down	2.479 down	0.00	92.34	Inorganic phosphate transporter
106120	8.56	0.06	12.87	0.03	12.32	0.33	19.812 up	13.457 up	0.00	181.01	esterase/lipase
106129	11.55	0.05	10.92	0.11	9.73	0.38	1.554 down	3.547 down	0.01	28.27	unknown protein. only present in ascomycota
106130	13.95	0.02	13.52	0.08	12.44	0.12	1.346 down	2.843 down	0.00	187.86	unknown protein
106138	8.36	0.00	7.30	0.01	6.61	0.32	2.081 down	3.340 down	0.00	32.81	SNF2 family DNA-dependent ATPase
106151	3.53	0.30	3.71	0.06	6.47	0.27	1.136 up	7.677 up	0.00	127.89	unknown protein
106152	5.09	0.02	5.67	0.16	6.15	0.19	1.488 up	2.079 up	0.01	27.98	unknown protein
106154	6.09	0.24	6.55	0.11	4.66	0.04	1.368 up	2.699 down	0.00	189.85	unknown protein
106164	10.81	0.00	8.06	0.07	12.21	0.13	6.740 down	2.623 up	0.00	962.65	short chain dehydrogenase/reductase
106168	4.76	0.12	5.17	0.04	6.53	0.33	1.325 up	3.414 up	0.00	37.64	SSCRP
106171	10.23	0.14	8.73	0.26	11.92	1.06	2.821 down	3.232 up	0.03	10.31	HET protein
106181	11.39	0.07	11.73	0.04	7.85	0.72	1.265 up	11.629 down	0.00	44.19	unique protein
106219	7.71	0.26	8.41	0.06	9.30	0.21	1.617 up	3.010 up	0.00	42.88	unknown protein
106223	12.86	0.05	11.45	0.20	10.26	0.26	2.658 down	6.040 down	0.00	92.59	unique protein
106234	12.55	0.08	11.75	0.16	11.53	0.18	1.739 down	2.030 down	0.01	28.00	integral membrane protein comprising an SPX and an EXS domain. therefore most pro
106242	9.79	0.12	9.28	0.11	8.52	0.19	1.424 down	2.404 down	0.00	43.38	unique protein
106245	7.80	0.08	7.06	0.04	5.75	0.36	1.665 down	4.117 down	0.00	38.52	catalase
106248	11.09	0.04	11.85	0.01	12.91	0.14	1.696 up	3.534 up	0.00	192.30	monosaccharide transporter (galactose permease ?)
106267	4.25	0.03	4.36	0.24	5.85	0.21	1.081 up	3.031 up	0.00	61.55	unique protein
106270	10.85	0.14	5.32	0.53	8.08	1.82	46.214 down	6.817 down	0.05	7.44	unknown protein

106272	8.37	0.12	7.82	0.01	7.00	0.30	1.455 down	2.574 down	0.01	24.28	PKS
106276	12.71	0.11	12.59	0.06	11.06	0.20	1.087 down	3.132 down	0.00	93.44	initiator tRNA phosphoribosyl transferase.
106297	10.92	0.07	11.40	0.02	9.89	0.26	1.393 up	2.036 down	0.00	39.86	Amino acid transporters
106314	8.84	0.01	9.22	0.03	6.11	0.20	1.306 up	6.595 down	0.00	351.56	Ankyrin
106315	7.19	0.33	8.37	0.04	9.20	0.31	2.271 up	4.033 up	0.00	33.69	serine protease
106356	12.26	0.12	11.94	0.05	11.12	0.33	1.240 down	2.196 down	0.01	14.88	unknown protein
106371	6.69	0.10	3.58	0.04	5.47	0.06	8.617 down	2.322 down	0.00	1022.21	SSCRP
106391	12.95	0.01	12.86	0.01	11.14	0.36	1.062 down	3.506 down	0.00	40.27	unknown protein
106405	5.90	0.06	6.88	0.01	7.56	0.42	1.978 up	3.158 up	0.01	17.68	extracellular lipase-like protein
106437	11.06	0.11	10.62	0.15	9.69	0.24	1.356 down	2.581 down	0.00	34.56	unknown protein
106445	5.01	0.40	4.84	0.38	7.67	0.40	1.126 down	6.337 up	0.00	48.88	unique protein
106452	11.33	0.21	9.63	0.00	9.12	0.25	3.258 down	4.639 down	0.00	74.10	unknown protein
106453	6.35	0.59	6.53	0.57	8.25	0.59	1.138 up	3.756 up	0.03	9.80	SSCRP
106470	2.42	0.11	2.36	0.22	3.61	0.14	1.047 down	2.278 up	0.00	62.53	unique protein
106479	2.63	0.15	2.84	0.27	4.45	0.10	1.156 up	3.527 up	0.00	118.28	unknown protein
106480	4.55	0.06	5.77	0.29	7.32	0.74	2.327 up	6.820 up	0.01	15.66	flavoprotein monooxygenases
106486	2.87	0.02	2.98	0.14	4.67	0.05	1.074 up	3.471 up	0.00	545.63	unique protein
106488	8.71	0.12	8.15	0.31	6.86	0.51	1.475 down	3.615 down	0.01	14.77	unknown protein
106490	4.39	0.13	4.64	0.21	5.94	0.31	1.188 up	2.925 up	0.00	29.69	unknown protein. 3 TM. only in Neurospora and Chaetomium
106491	8.56	0.06	6.98	0.10	6.22	0.28	2.986 down	5.048 down	0.00	74.76	unknown protein
106492	5.08	0.27	5.19	0.30	6.83	0.45	1.077 up	3.370 up	0.01	19.04	SSCRP
106493	2.28	0.02	2.40	0.05	3.86	0.16	1.085 up	2.974 up	0.00	152.76	unknown protein
106499	3.27	0.08	2.81	0.24	4.55	0.08	1.377 down	2.433 up	0.00	141.90	unknown protein
106538	9.51	0.02	12.40	0.21	12.85	0.42	7.440 up	10.140 up	0.00	67.85	HFB4
106556	7.18	0.20	6.64	0.04	8.85	0.90	1.458 down	3.185 up	0.04	8.00	unknown protein
106564	6.10	0.32	6.15	0.34	7.41	0.21	1.032 up	2.475 up	0.01	23.69	unique protein
106583	2.60	0.02	2.44	0.10	4.29	0.04	1.121 down	3.225 up	0.00	996.83	unique protein
106604	6.83	0.55	6.96	0.16	8.33	0.41	1.092 up	2.819 up	0.02	12.55	unique protein
106654	8.93	0.26	8.49	0.11	6.84	0.35	1.352 down	4.265 down	0.00	40.21	Zn2Cys6 transcriptional regulator
106657	11.17	0.24	10.54	0.00	9.79	0.10	1.546 down	2.603 down	0.00	74.92	Zn2Cys6 transcriptional regulator

106659	8.97	0.12	8.56	0.01	6.60	0.58	1.329 down	5.170 down	0.01	23.05	unknown protein
106661	4.37	0.17	3.58	0.03	5.90	0.21	1.727 down	2.889 up	0.00	118.76	aspartyl protease
106662	5.03	0.07	5.09	0.64	6.24	0.11	1.038 up	2.307 up	0.01	15.36	SSCRP
106677	9.36	0.11	7.74	0.13	7.44	0.07	3.067 down	3.773 down	0.00	287.76	Zn2Cys6 transcriptional regulator
106684	2.94	0.32	3.62	0.02	4.76	0.17	1.611 up	3.536 up	0.00	63.47	unknown protein
106686	9.25	0.02	8.06	0.14	11.51	0.74	2.277 down	4.800 up	0.01	26.98	unknown protein
106695	10.79	0.12	7.71	0.18	9.37	0.10	8.454 down	2.670 down	0.00	311.43	Sulfite oxidase. molybdopterin-binding component
106697	7.14	0.15	6.03	0.13	5.51	0.43	2.150 down	3.088 down	0.01	14.93	esterase/lipase. HGT
106706	11.27	0.05	10.77	0.01	8.28	0.42	1.414 down	7.936 down	0.00	72.47	Zn2Cys6 transcriptional regulator
106798	9.82	0.05	11.74	0.29	12.06	0.27	3.762 up	4.706 up	0.00	56.50	GCN5-N-acetyltransferase
106801	10.74	0.03	10.75	0.05	9.74	0.22	1.007 up	2.007 down	0.00	34.47	unknown protein
106812	2.68	0.18	2.82	0.03	4.99	0.27	1.106 up	4.957 up	0.00	97.91	unique protein
106828	10.98	0.11	11.35	0.01	9.89	0.13	1.297 up	2.124 down	0.00	136.12	unknown protein
106869	4.56	0.06	5.35	0.22	6.38	0.50	1.737 up	3.537 up	0.01	14.82	unknown protein
106897	4.65	0.01	5.10	0.19	5.90	0.31	1.367 up	2.386 up	0.01	17.74	unique protein
106928	4.13	0.07	4.17	0.04	6.26	0.35	1.027 up	4.367 up	0.00	60.65	Na/K ATPase alpha 1 subunit.
106939	5.26	0.21	7.45	0.22	6.60	0.28	4.556 up	2.534 up	0.00	36.92	protein kinase
106947	3.33	0.30	4.20	0.18	5.20	0.13	1.833 up	3.657 up	0.00	70.42	unique protein
106958	11.60	0.12	11.76	0.09	10.14	0.25	1.115 up	2.764 down	0.00	57.33	unique protein
106960	3.53	0.51	3.38	0.29	5.62	0.28	1.109 down	4.269 up	0.00	41.17	unknown protein
106998	4.85	0.09	4.72	0.14	6.17	0.54	1.098 down	2.488 up	0.02	10.78	unknown protein
107002	10.27	0.01	9.74	0.07	8.48	0.18	1.448 down	3.478 down	0.00	124.97	unknown protein
107003	8.80	0.08	7.99	0.06	10.86	0.59	1.757 down	4.165 up	0.00	30.54	unknown protein
107007	3.42	0.11	3.28	0.26	4.61	0.18	1.101 down	2.283 up	0.00	44.89	SSCRP
107008	2.95	0.12	3.23	0.14	5.25	0.08	1.208 up	4.930 up	0.00	448.46	unknown protein
107031	8.06	0.13	7.73	0.17	9.41	0.48	1.255 down	2.560 up	0.01	15.79	unique protein
107037	10.10	0.11	11.49	0.15	12.07	0.42	2.634 up	3.940 up	0.01	22.97	unknown protein
107042	6.99	0.12	8.06	0.08	8.07	0.37	2.094 up	2.118 up	0.03	10.08	PTH11 GPCR
107055	11.41	0.05	10.75	0.02	8.69	0.51	1.572 down	6.580 down	0.00	37.76	unknown protein
107070	2.78	0.08	2.73	0.06	4.19	0.06	1.036 down	2.653 up	0.00	465.97	unique protein

107071	3.19	0.01	3.69	0.18	5.10	0.29	1.412 up	3.767 up	0.00	52.01	HET-domain protein. unknown unknown
107107	5.89	0.12	5.64	0.00	7.06	0.32	1.193 down	2.243 up	0.01	26.35	unknown protein
107111	11.88	0.10	11.55	0.12	10.26	0.14	1.258 down	3.064 down	0.00	135.66	unknown protein
107112	5.79	0.14	6.68	0.26	7.11	0.12	1.851 up	2.498 up	0.00	43.82	unique protein
107123	8.55	0.08	9.49	0.05	9.72	0.13	1.913 up	2.239 up	0.00	76.03	mitochondrial import inner membrane translocase subunit tim16
107131	9.64	0.01	10.36	0.31	11.14	0.19	1.657 up	2.838 up	0.00	39.53	unknown protein
107137	13.80	0.10	13.60	0.03	12.66	0.16	1.151 down	2.205 down	0.00	65.25	sterol desaturase family
107142	4.26	0.05	5.25	0.25	5.70	0.16	1.980 up	2.709 up	0.00	48.62	neutral protease 2
107146	9.24	0.11	9.25	0.10	10.48	0.45	1.009 up	2.363 up	0.02	12.33	unknown protein
107194	3.92	0.30	4.96	0.23	5.50	0.24	2.057 up	2.995 up	0.01	26.73	unique protein
107202	10.12	0.10	8.17	0.02	8.85	0.13	3.857 down	2.407 down	0.00	161.99	unknown protein
107207	4.91	0.07	5.33	0.10	6.09	0.09	1.338 up	2.270 up	0.00	130.08	HMG box-containing protein
107208	2.41	0.02	2.49	0.04	3.82	0.03	1.057 up	2.665 up	0.00	2343.52	unique protein
107217	6.96	0.02	4.25	0.00	5.50	0.19	6.515 down	2.752 down	0.00	166.57	unknown protein
107239	9.94	0.05	11.34	0.14	11.05	0.13	2.637 up	2.160 up	0.00	76.09	microtubule protein BIM1. possibly involved in mating. karyogamy or meiosis
107258	12.08	0.11	12.20	0.04	10.86	0.19	1.087 up	2.331 down	0.00	66.64	1-acyl-sn-gylcerol-3-phosphate acyltransferase (Lysophosphatidic acid acyltransferase
107260	12.14	0.07	12.12	0.06	11.04	0.12	1.017 down	2.150 down	0.00	119.87	unknown protein
107268	12.06	0.12	11.68	0.04	10.96	0.09	1.302 down	2.148 down	0.00	120.56	Esterase/lipase/thioesterase
107294	12.26	0.01	12.02	0.21	10.90	0.34	1.177 down	2.568 down	0.01	20.22	RTA1-like protein. 7 TM. responds to xenobiotic stimuli
107297	10.92	0.10	9.33	0.12	7.45	0.17	3.011 down	11.076 down	0.00	368.20	unique protein
107306	6.29	0.07	6.55	0.02	5.27	0.18	1.201 up	2.031 down	0.00	68.00	unique protein
107329	4.80	0.15	4.36	0.54	6.80	0.22	1.361 down	3.984 up	0.00	55.51	methionine synthase
107340	14.37	0.03	13.30	0.11	11.84	0.48	2.102 down	5.791 down	0.00	32.32	unknown protein
107347	4.80	0.32	6.38	0.43	6.08	0.50	2.995 up	2.418 up	0.05	7.26	SSCRP
107349	3.43	0.11	3.75	0.04	4.75	0.21	1.248 up	2.496 up	0.00	47.81	unknown protein
107358	5.33	0.13	5.12	0.30	6.48	0.22	1.150 down	2.227 up	0.00	32.21	unique protein
107369	12.41	0.10	10.99	0.08	10.70	0.16	2.688 down	3.284 down	0.00	103.53	guanine nucleotide exchange factor-like protein
107386	11.23	0.09	11.28	0.16	12.71	0.40	1.032 up	2.794 up	0.01	20.43	unknown protein. only in Gibberella and Neurospora
107445	3.37	0.00	3.49	0.05	5.22	0.16	1.087 up	3.595 up	0.00	199.16	unknown protein
107463	10.53	0.13	9.45	0.12	9.49	0.24	2.112 down	2.059 down	0.01	20.27	exoribonuclease Dhp1

107464	7.23	0.00	7.47	0.09	10.75	0.31	1.179 up	11.473 up	0.00	194.77	unknown protein
107475	11.38	0.06	10.64	0.12	9.45	0.50	1.673 down	3.812 down	0.01	17.32	unknown protein
107483	9.31	0.13	9.36	0.04	7.83	0.14	1.037 up	2.775 down	0.00	145.80	unknown protein
107488	3.68	0.52	3.83	0.19	5.22	0.19	1.111 up	2.913 up	0.01	26.13	unknown protein
107494	12.60	0.02	9.59	0.10	10.48	0.14	8.068 down	4.350 down	0.00	371.71	unique protein
107495	11.95	0.05	7.25	0.16	10.94	0.16	25.930 down	2.009 down	0.00	603.83	unknown protein
107507	9.92	0.03	13.02	0.03	11.70	0.54	8.541 up	3.427 up	0.01	27.52	unknown protein
107513	5.11	0.16	5.96	0.11	6.14	0.32	1.801 up	2.031 up	0.03	10.26	unique protein
107524	9.58	0.03	9.51	0.02	8.08	0.33	1.048 down	2.835 down	0.00	33.04	unique protein
107526	5.17	0.11	5.15	0.48	6.39	0.11	1.014 down	2.315 up	0.01	26.89	short chain dehydrognease/reductase
107554	11.58	0.02	10.72	0.06	10.56	0.03	1.815 down	2.036 down	0.00	544.19	glutathione transferase
107564	9.34	0.04	10.36	0.05	10.44	0.29	2.034 up	2.146 up	0.01	16.56	Sin3-associated polypeptide Sap18
107595	3.84	0.14	3.94	0.04	5.32	0.29	1.072 up	2.779 up	0.00	37.87	unique protein
107601	11.55	0.24	11.33	0.09	12.74	0.34	1.169 down	2.275 up	0.01	20.73	unknown Zn-finger protein. Tim10/DPP type
107639	11.41	0.03	9.20	0.10	9.93	0.10	4.604 down	2.784 down	0.00	308.31	unknown protein
107641	5.52	0.02	5.94	0.19	11.06	0.08	1.342 up	46.831 up	0.00	2546.06	unique protein
107657	3.82	0.04	3.89	0.04	5.45	0.17	1.044 up	3.085 up	0.00	138.73	unique protein
107669	10.70	0.08	10.57	0.06	9.10	0.30	1.094 down	3.048 down	0.00	42.95	Dipeptidyl aminopeptidase
107670	10.98	0.02	10.99	0.13	8.80	0.21	1.009 up	4.515 down	0.00	154.43	unknown protein
107674	5.95	0.02	6.31	0.12	11.19	0.49	1.279 up	37.788 up	0.00	173.78	unique protein
107685	5.31	0.33	5.53	0.42	6.81	0.41	1.171 up	2.829 up	0.02	12.56	unique protein
107704	9.43	0.17	10.29	0.01	11.44	0.10	1.814 up	4.051 up	0.00	252.69	unknown protein. only in A. fumigatus
107717	3.27	0.21	3.26	0.02	5.12	0.28	1.004 down	3.605 up	0.00	61.26	SSCRP
107742	5.14	0.07	5.81	0.23	6.18	0.17	1.583 up	2.044 up	0.01	23.78	unique protein
107743	6.39	0.47	5.86	0.37	9.17	0.43	1.447 down	6.870 up	0.00	52.38	unique protein
107811	4.88	0.09	4.34	0.31	6.04	0.14	1.458 down	2.235 up	0.00	70.12	unknown protein
107814	3.07	0.13	3.65	0.03	4.67	0.14	1.489 up	3.026 up	0.00	129.78	unknown protein
107835	6.99	0.20	7.56	0.25	10.42	0.23	1.480 up	10.793 up	0.00	194.17	Arylacetamide deacetylase
107843	4.75	0.03	5.22	0.04	6.24	0.14	1.380 up	2.810 up	0.00	143.78	unique protein
107844	4.60	0.29	4.38	0.26	6.53	0.41	1.161 down	3.817 up	0.00	32.37	unknown protein. 3 TM. only in Gibberella and Chaetomium

107846	3.18	0.09	3.52	0.07	4.92	0.12	1.262 up	3.339 up	0.00	231.90	unknown protein
107848	9.85	0.08	11.61	0.23	11.92	0.42	3.382 up	4.186 up	0.01	25.48	multicopper oxidase type 1. secreted
107850	9.71	0.12	7.79	0.16	11.15	0.35	3.764 down	2.720 up	0.00	93.69	CE1 poly(3-hydroxybutyrate) depolymerase
107853	10.62	0.19	7.18	0.13	8.42	0.44	10.904 down	4.618 down	0.00	48.45	unknown protein
107857	9.52	0.06	8.77	0.06	8.40	0.21	1.684 down	2.167 down	0.00	30.75	unknown protein
107867	4.28	0.20	4.15	0.07	6.08	0.27	1.095 down	3.495 up	0.00	68.25	unique protein
107868	2.71	0.38	2.44	0.07	3.93	0.05	1.211 down	2.333 up	0.00	59.72	unknown protein
107869	5.12	0.13	3.59	0.11	6.35	0.20	2.888 down	2.341 up	0.00	178.26	unknown protein
107871	13.11	0.04	13.06	0.06	11.30	0.22	1.034 down	3.499 down	0.00	102.81	unknown protein
107888	11.77	0.19	11.35	0.22	9.91	0.40	1.333 down	3.619 down	0.01	24.63	unknown protein
107896	6.60	0.22	7.04	0.01	8.03	0.39	1.353 up	2.696 up	0.01	15.68	Isoflavone reductase
107914	9.63	0.10	9.37	0.10	7.58	0.19	1.197 down	4.142 down	0.00	150.08	unique protein
107935	6.88	0.01	6.30	0.07	5.85	0.22	1.500 down	2.048 down	0.01	23.64	Nucleolar GTPase/ATPase p130
107938	10.43	0.07	10.19	0.01	9.33	0.18	1.175 down	2.139 down	0.00	46.35	esterase or lipase
107947	12.36	0.13	12.35	0.05	10.52	0.26	1.010 down	3.591 down	0.00	75.29	Acyl-CoA synthetase
107949	3.64	0.22	3.37	0.17	4.88	0.10	1.208 down	2.364 up	0.00	87.52	unknown protein with ThiJ/PfpI domain
107961	5.57	0.24	5.45	0.29	7.18	0.35	1.088 down	3.059 up	0.01	27.76	unique protein
107974	4.73	0.37	4.83	0.45	7.11	0.43	1.077 up	5.221 up	0.00	30.65	BZIP transcriptional regulator transcription factor JlbA/IDI-4
107998	3.07	0.27	3.05	0.10	4.92	0.23	1.017 down	3.595 up	0.00	70.63	unique protein
108007	3.83	0.15	3.92	0.18	5.21	0.09	1.070 up	2.616 up	0.00	110.97	α/ß hydrolase lipase
108010	9.27	0.05	8.07	0.01	7.05	0.45	2.292 down	4.642 down	0.01	27.72	ABC transporter
108011	2.79	0.00	2.69	0.07	4.60	0.32	1.073 down	3.505 up	0.00	56.73	unknown protein
108012	3.10	0.12	3.08	0.00	4.61	0.14	1.013 down	2.836 up	0.00	161.05	serine peptidase S28
108013	4.28	0.23	3.91	0.10	5.85	0.09	1.292 down	2.980 up	0.00	177.36	Zn2Cys6 transcriptional regulator
108025	3.87	0.07	3.98	0.01	5.99	0.22	1.081 up	4.348 up	0.00	141.03	unknown protein
108075	5.26	0.05	6.74	0.03	6.91	0.19	2.786 up	3.138 up	0.00	82.53	unique protein
108081	12.80	0.21	13.62	0.06	13.82	0.10	1.766 up	2.025 up	0.00	45.10	kinetochore protein spc24. putative
108143	6.13	0.07	11.03	0.06	12.41	0.12	29.783 up	77.549 up	0.00	2605.29	unique protein
108144	4.07	0.30	7.99	0.09	9.49	0.24	15.129 up	42.756 up	0.00	368.68	unknown protein
108145	5.88	0.09	11.77	0.22	11.48	0.22	59.226 up	48.437 up	0.00	590.83	unique protein

108155	7.78	0.44	7.98	0.30	9.93	0.79	1.148 up	4.428 up	0.03	9.71	unknown protein. unique in fungi
108158	12.79	0.03	12.37	0.09	11.59	0.07	1.341 down	2.295 down	0.00	218.59	unknown protein
108185	4.12	0.23	4.27	0.21	6.09	0.25	1.108 up	3.932 up	0.00	62.49	unknown protein. unique in fungi
108188	3.28	0.19	3.28	0.00	5.35	0.48	1.002 down	4.188 up	0.00	29.87	unique protein
108201	12.53	0.14	12.04	0.07	10.60	0.14	1.405 down	3.825 down	0.00	177.30	Unknown protein with reductase domain
108208	12.34	0.02	11.70	0.16	10.66	0.46	1.553 down	3.192 down	0.01	15.49	unknown protein
108233	7.69	0.01	8.07	0.00	5.71	0.71	1.293 up	3.966 down	0.01	15.85	unknown protein. secreted. only in fungi
108251	2.69	0.06	2.87	0.06	4.33	0.06	1.130 up	3.113 up	0.00	649.20	SSCRP
108261	10.97	0.12	13.23	0.05	12.84	0.11	4.792 up	3.644 up	0.00	310.19	SSCRP
108263	11.91	0.07	11.88	0.11	10.75	0.27	1.021 down	2.224 down	0.01	27.03	unknown protein
108287	11.57	0.17	11.36	0.09	10.33	0.08	1.155 down	2.372 down	0.00	118.23	unknown protein
108333	9.27	0.32	8.98	0.05	7.77	0.24	1.223 down	2.829 down	0.00	34.40	unknown protein
108335	4.12	0.07	5.20	0.27	5.30	0.19	2.116 up	2.272 up	0.01	27.59	unknown protein
108339	6.47	0.39	6.89	0.27	8.22	0.35	1.337 up	3.360 up	0.01	20.42	unique protein
108340	7.64	0.02	6.75	0.06	10.08	0.19	1.860 down	5.397 up	0.00	406.14	unique protein
108341	6.90	0.55	7.50	0.09	8.89	0.59	1.519 up	3.967 up	0.02	11.28	unique protein
108342	3.05	0.16	3.56	0.44	5.20	0.08	1.424 up	4.452 up	0.00	79.38	unique protein
108343	5.67	0.17	6.74	0.20	6.96	0.29	2.097 up	2.438 up	0.01	17.71	unique protein
108344	5.04	0.08	5.15	0.12	6.32	0.30	1.076 up	2.424 up	0.01	26.47	unique protein
108346	3.46	0.08	3.59	0.18	5.28	0.26	1.099 up	3.550 up	0.00	65.72	GH18. chitinase CHI18-8
108348	5.19	0.27	5.61	0.03	6.46	0.38	1.335 up	2.410 up	0.02	11.86	unknown protein
108367	4.60	0.47	5.13	0.15	8.15	0.67	1.444 up	11.732 up	0.00	34.53	unique protein
108383	3.63	0.37	4.05	0.28	5.68	0.38	1.339 up	4.132 up	0.01	27.20	unique protein
108384	4.80	0.30	5.15	0.32	7.85	1.07	1.269 up	8.260 up	0.02	11.47	unique protein
108412	2.33	0.02	2.30	0.16	3.67	0.09	1.023 down	2.526 up	0.00	191.57	SSCRP
108418	6.25	0.06	7.39	0.01	7.81	0.28	2.204 up	2.947 up	0.00	34.58	SSCRP
108431	9.94	0.23	9.89	0.06	11.39	0.13	1.038 down	2.735 up	0.00	103.00	unknown protein
108449	10.53	0.13	11.14	0.02	9.44	0.14	1.525 up	2.132 down	0.00	134.86	unknown protein
108457	4.12	0.30	4.55	0.00	5.21	0.26	1.349 up	2.131 up	0.02	14.32	unique protein
108482	8.76	0.06	8.85	0.11	11.16	0.29	1.064 up	5.286 up	0.00	102.80	fumarylacetoacetate hydrolase

108542	10.56	0.07	11.56	0.21	11.80	0.19	2.003 up	2.357 up	0.00	32.11	unknown protein
108559	13.24	0.08	13.32	0.02	12.02	0.27	1.051 up	2.328 down	0.00	34.99	unknown protein
108566	5.95	0.24	6.34	0.09	7.56	0.27	1.313 up	3.061 up	0.00	36.07	unknown protein
108585	6.82	0.10	6.41	0.16	11.23	0.73	1.330 down	21.213 up	0.00	65.35	unknown protein with CHAP domain
108586	8.03	0.06	10.60	0.04	13.34	0.33	5.954 up	39.802 up	0.00	296.23	unknown protein
108604	4.70	0.06	4.67	0.59	6.30	0.37	1.020 down	3.048 up	0.01	17.22	unique protein
108605	7.11	0.06	7.84	0.12	8.67	0.21	1.661 up	2.953 up	0.00	54.22	unique protein
108635	6.16	0.11	6.94	0.25	7.59	0.12	1.715 up	2.706 up	0.00	57.99	unknown protein
108655	5.91	0.06	6.04	0.12	7.06	0.09	1.090 up	2.217 up	0.00	146.02	unknown protein. secreted
108663	7.53	0.22	8.83	0.05	12.65	0.16	2.454 up	34.752 up	0.00	789.27	SSCRP
108665	4.19	0.21	3.89	0.06	5.22	0.33	1.229 down	2.034 up	0.01	19.51	unknown protein
108681	4.07	0.21	4.23	0.30	5.90	0.20	1.117 up	3.541 up	0.00	61.22	unknown protein
108683	6.49	0.04	6.06	0.05	7.60	0.12	1.342 down	2.152 up	0.00	192.75	unknown protein. contains 5 TM
108686	9.02	0.01	7.62	0.17	6.79	0.32	2.650 down	4.702 down	0.00	49.70	Aspartyl protease
108721	3.11	0.14	3.03	0.09	6.78	1.12	1.053 down	12.803 up	0.01	18.11	unknown protein. unique in fungi
108749	10.60	0.05	10.57	0.05	13.86	0.09	1.017 down	9.597 up	0.00	1792.15	Copper transporter
108776	5.42	0.14	5.50	0.11	6.77	0.23	1.054 up	2.537 up	0.00	44.04	GH55 β-1.3-glucanase
108778	12.10	0.09	12.68	0.21	13.15	0.12	1.494 up	2.071 up	0.00	40.24	unknown protein
108802	5.40	0.43	7.23	0.16	6.47	0.50	3.568 up	2.104 up	0.03	8.78	unique protein
108806	13.41	0.26	13.31	0.20	12.04	0.12	1.073 down	2.590 down	0.00	60.39	unknown protein
108832	4.67	0.03	5.36	0.05	6.43	0.40	1.613 up	3.379 up	0.01	22.87	unknown protein
108866	8.15	0.03	9.44	0.08	9.99	0.19	2.443 up	3.576 up	0.00	93.55	unknown protein
108885	12.48	0.05	11.32	0.02	10.31	0.11	2.243 down	4.499 down	0.00	435.96	amidase
108893	10.09	0.02	8.02	0.18	8.29	0.21	4.190 down	3.480 down	0.00	80.26	MFS permease
108899	11.98	0.11	11.29	0.03	10.38	0.11	1.612 down	3.030 down	0.00	197.34	unknown protein
108914	11.09	0.02	10.39	0.00	13.20	0.73	1.630 down	4.303 up	0.01	19.69	methyltransferase type 11
108920	11.89	0.11	11.81	0.03	10.60	0.19	1.058 down	2.443 down	0.00	66.13	zinc-binding dehydrogenase
108928	3.79	0.20	4.10	0.14	4.92	0.19	1.235 up	2.186 up	0.00	28.83	unique protein
108940	12.64	0.10	11.93	0.26	10.68	0.16	1.633 down	3.881 down	0.00	92.71	Zn2Cys6 transcriptional regulator
108953	7.04	0.05	7.98	0.00	8.23	0.32	1.913 up	2.273 up	0.01	15.58	unknown protein

108996	9.41	0.22	10.37	0.26	12.84	0.67	1.946 up	10.748 up	0.00	30.91	unique protein
109030	2.61	0.09	3.29	0.04	4.54	0.14	1.610 up	3.826 up	0.00	195.92	unique protein
109044	3.59	0.12	3.69	0.36	5.01	0.19	1.071 up	2.676 up	0.00	37.24	unique protein
109071	4.63	0.45	4.22	0.23	7.66	0.42	1.333 down	8.135 up	0.00	66.01	unknown protein
109075	5.85	0.19	5.96	0.08	7.07	0.54	1.076 up	2.320 up	0.04	7.50	unknown protein
109102	3.64	0.13	3.96	0.14	5.27	0.27	1.240 up	3.093 up	0.00	42.10	unknown protein
109122	10.96	0.15	9.07	0.13	9.78	0.09	3.690 down	2.256 down	0.00	142.60	Amino acid transporters
109146	9.87	0.10	5.49	0.08	4.27	0.07	20.774 down	48.526 down	0.00	3317.18	PTH11 GPCR
109147	2.36	0.22	2.37	0.18	3.66	0.06	1.004 up	2.450 up	0.00	90.13	unknown protein
109231	4.17	0.29	3.84	0.02	6.01	0.25	1.265 down	3.577 up	0.00	76.81	SSCRP
109234	11.81	0.29	10.98	0.18	12.95	0.33	1.780 down	2.210 up	0.00	30.69	D-aminopeptidase
109237	4.75	0.40	4.77	0.12	6.45	0.25	1.014 up	3.251 up	0.00	40.54	Cytochrome P450
109239	3.60	0.42	3.18	0.39	5.80	0.53	1.331 down	4.596 up	0.01	24.74	unknown protein. only in Aspergilli
109243	4.67	0.31	5.14	0.27	7.99	0.60	1.386 up	9.969 up	0.00	38.89	unique protein
109244	10.65	0.08	9.82	0.17	9.58	0.24	1.775 down	2.097 down	0.01	18.07	unique protein
109249	10.73	0.05	9.00	0.08	8.78	0.33	3.319 down	3.862 down	0.00	40.37	unknown protein
109255	6.96	0.00	6.56	0.02	8.07	0.57	1.311 down	2.170 up	0.03	9.21	SSCRP
109264	3.44	0.45	3.93	0.01	4.61	0.19	1.409 up	2.256 up	0.01	16.10	unique protein
109267	5.75	0.25	6.81	0.08	6.83	0.37	2.083 up	2.106 up	0.03	9.11	unique protein
109278	7.01	0.15	8.24	0.07	9.85	0.82	2.351 up	7.186 up	0.02	13.89	GH24 lysozyme
109284	4.41	0.23	4.47	0.05	6.74	0.08	1.044 up	5.045 up	0.00	350.93	unknown protein
109304	3.87	0.24	4.23	0.05	5.65	0.26	1.284 up	3.443 up	0.00	50.11	unknown protein
109305	4.03	0.58	4.23	0.43	6.06	0.20	1.144 up	4.070 up	0.00	29.21	unique protein
109307	9.01	0.08	7.80	0.18	7.62	0.44	2.326 down	2.633 down	0.02	11.18	unknown protein
109319	5.26	0.22	5.58	0.10	7.79	0.22	1.253 up	5.783 up	0.00	136.60	unknown protein
109320	4.60	0.02	5.08	0.09	6.84	0.28	1.393 up	4.709 up	0.00	81.05	unique protein
109331	3.99	0.31	4.61	0.03	5.55	0.12	1.533 up	2.944 up	0.00	61.47	unknown protein. secreted. 5 TM
109334	4.07	0.15	4.41	0.14	5.89	0.37	1.270 up	3.527 up	0.00	30.22	unknown protein
109335	4.63	0.01	6.04	0.09	6.06	0.24	2.651 up	2.695 up	0.00	40.87	SSCRP
109338	3.88	0.12	3.47	0.43	5.40	0.08	1.325 down	2.868 up	0.00	68.86	unknown protein. in A. oryzae and G. zeae

109341	7.81	0.07	6.31	0.11	6.63	0.15	2.831 down	2.260 down	0.00	78.13	ankyrin
109345	3.44	0.04	3.41	0.21	5.12	0.44	1.019 down	3.225 up	0.01	22.77	unique protein
109349	2.70	0.14	2.76	0.06	4.07	0.10	1.041 up	2.587 up	0.00	172.41	short chain dehydrogenase/reductase
109351	2.75	0.04	3.06	0.04	4.64	0.19	1.237 up	3.722 up	0.00	136.67	unique protein
109353	3.78	0.09	4.55	0.16	5.49	0.12	1.696 up	3.270 up	0.00	139.14	unknown protein
109357	4.32	0.25	4.91	0.16	6.08	0.24	1.502 up	3.389 up	0.00	44.36	short chain dehydrognease/reductase
109359	5.56	0.14	5.72	0.25	6.64	0.45	1.115 up	2.116 up	0.04	7.48	unknown protein
109361	4.51	0.14	6.27	0.15	5.92	0.17	3.399 up	2.671 up	0.00	69.42	GT32 α-glycosyltransferase
109364	4.35	0.51	4.48	0.04	6.32	0.40	1.098 up	3.925 up	0.01	25.02	unknown protein
109367	9.99	0.02	4.94	0.10	6.36	0.56	33.158 down	12.434 down	0.00	74.15	unique protein
109370	3.19	0.27	3.42	0.00	4.72	0.12	1.169 up	2.885 up	0.00	90.48	SSCRP
109376	2.67	0.00	2.80	0.16	4.78	0.13	1.098 up	4.328 up	0.00	261.80	glutamate decarbounknown proteinlase
109379	12.45	0.04	10.85	0.12	10.79	0.26	3.043 down	3.165 down	0.00	46.07	short chain dehydrogenase/reductase
109388	3.92	0.33	3.74	0.04	5.08	0.14	1.137 down	2.230 up	0.00	47.87	unknown protein
109392	5.34	0.21	6.06	0.10	7.85	0.54	1.649 up	5.707 up	0.01	26.05	unknown protein
109410	10.70	0.05	10.31	0.20	9.09	0.25	1.307 down	3.048 down	0.00	43.94	unknown protein
109415	3.21	0.13	3.39	0.10	5.66	0.48	1.134 up	5.453 up	0.00	38.36	unknown protein
109416	12.14	0.08	11.94	0.05	11.14	0.41	1.147 down	2.005 down	0.04	8.14	unknown protein
109435	9.96	0.00	10.61	0.07	11.23	0.13	1.562 up	2.412 up	0.00	96.21	unknown protein. 1 TM
109475	4.68	0.07	4.87	0.04	6.85	0.47	1.147 up	4.516 up	0.00	32.23	unique protein
109486	9.07	0.09	9.49	0.08	8.04	0.52	1.343 up	2.030 down	0.03	9.64	unique protein
109489	3.88	0.31	4.14	0.10	5.48	0.30	1.200 up	3.025 up	0.00	29.73	unknown protein
109526	8.28	0.07	2.60	0.26	9.46	0.28	51.119 down	2.258 up	0.00	525.54	SNF2 family helicase/ATPase
109538	12.62	0.22	8.46	0.04	9.66	0.25	17.920 down	7.807 down	0.00	197.72	BZIP transcriptional regulator
109562	5.56	0.39	7.79	0.03	7.69	0.26	4.685 up	4.390 up	0.00	49.41	unique protein
109590	3.19	0.17	3.44	0.05	4.69	0.09	1.189 up	2.828 up	0.00	186.79	unknown protein
109607	2.27	0.17	2.19	0.04	3.58	0.04	1.056 down	2.491 up	0.00	261.83	unique protein
109608	3.13	0.13	3.33	0.10	4.72	0.19	1.148 up	3.013 up	0.00	85.18	unique protein
109668	5.00	0.11	4.82	0.03	6.60	0.36	1.135 down	3.029 up	0.00	35.17	unique protein
109677	10.46	0.11	10.96	0.20	8.52	0.25	1.422 up	3.833 down	0.00	102.97	MFS permease

109721	7.52	0.19	7.82	0.02	8.62	0.15	1.234 up	2.147 up	0.00	46.43	unknown protein
109725	4.14	0.26	4.08	0.02	6.24	0.11	1.040 down	4.279 up	0.00	213.87	unknown protein
109726	4.53	0.07	5.37	0.06	5.54	0.19	1.795 up	2.016 up	0.00	30.87	unique protein
109748	12.04	0.01	11.35	0.18	9.99	0.35	1.616 down	4.129 down	0.00	38.19	Zn2Cys6 transcriptional regulator
109756	10.96	0.06	11.00	0.09	9.81	0.15	1.026 up	2.215 down	0.00	81.77	unknown protein
109787	13.60	0.05	12.68	0.05	12.51	0.16	1.891 down	2.121 down	0.00	47.32	SSCRP
109790	11.91	0.05	12.12	0.19	12.99	0.21	1.158 up	2.102 up	0.01	27.08	pyridoxamine 5'-phosphate oxidase
109801	6.34	0.33	5.77	0.22	8.73	0.31	1.490 down	5.221 up	0.00	81.05	unknown protein
109815	5.02	0.06	4.72	0.00	6.14	0.21	1.229 down	2.176 up	0.00	59.92	unknown protein
109835	10.61	0.04	10.87	0.04	8.57	0.17	1.204 up	4.092 down	0.00	255.60	unknown protein. thaumatin family
109837	4.07	0.21	4.55	0.85	5.93	0.12	1.391 up	3.619 up	0.01	16.77	MFS permease
109850	12.58	0.02	10.77	0.00	9.99	0.12	3.502 down	6.017 down	0.00	550.87	unknown protein
109872	12.98	0.06	12.65	0.04	11.79	0.17	1.258 down	2.277 down	0.00	57.97	unknown protein
109883	12.05	0.06	11.99	0.20	10.89	0.35	1.039 down	2.228 down	0.01	15.63	unknown protein
109895	11.89	0.11	11.77	0.20	10.47	0.05	1.084 down	2.668 down	0.00	150.37	ATP-dependent Clp protease
109908	5.18	0.06	5.14	0.01	6.59	0.22	1.024 down	2.665 up	0.00	70.37	C2H2 transcriptional regulator
109910	5.26	0.11	5.25	0.17	6.61	0.29	1.006 down	2.551 up	0.00	32.21	unique protein. secreted
109911	3.93	0.18	4.17	0.12	6.27	0.54	1.179 up	5.082 up	0.01	27.13	SSCRP
109925	12.11	0.02	6.26	0.43	10.15	0.60	57.532 down	3.871 down	0.00	70.89	unknown protein
109929	11.89	0.12	11.56	0.17	10.27	0.16	1.256 down	3.070 down	0.00	89.64	unknown protein
109934	6.88	0.45	7.14	0.10	8.70	0.37	1.194 up	3.526 up	0.01	22.96	unknown protein
109943	3.07	0.03	3.05	0.03	4.75	0.10	1.015 down	3.202 up	0.00	460.78	unknown protein
109946	3.22	0.18	3.14	0.13	4.53	0.19	1.057 down	2.469 up	0.00	60.19	unknown protein
109949	5.83	0.20	5.65	0.23	7.88	0.42	1.132 down	4.140 up	0.00	37.51	unknown protein
109952	3.84	0.21	4.23	0.29	5.86	0.33	1.302 up	4.036 up	0.00	37.12	unknown protein. containing ankyrin repeats
109960	9.73	0.00	9.32	0.01	6.89	0.90	1.329 down	7.162 down	0.02	14.60	unknown protein in Sordariomycetes
109967	4.41	0.11	4.45	0.16	5.98	0.29	1.023 up	2.968 up	0.00	41.68	unique protein. 4 TM
109998	13.32	0.05	12.91	0.05	12.03	0.15	1.333 down	2.444 down	0.00	90.40	glutathione S-transferase
110007	10.98	0.02	10.55	0.08	9.28	0.22	1.345 down	3.235 down	0.00	73.32	unique protein
110025	5.20	0.21	5.87	0.08	7.34	0.14	1.590 up	4.411 up	0.00	157.65	SSCRP

110035	4.36	0.39	4.06	0.13	7.34	0.98	1.229 down	7.891 up	0.01	16.12	unknown protein. in Sordariomycetes
110056	6.31	0.07	6.53	0.01	7.83	0.45	1.163 up	2.870 up	0.01	16.65	unknown protein
110058	4.66	0.16	4.93	0.21	5.93	0.16	1.211 up	2.413 up	0.00	45.36	SSCRP
110078	6.74	0.24	6.49	0.25	8.69	0.57	1.188 down	3.866 up	0.01	19.59	SSCRP
110096	8.03	0.15	8.75	0.19	9.31	0.37	1.655 up	2.439 up	0.02	12.17	unknown protein
110117	6.81	0.44	7.39	0.26	9.11	0.62	1.491 up	4.906 up	0.01	14.86	SSCRP
110127	6.92	0.22	8.06	0.07	8.44	0.54	2.193 up	2.856 up	0.04	8.34	unique protein
110128	4.28	0.77	4.46	0.13	8.80	0.61	1.136 up	23.050 up	0.00	56.24	unique protein
110131	2.74	0.17	2.86	0.00	4.54	0.14	1.089 up	3.501 up	0.00	170.19	unknown protein
110133	11.52	0.12	11.06	0.08	10.32	0.14	1.377 down	2.299 down	0.00	63.62	Mitochondrial carrier protein
110151	4.59	0.06	5.24	0.21	10.46	0.22	1.560 up	58.240 up	0.00	814.50	unique secreted protein
110152	10.75	0.04	12.02	0.06	12.00	0.17	2.400 up	2.376 up	0.00	62.60	BZIP transcriptional regulator
110153	3.08	0.12	3.41	0.01	4.53	0.11	1.254 up	2.726 up	0.00	171.26	unique protein
110173	6.96	0.08	9.37	0.16	8.29	0.27	5.323 up	2.512 up	0.00	60.04	unknown protein
110197	4.79	0.22	5.00	0.08	6.02	0.26	1.161 up	2.347 up	0.01	24.97	unknown protein
110207	3.11	0.07	3.36	0.06	5.24	0.25	1.186 up	4.385 up	0.00	105.06	unique protein
110224	3.69	0.12	3.72	0.12	5.90	0.26	1.019 up	4.625 up	0.00	103.99	unknown protein
110225	6.20	0.17	6.64	0.15	7.49	0.41	1.358 up	2.437 up	0.02	10.94	unknown protein
110259	3.42	0.12	3.43	0.18	4.78	0.12	1.004 up	2.553 up	0.00	107.31	alginate lyases
110261	10.49	0.19	8.66	0.16	11.81	0.25	3.567 down	2.491 up	0.00	132.52	unique protein
110262	9.63	0.07	8.65	0.01	7.98	0.13	1.965 down	3.144 down	0.00	163.53	Zinc-binding oxidoreductase
110263	8.99	0.04	8.67	0.05	7.58	0.23	1.245 down	2.653 down	0.00	51.50	unknown protein
110265	8.11	0.16	4.43	0.10	7.02	0.37	12.882 down	2.138 down	0.00	81.62	unknown protein
110276	6.95	0.18	7.61	0.01	5.87	0.53	1.577 up	2.120 down	0.02	12.56	isoflavone reductase
110281	6.25	0.28	6.39	0.05	7.81	0.40	1.098 up	2.952 up	0.01	20.07	unknown protein
110295	9.88	0.18	10.34	0.14	11.73	0.37	1.372 up	3.610 up	0.00	30.44	ribosomal protein S14
110317	3.11	0.09	3.00	0.07	4.82	0.12	1.083 down	3.254 up	0.00	285.38	GH18. chitinase CHI18-17
110329	3.76	0.15	3.61	0.19	5.51	0.27	1.112 down	3.372 up	0.00	61.29	unknown protein. only present in ascomycota
110337	4.89	0.05	5.04	0.47	6.53	0.46	1.111 up	3.133 up	0.02	14.18	unknown protein with thij/pfpi domain
110343	2.89	0.13	3.23	0.01	5.09	0.35	1.257 up	4.573 up	0.00	54.07	unique protein

110345	4.53	0.05	4.74	0.07	5.65	0.17	1.156 up	2.179 up	0.00	57.77	unique protein
110363	5.58	0.10	6.92	0.12	8.76	0.21	2.528 up	9.052 up	0.00	230.09	unique protein
110366	9.35	0.04	8.65	0.09	7.54	0.28	1.620 down	3.498 down	0.00	46.95	unique protein
110396	11.49	0.03	11.34	0.16	13.17	0.22	1.116 down	3.199 up	0.00	88.22	unknown protein with Tim10/DDP family zinc finger
110403	8.85	0.00	8.93	0.26	7.67	0.37	1.058 up	2.273 down	0.01	15.58	unknown protein
110414	13.22	0.05	12.37	0.08	11.27	0.31	1.803 down	3.884 down	0.00	44.33	Aminotran_Aminotransferase class-III
110415	11.32	0.01	10.91	0.12	10.23	0.10	1.328 down	2.131 down	0.00	102.24	Serine/threonine protein kinase
110430	11.59	0.05	11.04	0.13	10.17	0.23	1.466 down	2.673 down	0.00	42.64	MFS permease
110433	2.40	0.10	2.59	0.11	3.92	0.10	1.141 up	2.874 up	0.00	204.88	unique protein
110440	9.72	0.13	8.62	0.02	7.46	0.09	2.145 down	4.792 down	0.00	446.17	unique protein
110455	8.68	0.09	8.21	0.16	6.06	0.53	1.381 down	6.166 down	0.00	33.03	short chain dehydrogenase/reductase
110457	6.36	0.21	6.19	0.09	7.94	0.37	1.125 down	2.982 up	0.00	29.54	SSCRP
110471	12.46	0.14	12.09	0.09	10.29	0.26	1.289 down	4.504 down	0.00	84.38	Cu2+/Zn2+ superoxide dismutase SOD1
110490	6.52	0.16	7.31	0.12	7.75	0.26	1.721 up	2.343 up	0.01	20.93	unknown protein
110569	6.00	0.40	6.23	0.18	7.36	0.15	1.168 up	2.568 up	0.00	31.05	SSCRP
110595	11.60	0.06	10.68	0.21	8.95	0.52	1.896 down	6.313 down	0.00	30.75	unknown protein
110600	9.36	0.11	10.06	0.09	10.37	0.08	1.629 up	2.023 up	0.00	86.96	unknown protein
110615	11.24	0.11	10.59	0.23	10.21	0.38	1.573 down	2.040 down	0.05	6.99	unknown protein
110638	12.85	0.13	12.92	0.07	11.69	0.12	1.045 up	2.235 down	0.00	110.36	mitochondrial carrier protein
110660	6.30	0.04	5.61	0.06	7.33	0.64	1.611 down	2.045 up	0.04	8.69	unknown protein
110663	4.80	0.26	5.60	0.26	6.38	0.21	1.743 up	3.007 up	0.00	32.23	AMP-dependent synthetase and ligase. putative
110665	4.64	0.14	4.99	0.22	7.82	0.71	1.277 up	9.084 up	0.00	29.02	unique protein
110666	4.02	0.18	3.42	0.33	8.15	0.80	1.512 down	17.592 up	0.00	47.69	ferric reductase
110684	3.59	0.09	3.89	0.42	5.25	0.40	1.232 up	3.155 up	0.01	17.18	unique protein
110686	8.95	0.14	8.81	0.11	7.68	0.27	1.099 down	2.404 down	0.00	29.61	unknown protein
110688	3.29	0.08	3.27	0.28	6.35	0.82	1.016 down	8.358 up	0.01	22.52	unique protein
110689	10.66	0.00	9.07	0.08	8.75	0.27	3.007 down	3.777 down	0.00	55.40	Zn2Cys6 transcriptional regulator
110691	5.68	0.16	5.70	0.02	7.58	0.30	1.009 up	3.730 up	0.00	60.28	unique protein
110712	4.29	0.42	4.08	0.25	5.42	0.11	1.161 down	2.177 up	0.01	27.98	unique protein
110729	3.76	0.05	4.13	0.04	5.42	0.08	1.294 up	3.166 up	0.00	486.94	unique protein

110732	4.31	0.25	3.44	0.21	5.35	0.10	1.826 down	2.061 up	0.00	96.54	unique protein
110735	2.98	0.22	3.13	0.01	4.95	0.16	1.106 up	3.912 up	0.00	149.63	unique protein
110740	4.39	0.07	4.79	0.31	6.15	0.25	1.315 up	3.374 up	0.00	43.87	unknown protein
110749	3.67	0.11	3.54	0.04	4.68	0.13	1.088 down	2.018 up	0.00	88.44	unknown protein
110752	3.08	0.14	3.01	0.04	4.61	0.13	1.050 down	2.880 up	0.00	170.13	unique protein
110753	2.94	0.29	3.22	0.23	4.81	0.16	1.208 up	3.642 up	0.00	72.73	Na+/proline symporter PutP
110757	9.79	0.04	7.40	0.03	7.76	0.13	5.229 down	4.072 down	0.00	341.34	imidazole proprionase-related amidohydrolase
110762	3.24	0.19	5.30	0.11	4.59	0.40	4.159 up	2.539 up	0.01	21.17	unknown protein
110763	3.35	0.25	3.47	0.08	5.16	0.26	1.086 up	3.513 up	0.00	56.98	unknown protein
110767	10.66	0.09	9.06	0.12	9.20	0.16	3.035 down	2.745 down	0.00	83.51	unique protein
110768	13.16	0.22	11.78	0.07	11.43	0.17	2.600 down	3.324 down	0.00	75.87	unknown protein
110791	2.61	0.07	2.51	0.13	4.45	0.16	1.074 down	3.579 up	0.00	179.94	unknown protein
110797	5.21	0.06	4.26	0.28	7.13	0.30	1.931 down	3.776 up	0.00	88.78	unknown protein. 1 TM. only in Gibberella
110811	13.72	0.03	13.59	0.01	12.31	0.14	1.088 down	2.654 down	0.00	143.60	unknown protein
110813	11.61	0.10	12.25	0.01	12.74	0.06	1.560 up	2.185 up	0.00	217.13	Amino acid transporter LysP
110825	6.98	0.07	5.97	0.18	5.98	0.32	2.009 down	2.000 down	0.02	10.77	unique protein
110831	9.34	0.05	9.07	0.04	8.10	0.35	1.207 down	2.371 down	0.01	17.31	unknown protein
110848	3.32	0.16	3.45	0.16	5.57	0.52	1.096 up	4.761 up	0.01	27.31	GH23 exo-ß-1.3-glucanase. distantly related
110850	7.33	0.08	5.89	0.11	6.02	0.32	2.702 down	2.474 down	0.01	21.30	Zn2Cys6 transcriptional regulator
110855	5.72	0.36	8.21	0.14	4.68	0.15	5.605 up	2.058 down	0.00	197.03	unique protein
110860	3.30	0.33	3.81	0.12	4.91	0.24	1.422 up	3.051 up	0.00	33.03	Na+-independent Cl/HCO3 exchanger AE1 and related transporters (SLC4 family)
110866	12.04	0.02	11.71	0.21	9.75	0.09	1.254 down	4.888 down	0.00	318.23	metal-dependent amidase/aminoacylase/carbounknown proteinpeptidase
110868	4.98	0.07	4.40	0.29	6.23	0.46	1.491 down	2.392 up	0.01	17.93	unknown protein
110890	11.15	0.04	11.81	0.16	12.15	0.05	1.586 up	2.003 up	0.00	100.27	porphobilinogen deaminase-like protein
110891	10.04	0.18	6.19	0.20	8.36	0.43	14.392 down	3.207 down	0.00	59.30	unique protein
110894	7.01	0.12	6.23	0.11	9.29	0.91	1.711 down	4.867 up	0.01	14.92	GH30 endo-β-1 6-galactanase
110901	8.71	0.03	8.00	0.06	7.37	0.30	1.634 down	2.520 down	0.01	21.87	Zn2Cys6 transcriptional regulator
110902	6.72	0.44	6.98	0.56	8.70	0.47	1.201 up	3.948 up	0.01	14.66	unique protein
110966	3.00	0.42	3.31	0.11	5.05	0.14	1.240 up	4.159 up	0.00	73.12	unknown protein
111005	3.31	0.04	3.81	0.35	4.99	0.14	1.407 up	3.203 up	0.00	59.76	unique protein

111012	7.76	0.50	7.79	0.23	9.71	0.80	1.020 up	3.849 up	0.04	8.30	unique protein
111027	6.00	0.26	6.68	0.08	8.43	0.17	1.596 up	5.366 up	0.00	144.57	unknown protein
111038	10.59	0.09	10.60	0.09	9.30	0.12	1.002 up	2.448 down	0.00	139.33	unique protein
111048	5.33	0.29	5.54	0.08	6.38	0.11	1.155 up	2.059 up	0.00	35.58	UbiA prenyltransferase. putative
111049	4.28	0.21	4.21	0.18	6.47	0.19	1.047 down	4.564 up	0.00	131.45	cytochrome P450 protein. class IV
111050	7.98	0.12	8.28	0.01	6.90	0.25	1.228 up	2.126 down	0.00	38.80	unique protein
111053	12.41	0.13	8.69	0.10	10.54	0.23	13.178 down	3.633 down	0.00	183.75	retrograde regulation protein 2
111059	9.53	0.10	9.46	0.00	8.18	0.34	1.050 down	2.545 down	0.01	23.87	unknown protein
111082	11.76	0.12	10.93	0.08	13.19	0.10	1.768 down	2.695 up	0.00	364.00	glutathione S transferase. 2 TM
111093	4.25	0.12	3.80	0.37	6.00	0.20	1.361 down	3.370 up	0.00	73.09	3-beta hydroxysteroid dehydrogenase/isomerase. putative
111095	7.57	0.03	10.01	0.10	9.96	0.56	5.410 up	5.226 up	0.01	22.68	unknown protein
111108	3.90	0.07	3.88	0.01	5.86	0.19	1.011 down	3.895 up	0.00	177.71	unknown protein
111109	4.13	0.12	4.62	0.16	5.44	0.24	1.405 up	2.480 up	0.00	30.31	unique protein
111110	7.89	0.02	9.39	0.13	10.24	0.43	2.815 up	5.076 up	0.00	32.79	SAM-dependent methyltransferases
111119	7.37	0.02	6.79	0.00	6.20	0.28	1.487 down	2.244 down	0.01	20.03	bZI) transcription factor. possibly related to AP1
111121	4.77	0.07	5.11	0.00	6.19	0.49	1.267 up	2.663 up	0.02	10.84	metallopeptidase
111124	9.66	0.04	9.78	0.02	8.26	0.30	1.089 up	2.641 down	0.00	40.86	unknown protein
111129	13.52	0.01	13.39	0.11	12.10	0.26	1.091 down	2.666 down	0.00	42.25	unknown protein
111135	11.96	0.08	10.78	0.21	10.25	0.12	2.277 down	3.293 down	0.00	106.10	unknown protein
111138	10.06	0.07	9.40	0.26	8.49	0.24	1.573 down	2.970 down	0.00	36.18	unknown protein. secreted
111144	3.53	0.38	3.71	0.20	5.39	0.20	1.139 up	3.648 up	0.00	52.26	unique protein
111170	6.77	0.06	7.54	0.12	8.76	0.57	1.712 up	3.986 up	0.02	14.29	unknown protein
111171	3.10	0.08	3.47	0.03	4.97	0.12	1.288 up	3.651 up	0.00	276.74	unique protein
111192	10.46	0.11	10.31	0.15	11.59	0.11	1.110 down	2.199 up	0.00	99.58	unknown protein
111194	14.46	0.04	14.35	0.04	13.27	0.25	1.078 down	2.276 down	0.00	33.36	unknown protein
111204	5.44	0.24	5.66	0.17	7.32	0.55	1.163 up	3.676 up	0.01	15.94	unknown protein
111205	7.76	0.24	8.22	0.06	9.35	0.45	1.381 up	3.027 up	0.01	14.93	SSCRP
111236	11.54	0.11	11.37	0.30	12.71	0.11	1.124 down	2.264 up	0.00	56.14	N-acetyltransferase of bacterial origin
111245	10.37	0.03	8.88	0.08	8.27	0.24	2.789 down	4.278 down	0.00	79.09	PL8 polysaccharide lyase; distantly related to chondroitin lyases
111252	4.70	0.70	4.78	0.62	7.54	0.37	1.054 up	7.154 up	0.00	30.49	unique protein

111272	4.56	0.14	4.91	0.40	6.15	0.36	1.274 up	3.013 up	0.01	18.40	unknown protein
111289	3.04	0.25	3.12	0.25	4.37	0.06	1.051 up	2.506 up	0.00	63.08	unique protein
111304	9.31	0.17	7.60	0.12	7.04	0.19	3.260 down	4.816 down	0.00	112.84	unknown protein
111326	4.46	0.12	4.62	0.02	5.53	0.33	1.112 up	2.090 up	0.02	14.26	unknown protein
111345	8.74	0.06	8.12	0.20	10.77	0.37	1.540 down	4.093 up	0.00	62.33	unknown protein. Sordariomycetes. 1 TM
111351	12.67	0.01	12.28	0.11	11.51	0.16	1.311 down	2.230 down	0.00	55.13	DHBP_synthase.-dihydroxy--butanone -phosphate synthase
111357	12.79	0.04	12.25	0.03	10.55	0.15	1.449 down	4.725 down	0.00	298.03	FAD-dependent oxidoreductase
111362	12.83	0.16	10.07	0.14	11.54	0.56	6.778 down	2.442 down	0.01	19.41	unknown protein
111372	5.73	0.40	6.56	0.12	7.11	0.18	1.779 up	2.619 up	0.01	23.35	unique protein
111382	12.26	0.05	10.90	0.26	9.46	0.22	2.571 down	6.966 down	0.00	130.67	ankyrin
111393	2.80	0.24	2.83	0.14	5.04	0.18	1.019 up	4.733 up	0.00	138.09	unique protein
111405	3.94	0.77	2.71	0.01	6.73	0.53	2.346 down	6.898 up	0.00	42.97	unique protein
111442	10.76	0.09	7.60	0.06	7.76	0.23	8.953 down	8.042 down	0.00	202.36	Epl1/Sm1
111443	8.50	0.06	6.38	0.06	10.22	0.19	4.343 down	3.299 up	0.00	444.33	unique protein
111446	12.13	0.08	12.11	0.03	9.66	0.46	1.017 down	5.552 down	0.00	46.77	Zn2Cys6 transcriptional regulator
111449	2.35	0.02	2.50	0.13	3.82	0.10	1.113 up	2.773 up	0.00	216.42	Epl1-like protein
111451	6.40	0.06	6.65	0.23	7.93	0.50	1.188 up	2.894 up	0.02	12.59	peptidase S41
111465	2.96	0.33	5.62	0.50	4.85	0.25	6.317 up	3.691 up	0.00	34.88	unique protein
111466	4.90	0.17	4.90	0.12	6.56	0.18	1.001 up	3.149 up	0.00	97.39	Zn2Cys6 transcriptional regulator
111476	10.24	0.03	9.74	0.05	8.63	0.11	1.416 down	3.062 down	0.00	263.15	unknown protein
111495	9.92	0.09	6.34	0.28	11.58	0.56	11.978 down	3.161 up	0.00	90.27	SSCRP
111499	5.59	0.17	5.96	0.31	6.95	0.23	1.291 up	2.560 up	0.01	25.10	MRSP1/expansin-like
111502	11.44	0.24	12.32	0.27	12.94	0.19	1.838 up	2.827 up	0.00	32.00	NADH:ubiquinone oxidoreductase. NDUFB7/B18 subunit
111515	8.59	0.16	5.53	0.19	6.67	0.22	8.323 down	3.766 down	0.00	116.51	Zn2Cys6 transcriptional regulator
111527	11.39	0.03	10.52	0.10	10.33	0.31	1.836 down	2.084 down	0.02	13.25	unique protein
111564	9.65	0.01	10.65	0.11	10.87	0.18	2.001 up	2.324 up	0.00	48.42	D-tyrosyl-tRNA deacylase
111566	8.25	0.08	4.45	0.02	7.15	0.26	13.910 down	2.134 down	0.00	183.82	unique protein
111567	11.27	0.02	7.00	0.00	8.72	0.24	19.198 down	5.836 down	0.00	258.98	C2H2 transcription factor
111570	10.18	0.06	9.19	0.02	8.62	0.10	1.982 down	2.951 down	0.00	220.01	short chain dehydrogenase/reductase
111584	3.80	0.07	3.99	0.25	5.22	0.11	1.145 up	2.679 up	0.00	83.23	unknown protein

111591	8.86	0.10	8.55	0.20	6.76	0.43	1.242 down	4.288 down	0.00	31.12	unknown protein
111593	3.12	0.34	3.22	0.02	5.24	0.11	1.069 up	4.337 up	0.00	141.78	unique protein
111632	8.71	0.07	9.33	0.01	10.71	0.28	1.541 up	3.999 up	0.00	64.18	unknown protein
111638	10.42	0.03	11.79	0.00	9.41	0.16	2.574 up	2.018 down	0.00	239.32	unknown protein
111645	4.89	0.25	5.95	0.13	6.21	0.14	2.085 up	2.497 up	0.00	43.65	unique protein
111663	9.81	0.17	10.30	0.10	10.86	0.12	1.400 up	2.074 up	0.00	46.38	unknown protein
111674	2.72	0.11	2.73	0.05	4.38	0.09	1.007 up	3.142 up	0.00	356.92	unique protein
111682	8.86	0.18	8.00	0.07	7.78	0.12	1.813 down	2.116 down	0.00	49.24	unknown protein
111729	3.08	0.11	3.15	0.12	4.40	0.07	1.049 up	2.506 up	0.00	212.13	HFBs
111731	6.08	0.08	6.30	0.10	4.83	0.23	1.164 up	2.379 down	0.00	54.77	unique protein
111733	3.39	0.32	3.12	0.21	4.96	0.10	1.207 down	2.955 up	0.00	80.91	GH92 α-1.2-mannosidase
111735	5.70	0.51	5.55	0.10	6.91	0.19	1.106 down	2.314 up	0.01	22.54	unique protein
111736	8.34	0.17	9.60	0.22	9.44	0.29	2.401 up	2.150 up	0.01	15.57	unique protein
111750	10.50	0.01	10.85	0.06	14.86	0.08	1.277 up	20.527 up	0.00	3531.03	ferric reductase
111755	10.29	0.07	9.92	0.06	8.83	0.10	1.291 down	2.742 down	0.00	221.86	transcriptional regulator. unknown
111758	9.61	0.08	9.06	0.11	10.91	0.39	1.464 down	2.467 up	0.01	27.98	unknown protein
111776	9.98	0.05	10.51	0.05	8.06	0.48	1.452 up	3.775 down	0.00	35.58	Zn2Cys6 transcriptional regulator
111778	7.80	0.06	10.81	0.05	8.96	0.36	8.063 up	2.238 up	0.00	59.93	unknown protein
111788	11.50	0.09	10.82	0.07	10.41	0.06	1.608 down	2.132 down	0.00	169.70	Synaptobrevin/VAMP-like protein (VAMP71 family)
111807	6.56	0.26	7.78	0.14	5.39	0.41	2.321 up	2.247 down	0.00	32.25	unknown protein
111808	3.83	0.22	4.23	0.04	5.42	0.21	1.312 up	3.002 up	0.00	55.41	unknown protein
111811	4.13	0.03	6.53	0.16	5.70	0.28	5.260 up	2.968 up	0.00	58.70	unique protein
111827	3.82	0.03	3.77	0.04	4.92	0.18	1.035 down	2.137 up	0.00	65.08	unknown protein
111832	11.53	0.06	10.63	0.00	10.36	0.14	1.854 down	2.241 down	0.00	71.31	Isoflavone reductase superfamily protein
111833	2.31	0.11	2.28	0.06	4.09	0.11	1.015 down	3.442 up	0.00	297.59	MFS permease
111838	8.80	0.18	8.15	0.12	6.74	0.32	1.572 down	4.166 down	0.00	44.24	serin endopeptidase
111849	9.08	0.03	7.99	0.15	10.12	0.08	2.121 down	2.055 up	0.00	362.84	GH30 endo-β-1.4-xylanase XYN4
111858	3.64	0.14	3.54	0.10	5.42	0.10	1.078 down	3.423 up	0.00	278.70	methyltransferase
111861	3.37	0.15	4.22	0.40	5.21	0.31	1.803 up	3.599 up	0.01	24.81	PTH11 GPCR
111874	7.22	0.04	4.60	0.29	5.63	0.24	6.170 down	3.012 down	0.00	67.63	swollenin 2

111875	11.89	0.05	10.96	0.02	10.24	0.35	1.909 down	3.131 down	0.01	24.95	unknown protein
111884	4.89	0.03	5.37	0.29	6.49	0.26	1.391 up	3.018 up	0.00	34.10	unknown protein
111888	10.13	0.10	10.10	0.14	8.98	0.21	1.025 down	2.228 down	0.00	39.98	MFS permease
111901	6.63	0.00	6.17	0.06	5.50	0.09	1.368 down	2.178 down	0.00	180.00	unknown proteins
111915	10.02	0.20	13.43	0.08	13.79	0.23	10.684 up	13.684 up	0.00	250.57	SSCRP
111923	6.77	0.02	8.73	0.21	11.07	0.42	3.901 up	19.749 up	0.00	114.50	unknown protein
111937	10.64	0.03	11.04	0.09	11.70	0.32	1.316 up	2.075 up	0.02	13.18	unique protein
111948	5.81	0.30	5.37	0.33	7.14	0.32	1.356 down	2.520 up	0.01	25.21	unique protein
111951	13.55	0.27	9.15	0.07	12.22	0.40	21.223 down	2.518 down	0.00	95.89	unique protein
111953	9.58	0.02	9.04	0.15	8.48	0.14	1.454 down	2.142 down	0.00	49.90	GH47 α-1.2-mannosidase
111955	8.19	0.10	7.53	0.12	9.58	0.63	1.589 down	2.613 up	0.02	13.23	unknown protein. 1 TM
111956	3.59	0.33	3.61	0.12	4.98	0.37	1.014 up	2.628 up	0.01	17.72	unknown protein
111958	3.71	0.02	3.87	0.00	5.47	0.23	1.111 up	3.370 up	0.00	85.79	unknown protein
111966	4.11	0.13	3.86	0.19	6.06	0.11	1.186 down	3.869 up	0.00	241.15	short chain dehydrogenase/reductase
111968	9.32	0.16	9.30	0.04	5.66	0.61	1.011 down	12.623 down	0.00	57.43	unknown protein. only in ascomycetes
112001	11.29	0.13	10.12	0.04	9.51	0.18	2.252 down	3.446 down	0.00	90.10	unknown protein
112002	8.54	0.08	7.98	0.04	9.93	0.12	1.473 down	2.624 up	0.00	275.86	SSCRP
112028	2.97	0.13	3.47	0.11	5.19	0.12	1.416 up	4.666 up	0.00	276.85	terpene synthase
112030	3.93	0.10	3.86	0.23	5.74	0.41	1.052 down	3.490 up	0.00	30.25	unique protein
112034	2.98	0.06	2.92	0.24	4.59	0.15	1.036 down	3.052 up	0.00	106.76	unknown protein
112037	3.28	0.00	3.36	0.16	5.03	0.06	1.059 up	3.377 up	0.00	402.53	SSCRP
112049	6.57	0.18	3.30	0.57	5.20	0.23	9.679 down	2.578 down	0.00	52.54	unique protein
112055	3.33	0.43	3.42	0.16	4.50	0.12	1.064 up	2.249 up	0.01	24.94	unique protein
112064	9.97	0.29	10.06	0.12	8.92	0.20	1.064 up	2.073 down	0.01	27.56	unique protein
112080	9.62	0.04	9.86	0.14	8.44	0.08	1.180 up	2.264 down	0.00	216.08	unknown protein
112081	12.34	0.23	11.55	0.07	10.27	0.18	1.738 down	4.201 down	0.00	99.15	Methionine synthase. vitamin-B12 independent. putative
112083	6.96	0.10	5.66	0.16	8.40	0.60	2.453 down	2.723 up	0.01	23.60	HET protein
112086	3.65	0.14	5.00	0.35	6.04	0.16	2.542 up	5.252 up	0.00	87.50	unknown protein
112094	8.35	0.05	7.61	0.02	6.88	0.23	1.676 down	2.767 down	0.00	44.70	unique protein
112113	3.19	0.09	3.40	0.39	4.58	0.11	1.152 up	2.611 up	0.00	42.13	unknown protein

112124	5.10	0.35	4.82	0.27	7.39	0.40	1.219 down	4.884 up	0.00	43.13	HFBs
112125	10.30	0.11	8.50	0.08	8.58	0.43	3.475 down	3.278 down	0.01	19.58	macrophomate synthase
112126	12.76	0.00	12.29	0.09	11.62	0.01	1.388 down	2.204 down	0.00	486.22	Methylmalonate semialdehyde dehydrogenase
112129	4.67	0.11	5.00	0.61	5.71	0.13	1.254 up	2.060 up	0.03	9.43	Zn2Cys6 transcriptional regulator
112133	2.98	0.12	3.07	0.14	5.09	0.24	1.064 up	4.317 up	0.00	102.06	unknown protein
112134	9.69	0.19	9.12	0.02	7.52	0.35	1.483 down	4.514 down	0.00	45.80	Zn2Cys6 transcriptional regulator
112140	4.08	0.33	3.64	0.23	8.33	0.38	1.359 down	18.991 up	0.00	169.30	GH28 exo-polygalacturonase PGX1
112163	7.40	0.07	6.74	0.16	8.85	0.19	1.586 down	2.729 up	0.00	126.20	unknown protein in Sordariomycetes
112180	11.93	0.09	11.27	0.00	10.06	0.06	1.573 down	3.638 down	0.00	672.74	unknown protein
112193	12.23	0.12	12.27	0.10	10.93	0.51	1.031 up	2.465 down	0.02	11.03	unknown protein
112211	12.37	0.06	12.19	0.05	11.34	0.15	1.131 down	2.038 down	0.00	62.12	exonuclease. putative
112215	4.97	0.22	6.19	0.22	6.32	0.12	2.337 up	2.548 up	0.00	47.00	unique protein
112222	6.75	0.26	5.94	0.36	5.40	0.26	1.752 down	2.550 down	0.01	15.32	unknown protein
112232	3.95	0.31	4.91	0.28	5.20	0.29	1.944 up	2.375 up	0.02	12.47	unique protein
112238	3.19	0.18	3.38	0.05	4.60	0.09	1.142 up	2.660 up	0.00	151.97	unique protein
112239	13.14	0.07	5.45	0.08	6.68	0.25	206.465 down	87.701 down	0.00	888.55	unknown protein
112247	11.69	0.04	8.42	0.04	8.14	0.48	9.643 down	11.725 down	0.00	64.34	3-isopropylmalate dehydrogenase
112255	2.50	0.04	2.78	0.15	4.02	0.08	1.219 up	2.863 up	0.00	231.25	unique protein
112267	11.17	0.15	10.28	0.08	9.55	0.32	1.846 down	3.066 down	0.01	26.34	unknown protein
112275	9.78	0.01	9.65	0.04	7.91	0.16	1.093 down	3.655 down	0.00	201.85	unique protein
112276	11.70	0.15	11.19	0.22	9.19	0.15	1.427 down	5.681 down	0.00	186.68	unique protein
112281	9.64	0.02	5.55	0.26	7.48	0.26	16.952 down	4.461 down	0.00	153.11	unique protein
112285	8.59	0.20	7.09	0.08	7.20	0.30	2.821 down	2.624 down	0.01	23.83	unknown protein
112288	5.03	0.06	4.84	0.16	6.85	0.42	1.141 down	3.536 up	0.00	33.14	unknown protein
112328	12.78	0.04	12.09	0.02	10.86	0.27	1.612 down	3.779 down	0.00	60.30	peptidase M14
112330	12.52	0.08	12.27	0.09	11.04	0.42	1.190 down	2.780 down	0.01	17.11	transcriptional regulator. putative
112338	4.14	0.56	4.33	0.15	7.00	0.86	1.140 up	7.254 up	0.01	14.93	unique protein
112369	2.52	0.07	2.83	0.21	4.62	0.35	1.242 up	4.281 up	0.00	45.88	unique protein
112370	3.56	0.06	3.65	0.22	4.93	0.15	1.069 up	2.586 up	0.00	74.64	unique protein
112386	3.25	0.22	3.27	0.41	5.38	0.23	1.011 up	4.348 up	0.00	59.21	unknown protein

112387	4.89	0.28	4.43	0.30	6.19	0.57	1.376 down	2.463 up	0.02	10.83	unknown protein with WD repeats
112390	14.26	0.08	13.69	0.05	12.74	0.24	1.487 down	2.876 down	0.00	48.53	unknown protein
112392	4.90	0.08	4.95	0.22	6.93	0.37	1.037 up	4.085 up	0.00	43.65	GH11 endo-ß-1.4-xylanase XYN5
112396	4.41	0.33	4.98	0.32	7.15	0.16	1.484 up	6.680 up	0.00	104.95	unique secreted protein
112398	8.75	0.22	7.77	0.02	7.14	0.17	1.968 down	3.059 down	0.00	65.58	unique protein
112400	10.88	0.11	10.43	0.18	9.66	0.18	1.362 down	2.321 down	0.00	38.43	unique protein
112401	11.01	0.04	10.72	0.23	9.46	0.24	1.224 down	2.921 down	0.00	45.13	Zn2Cys6 transcriptional regulator
112427	2.29	0.12	2.40	0.03	3.95	0.09	1.075 up	3.158 up	0.00	319.84	unique protein
112439	14.58	0.08	13.73	0.05	12.04	0.20	1.813 down	5.854 down	0.00	188.11	unknown protein
112441	9.99	0.14	10.78	0.01	8.93	0.18	1.728 up	2.077 down	0.00	101.71	Vacuolar sorting protein Vps52
112459	7.67	0.13	7.43	0.28	6.10	0.48	1.180 down	2.970 down	0.02	13.73	unique protein
112477	4.05	0.45	4.35	0.13	5.81	0.36	1.228 up	3.370 up	0.01	21.45	unknown protein
112478	6.50	0.07	6.10	0.03	10.14	0.85	1.325 down	12.441 up	0.00	34.31	unknown protein. only in A. oryzae
112490	7.17	0.22	6.82	0.14	5.71	0.39	1.276 down	2.755 down	0.01	16.31	unknown protein
112494	3.81	0.17	3.90	0.36	5.18	0.14	1.066 up	2.581 up	0.00	39.68	unknown protein
112496	9.60	0.06	10.00	0.15	7.73	0.22	1.319 up	3.655 down	0.00	131.96	heterocompatibility domain protein
112502	11.83	0.01	10.86	0.04	10.06	0.20	1.955 down	3.398 down	0.00	90.68	unknown protein
112514	6.13	0.40	6.65	0.08	7.33	0.20	1.440 up	2.308 up	0.01	17.69	unique protein
112518	3.54	0.12	3.73	0.42	5.68	0.18	1.138 up	4.409 up	0.00	72.78	unique protein
112519	10.33	0.08	9.99	0.13	8.90	0.32	1.264 down	2.695 down	0.01	24.53	vacuolar endopolyphosphatase
112520	8.73	0.18	9.13	0.11	10.19	0.15	1.314 up	2.734 up	0.00	73.31	unique protein
112523	6.38	0.15	6.27	0.22	7.86	0.65	1.075 down	2.781 up	0.03	8.80	unknown protein
112532	8.32	0.03	8.36	0.06	7.01	0.40	1.029 up	2.471 down	0.01	18.22	bHLH transcriptional regulator
112535	4.79	0.13	6.23	0.21	6.01	0.31	2.712 up	2.327 up	0.01	18.14	unique protein
112536	11.57	0.01	10.77	0.25	10.19	0.30	1.749 down	2.604 down	0.01	19.50	unknown protein
112538	6.90	0.09	5.41	0.02	7.95	0.16	2.815 down	2.072 up	0.00	247.82	C2H2 transcription factor
112539	6.70	0.02	5.29	0.14	5.51	0.27	2.661 down	2.286 down	0.01	25.71	Zn2Cys6 transcriptional regulator
112551	10.57	0.05	9.67	0.07	8.97	0.15	1.870 down	3.024 down	0.00	110.40	unknown protein
112559	2.56	0.11	2.73	0.01	4.30	0.15	1.126 up	3.351 up	0.00	172.72	unknown protein
112560	10.09	0.28	9.71	0.05	8.35	0.20	1.298 down	3.333 down	0.00	60.94	Zn2Cys6 transcriptional regulator

112563	3.53	0.05	3.64	0.03	5.96	0.28	1.078 up	5.371 up	0.00	114.40	α-ketoglutarate dependent (FeII) dioxygenase
112578	4.55	0.30	4.19	0.22	5.65	0.21	1.287 down	2.136 up	0.00	30.96	unknown protein
112580	2.76	0.04	2.78	0.05	4.54	0.19	1.015 up	3.443 up	0.00	143.16	unknown protein
112596	11.18	0.15	10.64	0.08	8.98	0.11	1.448 down	4.583 down	0.00	291.51	flavoprotein monooxygenase
112602	2.47	0.06	2.36	0.01	3.85	0.03	1.078 down	2.595 up	0.00	1398.90	unknown protein
112603	2.26	0.20	2.42	0.10	3.71	0.06	1.121 up	2.732 up	0.00	153.62	unique protein
112604	2.29	0.17	2.36	0.06	3.80	0.10	1.049 up	2.849 up	0.00	171.94	unique protein
112605	2.16	0.17	2.21	0.09	3.54	0.09	1.033 up	2.608 up	0.00	154.51	unique protein
112629	5.06	0.64	5.42	0.08	6.60	0.49	1.284 up	2.916 up	0.04	8.46	unique protein
112631	5.90	0.15	6.80	0.25	7.58	0.20	1.875 up	3.216 up	0.00	46.98	unique protein
112638	3.49	0.27	3.57	0.24	5.04	0.34	1.056 up	2.926 up	0.01	23.73	unknown protein
112649	2.20	0.08	2.44	0.23	3.94	0.06	1.184 up	3.356 up	0.00	194.92	unknown protein
112651	2.58	0.08	2.55	0.05	3.70	0.03	1.020 down	2.173 up	0.00	513.28	unknown protein
112656	8.57	0.03	7.53	0.25	7.27	0.18	2.052 down	2.474 down	0.00	36.25	unique protein
112665	9.82	0.02	7.25	0.16	7.18	0.28	5.943 down	6.267 down	0.00	99.23	GCN5-related N-acetyltransferase
112675	2.43	0.02	2.45	0.24	4.53	0.25	1.007 up	4.269 up	0.00	90.23	unknown protein
112676	1.98	0.18	2.08	0.14	3.48	0.10	1.074 up	2.829 up	0.00	134.89	unique protein
112678	2.42	0.07	2.46	0.07	3.65	0.15	1.030 up	2.347 up	0.00	90.78	unique protein
112679	2.45	0.08	2.47	0.13	4.09	0.10	1.014 up	3.120 up	0.00	247.52	unique protein
112683	2.21	0.14	2.28	0.19	3.59	0.07	1.047 up	2.594 up	0.00	124.38	unknown protein
112685	2.53	0.02	2.72	0.18	4.30	0.10	1.141 up	3.407 up	0.00	214.57	unknown protein
112688	2.72	0.11	2.84	0.13	4.16	0.41	1.086 up	2.713 up	0.01	17.91	unique protein
112689	2.52	0.26	2.59	0.35	4.34	0.15	1.054 up	3.548 up	0.00	62.28	unknown protein
112695	3.71	0.40	3.73	0.24	5.01	0.13	1.011 up	2.453 up	0.00	30.54	SSCRP
119534	13.98	0.01	13.69	0.06	12.64	0.17	1.220 down	2.526 down	0.00	79.21	unique protein
119552	8.94	0.05	9.19	0.08	10.38	0.07	1.189 up	2.725 up	0.00	383.62	unique protein
119556	11.42	0.03	9.96	0.01	10.03	0.55	2.752 down	2.620 down	0.04	8.39	unknown protein. secreted
119568	11.23	0.17	10.96	0.04	9.39	0.28	1.206 down	3.590 down	0.00	55.13	unknown protein
119607	12.48	0.04	11.67	0.09	10.95	0.10	1.762 down	2.907 down	0.00	191.47	unknown protein
119619	10.33	0.13	8.28	0.15	8.86	0.44	4.165 down	2.780 down	0.01	18.34	unknown protein

119620	11.04	0.02	10.62	0.11	9.84	0.30	1.343 down	2.295 down	0.01	18.47	Zn2Cys6 transcriptional regulator
119710	12.91	0.01	10.98	0.08	10.81	0.13	3.821 down	4.284 down	0.00	257.88	MFS multidrug transporter
119723	12.63	0.01	12.57	0.29	11.60	0.28	1.044 down	2.050 down	0.01	15.65	NSF attachment protein Sec17/alpha-SNAP
119789	13.09	0.12	12.64	0.18	11.62	0.49	1.369 down	2.762 down	0.02	10.73	MFS permease
119800	11.17	0.04	12.14	0.04	12.17	0.10	1.953 up	2.000 up	0.00	113.19	unknown protein
119805	13.14	0.20	13.44	0.16	14.20	0.29	1.232 up	2.096 up	0.02	14.34	HFBs
119806	12.18	0.05	12.97	0.00	13.60	0.10	1.723 up	2.677 up	0.00	200.54	unknown protein. only in Gibberella. Magnaporthe and Chaetomium
119816	11.20	0.12	11.84	0.10	12.45	0.36	1.562 up	2.392 up	0.02	12.97	unknown protein
119823	13.22	0.04	12.51	0.15	11.79	0.19	1.635 down	2.686 down	0.00	54.82	SAM-dependent methyltransferases
119825	3.61	0.19	3.93	0.00	5.31	0.49	1.242 up	3.237 up	0.01	15.96	unknown protein
119826	11.37	0.25	10.07	0.03	7.13	0.62	2.461 down	18.872 down	0.00	55.71	C2H2 transcriptional regulator
119839	3.87	0.60	3.02	0.17	5.44	0.35	1.805 down	2.966 up	0.00	29.04	cyanamide hydratase
119856	12.24	0.22	12.23	0.02	11.22	0.23	1.007 down	2.034 down	0.01	25.56	E3 ubiquitin-protein ligase/Putative upstream regulatory element binding protein
119857	7.15	0.06	11.56	0.00	10.25	0.26	21.231 up	8.567 up	0.00	258.13	unique protein
119860	10.86	0.08	11.37	0.02	9.71	0.35	1.423 up	2.222 down	0.01	27.66	unknown protein
119864	9.25	0.02	6.08	0.25	6.95	0.32	9.027 down	4.933 down	0.00	74.84	unknown protein
119876	11.86	0.20	12.00	0.07	12.88	0.12	1.103 up	2.027 up	0.00	50.03	aspartyl protease
119895	11.86	0.07	11.91	0.13	10.61	0.32	1.040 up	2.374 down	0.01	24.19	unknown protein
119896	11.85	0.01	11.47	0.04	9.97	0.28	1.306 down	3.683 down	0.00	61.71	malic enzyme
119902	12.69	0.11	10.43	0.05	11.41	0.25	4.795 down	2.418 down	0.00	65.44	unique protein
119960	9.28	0.14	9.61	0.23	10.48	0.28	1.254 up	2.295 up	0.01	18.07	unique protein
119963	12.77	0.05	13.04	0.09	13.80	0.05	1.202 up	2.031 up	0.00	244.10	HFBs
119989	13.80	0.03	13.75	0.12	14.81	0.14	1.034 down	2.015 up	0.00	74.14	HFB2
120008	12.11	0.28	13.17	0.14	13.12	0.27	2.084 up	2.017 up	0.02	12.86	GMC oxidoreductase
120031	12.53	0.11	13.82	0.02	14.12	0.30	2.450 up	3.009 up	0.00	31.30	unknown protein
120060	12.38	0.08	12.22	0.10	11.37	0.21	1.112 down	2.008 down	0.00	29.67	unknown protein with pleckstrin like domain
120079	11.02	0.19	10.35	0.16	9.64	0.26	1.595 down	2.608 down	0.01	25.40	mitochondrial acetoacetyl-CoA thiolase-like protein
120088	10.22	0.12	10.60	0.10	12.10	0.31	1.302 up	3.685 up	0.00	46.15	cytosolic asparaginyl-tRNA synthetase. required for protein synthesis. catalyzes the sp
120106	11.44	0.10	12.44	0.28	12.85	0.26	1.999 up	2.648 up	0.01	22.23	Isopentenyl diphosphate:dimethylallyl diphosphate isomerase
120113	11.19	0.00	10.66	0.12	10.15	0.18	1.444 down	2.062 down	0.00	34.96	unknown protein

120125	12.13	0.12	11.78	0.20	10.85	0.14	1.277 down	2.433 down	0.00	56.09	unknown protein
120160	12.49	0.09	13.00	0.37	13.74	0.10	1.428 up	2.378 up	0.00	31.86	unknown protein
120172	12.14	0.06	11.77	0.16	11.04	0.41	1.285 down	2.139 down	0.04	8.31	flavodoxin and radical SAM domain protein
120198	13.70	0.17	13.30	0.01	12.32	0.18	1.323 down	2.604 down	0.00	57.56	glycosyl transferase. family 35. glycogen phosphorylase 1
120229	7.18	0.12	5.04	0.56	13.25	0.80	4.392 down	67.461 up	0.00	117.81	GH10 endo-ß-1.4-xylanase XYN3
120248	10.10	0.11	8.90	0.03	9.06	0.44	2.296 down	2.051 down	0.04	7.61	unknown protein
120260	8.99	0.01	9.28	0.06	10.18	0.39	1.219 up	2.267 up	0.02	12.22	ribosomal protein L24e. putative
120272	11.02	0.12	10.84	0.07	9.96	0.14	1.138 down	2.083 down	0.00	59.65	64 kDa mitochondrial NADH dehydrogenase. putative
120294	5.83	0.19	6.52	0.41	6.98	0.07	1.606 up	2.216 up	0.01	20.21	unknown protein
120311	10.57	0.15	8.77	0.14	12.83	0.22	3.486 down	4.803 up	0.00	321.21	unique protein
120312	10.86	0.21	6.74	0.10	14.81	0.26	17.338 down	15.462 up	0.00	846.37	GH5 endo-β-1.4-glucanase EGL2/CEL5a
120326	10.77	0.15	10.21	0.05	8.72	0.29	1.472 down	4.129 down	0.00	59.77	unknown protein
120351	9.69	0.17	10.45	0.11	10.71	0.14	1.691 up	2.020 up	0.00	36.34	unknown protein
120363	7.04	0.14	7.25	0.16	5.93	0.56	1.151 up	2.172 down	0.04	7.72	C2H2 transcriptional regulator
120365	11.07	0.12	10.04	0.11	9.78	0.33	2.044 down	2.434 down	0.01	15.71	BZIP transcriptional regulator
120370	12.72	0.05	13.33	0.05	13.74	0.02	1.519 up	2.024 up	0.00	501.09	unknown protein
120371	13.55	0.12	6.22	0.16	10.49	0.85	161.771 down	8.375 down	0.00	61.55	Catalase
120381	11.41	0.09	12.85	0.06	8.69	0.45	2.719 up	6.564 down	0.00	104.43	unique protein
120415	10.57	0.02	12.80	0.11	11.67	0.11	4.687 up	2.154 up	0.00	273.94	unknown protein
120451	6.67	0.10	7.57	0.07	8.38	0.30	1.872 up	3.275 up	0.00	34.67	unknown protein
120473	8.00	0.01	8.22	0.08	9.09	0.28	1.170 up	2.137 up	0.01	21.12	Dihydrolipoamide transacylase (alpha-keto acid dehydrogenase E2 subunit)
120475	13.16	0.02	12.41	0.02	11.91	0.16	1.685 down	2.386 down	0.00	65.47	C2H2 transcriptional regulator
120486	9.09	0.09	9.79	0.01	11.21	0.24	1.624 up	4.346 up	0.00	91.20	auxiliary protein of DNA polymerase delta
120489	12.66	0.06	11.43	0.06	11.08	0.04	2.331 down	2.972 down	0.00	698.58	unknown protein
120504	9.93	0.06	8.58	0.07	13.18	0.28	2.557 down	9.472 up	0.00	323.72	unique protein
120571	9.68	0.17	10.36	0.12	12.03	0.55	1.596 up	5.082 up	0.01	22.57	pre-rRNA-processing protein PNO1
120597	14.15	0.09	12.62	0.07	11.06	0.18	2.887 down	8.540 down	0.00	286.58	C2H2 transcriptional regulator
120623	14.26	0.09	13.16	0.07	13.05	0.30	2.154 down	2.316 down	0.01	18.21	unknown protein
120688	5.26	0.28	5.84	0.19	6.66	0.28	1.495 up	2.635 up	0.01	20.20	unique protein
120697	11.88	0.15	13.65	0.12	14.05	0.18	3.428 up	4.518 up	0.00	125.05	SSCRP

120702	9.03	0.30	9.29	0.03	11.27	0.08	1.202 up	4.743 up	0.00	206.28	unknown protein
120715	11.33	0.17	10.27	0.17	10.31	0.23	2.085 down	2.028 down	0.01	18.08	Zn2Cys6 transcriptional regulator
120733	10.99	0.09	11.55	0.01	12.86	0.21	1.470 up	3.649 up	0.00	98.31	mitochondrial import inner membrane translocase subunit TIM13 / Zn-finger. Tim10/[illegible]
120749	13.19	0.01	11.91	0.08	14.50	0.06	2.428 down	2.475 up	0.00	1411.91	GH1 ß-glucosidase BGL2/CEL1a
120751	12.33	0.09	11.32	0.08	11.18	0.13	2.015 down	2.226 down	0.00	75.50	unknown protein
120752	11.71	0.08	11.23	0.02	9.96	0.17	1.396 down	3.362 down	0.00	129.33	K+ transporter Trk1
120784	12.80	0.12	12.12	0.09	11.33	0.12	1.608 down	2.766 down	0.00	108.24	cell wall mannoprotein
120791	11.95	0.07	12.45	0.25	13.03	0.35	1.410 up	2.105 up	0.03	9.32	unknown protein
120823	13.71	0.13	14.20	0.12	14.79	0.03	1.409 up	2.117 up	0.00	113.10	cell wall mannoprotein
120828	11.92	0.11	12.23	0.43	13.21	0.36	1.240 up	2.442 up	0.02	11.44	unknown protein
120872	11.24	0.11	9.96	0.01	9.47	0.44	2.434 down	3.420 down	0.01	17.82	GCN5 N-acetyltransferase
120873	4.82	0.29	4.73	0.18	8.28	0.26	1.069 down	11.000 up	0.00	191.27	GH71 α-1 3-glucanase
120889	11.83	0.28	12.17	0.07	10.75	0.38	1.271 up	2.110 down	0.01	15.46	cytochrome P450. putative
120911	12.54	0.03	10.88	0.12	9.44	0.18	3.158 down	8.588 down	0.00	300.30	short chain dehydrogenase/reductase
120926	6.16	0.00	4.14	0.41	7.47	0.56	4.050 down	2.468 up	0.00	33.19	unique protein
120927	5.16	0.18	6.13	0.16	7.84	0.76	1.963 up	6.440 up	0.01	14.82	ankyrin containing protein
120931	7.20	0.15	10.08	0.13	10.84	0.20	7.346 up	12.502 up	0.00	275.08	unknown protein
120961	8.65	0.10	3.31	0.15	13.06	0.69	40.524 down	21.244 up	0.00	218.45	GH61 polysaccharide monooxygenase CEL61b
120968	6.19	0.65	4.64	0.42	9.96	0.23	2.928 down	13.631 up	0.00	143.64	Copper chaperone for superoxide dismutase
120969	10.53	0.00	11.16	0.08	11.64	0.04	1.548 up	2.148 up	0.00	354.23	NADPH oxidase regulator NoxR
120978	13.23	0.03	13.39	0.29	14.24	0.14	1.121 up	2.011 up	0.00	29.96	large ribosomal subunit (protein L34e).
120981	11.07	0.07	10.66	0.03	9.43	0.29	1.324 down	3.117 down	0.00	42.51	magnesium and cobalt transporter CorA
120993	4.37	0.44	3.99	0.15	6.23	0.22	1.306 down	3.637 up	0.00	58.22	unknown protein
121042	8.57	0.50	9.87	0.05	11.49	0.47	2.457 up	7.562 up	0.00	32.25	unknown protein
121058	3.75	0.31	3.92	0.01	4.92	0.09	1.119 up	2.244 up	0.00	49.28	unknown protein
121095	11.22	0.04	10.98	0.10	9.89	0.26	1.186 down	2.528 down	0.00	36.27	cell cycle control protein (Cwf19). putative
121107	11.66	0.05	11.94	0.13	10.55	0.20	1.213 up	2.152 down	0.00	56.60	Zn2Cys6 transcriptional regulator
121121	9.74	0.05	10.22	0.04	10.88	0.38	1.399 up	2.196 up	0.03	10.51	Zn2Cys6 transcriptional regulator
121127	10.11	0.01	6.03	0.25	12.36	0.46	17.003 down	4.756 up	0.00	189.98	GH3 β-xylosidase BXL1
121130	8.65	0.16	8.68	0.12	7.54	0.44	1.021 up	2.166 down	0.03	10.48	Zn2Cys6 transcriptional regulator

121135	2.40	0.04	3.51	0.56	3.83	0.10	2.155 up	2.695 up	0.01	20.25	SSCRP
121171	8.76	0.21	9.82	0.07	9.89	0.37	2.084 up	2.196 up	0.03	9.86	replication fork protection component Swi3
121187	11.13	0.12	9.56	0.17	9.66	0.12	2.968 down	2.772 down	0.00	98.83	unknown protein
121203	11.80	0.02	11.19	0.14	10.77	0.24	1.527 down	2.032 down	0.01	18.13	unknown protein
121226	10.37	0.15	11.73	0.03	11.46	0.47	2.555 up	2.130 up	0.04	7.90	unknown protein
121230	12.08	0.04	12.21	0.10	13.33	0.32	1.100 up	2.390 up	0.01	22.86	unique protein
121294	11.82	0.19	11.08	0.13	10.59	0.29	1.670 down	2.337 down	0.01	16.22	glucan endo-1.3(4)-β-D-glucosidase
121295	13.13	0.04	12.85	0.24	11.98	0.16	1.210 down	2.214 down	0.00	37.63	Dolichol kinase Sec59
121308	12.31	0.04	13.04	0.22	13.37	0.13	1.666 up	2.084 up	0.00	37.60	PutA delta-1-pyrroline-5-carboxylate dehydrogenase
121315	4.02	0.48	4.44	0.05	5.78	0.25	1.335 up	3.384 up	0.00	29.29	unknown protein
121337	5.24	0.27	4.61	0.24	6.26	0.34	1.552 down	2.022 up	0.01	20.66	unknown protein
121372	12.83	0.02	12.95	0.04	14.24	0.04	1.084 up	2.660 up	0.00	1177.99	60S ribosomal protein L38 by homology with corresponding proteins in other fungi an
121397	12.35	0.37	12.30	0.06	13.39	0.19	1.036 down	2.050 up	0.01	22.62	Sec61alpha subunit
121405	12.11	0.10	10.11	0.02	8.67	0.26	4.002 down	10.879 down	0.00	190.87	4-aminobutyrate aminotransferase
121412	10.15	0.00	12.32	0.08	11.64	0.14	4.497 up	2.811 up	0.00	204.64	Zn2Cys6 transcriptional regulator
121415	8.47	0.06	12.02	0.13	13.35	0.16	11.737 up	29.400 up	0.00	834.80	Zn2Cys6 transcriptional regulator
121417	4.74	0.18	4.32	0.56	5.95	0.17	1.340 down	2.317 up	0.01	24.46	unknown protein with fasciclin domain
121439	12.27	0.22	10.67	0.07	10.88	0.30	3.040 down	2.622 down	0.01	25.39	unknown protein
121441	12.83	0.05	13.11	0.36	11.10	0.22	1.208 up	3.323 down	0.00	63.12	carbounknown proteinlic acid transporter
121464	12.11	0.01	12.35	0.07	13.80	0.14	1.181 up	3.226 up	0.00	186.05	GcvHGlycine cleavage system H protein (lipoate-binding)
121474	12.36	0.07	11.85	0.09	10.33	0.39	1.420 down	4.098 down	0.00	35.59	Zn2Cys6 transcriptional regulator
121486	11.63	0.07	7.94	0.18	8.78	1.35	12.952 down	7.206 down	0.05	7.13	unknown protein
121495	4.55	0.24	3.80	0.05	7.79	0.18	1.677 down	9.463 up	0.00	430.05	Vacuolar proteinase B (yscB). a serine protease of the subtilisin family
121498	12.43	0.09	8.82	0.01	10.99	0.27	12.234 down	2.704 down	0.00	152.30	Phosphatidylserine decarboxylase
121516	13.61	0.04	13.96	0.04	14.70	0.09	1.279 up	2.128 up	0.00	148.78	histone H2B
121579	11.15	0.04	11.75	0.13	12.18	0.15	1.519 up	2.047 up	0.00	43.56	unknown protein
121594	6.92	0.04	4.35	0.39	5.42	0.14	5.975 down	2.831 down	0.00	78.88	unique protein
121602	12.22	0.03	11.99	0.07	10.88	0.09	1.168 down	2.527 down	0.00	249.24	Zn2Cys6 transcriptional regulator
121608	11.32	0.18	10.10	0.03	8.39	0.28	2.331 down	7.628 down	0.00	111.92	MFS permease
121638	11.27	0.14	9.76	0.04	10.16	0.38	2.859 down	2.162 down	0.02	13.96	unique protein

121664	7.66	0.12	7.63	0.20	8.93	0.51	1.022 down	2.411 up	0.03	9.97	Glutamate decarboxylase and related proteins
121693	12.49	0.18	11.28	0.00	10.92	0.14	2.324 down	2.982 down	0.00	91.88	Glutathione-dependent formaldehyde-activating. GFA
121735	10.35	0.09	12.11	0.07	12.74	0.46	3.392 up	5.256 up	0.00	30.06	GH3 β-glucosidase CEL3b
121741	12.65	0.02	13.06	0.27	13.67	0.16	1.323 up	2.025 up	0.01	25.48	Ribosomal protein S7
121743	12.98	0.20	13.10	0.04	11.76	0.59	1.088 up	2.331 down	0.04	7.69	Mitochondrial succinate/fumarate antiporter
121746	10.83	0.07	11.79	0.14	12.79	0.48	1.952 up	3.896 up	0.01	18.19	GH55 exo-1 3-β-glucanase GLUC78
121773	10.87	0.03	11.79	0.11	9.81	0.57	1.896 up	2.074 down	0.02	13.67	unknown protein
121785	10.15	0.02	5.84	0.13	9.01	0.21	19.861 down	2.194 down	0.00	357.38	ATP-dependent RNA helicase
121800	13.18	0.10	12.38	0.13	11.86	0.17	1.748 down	2.510 down	0.00	52.17	stomatin-like protein
121804	10.73	0.07	11.02	0.01	12.03	0.23	1.225 up	2.470 up	0.00	42.84	Ribosomal protein S15.
121824	11.53	0.16	11.37	0.18	12.87	0.30	1.118 down	2.540 up	0.00	31.83	ATP citrate lyase. alpha subunit
121864	11.79	0.12	11.70	0.10	10.16	0.30	1.059 down	3.088 down	0.00	42.20	unknown protein
121870	10.48	0.16	8.58	0.04	9.20	0.13	3.727 down	2.435 down	0.00	128.32	unknown protein
121877	10.63	0.07	8.40	0.32	8.74	0.09	4.705 down	3.710 down	0.00	113.70	epoxide hydrolase
121883	10.62	0.25	13.39	0.20	12.84	0.23	6.827 up	4.673 up	0.00	89.26	unique secreted protein
121906	12.31	0.11	12.81	0.11	13.33	0.18	1.414 up	2.027 up	0.00	28.94	40s ribosomal protein S14 (S11 family).
121944	13.08	0.04	12.90	0.02	11.98	0.23	1.128 down	2.144 down	0.00	32.29	serine/threonine protein kinase
121948	11.13	0.07	11.98	0.33	12.74	0.18	1.810 up	3.070 up	0.00	42.02	signal peptidase spc12
121968	11.79	0.07	11.02	0.01	10.64	0.20	1.714 down	2.219 down	0.00	35.18	serine peptidase S28
121989	12.21	0.01	12.04	0.12	11.05	0.22	1.126 down	2.243 down	0.00	38.75	oxalate decarboxylase
122007	9.81	0.02	11.27	0.00	12.07	0.12	2.737 up	4.789 up	0.00	366.67	unique protein
122040	12.31	0.12	12.55	0.09	13.34	0.08	1.178 up	2.036 up	0.00	102.68	ribosomal protein L31e.
122048	11.56	0.05	12.03	0.37	13.40	0.28	1.382 up	3.580 up	0.00	36.35	Sec61 beta subunit
122081	8.69	0.23	7.09	0.06	13.76	0.40	3.019 down	33.524 up	0.00	332.44	GH7 Endo-β-1.4-glucanase EGL1/CEL7b
122089	10.66	0.22	7.76	0.24	9.05	0.47	7.444 down	3.053 down	0.01	26.94	unknown protein
122091	12.42	0.00	11.60	0.00	11.04	0.04	1.767 down	2.592 down	0.00	1206.45	phospholipase of papatin-family
122096	5.80	0.30	6.49	0.16	8.68	0.50	1.613 up	7.382 up	0.00	38.73	unknown protein
122102	9.89	0.02	9.79	0.03	8.85	0.20	1.067 down	2.049 down	0.00	42.05	unknown protein
122104	10.48	0.21	9.95	0.23	9.24	0.29	1.447 down	2.368 down	0.01	16.05	Xanthine/uracil permease family
122113	13.45	0.10	13.78	0.01	14.53	0.08	1.257 up	2.126 up	0.00	145.86	unknown protein

122127	13.76	0.11	14.42	0.06	14.78	0.25	1.574 up	2.024 up	0.01	16.94	SSCRP
122140	12.07	0.21	11.95	0.26	10.69	0.49	1.085 down	2.592 down	0.03	10.38	unique protein
122160	9.13	0.18	10.01	0.12	6.87	0.29	1.845 up	4.787 down	0.00	128.41	Heat shock proteinwith DnaJ domain
122169	11.58	0.16	11.30	0.08	10.57	0.20	1.214 down	2.011 down	0.01	27.05	Histidine kinase. part of a two component signal transduction system
122212	12.50	0.13	12.15	0.11	11.37	0.25	1.276 down	2.188 down	0.01	22.84	vacuolar protein-sorting protein BRO1
122240	13.30	0.03	13.09	0.07	12.16	0.24	1.159 down	2.210 down	0.00	29.68	Carnitine o-acyltransferase
122278	9.28	0.10	9.14	0.06	10.33	0.18	1.099 down	2.065 up	0.00	54.12	U3 small nucleolar RNA associated protein (SOF1)
122284	14.05	0.07	13.54	0.05	12.66	0.09	1.420 down	2.615 down	0.00	220.23	vel1. velvet protein
122293	11.06	0.08	11.01	0.09	9.58	0.16	1.035 down	2.797 down	0.00	118.68	unknown protein
122350	12.43	0.05	11.98	0.04	10.61	0.21	1.366 down	3.530 down	0.00	94.48	Glutamate decarboxylase
122363	8.91	0.06	7.44	0.07	10.23	0.31	2.768 down	2.497 up	0.00	89.98	Hsp26/Hsp42
122366	10.77	0.13	10.55	0.04	9.69	0.19	1.161 down	2.117 down	0.00	38.05	unknown protein
122374	11.12	0.03	13.66	0.05	13.94	0.43	5.802 up	7.071 up	0.00	49.66	MRSP1/expansin-like
122422	9.27	0.13	12.00	0.07	11.46	0.21	6.607 up	4.563 up	0.00	143.26	SSCRP
122448	7.53	0.06	7.88	0.04	10.44	0.07	1.273 up	7.482 up	0.00	1764.05	C2H2 transcription factor
122499	8.17	0.03	7.31	0.51	9.71	0.16	1.816 down	2.904 up	0.00	63.03	unknown protein
122505	12.45	0.04	10.91	0.08	10.41	0.09	2.914 down	4.134 down	0.00	473.05	aryl-alcohol dehydrogenase
122526	6.72	0.12	6.77	0.17	9.27	0.26	1.039 up	5.874 up	0.00	126.86	unknown protein
122529	10.64	0.12	11.90	0.23	11.96	0.50	2.395 up	2.493 up	0.04	7.62	S1/P1 Nuclease
122541	12.54	0.01	12.30	0.07	10.99	0.21	1.175 down	2.915 down	0.00	76.12	C2H2 transcriptional regulator
122556	7.37	0.20	10.21	0.23	11.18	0.90	7.167 up	13.985 up	0.01	19.08	Short-chain dehydrogenase/reductase
122567	13.64	0.27	12.79	0.04	12.57	0.21	1.795 down	2.102 down	0.01	19.33	unknown protein
122572	13.14	0.01	13.00	0.18	14.35	0.10	1.105 down	2.299 up	0.00	129.62	hsp70 family protein
122579	9.39	0.06	11.87	0.05	11.69	0.44	5.546 up	4.905 up	0.00	36.15	unknown protein. 4TM
122614	11.35	0.16	10.20	0.07	9.58	0.31	2.211 down	3.394 down	0.00	31.82	unique protein
122629	7.92	0.12	9.97	0.01	9.72	0.49	4.133 up	3.481 up	0.01	18.37	unique protein
122745	8.89	0.07	9.77	0.22	10.58	0.07	1.845 up	3.234 up	0.00	142.63	2-oxoisovalerate dehydrogenase subunit beta. putative
122778	12.17	0.04	9.23	0.02	10.73	0.07	7.686 down	2.730 down	0.00	1319.23	short chain dehydrogenase/reductase
122780	11.31	0.04	13.46	0.10	13.91	0.27	4.450 up	6.086 up	0.00	99.32	GH28 exo-rhamnogalacturonase RGX1
122795	9.60	0.26	8.99	0.10	8.15	0.31	1.517 down	2.720 down	0.01	19.98	PTH11 GPCR

122813	10.24	0.11	11.33	0.01	12.23	0.44	2.128 up	3.983 up	0.01	22.96	unique protein
122819	10.36	0.14	11.24	0.18	12.30	0.27	1.837 up	3.820 up	0.00	49.29	DNA replication complex GINS protein PSF3
122820	8.77	0.14	4.27	0.15	7.06	0.88	22.692 down	3.281 down	0.01	22.01	Kynurenine aminotransferase. glutamine transaminase K
122824	6.85	0.25	3.43	0.13	11.99	0.62	10.764 down	35.206 up	0.00	212.69	PTH11 GPCR
122825	10.74	0.09	9.70	0.06	8.79	0.32	2.051 down	3.847 down	0.00	40.84	unknown protein
122870	10.96	0.12	12.03	0.03	12.80	0.38	2.089 up	3.578 up	0.01	24.87	cell wall protein. CwpA
122889	8.01	0.14	2.83	0.17	6.59	0.33	36.228 down	2.689 down	0.00	195.06	unknown protein
122941	9.63	0.11	8.78	0.01	11.38	0.25	1.806 down	3.349 up	0.00	129.57	unique secreted protein with CFEM domain
122974	11.25	0.14	10.95	0.01	10.07	0.24	1.233 down	2.272 down	0.00	29.73	unknown protein
122992	9.74	0.08	8.52	0.10	11.09	0.23	2.333 down	2.545 up	0.00	126.36	GT 31 ß-glycosyltransferase
122994	12.28	0.17	11.93	0.00	11.25	0.11	1.279 down	2.043 down	0.00	61.14	unknown protein
122995	8.00	0.04	8.16	0.17	6.75	0.55	1.115 up	2.386 down	0.03	9.42	unknown protein
123009	12.53	0.02	14.10	0.06	13.90	0.23	2.980 up	2.582 up	0.00	48.38	glutamine synthetase
123019	11.07	0.05	10.70	0.09	9.89	0.20	1.298 down	2.272 down	0.00	41.46	unknown protein
123029	13.53	0.10	13.11	0.20	10.84	0.35	1.339 down	6.480 down	0.00	75.95	Cu2+/Zn2+ superoxide dismutase SOD1
123039	12.74	0.09	12.70	0.20	13.93	0.06	1.031 down	2.278 up	0.00	130.26	HFBs
123079	11.06	0.07	13.99	0.02	14.34	0.22	7.591 up	9.716 up	0.00	244.91	short chain dehydrogenase/reductase
123084	9.38	0.02	8.38	0.11	8.34	0.27	1.995 down	2.052 down	0.01	16.78	chloroperoxidase
123086	6.32	0.11	6.77	0.12	13.89	0.15	1.367 up	190.175 up	0.00	2760.75	SSCRP
123095	6.80	0.30	8.33	0.03	5.43	0.42	2.880 up	2.588 down	0.00	46.56	unique protein
123120	10.85	0.28	11.11	0.07	12.01	0.18	1.200 up	2.241 up	0.00	30.89	unknown protein. 8 TM
123149	9.34	0.07	7.99	0.34	7.72	0.21	2.559 down	3.075 down	0.00	36.14	unknown protein
123180	10.82	0.10	11.15	0.06	11.94	0.33	1.257 up	2.173 up	0.02	14.43	Seryl-tRNASynthetase. class IIa.
123191	12.70	0.05	12.66	0.11	14.06	0.12	1.029 down	2.562 up	0.00	165.16	unknown protein
123199	9.46	0.08	11.23	0.02	12.00	0.56	3.404 up	5.813 up	0.01	22.62	SSCRP
123204	12.33	0.08	13.99	0.03	13.74	0.05	3.159 up	2.647 up	0.00	646.36	lipase/esterase
123205	5.51	0.02	5.47	0.26	6.97	0.23	1.029 down	2.750 up	0.00	47.08	unknown protein
123207	12.43	0.09	14.15	0.05	13.93	0.11	3.296 up	2.828 up	0.00	206.34	unknown protein
123226	12.64	0.12	10.99	0.08	9.60	0.27	3.145 down	8.238 down	0.00	130.71	GH37 α.α-trehalase
123227	9.49	0.03	9.24	0.13	8.12	0.15	1.187 down	2.591 down	0.00	92.98	unknown protein

123232	10.75	0.20	6.77	0.05	14.31	0.26	15.789 down	11.782 up	0.00	784.21	GH12 endo-ß-1.4-glucanase
123236	7.67	0.06	7.96	0.16	9.26	0.41	1.222 up	3.011 up	0.01	19.97	SSCRP
123256	7.44	0.13	8.56	0.08	8.61	0.24	2.168 up	2.251 up	0.01	25.23	unknown protein
123262	10.49	0.17	9.91	0.03	8.48	0.23	1.494 down	4.007 down	0.00	82.06	unique protein. HTG. amidase domain. 1 TM
123265	10.92	0.08	11.77	0.01	12.14	0.40	1.801 up	2.330 up	0.03	10.28	unknown protein
123274	12.85	0.29	10.35	0.08	10.70	0.04	5.664 down	4.439 down	0.00	201.71	Aldehyde dehydrogenase
123278	4.56	0.35	5.46	0.34	6.17	0.35	1.875 up	3.054 up	0.01	14.69	unknown protein
123282	8.45	0.13	7.38	0.30	6.72	0.27	2.097 down	3.305 down	0.00	30.89	unknown protein
123325	9.39	0.19	10.46	0.03	10.74	0.23	2.108 up	2.548 up	0.00	31.56	zinc transporter
123396	7.30	0.23	7.27	0.26	8.97	0.53	1.023 down	3.179 up	0.01	14.81	unknown protein
123441	9.28	0.01	10.54	0.33	10.49	0.33	2.384 up	2.308 up	0.02	12.78	unknown protein
123455	9.11	0.08	9.06	0.03	7.76	0.37	1.035 down	2.549 down	0.01	21.40	unknown protein
123473	11.89	0.04	9.96	0.02	8.04	0.15	3.810 down	14.364 down	0.00	761.02	MFS permease
123475	14.92	0.06	14.31	0.13	13.02	0.55	1.527 down	3.709 down	0.02	14.47	cell wall Thr-rich mannoprotein. Distantly related to S. cerevisiae Dan4p.
123476	8.74	0.04	8.61	0.06	11.31	0.34	1.094 down	5.959 up	0.00	98.04	unknown protein
123493	12.52	0.11	12.42	0.10	10.81	0.28	1.073 down	3.276 down	0.00	55.30	Ubiquitin-protein ligase (E3)
123509	9.77	0.07	10.37	0.05	7.79	0.36	1.506 up	3.955 down	0.00	68.32	Zn2Cys6 transcriptional regulator
123510	10.55	0.21	10.14	0.11	7.46	0.53	1.330 down	8.529 down	0.00	46.60	Zn2Cys6 transcriptional regulator
123553	8.78	0.09	10.25	0.15	9.88	0.40	2.779 up	2.147 up	0.02	11.79	short chain dehydrogenase/reductase
123572	10.71	0.04	9.31	0.05	9.52	0.25	2.634 down	2.278 down	0.00	32.50	Phospholipase A2
123604	12.96	0.07	13.26	0.28	13.99	0.23	1.224 up	2.042 up	0.01	16.69	Rps24 (family S16) by homology with similar proteins in other fungi.
123608	7.21	0.05	7.67	0.04	8.59	0.19	1.373 up	2.603 up	0.00	65.47	unique protein
123616	13.85	0.08	14.84	0.18	15.10	0.12	1.984 up	2.365 up	0.00	60.77	short unique protein
123636	12.27	0.10	11.48	0.07	10.86	0.25	1.722 down	2.658 down	0.00	33.73	unknown protein
123639	3.68	0.23	6.03	0.38	5.07	0.25	5.100 up	2.625 up	0.00	37.05	GH64 endo-1.3-β-glucanase
123649	9.84	0.06	9.04	0.11	7.87	0.35	1.749 down	3.910 down	0.00	36.97	unique protein
123659	13.76	0.02	9.39	0.02	11.31	0.07	20.748 down	5.487 down	0.00	3163.03	cell wall protein. istantly related to S. cerevisiae Pir3p.
123695	9.22	0.06	9.99	0.26	10.71	0.11	1.705 up	2.807 up	0.00	68.68	unknown protein
123697	13.70	0.00	12.30	0.03	11.36	0.10	2.644 down	5.059 down	0.00	611.63	Unknown protein
123723	12.27	0.20	12.16	0.10	10.87	0.62	1.085 down	2.645 down	0.04	7.59	Arylacetamide deacetylase

123732	10.92	0.04	9.08	0.12	8.72	0.16	3.583 down	4.606 down	0.00	181.52	unique protein
123740	13.80	0.18	13.29	0.15	12.60	0.20	1.430 down	2.305 down	0.00	29.28	unknown protein
123779	9.12	0.04	9.77	0.05	7.70	0.25	1.577 up	2.671 down	0.00	88.00	unique protein
123805	11.77	0.08	11.32	0.04	10.43	0.12	1.360 down	2.531 down	0.00	138.39	DHBP_synthase.-dihydroxy--butanone -phosphate synthase
123806	11.98	0.00	10.38	0.10	10.07	0.43	3.020 down	3.750 down	0.01	21.98	GPCR. secretin like
123818	11.69	0.07	7.14	0.01	13.01	0.61	23.487 down	2.491 up	0.00	104.73	GH11 endo-ß-1.4-xylanase XYN2
123827	14.33	0.04	13.73	0.03	12.88	0.31	1.521 down	2.737 down	0.01	26.72	bifunctional catalase/peroxidase
123850	13.38	0.04	13.73	0.12	14.50	0.10	1.270 up	2.171 up	0.00	107.42	60s acid ribosomal protein P1 based on homology to corresponding proteins in fungi a
123865	11.33	0.22	13.67	0.03	12.34	0.45	5.058 up	2.021 up	0.01	20.81	Peptidase S8 and S53. subtilisin. kexin. sedolisin
123888	12.51	0.07	11.04	0.13	10.04	0.27	2.767 down	5.528 down	0.00	83.91	unknown protein
123940	4.84	0.37	3.14	0.19	10.18	1.69	3.232 down	40.577 up	0.01	22.82	GH115 methylglurunoyl esterase CIP2
123955	9.64	0.23	13.88	0.14	12.72	0.16	18.904 up	8.468 up	0.00	330.87	Epl1/Sm1
123967	13.79	0.07	14.14	0.06	14.96	0.16	1.272 up	2.252 up	0.00	60.58	HFB3
123968	12.70	0.20	14.32	0.04	14.05	0.07	3.090 up	2.561 up	0.00	140.23	unknown protein
123989	14.28	0.04	11.50	0.12	15.59	0.09	6.841 down	2.485 up	0.00	1418.75	GH7 Cellobiohydrolase CBH1/CEL7a
123992	11.22	0.10	9.33	0.06	13.13	0.65	3.714 down	3.757 up	0.00	38.57	swollenin
123999	7.03	0.26	8.21	0.10	8.27	0.21	2.266 up	2.361 up	0.01	26.76	NADH:flavin oxidoreductase/NADH oxidase
124022	9.18	0.01	6.09	0.20	10.20	0.22	8.500 down	2.036 up	0.00	297.26	Zn2Cys6 transcriptional regulator
124030	10.88	0.02	10.08	0.01	9.77	0.38	1.742 down	2.166 down	0.03	9.47	unknown protein with TIM barrel
124043	3.51	0.39	3.82	0.23	6.63	0.23	1.241 up	8.695 up	0.00	122.71	GH18. chitinase CHI18-14
124059	12.76	0.03	10.05	0.12	13.82	0.05	6.566 down	2.088 up	0.00	2235.49	SSCRP
124065	9.38	0.17	8.53	0.23	12.11	0.39	1.804 down	6.623 up	0.00	98.19	unknown protein. only in bacteria; contains large nuclear transport factor 2 domain
124079	3.15	0.17	3.60	0.27	4.60	0.31	1.365 up	2.734 up	0.01	21.12	Multicopper oxidases
124104	12.47	0.05	12.16	0.09	11.17	0.29	1.247 down	2.473 down	0.01	25.86	unknown protein
124115	13.54	0.15	12.82	0.13	11.26	0.08	1.640 down	4.849 down	0.00	350.81	phosphoenolpyruvate carboxykinase AcuF
124116	10.52	0.02	10.85	0.29	9.46	0.18	1.254 up	2.082 down	0.00	42.80	unknown protein
124119	11.53	0.12	11.32	0.07	9.61	0.26	1.157 down	3.788 down	0.00	73.41	unique protein
124157	13.20	0.03	13.27	0.05	11.47	0.13	1.051 up	3.312 down	0.00	309.69	unknown protein
124170	10.02	0.05	9.59	0.04	8.34	0.25	1.352 down	3.206 down	0.00	59.19	arsenite methyltransferase
124177	13.23	0.08	12.48	0.11	11.86	0.31	1.683 down	2.586 down	0.01	21.10	unknown protein

124187	9.90	0.07	10.12	0.15	11.25	0.14	1.166 up	2.551 up	0.00	90.56	ER-associated protein degradation
124210	13.23	0.01	13.42	0.11	14.65	0.21	1.144 up	2.673 up	0.00	62.30	histone H3
124222	13.50	0.18	13.08	0.11	11.42	0.24	1.336 down	4.232 down	0.00	82.95	CaaX-protease. related to E. nidulans rce1. involved in signal transduction
124228	12.36	0.05	11.82	0.03	10.59	0.11	1.455 down	3.406 down	0.00	300.48	GT2 chitin synthase
124246	9.39	0.13	9.85	0.08	10.41	0.28	1.375 up	2.022 up	0.02	14.02	unknown protein
124249	10.65	0.17	11.83	0.09	12.52	0.38	2.271 up	3.665 up	0.01	24.77	unknown protein
124256	10.29	0.12	11.18	0.14	12.41	0.41	1.855 up	4.359 up	0.00	30.22	phospholipase-like protein
124260	9.65	0.04	9.48	0.07	8.35	0.20	1.123 down	2.464 down	0.00	61.37	Zn2Cys6 transcriptional regulator
124278	11.40	0.01	12.33	0.04	12.90	0.28	1.907 up	2.834 up	0.00	32.90	unknown protein with EXS domain
124283	8.92	0.11	10.62	0.00	11.80	0.27	3.244 up	7.355 up	0.00	123.52	unknown protein. SET and MYND domains
124286	12.19	0.09	11.84	0.07	10.97	0.21	1.274 down	2.340 down	0.00	40.53	Heteromeric CCAAT factors
124295	11.87	0.00	10.23	0.20	13.46	0.18	3.114 down	3.010 up	0.00	262.78	SSCRP
124296	9.17	0.05	7.68	0.10	10.38	1.04	2.810 down	2.316 up	0.04	7.65	unique protein
124299	3.13	0.08	3.23	0.35	5.31	0.33	1.069 up	4.537 up	0.00	50.27	HET-domain protein with WD40 repeats
124302	12.96	0.02	12.54	0.02	11.69	0.10	1.335 down	2.406 down	0.00	203.45	response regulator receiver. distantly related to S. cerevisiae Ssk1p and S. pombe Mcs
124310	3.05	0.45	3.63	0.04	4.94	0.36	1.497 up	3.722 up	0.01	23.04	unknown protein
124338	11.12	0.01	8.97	0.02	8.17	0.19	4.453 down	7.751 down	0.00	270.02	unique protein
124339	5.73	0.16	6.59	0.08	7.43	0.24	1.818 up	3.250 up	0.00	48.41	unique protein
124341	11.80	0.08	12.45	0.01	9.85	0.77	1.561 up	3.879 down	0.01	15.16	mating protein MAT1-2-1

* Abbreviations: SEQ-ID, Trire2:number; S.D. standard deviation; "ratio" specifies the expression ratio vs the parent strain. Note that expression levels (but not the ratios) are given as log2 by the ArrayStar evaluation package

Table S3 Comparison of expression results from microarrays and qPCR for selected genes*

Trire2:	gene category	array results		p-value	qPCR results			
		Δlae1	*lae1OE*		*Δlae1*		*lae1OE*	
						S.D.		S.D.
5647	PTH	3.364 down	1.567 down	0.00	0.73	± 0.11	0.55	± 0.14
39587	PTH	155.090 down	2.883 down	0.05	0.03	± 0.02	0.11	± 0.04
62462	PTH	1.475 down	2.438 up	0.00	1.9	± 0.87	1.8	± 0.47
69904	PTH	4.888 down	1.125 up	0.00	1.3	± 0.67	1.3	± 0.17
76763	PTH	3.952 down	1.267 up	0.05	0.3	± 0.12	0.22	± 0.18
82041	PTH	4.287 down	1.305 down	0.04	< 0.1	ND	0.45	± 0.11
105224	PTH	2.763 down	4.166 down	0.07	0.15	± 0.08	0.18	± 0.09
110339	PTH	1.078 down	11.197 up	0.07	3.4	± 2.3	268	± 78.4
110744	PTH	5.100 down	1.514 down	0.00	0.15	± 0.11	1.4	± 0.12
121990	PTH	4.330 down	1.236 down	0.04	0.35	± 0.23	2.2	± 1.2
122824	PTH	10.764 down	35.206 up	0.00	0.22	± 0.04	81.3	± 33.4
112083	HET	2.453 down	2.723 up	0.01	< 0.1	ND	0.3	± 0.08
69187	HET	2.647 down	1.827 down	0.02	0.85	± 0.35	3	± 1.6
107071	HET	1.412 up	3.767 up	0.00	0.83	± 0.42	4.6	± 1.1
111494	HET	2.414 down	1.882 down	0.01	0.81	± 0.28	2.6	± 1.4
23171	NRPS	3.634 up	9.894 up	0.00	1.44	± 0.33	4.14	± 0.51

* values are means of at least 3 replica, S.D. standard deviation

Table S4 Global H3K9 and H3K4 methylation patterns in relation to regulation by LAE1*

protein ID	H3K9me3			H3K4me2			H3K4me3			ratio *[Δlae1]*	ratio *[lae1OE]*	Annotation
	WT	*Δlae1*	*lae1OE*	WT	*Δlae1*	*lae1OE*	WT	*Δlae1*	*lae1OE*			
1673				+	+	+	+	+	+			Eukaryotic translation initiation factor 4E (eIF-4E)
1679					+		+					unknown protein
1683				+	+	+	+	+	+			Rap-RAN GTPase activating protein tuberin
1692				+	+	+	+	+	+			ribosomal protein L23
1702				+	+	+	+	+	+			DNA polymerase V (phi)
1735				+	+	+	+	+	+			unknown protein
1737												unknown protein
1751												FAD monooxygenase
1766				+	+	+	+	+	+			unknown protein
1777				+	+	+	+	+	+			unknown protein
1818				+	+	+	+	+	+			unknown protein
1847				+	+	+	+	+	+			RNA polymerase II transcription elongation factor (Ctr9)
1857				+	+	+	+	+	+			histone H4
1879				+	+	+	+	+	+			unknown protein with dDENN domain
1885					+							GH15 glucamylase with starch binding domain
1912				+	+	+	+	+	+			unknown protein
1925				+	+	+	+	+	+			fatty acid hydroxylase. Cytb5. SUR2-type hydroxylase/desaturase. catalytic region
1927												unknown protein
1935				+	+	+	+	+	+			unknown protein with DENN domain
1941												myb transcriptional regulator
1947					+							unknown protein
1949												unknown protein
1958												unknown protein. tetratricopeptide repeats
1959										1.359 up	4.687 up	Haloacid dehalogenase-like hydrolase

1960									unknown protein
1977	+	+	+	+	+	+	1.106 down	2.191 down	Peptidase_M28
1983	+	+	+	+	+	+			RNA binding domain protein PUA
1992									metal dependent phosphohydrolase
1993	+		+				1.057 down	2.381 down	γ-glutamyl phosphate reductase GPR
1997	+	+	+	+	+	+			kinesin
1999	+	+	+	+	+	+			unknown protein
2015									GCN5-related N-acetyltransferase
2033							2.855 down	2.205 up	unknown protein
2036							1.090 down	2.856 up	cysteine peptidase
2038									Alcohol dehydrogenase. class IV
2044									dipeptidyl peptidase 5
2050	+	+	+	+	+	+			translation initiation factor eIF-4G
2068									MFS permease
2071	+	+	+	+	+	+	1.875 up	2.266 up	unknown protein. 5 TM
2076							13.496 down	8.504 down	malate permease
2087	+	+	+	+	+	+			exosome complex exonuclease RRP43
2089									Rad21/Rec8 like protein. N-terminal
2091	+	+	+	+	+	+	1.604 down	2.676 down	6-phosphofructokinase
2095	+	+	+	+	+	+			DNA polymerase delta. catalytic subunit
2096	+		+				1.637 up	3.429 down	Zn2Cys6 transcriptional regulator
2100		+							unknown protein DUF1531
2108	+	+	+	+	+	+			unknown protein
2125							1.146 up	2.029 down	Fructose-2.6-bisphosphatase

2148	+	+	+				2.133 down	2.651 down	Zn2Cys6 transcriptional regulator
2173	+	+	+	+		+			unknown protein
2185			+				1.764 up	7.251 up	GCN5-related N-acetyltransferase
2186									unknown protein
2200									short chain dehydrogenase/reductase
2211									Transketolase
2223							1.178 up	2.618 up	succinate-CoA ligase. alpha subunit
2231									unknown protein
2241	+	+	+	+	+	+			WASP-like pretein las17p
2250									GCN5-related N-acetyltransferase
2268	+	+	+	+	+	+			inner centromere protein
2269	+	+	+	+	+	+			condensin complex component SMC2
2316	+	+	+	+	+	+	3.965 down	4.082 down	unknown protein with caleosin domain
2319	+	+	+	+	+	+			unknown protein
2322							1.344 down	2.612 down	unknown protein
2343		+							unknown protein
2348									unknown protein
2358							1.269 down	2.915 down	unknown protein with HIT domain
2362									Zinc-containing alcohol dehydrogenase
2365		+		+					Unknown protein
2369									SNF2 family domain-containing protein
2373	+	+	+	+	+	+			unknown protein
2374	+	+	+	+	+	+			actin-like protein
2384	+	+	+	+	+	+			unknown protein
2392	+	+	+	+	+	+	1.209 down	4.288 down	peroxisomal hydratase-dehydrogenase-epimerase

2399	+	+	+	+	+	+	1.056 down	3.210 down	unknown protein
2433	+	+	+	+	+	+			methyltransferase
2439	+	+	+	+	+	+			Arc15 (N.crassa) ortholog
2441	+	+	+	+	+	+			unknown protein
2451	+	+	+	+	+	+			Secretion related small GTPase Rab6/Ypt6/Ryh1
2474	+	+	+	+	+	+			polyphosphoinositide phosphatase. putative
2476	+	+	+	+	+	+			vacuolar ATP synthase subunit D
2489							1.820 down	3.356 up	Ribonuclease T2
2492							1.832 down	4.378 down	Phosphatidylinositol-4-phosphate 5-kinase
2499							1.182 up	4.741 down	unknown protein
2501									pyrroline-5-carboxylate reductase
2508	+	+	+	+	+	+			chaperonine Cpn60/TCP1. t-complex protein t. subunit ß
2517							1.399 down	2.149 up	Flavoprotein monooxygenase. putative
2521									FAD dependent oxidoreductase
2529	+	+	+	+	+	+			unknown protein with a possible Interpro domain corresponding to ribosomal protein S12
2537	+	+	+	+	+	+			RHO protein GDP dissociation inhibitor
2540									MFS permease
2558	+	+	+	+	+	+			unknown protein
2561	+	+	+	+	+	+			Protein transport protein Sec1
2568	+	+	+	+	+	+			unknown protein
2570							1.448 up	2.929 up	short chain dehydrogenase/reductase
2574	+	+	+	+	+	+			glycerol-3-phosphate dehydrogenase. NAD-dependent
2583									Imidazoleglycerol-phosphate synthase subunit H
2591	+	+	+	+	+	+			UbiE-like methylase
2599	+	+	+	+	+	+			unknown protein
2629		+							methionine-R-sulfoxide reductase B2

2634	+	+	+	+	+	+			Erg27 3-keto steroid reductase
2648	+	+	+	+	+	+			Fe(II)/2-oxoglutarate-dependent diounknown proteingenase
2660	+	+	+	+	+	+			actin-related protein 2/3 complex subunit. putative
2662	+	+	+	+	+	+			GH47 α-1.2-mannosidase
2666									Sin4. RNA polymerase II Mediator complex subunit
2676	+	+	+	+	+	+			NADH-ubiquinone oxidoreductase 12 kDa subunit
2687	+	+	+				4.071 down	3.173 down	AAA ATPase
2698	+	+	+						translocator protein
2703	+	+	+	+	+	+	1.074 up	2.372 down	unknown protein
2707									Cullin family protein
2716	+	+	+	+	+	+			RNA 3'-terminal phosphate cyclase
2721	+	+	+	+	+	+			unknown protein
2730							1.024 down	2.171 down	unknown protein
2735									GH18 chitinase CHI18-6
2745	+	+	+	+	+	+	1.115 down	2.175 down	fumarate hydratase-like protein
2759	+	+	+	+	+	+			HECT-domain-containing protein
2776									acetate non-utilizing protein 9. putative
2790		+							aminoacyl-tRNA synthase.
2826	+	+	+	+	+	+			SNF2-like helicase
2829	+	+	+	+	+	+			serine/threonine protein kinase
2830	+	+	+	+	+	+			unknown protein
2835	+	+	+	+	+	+	1.112 down	2.275 down	unknown protein
2837	+	+	+	+	+	+			unknown protein
2845	+	+	+	+	+	+			G-protein alpha subunit 2 GNA2
2852									unknown protein
2869	+	+	+	+	+	+			MPE1. zinc knuckle domain-containing protein MPE1 [Verticillium albo-atrum VaMs.102]

2902									short chain dehydrogenase/reductase
2912	+	+	+	+	+	+			3-oxo-5-alpha-steroid 4-dehydrogenase
2916	+	+	+	+	+	+			unknown protein
2956	+	+	+	+	+	+			HECT domain containing protein (E3 ubiquitin-protein ligase)
2981	+	+	+						citrate lyase. beta subunit
2987	+	+	+	+	+	+			cytoplasmic dynein intermediate chain (?)
3001	+	+	+	+	+	+			nucleolar protein 12
3007									transcriptional regulator HMG type
3027	+	+	+	+	+	+			Pso2 (Snm1). involved in DNA interstrand crosslink repair
3043							1.393 up	3.664 up	SSCRP
3049							1.898 down	7.341 down	methionine aminopeptidase
3055	+			+		+	22.757 down	2.485 down	short chain dehydrogenase/reductase
3063		+							unknown protein
3067	+	+	+	+	+	+			Chitin synthase export chaperone
3094							1.265 down	2.986 up	GH30 glucan endo 1.6-β-glucanase
3101	+	+	+	+	+	+			N-acetylglucosaminyl-phosphatidylinositol deacetylase. putative
3121							1.379 up	3.066 up	cytochrome P450 oxidoreductase OrdA-like
3129									alpha-tubulin suppressor protein Aats1
3196	+	+	+	+	+	+	1.138 down	3.171 down	GH38 α-mannosidase
3208	+	+	+	+	+	+			MMR; inhibition of homeologous recombination
3212	+	+	+	+	+	+			DNA replication licensing factor mcm7
3262							1.315 up	3.244 up	succinate semialdehyde dehydrogenase (NADP)
3267	+	+	+				2.019 up	3.543 down	MRSP1/expansin-like
3283									short-chain dehydrogenase/reductase
3287									Short-chain dehydrogenase/reductase
3292									short chain dehydrogenase/reductase

3310				+	+	+	+	+	+			C2H2 transcriptional regulator
3327						+						NADH:flavin oxidoreductase/NADH oxidase
3330												MFS permease
3333												Alcohol dehydrogenase zinc-binding domain protein
3340				+	+	+	+	+	+			t-SNARE syntaxin.cis-Golgi
3345				+	+	+	+	+	+			splicing factor 3B subunit 10
3350				+	+	+	+	+	+	1.265 up	2.002 down	phosphopantothenate-cysteine ligase
3351				+	+	+	+	+	+			unknown protein
3362				+	+	+	+	+	+			Phosphatidylinositol 3- and 4-kinase
3363												unknown protein. 9 TM
3364										1.768 down	4.386 down	Zinc-containing alcohol dehydrogenase
3372				+		+						IMP-specific 5'-nucleotidase
3374				+	+	+	+	+	+			unknown protein
3394				+	+	+	+	+	+			unknown protein
3397				+	+	+	+	+	+			unknown protein
3400				+	+	+	+	+	+			unknown protein
3405				+	+	+		+				MFS permease
3412												Na+/proline symporter PutP
3419				+	+	+	+	+	+			pre-mRNA-splicing factor SLU7
3422	+	+	+							1.166 up	4.207 up	unknown protein with oxidoreductase domain
3434					+	+						unknown protein with patatin domain
3447												NADP-dependent alcohol dehydrogenase (class V)
3449	+											Zn2Cys6 transcriptional regulator
3462						+				1.083 up	2.060 down	UreF urease accessory protein
3464				+	+	+	+	+	+			acyl-CoA dehydrogenase. putative
3481										1.236 down	2.211 down	unknown protein

3488	+	+	+	+			2.876 down	2.578 down	unknown protein
3501							1.227 down	2.035 down	unknown protein
3506									2OG-Fe(II) oxygenase superfamily protein
3508	+	+	+	+	+	+			unknown protein
3525	+	+	+	+	+	+			TRAPP complex componnet Trs31
3529							1.613 up	2.586 up	unknown protein
3532									MFS sugar permease
3568	+	+	+	+		+	5.337 down	3.682 down	phospholipase A2
3579	+	+	+	+	+	+	1.046 up	4.104 up	ATP-dependent RNA helicases.
3580		+							unknown protein
3591	+	+	+	+	+	+			Formyltetrahydrofolate hydrolase PurU
3596	+	+	+	+	+	+	1.470 down	2.050 up	uroporphyrinogen synthase
3600	+	+	+	+	+	+			Phosphatidylserine/phosphatidylglycerophosphate/cardiolipin synthase
3605	+	+	+	+	+	+	1.377 down	2.431 down	Zn2Cys6 transcriptional regulator
3641		+							Cystathionine beta-lyases/cystathionine gamma-synthases
3645	+	+	+						unknown protein with HIT domain
3653	+	+	+	+	+	+			2-oxoglutarate dehydrogenase component E2
3662		+							SGT1
3671	+	+	+	+	+	+			Gar1 protein with RNA-binding region
3707									unknown protein with TPR repeat
3716	+		+						L-PSP endoribonuclease family protein Brt1
3717									dihydrodipicolinate synthetase. putative
3718									unknown protein
3719									Flavonol reductase/cinnamoyl-CoA reductase
3739									GH43 β-xylosidase/α-L-arabinofuranosidase
3741		+							unknown protein

3765												MFS permease
3779				+	+	+	+	+	+			DNA polymerase subunit epsilon
3783				+	+	+	+	+	+			unknown protein
3787						+						unknown protein
3803				+	+	+	+	+	+			mRNA 3'-end-processing protein RNA14. putative
3813				+	+	+	+	+	+			unknown protein VanZ like family
3817				+	+	+	+	+	+			ER to golgi transport protein/RAD50-interacting protein 1.
3823												methionine synthase. vitamin-B12 independent
3824				+	+	+	+	+	+			HVA22 domain membrane protein. pathogenicity related
3830				+	+	+	+	+	+			MAPKK Bck-1 like MAP kinase kinase. involved in response to osmotic and temperature st
3835	+	+	+	+	+	+	+	+	+	1.002 down	3.172 down	3-ketoacyl-CoA thiolase
3847				+	+	+						unknown protein
3856				+	+	+	+	+	+			copper fist DNA-binding domain-containing protein
3873				+	+	+						cAMP phosphodiesterase class II PDE1. low affinity
3889				+	+	+	+	+	+	1.099 down	2.382 down	unknown protein
3891				+	+							unknown protein
3892				+	+	+	+	+	+			importin ß KapM
3909				+	+	+	+	+	+	1.710 up	2.528 up	Mitochondrial ribosomal protein L43
3914										11.485 down	14.492 down	Endoplasmic reticulum protein EP58
3918				+	+	+	+	+	+			unknown protein
3949				+	+	+	+	+	+			Myb. DNA-binding
3976				+	+	+	+	+	+			unknown protein
3984				+	+	+	+	+	+			increased rDNA silencing protein 4
3987				+	+	+	+	+	+			unknown protein. only in fungi
4004				+	+	+	+	+	+			FACT complex subunit pob3
4009				+	+	+	+	+	+			lactate/malate dehydrogenase
4027				+	+	+						GT25 b-glycosyltransferases

4040	+	+	+	+	+	+	1.353 down	2.187 down	RNA polymerase Rpc34 subunit
4064	+	+	+	+	+	+	1.267 up	3.025 up	Threonine dehydratase
4069	+	+	+	+	+	+	1.008 down	2.480 down	AMP-dependent synthetase and ligase
4097	+	+	+						unknown protein
4104									Arginase SpeB
4109	+	+	+				1.204 up	2.228 down	Aspartate/tyrosine/aromatic aminotransferase
4114							8.272 down	2.337 down	FAD linked oxidase
4117	+	+	+						alpha-aminoadipate reductase lys2
4124							19.177 up	11.315 up	myb transcriptional regulator
4146									flavoprotein monooxygenase
4152	+	+	+	+	+	+			GMP synthase GuA
4154	+	+	+	+	+	+			unknown protein
4170	+	+	+	+	+	+			unknown proteiun with WD repeats
4171							9.788 down	2.519 down	calcium transporter
4179									isoflavone reductase family protein
4196	+	+	+	+	+	+			Vacuolar ATP synthase 20 kDa proteolipid subunit
4213									ribonuclease T2
4221									unknown protein
4231	+	+	+	+	+	+			GATA type transcriptional regulator
4240							8.960 down	9.188 down	stress response protein Rds1; secreted
4244	+	+	+	+	+	+			unknown protein with DEAD/DEAH box helicase domain
4265	+	+	+	+	+	+			ribonuclease HI (RNaseH).
4284	+	+	+	+	+	+			ribosomal protein L3
4290	+	+	+	+	+	+			unknown protein
4294	+	+	+	+	+	+			pre-rRNA processing protein Rrp12

4308	+	+	+	+	+	+	1.717 down	2.021 down	Aminopeptidase N
4366									unknown protein
4370									ER membrane protein.
4373									glutaredoxin domain-containing protein
4421									unknown protein
4422	+	+	+	+	+	+			unknown protein
4426									unknown protein
4428	+	+	+	+	+	+	1.234 down	2.098 down	unknown protein. TPR domain
4430									Developmental regulatory protein WetA
4442	+						2.342 down	3.378 down	SAM-dependent methyltransferase
4454	+	+							unknown protein. secreted
4475									NAD dependent epimerase dehydratase family protein. putative
4479	+	+	+	+	+	+	1.597 up	2.318 up	small nuclear ribonucleoprotein Sm D2
4480	+	+	+	+	+	+			dihydroxacetione kinase Dak1
4484									GCN5-N-acetyltransferase activity
4494							1.224 down	3.085 down	unknown protein
4508	+	+	+	+	+	+			GPCR. related to N. crassa Stm1-like GPR-6
4514									SSCRP
4517							2.027 up	14.425 up	Cytochrome P450 CYP4/CYP19/CYP26 subfamilies
4526	+	+	+		+				unknown protein. only in ascomycota
4536	+	+	+	+	+	+			unknown protein
4537	+	+	+	+	+	+			alanyl-tRNA synthetase. class IIc.
4558	+	+	+	+	+	+			unknown protein
4561	+	+	+	+		+	2.718 down	2.569 down	GT α-1.6-mannosyltransferase
4592									F-box domain-containing protein
4622	+	+	+	+	+	+			thymidylate synthase

4626	+	+	+				2.627 down	5.010 down	unknown protein. C2 domain
4654	+	+	+	+	+	+			Histidinol dehydrogenase
4659	+	+	+	+	+	+			unknown protein
4663	+	+	+	+	+	+			unknown protein
4668	+	+	+	+	+	+			cytochrome c oxidase assembly protein
4677		+					1.045 down	2.710 down	unknown protein
4682	+	+	+	+	+	+			short chain dehydrogenase/reductase
4694	+	+	+	+	+	+			unknown protein
4705	+	+	+	+	+	+			Stress response protein UspA
4707	+	+	+	+	+	+			E3 ubiquitin-protein ligase. IQ and HECT domain
4721	+	+	+	+	+	+			mitochondrial Rho GTPase1
4726							1.022 down	2.629 down	Cytochrome P450 monooxygenase
4737							2.471 down	3.457 down	arylamine N-acetyltransferase 1
4744									transaldolase
4748	+	+	+	+	+	+	1.042 down	2.213 down	C2H2 transcription factor
4751	+	+	+	+	+	+			CDC24. rho-family GTPase
4767	+	+	+	+	+	+			biotin synthase
4774									MFS permease
4791		+	+	+	+	+			unknown protein
4843	+	+	+	+	+	+			Carbon-nitrogen hydrolase
4847	+	+	+	+	+	+	1.229 down	2.232 down	unknown protein
4851	+		+	+		+	194.650 down	3.832 down	unknown protein
4860	+	+	+	+	+	+			Siroheme synthase CysG
4862		+	+						unknown protein

4875	+	+	+	+	+	+	1.521 down	4.324 down	carboxy-cis.cis-muconate cyclase
4876	+	+	+	+			2.534 down	5.033 down	catechol dioxygenase
4878	+	+	+	+	+	+			ubiquinol-cytochrome c reductase complex 17 kd protein. putative
4885	+	+	+						Zn2Cys6 transcriptional regulator
4886									unknown protein
4901	+	+	+	+	+	+			cytochrome c oxidase assembly protein COX16. putative
4905	+	+	+		+		3.713 up	2.852 up	unknown protein
4913		+							WD repeat domain-containing phosphoinositide-interacting protein
4915	+	+	+	+	+	+			dynein light intermediate chain (DLIC).
4921	+	+	+				1.338 down	2.114 down	C2H2 transcriptional regulator
4933	+	+	+	+	+	+			bHLH transcriptional regulator
4936									unknown protein
4939		+							DNA repair and TFIIH regulator. required for both nucleotide excision repair and RNA poly
4941	+	+	+	+	+	+	1.301 down	7.840 down	phenylacetyl-CoA ligase.
4945	+	+	+	+	+	+	1.121 up	2.001 down	MAPKKK mitogen activated protein kinase kinase kinase Ste11
4947	+	+	+	+	+	+			TRAPP complex component Trs20
4950							1.291 down	2.153 down	urea transporter
4952							2.137 down	2.872 up	unknown protein
4981	+	+	+	+	+	+			dihydrounknown protein-acid dehydratase
4982	+	+	+	+	+	+			ATP-dependent Clp protease proteolytic subunit
4989	+	+	+	+	+	+			histone acetyltransferase
4990									short chain dehydrogenase/reductase
4996	+	+	+	+	+	+			unknown protein
4999							16.108 up	10.569 up	cytochrome P450 monooxygenase

5000							1.974 up	3.348 up	MFS permease
5007									unknown protein
5011	+	+	+	+	+	+			SSCRP
5013	+	+	+	+	+	+			unknown protein
5016			+						unknown secreted protein. only in Gibberella. Magnaporthe. Chaetomium and rats (!)
5026							2.634 up	3.969 up	SSCRP
5041	+	+	+	+	+	+			transcriptional regulator. unknown
5048	+	+	+	+	+	+			unknown protein
5058	+	+	+	+	+	+			unknown protein
5063	+	+	+	+	+	+	1.647 down	2.797 down	unknown protein
5064		+							RNA polymerase III subunitRpc25
5066	+	+	+	+	+	+			Signal peptidase complex. subunit Spc2. SPC25
5072	+	+	+	+	+	+	1.160 up	2.286 up	unknown protein
5084							5.928 down	19.550 down	conidiation-specific protein 10
5088									GCN5-related acetyltransferase
5091									Nitrilase/cyanide hydratase and apolipoprotein N-acyltransferase
5095	+	+	+	+	+	+			Pre-mRNA-splicing factor ISY1
5107									ACC deaminase [Trichoderma asperellum]
5112									short chain dehydrogenase/reductase
5119									ferrooxidoreductase
5127									casein kinase II. alpha subunit. involved in regulation of circadian clock in Neurospora cras
5135									D-arabinono-1.4-lactone oxidase
5140	+	+	+	+	+	+			Cwf15/Cwc15 cell cycle control
5148	+	+	+	+	+	+			Cullin (cell cycle)
5164	+	+	+	+	+	+			unknown protein
5182							6.435 down	4.438 down	iron-dependent peroxidase
5196	+	+					1.006 up	3.749 down	C-type cyclin

5202	+	+	+	+	+	+			unknown protein
5206	+		+						pyridine nucleotide-disulphide oxidoreductase AMID-like
5209	+	+	+	+	+	+			Secretion related small GTPase Rab11/Ypt3
5227	+	+	+	+	+	+			Isoleucine tRNA synthase.
5233	+	+	+	+	+	+			Aspartate/otherAminotransferase
5247			+						O6-methylguanine alkyltransferase
5249	+	+	+						fructosamine kinase
5250	+	+	+	+	+	+			unknown protein
5270	+	+	+						unknown protein
5275							1.024 up	3.633 down	unknown protein with SET domain
5278									Ras small GTPase.Rho type
5296			+				1.019 down	2.155 down	DHQase_I. Type I 3-dehydroquinase
5308							1.050 up	2.558 up	unknown protein
5319	+	+	+	+	+	+	1.747 down	4.094 down	unknown protein
5324	+	+	+	+	+	+	1.384 up	2.343 up	Ribosomal protein L14b
5330							1.011 down	2.195 up	glutathione S-transferase. putative
5337									γ-glutamyltranspeptidase
5345	+		+			+			FAD-containing oxidoreductase
5347									unknown protein
5350	+	+	+	+	+	+			SRP receptor. betaSubunit
5359									unknown protein. Duf1348
5363									unknown protein
5366							1.008 up	2.555 down	O-methyltransferase. putative
5368									short chain dehydrogenase/reductase
5369							1.250 down	5.811 up	Metallocarboxypeptidase. putative

5371									Prolyl 4-hydroxylase. alpha subunit
5377	+	+	+	+	+	+			Ethanolamine-P-transferase GPI11/PIG-F. involved in glycosylphosphatidylinositol anchor
5387	+	+	+	+	+	+			NADH-ubiquinone oxidoreductase 24 kDa subunit. mitochondrial
5400	+	+	+	+	+	+			unknown protein
5403	+	+	+	+	+	+			enoyl-CoA hydratase/isomerase
5407	+	+	+	+	+	+			unknown protein
5431	+	+	+	+	+	+			unknown protein
5436	+	+	+	+	+	+			unknown protein containing major histocompatibility complex
5446		+	+						unknown protein
5460		+							unknown protein
5466	+	+	+	+	+	+			high osmolarity signaling protein Sho1. putative
5502									unknown protein
5504	+	+							class I glutamine amidotransferase. putative
5506	+	+	+	+	+	+			RNA-binding ATP-dependent helicases.
5530	+	+	+	+	+	+	1.651 down	2.780 down	unknown protein
5536	+	+	+	+	+	+			ankyrin repeat-containing protein
5578	+	+	+						unknown protein
5598	+	+	+	+	+	+			Endosome-associated ubiquitin isopeptidase
5607									unknown protein
5610	+	+	+	+	+	+			half-sized ABC transporter
5612							2.028 down	2.858 down	MFS permease
5614									unknown protein
5633									unknown protein
5645	+	+	+	+	+	+			γ-glutamyltranspeptidase
5647									PTH11 GPCR
5651	+	+	+	+	+	+	3.222 down	3.328 down	unknown protein
5656							1.057 up	2.348 down	MFS permease

5659	+	+	+	+					pre-mRNA splicing factor. putative [Cryptococcus neoformans].
5664	+	+	+	+	+	+			transcriptional regulator APSES type
5675	+	+	+	+	+	+	1.130 down	2.106 down	C2H2 transcriptional regulator
5710	+	+	+	+	+	+			periodic tryptophan protein PWP2
5737							1.148 up	2.194 down	tyrosine/serine protein phosphatase. putative
5742		+	+						unknown protein
5771	+	+	+	+	+	+			Dual specificity phosphatase
5776	+	+	+	+	+	+	2.029 down	2.478 down	glucose-6-phosphate isomerase
5787									Amino acid transporters
5789									Cytochrome P450 CYP3/CYP5/CYP6/CYP9 subfamilies
5807							3.606 down	3.055 down	GH95 α-L-fucosidase
5812									short chain dehydrogenase/reductase
5818							2.086 down	2.049 down	DSBA oxidoreductase
5836							2.584 up	2.073 up	GH2 β-mannosidase
5844									unknown protein
5847	+	+	+	+	+	+			unknown protein
5849									unknown protein. Duf967
5850	+	+	+	+	+	+			GCD
5855									NmrA family protein
5863									enoyl-CoA hydratase/isomerase
5868	+	+	+	+	+	+			Single-stranded nucleic acid binding R3H
5871	+	+	+	+	+	+			unknown protein
5888							1.400 down	2.069 up	fatty-acid amide hydrolase. putative
5889							2.014 down	2.156 down	amidase

5890										1.177 down	2.908 up	MFS permease
5894												unknown protein
5898				+	+	+	+	+	+			unknown protein
5899				+	+	+	+	+	+			DNA replication licensing factor mcm2
5912				+	+	+	+	+	+	1.225 down	2.059 down	unknown protein
5916				+	+	+	+	+	+			unknown protein
5917				+	+	+	+	+	+			unknown protein
5924												unknown protein
5927				+								Zn2Cys6 transcriptional regulator
5942				+	+	+	+	+	+	1.845 down	3.665 down	unknown protein
5970										4.533 down	6.710 down	Amidase
5971												unknown protein
5979				+	+	+	+	+	+			integral membrane protein (Ptm1). putative
5991												importin ß KapN
6005												MFS permease
6011				+	+	+	+	+	+			unknown protein
6014				+	+	+	+	+	+			unknown protein
6015				+	+	+	+	+	+			RNA polymerase Rpb1
6037	+		+	+	+	+	+	+	+			unknown protein
6057				+	+	+	+	+	+			unknown protein
6067				+	+	+	+	+	+			unknown protein
6085										2.362 up	2.041 up	Enoyl-CoA hydratase/isomerase. putative
6096												fumarylacetoacetate hydrolase
6103										1.179 up	2.196 up	MFS multidrug transporter. putative
6107												ferric reductase
6108				+	+	+						SAM-dependent methyltransferase
21120												unknown protein

21129									unknown protein
21135	+	+	+	+	+	+			unknown protein
21150	+	+	+	+	+	+			unknown protein
21152	+	+	+	+	+	+	1.364 up	2.991 up	unknown protein
21166	+	+	+	+	+	+			Splicing factor motif
21168	+	+	+	+	+	+			MAPKK. MAP kinase kinase Mkk1
21170	+	+	+	+	+	+	1.440 up	2.300 up	Ribosomal protein S12
21174	+	+	+	+	+	+			myb transcriptional regulator
21176									Casein kinase (serine/threonine/tyrosine protein kinase)
21181	+	+	+	+	+	+			cell division cycle 37 protein. CDC37
21193	+	+	+	+	+	+			unknown protein
21198	+	+	+	+	+	+			cleavage and polyadenylation specificity factor subunit 5
21211	+	+	+	+	+	+			Forkhead
21214	+	+	+	+	+	+			Sorting nexin-41
21215	+	+	+	+	+	+			unknown protein
21221							1.044 up	2.038 down	unknown protein
21226									unknown protein
21246	+	+	+	+	+	+			Ubiquitin fusion degradation protein Ufd1
21249	+	+	+	+	+	+			unknown protein
21250	+	+	+	+	+	+			AAA family ATPase Rvb2/Reptin. putative
21255	+	+	+	+	+	+			bHLH transcriptional regulator
21256	+	+	+	+	+	+			protein phosphatase 2C
21270	+	+	+	+	+	+			CAP20 virulence factor
21278	+	+	+						unknown protein
21279	+	+	+	+	+	+			unknown protein
21288	+	+	+	+	+	+	1.035 up	2.182 down	Vacuolar segregation protein Pep7
21293	+	+	+	+	+	+			unknown protein
21294	+	+	+	+	+	+			rho2

21306	+	+	+	+	+	+			serine/threonine protein kinase PRP4
21324	+	+	+	+	+	+			protein phosphatase PP2A regulatory subunit A
21327									Esterase/lipase/thioesterase
21330	+	+	+	+	+	+			unknown protein
21342	+	+	+	+	+	+	1.056 up	2.204 down	TUL1 Golgi-localized RING-finger ubiquitin ligase (E3)
21345	+	+	+	+	+	+			unknown protein
21358	+	+	+	+	+	+			unknown protein
21363	+	+	+	+	+	+			Pseudouridylate synthase TruB
21373	+	+	+						germinal center kinase. related to S. cerevisiae Sps1p
21384	+	+	+	+	+	+			RNA-binding.Ran Zn-finger protein. probably involved inRegulation ofReceptor mediated t
21388	+	+	+	+	+	+			unknown protein
21392		+							Component. nce2. of non-classical secretion pathway; involved in secretion of proteins th
21396							1.497 down	2.263 down	sugar isomerase
21398	+	+	+	+	+	+			unknown protein
21406	+	+	+	+	+	+			phosphoglycerate kinase
21407									unknown protein
21412	+	+	+	+	+	+	2.118 down	2.000 down	unknown protein
21415	+	+	+	+	+	+	1.989 down	4.412 down	unknown protein
21416	+	+	+	+	+	+	1.698 down	3.251 down	unknown protein
21422	+	+	+	+	+		1.013 down	3.152 down	MFS permease
21425	+	+	+	+	+	+			DEAD/DEAH box helicase
21426	+	+	+	+	+	+			unknown protein
21433	+	+	+	+	+	+			C2H2 transcriptional regulator
21435									Orotate phosphoribosyl transferase
21437	+	+	+	+	+	+			Spc97 / Spc98 family. spindle pole body

21440		+	+	+	+	+	+			unknown protein
21441		+	+	+	+	+	+			peroxisomal targeting signal 2 receptor. putative
21442										unknown protein
21444		+	+	+	+	+	+			unknown protein
21450										aldehyde dehydrogenase
21452		+	+	+	+	+	+			unknown protein
21453		+	+	+	+	+	+			ATP-dependent RNA helicase DBP7. putative
21460										MMR; HR regulation
21461		+	+	+	+	+	+	1.270 down	2.003 down	ATP-dependent protease La. putative
21468		+	+	+	+	+	+			HFBs
21481			+							CinA Predicted nucleotide-utilizing enzyme related to molybdopterin-biosynthesis enzym
21498		+	+	+	+	+	+	2.221 up	3.163 up	unknown protein
21505		+	+	+	+	+	+	1.330 down	2.467 down	G-protein alpha subunit 3 GNA-3
21509		+	+	+	+		+			blue light inducible protein BLI-3
21534		+	+	+	+	+	+			di-trans.poly-cis-decaprenylcistransferase
21535		+	+	+	+	+	+			ATP synthase delta subunit
21542		+	+	+	+	+	+			histone acetyltransferase ESA1
21549		+	+	+	+	+	+			tRNA-splicing endonuclease subunit (tRNA-intron endonuclease).
21553		+	+	+	+	+	+			NADPH-adrenodoxin reductase. putative
21557	+	+	+	+	+	+	+			chromatin remodelling factors. contains a SWIRM domain and a DNA-binding Myb-domai
21564		+	+	+	+	+	+			unknown protein
21571		+	+	+	+	+	+	1.253 down	2.019 down	unknown protein
21576		+	+	+	+	+	+			GT α-1.2-mannosyltransferase
21588		+	+	+	+	+	+			endomembrane protein 70
21595								6.057 down	3.914 down	MFS permease
21606		+	+	+	+	+	+			NADH:ubiquinone oxidoreductase 20.1kD subunit. putative
21609		+	+	+	+	+	+			Ubiquinol cytochrome reductase

21618									thioredoxin-like protein
21633	+	+	+	+	+	+			unknown protein
21635									asparagine synthase-like protein
21641	+	+	+	+	+	+			Pep1/vps10 homolog; transmembrane sorting receptor for vacuolar hydrolases; cycles be
21646	+	+	+	+	+	+			Calpactin I heavy chain. calcium ion binding
21649	+	+	+	+	+	+			unknown protein
21653		+							DNA replication complex GINS protein PSF1. putative
21658	+	+	+	+	+	+			Unknown protein
21659	+	+	+	+	+	+			Dipeptidyl peptidase III. member of MEROPS peptidase family M49 of metallopeptidases;
21663	+	+	+	+	+	+			inosine-uridine preferring nucleoside hydrolase
21664	+	+	+	+	+	+			unknown protein
21668	+	+	+	+	+	+			unknown protein
21673	+	+	+	+	+	+			ATP synthase alpha chain. mitochondrial precursor
21677	+	+	+	+	+	+			unknown protein
21716	+	+	+	+	+	+			Acetate kinase
21725	+	+	+		+				GH20 exochitinase
21737	+	+	+	+	+	+			unknown protein
21742	+	+	+	+	+	+			Tubulin beta chain 1
21743	+	+	+	+	+	+			unknown protein
21745									unknown protein
21746									carboxymuconolactone decarboxylase. putative
21747									unknown protein
21749	+	+	+	+	+	+			mitogen-activated protein kinase MAF1 . putative
21752	+	+	+	+	+	+			unknown protein
21758	+	+	+	+	+	+	1.165 up	2.832 down	isocitrate lyase
21773	+	+	+	+	+	+			mitochondrial import inner membrane translocase subunit tim-14
21775	+	+	+	+	+	+			unknown protein
21782	+	+	+	+	+	+			GTP binding protein
21784	+	+	+	+	+	+			coiled-coil domain-containing protein 55. putative

21798	+	+	+	+	+	+			Unknown protein
21817	+	+	+	+	+	+	1.918 down	2.520 down	unknown protein
21824	+	+	+	+	+	+			MaoC-like peroxisomal dehydratase
21836	+	+	+	+	+	+	1.775 down	4.346 down	phosphoglucomutase/phosphomannomutase
21846	+	+	+	+	+	+			unknown protein
21873	+	+	+	+	+	+			dihydrounknown protein-acid dehydratase
21876									Zinc-binding oxidoreductase
21890	+	+	+	+	+	+			Ribosomal protein L32 based on homology to the corresponding protein in N. crassa.
21900	+	+	+	+	+	+			unknown protein
21907	+	+	+	+	+	+			unknown protein
21908	+	+	+	+	+	+			fatty acid elongase. 5 TM. GNS1/SUR4 membrane protein
21919	+	+	+	+	+	+			unknown protein
21924	+	+	+	+	+	+			unknown protein
21937	+	+	+	+	+	+			Heat shock protein DnaJ
21953	+	+	+	+	+	+			unknown protein
21957	+	+	+	+	+	+			pyruvate carboxylase (cytosolic)
21960	+	+	+				1.223 up	2.842 up	phospholipase C
21971	+	+	+	+	+	+			unknown protein
21972	+	+	+	+	+	+			small nuclear ribonucleoprotein
21982	+	+	+	+	+	+			Epl1/Sm1
21997									Zn2Cys6 transcriptional regulator
22000									unknown protein
22004		+	+						D-galacturonic acid reductase
22005	+	+	+	+	+	+			Dolichyl-P-mannose:protein O-mannosyl transferase
22009									unknown protein
22012	+	+	+	+	+	+			unknown protein
22013	+	+	+	+	+	+			Hsp70 chaperone (BiP). putative

22030	+	+	+	+	+	+	1.033 up	2.442 down	unknown protein
22035	+	+	+				1.702 down	2.376 down	unknown protein
22050	+	+	+	+	+	+			unknown protein
22064	+	+	+	+	+	+	1.726 down	3.163 down	DNAJ heat shock family protein
22072									GH63 processing α-glucosidase
22076	+	+	+	+	+	+	1.294 up	2.266 up	lysine decarboxylase
22093	+	+	+	+	+	+	2.004 down	6.633 down	Protein farnesyltransferase. alpha subunit
22104	+	+	+	+	+	+			MRP-type ABC transporter
22110									Flavin-containing monooxygenase
22115									poly polymerase (Poly[ADP-ribose] synthetase)
22117	+	+	+	+	+	+			RUM1 repressor of b mating type related genes. possibly involved in fruiting body formati
22119	+	+	+	+	+	+			ATP-dependent RNA helicase FAL1
22129							3.396 up	3.195 up	GH61 polysaccharide monooxygenase
22143	+	+	+	+	+	+			unknown protein
22148	+	+	+	+	+	+			serine palmitoyl CoA transferase subunit-like protein
22153	+	+	+	+	+	+			ran-interacting Mog1 protein
22154	+	+	+	+	+	+			AAA ATPase
22164	+	+	+	+	+	+			heat shock protein. Hsp40. DnaJ
22168							2.016 down	2.163 down	unknown protein
22181									unknown protein
22191	+	+	+	+	+	+			T-complex protein 1
22197		+							GH1 ß-glucosidase CEL1b
22201		+	+				1.223 down	2.717 down	unknown protein
22210									Zinc carboxypeptidase
22221	+	+	+	+	+	+			unknown protein

22245	+	+	+	+	+	+			aureobasidin resistance protein Aur1
22251	+	+	+	+	+	+	1.199 up	4.105 up	Mitochondrial carnitine-acylcarnitine carrier protein
22252	+	+	+	+	+	+			GH47 α-1.2-mannosidase
22257	+	+	+	+	+				unknown protein
22264	+	+	+	+	+	+			AATF-like transcription factor Bfr2
22271									Zinc-binding oxidoreductase
22277	+	+	+	+	+	+	2.830 up	2.159 up	sideroflexin-1
22283	+	+	+	+	+	+	1.179 down	2.130 down	unknown protein
22284									unknown protein
22287									Polyadenylation factor subunit 2. putative
22294	+	+	+	+	+	+			Nuclear transport factor 2
22300	+	+	+	+	+	+			vacuolar ATP synthase subunit d
22314	+	+	+	+	+	+			cell division control protein
22331									phospholipase D Active site motif protein / IQ calmodulin-binding motif protein
22332	+	+	+	+	+	+	1.838 up	2.292 up	small nuclear ribonucleoprotein E
22341	+	+	+	+	+	+			unknown protein
22351	+	+	+	+	+	+	2.142 up	4.051 up	legume-like lectin
22365	+	+	+	+	+	+			unknown protein
22375	+	+	+	+	+	+			unknown protein
22381	+	+	+	+	+	+			calcium/calmodulin-dependent protein kinase
22386	+	+	+	+	+	+	1.066 up	6.519 up	Epl1/Sm1
22388							1.051 up	2.048 down	unknown protein
22402	+	+							Peptidase M. neutral zinc metallopeptidases. zinc-binding site
22415	+	+	+	+	+	+			aldose-1-epimerase
22417	+	+	+	+	+	+			unknown protein
22426							1.434 down	2.484 down	unknown protein
22453									glutathione-S-transferase

22459							2.822 up	2.322 up	carboxypeptidase A
22464							1.475 down	7.818 down	Bifunctional P-450:NADPH-P450 reductase
22466	+	+	+	+	+	+			endomembrane protein 70
22472	+	+	+	+	+	+			U2 small nuclear ribonucleoprotein A. putative
22481									cytochrome b2. mitochondrial precursor
22484	+	+	+	+	+	+			glycolipid transfer protein
22489	+	+	+	+	+	+			electron transfer flavoprotein domain-containing protein
22492	+	+	+	+	+	+			COMPASS complex protein. putative
22496	+	+	+	+	+	+			unknown protein
22510	+	+	+		+				unknown protein
22512	+	+	+				3.243 up	7.442 up	short chain dehydrogenase/reductase
22515									nucleotide deaminase TAD3
22527	+	+	+	+	+	+			Dolichyl-P-mannose:protein O-mannosyl transferase
22528	+	+	+	+	+	+			Unknown protein with zinc finger domain.
22531	+	+	+	+	+	+			Membrane coat complex Retromer. subunit Vps29
22532	+	+	+	+	+	+	1.118 down	2.049 down	BZIP transcriptional regulator
22535	+	+	+						Zn2Cys6 transcriptional regulator
22538	+	+	+	+	+	+			Vacuolar protein sorting-associated protein Vps11
22559	+	+	+	+	+	+	1.385 down	2.173 down	unknown protein
22560									Nucleotide exchange factor Sil1
22563	+	+	+	+	+	+			unknown protein
22564	+	+	+	+	+	+			HFBs
22573		+	+						unknown protein
22589	+	+	+	+	+	+			unknown protein
22590									unknown protein
22598	+	+	+	+	+	+			pre-mRNA-processing factor 39
22608	+	+	+	+	+	+			porin. outer mitochondrial membrane protein

22611	+	+	+	+	+	+			IMP4. an SSU processome componentInvolvedIn pre 18s rRNA processing.
22618	+	+	+	+	+	+			unknown protein
22625	+	+	+	+	+	+	1.520 up	2.304 up	Molecular chaperone (DnaJ superfamily)
22632	+	+	+	+	+	+			aconitate hydratase
22633									S-(hydroxymethyl)glutathione dehydrogenase
22637	+	+	+						ERCC8 (CSA. CKN1) involved in transcription-coupled nucleotideExcision repair
22645									cell division cycle protein 23. putative
22647	+	+	+	+	+	+			unknown protein
22654							1.783 down	4.500 down	monodehydroascorbate reductase
22660	+	+	+	+	+	+			unknown protein
22661	+	+	+	+	+	+			unknown protein
22667	+	+	+				1.465 up	2.189 up	pre-rRNA-processing protein IPI1. putative
22678	+	+	+	+	+	+			2OG-Fe(II) oxygenase superfamily protein
22689	+	+	+	+	+	+			ribosome biogenesis protein Pescadillo. putative
22694	+	+	+	+	+	+			unknown protein
22705							1.175 up	2.255 down	urease
22707	+	+	+	+	+	+	1.183 up	2.228 up	unknown protein
22712	+	+	+	+	+	+			unknown protein
22713	+	+	+	+	+	+			U5 small nuclear ribonucleoprotein. Contains possible Interpro domain corresponding to e
22718	+	+	+				1.375 up	2.028 up	Glutaminylpeptide cyclotransferase
22731	+	+	+	+	+	+			autophagy protein Apg6
22737	+	+	+	+	+	+			unknown protein
22739	+	+	+	+	+	+			unknown protein
22741	+	+	+	+	+	+	1.330 down	2.539 down	unknown protein
22752	+	+	+	+	+	+			unknown protein
22755	+	+	+	+	+	+			Zn2Cys6 transcriptional regulator
22771		+							short chain dehydrogenase/reductase

22774	+	+	+	+	+	+			bHLH transcriptional regulator
22783	+	+	+	+	+	+			SNF2 family DNA-dependent ATPase
22785		+					1.166 down	3.150 down	C2H2 transcriptional regulator (amdA ?)
22798	+	+	+	+	+	+			Erg19p of Saccharomyces cerevisiae. a mevalonate pyrophosphate decarboxylase in theEr
22799	+	+	+	+	+	+			unknown protein
22804	+	+	+	+	+	+			glutaminyl-trna synthetase.
22816	+	+	+	+	+	+			NADH dehydrogenase iron-sulfur protein
22821	+	+	+	+	+	+			ribosomal protein L22/17
22826									PrsA Phosphoribosylpyrophosphate synthetase
22830	+	+					1.569 down	2.876 down	Glutathione S-transferase
22831									unknown protein
22839	+		+	+		+			translation initiation regulator Gnc20
22841	+	+	+	+	+	+	1.082 up	2.560 up	unknown protein
22845		+	+						ferric reductase
22863	+	+	+	+	+	+			unknown protein
22869	+	+	+	+	+	+			unknown protein
22874	+	+	+	+	+	+			septin-like protein
22875	+	+	+	+	+	+	1.047 down	3.346 down	unknown protein
22879	+	+	+	+	+	+			unknown protein
22881	+	+	+	+	+	+			cdc9p ligase
22885	+	+	+	+	+	+			ATP11 protein
22891	+	+	+	+	+	+			ubiquitin-conjugating enzyme
22910	+	+	+	+	+	+			GDP-forming succinate-CoA ligase. alpha subunit
22911	+	+	+	+	+	+			endonuclease/Exonuclease/phosphatase. putative
22912	+		+	+		+			MFS permease (glucose permease HXT1)
22914	+	+	+	+	+	+			GH72 β-1 3-glucanosyltransferase
22915									glucose oxidase

22925				+	+	+	+	+	+			Zn2Cys6 transcriptional regulator
22945				+	+	+	+	+	+			uracil phosphoribosyltransferase
22950				+	+	+	+	+	+			MYND domain protein (SamB)
22994				+	+	+	+	+	+			AAA-ATPase Cdc48
23001				+	+	+	+	+	+			Golgi complex component. cog1
23015				+	+	+	+	+	+			unknown protein
23028	+	+	+	+	+	+	+	+	+			Ca2+ permeable channel. related to N. crassa NCU02762.1
23034				+	+	+						unknown protein
23048				+	+	+	+	+	+			unknown protein
23050				+	+	+	+	+	+			unknown protein
23059				+	+	+	+	+	+	1.461 up	2.879 up	Ribosomal protein L24/L26
23062				+	+	+	+		+			unknown protein
23063				+	+	+	+	+	+			Vacuolar protein sorting-associated protein Vps24
23070				+	+	+	+	+	+			unknown protein
23083										2.470 up	3.030 up	Flavoprotein monooxygenase
23087												unknown protein
23090												short chain dehydrogenase/reductase
23111				+	+	+	+	+	+			unknown protein
23115				+	+	+	+	+	+	1.866 down	3.393 up	expansin
23118				+	+	+	+	+	+			unknown protein
23124				+	+	+	+	+	+	1.169 up	2.158 down	unknown protein
23146				+	+	+				1.007 up	2.499 up	unknown protein
23152										1.145 down	2.143 down	unknown LMBR1 domain protein
23154				+	+	+	+	+	+			transcriptional regulator. unknown
23171						+				3.634 up	9.894 up	NRPS
23173				+	+	+	+	+	+			unknown protein
23181				+	+	+	+	+	+			unknown protein

23184	+	+	+	+	+	+			Isocitrate/isopropylmalate dehydrogenase
23190	+	+	+	+	+	+			mRNA cleavage factor complex II protein Clp1. putative
23193	+	+	+	+	+	+	1.403 down	2.313 up	importin ß KapJ
23200	+	+	+	+	+	+			fructose-bisphosphate aldolase
23206	+	+	+	+	+	+			19S regulatory particle ATPase Rpt5
23208	+	+	+	+	+	+			eukaryotic ribosome biogenesis protein 1
23209									5'-nucleotidase
23221	+	+	+	+	+	+			cation pump. Calcium transport
23228									unknown protein Duf1479
23237	+	+	+	+	+	+			Methylthioadenosine phosphorylase MTAP
23238									unknown protein
23240									unknown protein
23263	+	+	+	+	+	+			ADP-ribosylation factor Arf6
23268									unknown protein
23271	+	+	+	+		+	1.398 up	2.713 up	ER-derived vesicles protein Erv41
23276	+	+	+	+	+	+			Integral ER membrane protein that regulates phospholipid metabolism
23287	+	+	+	+	+	+			unknown protein
23292									Zinc-binding oxidoreductase
23294	+	+	+	+	+	+			Zn2Cys6 transcriptional regulator
23295	+	+	+	+	+	+			calcium-binding mitochondrial carrier protein Aralar1
23297	+	+	+	+	+	+			unknown protein
23298	+	+	+	+	+	+			Chorismate_syntChorismate synthase
23316	+	+	+	+	+	+			Yip1 interacting protein Yop1
23323	+	+	+	+	+	+			unknown protein
23327	+	+	+	+	+	+			rRNA methyltransferase
23332									unknown protein
23338	+	+	+	+	+	+			unknown protein
23346									GH20 exochitinase
23347	+	+	+	+	+	+			unknown protein

23353									ferric reductase
23363	+	+	+	+	+	+			protein hob3 (involved in cell cycle)
23367	+	+							choline oxidase
23368	+	+	+						Flavin-containing monooxygenase
23382									aldehyde reductase AKR7
23408									unknown protein
23413									inositol monophosphatase family protein
23415	+	+	+	+	+	+			amino acid permease Dip5
23417	+	+	+	+	+	+			phosphotyrosine protein phosphatase
23420	+	+	+	+	+	+			prohibitin PHB1
23431	+	+	+	+	+	+			Cytochrome bd ubiquinol oxidase. 14 kDa subunit
23446	+	+	+	+	+	+			mitotic spindle checkpoint component mad2
23452	+	+	+	+	+	+			Post-transcriptional gene regulator. RNA-binding protein
23455		+							unknown protein
23458	+	+	+	+	+				glutathione S-transferase
23462							1.605 down	2.515 down	unknown protein
23475	+	+	+	+	+	+	1.594 down	2.142 down	Peptidase family M28 protein
23489	+	+	+	+	+	+			unknown protein
25040	+	+	+	+	+	+			Lrg1. expressed in sporulation
25159	+	+	+	+	+	+	1.924 down	2.060 down	PTPc. Protein tyrosine phosphatases (PTP)
25224									GH65 α.α-trehalase
25947	+	+	+	+	+	+			unknown protein
26019	+	+	+	+	+	+			unknown protein
26029	+	+	+	+	+	+			ATP-dependent RNA helicase MAK5. putative
26151	+	+	+	+	+	+			unknown protein of Lalv9 family
26160									unknown protein
26277	+	+	+	+	+	+			unknown protein

26462	+	+	+	+	+	+			cAMP-mediated signaling protein SOK1
26575									transcriptional regulator. unknown
26598	+	+	+	+	+	+			unknown protein
26642							2.673 down	3.309 down	MFS permease
26746	+	+	+	+	+	+			unknown protein
26787	+	+	+	+	+	+	1.036 up	2.073 down	ADP-ribosylation factor-binding protein Gga2
26871							1.361 down	2.021 down	Zn2Cys6 transcriptional regulator
27020	+	+	+	+	+	+			unknown protein
27025	+	+	+	+	+	+			Protein kinase
27042							1.852 down	2.192 down	unknown protein
27085	+	+	+	+	+	+			unknown protein
27132	+	+	+	+	+	+			unknown protein
27181							2.740 down	18.345 down	MFS permease
27219							1.966 up	2.694 up	GH27 α-galactosidase
27259									GH27 α-galactosidase
27357	+	+	+	+	+	+			Winged helix repressor DNA-binding
27384	+	+	+	+	+	+			SCF E3 ubiquitin ligase complex F-box protein grr1. putative
27395	+	+							GH76 α-1.6-mannanase
27398	+	+	+	+	+	+			origin recognition complex. subunit 2
27406	+	+	+	+	+	+			tyrosine-protein phosphatase CDC14
27422							2.149 up	2.109 up	RNA helicase DEAD/DEAH box
27492	+	+	+	+	+	+			tRNA pseudouridine synthase.
27554									GH61 polysaccharide monooxygenase
27599	+	+	+	+	+	+			cation efflux family protein
27600		+	+						Zn2Cys6 transcriptional regulator
27649	+	+	+	+	+	+			transcriptional regulator Rme1. repressor of the meiosis regulator protein IME1

27689									MFS permease
27697							1.122 up	3.020 down	Amidase
27706									Cytochrome P450 CYP2 subfamily
27719	+	+	+	+	+	+			Glucan synthesis regulatory protein
27722									Cytochrome P450 CYP2 subfamily
27770							1.439 up	6.637 down	MFS permease
27939							1.098 up	3.071 down	unknown protein
27948	+	+	+	+	+	+			GPCR. rhodopsin type
27983							1.272 down	3.331 up	PTH11 GPCR
27992							4.000 up	3.240 up	PTH11 GPCR
28036							4.652 down	3.610 down	MFS permease
28050									unknown protein
28159	+	+	+	+	+	+			Protoheme IX farnesyltransferase
28185									unknown protein. only in Chaetomium. A. oryzae. fumigatus and Streptomyces
28199	+	+	+	+	+	+			Mitochondrial import inner membrane translocase subunit tim-50
28353									unknown protein
28563		+							unknown protein
28655	+	+	+	+	+	+			unknown protein
28781									NDT80/PhoG like DNA-binding domain-containing protein
28787									unknown protein
28865	+	+	+	+		+			unknown protein
28928	+	+	+		+				PDI related protein Mpd1/PrpA
28975	+	+	+	+	+	+			Zn2Cys6 transcriptional regulator
29108		+	+						Peptidase D
29115	+	+	+	+	+	+			unknown protein
29142	+	+	+	+	+	+			unknown protein

29220	+	+	+	+	+	+			translation initiation factor eIF-2B delta subunit.
29275	+	+	+	+	+	+	1.297 up	3.219 up	unknown protein
29333	+	+	+	+	+	+			RNA binding protein RNP24
29346									phosphatidate-phosphatase
29439	+	+	+	+	+	+	1.808 down	2.325 down	unknown protein
29568	+	+	+	+	+	+			C-type cyclin
29619									Calpactin I heavy chain. calcium ion binding
29642							1.468 up	3.998 up	Phosphatidylserine decarboxylase
29709	+	+	+	+	+	+			stomatin-like protein
29710	+	+	+	+	+	+	1.362 down	3.454 down	unknown protein
29713									unknown protein
29716	+	+	+	+	+	+			thiamine-repressible mitochondrial transport protein THI74. putative
29756	+	+	+	+	+	+			UDP-N-acetylglucosamine transporter
29770	+	+	+	+					unknown protein
29932	+	+	+	+	+	+			unknown protein
29957	+	+	+	+	+	+			unknown protein
29993							1.516 down	3.465 down	unknown protein
30017									unknown protein
30018	+	+	+	+	+	+			unknown protein
30068									unknown protein
30075									unknown protein
30084							2.706 up	2.313 up	unknown protein
30144									unknown protein
30166							1.698 down	6.766 down	unknown protein
30214									GT glycosyltransferase. related to A. fumigatus capsule polysaccharide biosynthesis
30250		+	+						N-acetylglucosaminyltransferase
30274	+	+	+	+	+	+	1.148 up	3.126 up	unknown protein

30275	+	+	+	+	+	+			unknown protein
30330	+	+	+	+	+	+			unknown protein
30455	+	+	+	+	+	+			Translocation protein Sec62
30465							1.557 down	2.611 up	unknown protein. WD-repeats
30476	+	+	+	+	+	+			oxidation resistance protein 1. putative
30478	+	+	+	+	+	+			unknown protein
30578			+				1.547 down	4.472 down	HhH-GPD family base excision DNA repair protein
30635	+	+	+	+	+	+			unknown protein
30668									short chain dehydrogenase/reductase
30758									unknown protein
30759							1.892 down	2.654 down	zinc containing alcohol dehydrogenase superfamily
30776							1.036 up	2.281 up	alpha-ketoglutarate-dependent sulfonate diounknown proteingenase
30805	+	+	+	+	+	+			endonuclease III-like excision repair N-glycosylase involved in the repair of DNA base dam
30969									unknown protein
31041									unknown protein
31069									SSCP
31075	+	+	+	+	+	+			unknown protein
31118		+	+						unknown protein
31134	+	+			+		1.116 down	7.005 down	isoprenylcysteine carboxyl methyltransferase
31210							1.033 up	4.325 down	unknown protein
31227	+	+	+	+	+	+			unknown protein
31248		+					1.341 down	3.187 down	Ribonuclease. T2 family
31415		+							unknown protein
31447							1.867 up	2.863 up	unknown protein
31481	+	+	+	+	+	+	2.395 up	2.074 up	chaperone protein dnaJ 6

31521	+	+	+	+	+	+			unknown protein
31551	+	+	+	+	+	+			Cytidylyltransferase
31559	+	+	+	+	+	+			unknown protein
31611	+	+	+	+	+	+			SSCRP
31733	+	+	+	+	+	+			ubiquitin-conjugating enzyme. putative
31798	+	+	+	+	+	+			transfer of mannosylphosphate
31812									unknown protein
31869	+	+	+	+	+	+			unknown protein
31976	+	+	+	+	+	+			ribosomal protein L6
32002	+	+	+	+	+	+			unknown protein
32027									unknown protein
32069	+	+	+	+	+	+			unknown protein
32087							1.346 up	4.040 up	short chain dehydrogenase/reductase
32149		+	+			+			unknown protein
32203	+	+	+	+	+	+			unknown protein
32204									unknown protein
32212							1.166 down	2.618 up	SSCRP
32243	+		+				1.428 down	3.056 down	Alpha/beta hydrolase
32261	+	+	+	+	+	+	1.832 down	2.678 down	unknown protein
32263	+	+	+						unknown protein
32293									SAM dependent methyltransferase-like
32364							1.586 down	6.507 down	GDSL lipase
32402							1.009 down	3.214 up	glutathione S-transferase GliG. putative
32473	+	+	+	+	+	+			mitochondrial ribosomal protein RSM25.
32478							1.093 up	3.281 up	unknown protein
32543	+	+	+	+	+	+			unknown protein

32639							1.471 up	4.886 up	unknown protein
32712									unknown protein. NACHT domain/ankyrin repeats
32714	+	+	+			+			unknown protein
32716									unknown protein
32747							1.420 up	2.856 up	unknown protein
32755									Histone tail methylase containing SET domain
32798	+	+	+	+	+	+	1.207 up	5.624 up	unknown protein
32849	+	+	+	+	+	+	1.465 down	2.581 down	unknown protein
33029							1.001 up	4.594 up	unknown protein
33058							2.148 up	2.491 up	unknown protein
33067	+	+	+	+	+	+			unknown protein
33085	+	+	+	+	+	+			TFIID and SAGA complexe subunit
33090	+	+	+	+	+	+			magnesium-dependent phosphatase. putative
33169									dienelactone hydrolase
33197	+	+	+	+	+	+			mitochondrial chaperone Frataxin. putative
33207		+					1.147 up	2.231 down	cation transport protein ChaC. putative
33263			+						unknown protein
33312	+	+	+	+	+	+			unknown protein
33342	+	+	+	+	+	+	1.461 up	7.618 up	Oligosaccharyltransferase. epsilon subunit
33359	+	+	+	+	+	+	1.743 up	4.041 up	translation initiation factor SUI1 and density-regulated protein.
33371							1.078 up	2.569 up	phosphoglycerate mutase family protein
33387	+	+	+	+	+	+	1.411 down	3.518 down	GCN5-related N-acetyltransferase. putative
33475	+	+	+	+	+	+			unknown protein
33482	+	+	+	+	+	+			unknown protein
33502									unknown protein
33586	+	+	+	+	+	+			ATP syntase delta chain
33632		+	+						unknown protein

33637		+	+			+			TRAPP complex component Trs23
33650	+	+	+						unknown protein
33660	+	+	+	+	+	+			unknown protein
33692	+	+	+	+	+	+			unknown protein
33704	+	+	+	+	+	+			transcriptional regulator. unknown
33723							1.977 up	2.839 up	ankyrin repeat-containing protein. putative
33728	+	+	+	+	+	+			unknown protein
33763									unknown protein
33811									unknown protein
33815	+	+	+	+	+	+			U2 snRNP component IST3
33827									unknown protein
33840	+	+	+	+	+	+			unknown protein
33894	+	+	+	+		+			unknown protein
33895	+	+	+	+	+	+			Peptidylprolyl isomerase. FKBP-type
33949							1.178 up	2.535 up	NADH:cytochrome b5 reductase (CBR). putative
33960		+					1.529 up	2.528 up	prefoldin subunit 6. putative
34079	+	+	+	+	+	+			small nuclear ribonucleoprotein Sm D3
34112	+	+	+	+	+	+			ribosomal protein L22.
34149	+	+	+	+	+	+			U6 snRNA-associated Sm-like protein LSm7. putative
34183							1.467 up	2.019 up	unknown protein
34197	+	+	+				1.125 up	2.879 down	BolA-like protein
34221	+	+							myb transcriptional regulator
34248									transcriptional regulator HMG type
34252							1.742 down	5.722 down	unknown protein
34272									unknown protein. only present in Neurospora. Magnaporthe. Chaetomium
34274	+	+	+	+	+	+			unknown protein
34277		+							unknown protein
34280	+	+					2.015 up	2.031 up	unknown protein

34297									SSCRP
34306	+	+	+	+	+	+			mitochondrial cytochrome c oxidase assembly factor. putative
34312							1.230 up	6.305 up	conidiation protein 6
34327	+	+	+	+	+	+	1.702 up	2.138 up	NADH-ubiquinone oxidoreductase B12 subunit. putative
34353									unknown protein
34378	+	+	+	+	+	+			Mitochondrial cytochrome c oxidase subunit VIIc
34402	+	+	+	+		+			Histone H1/H5
34611	+	+	+	+	+	+			unknown protein
34726	+	+	+	+	+	+	1.973 up	2.671 up	Guanine nucleotide exchange factor for Ras-likeGTPases (RasGEF)
34985			+						C-5 cytosine-specific DNA methylase
35109							1.535 down	17.094 up	unknown protein. contains WD repeats
35137							1.427 down	2.234 up	unknown protein
35183	+	+	+	+	+	+			unknown protein
35186									unknown protein
35202									unknown protein
35240	+	+	+	+	+	+			unknown protein
35316	+	+	+	+	+	+			SIT4 phosphatase associated protein
35317	+	+	+	+	+	+			unknown protein
35375	+	+	+	+	+	+			TBC domain protein (GAP)
35386	+	+	+	+	+	+			actin-interacting protein AIP3
35454	+	+	+	+	+	+			Zn2Cys6 transcriptional regulator
35465	+	+	+	+	+	+			Molecular chaperone Hsp70 family Lhs1
35522	+								AMPD. AMP deaminase
35534							1.338 up	2.277 up	unknown protein
35552									C2H2 transcription factor
35556	+	+	+	+	+	+			AAA ATPase
35726							1.121 up	2.775 up	Subtilisin like protease
35768									unknown protein

35777	+						1.359 down	2.383 down	K(+)/H(+) antiporter 1
35867	+	+	+	+	+	+			Protein interacting with poly(A)-binding protein
36006	+	+	+	+	+	+	1.591 down	2.621 down	Serine/Threonine protein kinase
36134	+	+	+	+	+	+			protein kinase family protein
36159	+	+	+	+	+	+	1.202 down	2.237 down	unknown protein
36267	+	+	+	+		+			unknown protein. 3TM
36335							1.362 up	7.762 down	Zn2Cys6 transcriptional regulator
36373									Vacuolar assembly/sorting protein VPS9
36391	+	+	+	+	+	+			C2H2 transcriptional regulator
36468							1.872 down	3.992 down	Zn2Cys6 transcriptional regulator
36530		+					1.084 down	2.227 down	unknown protein
36543	+	+	+	+	+	+			transcriptional regulator STE12
36576	+	+	+	+	+	+			unknown protein
36608							1.657 up	2.149 down	unknown protein
36639	+	+	+	+	+	+			unknown protein
36703							1.326 down	2.569 down	Zn2Cys6 transcriptional regulator
36727	+	+	+	+	+	+			unknown protein
36822									unknown protein
36855									unknown protein
36941	+	+	+	+	+	+			DEAD/DEAH box helicase
37060	+	+	+	+	+	+			Ca-permeable channel. related to A. nidulans Mid2
37067	+	+	+	+	+	+			unknown protein
37214	+	+	+	+	+	+			unknown protein
37262									unknown protein

37271	+	+	+	+	+	+			unknown protein with SH3 domain
37316							1.014 down	3.350 up	unknown protein
37366	+	+	+	+	+	+			MFS permease
37368	+	+	+	+	+	+			autophagy related lipase Atg15. putative
37525							5.125 up	3.989 up	GPCR . contains RGS domain
37593	+	+	+	+	+	+			GTP binding protein
37665									unknown protein
37761	+	+	+	+	+	+			splicing factor U2AF 65 kDa subunit
37783							3.819 down	7.917 down	unknown protein
37827									Cytochrome P450
37833	+	+	+	+	+	+			UBA/THIF-type NAD/FAD binding fold
37921									Cytochrome P450 CYP2 subfamily
37933		+							myb transcriptional regulator
37950							1.199 up	4.659 up	unknown protein
38048							1.436 up	2.503 down	unknown protein
38080	+	+	+	+	+	+	1.013 up	2.511 down	C2H2 transcriptional regulator
38227									unknown protein
38274									HET protein
38341									MFS permease
38372	+	+	+	+	+	+			RluA family pseudouridine synthase
38441			+						unknown protein
38527									unknown protein. only present in Aspergilli and Gibberella
38536									GH16 glucan endo-1.3(4)-β-D-glucosidase
38573	+	+	+			+			unknown protein
38603							1.162 up	2.404 down	MFS permease

38640	+	+	+	+	+	+	1.062 up	2.448 down	unknown protein
38749									Cytochrome P450 CYP2 subfamily
38767	+	+	+	+	+	+			mRNA capping enzyme
38781									unknown protein
38812									iron transporter
38833							1.641 down	3.197 down	unknown protein
38863	+	+	+	+	+	+	1.257 down	2.096 down	unknown protein
38876									unknown protein
39156									SSCRP
39221		+					1.032 up	2.130 down	Zn2Cys6 transcriptional regulator
39285									unknown protein
39351									unknown protein
39387	+	+	+	+	+	+			Pex2/Pex12
39426									unknown protein
39535									mannitol-1-phosphate dehydrogenase
39578									unknown protein
39587									unknown protein
39588									Pyridine nucleotide-disulphide oxidoreductase.
39590	+	+	+	+	+	+	1.352 up	3.305 up	Membrane protein Erj5 with DnaJ domain
39606							1.531 down	2.360 down	unknown protein
39637									C4-dicarboxylate transporter/malic acid transport protein
39753									unknown protein
39755	+	+	+				1.818 down	2.781 down	GH16 glucan endo-1.3(4)-β-D-glucosidase
39827	+	+	+	+	+	+			unknown protein
39883	+	+	+						unknown protein

39911									SAM-dependent methyltransferases
39942	+	+	+	+	+	+			GH17 glucan endo-1.3-β-glucosidase
39996									6-phosphogluconolactonase. putative (check!)
40053	+	+	+	+	+	+			unknown protein
40074									sedoheptulose-1.7-bisphosphatase
40114	+	+	+	+	+	+			unknown protein
40156	+	+	+						unknown protein
40199									unknown protein
40216	+	+	+	+	+	+			unknown protein
40250	+	+	+	+	+	+			unknown protein
40290	+	+	+	+	+	+	1.281 up	2.479 up	unknown protein
40298	+		+						unknown protein
40338	+	+	+	+	+	+			unknown protein
40343									unknown protein
40346		+					1.959 up	2.292 up	formamidopyrimidine-DNA glycosylase
40374									unknown protein
40519	+	+	+	+	+	+			unknown protein
40534	+	+	+	+	+	+			unknown protein
40538	+	+	+	+	+	+			unknown protein
40551	+	+	+	+	+	+			velB
40618									unknown protein. secreted. only in ascomycota
40758	+	+	+	+	+	+			Homocysteine/selenocysteine methylase (S-methylmethionine-dependent). COG2040. MI
40775							2.429 down	2.752 down	RTA1 protein. 7 TNM. responds to xenobiotic stimulus
40808							10.571 up	2.515 down	short-chain dehydrogenase/reductase
40814	+	+	+	+	+	+			unknown protein
40830									unknown protein
40914									unknown protein
40918	+	+	+	+	+	+			unknown protein

40943										1.207 down	3.540 down	unknown protein
40945					+							N-glycosylase/DNA lyase
40996				+	+	+	+	+	+			unknown protein
41001				+	+	+	+	+	+	1.137 up	2.303 up	unknown protein with Nif domain
41009												Ras-like GTPase. Rho type
41035				+	+	+						G-protein coupled receptor protein. contains Molluscan rhodopsine C-terminal tail. possib
41111				+	+	+	+	+	+			RNA polymerase II transcription mediator complex subunit (Med6). putative
41149												unknown protein. 2 TM. only present in fungi. distantly related to acetlytransferase from
41152												unknown protein
41171										1.220 down	4.325 down	unknown protein
41178					+							aquaporin-2 (major intrinsic protein)
41208												metallophosphoesterase domain-containing protein. putative
41248												CE3 acetyl xylan esterase
41260	+	+	+							1.057 up	2.770 up	PTH11 GPCR
41312				+	+	+						short chain dehydrognease/reductase
41325										1.625 up	2.104 down	RTA1 like protein; 7 TM
41362												unknown protein
41425										1.086 down	3.458 down	unknown protein
41428				+	+	+	+	+	+	1.027 down	2.445 down	Tyrosine specific protein phosphatase and dual specificity protein phosphatase
41501						+						unknown protein
41504										1.054 up	2.124 up	phosphopantetheinyl transferase. putative
41518				+	+	+	+	+	+			unknown protein
41546				+	+	+	+	+	+			unknown protein
41573				+	+	+	+	+	+	1.293 down	2.085 down	BZIP transcriptional regulator

41590								2.028 down	3.551 down	iron transporter
41604										unknown protein. 4 TM. unknown in yeast
41607										unknown protein
41617				+			+	372.860 down	5.394 up	lae1
41660										HET domain protein
41663			+			+				unknown protein
41664										HET domain-containing protein
41690										unknown protein
41699				+				2.380 up	3.449 up	HET protein
41712										ubiquitin domain
41719		+	+	+						CutC family protein
41761		+	+					1.077 down	2.205 down	iron transporter
41768								1.172 up	3.543 up	GH16 cell wall glucanosyltransferase
41794										unknown protein
41800		+	+	+	+	+	+			unknown protein
41895										glutathione-S-transferase
41942	+							1.770 up	2.165 down	unknown protein
41987		+	+	+	+	+	+			unknown protein
42027		+	+	+	+	+	+			unknown protein
42043								1.059 up	3.502 up	unknown protein
42053			+							unknown protein
42063										unknown protein
42068		+	+	+	+	+	+			unknown protein
42152								1.491 down	5.173 down	GH75 chitosanase
42181		+	+	+	+	+	+	1.175 up	2.499 up	unknown protein
42193		+	+	+	+	+	+			unknown protein

42249	+	+	+	+	+	+			transcriptional regulator UMC1
42264							1.681 up	8.807 down	3'-5' exonuclease
42267									ankyrin repeat protein
42301	+	+	+	+	+	+			unknown protein
42326							1.404 up	2.046 down	unknown protein
42329	+	+	+	+	+	+			unknown protein
42346	+	+							unknown protein
42355							1.434 up	2.154 down	unknown protein
42377		+							unknown protein
42418	+	+	+	+	+	+			unknown protein
42449							1.313 up	2.689 up	N-acetyltransferase activity
42455	+	+	+	+	+	+			unknown protein
42462	+	+	+	+	+	+			unknown protein
42513									unknown protein
42571							1.196 down	2.069 up	unknown protein
42752			+						unique protein
42766									integral membrane protein
42792									unknown protein
42825			+						Cytidine/deoxycytidylate deaminase
42848	+	+	+	+	+	+	2.421 down	2.534 down	unknown protein
42858	+	+							unknown protein
42866	+	+	+				2.932 down	3.059 down	thioesterase family protein
42915	+	+	+	+	+	+			unknown protein
42919	+	+	+	+	+	+	1.237 up	2.199 up	ubiquitin fusion protein

42942									1.087 down	2.798 up	unknown protein
42953			+	+	+	+	+	+			unknown protein
42972			+	+	+	+	+	+			unknown protein. only present in Gibberella and Magnaporthe
43071			+	+	+	+	+	+			unknown protein
43076				+					1.070 up	2.626 down	unknown protein
43083			+	+	+	+			1.236 down	3.216 up	unknown protein
43101											unknown protein
43115					+				2.885 up	2.190 up	SSCRP
43129			+	+	+	+	+	+			unknown protein
43161									1.364 up	3.214 up	Carboxylesterase and related proteins
43176			+	+	+	+	+	+			unknown protein
43191			+	+	+	+	+	+			unknown protein
43195											unknown protein
43199	+	+	+		+				1.442 up	3.792 up	unknown protein
43225			+	+	+	+	+	+	2.007 up	3.317 up	U6 small nuclear ribonucleoprotein (Lsm3). putative
43236			+	+	+	+	+	+	1.449 up	2.153 up	unknown protein
43242			+	+	+			+			unknown protein
43268			+	+	+	+	+	+			unknown protein
43269			+	+	+	+	+	+	3.176 up	2.723 up	bHLH transcriptional regulator
43286			+	+	+						unknown protein
43302	+	+	+		+				1.007 down	5.446 up	unknown protein
43312			+	+	+						unknown protein
43347				+							DASH complex subunit Dad3. putative
43371											unknown protein
43392	+	+	+		+			+	1.651 up	5.859 up	unique protein
43397											unknown protein. secreted. only in hypocreaceae. HTG
43398				+							

43401								1.387 up	19.063 up	unknown protein
43418	+	+	+	+	+	+	+			unknown protein
43430										unknown secreted protein
43472										unknown protein
43599		+	+	+	+	+	+			Dynamin-like GTPase Vps1
43615		+	+	+	+	+	+			eukaryotic translation initiation factor 2. subunit 1 alpha. 35kDa.
43618		+	+	+	+	+	+			RNA polymerase II-associated protein
43649		+	+	+	+	+	+			T-complex protein 1
43662										SAICAR synthase
43664		+	+	+	+	+	+			UTP-glucose-1-phosphate uridylyltransferase
43671			+							ammonium permease MEA1
43680		+	+	+	+	+	+			pre-mRNA branch site protein p14
43701		+	+	+	+	+	+	1.993 up	4.588 up	MFS multidrug transporter
43770		+	+	+	+	+	+			unknown protein
43802		+	+	+	+	+	+			Protein invovled in ER retention Rer1
43814		+	+	+	+	+	+			Ribosomal protein L43A (L37 family) by homology to the corresponding protein of Candid
43817		+	+	+	+	+	+			unknown protein
43831		+	+	+	+	+	+			phospholipid-translocating P-type ATPase
43873										GH18 chitinase CHI18-12
43877		+	+	+	+	+	+			Arfs GEF Sec7
43884		+	+	+	+	+	+			unknown protein
43893								1.466 down	2.366 down	unknown protein
43906		+	+	+	+	+	+			60s ribosomal protein L1.
43919		+	+	+	+	+	+			unknown protein
43961										RNA polymerase III transcription initiation factor complex (TFIIIC) subunit part of the Tau
43974		+	+	+	+	+	+	1.608 up	2.180 up	UDP-galactose transporter
44035		+	+	+	+	+	+			unknown protein
44038		+	+	+	+	+	+			dihydrolipoyllysine-residue acetyltransferase-like protein
44041		+	+	+	+	+	+			NADH dehydrogenase (ubiquinone) Fe-S protein 1

44088	+	+	+	+	+	+			unknown protein
44097	+	+	+	+	+	+			AAA family ATPase Pontin. putative
44117							1.424 down	2.529 down	Heterokaryon incompatibility protein HEC-C
44175	+	+	+				5.701 down	10.981 down	MFS H+ sugar transporter
44178	+	+	+	+		+			unknown protein
44214	+	+	+	+	+	+			CE5 acetyl xylan esterase AXE2
44230	+	+	+	+	+	+			GTP-binding protein EsdC
44251	+	+	+	+	+	+	1.078 down	2.544 up	GTPase-activating protein Msb3 (Sec4/Rabs)
44278	+	+	+	+		+	2.647 down	4.813 down	Rab geranyl transferase escort protein
44290	+	+	+	+	+	+			C2H2 transcriptional regulator
44306									unknown protein
44330	+	+	+	+	+	+			serine/threonine protein kinase YAK1
44362							7.751 up	8.294 up	unknown protein
44366							2.297 down	2.179 down	Glycosylphosphatidylinositol-specific phospholipase C
44386	+	+	+	+	+	+			ribosomal protein MRPL40.
44389									Urease accessory protein UreG
44399	+	+	+	+	+	+			unknown protein
44419	+	+	+	+					ARG5.6
44434	+	+	+	+	+	+			unknown protein
44449	+	+	+	+	+	+			centromere/microtubule binding protein CBF5
44459	+	+	+	+	+	+	2.076 up	3.266 up	Rfc4p (replication factor C) subunit 4
44476	+		+				4.980 down	2.986 down	MRP-type ABC transporter
44488	+	+	+	+	+	+			unknown protein
44492	+	+	+	+	+	+			mitochondrial import receptor subunit TOM40
44504	+	+	+	+	+	+			actin

44529	+	+	+	+	+	+			glycogen synthase involved in carbohydrate transport and metabolism
44546	+	+	+	+	+	+			unknown protein
44624	+	+	+	+	+	+			unknown protein
44628									Kynureninase
44640							2.128 up	2.630 up	unknown protein
44684	+	+	+	+	+	+			unknown protein
44700	+	+	+						unknown protein. only present in ascomycota and Bacteriophage phBC6A51
44704	+	+	+	+	+	+			Rab family GTPase activating protein
44705	+	+	+	+	+	+			cell lysis protein-like protein
44725	+	+	+	+	+	+			PDI related protein Eps1
44747									SNF2 family helicase
44764									Unknown protein with GRF zinc finger domain.
44781									Transcription initiation factor IID. 18 kDa subunit
44814	+	+	+	+	+	+			COPI-coated vesicle protein. putative
44849	+	+	+	+	+	+			ADP-ribosylation factor GTPase activating protein
44868	+	+	+	+	+	+			unknown protein
44878	+	+	+	+	+	+	1.298 down	2.413 down	unknown protein
44928									5-formyltetrahydrofolate cyclo-ligase
44943	+	+	+	+	+	+			unknown protein
44956	+	+	+	+	+	+			MFS permease
44960	+	+	+	+	+	+			pre-mRNA splicing factor CEF1. putative
44965	+								zinc binding oxidoreductase
44967	+	+	+	+		+			unknown protein.
44987	+	+	+	+	+	+			ERG25 methylsterol desaturase. SUR2-type hydroxylase/desaturase. catalytic region
45018	+	+	+	+	+	+			MAPK3. probably involved in stress response and osmosensing. Hog1p
45050	+	+	+	+	+	+			vacuolar sorting protein. vps17
45085	+	+	+	+	+	+			unknown protein
45112	+	+	+						serine/threonine protein kinase
45138	+	+	+	+	+	+			sulfite reductase. ß-subunit

45153		+	+	+	+	+	+			mitochondrial F1F0 ATP synthase subunit F Atp17
45191		+	+	+	+	+	+			unknown protein
45242		+	+	+	+	+	+			Transmembran serin/threoninkinase Ire1
45250		+	+	+	+	+	+	1.483 up	4.138 up	oleate-delta12-desaturase
45252		+	+	+	+	+	+			unknown protein
45317		+	+	+	+	+				unknown protein
45343										Sodium Bile acid symporter family protein
45369										unknown protein
45445		+	+	+	+	+	+			tyrosinase
45456		+	+	+	+	+	+			Protein kinase
45459		+	+	+						BCAT_beta_family
45476		+	+	+	+	+	+			v-Snare Sft1; intra-Golgi
45503										ankyrin containing protein
45512								1.314 up	2.214 up	unknown protein
45523										MFS permease
45571										short chain dehydrogenase/reductase
45573		+	+	+				1.329 up	2.118 up	PTH11 GPCR
45595		+	+	+	+	+	+			unknown protein
45598		+	+	+	+	+	+	2.064 down	2.003 down	unknown protein
45604		+	+	+	+	+	+			ADP-ribosylation factor Arf1
45624		+	+	+	+	+	+			unknown protein
45652										Protein kinase
45675								1.482 down	3.326 down	unknown protein
45688	+									unknown protein
45689		+	+	+	+	+	+			unknown protein
45717				+				4.496 down	4.485 down	GH47 α-1.2-mannosidase
45727		+	+	+	+	+	+			Metallophosphoesterase

45732	+	+	+	+	+	+			Oligosaccharyltransferase. gamma subunit
45736	+	+	+	+	+	+			CPC2
45748	+	+	+	+	+	+			unknown protein
45775	+	+	+	+	+	+			Cytoskeleton assemblyControl protein. sla1. interacts with proteins regulating actin dynar
45852									unknown protein
45866	+	+	+	+					C2H2 transcriptional regulator
45868	+	+	+	+		+			phosphate transporter
45912	+	+	+	+	+	+			60s ribosomal protein L36 based on homology to the corresponding protein in other fungi
45971	+	+							mismatched base pair and cruciform DNA recognition protein
45980	+	+	+	+	+	+			unknown protein
45998	+	+	+	+	+	+			serine threonine protein kinase SNF1
46002	+	+	+	+	+	+			unknown protein. only in fungi
46035									unknown protein
46057	+	+	+	+	+	+	1.111 up	2.011 up	unknown protein
46127	+		+						unknown protein
46128							2.682 down	2.114 down	AAA ATPase
46203	+	+	+	+	+	+			unknown protein
46209									SAM-methyltransferase. only Hypocreaceae
46238	+	+	+	+	+	+			S-adenosylmethionine synthetase
46240	+	+	+	+	+	+			ATP-binding protein
46242	+	+	+	+	+	+			unknown protein
46244	+	+	+	+	+	+			tetrahydrofolate dehydrogenase/cyclohydrolase
46247	+	+	+	+	+	+			unknown protein
46266	+	+	+	+	+	+			unknown protein
46285									Hsp26/Hsp42
46301	+	+	+	+	+	+			ubiquinone biosynthesis monooxygenase COQ6
46320							1.441 up	2.162 up	small nuclear ribonucleoprotein LSM2
46382	+	+	+	+	+	+			elongation factor Tu (GTP binding).
46421	+	+	+	+	+	+			GT α-1.2-mannosyltransferase

46443		+	+	+				1.623 up	2.437 down	GT α-1.6-mannosyltransferase
46446		+	+	+	+	+	+	1.162 down	2.194 down	Thioredoxin binding protein TBP-2
46463		+	+	+	+	+	+			serine palmitoyl transferase. subunit LCBB
46469		+	+	+	+	+	+			G-protein beta subunit
46490		+	+	+	+	+	+			Rps16 gene encoding protein component of the small (40S)Ribosomal subunit (S9 family).
46493										unknown protein
46545		+	+	+	+	+	+	2.305 up	18.397 up	Prephenate dehydrogenase
46582		+	+	+	+	+	+			unknown protein
46597		+	+	+	+	+	+			unknown protein
46607		+	+	+	+	+	+			Nop14p. maturaltion of 18rRNA
46613		+	+	+	+	+	+			unknown protein
46619		+	+	+	+	+	+			ATP-dependent RNA helicase eIF4A
46639		+	+	+	+	+	+			Prenylated Rab acceptor 1 Yip3
46687		+	+	+	+	+	+			unknown protein with PX domain
46702		+	+	+	+	+	+			eukaryotic translation initiation factor 3 subunit M
46763		+	+	+	+	+	+	1.281 up	3.639 down	endonuclease/exonuclease/phosphatase family protein
46764										unknown protein
46794		+	+	+	+	+	+			oligopeptide transporter
46816								2.115 down	3.253 up	GH3 β-glucosidase CEL3d
46819	+									MFS hexose transporter
46862		+	+	+	+	+	+			transcription factor IWS1
46882		+	+	+	+	+	+			unknown protein
46902		+	+	+	+	+	+			Transcriptional activator HAC1. UPR regulator
46936		+	+	+	+	+	+			2-deoxy-D-gluconate 3-dehydrogenase
46958		+	+	+	+	+	+			Translation elongation factor 1a
47020				+						Phosphatidylinositol transfer protein
47049		+	+	+	+	+	+			unknown protein

47055	+	+	+	+	+	+			RAS small GTPase. Rac subfamily. related to Colletotrichum trifolii RAC1
47066	+	+	+	+	+	+			Sulfate adenylyltransferase
47069	+	+	+	+	+	+			unknown protein
47077							1.986 down	2.020 down	diounknown proteingenase
47127	+	+	+	+	+	+			peptidase M18
47134									unknown protein
47136	+	+	+	+	+	+	1.064 up	2.384 up	Glutathione peroxidase
47153	+	+	+	+	+	+			D-galacturonic acid reductase
47167	+	+	+	+	+	+			unknown protein
47175	+	+	+	+	+	+			Aa_transTransmembrane amino acid transporter protein
47221	+	+	+	+	+	+			Nucleoside diphosphate kinase
47268	+	+	+		+	+	2.346 up	2.922 up	GH3 β-glucosidase BGL3i
47286	+	+	+				1.704 down	2.369 down	unknown protein
47290	+	+	+	+	+	+			protein palmitoyl transferase PFA3
47315							2.264 down	2.449 down	P-type ATPase
47317	+	+	+	+	+				Zn2Cys6 transcriptional regulator
47330	+	+	+	+	+	+	1.578 up	4.142 up	SSCRP
47420	+								FAD binding domain-containing protein
47424									unknown protein
47432	+	+	+	+	+	+			NAD(P) transhydrogenase beta subunit
47479							1.229 up	3.275 down	Zn2Cys6 transcriptional regulator
47510	+	+	+	+	+	+	1.451 up	2.230 up	Complex 1 LYR protein
47600	+	+	+	+	+	+			unknown protein
47603	+	+	+	+	+	+	1.351 down	2.082 up	succinyl-CoA:3-ketoacid-coenzyme A transferase subunit A
47611		+							unknown protein
47621	+	+	+	+	+	+			unknown protein

47635				+	+	+	+	+	+			stress response regulator SrrA
47670				+	+	+	+	+	+			unknown protein
47710				+	+	+	+	+	+			MFS permease
47733					+	+				3.912 up	5.034 up	SSCRP
47752				+	+	+	+	+	+			unknown protein
47766				+	+	+	+	+	+			unknown protein
47795				+	+	+	+	+	+	1.410 up	2.027 up	ribosomal protein S10.
47806				+	+	+	+	+	+			unknown protein
47814					+	+				1.035 down	2.472 down	prenylcysteine lyase. putative
47829				+	+	+	+	+	+			unknown protein
47838				+	+	+	+	+	+			histone chaperone ASF1
47897				+	+	+	+	+	+			PDR-type ABC transporters
47926				+	+	+	+			1.723 up	4.383 up	ABC1 family protein
47930				+	+	+	+	+	+			Mitochondrial oxoglutarate/malate carrier proteins
47971				+	+	+	+	+	+			unknown protein. WD domains
47987				+	+	+	+	+	+			ZIP Zinc transporter
48001				+	+	+	+	+	+			unknown protein with DnaJ domain
48055				+	+	+	+	+	+			unknown protein
48058				+	+	+	+	+	+			unknown protein
48080										1.148 up	12.864 down	unknown protein
48146				+	+	+	+	+	+			leucyl-tRNA synthetase. class Ia
48170				+	+	+	+	+	+			ribosomal protein L27e. Homologue of yeast RPL27a/b.
48178				+	+	+	+	+	+			UDP-galactopyranose mutase
48211				+	+	+	+	+	+	1.886 down	4.585 down	unknown protein. intracellular
48225				+	+	+	+	+	+			unknown protein
48266	+	+	+	+	+	+	+	+	+			unique protein
48280					+					1.455 up	16.937 up	unknown protein

48281	+	+	+	+	+	+			Zn2Cys6 transcriptional regulator
48295		+					1.862 up	2.929 up	unknown protein
48301	+	+	+	+	+	+			unknown protein
48351	+	+	+	+	+	+			protease required for autophagy
48366	+	+	+	+	+	+	1.269 up	2.111 up	26S proteasome regulatory complex subunit Rpn12
48386	+	+	+	+	+	+			Histone deacetylase; regulates transcription and silencing
48398	+	+	+	+	+	+			unknown protein
48438			+				1.270 down	3.364 down	Zn2Cys6 transcriptional regulator
48444	+		+						MFS maltose permease
48478	+	+	+		+				t-SNARE. SSO2
48482									unknown protein
48486	+	+	+	+	+	+			unknown protein
48516	+	+	+	+	+	+			half-sized ABC transporter
48541	+	+	+	+	+	+			unknown protein
48599									unknown protein
48603									glycerophosphoryl diester phosphodiesterase family protein
48653	+	+	+	+	+	+			F-box domain-containing protein
48686	+	+	+	+	+	+			unknown protein
48707	+	+	+	+	+	+			Bifunctional trehalose-6-phosphate synthase/trehalose-6-phosphate phosphatase
48747	+	+	+						unknown protein
48773									Zn2Cys6 transcriptional regulator
48788	+	+	+	+	+	+			fatty acid synthase - candidate FAS2
48792	+	+					2.193 up	3.449 up	amidase
48819	+	+	+	+	+	+			aspartyl-tRNA synthetase. class IIb.
48835	+	+	+	+	+	+			ARF GAP zinc finger protein Gcs1
48852		+							unknown protein
48865	+	+	+	+	+	+			unknown protein
48883	+	+	+	+	+	+			unknown protein
48910	+	+	+	+	+	+			protein phosphatase 2A

48924	+	+	+	+	+	+			calcipressin
48951	+	+	+	+	+	+	1.266 down	2.215 down	unknown protein
49038	+	+	+	+	+	+			eukaryotic initiation factor 3. gamma subunit.
49048							2.152 down	6.111 down	RNA-dependent RNA-polymerase
49059	+	+	+	+	+	+			nuclear exosomal RNA helicase. related to N. crassa FRH
49081							3.051 down	21.255 up	GH74 Xyloglucanase CEL74a
49099	+	+	+	+	+	+			glutaredoxin
49112	+	+	+				2.004 down	2.831 down	Glutamate-1-semialdehyde aminotransferase
49149	+	+	+	+	+	+	1.817 up	3.015 up	Translocation protein Sec66
49193	+	+	+		+		1.274 up	2.066 up	GH17 glucan 1.3-β-glucosidase
49197	+	+	+	+	+	+			threonyl-tRNA synthetase. class IIa.
49205	+	+	+	+	+	+			cytochrome C peroxidase
49213	+	+	+	+	+	+			14-3-3 protein
49232	+	+	+	+	+	+			Zn2Cys6 transcriptional regulator
49274									glucan endo-1.3(4)-β-D-glucosidase
49295	+	+	+	+	+	+	1.128 down	2.004 down	unknown protein
49304	+	+	+	+	+	+			unknown protein
49308	+	+	+	+	+	+			unknown protein
49366							9.869 down	4.569 down	unknown protein
49372	+	+	+	+	+	+			Zn2Cys6 transcriptional regulator
49373	+	+	+	+	+	+			UTP15. encoding a component of the SSU processome
49399	+	+	+	+	+	+			Adenosine deaminase-related growth factor
49409	+	+	+	+	+	+			GH76 α-1.6-mannanase
49489	+	+	+	+	+	+			long-chain-fatty-acid-CoA ligase
49517		+					1.469 up	2.630 up	unknown protein

49522	+	+	+	+	+	+			Transcription factor TFIIS
49589	+	+	+	+	+	+	1.387 down	133.610 down	unknown protein
49594	+	+	+	+	+	+			mitochondrial import inner membrane translocase subunit TIM10
49649									unknown protein
49696	+	+	+	+	+	+			unknown protein with DnaJ domain
49746	+	+	+	+	+	+			unknown protein with Kelch repeats
49753	+	+	+	+	+	+	1.570 down	3.133 down	L-arabinitol 4-dehydrogenase
49770	+	+	+	+	+	+			ATP-dependent RNA helicase ROK1. putative
49795	+	+	+		+	+			unknown protein
49818	+	+	+	+	+	+			unknown protein
49832							1.155 down	2.510 down	QDE2. Argonaute-like protein. essential for quelling
49838	+	+	+	+	+	+			unknown protein
49854	+	+	+	+	+	+			BZIP transcriptional regulator
49864	+	+	+	+	+	+	1.322 up	2.180 up	unknown protein
49873	+	+	+	+	+	+			transcriptional regulator. unknown
49888									AAA ATPase
49898									glucosamine-6-phosphate isomerase
49909	+	+	+	+	+	+			Ribosomal protein S7
49918	+	+	+	+	+	+			transcriptional regulator. unknown
49923	+	+	+	+	+	+			26S proteasome regulatory complex subunit Rpn7
49928			+				1.144 down	7.578 up	unknown protein. only in Sordariomycetes
49946									Glutathione S-transferase
49970	+	+	+	+	+	+			H+ nucleoside cotransporter
49976							14.409 down	30.525 up	GH5 endo-β-1.4-glucanase EGL5/CEL45a
49979	+	+	+	+	+	+			OPT oligopeptide transporter. putative
49992	+	+	+	+	+	+			BTN1

50039	+	+	+	+	+	+			Mn superoxide dismutase. mitochondrial
50068	+	+	+	+	+	+			Histidine kinase
50071	+	+	+	+	+	+			protein kinase IME2. inducer of meiosis
50077							2.121 up	2.333 up	Amino acid transporters
50104							1.752 up	2.040 up	phosphatidyl synthase (A. fumigatus)
50131	+	+	+	+	+	+			peroxisomal biogenesis factor 11
50159									3-methyl-2-oxobutanoate hydroxymethyltransferase
50160		+							Palmitoyltransferase SWF1
50212			+						GT Glycosyltransferases not yet assigned to a family
50215	+		+			+	6.380 down	2.374 up	GH16 endo-1.3-β-D-glucosidase/1.3-glucan binding protein
50249	+	+	+	+	+	+	1.231 down	2.103 down	peptidase family M3
50268	+	+	+	+	+	+			sir2 family histone deacetylase
50299	+	+	+	+	+	+			unknown protein with TPR repeats
50316	+	+	+	+	+	+			unknown protein
50323							2.246 up	3.620 up	OOC1
50335	+	+	+	+	+	+			cdc42
50390									5' nucleotidase
50429	+	+	+	+	+	+	1.620 up	3.515 up	unknown protein
50449	+	+	+	+	+	+			unknown protein
50491	+	+	+	+	+	+			unknown protein
50516	+	+	+	+	+	+	1.621 up	2.032 up	ATPase. V-type
50531	+	+	+	+	+	+			2-oxoglutarate dehydrogenase-like protein
50536	+	+	+	+	+	+			unknown protein
50539	+	+	+	+	+	+			unknown protein
50542	+	+	+	+	+	+			ABC transporter
50583							1.556 down	3.869 down	Guanine deaminase (GDEase). an aminohydrolase responsible for the conversion ofGuani
50593							2.758 up	2.845 up	5' nucleotidase

50607	+	+	+	+	+	+			Sterol O-acyltransferase/Diacylglycerol O-acyltransferase
50616	+	+	+	+		+	1.118 up	8.243 down	Coenzyme A transferase
50618	+	+	+	+	+	+			MFS permease
50625	+	+	+	+	+	+	1.201 down	2.018 down	unknown protein
50647									ERAD-associated E3 ubiquitin-protein ligase HRD1
50707	+	+	+	+	+	+			unknown protein with WD repeats
50712	+	+	+	+	+	+			RNA binding protein
50786	+	+	+	+	+	+	3.020 up	2.845 up	Flavin-linked sulfhydryl oxidase
50793									tyrosinase
50882	+	+	+	+	+	+			pre-mRNA-splicing factor CWC25. putative
50894									MFS permease
50913	+	+	+	+	+	+			Peptidase C50. separase bimB
50947	+	+	+			+			cell division cycle protein 123 . putative
50996	+	+		+		+	4.528 down	7.482 down	unknown protein. C2 domain
51103	+	+	+	+	+	+			Tryptophan synthase
51110	+	+	+	+	+	+			IndA1 amino acid transporter
51130	+	+	+	+	+	+	1.063 down	2.182 up	cytochrome c
51153	+	+	+	+	+	+			GT α-1.2-mannosyltransferase
51171									unknown protein
51197	+	+	+	+	+	+			unknown protein with CCHC finger
51212	+	+	+	+	+	+			Ribosomal protein L5
51217									sulfate transporter. putative
51228	+	+	+	+	+	+			histone H1-binding protein. putative
51287	+	+	+	+	+	+			histone-lysine N-methyltransferase SET9
51365	+	+	+	+	+	+			Subtilisin-like protease PPRC1
51375	+	+	+	+	+	+	1.968 up	3.018 up	unknown protein
51378	+	+	+	+	+	+			Ubiquitin-like modifier-activating enzyme atg-7. putative

51407	+	+	+	+	+	+	1.753 down	2.200 down	unknown protein
51415	+	+	+	+			1.841 down	2.361 down	unknown protein
51420	+	+	+	+	+	+			ribosomal RNA adenine methylase transferase.
51426	+	+	+	+	+	+	1.256 up	2.757 up	Ribosomal protein L30 based on homology with similar proteins in other fungi.
51430	+	+	+	+	+	+			ribosomal protein L10e.
51443	+	+	+	+	+	+			unknown protein
51455	+	+	+	+	+	+			ATP-dependent RNA helicase DBP5
51483									Haloacid dehalogenase-like hydrolase
51492	+	+	+	+	+	+			Chitin synthase
51499	+	+	+	+	+	+			ketol-acid reductoisomerase. mitochondrial precursor
51528	+	+	+	+	+	+			unknown protein
51558							1.335 down	2.630 down	unknown protein
51562	+	+	+	+	+	+			choline kinase . putative
51603		+	+				1.097 down	2.285 down	unknown protein
51650		+	+						unknown protein
51654							1.231 down	2.496 down	unknown protein
51686		+							Dienelactone hydrolase
51697	+	+	+	+	+	+			unknown protein
51707	+	+	+	+	+	+			mitochondrial import inner membrane translocase subunit tim-17
51722	+	+	+	+	+	+			C2H2 transcriptional regulator
51734	+	+	+	+	+	+			Vesicle coat complex COPI. beta subunit
51806	+	+	+	+	+	+			Phosphatidate cytidylyltransferase
51846							1.113 down	2.358 down	Zn2Cys6 transcriptional regulator
51868	+	+	+	+	+	+			unknown protein
51893		+	+						peroxidase/hem containing

51907	+	+	+		+				Zn2Cys6 transcriptional regulator
51946	+	+	+	+	+	+			aminoacyl-tRNA synthetase
51967	+	+	+	+	+	+			class I peptide chain release factor.
51998									amidohydrolase family protein
52012									unknown protein
52021	+	+	+	+	+	+			protein kinase
52050	+	+	+	+	+	+			cyclophilin type peptidyl-prolyl cis-trans isomerase/CLD
52055	+	+	+	+	+	+			mitochondrial NADP-dependent isocitrate dehydrogenase
52073	+	+	+	+	+	+	2.362 down	2.743 down	sulfatase
52126	+	+	+	+	+	+			unknown protein
52130	+	+	+	+	+	+			Calcineurin B subunit. Protein phosphatase 2B subunit
52144	+	+	+	+	+	+			serine/threonine protein phosphatase type 5
52156	+	+	+	+	+	+			unknown protein
52165		+	+						unknown protein. 1 TM
52172	+	+	+	+	+	+			unknown protein
52185	+	+	+	+	+	+			actin filament-coating protein tropomyosin. putative
52208	+	+	+	+	+	+			Vesicle coat complex COPII. Sec23
52222							1.733 down	2.984 down	ankyrin repeat domain-containing protein
52248	+	+	+	+	+	+			ribosomal protein L1.
52267	+	+	+						mannitol-1-phosphate dehydrogenase
52315							1.038 down	1882.167 up	copper transporter Ctr
52334	+	+	+	+	+	+			BAR domain-containing protein
52337	+	+	+	+	+	+			Thioredoxin-like protein
52349	+	+	+	+	+	+			unknown protein
52368	+	+	+	+	+	+			Zn2Cys6 transcriptional regulator
52375	+	+	+	+	+	+			Aldehyde dehydrogenase
52432	+								Cytochrome P450 CYP2 subfamily

52438				+	+	+	+		+			unknown protein
52446				+	+	+	+		+			unknown protein
52451				+	+	+	+	+	+			ER lumen protein retaining receptor Erd2
52456				+	+	+	+	+	+			Co-chaperone for Hsp40p
52463				+	+	+	+	+	+			zinc metalloprotease
52476										38.161 up	23.863 up	unknown protein
52489												cytosin/purin permease
52499				+	+	+	+	+	+	1.035 down	2.786 down	C2H2 transcriptional regulator
52505				+	+	+	+	+	+	1.315 up	2.034 up	unknown protein
52520				+	+	+	+	+	+			Cyclin
52521				+	+	+	+					unknown protein
52532				+	+	+	+	+	+			unknown protein
52537				+	+	+	+	+	+			unknown protein
52539				+	+	+	+	+	+			unknown protein
52550				+	+	+	+	+	+			Epsin-like protein.Ent2. involved inEndocytosis and actin patch assembly
52553				+	+	+	+	+	+			unknown protein
52587				+	+	+	+	+	+			unknown protein
52665				+	+	+	+	+	+			Peptidase C19. ubiquitin carboxyl-terminal hydrolase 2. putative
52670				+	+	+	+	+	+			Mitochondrial carrier protein
52701				+	+	+	+	+	+			unknown protein
52709				+	+	+	+	+	+			unknown protein
52718				+	+	+	+	+	+			short chain dehydrognease/reductase
52763			+	+	+	+	+	+	+			60s ribosomal protein L29 based on homology to the L29 protein of yeast.
52829				+	+	+	+	+	+			serine incorporator
52839				+	+	+	+	+	+			unknown protein
52841				+	+	+	+	+	+			Zn2Cys6 transcriptional regulator
52847	+	+	+	+	+	+	+	+	+			NADH-ubiquinone oxidoreductase 299 kDa subunit. putative
52875				+	+	+	+	+	+	1.522 down	3.806 down	Zn2Cys6 transcriptional regulator

52924			+						transcriptional activator. zinc finger. NF-X1-type
52956		+							3'-5' exonuclease
52960									unknown protein
52972									unknown protein
52976	+	+	+	+	+	+			nucleolar RNA binding protein NifK
52979	+	+	+						unknown protein
53004	+	+	+	+	+	+	1.103 up	2.307 down	protein tyrosine phosphatase activity. M phase of mitotic cell cycle
53025									SAM-dependent methyltransferase
53027	+	+	+	+	+	+			unknown protein
53029							1.115 down	2.011 up	ß-lactamase-like protein
53046	+	+	+	+	+	+			Endoplasmic reticulum vesicle protein. Erv25
53053							4.738 up	5.307 up	unknown protein
53067									Zn2Cys6 transcriptional regulator
53076	+	+	+	+	+	+			unknown protein
53079							4.105 down	5.546 down	NADP/FAD dependent oxidoreductase
53080	+	+	+	+	+	+			unknown protein
53091									Threonine/serine dehydratases
53103	+	+	+	+	+	+			cyclophilin-type peptidyl-prolyl cis-trans isomerase
53105	+	+	+	+	+	+			DNA polymerase alpha catalytic subunit
53106									Zn2Cys6 transcriptional regulator
53123	+	+	+	+	+	+			dihydrodipicolinate synthase. putative
53125	+	+	+	+	+	+			Mitochondrial substrate carrier
53133	+	+	+	+	+	+			unknown protein
53153							1.092 down	2.593 down	unknown protein
53167									unknown protein
53168							1.024 down	3.572 up	Cytochrome P450. putative

53169		+								Catalytic (alpha) subunit of C-terminal domain kinase I (CTDK-I)
53172										lariat debranching enzyme domain-containing protein
53186								1.317 up	4.326 up	PL20 glucuronan lyase A
53187	+	+								UreD urease accessory protein
53220										unknown protein
53226	+	+	+	+	+	+				unknown protein
53238	+		+			+				GPCR. secretin like
53239										unknown protein
53246	+	+	+	+		+				unknown protein
53254	+	+	+	+	+	+				Signal peptidase Sec11
53259	+	+	+	+	+	+				unknown protein
53267										unknown protein. only in fungi
53282								1.047 down	2.361 up	NAD(P)H-dependent FMN reductase LOT6. putative
53286	+	+	+	+	+	+				3' exoribonuclease family protein
53302										unknown protein
53314								1.507 up	2.126 up	unknown protein
53316	+	+	+	+	+	+				TRAPP complex component Bet5
53318	+	+	+	+	+	+				cytochrome c oxidase subunit Va
53331										short chain dehydrogenase/reductase
53334	+	+	+					1.530 up	2.122 up	unknown protein
53342	+	+	+	+	+	+				Peroxin-3
53343	+	+	+	+	+	+				Zinc-dependent metalloprotease. ADAM_fungal subgroup
53349	+	+	+	+	+	+				Histidine kinase. part of a two-component phosphorelay system involved in signal transdu
53350	+	+	+	+	+	+				39S ribosomal protein L53/MRP-L53. putative
53360								1.942 down	2.594 down	SSCRP
53366	+	+	+	+		+		1.353 up	2.151 up	translation initiation protein Sua5p.
53372	+		+	+		+		2.753 down	2.231 down	acyltransferase 3

53373		+	+	+	+	+	+	1.248 down	3.435 down	CoA-transferase family III
53378								1.733 up	3.298 up	siderophore transporter
53381										SNF2 family DNA-dependent ATPase domain-containing protein
53395		+	+	+	+	+	+			unknown protein
53401								1.200 up	2.706 down	unknown protein
53402		+	+	+	+	+	+			Serine/threonine protein kinase
53428								1.005 up	9.099 down	SAM-dependent metyhltransferase
53430			+							Flavoprotein monooxygenase.
53431		+								unknown protein
53434										MFS permease
53437		+	+	+	+	+	+			RNA polymerase II-associated protein.
53443		+	+	+	+	+	+			unknown protein
53446		+	+	+	+	+	+			RRP9. encoding a protein involved in pre-rRNA processing. associated with U3 snRNP; com
53452	+							1.070 up	3.399 up	unknown protein
53462		+	+	+	+	+	+			unknown protein
53468										Lif1p of Saccharomyces cerevisiae. a protein involved in DNA double strand break repair;
53475				+				2.083 down	2.851 down	MFS permease
53481		+	+	+	+	+	+			unknown protein
53484		+	+	+	+	+	+			transcriptional regulator transcriptional regulator APSES type
53492		+	+	+	+	+	+			unknown protein
53495										unknown protein
53498		+	+	+						ubiquitin related modifier 1
53499										unknown protein
53500		+	+	+	+			1.012 down	6.223 down	unknown protein
53501		+	+	+				1.921 down	2.097 down	unknown protein

53503									unknown protein
53525	+	+	+				1.224 up	2.126 up	unknown protein (tyrosin phosphatase domain)
53526		+							Zinc-containing alcohol dehydrogenase
53529	+	+	+	+	+	+			low temperature viability protein
53535	+	+	+	+	+	+			v-SNARE Sec20
53542	+	+	+	+	+	+	1.254 down	2.746 down	GH76 α-1.6-mannanase
53554	+	+	+	+	+	+			20S proteasome beta-type subunit Pre1
53558									MDR-type ABC transporters
53561							2.483 down	4.876 down	unknown protein
53562	+	+	+	+	+	+			rho4
53567	+	+	+	+	+	+			glutathione reductase
53569	+	+	+	+		+			light-regulation protein. related to Drosophila TIMELESS
53570									unknown protein
53576	+	+	+	+	+	+			unknown protein
53585	+	+	+	+	+	+			transcriptional regulator. unknown
53596	+	+	+	+	+	+	1.330 up	2.044 up	unknown protein
53601	+	+	+	+	+	+			Synaptobrevin/VAMP-like protein
53605									unknown protein
53611							2.105 down	4.822 down	MFS permease
53615	+	+	+	+	+	+	1.383 down	2.620 down	unknown protein with WD repeats
53628	+	+	+	+	+	+			histone H3 methyltransferase complex and RNA cleavage factor II complex. subunit SWD2
53647	+	+	+	+	+	+			serine/threonine-protein kinase hal4
53659	+	+	+	+	+	+			DNA repair protein (Tof1)
53660									DEAD/DEAH box helicase
53665							1.329 up	4.049 up	unknown protein
53669	+	+	+	+	+	+			eukaryotic translation initiation factor 3 subunit F

53672	+	+	+	+	+	+	1.729 down	2.741 down	unknown protein
53673							1.835 down	2.072 up	NADP:D-xylose dehydrogenase [Hypocrea jecorina]
53684									unknown protein
53685	+	+	+	+	+	+			phosphodiesterase/nucleotide pyrophosphatasetype 1
53701	+	+					1.149 up	2.411 up	unknown protein
53721									DNA replication complex GINS protein SLD5. putative
53722	+	+	+	+	+	+			unknown protein
53728	+	+	+	+	+	+			inorganic pyrophosphatase
53731	+	+	+						GH5 endo-β-1.4-glucanase
53747							1.468 up	2.879 down	unknown protein
53776									Protein kinase
53777							2.621 down	10.284 down	unknown protein
53785	+	+	+	+	+	+			unknown protein
53796	+	+	+	+	+	+			unknown protein
53811	+	+	+	+	+	+			unknown protein
53812	+	+	+	+	+	+			unknown protein
53815									isochorismatase family hydrolase. putative
53818									ABC transporter superfamily?!
53824							9.191 down	4.343 down	unknown secreted protein
53848	+	+	+	+	+	+			unknown protein
53858									unknown protein
53859							1.176 down	3.203 down	C-4 sterol methyl oxidase
53862							1.501 up	3.430 up	unknown protein with WD repeats
53863							1.246 down	2.616 up	Flavoprotein monooxygenase

53868							1.960 down	3.207 down	NADH:flavin oxidoreductase/NADH oxidase
53870	+	+	+	+	+	+			unknown protein
53872									Dihydroxy-acid dehydratase
53877		+							Zn2Cys6 transcriptional regulator
53888	+	+	+	+	+	+	1.981 down	2.663 down	pirin
53893	+	+	+	+	+	+			unknown protein
53900									unknown protein
53902	+	+	+	+	+	+			histidine kinase class IV Fos-1
53903							1.980 down	8.402 down	MFS permease
53939							1.238 up	2.099 down	DNA polymerase y family member involved in translesion synthesis during DNA repair
53947	+	+	+	+	+	+			HFBs
53952	+	+	+	+	+	+			unknown protein
53956	+	+	+			+	1.231 up	2.129 down	unknown protein
53961							13.697 down	8.413 down	Aspartyl protease
53964							1.471 up	6.049 up	oxalate decarboxylase
53972	+	+	+	+	+	+			ATP-dependent RNA helicase DBP8 (EC 3.6.4.13)
53981	+	+	+	+	+	+			unknown protein
53986									deounknown proteinribonuclease TatD
53989							1.298 down	2.593 down	pfkB family kinase. putative
53995									RAI1
54005									MFS permease
54007									transcriptional regulator HMG type
54036									MFS permease
54042	+	+	+	+	+	+			Component of oligomeric golgi complex Cog4/Sec38

54048										2.510 down	5.314 down	unknown protein
54052										2.977 up	18.694 up	unknown protein
54064				+	+	+	+	+	+			mitochondrial ATP-dependent RNA-helicase mrh4
54071												NAD+ synthetases/Nitrilase/cyanide hydratase/apolipoprotein N-acyltransferase
54086												short chain dehydrognease/reductase
54089										1.367 up	3.852 up	unknown protein
54128										1.056 down	2.477 down	IlvA Threonine dehydratase
54129												Heme peroxidase. unknown in Sordariomycetes
54134												unknown protein
54144						+				1.097 down	3.141 up	ferric reductase
54157					+							unknown protein
54160										1.095 down	3.539 up	GliI [Aspergillus fumigatus]
54166										1.179 down	2.905 down	Cytochrome P450
54171												unknown protein
54179										1.067 up	2.827 up	glutathione S-transferase
54181				+	+	+	+	+	+			siroheme synthase
54195				+	+	+	+	+	+			Cyclin-K . putative
54198				+	+	+						unknown protein
54202				+	+	+	+	+	+	1.098 down	2.275 down	unknown protein. contains DUF155 domain
54208												unknown protein
54219												CE5 acetyl xylan esterase
54223	+	+	+									short chain dehydrogenase/reductase
54226										1.394 down	2.478 up	unknown protein
54227										9.221 down	7.643 down	short-chain dehydrogenase/reductase

54230							1.777 down	3.192 down	NACHT domain WD40 repeat-containing protein. related to HET
54239							4.733 down	3.356 down	Multicopper oxidases
54242									GH55 β-1.3-glucanase
54244									unknown protein
54256	+	+	+	+	+	+			RNA binding protein
54260									potential indoleamine 2.3-dioxygenase
54283	+	+	+	+	+	+			Utp18p. a component of the SSU processome
54285	+	+	+	+	+	+	1.239 down	2.840 down	unknown protein
54329	+	+	+	+	+	+			unknown protein
54330	+	+	+	+	+	+			Member of SIR2 proteins
54335	+	+	+	+	+	+	1.329 down	2.742 down	unknown protein
54349	+	+	+	+	+	+	1.637 up	3.184 up	small nuclear ribonucleoprotein F
54352							2.527 down	13.359 down	unknown protein.
54365	+	+	+	+	+	+			Amino acid transporter
54366							1.169 down	2.940 down	ceramidase. nonlysosomal
54372									unknown protein
54384	+	+	+	+	+	+			unknown protein
54391		+							unknown protein
54393									unknown protein
54395	+	+	+	+		+			Transcriptional regulator FlbA
54407	+	+	+	+	+	+			unknown protein
54408	+	+	+	+	+	+			guanylate kinase. putative
54419		+							RNase3 domain-containing protein
54426							1.145 up	2.457 up	unknown protein
54427	+	+	+						unknown protein

54437									Zn2Cys6 transcriptional regulator
54441	+	+	+	+	+	+			HECT-domain-containing protein
54444							1.005 down	3.004 down	unknown protein
54448									deounknown proteinhypusine synthase
54450	+	+	+	+	+	+			mRNA capping enzyme
54454	+	+	+	+	+	+			26S proteasome regulatory complex subunit Rpn6
54458		+							unknown protein
54461							1.056 down	7.740 up	MFS permease
54462							1.567 up	3.654 up	unknown protein
54469	+	+	+	+	+	+	1.174 down	2.646 down	Vacuolar transporter chaperone 4 (vtc4). putative
54471	+	+	+	+	+	+			unknown protein
54487									unknown protein
54502									Zn2Cys6 transcriptional regulator
54508									Molybdopterin synthase catalytic subunit
54511									unknown protein. contains BTB/POZ domain (=protein binding)
54514	+	+	+	+	+	+			unknown protein
54521	+	+	+	+	+	+			ATP binding protein
54525	+	+	+	+	+	+			AldedhAldehyde dehydrogenase family
54535	+	+	+	+	+	+			unknown protein
54541	+	+	+	+	+	+			protein for structural maintenance of chromosome protein 3 (sister chromatid cohesion complex Cohesin. subunit SMC3)
54550	+	+	+	+	+		1.001 down	2.149 down	short chain dehydrognease/reductase
54554	+	+	+	+	+	+			GTP cyclohydrolase 1
54564	+	+	+	+					Pro_dhProline dehydrogenase aligned
54567	+	+	+	+	+	+			Zn2Cys6 transcriptional regulator
54583	+	+	+						unknown protein
54589	+	+	+	+	+	+			type 2A phosphatase activator TIP41

54596	+	+	+	+	+	+			unknown protein
54598	+	+	+	+	+	+			snf5/smarcb1/ini1
54611	+	+	+	+	+	+	1.086 up	3.175 up	unknown protein
54615	+	+	+	+	+	+			unknown protein
54616									unknown protein
54622	+		+				2.553 down	4.686 up	unknown protein
54632	+	+	+						MFS permease
54633	+	+	+	+	+	+	1.339 down	2.183 down	GT ß-glycosyltransferases
54659							1.315 down	3.606 down	unknown protein
54667							6.311 up	11.946 up	acyl-CoA synthetase
54669	+	+	+	+	+	+	1.552 up	9.480 up	Golgi matrix protein. rud3. involved in the structural organization of the cis-Golgi
54670	+	+	+	+	+	+			SWI-SNF complex subunit (Snf5). putative
54674	+	+	+	+	+	+	1.375 up	2.033 down	unknown protein
54675			+						Transcriptional regulator PacG/VIB-1
54676	+	+	+	+	+	+	1.169 up	2.232 up	COX17. cytochrome C oxidase assembly protein
54683	+	+	+	+	+	+			oxysterol binding protein. contains an Ankyrin and a Pleckstrin-homology related domain
54686	+	+	+	+	+	+			unknown protein
54694							4.224 down	10.692 down	aryl-alcohol dehydrogenases
54703	+	+	+	+		+	2.647 down	2.513 down	C2H2 transcriptional regulator
54709	+	+	+	+	+	+			cell cycle control protein cwf14
54710	+	+	+	+	+	+			unknown protein
54722		+	+	+		+			unknown protein
54723							1.025 down	2.698 up	SSCRP
54736	+	+	+	+	+	+			ATP-dependent RNA helicase ded-1

54761							3.862 down	3.324 up	quercetin 2.3-diounknown proteingenase
54768							6.432 up	4.254 up	unknown protein
54784							1.049 up	3.593 up	unknown protein
54789							1.464 up	3.105 up	short chain dehydrogenase/reductase
54790							1.395 down	3.263 down	unknown protein
54809									bicarbonate transporter
54813		+							unknown protein
54819									unknown protein
54846							1.025 down	3.366 down	half-sized ABC transporter
54850									AT DNA binding protein. putative
54852	+	+	+	+	+	+			SYF2 splicing factor
54858			+						unknown protein
54865	+	+	+	+		+			Amino acid transporters
54868	+	+	+	+	+	+			unknown protein
54870							1.025 down	2.143 down	acetate—CoA ligase
54886									unknown protein
54890	+	+	+	+	+	+			unknown protein
54893							1.138 up	2.349 down	unknown protein
54902							1.062 up	3.137 down	unknown protein
54926									unknown protein
54954							1.163 down	2.740 down	MRP-type ABC transporter
54961	+	+	+	+	+	+			unknown protein
54962									iron transporter
54972							3.231 down	7.847 down	MFS permease

54991									short chain dehydrogenase/reductase
54997									isoamyl alcohol oxidase
54999		+							MFS permease
55005		+	+						GT22 ALG9 mannosyltransferase
55029	+	+	+	+	+	+			rRNA processing protein Rrp8
55034	+	+	+	+	+	+	1.932 down	3.027 down	unknown protein
55036							2.055 up	3.968 up	unknown protein
55039		+					3.534 up	3.347 up	unknown protein
55041							1.115 down	2.113 down	unknown protein
55049									casein kinase 1 delta
55052									unknown protein
55055	+	+	+	+	+	+			MTHFRMethylenetetrahydrofolate reductase
55060	+	+	+	+	+	+	2.487 up	2.002 up	kinesin heavy chain
55062									unique protein
55067	+	+	+	+	+	+			import inner membrane translocase subunit tim-21. mitochondrial . putative
55073	+	+	+	+	+	+			spo7
55077									MFS permease
55088	+	+	+	+	+	+			NADH-ubiquinone oxidoreductase 18 kDa subunit. putative
55099									polynucleotide adenylyltransferase.
55105							1.935 down	2.440 down	Zn2Cys6 transcriptional regulator
55114	+	+	+	+	+	+			Nucleoside diphosphatase involved in protein glycosylation in the Golgi
55123									unknown protein
55125	+	+	+	+	+	+			unknown protein
55126							2.304 down	2.382 down	unknown protein
55161									D-lactate dehydrogenase. mitochondrial precursor
55172	+	+	+	+	+	+	1.324 up	3.179 up	unknown protein
55179									Arginase family protein

55180	+	+	+	+	+	+			unknown protein
55183	+	+	+	+	+	+			unknown protein
55185	+	+	+	+	+	+			RNA polymerase Rpb5
55190									aminotransferase
55191	+	+	+	+	+	+			inner membrane magnesium transporter MRS2
55193							2.101 down	2.923 down	unknown protein
55201									histone H4. putative
55204	+	+	+	+	+	+			vacuolar ATP synthase subunit F
55205	+	+	+	+	+	+			unknown protein
55208	+	+	+	+	+	+			maintenance of ploidy protein mob1
55213	+	+	+				1.066 up	2.472 down	tRNA-specific adenosine deaminase
55224	+	+	+	+	+	+			unknown protein
55226		+	+						unknown protein
55237	+	+	+	+	+	+			unknown protein
55240							6.843 down	3.475 down	large-conductance mechanosensitive channel
55242	+	+	+	+	+	+			unknown protein
55245									ribokinase
55252									unknown protein
55263	+	+	+	+	+	+			ubiquitin-conjugating enzyme
55272							2.401 up	2.322 up	unknown protein
55274		+	+		+				Zn2Cys6 transcriptional regulator
55276									thioesterase superfamily protein
55279	+	+	+	+	+	+	1.618 down	2.786 down	unknown protein
55306									Acyl-CoA synthetase
55318	+	+	+	+	+	+			fatty acid elongase-like protein
55319									GH54 α-L-arabinofuranosidase ABF3
55321	+	+	+	+	+	+			phosphatidylinositolglycan class N. putative

55335			+	+	+	+	+	+			nucleotide binding protein Nbp35. putative
55341			+	+	+	+	+	+			Chitin synthase. homolog of N.CrassaChs2.Chitin synthase
55349											RNAse P Rpr2/Rpp21/SNM1 subunit domain-containing protein. putative
55351				+							Alkyl hydroperoxide reductase/peroxiredoxin
55353			+	+	+	+	+	+	1.233 down	3.611 down	pathotenate kinase
55358											flavin-binding monooxygenase. putative
55362			+	+	+	+	+	+	2.356 down	2.012 down	Heat shock protein 70
55365			+	+	+			+			DASH complex subunit Dad4
55374									1.298 down	2.104 up	unknown protein
55375			+		+						unknown protein
55381			+	+	+	+	+	+			unknown protein
55384			+	+	+						Endoribonuclease YSH1 (mRNA 3'-end-processing protein YSH1)
55401			+	+	+	+	+	+			unknown protein
55406			+	+	+	+	+	+			unknown protein
55407									2.000 down	2.158 down	MFS permease
55415	+	+	+	+	+	+	+	+			ARF GEF2 Gea2
55417			+	+	+	+	+	+			dynein heavy chain
55421			+	+	+	+	+	+			unknown protein
55422			+	+	+						elongation factor 2 kinase. putative
55443			+	+	+				2.239 up	2.233 up	unknown protein
55454									1.195 down	3.038 down	unknown protein
55467			+	+	+	+	+	+			CDC45-like protein
55478											unknown protein. 2 TM
55508			+	+	+	+	+	+			AFG1-like ATPase
55511			+	+	+	+	+	+			cation efflux family protein
55519			+	+	+	+	+	+			unknown protein

55534	+	+	+	+	+	+			GlcNAc-1-P transferase
55543									serine/threonine protein kinase with similarity to casein kinase I
55545	+	+	+	+	+	+			Unknown protein with WD-40 repeats
55548	+	+	+	+	+	+			RNA polymerase II transcription mediator
55550	+	+	+	+	+	+			aps2. of the adaptor protein complex AP-2 of clathrin-coated vesicles. small subunit
55552	+	+	+	+	+	+			Nascent polypeptide-associated complex subunit beta (NAC-beta)
55559									unknown protein
55561									PTH11 GPCR
55566		+					1.272 up	2.085 up	multicopper oidase
55570	+	+	+	+	+	+			Exocyst component Exo70
55575	+	+	+	+	+	+			unknown protein
55584							1.131 down	2.033 down	unknown protein
55589							1.915 up	2.906 up	3' exoribonuclease involved in RNA processing during translation.
55592	+	+	+	+	+	+			Mob1/phocein family protein
55595	+	+	+	+	+	+	1.311 up	2.042 up	Ca2+ transporter
55597	+	+	+	+	+	+	1.384 down	2.787 down	unknown protein
55599	+	+					1.710 down	2.246 down	RNA 12 protein
55627	+	+	+	+	+	+			CDP-alcohol phosphatidyltransferase
55629									unknown protein
55630							3.870 down	7.837 down	monocarboxylate transporter
55631			+				1.531 down	2.294 down	phospholipase
55634							1.319 down	2.506 down	MFS permease
55636		+		+			1.750 down	2.511 down	MRP-type ABC transporter
55644	+	+	+	+	+	+			20S proteasome alpha subunit Pup2

55660	+	+	+	+	+	+			Transcription factor TFIIS
55666							1.057 up	4.246 up	unknown protein with WD repeats
55668		+							unknown protein
55669	+	+	+	+	+	+			mRNA decapping hydrolase. putative
55671							1.665 up	2.237 up	unknown protein
55679	+	+	+	+	+	+			unknown protein
55681									unknown protein
55698									unknown protein
55699									unknown protein
55706	+	+	+	+	+	+			SCF ubiquitin ligase complex subunit. cullin
55709		+							unknown protein
55719							2.340 up	2.304 up	Protein kinase
55723									PotE
55731	+	+	+	+	+	+			cation channel family protein
55733		+							GH92 α-1.2-mannosidase
55747									ABC transporter
55752	+	+	+	+	+	+			ribosomal protein L29. Shows amino acid sequence similarity to S. cerevisiae mitochondri[illegible]
55759									Zn2Cys6 transcriptional regulator
55766	+	+	+	+	+	+			nucleolar GTP-binding protein 1
55774	+	+	+	+	+	+			Secretion related small GTPase
55782							1.283 up	5.432 up	dipeptidyl peptidase 5
55788	+	+	+	+	+	+			Ubiquitin-conjugating enzyme E2. putative
55790	+	+	+	+	+	+			N2.N2-dimethylguanosine tRNA methyltransferase.
55796	+	+	+	+	+	+			cyclin domain-containing protein
55802	+	+					1.351 up	6.833 up	GH76 α-1.6-mannanase
55803	+	+	+	+	+	+			protein kinase RIO1
55814									unknown protein
55818	+	+	+	+	+	+			unknown protein
55837	+	+	+			+			Adenine phosphoribosyl transferases
55838	+	+	+						unknown protein

55855									unknown protein
55857	+	+	+	+	+	+			Exocyst component Ex084
55868	+	+	+	+	+	+			PP2Ac. Protein phosphatase 2A homologues
55871							3.474 down	2.975 down	Short-chain dehydrogenase/reductase
55876									unknown protein
55881									unknown protein
55886									GH16 glucan endo-1.3(4)-β-D-glucosidase
55887							4.792 up	38.374 up	unknown protein. secreted
55891									UBA/THIF-type NAD/FAD binding fold
55902	+	+	+	+	+	+			Replication protein A2
55912	+	+	+	+	+	+			Cell division/GTP binding protein
55928	+	+	+	+	+	+			bifunctional carbamoylphosphate synthase/aspartate carbamoyltransferase
55939	+	+	+			+			ran binding protein in the microtubule-organising centre. putative
55945	+	+	+	+	+	+			Exocyst component Sec5
55950									unknown protein. 5 TM
55969	+	+	+	+	+	+			Translation initiation factor. eIF1A.
55981	+	+	+	+	+	+			tRNA cytosine-5-methylases
55990									unknown protein
55993	+	+	+	+	+	+			unknown protein
55999							2.100 down	2.335 up	GH27 α-galactosidase
56002									unknown protein
56003		+							RNA binding protein MSSP-2
56026	+	+	+	+	+	+	1.396 down	2.779 down	unknown protein
56033									unknown protein
56048									unknown protein
56064							1.179 down	2.519 down	unknown protein
56070	+	+	+	+	+	+			unknown protein

56074	+	+	+	+	+	+			EH domain-containing protein.End3. involved inEndocytosis. actin cytoskeletal organizatio
56077	+	+	+						Zn2Cys6 transcriptional regulator
56081									unknown protein
56089	+	+	+	+	+	+			unknown protein
56093							1.247 up	3.202 up	unknown protein
56095									MFS permease
56115	+	+	+	+	+	+			Spc97 / Spc98 family proteins involved in spindle pole body
56117		+					1.054 up	2.548 down	unknown protein
56118	+	+	+	+	+	+			unknown protein
56129									hexokinase
56140	+	+	+	+	+	+			unknown protein
56141	+		+			+	1.738 down	3.095 down	unknown protein
56146	+	+	+	+	+	+			unknown protein
56149	+	+	+	+	+	+			Protein with homology to anucleate Primary sterigmata Protein apsA (Aspergillus nidulan
56150									unknown protein
56169		+							vacuolar protein sorting protein (Vps36). putative
56176									MRP-type ABC transporter
56196	+	+	+	+	+	+			unknown protein
56203									dolichyl-phosphate-mannose a-mannosyltransferases
56206	+	+	+	+	+	+			unknown protein
56211							5.270 up	4.898 up	unknown protein
56214									Zn2Cys6 transcriptional regulator
56215		+							26S proteasome non-ATPase regulatory subunit 9. putative
56218							1.453 up	2.688 down	unknown protein
56236							1.201 down	2.746 up	SAM-dependent methyltransferases
56256	+	+	+	+	+	+	1.034 down	2.133 down	unknown protein

56259							1.422 up	3.484 up	unknown protein
56261	+	+	+	+	+	+			unknown protein
56267	+	+	+						pyridoxal kinase
56272									Cytochrome P450 monooxygenase
56278							1.217 up	3.688 up	unknown protein. in Sordariomycetes
56289							1.404 up	2.177 down	MFS permease
56291									SAM dependent methyltransferase
56314									Amino acid transporter PotE
56318	+	+	+	+	+	+			Cargo transport protein Emp24 (p24 protein family)
56326							1.370 up	2.884 up	Zinc-containing alcohol dehydrogenase
56328									GT31 b-glycosyltransferases
56341	+		+						unknown protein
56344	+	+	+	+	+	+			unknown protein
56345	+	+	+	+	+	+			ribosomal RNA methyltransferase RrmJ/FtsJ domain.
56350							1.175 down	2.450 up	cysteine synthase. putative
56354									histone-lysine N methyltransferase
56363	+	+				+			unknown protein
56370									Isocitrate/isopropylmalate dehydrogenase
56376	+	+	+	+	+	+	1.888 up	2.202 up	unknown protein
56384	+	+	+	+	+	+			Nucleolar protein that binds nuclear localization sequences
56396									unknown protein
56399							1.669 down	2.925 down	unknown protein
56408	+	+	+	+	+	+	1.081 up	2.056 down	unknown protein
56409	+	+	+	+	+	+			unknown protein
56418									GH55 β-1 3-glucanase
56421	+	+	+	+	+	+			mitochondrial carrier protein

56426	+	+	+	+	+	+	1.116 down	2.044 down	GCPR. mPR-type
56427	+	+	+				3.269 down	2.089 down	Arylacetamide deacetylase
56431									unknown protein
56432	+	+	+	+	+	+			FMN-dependent dehydrogenase
56434	+	+	+	+	+	+	1.327 down	3.728 up	FKBP-type peptidyl-prolyl isomerase. putative
56440									Ion transport protein
56448							2.402 down	2.663 up	GH18 chitinase CHI18-11
56467	+	+	+	+	+	+	1.678 up	2.430 up	Mitochondrial deoxynucleotide carrier protein
56469	+	+	+	+	+	+	1.172 up	2.548 down	unknown protein
56470									LdhA Lactate dehydrogenase and related dehydrogenases
56499							1.754 up	2.959 up	N-acyl-phosphatidylethanolamine-hydrolyzing phospholipase D. putative
56512	+	+	+	+	+	+			unknown protein
56515							1.767 down	9.316 down	Regulation of HR towards crossover
56525									UbiD family decarbounknown proteinlase
56531	+	+	+	+	+	+			unknown protein
56546							4.290 up	5.674 up	unknown protein. 3TM
56547		+							unknown protein
56559	+	+	+	+	+	+			unknown protein
56565	+	+	+	+	+	+			unknown protein
56568	+	+	+	+	+	+			unknown protein
56572		+					1.383 down	4.784 down	aminotransferase. classes I and II family
56582	+	+	+	+	+	+			unknown protein
56587							1.259 down	5.312 down	GCN5-related N-acetyltransferase
56593									unknown protein

56603	+	+	+	+	+	+	+	+	+			unknown protein
56605				+	+	+	+	+	+			ß-arrestin. probable adaptor and transducer involved in signalling. related to the E. nidula
56610												unknown protein
56614				+	+	+						unknown protein
56625										1.101 up	2.505 down	CoA-transferase family III
56631				+	+	+	+	+	+			ribulose-phosphate 3-epimerase
56638				+	+	+	+	+	+			animal a-N-acetylhexosaminyltransferases
56646										1.307 down	2.940 down	monoamine oxidase
56671				+	+	+	+	+	+	1.885 up	2.349 up	unknown protein
56682												unknown protein
56684												MFS permease
56687												cytochrome P450 protein
56690												mannose-1-phosphate guanylyltransferase. putative
56700												unknown protein
56717												unknown protein
56720				+	+	+	+	+	+			unknown protein
56726				+	+	+	+	+	+	1.287 up	2.717 up	Branched chain alpha-keto acid dehydrogenase complex. alpha subunit
56744				+	+	+	+	+	+			Calcium transporter
56758						+	+	+	+	1.330 up	2.799 up	unknown protein
56771												SSCRP
56772				+	+	+			+			transcriptional regulator. unknown
56780												DNA polymerase X family
56789				+	+	+	+	+	+			cell division control protein Cdc6
56792				+	+	+	+	+	+			SSCRP
56804												unknown protein
56812				+	+	+	+	+	+			GT α-1.2 glucosyltransferase
56819										2.546 down	3.415 down	glutathione-S-transferase

56830							1.988 down	2.437 down	dipeptidyl peptidase 5
56831	+	+	+	+	+	+	1.247 up	2.100 up	Co-chaperone Hsc20
56835									unknown protein
56838									short chain dehydrogenase/reductase
56839	+	+	+						Zinc-containing alcohol dehydrogenase
56840							6.499 down	6.709 up	unknown protein
56845									flavoprotein monooxygenase
56853							2.107 down	3.913 down	unknown protein
56856	+	+	+	+	+	+			DNA replication complex GINS protein (Psf2). putative
56860	+	+	+	+	+	+	1.964 down	3.588 down	unknown protein
56863	+	+	+	+	+	+	6.161 down	3.218 down	unknown protein
56864	+	+	+	+	+	+			unknown protein
56872	+	+	+	+	+	+			PP2Ac. Protein phosphatase 2A homologues. catalytic domain
56876	+	+	+	+	+	+			polysaccharide export protein CAP59
56894							1.156 up	2.606 up	GH18 chitinase CHI18-10
56896									unknown protein
56897									unknown protein
56911									Urea transporter
56920	+	+	+	+	+	+			Leucine aminopeptidase 1
56934	+	+	+	+	+	+			gluconokinase. thermoresistant glucokinase family
56942	+	+	+	+	+	+			unknown protein
56952	+	+	+						MFS permease
56966									Cytochrome P450. putative
56996							1.054 down	15.988 up	GH5 β-Mannanase MAN1

57002	+	+	+	+	+	+	1.266 down	2.875 down	unknown protein
57008							1.238 up	3.284 up	unknown protein
57010									prenyltransferase and squalene oxidase
57015									Amino acid transporters
57016	+	+	+	+	+	+			germinal center kinase. related to S. cerevisiae Kic1p
57034									unknown protein
57045							1.818 down	2.101 down	unknown protein
57049	+	+	+	+	+	+	1.578 down	2.408 down	tRNA selenocysteine-associated protein 1
57059	+	+	+	+	+	+			homeobox transcriptional regulator
57071	+	+	+	+	+	+			unknown protein
57078									unknown protein
57088									Sulfate/bicarbonate/oxalate exchanger SAT-1 and related transporters (SLC26 family)
57091	+	+	+	+	+	+			GDP-mannose transporter
57098									unknown protein
57100	+	+	+	+	+	+			unknown protein
57101							1.475 up	3.705 up	PTH11 GPCR
57112							1.156 down	2.420 down	HET and Ankyrin domain protein
57128	+	+	+	+	+	+	1.584 down	2.356 down	GH13 glycogen debranching enyzme
57129	+	+	+	+	+	+			Protein transport protein Yos1
57140	+	+	+	+	+	+			unknown protein
57141	+	+	+	+	+	+			FAD binding domain-containing protein
57167	+	+	+	+	+	+			SHR3. an ER membrane protein involved in packing of amino acid permeases into ER-Golg
57169	+	+	+	+	+	+			unknown protein
57178	+	+	+	+	+	+			nuclear mRNA splicing factor-associated protein. putative
57179							1.125 up	8.449 up	GH105/GH88 glycosyl hydrolase
57185							3.912 up	2.408 up	Amino acid transporters

57198	+	+	+	+	+	+	1.567 down	3.568 down	Rad10
57204									lipase. secreted
57208	+	+	+	+	+	+			ubiquitin-like modifier SUMO. putative
57217	+	+	+	+	+	+			unknown protein
57220	+	+	+						histone deacetylase
57234			+						unknown protein
57237									unknown protein
57252	+	+	+	+	+	+			polyadenylation factor subunit CstF64
57253	+	+	+	+	+	+			unknown protein
57263	+	+	+	+	+	+			protoporphyrinogen oxidase. putative
57274	+	+	+	+	+	+			unknown protein
57277									unknown protein
57280			+						unknown protein
57282	+	+	+	+	+	+			MYG1. putative
57286	+	+	+	+	+	+	2.161 up	2.381 up	unknown protein
57287	+	+	+	+	+	+			chitin synthase activator. putative
57291									unknown protein
57295	+	+	+	+	+	+			Transcription elongation factor spt-6
57302		+							dihydrofolate reductase
57322	+	+	+	+		+	1.005 down	2.955 down	ARO Transcriptional regulators containing a DNA-binding HTH domain and an aminotransf
57334	+	+	+	+	+	+			HECT-domain-containing protein
57335	+	+	+	+	+	+			unknown protein
57357	+	+	+	+	+	+	1.124 up	2.654 up	Glycine cleavage T protein (aminomethyl transferase). putative
57370	+	+	+	+	+	+			AMP-dependent synthetase and ligase
57383							1.846 down	2.443 down	unknown protein
57391	+	+	+	+	+	+			unknown protein
57397		+	+				2.316 up	2.850 up	unknown protein

57399	+	+	+	+	+	+			cAMP dependent protein kinase. protein kinase A. catalytic subunit
57402	+	+	+	+	+	+			SSCRP
57421	+	+	+	+	+	+			G2/mitotic-specific cyclin-B [Neurospora crassa OR74A]
57424	+	+	+	+	+	+			QIP. Putative exonuclease protein. involved in quelling
57430									Glutamate-cysteine ligase
57433							1.035 down	2.620 up	Peptidase S8 and S53. subtilisin. kexin. sedolisin
57435	+	+	+	+	+	+			unknown protein
57465	+	+	+	+	+	+	1.074 down	2.235 down	Vsp9 domain protein
57472	+	+	+	+					unknown protein
57474	+	+	+	+	+	+			unknown protein
57476	+	+	+	+	+	+			unknown protein
57488							1.149 down	2.350 down	Top3 gene
57494	+	+	+	+	+	+			UTP10. encoding a component of the SSU processome containing the U3 snoRNA
57508	+	+	+	+	+	+			Dynamin-related GTPase
57513	+	+	+	+	+	+			MAPKK. mitogen activated protein kinase kinase Ssk2p
57524							1.336 down	2.065 down	amidohydrolase 2
57526							8.271 down	5.647 down	GPCR. mating type pheromone G-protein coupled receptor
57527	+	+	+				1.029 down	3.217 down	unknown protein
57528									unknown protein
57534	+	+	+	+	+	+			Zn2Cys6 transcriptional regulator
57535		+							acylphosphatase. putative
57537							1.299 up	2.213 up	unknown protein
57555							2.737 down	3.347 down	cytochrome P450 monooxygenase
57558									unknown protein

57568		+	+				1.042 down	2.344 down	unknown protein
57574	+	+	+	+	+	+			unknown protein
57575							1.168 up	2.904 up	unknown protein
57580	+	+	+	+	+	+			unknown protein
57581	+	+	+	+	+	+			unknown protein
57592		+					1.009 down	2.373 down	unknown protein
57595	+	+	+	+	+	+			ribosomal protein L7/12 domain. Shares amino acid sequence similarity to S. cerevisiae m
57600	+	+	+	+	+	+			FACT complex protein (Facilitates chromatin transcription complex subunit SPT16)
57603	+	+	+	+	+	+	1.449 down	2.098 down	unknown protein
57608	+	+	+	+	+	+			SNF2 family helicase/ATPase. putative
57609	+	+	+	+	+	+			unknown protein
57613	+	+	+	+	+	+			exonuclease family protein
57631	+	+	+	+	+	+			unknown protein
57632	+	+	+	+	+	+			unknown protein. WD repeats
57643	+	+					1.743 down	18.548 down	unknown protein
57647									3-beta hydroxysteroid dehydrogenase/isomerase. putative
57666	+	+	+	+	+	+			unknown protein
57671		+							unknown protein
57676	+	+	+	+	+	+			Translation initiation factor 3. subunit i (elf-3i)
57696	+	+	+	+	+	+			unknown protein
57720	+	+	+	+	+	+			Swr1p complex component (Swc5). putative
57727	+	+	+	+	+	+			unknown protein
57728	+	+	+	+	+	+			NimA interactive protein. putative
57730	+	+	+	+	+	+			unknown protein
57735	+	+	+	+	+	+	1.398 down	2.025 down	GATA type transcriptional regulator
57737		+	+						unknown protein

57749							2.035 down	3.764 down	MFS permease
57750	+	+	+	+	+	+			unknown protein with WD repeats
57756			+						unknown protein
57760	+	+	+	+	+	+	1.349 down	2.915 down	Zn2Cys6 transcriptional regulator
57776									unknown protein
57784	+	+	+	+	+	+			Golgi apparatus membrane protein tvp-23
57823									unknown protein
57832		+							vacuolar ATPase assembly integral membrane protein VMA21. putative
57840	+	+							bZIP transcription factor
57855	+	+	+	+	+	+			unknown protein
57857									GH2 β-mannosidase
57860							1.153 down	3.578 down	Multicopper oxidases
57864									unknown protein
57865	+	+	+	+	+	+	1.281 up	2.816 up	unknown protein
57868							2.442 up	2.062 up	Delta 1-pyrroline-5-carboxylate reductase
57869	+	+	+	+	+	+			unknown protein
57870	+	+	+	+	+	+	2.398 up	3.119 up	histone H3
57886	+	+	+	+	+	+			unknown protein
57891	+	+	+	+	+	+	1.078 down	2.186 down	actinin actin binding and calcium-binding EF-hand domains
57904	+	+	+	+	+	+			checkpoint serine/threonine-protein kinase BUB1. putative
57905	+	+	+	+	+	+			unknown protein
57914							1.158 up	18.424 down	Esterase/lipase/thioesterase
57923	+	+	+	+	+	+			unknown protein
57935	+	+	+	+	+	+			snf2 chromatin remodeling protein
57936	+	+	+	+	+	+			unknown protein
57940	+		+	+		+			alternative oxidase aox1

57947	+	+	+	+	+	+			BioF -keto--aminopelargonate synthetase and related enzymes
57957	+	+	+	+	+	+			transcriptional regulator. unknown
57965							1.179 up	2.203 down	DSBA family oxidoreductase. putative
57975	+	+	+	+	+	+	1.804 up	3.326 up	phospholipase C. related to Aspergillus fumigatus phosphatidylinositol phospholipase C
57997	+	+	+	+	+	+			acting-binding cofilin/tropomyosin domain
58011		+	+						C2H2 conidiation transcription factor FlbC
58016							1.417 up	3.118 up	unknown protein
58026							1.086 down	2.694 down	glutathione S-transferase domain-containing protein
58036	+	+	+	+	+	+			prefoldin subunit 1. putative
58051							1.387 down	2.606 down	unknown protein
58055	+	+	+	+	+	+			UbiA prenyltransferase containing 9 transmembrane domains; related to S. cerevisiae Par
58058									unknown protein
58063	+	+	+	+	+	+			unknown protein
58065		+							unknown protein
58066									short chain dehydrogenase/reductase
58068	+	+	+		+				haloacid dehalogenase-like hydrolase
58073	+	+	+	+	+	+			uroporphyrinogen decarboxylase
58076	+	+	+	+	+	+			apurinic/apyrimidinic endonuclease-like protein
58077	+	+	+	+	+	+			unknown protein
58114	+	+	+	+	+	+			unknown protein
58115									unknown protein
58117									GH89 α-N-acetylglucosaminidase
58119									unknown protein
58125	+	+	+	+	+	+			20S proteasome beta-type subunit Pup3
58130	+	+	+	+	+	+	1.563 down	2.246 down	bHLH transcriptional regulator
58150	+	+	+	+	+	+			Peptidase C19. ubiquitin carboxyl-terminal hydrolase 2
58158									OPT family small oligopeptide transporter

58161	+	+	+	+	+	+			GTPase-activating protein (GAP) for Rab family members.Gyp5. involved in ER toGolgi traf
58162									alkaline phytoceramidase
58170	+	+	+	+	+	+			ethanolamine kinase. putative
58183	+	+	+	+	+	+			MIF4G domain-containing protein (Initiation factor eIF-4 gamma. middle)
58184	+	+	+	+	+	+			DNA polymerase epsilon. subunit B
58188	+	+	+	+	+	+			Chitin synthase
58191	+	+	+	+	+	+			unknown protein
58201	+	+	+	+	+	+			mitochondrial NADH-ubiquinone oxidoreductase 20 kD subunit
58205	+	+	+	+	+	+			tyrosine protein kinase
58213									Ku80p (Hdf2p)
58220	+	+	+						MFS permease
58225									unknown protein
58227							2.248 up	4.000 up	GCN5-related acetyltransferase
58229	+	+	+	+	+	+	2.134 up	2.030 up	Ubiquitin-like protein (HubA)
58239									GH16 cell wall glucanosyltransferase
58244							1.892 down	4.551 down	unknown protein
58264							1.747 up	2.575 up	paxU orthologue ? (indole-terpene biosynthesis?)
58267	+	+	+	+	+	+	1.428 up	3.018 up	tRNA-dihydrouridine synthase.
58282	+	+	+						esterase family 9
58285							1.981 down	2.096 down	PKS-NRPS
58289									Zinc-binding oxidoreductase
58296							1.107 up	2.676 up	MFS permease
58299	+	+	+	+	+	+			RRB1 gene encoding an essential nuclear protein involved in early steps ofRibosome bioge
58321	+	+	+	+	+	+			ubiquitin-conjugating enzyme
58333							1.519 down	2.281 down	FAD binding protein
58356									glycerol kinase
58361	+	+	+						dual specificity phosphatase

58363									unknown protein
58366							1.280 down	2.944 up	AAA+-type ATPase
58370		+					1.865 down	2.339 down	mtDNA repair protein
58381	+	+	+	+	+	+			unknown protein
58387		+	+				1.261 down	2.312 down	proline iminopeptidase
58389	+	+	+						Zn2Cys6 transcriptional regulator
58391									unknown protein
58396									unknown protein
58404	+	+	+	+	+	+			unknown protein
58405	+	+	+	+	+	+			splicing factor U2AF 23 kDa subunit
58412							3.697 down	3.471 down	AdhP Zn-dependent alcohol dehydrogenases
58418									ß-lactamase-like protein
58421									FAD binding protein
58427	+	+	+	+	+	+			ATP-dependent helicase. DEAD-box
58428	+	+	+	+	+	+			Ribosomal biogenesis regulatory protein RRS1
58431							1.400 up	2.947 up	unknown protein
58442		+							unknown protein
58450							1.176 up	2.349 up	GH3 β-xylosidase XYL3b
58456							9.297 down	5.379 down	Zn2Cys6 transcriptional regulator
58466									alcohol dehydrogenase. putative
58472									Catalase
58475	+	+	+	+	+	+			unknown protein
58476	+	+	+	+	+	+			serine/threonine protein kinase
58479							1.418 down	3.365 down	short chain dehydrogenase/reductase
58489	+	+	+	+	+	+			unknown protein

58492	+	+	+	+	+	+	1.397 up	2.204 up	unknown protein
58493	+	+	+	+	+	+			Cytochrome c oxidase. subunit Vb
58505	+	+	+						cytochrome P450
58509							1.223 up	2.335 down	DNA ligase IV involved in non-homologous end joining of double-strand DNA breaks
58511	+	+	+				3.383 down	10.118 down	amino acid permease (GABA)
58519	+	+	+	+	+	+			unknown protein
58521							1.160 up	3.571 up	SAM (and some other nucleotide) binding motif
58525							2.797 down	3.337 down	unknown protein
58535							1.475 up	8.270 up	unknown protein
58539	+	+	+	+	+	+			Mitochondrial ribosomal protein of the small subunit. has similarity to mammalian apopto
58541									unknown protein with Kelch repeats
58549	+	+	+	+	+	+			cellular morphogenesis regulator DopA. putative
58550									glycan biosynthesis protein (PigL).
58560							1.242 down	2.103 down	MFS monocarboxylate transporter
58561	+	+							MFS permease
58563							1.004 up	2.070 up	oxalate decarboxylase
58574							1.092 up	2.601 up	unknown protein
58580									α-ketoglutarate dependent (FeII) oxygenase
58584			+						aquaglyceroporin
58587	+	+	+	+	+	+			Protein phosphatase 2C type Ptc1
58601							1.251 down	3.900 up	unknown protein
58602									unknown protein
58603			+				1.070 up	4.702 up	unknown protein. contains Splecktrin motif
58607	+	+	+	+	+	+			chromosome condensation protein (CrcB). putative
58609	+	+	+	+	+	+			GT α-1.6-mannosyltransferase
58612	+	+	+	+	+	+			ounknown proteinsterol binding protein

58613	+	+	+	+					Thiamine pyrophosphokinase
58627	+	+	+	+	+	+			ATP-dependent RNA helicase dbp9
58628	+	+	+	+	+	+			C2H2 transcription factor (Con7)
58631	+	+	+	+	+	+			Rad5p. a ubiquitin-protein ligase that functions in DNA damage tolerance
58634	+	+	+	+	+	+	1.499 down	3.290 down	Zn2Cys6 Fungal transcriptional regulator
58636	+	+	+	+	+	+			vacuolar fusion protein MON1
58638	+	+	+	+	+	+			vacuolar-sorting protein SNF8. putative
58639							10.563 down	2.584 down	unknown protein
58640	+	+	+	+	+	+	1.516 up	2.972 up	unknown protein
58651	+	+	+	+	+	+	1.084 down	2.597 up	adenylosuccinate synthase
58654									geranylgeranyltransferase beta subunit
58658	+	+							DNA helicase. putative
58669									aspartyl protease
58672									short chain dehydrogenase/reductase
58675							1.577 down	4.152 down	short chain dehydrogenase/reductase
58689							1.277 down	2.717 down	ERG5 C-22 sterol desaturase. a cytochrome P450 enzyme that catalyzes the formation of t
58694	+	+	+	+	+	+	1.700 down	2.232 down	unknown protein
58696									DEAD/DEAH box helicase
58698									subtilisin like protease (SUB2)
58699							1.682 down	2.440 down	unknown protein
58701									Mannose-6-phosphate isomerase
58717									ß-lactamase class C
58731									unknown protein
58734		+					1.390 up	3.106 up	prefoldin subunit 2

58746	+	+	+				1.343 down	2.596 down	unknown protein
58753		+	+						MmsB. 3-hydroxyisobutyrate dehydrogenase
58765									unknown protein
58767									unknown protein
58772			+						cytochrome P450 monooxygenase
58774									unknown protein
58783	+	+	+	+	+	+			alpha/beta hydrolase
58790									glycerol-3-phosphate phosphatase. putative
58802									GH95 α-L-fucosidase
58804									Rad9. that functions in DNARepair
58814									unknown protein with WD repeats
58815	+	+	+	+	+	+			alternative NADH-dehydrogenase. putative
58823							1.531 up	2.674 up	MRSP1/expansin-like
58837							1.566 down	2.698 down	unknown protein
58848							1.314 down	2.539 up	unknown protein
58851									Phosphatidylserine receptor protein. contains F-box and transcription factor jumonji
58853							2.977 up	19.052 up	Homeodomain-like
58856	+	+	+	+	+	+			phosducin like protein. class I
58857									unknown protein
58867									unknown protein
58869	+	+							unknown protein
58880							1.041 down	5.309 up	MRSP1/expansin-like
58885									Amidases
58887							1.134 down	2.799 up	GH78 α-L-rhamnosidase
58897	+	+	+	+	+	+	1.497 down	3.468 down	C2H2 transcriptional regulator

58899	+	+	+						MDR-type ABC transporters
58910									unknown protein
58918									Zn2Cys6 transcriptional regulator
58928	+	+	+	+	+	+			SNF2 family chromodomain-helicase DNA-binding protein
58952							1.547 down	2.103 down	calcium transporting ATPase. ion pump
58953									Cytochrome P450 CYP3/CYP5/CYP6/CYP9 subfamilies
58960	+	+	+	+	+	+			unknown protein
58971									RNA cap guanine-N2 methyltransferase. putative
58976	+	+	+	+	+	+			RRN3. required for the transcription of rDNA
58989									unknown protein
58990	+	+	+	+	+	+	1.491 up	2.112 up	v-SNARE Bos1. ER-Golgi
59002									unknown protein
59014									PDR-type ABC transporters
59023	+	+	+	+	+	+			Fe superoxide dismutase
59028			+				2.016 up	4.997 up	unknown protein
59042	+	+	+	+	+	+			unknown protein
59050							1.216 down	9.714 up	Ankyrin
59053	+	+	+	+	+	+	1.156 down	2.291 down	Adenosine/AMP deaminase
59056									unknown protein
59062	+	+	+	+	+	+			unknown protein
59065							1.339 up	2.752 up	unknown protein
59067	+	+	+	+	+	+	1.180 up	2.397 up	Zn2Cys6 transcriptional regulator
59068									thiamin biosynthesis protein thi-4 . putative
59070	+	+	+	+	+	+			peroxisome biosynthesis protein (PAS1/Peroxin-1).
59073							1.110 down	2.213 down	unknown protein
59078									unknown protein
59081							1.066 up	3.150 up	CsdB Selenocysteine lyase

59082									GH18. chitinaseCHI18-2
59095									Flavoprotein monooxygenase. putative
59098	+	+	+	+	+	+			unknown protein required for sexual development
59100	+	+	+	+					unknown protein
59105	+	+	+	+	+	+			unknown protein
59112	+	+	+	+	+	+			unknown protein
59119									amidohydrolase family protein
59137	+	+	+	+	+	+			unknown protein
59147	+	+	+						DNA lyase
59151							17.018 down	4.712 down	SSCRP
59152	+	+							unknown protein
59166	+	+	+	+	+	+			Mob1/phocein family protein
59168	+	+	+	+	+	+			Doa1. WD protein required for ubiquitin-mediated proteolysis
59173			+						MFS permease (A. fumigatus: "light regulation of gametogenesis protein 6")
59184	+	+							DEAD/DEAH box RNA helicase (Ski2). putative
59188							1.257 up	4.261 down	unknown protein
59190							1.614 down	2.775 down	MFS permease
59196							2.101 up	4.067 up	short chain dehydrogenase/reductase
59197									unknown protein. only in Gibberella and Chaetomium
59205									unknown protein
59232									AAA ATPase
59234	+	+					1.330 down	3.943 down	Zn2Cys6 transcriptional regulator
59244							1.079 up	4.037 up	metalloprotease. putative
59248		+							unknown protein
59267									pyruvate decarboxylase
59270	+	+	+	+	+	+			unknown protein

59272							1.314 down	3.044 down	MFS permease
59280	+	+	+	+	+	+			ARP23 complex 20 kDa subunit
59296									anaphase-promoting complex protein
59302	+	+	+	+	+	+			unknown protein
59315							1.723 up	2.386 down	PKS
59322							2.327 down	5.863 down	Zinc-binding oxidoreductase
59333							1.215 up	4.018 up	MFS permease
59338									D-Alanine aminotransferase
59346	+	+	+	+	+	+			chitin biosynthesis protein CHS5
59351							2.679 down	3.796 down	1-aminocyclopropane-1-carboxylate synthase
59352							1.659 down	3.656 down	cytochrome P450
59353							1.228 up	2.423 down	Zn2Cys6 transcriptional regulator
59354							2.000 down	3.047 down	Zn2Cys6 transcriptional regulator
59359	+	+	+	+	+	+			unknown protein
59362									unknown protein
59364							16.146 down	25.526 down	Sexual differentiation process protein ISP4
59368							5.742 up	33.950 up	unknown protein. Duf341
59372							21.869 down	3.935 down	unknown protein
59377									Cytochrome P450 CYP2 subfamily
59381							1.602 down	6.982 down	SAM-dependent methyltransferases
59382	+	+		+	+		1.643 down	9.290 down	unknown protein

59384									histidine kinase class I. M27Mp
59388									MFS permease
59391							1.035 down	4.898 down	GH27 α-galactosidase
59396							1.260 down	2.444 up	unknown protein
59402									arsenate reductase Arc2
59417		+							unknown protein
59434	+	+	+	+	+	+			unknown protein
59435	+	+	+	+	+	+			imh1. mediates transport between an endosomal compartment and the Golgi
59472	+	+	+	+	+	+			mitochondrial carrier protein
59482							2.690 up	5.271 up	PKS
59491							1.664 down	6.146 down	unknown protein
59508							1.443 down	2.313 up	dipeptidyl peptidase 5
59515	+	+	+	+	+	+	2.504 up	3.481 up	MFS permease
59542	+	+	+	+	+	+			Guanyl-nucleotide exchange factor Sec2
59544	+	+	+	+	+	+			SEN1 (tRNA splicing complex component).
59546	+	+	+				1.441 down	2.105 down	Zn2Cys6 transcriptional regulator
59549									beta-alanine synthase
59553									unknown protein
59558									unknown protein
59578									GH13 α-glucosidase
59579	+	+	+	+	+	+			unknown protein
59582							1.451 up	3.142 up	unknown protein
59584	+	+	+	+	+	+	1.644 up	2.685 up	unknown protein
59597							1.231 up	2.859 up	unknown protein
59598							1.883 down	4.278 down	unknown protein

59609	+	+	+	+	+	+	1.007 up	2.188 down	unknown protein
59624									short chain dehydrogenase/reductase
59625									unknown protein
59628							1.342 up	4.185 up	unknown protein
59642							1.211 down	3.427 down	α/ß hydrolase lipase/epoxide hydrolase
59649							1.129 up	3.892 down	Zinc-containing alcohol dehydrogenase
59655									unknown protein
59665				+	+	+	1.031 down	2.486 down	unknown protein
59668	+	+	+	+	+				DBF2. cell cyle protein kinase
59669							1.081 up	2.171 down	extracellular salicylate hydroxylase/monooxygenase. putative
59672									nitroreductase family protein
59673									acetyltransferase. GNAT family family
59689							1.219 down	2.438 down	GH2 β-mannosidase
59690							1.119 down	3.442 up	unknown protein
59697	+	+	+	+	+	+			unknown protein
59698									short chain dehydrogenase/reductase
59700									short chain dehydrognease/reductase
59705	+	+	+	+	+	+			unknown protein
59723									dipeptidyl peptidase 5
59726							1.062 up	5.297 up	DNA photolyase. N-terminal. class 1. FAD-binding
59740							1.298 down	3.502 down	transcriptional regulator. unknown
59746							1.158 down	3.233 down	oxaloacetase-like protein

59747				+	+	+	+	+	+	1.417 down	2.643 down	Rho GTPase-activating protein involved in signal transduction mechanisms
59751				+	+	+	+	+	+	1.475 down	2.093 down	Ribonucleases P/MRP protein subunit POP1 containing protein
59759		+										unknown protein
59760												Zn2Cys6 transcriptional regulator
59763										2.804 up	4.151 up	unknown protein
59768												unknown protein
59770										1.525 down	3.754 down	aryl-alcohol dehydrogenases
59771												UbiA prenyltransferase. putative
59775												unknown protein
59778					+	+				1.104 down	5.861 down	GPCR. related to A nidulans GprC
59791										1.143 up	4.978 up	GH18. chitinase CHI18-15
59796										1.407 down	2.193 down	MFS permease
59801	+	+	+							1.082 down	3.116 up	unknown protein
59814				+	+	+	+	+	+			transcriptional regulator. unknown
59827										1.592 up	4.546 up	unknown protein
59830												short-chain dehydrogenase/reductase
59833				+	+	+	+	+	+	2.185 down	2.973 down	unknown protein
59836				+	+	+	+	+	+			nuclear transport factor 2 domain-containing protein
59838				+		+						unknown protein
59843										1.113 up	2.512 down	AMP-dependent synthetase and ligase. acetoacetyl-CoA synthase-like
59846				+	+	+	+	+	+			lanosterol synthase
59862				+	+	+	+	+	+			t-SNARE Tlg1; fusion endosome-derived vesicles with late Golgi
59876										1.341 down	5.318 up	xenobiotic compound monoounknown proteingenase. DszA family

59887										5.385 down	4.318 down	L-lactate dehydrogenase. putative
59894												Phosphatidylinositol transfer protein
59900												unknown protein
59919				+	+	+	+	+	+			RNAse III domain protein
59936				+	+	+	+	+	+	1.599 up	2.570 up	unknown protein
59940				+	+	+	+		+	3.732 down	3.082 down	unknown protein
59944				+	+	+	+	+	+			calcineurin catalytic subunit
59951				+	+	+	+					CobW domain protein
59952										2.117 up	5.176 down	Amino acid transporter PotE
59958				+	+	+	+	+	+			unknown protein
59964				+	+	+	+	+	+			DNA replication factor A subunit Ssb3. putative
59987				+	+	+	+	+	+			Ubiquitin conjugating enzyme Ubc7
59988				+	+	+	+	+	+			ATP-dependent RNA helicase HAS1
59991				+	+	+	+	+	+			MAK16 protein. putative
59994												tRNA isopentenyltransferase.
60000				+	+	+	+	+	+			ubiquitin-conjugating enzyme
60002	+	+	+									
60004										1.338 down	2.648 down	Zn2Cys6 transcriptional regulator
60008				+	+	+	+	+	+			ribosomal protein MRPL35.
60018												AAA+ ATPase
60028										1.122 down	3.193 up	unknown protein
60033				+	+	+	+	+	+			short chain dehydrogenase/reductase
60051				+	+	+	+	+	+			GCN2. a kinase phosphorylating the alpha subunit of the translation initiation factor eIF2.
60052										1.895 down	4.237 down	short chain dehydrogenase/reductase
60067				+	+	+	+	+	+			L-threo-3-deoxy-hexulosonate aldolase

60085												GH31 α-glucosidase
60086												MFS permease
60089				+	+	+	+	+	+			succinate dehydrogenase cytochrome b subunit
60091												unknown protein
60095				+	+	+						unknown protein
60115				+	+	+	+	+	+			Rad9. DNA damage sensor.
60116				+	+	+	+		+	1.630 down	8.900 down	MRP-type ABC transporter
60118					+							PKS
60123				+	+	+	+	+	+			unknown protein
60132	+	+	+									C2H2 transcriptional regulator
60136												COPII coat assembly protein Sec16
60144										1.036 up	5.498 up	Amino acid transporters
60149												unknown protein DUF227
60162				+	+	+	+	+	+			anaphase-promoting complex subunit cut9
60163												Esterase/lipase/thioesterase domain; alpha/beta hydrolase. putative
60177												MFS permease
60182										1.995 down	2.394 up	unknown protein
60187										1.217 down	2.281 down	enoyl-CoA hydratase/isomerase
60194				+	+	+	+	+	+	1.588 down	2.163 down	phosphatidylinositol-specific phospholipase C (MPLC1)
60206												unknown protein
60215												Zn2Cys6 transcriptional regulator
60216				+	+	+						unknown protein
60222												SEC16. COPII coat assembly protein
60232										1.226 up	2.958 down	unknown protein
60243				+	+	+	+	+	+			medium subunit. apm1. of the adaptor protein complex AP-1 of clathrin-coated vesicles; i
60250				+	+	+	+	+	+			cysteinyl-tRNA synthetase. class Ia.

60255		+	+	+						tRNA-splicing endonuclease beta chain.
60270		+	+	+	+	+	+	1.362 down	2.202 down	unknown protein
60282								1.318 down	2.532 down	Zn2Cys6 transcriptional regulator
60283		+	+	+	+	+	+			unknown protein
60300		+	+	+	+	+	+			unknown protein
60319		+	+	+	+	+	+			unknown protein
60328								1.299 down	3.043 down	unknown protein
60329	+									MFS permease
60331		+	+	+	+	+	+			Small GTPase of the Rab/Ypt family. ypt7 and rab7 involved in vacuolar biogenesis
60337								7.097 down	2.932 down	unknown protein
60338		+	+	+	+	+	+			SAM methyltransferase. tRNA-methylztransferase subunit GCD14 (yeast).
60345		+	+	+	+	+	+			unknown protein
60346		+	+	+	+	+	+			Para-aminobenzoate (PABA) synthase PabaA
60349		+	+	+	+	+	+			Exocyst component Sec8
60352								3.078 down	4.788 down	NADH-dehydrogenase (ubiquinone)
60370								5.514 down	3.175 down	unknown protein
60374										choline oxidase
60378								2.670 up	2.164 down	AAA ATPase
60392		+	+	+	+	+	+			Mitochondrial import inner membrane translocase. subunit Tim17/22
60402										tRNA modification GTPase TrmE. putative
60406		+	+	+	+	+	+			mitochondrial matrix iron-sulfur protein
60418								1.458 up	2.832 up	Aldehyde dehydrogenase
60419								1.005 down	2.848 up	acid phosphatase. putative

60422							2.764 down	2.876 down	unknown protein
60425		+							Mechanosensitive ion channel family protein
60426	+	+	+	+	+	+			unknown protein
60445							3.424 down	9.081 down	unknown protein
60450	+	+	+	+	+	+			Hus1 protein of Schizosaccharomyces pombe and mammals. a component of the 9-1-1 rep
60456							1.149 up	2.137 down	unknown protein
60458									NRPS
60467									MFS monocarboxylate transporter. putative
60482		+							unknown protein
60487									unknown protein
60489							2.666 down	2.296 up	CE5 cutinase
60490							1.104 up	2.155 down	2-nitropropane dioxygenase
60492									unknown protein
60493	+	+	+						ribosomal RNA methyltransferase RrmJ/FtsJ domain.
60498	+	+	+	+	+	+			unknown protein
60508									unknown protein
60517									short chain dehydrogenase/reductase
60518									short chain dehydrogenase/reductase
60551	+	+	+	+	+	+			PotE Amino acid transporters
60557							1.123 up	2.760 up	unknown protein
60558									Zn2Cys6 transcriptional regulator
60560							31.933 down	2.174 down	unknown protein
60562									unknown protein
60564									mitochondrial mRNA processing protein PET127. putative

60565							1.012 up	4.780 down	unknown protein
60578	+	+	+	+	+	+	1.131 down	2.268 down	Zn2Cys6 transcriptional regulator
60581									acetylornithine deacetylase
60585									C2H2 transcriptional regulator
60591	+	+	+	+	+	+			unknown protein
60608									unknown protein
60616	+	+	+	+		+	4.708 down	3.985 down	unknown protein
60626	+	+	+	+	+	+			Vesicle coat complex COPII. Sec24
60627									Zn2Cys6 transcriptional regulator
60634	+	+	+	+	+	+	1.156 up	2.372 down	transcriptional regulator. unknown
60635							2.154 down	25.709 up	GH92 α-1.2-mannosidase
60638							2.099 down	5.875 up	unknown protein
60664	+	+	+	+	+	+	1.239 down	2.244 down	unknown protein with PH domain (putative phosphatidylinositol 4.5-bisphosphate protei
60665									Zn2Cys6 transcriptional regulator
60671		+							metallo-beta-lactamase domain protein. putative
60676	+	+	+	+	+	+			cysteine protease. mammalian caspases
60698	+	+	+	+	+	+			unknown protein
60729	+	+	+	+	+	+			unknown protein
60737	+	+	+	+		+			unknown protein
60739									short-chain dehydrogenase/reductase
60743	+	+	+	+	+	+	1.540 up	2.098 up	Dolichyl-phosphate mannosyltransferase polypeptide 3
60751									NRPS
60752									dipeptidyl peptidase 5
60756									unknown protein

60758		+		+	+		+	12.719 down	5.079 down	SAM-dependent methyltransferase
60761		+	+	+	+	+	+	2.016 down	3.554 down	transcriptional regulator Grainyhead/CP2
60768			+							unknown protein
60769		+	+	+						Exonuclease
60771		+	+	+	+	+	+	1.632 up	2.232 up	prefoldin chaperone
60773			+							ankyrin
60779		+	+	+	+	+	+			unknown protein
60780		+	+	+	+	+	+			unknown protein
60784										FAD-binding protein
60791										subtilisin like protease
60793		+	+	+	+	+	+			unknown protein
60796		+	+	+	+	+	+			mitochondrial ribosomal protein subunit S4
60810								12.659 down	3.051 down	unknown protein. GPR1/FUN34/yaaH protein. 6TMs
60814										glutamyl-tRNA amidotransferase subunit B in other fungi.
60815		+	+	+	+	+	+			CRAL/TRIO domain-containing protein
60825								1.099 up	4.054 up	Cytochrome P450. putative
60835								1.105 down	2.255 down	PutA delta-1-pyrroline-5-carboxylate dehydrogenase
60847		+	+	+	+		+	1.334 down	2.048 down	Mitochondrial F1F0-ATP synthase. subunit c/ATP9/proteolipid
60849		+	+	+	+		+			FAD-dependent sulfhydryl oxidase Erv1
60850		+	+	+			+	1.142 down	2.173 down	Mitochondrial initiation factor 2 (IF-2).
60855										tRNA (guanine-N(1)-)-methyltransferase. putative
60867	+	+	+	+	+	+	+			unknown protein
60873		+	+	+						ATP-dependent DNA ligase
60879		+	+	+	+	+	+			unknown protein
60887		+	+	+		+				unknown protein

60889									MFS permease
60890	+	+	+	+	+	+	1.224 up	3.235 up	L-galactose dehydrogenase (L-GalDH). putative
60897							1.013 down	2.589 down	Zn2Cys6 transcriptional regulator
60928	+	+	+	+	+	+			GTPase involved in G-protein signaling in the adenylate cyclase activating pathway
60931							1.335 up	2.042 down	Zn2Cys6 transcriptional regulator
60945			+				4.781 down	2.081 up	MFS permease
60949							1.906 up	2.613 down	unknown protein
60951	+	+	+	+	+	+	1.275 down	2.289 down	unknown protein
60956	+	+	+	+	+	+			unknown protein
60981							1.043 down	2.651 down	unknown protein
60987							1.100 up	3.317 down	MRP-type ABC transporter
60988									phosphate transporter
61000	+	+	+	+	+	+			cleavage and polyadenylylation specificity factor. putative
61020		+	+	+		+	1.740 up	2.330 up	thioesterase superfamily protein
61032							1.363 down	2.274 down	unknown protein
61039	+	+	+	+	+	+			Rad27. DNA structure-specific endonucleases involved in Okazaki fragment maturation
61042	+	+	+	+	+	+			MMR; inhibition of homeologous recombination
61043	+	+	+	+	+	+			unknown protein
61055							1.851 down	3.390 up	unique protein
61066							1.434 down	2.615 down	GMC oxidoreductase
61074									MRP-type ABC transporter
61075									Rad4p. involved in nucleotide excisionRepair.

61076									unknown protein
61078	+	+	+	+		+			esterase/ lipase
61081									histone acetyltransferase SPT10
61097									unknown protein
61103	+	+	+	+	+	+			Signal recognition particle. subunit Srp14
61114									Amino acid transporter LysP
61116		+					1.039 up	2.148 down	ferric reductase
61121							1.160 down	2.413 down	arylsulfatase. putative
61122	+	+	+	+	+	+			unknown protein
61127	+	+	+	+		+	1.975 down	3.274 down	Serine carboxypeptidase
61134	+	+	+	+	+	+	1.429 up	2.205 up	unknown protein
61142									unknown protein
61153	+	+	+	+	+	+			uroporphyrinogen synthase
61159	+	+	+	+	+	+			Component of oligomeric golgi complex Cog3/Sec34
61161	+	+	+	+	+	+			Peptidase S54. rhomboid
61164									unknown protein
61183									Polynucleotide 5'-hydroxyl-kinase GRC3. putative
61189	+	+	+	+	+	+			tubulin-specific chaperone c
61190	+	+	+	+	+	+	1.766 up	2.218 up	BolA domain-containing protein
61208	+	+	+	+	+	+			unique protein
61212	+	+	+	+	+	+			cytochrome P450 monooxygenase
61219	+	+	+	+	+	+			xanthine phosphoribosyltransferase 1
61222							1.149 down	3.238 down	unknown protein
61223			+						unknown protein
61227									cobalamin synthesis protein
61229	+	+	+	+	+	+			unknown protein
61239									unknown protein

61258									Peptidase C12. ubiquitin carboxyl-terminal hydrolase 1
61278							1.175 down	2.137 down	MFS permease
61279	+	+	+	+	+	+			UTP22. U3 snoRNP protein involved in maturation of pre-18S rRNA and SSU processome p
61284	+	+	+	+	+	+			methyltransferase
61288									unknown protein
61292	+	+	+	+	+	+			unknown protein
61293							1.030 down	2.666 down	dipeptidyl peptidase 5
61298	+	+	+	+					unknown protein
61304									Prolidase and Aminopeptidase P
61307	+	+	+	+	+	+			unknown protein
61322	+	+	+	+	+	+			poly(A) polymerase with similarity to the DNA polymerase sigma family
61350									MFS permease
61354									unknown protein
61367	+	+	+	+	+	+			fatty acid desaturase
61374									MFS permease
61382	+	+	+	+	+	+			Integral membrane protein Sys1
61383				+			1.849 down	2.716 down	unknown protein
61402	+	+	+	+	+	+			unknown protein
61403									Hydantoinase/oxoprolinase
61408	+	+	+	+	+	+			RAS-GTPase activating protein (RasGAP)
61409	+	+	+	+	+	+			unknown protein
61420	+		+			+			unknown protein
61441	+	+	+		+	+	1.542 down	2.246 down	unknown protein
61442	+	+	+	+	+	+			enoyl-CoA hydratase/isomerase
61462									unknown protein
61465	+	+	+	+	+	+			SNARE complex subunit Vam7
61470	+	+	+	+	+	+			Sar1 GTPase

61471				+	+	+	+	+	+			unknown protein
61476										1.409 down	3.821 down	Zn2Cys6 transcriptional regulator
61486					+							GPI ethanolamine phosphate transferase
61496				+	+	+				1.036 down	2.757 up	MFS permease
61503				+	+	+	+	+	+			unknown protein
61504										2.093 down	3.731 down	unknown protein
61508				+	+	+	+	+	+			chromatin assembly protein. putative
61517				+	+	+	+	+	+			unknown protein
61523				+	+	+	+	+	+			KTI11 gene that encodes a protein involved in diphthamide synthesis.
61526										2.413 down	3.380 down	unknown protein
61530				+	+	+	+	+	+			unknown protein
61532				+	+	+						mRNA binding protein Pumilio 2. putative
61536												aspartyl protease
61541				+	+	+	+	+	+			chromatin remodelling subunit ARP8
61544				+	+	+	+	+	+			Rfc3p of Saccharomyces cerevisiae.
61550				+	+	+	+	+	+			DEAD/DEAH box helicase
61551				+	+	+	+	+	+			DNA polymeraseDelta. subunit 4
61553										5.669 down	4.651 down	D-xylulose 5-phosphate/D-fructose 6-phosphate phosphoketolase
61556				+	+	+	+	+	+			unknown protein
61567				+	+	+	+	+	+			SSCRP
61570				+	+	+						Gß-WD40 protein Ski8 (Sordaria)
61576	+	+	+			+						unknown protein
61577				+	+	+	+	+	+			unknown protein
61584				+	+	+	+	+	+			unknown protein
61588				+	+	+	+	+	+			unknown protein
61593	+	+	+	+	+	+	+	+	+			unknown protein

61595	+	+	+	+	+	+			unknown protein
61605							9.729 up	2.443 up	Vacuolar sorting protein VPS1. dynamin. and related proteins
61618							1.187 up	3.255 up	unknown protein
61632							2.128 up	2.019 up	unknown protein
61642	+	+	+				1.927 down	3.207 down	unknown protein. secreted
61675	+	+							unknown protein
61677	+	+	+	+	+	+			sphingosine-1-phosphate phosphohydrolase
61690		+							short chain dehydrogenase/reductase
61692	+	+	+	+	+	+			Chromosome condensation protein 3. C-terminal
61701	+	+	+	+	+	+			Metal-dependent hydrolases belonging to the beta-lactamase superfamily.
61703	+	+	+	+		+	1.316 up	2.241 up	germinal center kinase. related to S. cerevisiae Kic1
61705	+	+							sterol o-acyltransferase
61715	+	+	+	+	+	+			unknown protein
61720	+	+	+	+	+	+			histidine kinase class X. HHK1
61746									phospholipase C. related to Cryphonectria parasitica phosphatidyl inositol-specific phosph
61750	+	+	+	+	+	+			Sur2 sphinganine C4-hydroxylase of Saccharomyces cerevisiae
61763			+						unknown protein
61765		+							Peptidase D
61771									unknown protein
61794	+	+	+	+	+	+			nuclease. mitochondrial
61798	+	+	+				1.072 down	2.271 up	unknown protein. only in ascomycota
61804	+	+	+	+	+	+			ribosomal protein YmL32 precursor. Contains possible InterPro domain for ribosomal L32p
61815	+	+	+	+	+	+			RNA polymerase Rpb6
61819									unknown protein
61830							1.405 down	2.945 up	SSCRP
61839									unknown protein
61858							1.334 down	2.801 up	unknown protein

61863					+	+						unknown protein
61874				+	+	+	+	+	+			Mg2+ transporter protein. CorA-like
61885				+	+	+	+	+	+			unknown protein
61893				+	+	+						2.3-diketo-5-methylthio-1-phosphopentane phosphatase
61907				+	+	+	+	+	+	1.002 up	2.049 down	unknown protein
61910					+					1.396 up	2.016 down	Aldehyde dehydrogenase
61912				+	+	+	+	+	+			Vacuolar carboxypeptidase Cps1
61924				+	+	+	+	+	+			gelsolin
61939										1.044 down	3.007 up	unknown protein
61945				+	+	+	+	+	+			unknown protein
61946				+	+	+						tyrosyl-DNA phosphodiesterase. putative
61948										1.383 down	3.258 up	Protein disulfide isomerase 2
61965				+	+	+	+	+	+			unknown protein
61967				+	+	+	+	+	+			unknown protein
61971				+	+	+	+	+	+			TRAPP complex component Trs33
61987										1.122 down	2.063 down	unknown protein
61988				+	+	+	+	+	+			unknown protein
61995	+	+	+	+	+	+	+	+	+	1.071 up	2.258 up	unknown protein
62000				+	+	+	+	+	+			twinfilin-1
62003				+	+	+	+	+	+			signalosome subunit 5 (CsnE) putatively involved in regulation of sexual development
62004				+	+	+	+	+	+			unknown protein
62017				+	+	+	+	+	+			unknown protein
62026				+	+	+	+	+	+			mediator of RNA polymerase II transcription subunit 10. putative
62029				+	+	+	+	+	+			pre-mRNA-splicing factor 18
62034										1.683 down	4.546 down	unknown protein

62040	+	+	+	+	+	+			ATPase Sec18. required for ER to Golgi transport etc
62041									unknown protein
62053									unknown protein
62057	+	+	+	+	+	+			DNA repair protein Rhp26/Rad26
62059									unknown protein
62063	+	+	+	+	+	+			U1 small nuclear ribonucleoprotein
62071		+							histone acetylase complex subunit
62078									unknown protein
62086	+	+	+	+	+	+	1.555 up	2.551 up	unknown protein
62091									Gluconate kinase
62100	+	+				+			Hsp30
62103	+		+	+					unknown protein
62114							1.978 down	3.675 down	unknown protein
62120	+	+	+	+	+	+			importin ß KapK
62121									HET-E-1 / beta transducin-like protein HET-E2C*4
62130	+	+	+						unknown protein
62153							1.503 down	2.219 down	malate dehydrogenase
62165									catechol dioxigenase
62166									GH2 β-mannosidase
62168	+	+	+	+	+	+			palmitoyltransferase
62169	+	+	+	+	+	+			protein kinase
62171		+	+				1.142 down	5.770 up	MFS permease
62172									amino acid permease (PotE?)
62181	+	+	+	+	+	+	1.073 down	2.658 down	Protein kinase. Ca2+-dependent
62182	+	+	+	+	+	+			UDP-glucose:sterol b-glucosyltransferase
62198									Cytosine-purine permease
62199		+							Zn2Cys6 transcriptional regulator

62213	+	+	+	+		+	1.021 down	3.707 down	unknown protein with fasciclin domain
62219	+	+	+	+	+	+			NADH-quinone oxidoreductase
62225	+	+	+	+	+	+			unknown protein
62226	+		+			+			DNA repair helicase family protein
62230									esterase/lipase
62231									Cytochrome P450 CYP3/CYP5/CYP6/CYP9 subfamilies
62232	+	+	+	+	+	+			unknown protein
62244									Zn2Cys6 transcriptional regulator
62254	+	+	+	+	+	+			Secretion related small GTPase Rab4
62255							1.092 up	3.526 up	lipase. lipocalin related
62263									Cytochrome P450 CYP2 subfamily
62271									Acetyltransf_Acetyltransferase (GNAT) family
62285							1.215 down	3.054 down	unknown protein
62297	+	+	+	+	+	+			Zn2Cys6 transcriptional regulator
62299	+	+	+	+	+	+			unknown protein
62300									unknown protein
62301	+	+	+	+	+	+			MMR; HR regulation
62305	+	+	+	+	+	+			UDP-glucose glycoprotein a-glucosyltransferase
62314									unknown protein
62323							1.296 up	2.216 down	unknown protein
62333							1.003 up	3.830 up	unknown protein
62335	+	+	+	+	+	+			methionine aminopeptidase
62340	+	+	+	+	+	+	2.892 up	2.669 up	unknown protein
62345	+	+	+	+	+	+			translation initiation factor ?
62351	+	+	+	+	+	+			unknown protein
62352	+	+	+	+	+	+			unknown protein
62359									isoflavon reductase

62362	+	+	+	+	+	+			calcium transporting ATPase. ion pump
62367	+	+	+	+	+	+			Sulfite oxidase. molybdopterin-binding component
62377	+	+	+	+	+	+			GT64 a-N-acetylhexosaminyltransferases
62380							18.482 down	2.628 down	MFS permease (galactose permease ?)
62386									Zn2Cys6 transcriptional regulator
62389	+	+	+	+	+	+			NADH dehydrogenase (ubiquinone) 1 alpha subcomplex. 13. cell death-regulatory protein
62401							1.136 down	2.453 down	guanine nucleotide exchange factor synembryn. putative
62424							1.165 down	3.248 down	UDP-glucose:sterol b-glucosyltransferase
62426	+	+	+	+	+	+			GTPase activator protein for Ras-likeGTPase (RasGAP)
62432	+	+	+	+	+	+			Zn2Cys6 transcriptional regulator
62439									short chain dehydrogenase/reductase
62447							1.686 down	4.938 down	unknown protein
62462	+	+	+	+	+	+	1.475 down	2.438 up	PTH11 GPCR
62463		+							FAD binding domain containing protein
62467	+	+	+	+	+	+			protein required for sporulation
62470	+	+	+	+	+	+			mitochondrial elongation factor G.
62475									glutathione-dependent formaldehyde-activating
62477	+	+	+	+	+	+	2.257 up	2.568 up	Vacuolar protein sorting-associated protein Vps28
62480	+	+	+	+	+	+	1.686 up	2.049 up	mitochondrial tricarboxylate transporter (Ctp). putative
62484							2.877 down	3.937 down	unknown protein
62488							3.022 up	2.780 up	MFS permease
62493	+	+	+	+	+	+			chromatin structure-remodeling complex protein RSC1. putative
62502	+		+				1.270 up	2.522 down	MFS permease
62522									SSCRP

62535			+	+	+						unknown protein
62537			+	+	+	+	+	+			unknown protein
62548											unknown protein
62556			+	+	+	+	+	+	2.118 down	3.153 down	unknown protein
62563			+	+	+	+	+	+			phosphatidylinositol-glycan biosynthesis class S protein
62576			+		+						alpha/beta hydrolase
62577			+	+	+	+	+	+			unknown protein
62581											gluconolactonase-like protein
62583			+	+	+	+	+	+			phosphate phosphoenolpyruvate translocator protein
62603											unknown protein
62611			+	+	+	+	+	+	1.888 down	8.764 down	MRP-type ABC transporter
62613			+	+	+	+	+	+			meiotically up-regulated protein
62622											Zn2Cys6 transcriptional regulator
62633											unknown protein
62634											anaphase promoting complex subunit APC11
62643											SAM-dependent methyltransferases
62645			+	+	+		+				GH18. chitinase CHI18-4
62651									1.323 down	5.377 down	long-chain fatty acid transporter.
62658			+	+	+	+	+	+			unknown protein
62663			+	+	+	+	+	+	1.221 up	2.548 up	unknown protein
62676			+	+	+	+	+	+			unknown protein
62685	+	+	+	+	+	+	+	+			Component of oligomeric golgi complex Cog6/Sec37
62693				+					1.031 up	18.111 down	ABC-transporter Ste6p
62702			+	+	+						unknown protein
62703				+	+						unknown protein
62704									1.191 down	2.177 down	GH18. chitinase CHI18-3

62706	+	+	+	+	+	+			protein phosphatase
62709	+	+	+	+	+	+			unknown protein
62711	+	+	+	+	+	+	1.252 down	2.050 down	amino acid transporter
62716							2.434 up	515.554 up	Ctr copper transporter family protein
62718	+	+	+	+	+	+			ubiquitin-conjugating enzyme
62720	+	+	+	+	+	+			unknown protein
62721	+	+	+	+	+	+			ribosomal protein S8. MRPS8.
62747			+						MFS permease
62751	+	+	+	+	+	+			histidine kinase HHK6
62765									unknown protein with NUDIX domain (hydrolase)
62769	+	+	+	+	+	+			DPH2. required for diphthamide synthesis.
62780	+	+	+						SAM methyltransferase. TRM12
62787	+	+	+	+	+	+			Zn2Cys6 transcriptional regulator
62789	+	+	+	+	+	+			unknown protein
62805							1.427 up	2.758 down	C2H2 transcriptional regulator
62809	+	+	+	+	+	+			Unknown protein with WD40 repeats
62820	+	+	+	+	+	+			Ribosomal protein(60S) L152/L15B
62821	+	+	+	+	+	+			unknown protein
62826									unknown protein
62835	+	+	+	+	+	+			Calcium transporter
62836	+	+	+	+	+	+	1.377 down	2.777 down	unknown protein
62871									unknown protein
62872							2.024 up	9.608 down	unknown protein GPR1/FUN34/yaaH-like
62880	+	+	+	+	+	+			unknown protein
62886		+							unknown protein
62912	+	+	+	+	+	+	1.810 up	2.580 up	unknown protein. 1TM

62914	+	+	+	+					Rpp14 family protein
62922	+	+	+	+	+	+			unknown protein
62929	+	+	+	+	+	+			unknown protein
62938									unknown protein
62962	+	+	+	+	+	+			unknown protein
62964	+	+	+	+	+	+			transcriptional regulator HMG type
62971									MFS permease
62975	+	+	+	+	+	+			unknown protein
62977							2.778 down	3.175 down	nucleoside diphosphate sugar epimerase. secreted
62979									unknown protein
62985	+	+	+						lipase/esterase
62986	+	+	+	+	+	+			unknown protein
63001									pyridoxamine phosphate oxidase family protein
63006									inositol monophosphatase
63007	+	+	+				1.582 down	3.793 down	meiotic chromosome segregation protein. putative
63011	+	+	+						unknown protein
63038	+	+	+	+	+	+			Rpc82. DNA-directedRNA polymerase III subunit; In S. cerevisiae. the enzyme is composed
63047	+	+	+						MMR; HR regulation
63053	+	+	+	+	+	+			Secretion related small GTPase Rab2
63088	+	+	+			+			unknown protein
63092	+	+	+	+	+	+			ribitol kinase
63093	+	+	+						unknown protein
63102	+	+	+	+	+	+			ARP2/3 complex. 21 kDa p21-Arc subunit
63104									unknown protein
63107									unknown protein
63125			+		+				unknown protein
63145	+	+	+	+	+	+			MFS permease
63151									unknown protein

63152							1.122 down	2.397 down	Cut9 interacting protein Scn1. putative
63156	+	+	+	+	+	+			unknown protein
63157		+				+			unknown protein
63159	+	+	+	+	+	+			straitin Pro11 (stalk rot protein)
63163							1.015 up	2.105 down	unknown protein
63173	+	+	+	+	+	+			unknown protein with YIP1 domain
63180	+	+	+	+	+	+			acetylornithine aminotransferase-like protein
63200									ku70
63202							1.213 down	4.578 down	alpha/beta hydrolase.
63204							1.214 down	2.179 down	flavodoxin domain containing protein
63206	+	+	+	+	+	+			ZIP Zinc transporter
63217	+	+	+		+				unknown protein
63240									HET-E-1. putative
63257									unknown protein
63269	+	+	+	+	+	+			mitochondrial ribosomal protein MRPL3.
63272	+	+	+	+	+	+			NifU-like protein
63277		+							unknown protein
63290									unknown protein
63293	+	+	+	+	+	+			unknown protein
63328	+	+							unknown protein
63341	+	+	+						Phosphatidylinositol:UDP-GlcNAc a-GlcNAc-transferase (Pig-A)
63347	+	+	+	+	+	+			Phosphatidylinositol-4-kinase stt4
63352	+	+	+	+	+	+			unknown protein
63354	+	+	+	+	+	+			phosphoadenosine phosphosulfate reductase
63364									unknown protein
63378									unknown protein
63379	+	+	+	+	+	+	2.029 up	2.439 up	RNA polymerase II transcriptional coactivator. putative

63381									unknown protein
63382	+	+	+	+	+	+			unknown protein
63395							1.427 up	2.347 down	Glycerophosphoryl diester phosphodiesterase
63397	+	+	+		+	+			unknown protein
63398	+	+							Peptidase M48. Ste24p
63400							1.303 down	2.106 down	mitochondrial exoribonuclease Cyt-4
63413			+						unknown protein. only in ascomycota
63416									unknown protein
63419									Met-10+ like-protein
63435									unknown protein
63441	+	+	+	+	+	+			mitochondrial precursor proteins import receptor
63442	+	+	+	+	+	+			SWR1-complex protein 4. putative
63454	+	+	+	+	+	+			DNA replication licensing factor mcm2
63464		+	+						unknown protein
63465	+	+	+	+	+	+			unknown protein
63484	+	+							chromodomain-helicase-DNA-binding protein
63503							1.398 up	2.238 up	rrbosomal protein S2 and tRNA/rRNA methyltransferase.
63507	+	+	+	+	+	+			Di-trans.poly-cis-decaprenylcistransferase. putative
63511	+	+	+	+	+	+			unknown protein
63526							2.301 up	6.318 up	SSCRP
63532	+	+	+	+	+	+			Zn2Cys6 transcriptional regulator
63542	+	+	+	+	+	+			unknown protein
63547	+	+	+	+	+	+			DNA-directed RNA polymerase II subunit RPB7
63558	+	+	+						unknown protein
63568									SNF2-like helicase. ATPase domain
63620	+	+	+	+	+	+			unknown protein
63624	+	+	+	+	+	+			splicing factor 3A subunit 2
63632	+	+	+	+	+	+			unknown protein

63648		+							unknown protein
63653							9.788 up	5.352 up	ankyrin
63657									unknown protein
63678	+	+	+	+	+	+			unknown protein
63687							4.207 down	2.154 down	ankyrin
63692							1.343 down	2.729 down	dipeptidyl peptidase 5
63702	+	+	+	+	+	+	1.342 down	2.367 down	unknown protein
63703									molybdopterin synthase small subunit CnxG. putative
63709	+	+	+	+	+	+			cystathionine beta-synthase (beta-thionase). putative
63710							1.246 down	2.107 up	MMR; HR regulation
63717	+	+	+	+	+	+			unknown protein
63727							1.004 down	2.219 down	Acyl-CoA dehydrogenase. central region
63733			+						unknown protein
63742	+	+	+	+	+	+			Demethoxyubiquinone hydroxylase
63744	+	+	+	+	+	+			unknown protein
63746	+	+	+	+	+	+			unknown protein
63747	+	+	+	+	+	+			unknown protein with PWWP domain
63751	+	+	+	+	+	+			19S regulatory particle ATPase Rpt4
63754	+	+	+	+	+	+			t-SNARE. SSO1
63756							2.781 down	2.539 down	helicase. DEAD-box superfamily
63772	+	+	+	+	+	+			chromatin structure-remodeling complex protein. putative
63774	+	+	+	+	+	+			GTPase. related to S. cerevisiae NuclearGTP-binding protein NUG1 (NuclearGTPase 1)
63780	+	+	+	+	+	+			mitochondrial inner import translocase TIM54
63813	+	+	+	+	+	+			cytochrome b5. putative
63815	+	+	+	+	+	+	1.051 up	4.989 up	Membrane coat complex Retromer. subunit Vps26

63828			+						GT α-1.6-mannosyltransferase
63837							1.072 down	2.580 down	unknown protein
63868							2.728 down	2.899 down	unknown protein
63869		+	+						unknown protein
63881		+					2.236 up	2.218 up	unknown protein
63882	+	+	+	+	+	+			enoyl-CoA hydratase
63889	+	+	+						Adenosine deaminase-related growth factors
63899	+	+	+	+	+	+	1.554 up	4.639 up	unknown protein
63909									unknown protein
63913									unknown protein
63914	+	+	+	+	+	+	1.761 down	3.150 down	integral membrane protein. putative
63919	+	+	+	+	+	+	2.195 up	2.727 up	Cys/Met metabolism PLP-dependent enzyme
63935	+	+	+	+	+	+			isoleucyl-tRNA synthetase. class Ia.
63937	+	+	+	+	+	+			mip1/kog1p. putative guanine nucleotide binding protein
63946	+	+	+	+	+	+			GPI transamidase subunit PIG-U
63952	+	+	+	+	+	+			unknown protein
63955	+	+	+						unknown protein
63966							1.027 down	3.494 up	high-affinity glucose transporter
63967									GPI-anchored wall transfer protein 1. putative
63972									cytochrome c oxidase assembly protein (Pet117). putative
63978							1.133 down	2.490 down	Zn2Cys6 transcriptional regulator
63981									GPCR . contains RGS domain
64000	+	+	+	+	+	+			unknown protein
64009	+	+	+	+	+	+			importin ß KapH
64010	+	+	+						unknown protein
64011	+	+	+	+	+	+			unknown protein

64018		+					1.107 up	3.214 down	GPCR. mating type pheromone G-protein coupled receptor
64023	+	+	+	+	+	+			ERAD-associated E3 ubiquitin-protein ligase component HRD3
64029							1.233 down	2.447 down	folylpolyglutamate synthase . putative
64044			+						unknown protein
64049							1.064 down	7.850 up	unknown protein
64065	+	+	+	+	+				tRNA(His) guanylyltransferase . putative
64066							1.196 up	2.742 down	acyltransferase 3
64110	+	+	+	+	+				unknown protein
64111	+	+	+	+	+	+			structural maintenance of chromosomes 5 smc5
64112	+	+	+	+	+	+			unknown protein
64117	+	+	+	+	+	+			uracil-DNA glycosylase
64125									Serine/threonine protein kinase
64130	+	+	+	+	+	+			unknown protein
64138	+	+	+	+	+	+			SET1 complex component swd1
64144									cyclophilin type peptidyl-prolyl cis-trans isomerase/CLD
64167		+							Sexual differentiation process protein ISP4
64172							1.590 up	2.704 up	Glutathione-dependent formaldehyde-activating.GFA
64175									transcriptional regulator HMG type
64179	+	+	+	+	+	+			unknown protein
64181							1.885 up	2.427 up	SSCRP
64193	+	+	+	+	+	+			glutamate carbounknown proteinpeptidase
64196	+	+	+	+	+	+			serine/threonine protein kinase
64212	+	+	+	+	+	+			unknown protein
64249		+							septum formation protein Maf
64261	+	+	+	+	+	+			subtilisin like protease
64271	+	+	+	+	+	+			unknown protein
64272	+	+	+	+	+	+			unknown protein

64274									unknown protein
64285	+	+	+	+	+	+			GH47 α-mannosidase
64288							1.188 down	2.399 up	unknown protein
64295							1.242 down	2.735 down	nitrogen permease regulator Npr2. putative
64298	+	+	+	+	+	+			kinesin-like protein
64300	+	+	+	+	+	+			cell polarity protein (Tea1)
64308	+	+							unknown protein
64312							1.953 down	4.165 down	unknown protein. only present in fungi
64314									MFS permease
64318	+	+	+	+	+	+	1.295 up	2.086 up	unknown protein
64322									unknown protein
64327	+	+	+	+	+	+			threonyl-tRNA synthetase. class IIa.
64330									indoleamine 2.3-dioxygenase
64332	+	+	+	+	+	+			TRAPP complex component Bet3
64345	+	+	+	+	+	+			3-Methylcrotonyl-CoA carboxylase. non-biotin containing subunit/Acetyl-CoA carboxylase
64347	+	+	+	+	+	+			unknown protein
64358									unknown protein
64370							2.259 down	8.211 down	calpain-like protease
64372			+						unknown protein
64375	+	+	+	+	+	+			GH5 glucan β-1.3-glucosidase
64377									Cytochrome P only
64385									Riboflavin kinase / FAD synthetase
64392	+	+	+	+	+	+			unknown protein with Kelch domain
64397									Ceramidase family protein. associated toCellulase signal transduction (PMID: 15288024)
64420	+	+	+	+	+	+			unknown protein
64421	+	+	+	+	+	+			unknown protein
64437	+	+	+	+	+	+			BRCT domain-containing protein

64448	+	+	+				1.720 up	2.858 up	unknown protein
64469							1.902 down	2.170 down	Hydrolases of alpha/beta hydrolase superfamily
64485									unknown protein
64498	+	+	+	+	+	+			Xrs2. Interacts with Tel1. enhances Mre11 nuclease activity
64543									GT50 GPI mannosyltransferase 1
64545							2.286 down	3.702 down	MFS permease
64561	+	+	+	+	+	+			cytochrome b5. putative
64600	+	+	+	+	+	+			Oligosaccharyltransferase. delta subunit
64601	+	+	+						unknown protein
64608	+	+	+	+	+	+			Regulatory protein involved in catabolite repression. part of a complex with creB that regu
64617									Guanine nucleotide-binding protein beta subunit-like protein. contains WD40-repeats
64620							1.383 down	2.236 down	Alpha/beta hydrolase
64625									unknown protein
64642	+	+	+	+	+	+			Rad1/Rec1/Rad17 repair protein
64644	+	+	+	+	+	+			unknown protein
64654	+	+	+	+	+	+			Vacuolar protein sorting-associated protein Vps8
64656									unknown protein
64658	+	+	+	+	+	+			Pex13 protein
64667	+	+	+				4.014 down	3.283 down	unknown protein
64672							1.298 down	4.986 down	Helix-turn-helix. AraC type
64676		+					3.856 up	2.420 up	unknown protein
64680	+	+	+	+	+	+			Histone acetyltransferase. catalytic subunit of the ADA and SAGA complexes
64683									FAD-binding domain
64684	+	+	+	+	+	+			chromatin assembly factor 1 subunit B. putative
64685	+	+	+	+	+	+			mitochondrial hypoxia responsive domain-containing protein
64696	+	+	+	+	+	+			unknown protein

64704				+	+	+	+	+	+			glycerol:H+ symporter (Gup1). putative
64710										1.372 down	4.096 down	AAA+-type ATPase
64719										1.795 down	2.267 down	Subtilisin like protease (SUB3)
64720												short chain dehydrogenase/reductase
64752				+	+	+	+	+	+			RNA polymerase N/8 kDa subunit
64757				+	+	+	+	+	+			unknown protein
64758												MFS permease
64777				+	+	+	+	+	+			unknown protein
64778												unknown protein
64784				+	+	+	+	+	+			Zn2Cys6 transcriptional regulator
64790				+	+	+	+		+			Acetate transporter required for normal sporulation; phosphorylated in mitochondria
64794				+	+	+	+	+	+			unknown protein
64818				+	+	+	+	+	+			Mitochondrial substrate carrier
64820				+	+	+	+	+	+			unknown protein with WD repeats
64827					+					1.302 down	3.227 down	GH36 raffinose synthase domain protein
64832				+	+	+			+			G/T mismatch-specific thymine DNA glycosylase
64834										1.158 down	2.680 down	unknown protein
64836												unknown protein
64866												DSBA-like thioredoxin domain-containing protein
64869												Cytochrome P450 CYP4/CYP19/CYP26 subfamilies
64874				+	+	+	+	+	+	1.271 up	2.365 down	MFS toxin efflux pump
64882										3.852 down	3.417 down	MFS permease
64898	+	+	+									unknown protein
64900												Cytochrome P450 CYP2 subfamily
64906												GH5 endo-β-1.6-glucanase

64916	+	+	+	+			1.689 up	2.720 down	unknown protein
64919							1.410 up	3.266 up	MFS permease
64920									Monocarboxylate transporter
64922									unknown protein. only present in Gibberella. Neurospora and Magnaporthe
64925	+	+	+	+		+	2.051 up	2.320 up	GT32 a-glycosyltransferase
64937							1.045 up	2.616 up	unknown protein
64938									dual specificity phosphatase
64951							1.147 down	3.583 up	unknown protein
64956							1.370 up	3.208 up	unknown protein
64959	+	+		+		+	13.328 down	2.437 down	phosphatidyl synthase
64963	+	+	+						unknown protein
64971							2.033 down	2.559 down	Amino acid permeases
64972									unknown protein
64989	+	+	+	+	+	+			unknown protein
64996							6.698 up	5.718 up	nitrilase
65004									unknown protein
65012	+								unknown protein
65018	+	+	+	+	+	+			unknown protein
65021							1.331 up	3.039 up	short chain dehydrogenase/reductase
65029							1.263 down	2.182 down	2OG-Fe(II) oxygenase superfamily protein
65033	+	+	+	+	+	+			unknown protein. secreted
65036									cytochrome P450 monooxygenase
65037							1.602 down	2.345 up	pyrophosphatase
65039									sexual development protein

65040							1.088 down	2.634 up	cytochrome P450. putative
65041									SAM-dependent methyltransferase
65046		+	+						unknown protein
65055		+							fatty acid desaturase
65060	+	+	+	+	+	+			unknown protein
65067							1.860 down	3.403 up	short chain dehydrogenase/reductase
65070									Zn2Cys6 transcriptional regulator
65085									MFS permease
65095							1.135 up	2.378 up	unknown protein
65096							1.360 up	2.422 up	3'-tRNA processing endoribonuclease
65097									Zinc-binding oxidoreductase
65098							1.379 up	4.278 up	FAD-binding domain and SignalP-predicted secretion signal. Distantly related to tre36816.
65102							1.007 up	2.577 up	unknown protein
65104	+	+	+	+	+	+			Vacuolar protein sorting-associated protein Vps13
65106							1.083 up	27.491 up	CN_hydrolase
65107	+	+	+	+	+	+	1.357 down	2.374 down	cytochrome P450
65114									MFS permease
65116							1.187 up	2.497 up	PKS
65117							2.782 down	2.425 down	ankyrin
65128	+	+	+	+	+	+			polynucleotide kinase 3 phosphatase
65133							1.370 up	2.840 down	Zn2Cys6 transcriptional regulator
65137									GH64 endo-1.3-β-glucanase
65141							1.022 up	2.394 up	cytochrome P450 monooxygenase
65142							1.800 up	2.379 up	aldehyde dehydrogenase ALDH
65147									unknown protein

65153							1.019 up	2.030 down	MFS permease
65156			+				1.887 up	2.672 up	S1/P1 nuclease
65162			+				3.710 up	5.381 up	GH18 endo-N-acetyl-ß-D-glucosaminidase Endo T
65164	+	+	+				1.711 down	2.468 down	unknown protein
65171									unknown protein
65172							1.018 up	2.221 up	PKS
65179									adenylosuccinate lyase
65190									nitrilase
65191									MFS permease (maltose permease)
65198									BioA Adenosylmethionine--amino--oxononanoate aminotransferase
65200	+	+	+	+	+	+			Vacuolar protein sorting-associated protein Vsp60
65209	+	+	+	+	+	+			unknown protein
65214	+	+	+	+	+	+			unknown protein
65215									CE4 imidase
65223							1.609 up	2.156 down	unknown protein
65225	+	+	+	+	+	+			Encodes a possible homologue of the S. cerevisiae UTP7 gene. encoding a component of t
65229							3.163 up	2.411 up	unknown protein
65232							1.132 up	3.068 down	short chain dehydrogenase/reductase
65242	+	+	+	+	+	+			adaptin N-terminal region. Homologue of yeast GCN1 which encodes a translational activa
65275									Glucose/sorbosone dehydrogenases
65286							1.227 up	2.536 down	unknown protein
65290	+	+	+	+	+	+			CPC1. cross-pathway control protein 1
65292	+	+	+	+	+	+			AMP-dependent synthetase and ligase
65295	+	+	+	+	+	+			Serine hydroxymethyltransferase
65313	+	+	+	+	+	+			Chromosome segregation protein

65315	+		+				16.360 down	11.572 down	bZIP transcription factor
65324	+	+	+	+	+	+	1.609 up	5.921 up	metacaspase CasA
65333							2.438 down	4.942 down	GH15 alpha-glycosidase (Glucoamylase and related glycosyl hydrolases)
65360									unknown protein
65380							2.788 up	2.669 up	GH47 α-1.2-mannosidase
65402	+	+	+	+	+	+			unknown protein
65406		+	+						cell wall glucanosyltransferase
65410	+	+	+	+	+	+			Phosphoadenosine phosphosulfate reductase
65411	+	+	+	+	+	+			nicotinate-nucleotide diphosphorylase
65433									D-arabinitol dehydrogenase ArbD
65437	+	+	+	+	+	+			unknown protein
65447	+	+	+	+	+	+			unknown protein
65452	+	+	+	+	+	+			SerB Phosphoserine phosphatase
65480	+	+	+	+	+	+			unknown protein
65483									unknown protein
65490									SpermidineSynthase
65493									MFS permease
65494							3.957 up	2.590 up	metallopeptidase
65496	+	+	+	+		+			short chain dehydrognease/reductase
65499							1.096 down	2.838 up	Tyrosine specific protein phosphatase and dual specificity protein phosphatase
65508	+	+	+	+	+	+	1.109 down	2.452 down	fructose-bisphosphatase
65522	+		+				10.051 down	4.155 down	unknown protein
65530	+	+	+	+	+	+			GTP-binding (elongation factor Tu).
65533			+						unknown protein
65547							1.235 up	3.156 down	mandelate racemase/muconate lactonase-like protein

65553				+	+	+	+	+	+			ubiquitin-conjugating enzyme
65555				+	+	+						unknown protein
65559				+	+	+	+	+	+			Respiratory-chain NADH dehydrogenase. 51 kDa subunit
65572										1.131 down	2.638 down	unknown protein
65583												MFS permease
65588												short chain dehydrognease/reductase
65591				+	+	+	+	+	+			26S proteasome regulatory complex subunit Rpn10
65595	+		+	+	+	+			+			unknown protein
65603				+	+	+	+	+	+	1.730 up	2.704 up	unknown protein
65607				+	+	+	+	+	+	1.071 down	2.279 down	RgsC. regulator of G-protein signaling
65615				+	+	+	+	+	+			RibosomalRNA methyltransferaseRrmJ/FtsJ domain.
65623				+	+	+	+	+	+			Sec61 gamma subunit
65625				+	+	+	+	+	+			unknown protein
65640				+	+	+	+	+	+	1.692 up	3.499 up	unknown protein
65646				+	+	+	+	+	+			GT α-1.6-mannosyltransferase
65659				+	+	+	+	+	+			calcium binding EF-hand protein
65671				+	+	+	+	+	+			unknown protein
65672					+		+		+			ubiquitin-protein ligase molybdopterin-converting factor
65677					+							Esterase/lipase/thioesterase
65685	+		+									histidinol phosphatase
65695				+	+	+						unknown protein
65705					+							unknown protein
65711						+				1.504 up	2.757 up	SAM-dependent methyltransferase
65717				+	+	+	+	+	+			NADH-ubiquinone oxidoreductase 10.5 kDa subunit
65718				+	+	+	+	+	+	2.383 down	2.523 down	unknown protein
65735												unknown protein
65736										2.122 up	4.485 up	catabolic 3-dehydroquinase

65739							1.548 down	4.721 down	unknown protein
65741			+						tryptophanyl-tRNA synthetase
65743									unknown protein
65744									MFS permease
65746			+				1.162 down	2.609 down	Zn2Cys6 transcriptional regulator
65750	+	+	+	+	+	+			RNA-binding. negative regulator of differentiation 1
65760									unknown protein
65768	+	+	+	+	+	+	1.275 up	2.053 down	unknown protein
65771							1.125 up	6.530 up	NACHT domain WD40 repeat-containing protein. related to HET
65773	+	+	+	+	+	+			unknown protein
65774	+	+	+	+	+	+			unknown protein
65782									lipase/esterase (α/ß fold)
65808	+	+	+	+	+	+			mitochondrial ribosomal protein MRPL31 (S. cerevisiae).
65810									unique protein
65816	+	+	+	+	+	+			unknown protein
65817	+	+	+	+			5.911 down	3.410 down	GT α-1.3-mannosyltransferase CMT1
65819							1.477 down	2.505 down	Molecular chaperone Hsp70 family
65821	+	+	+	+	+	+	1.160 down	2.113 down	vacuolar targeting protein Atg18
65832		+							Neurospora ro-7
65854									Zn2Cys6 transcriptional regulator
65869									Shikimate kinase
65873	+	+	+	+	+	+	1.464 down	2.278 down	protein kinase A. catalytic subunit
65880	+	+	+	+	+	+			voltage gated chloride channel
65882							1.350 down	2.411 down	dihydrodipicolinate synthase . putative

65883							4.669 down	4.131 down	D-isomer-specific 2-hydroxy acid dehydrogenase
65887	+	+	+	+	+	+			helix-turn-helix domain-containing protein
65891							1.574 down	2.603 down	PKS
65895	+	+	+	+	+	+			mediator of RNA polymerase II transcription subunit 31
65915									MFS permease
65921	+	+	+	+	+	+	1.005 down	3.414 up	Acetyl/propionyl-CoA carboxylase alpha subunit
65925							1.289 up	2.282 up	cytidine and deoxycytidylate deaminase zinc-binding region
65926	+	+	+	+	+	+			Mgs1. a DNA-dependent ATPase possibly involved in Okazaki fragment processing
65927	+	+	+	+	+	+			unknown protein
65933							2.112 up	2.174 up	Epl1/Sm1
65945	+	+	+	+	+	+			annexin ANXC4
65948									pirin
65949							7.410 up	2.214 up	unknown protein
65950							1.188 up	2.931 down	unknown protein
65957		+	+				1.022 down	2.086 down	unknown protein
65965	+	+	+	+	+	+			unknown protein
65969			+						aldolase. class II
65971									unknown protein
65975							1.609 down	2.796 down	unknown protein
65977									unknown protein with WD repeats
65982	+	+	+	+	+	+			GTPase activating protein for RabGTPases (Ras superfamily of smallGTPases). related to S.
65986							1.463 down	2.718 down	GH27 α-galactosidase
65992				+	+	+			unknown protein
65996	+	+	+	+	+	+			unknown protein

65997				+	+	+	+	+	+	1.751 up	3.188 up	Cyclophilin type peptidyl-prolyl cis-trans isomerase
66012				+	+	+	+	+	+			nuclear cap-binding protein complex. small subunit
66016										4.207 down	3.062 down	dienelactone hydrolase
66023					+					1.028 down	2.627 down	unknown protein
66034										1.104 down	5.924 down	unknown protein
66041				+	+	+			+			GH18 chitinase CHI18-18
66047												Zn2Cys6 transcriptional regulator
66059						+						unknown protein
66075				+	+	+	+	+	+			unknown protein
66077										1.149 down	3.847 up	ooc1-related protein
66087												integral membrane protein. Mpv17/PMP22 family. putative
66091				+	+	+	+	+	+			RNA polymerase II transcription factor. putative
66092					+	+				8.365 down	2.706 down	unique protein
66103										1.891 up	2.429 up	WbbJ Acetyltransferase (isoleucine patch superfamily)
66111												MRP-type ABC transporter
66117												short chain dehydrogenase/reductase
66128				+	+	+						Mec1p/ATR family of phosphatidyl inositol-3-kinase-like proteins involved in DNA damage
66132				+	+	+	+	+	+			AAA ATPase
66136				+	+	+	+	+	+			bimA
66141				+	+	+	+	+	+			Vacuolar sorting protein Pep3/Vps18
66154				+	+	+	+	+	+			eukaryotic rRNA processing protein EBP2
66161	+		+	+	+	+	+	+	+			unknown protein
66163				+	+	+	+	+	+			unknown protein
66165				+	+	+	+	+	+			unknown protein
66173				+	+	+	+	+	+			unknown protein
66175												short chain dehydrognease/reductase

66183									Peptidase_SSerine carboxypeptidase
66210							1.461 up	2.221 up	unknown protein
66218		+							unknown protein
66222									unknown protein
66228							1.186 up	3.451 up	unknown protein
66232	+	+	+	+	+	+			unknown protein
66256									unknown protein
66261			+						nucleoside phosphorylase
66268	+	+	+	+	+	+	1.083 down	2.323 down	unknown protein
66269	+	+	+	+	+	+			unknown protein
66270	+	+	+	+	+	+			Golgi membrane protein. coy1. with similarity to mammalian CASP
66276	+								unknown protein
66280	+	+	+						unknown protein
66286									unknown protein
66294	+	+	+	+	+	+			ATP-NAD/AcoX kinase
66296	+	+	+	+	+	+			eukaryotic and archaeal DNA primase
66303									unknown protein
66308	+	+	+	+	+	+			Metallocarboxypeptidase. putative
66311									MFS permease
66324									alpha/beta hydrolase
66329									unknown protein
66345							1.222 down	6.910 up	Mn superoxide dismutase
66351	+	+	+	+	+	+			unknown protein
66353	+	+	+	+	+	+			unknown protein
66370							1.105 down	4.544 up	unknown protein
66394		+					1.099 up	2.982 down	DNA polymerase family X member. Most closely related to DNA polymerase mu. an enzym
66405									transcriptional regulator. unknown

66421		+							unknown protein
66432									microsomal glutathione S transferase.
66433		+							GCN5 N-acetyltransferase
66436	+	+	+	+	+	+			Nop1. component of the SSU processome involved in processing of pre-18S rRNA
66437									unknown protein
66438	+	+	+	+	+	+			unknown protein
66453									cytochrome P450 monooxygenase
66469									unknown protein
66474	+	+	+	+	+	+			unknown protein
66480	+	+	+	+	+	+			RheB GTPase.Ras small GTPase.Ras-type.Related to Aspergillus fumigatusRheb GTPaseRh[illegible]
66484	+	+	+	+	+	+			Guanine nucleotide exchange factor. syt1
66486	+	+	+	+	+	+			kinesin-like motor protein Kar3p required for karyogamy
66510							1.090 down	2.723 up	short chain dehydrognease/reductase
66517	+	+	+	+					homoserine O-acetyltransferase
66521							2.283 up	2.125 down	unknown protein
66524	+	+	+	+	+	+			Complex_LYRComplex protein (LYR family)
66534							14.354 down	3.374 down	Cytochrome P450 CYP2 subfamily
66541	+	+	+	+	+	+			methylisocitrate lyase
66544									NAD binding NADP oxidoreductase coenzyme F420-dependent
66550	+	+	+	+	+	+			signal recognition particle receptor. beta subunit
66551							2.368 down	2.445 down	AAA ATPase
66562									unknown protein
66563							2.132 up	2.812 up	unknown protein
66583	+	+	+			+			unknown protein
66592	+		+						Fumarylacetoacetate (FAA) hydrolase
66598							3.037 down	3.008 down	NADPH:quinone oxidoreductase

66604	+	+	+	+	+	+			1-alkyl-2-acetylglycerophosphocholine esterase
66606							1.256 up	2.361 down	Zn2Cys6 transcriptional regulator
66608	+	+	+	+	+	+			dipeptidyl aminopeptidase. alpha factor processing
66609	+	+	+	+	+	+			unknown protein
66611									Amino acid transporter LysP
66614							1.167 down	3.892 down	alpha/beta hydrolase
66616									Purple acid phosphatase
66647									Haloacid dehalogenase-like hydrolase
66657							1.494 down	3.626 down	MFS permease
66662							1.148 down	3.023 down	Mandelate racemase/muconate lactonizing protein
66679									unknown protein
66687	+	+	+	+	+	+			b-glycosyltransferases
66689									Adenine deaminase/adenosine deaminase
66695	+	+	+	+	+	+			unknown protein
66696									unknown protein. Duf636
66702	+	+	+	+	+	+			Protein phosphatase 2C-like
66707	+	+	+	+	+	+			20S proteasome beta-type subunit Pre3
66719	+	+	+	+	+	+			unknown protein
66726							10.629 down	2.210 down	FAD binding protein
66751							1.261 down	2.246 up	unknown protein
66753							1.065 up	3.341 up	unknown protein
66758							1.479 down	2.110 up	Ankyrin
66766							1.758 down	19.235 up	Carbon-nitrogen hydrolase

66776										1.399 down	2.410 up	unknown protein
66786										4.715 down	2.649 down	unknown protein
66788										3.890 up	5.984 up	unknown protein
66789												GH75 chitosanase
66792				+	+	+	+	+	+			GH17 glucan endo-1.3-β-glucosidase
66795	+	+	+									GMC oxidoreductase family protein
66804				+	+	+	+	+	+	1.376 down	2.571 down	GH69: candidate a-glycosyltransferase
66814				+	+	+	+	+	+			GT α-1.3-mannosyltransferase
66819										1.208 down	2.170 down	Amino acid transporter LysP
66827										1.251 down	4.262 down	Zinc-containing alcohol dehydrogenase
66828				+	+	+	+	+	+	1.603 up	2.380 down	Zn2Cys6 transcriptional regulator
66832												GH3 β-glucosidase
66835				+	+	+	+	+	+			NADH-ubiquinone oxidoreductase 9.5 kDa subunit. putative
66843						+						GH16 cell wall glucanosyltransferase
66844				+	+	+	+	+	+			GPI ethanolamine phosphate transferase. putative
66854												Monocarboxylate transporter
66859										1.205 up	4.750 up	serine peptidase S28
66865				+	+	+				3.263 down	4.034 down	unknown protein
66877										1.094 down	2.003 down	unknown protein
66881				+	+	+	+	+	+			unknown protein
66888				+						11.913 down	5.286 down	GT α-1.3-mannosyltransferase
66895					+					1.971 up	4.834 up	3' exoribonuclease
66913				+	+	+	+	+	+			Phosphatidylinositol 3-kinase Vps34

66926	+	+	+	+	+	+			unknown protein
66928									Serine/threonine-protein kinase tel1
66929	+	+	+	+		+			unknown protein
66935									Phosphatidylserine decarboxylase
66937	+		+				1.717 down	4.094 down	FAD binding domain-containing protein
66950							1.020 up	2.504 down	serine/threonine protein kinase.
66958	+	+	+	+	+	+			Mitochondrial carrier protein
66960	+	+	+	+	+	+			zinc binding oxidoreductase
66963	+	+	+	+	+	+			MFS permease
66966	+	+	+	+	+	+			unknown protein
66985	+	+	+	+	+	+			stress activated MAP kinase interacting protein
66999							1.480 down	6.807 up	AMP-dependent synthetase and ligase. putative
67003									PabA. Anthranilate/para-aminobenzoate synthases component II;
67008	+	+	+	+	+	+			unknown protein
67010	+	+	+	+	+	+			ceramide synthase membrane component Lag1
67013									catalase
67024		+	+				1.411 down	3.681 up	unknown protein
67026									siderophore transporter
67030	+	+							unknown protein
67035	+	+	+	+	+	+	1.492 down	4.701 down	unknown protein
67052		+							unknown protein
67053	+	+	+	+	+	+	1.132 down	2.145 down	Ubiquitin carboxyl-terminal hydrolase
67057	+	+	+	+	+	+			silencing information regulator. Sir2 family
67079									unknown protein
67084									unknown protein

67107	+	+	+	+	+	+	2.535 up	3.231 up	MutS-related protein involved in mismatch repair
67108	+								unknown protein
67109							1.288 down	3.491 up	unknown protein
67110	+	+	+	+	+	+			unknown protein
67116	+	+	+	+	+	+			ADP-ribosylation factor-like protein. arl3; GTPase of the Ras superfamily
67117			+						3'-5' exonuclease
67130	+	+	+	+	+	+			elongator complex protein
67133	+	+	+	+	+	+	2.323 down	2.620 down	unknown protein
67147	+	+	+	+	+	+			dTDP-glucose 4.6-dehydratase
67174	+	+	+	+	+	+			origin recognition complex subunit Orc5. putative
67189									NRPS
67205									arginine-tRNA-protein transferase.
67209	+	+	+	+	+	+			Forkhead
67215	+	+	+	+	+	+			PrsA Phosphoribosylpyrophosphate synthetase
67217	+	+	+	+	+	+			iron-sulfur cluster assembly accessory protein Isa1. putative
67239									unknown protein
67272	+	+	+	+	+	+	1.492 down	2.516 down	CRO1. required for syncytial to cellular transision. involved in sexual development
67275		+	+				2.518 up	2.241 up	RAS1
67281	+	+	+	+	+	+			unknown protein with WD repeats
67286							1.279 down	3.345 down	unknown protein
67290							1.231 up	3.539 up	unknown protein
67295		+							unknown protein
67300	+	+	+	+	+	+			unknown protein
67324	+								unknown protein
67325	+	+	+	+	+	+			U3 small nucleolar ribonucleoprotein Mpp10
67334									MFS permease
67339	+	+	+	+	+	+			Zn2Cys6 transcriptional regulator

67350		+	+						unknown protein
67356	+	+	+	+	+	+			t-SNARE. Pep12. Golgi and vacuole
67360									unknown protein with WD repeats
67366	+	+	+	+	+	+			ribosomal protein MRPL49.
67377									Cytochrome P450 / E-class P450. group I
67382	+	+	+	+	+	+			endosomal t-SNARE Syn8
67394	+	+	+	+	+	+			unknown protein
67408		+	+						AMA1 which is an activator of meiotic anaphase promoting complex and required for initi
67418							1.399 up	3.015 up	C2H2 transcription factor
67420	+	+	+	+	+	+			Autophagic death protein Aut7/IDI-7
67430									unknown protein
67446									ThrC Threonine synthase
67448		+	+				1.030 down	3.245 down	AGA_beta. Aspartylglucosaminidase family
67467	+	+	+	+	+	+			SepB
67469	+	+	+	+	+	+	3.172 down	3.022 down	MFS permease (maltose permease)
67470									unknown protein
67472									unknown protein
67473	+	+	+	+	+	+			v-SNARE Vti1; cis-Golgi membrane traffic; vacuolar sorting pathways
67476	+	+	+	+	+	+			transcriptional regulator. unknown
67483	+	+	+						Zn2Cys6 transcriptional regulator
67484									AMP deaminase
67493									Alkaline phytoceramidase
67494							1.575 down	4.957 down	Golgi GDP-mannose transporter
67504									ribosomal protein S2.
67507	+	+	+	+	+	+			Triacylglycerol lipase
67509									unknown protein
67523	+	+	+	+	+	+			choline-phosphate cytidylyltransferase

67527									Mus81p. a subunit of the Mus81-Mms4 structure-specific endonuclease funtioning in mit
67534	+	+	+	+	+	+			serine/threonine-protein kinase
67538	+	+	+	+	+	+	26.140 down	6.954 down	Catalase
67540									unknown protein
67541									MFS permease
67546	+	+	+	+	+	+			unknown protein
67557	+	+	+	+	+	+			peptidyl-prolyl cis/trans isomerase
67562	+	+							unknown protein
67579							2.166 down	2.901 up	phospholipase A2
67588									NADH-dehydrogenase (ubiquinone)
67595	+	+	+	+	+	+			unknown protein
67597									unknown protein
67600							1.108 up	3.619 up	Fungal chitin synthase
67605							1.152 up	3.525 down	unknown protein
67607							1.160 up	4.956 up	unknown protein
67616		+					1.632 down	2.155 down	unknown protein
67627							1.034 up	2.664 up	short chain dehydrognease/reductase
67639	+		+				1.038 down	2.249 down	IlvB. Thiamine pyrophosphate-requiring enzymes
67642									unknown protein. UPF0075
67647	+	+	+	+	+	+			unknown protein containing a putative BTB/POZ domain
67658			+						A/G-specific adenine DNA glycosylase
67678							1.211 up	4.457 up	chitin deacetylase
67692							2.318 down	2.175 down	MFS permease
67698							1.450 up	2.387 up	nucleotide sugar dehydrogenase (UDP-Glc?)

67699							15.411 down	3.753 down	FAD/NAD-oxidoreductase. only in Hypocreaceae
67707							1.340 up	2.879 up	unknown protein with TLC domain
67717							1.614 down	3.520 down	unknown protein. ß-lactamase HMMPfam
67718									Malate/L-lactate dehydrogenase
67732									MRP-type ABC transporter
67738									unknown protein
67742									QDE1. RdRP. essential for quelling
67751									unknown protein
67752									MFS permease
67757									4.5-dioxygenase (only in Amanita muscaria)
67761			+				2.447 up	3.908 up	calpain-like protease
67772									Isoflavone reductase
67778									Hydroxysteroid 17-beta dehydrogenase 11
67795	+	+	+	+	+	+			Ankyrin
67806									Amino acid permease
67840	+	+	+	+	+	+			UTP11. encoding a component of the SSU processome
67844			+						GH76 α-1.6-mannanase
67859									aspartyl-tRNA synthase.
67866							3.890 down	2.442 down	unknown protein
67868									unknown protein
67882									unknown protein with NUDIX domain (hydrolase)
67902	+	+	+						unknown secreted protein
67907	+	+							transfer of mannosylphosphate
67923	+	+	+	+	+	+			unknown protein
67929									Cytidine/deoxycytidylate deaminase. zinc-binding region
67931									phosphatidylethanolamine N-methyltransferase
67938	+	+							short chain dehydrogenase/reductase

67941	+	+	+	+	+	+			unknown protein
67957	+	+	+	+	+	+			Cdk-activating kinase assembly factor (MAT 1)
67958							1.153 down	2.276 up	unknown protein. only present in Magnaporthe. Chaetomium and Gibberella
67964		+	+				2.182 down	2.462 down	Cytochrome P450 CYP2 subfamily
67971									MYND-type Zn-finger protein
67982	+	+	+		+	+			protein kinase WEE1. cell cycle control
67983		+	+						nuclear migration protein. ami1. of N. crassa
67988									unknown protein
68000	+	+	+	+	+	+			thiopurine S-methyltransferase. putative
68019							2.337 down	4.319 down	beta-lactamase superfamily
68022	+	+	+	+	+	+			geranylgeranyl diphosphate synthase. related to N. crassa albino-3
68026	+	+	+	+	+	+			integral peroxisomal membrane peroxin. putative
68028							1.039 down	2.340 down	unknown protein
68036							1.572 down	2.329 down	Cystathionine beta-lyases/cystathionine gamma-synthases
68058									unknown protein
68064	+	+	+	+					GH43 β-xylosidase/α-L-arabinofuranosidase
68067	+	+	+	+	+	+			meu10. a GPI-anhored cell wall protein.
68068	+	+	+	+	+	+	1.157 down	2.086 down	unknown protein
68072		+							HhH-GPD superfamily base excision DNA repair protein
68080	+	+	+	+	+	+			unknown protein
68086							1.050 up	3.395 down	unknown protein
68097	+	+	+	+	+	+			MADS-box
68104									unknown protein
68106	+	+	+	+	+	+			unknown protein

68107	+	+	+	+	+	+			Ribosomal protein S30 by homology with the corresponding protein of Ashbya gossypii.
68110	+	+	+	+	+	+			cyclin-dependent kinase regulatory subunit
68122	+	+	+						MFS permease
68130	+	+	+	+	+	+			unknown protein
68131							1.392 down	2.566 down	heterocompatibility domain protein
68154	+	+	+	+	+	+			unknown protein
68161							2.279 down	4.504 down	Arylacetamide deacetylase
68169							3.450 down	2.553 down	Calcium transporter
68178		+	+						unknown protein. HAD superfamily hydrolase
68184	+	+	+	+	+	+			pre-rRNA processing protein Esf1
68192			+						dynamin family protein
68204							1.209 up	3.375 down	NRPS
68207	+	+							SSCRP
68208	+	+	+	+	+	+			unknown protein
68212									GCPR. mPR-type
68230	+	+	+	+	+	+			N-methylhydantoinase A/acetone carboxylase HyuA. beta subunit
68254									Zn2Cys6 transcriptional regulator
68274									unknown protein
68279	+	+	+	+	+	+	4.046 down	5.749 down	Short-chain dehydrogenase/reductase
68291		+							pyruvate formate lyase activating enzyme (radical SAM superfamily)
68304	+	+	+	+	+	+			26S proteasome regulatory complex subunit Rpn5
68326	+	+	+	+	+	+			unknown protein
68336							1.171 up	2.489 up	short chain dehydrogenase/reductase
68338	+	+	+	+	+	+			NIPSNAP family protein
68347									GH18. chitinase CHI 18-16
68348							4.893 up	11.521 up	SAM-dependent methyltransferase

68358												unknown protein
68364				+	+	+	+	+	+			Protein kinase
68371										1.138 down	4.760 up	protein kinase. unusual
68401										1.199 up	3.353 up	unknown protein
68412				+	+	+	+		+			Sphingoid long-chain base kinase
68425	+	+	+									unknown protein
68427												metal-dependent phosphohydrolase
68430				+	+	+	+	+	+			unknown protein
68436				+	+	+	+	+	+			unknown protein
68438				+	+	+	+	+	+			vacuolar import and degradation protein. vid24
68444												haloacid dehalogenase-like hydrolase. putative
68455					+	+				1.145 down	2.398 down	Zn2Cys6 transcriptional regulator
68466										1.060 down	2.389 up	glycerone kinase
68470												GT4 glycosyl transferase
68479				+	+	+	+	+	+			unknown protein
68492				+	+	+	+	+	+			ounknown proteinsterol binding protein
68497				+	+	+	+	+	+			unknown protein
68499												unknown protein
68508										1.011 up	2.661 down	unknown protein
68522												Adenosine deaminase-related growth factors
68527				+	+	+	+	+	+			UVSD; probable regulator of DNA damage response
68548					+							unknown protein
68566				+	+	+						unknown protein
68571												AAA ATPase. central region
68574										7.717 down	3.343 down	unknown protein
68582				+	+	+						tRNA ligase TRL1

68585									galactose-6-phosphate isomerase
68587									unknown protein. only in Gibberella. Magnaporthe. Coccidioides and Chaetomium
68588									unknown protein
68590							1.715 down	2.584 down	Alcohol dehydrogenase. class V
68605									DNA/RNA methyltransferase. Shares significant amino acid sequence identity (E = 0.0) wit
68606									triose-phosphate isomerase
68608	+	+	+	+	+		1.260 down	2.507 down	Thiazole biosynthetic enzyme (Stress-inducible protein sti35)
68615									Amidase
68618									unknown protein
68622									unknown protein
68624	+	+	+	+	+	+			unknown protein
68636	+	+	+	+	+	+	1.349 up	2.275 up	ribosomal protein S11
68640							1.331 up	2.079 up	unknown protein
68647	+	+	+	+	+	+			unknown protein
68660									unknown protein
68662									aspartyl protease
68704									Arylacetamide deacetylase
68705	+	+		+	+	+	1.079 up	34.693 down	cytochrome P450 monooxygenase
68706									unknown protein. only in hypocreaceae
68717	+	+	+	+	+	+			unknown protein
68728	+	+	+	+	+	+			hexaprenyl pyrophosphate synthase
68729	+	+	+	+	+	+			unknown protein
68755							5.158 down	10.057 down	short chain dehydrogenase/reductase
68803									RTA1 like protein
68812									MFS permease
68813			+				3.217 down	2.939 down	MFS permease

68821										B-glycosyltransferase
68831										GT α-1.2-mannosyltransferase
68842										unknown protein
68843										unknown protein
68850		+	+	+	+	+	+			unknown protein
68869										MFS permease
68875		+	+	+	+	+	+			unknown protein
68876		+	+	+	+	+	+			unknown protein
68889								1.881 down	3.905 down	PDR-type ABC transporters
68909		+	+	+	+	+	+			40s ribosomal protein S26E (CRP5) (13.6 kDa ribosomal protein).
68924								7.616 up	16.584 up	cyclopropane/fatty acid synthase; plant related
68925								1.051 down	2.454 down	MFS permease
68926		+	+	+	+	+	+			actin polymerization protein Bzz1. putative
68927	+	+	+	+	+	+	+	1.069 up	2.266 down	unknown protein
68930										unknown protein
68941										Glutathione S-transferase
68948										unknown protein
68950										chloroperoxidase
68956				+						D-aspartate oxidase
68961										esterase
68966								1.034 down	2.063 down	flavoprotein. putative
68972										MFS permease
68973										acid phosphatase with metalloesterase domain
68988										unknown protein
68990								1.242 up	3.578 down	MFS permease
68997										unknown TPR domain protein. unknown

69013	+	+	+	+	+	+			unknown protein
69021	+	+	+	+	+	+			SSCRP
69026							1.237 down	2.080 down	MFS permease
69035	+	+	+	+	+	+			Choline phosphate cytidylyltransferase/Predicted CDP-ethanolamine synthase
69052									recombination hotspot-binding protein (Translin). putative
69055									unknown protein
69061	+	+	+						phosphoglycerate mutase
69064									SSCRP
69066									alpha/beta hydrolase
69068		+							contains: UBA/TS-N domain and DNA-binding domain
69077	+		+			+			Zn2Cys6 transcriptional regulator
69081									mitochondrial cytochrome b2. putative
69115									dienelactone hydrolase
69118	+	+	+	+	+	+			unknown protein
69122	+	+	+	+	+	+			fatty acid desaturase
69123	+	+	+	+	+	+			GH76 α-1.6-mannanase
69131	+	+	+	+	+	+	1.105 down	2.018 down	Unknown protein with FYVE/PHD zinc finger domain.
69141	+	+	+	+	+	+			neutral amino acid permeasde
69153	+	+							phosphoribulokinase/uridine kinase family protein
69164	+	+	+						MFS permease
69168									CipA (Aspergillus) oxidoreductase
69171							1.728 down	3.127 up	unknown protein
69179									molecular chaperone. contains ABC-1 domain
69181							1.129 down	3.553 up	unknown protein
69187									unknown protein
69189									unknown protein

69210							1.031 down	3.440 up	FoNIIA
69211	+	+	+	+	+	+			GT α-1.2-mannosyltransferase
69217	+	+	+	+	+	+			protease-like protein
69222	+	+	+	+	+	+			unknown protein
69224	+	+	+	+	+	+			F-box protein involved in recycling plasma membrane proteins internalized by endocytosi
69228							1.094 down	2.637 down	BioA Adenosylmethionine--amino--oxononanoate aminotransferase
69245							5.937 down	2.282 down	GH2 β-mannosidase
69257	+	+	+	+	+	+	1.344 up	2.311 up	Peptidase M22. glycoprotease
69267	+	+	+	+	+	+			unknown protein
69276							2.896 down	19.619 up	GH30 endo-β-1.4-xylanase
69281	+	+	+	+	+	+			cation diffusion facilitator family transporter
69282	+	+					3.538 down	4.297 down	monocarboxylate transporter
69287	+	+	+	+	+	+	1.318 up	2.617 up	unknown protein
69288		+							unknown protein
69290			+						Asparaginase. Asparaginase (amidohydrolase); 3' domain cd00204. ANK. ankyrin repeats
69291							1.081 up	2.804 up	FoNIIA
69303							7.661 up	4.594 up	unknown protein
69316									unknown protein
69349	+	+	+	+	+	+			Winged helix repressor DNA-binding
69362							1.078 down	3.080 down	phospholipase A2 protein family
69375							4.623 down	2.086 down	unknown protein
69381	+	+							nuclear protein Es2. putative
69384	+	+	+	+			1.593 down	2.602 down	unknown protein
69399	+	+	+	+	+	+	1.500 up	2.501 up	mitochondrial 40S ribosomal protein [Aspergillus niger]. Possible homologue of yeast SWS

69416		+					1.030 up	2.247 down	unknown protein
69423	+	+	+	+	+				unknown protein
69425									unknown protein
69426							2.487 down	2.651 down	copper transporter
69437	+	+	+	+	+	+			G2/mitotic-specific cyclin
69465									unknown protein
69468		+							unknown protein
69479	+	+	+	+	+	+	1.180 up	2.111 down	unknown protein
69483									unknown protein
69486							1.179 up	3.826 up	unknown protein
69489									serine carboxypeptidase
69490							1.036 down	3.654 up	CE4 chitin deacetylase
69493									GH92 α-1.2-mannosidase
69494									DCL1. Dicer-like protein. involved in quelling
69496	+	+	+	+		+			serine/threonine-protein kinase csk1
69500									PTH11 GPCR
69502	+	+	+	+	+	+			short chain dehydrogenase/reductase
69528	+	+	+	+	+	+	3.159 up	2.513 up	unknown protein
69529									α-ketoglutarate dependent (FeII) dioxygenase
69537									unknown protein. contains F-box and WD repeat
69555							1.111 up	3.726 up	aspartyl protease. Aspergillopepsin-like
69557									GH3 β-N-acetylglucosaminidase
69563									MFS permease
69569							2.283 up	2.392 up	unknown protein
69574							1.370 down	6.767 down	MFS permease
69605									Cystathionine beta-lyases/cystathionine gamma-synthases

69611										MFS permease
69613										unknown protein (Duf227)
69625										Glyoxylase
69647										enoyl-CoA hydratase/isomerase
69648								1.449 down	4.473 up	Cytochrome P450 CYP11/CYP12/CYP24/CYP27 subfamilies
69650										FAD binding protein
69651										MFS permease
69652										flavin-dependent halogenase O-methyltransferase bifunctional protein
69656										MYND zinc finger protein
69663										alpha/beta hydrolase
69679								1.091 up	3.165 up	ABC transporter
69686		+	+	+	+	+	+			unknown protein
69688								1.121 up	4.039 up	unknown protein
69692								1.557 up	21.254 up	Quinoprotein amine dehydrogenase beta chain-like protein
69695								1.123 down	9.330 down	Zn2Cys6 transcriptional regulator
69696								1.023 down	4.071 up	coenzyme F420-dependent N5.N10-methylene tetrahydromethanopterin reductase
69700										GH89 α-N-acetylglucosaminidase
69712								1.328 down	2.110 up	MFS permease
69727		+	+	+	+	+	+			unknown protein
69736										GH30 glucan endo 1.6-β-glucanase
69742								2.206 up	8.201 up	esterase
69751								1.838 up	2.952 down	GH16
69753	+									ADA. Adenosine deaminase
69755										L-asparaginase II. putative
69757			+	+				1.663 up	2.068 up	Peptidyl-tRNA hydrolase
69769										NADH:flavin oxidoreductase/NADH oxidase. putative

69771									Monocarboxylate transporter
69777							1.812 up	3.486 up	unknown protein
69791									unknown protein
69793	+	+	+	+	+	+			Cyclin associated with protein kinase Kin28p. which is the TFIIH-associated carboxy-termi
69805							1.046 down	2.384 up	unknown protein
69811							2.584 up	7.460 down	γ-glutamyltransferase
69816		+	+				1.168 up	2.477 up	aminoglycoside 3-N-acetyltransferase
69823									aminotransferase. putative
69825							2.347 up	2.300 up	unknown protein
69834									MFS permease.
69838	+	+	+	+	+	+			60S ribosome subunit biogenesis protein NIP7
69840									short chain dehydrogenase/reductase
69841							1.006 down	3.008 down	unknown protein
69853	+	+	+	+	+	+			unknown protein
69857							1.198 down	2.075 up	unknown protein
69858		+	+	+	+				diacylglycerol pyrophosphate phosphatase
69863			+						Amidase
69868							1.493 up	5.306 up	GDP-mannose a-mannosyltransferases;Distant relative
69870							1.058 up	3.223 up	FAD monooxygenase
69879		+					1.796 up	2.098 up	unknown protein
69881							1.086 up	2.584 up	Dit1p. spore wall maturation
69883							1.557 down	2.245 up	cytochrome P450. putative
69885							4.762 down	2.134 down	initiation factor 2B
69896							1.209 down	3.938 up	alpha/beta hydrolase

69901							1.300 down	2.309 up	unknown protein
69904	+	+	+						PTH11 GPCR
69920							1.058 up	2.467 up	unknown protein
69926									glycosyltransferases. Glycosyltransferases not yet assigned to a family
69928							1.113 up	3.432 up	unknown protein
69933							1.185 up	2.711 down	unknown protein
69944							3.310 down	3.091 up	GH31 α-xylosidase/α-glucosidase
69946							3.780 down	3.042 down	NRPS. siderophore synthase
69950		+					1.329 up	2.635 up	unknown protein
69956							7.346 down	2.442 down	Alcohol dehydrogenase. class V
69957									MFS permease
69960	+	+	+	+	+	+			HIR1 (histone transcription regulator)
69963									unknown protein
69972	+	+	+	+		+	1.364 down	4.630 down	Zn2Cys6 transcriptional regulator
69975	+	+	+	+	+	+			MFS permease
69998	+	+	+	+	+	+			Rad14. binsd to damaged DNA during nucleotide excisionRepair
70001									unknown protein
70017	+	+	+	+	+	+	1.423 up	5.487 up	mitochondrial ribosomal protein S19/S15.
70021							1.259 down	2.096 up	CE3 acetyl xylan Esterase
70025							1.429 down	5.203 down	alcohol dehydrogenase. zinc-containing. putative
70026	+	+	+	+	+	+			unknown protein
70028									unknown WD40 and NACHT domain protein. unknown
70035	+	+	+	+	+	+			GT α-1.3-mannosyltransferase
70040	+	+	+	+	+	+			Bet4p. alpha subunit of geranygeranyltransferase required for vesicle traffic between ER a

70071			+	+	+	+	+	+			Zn2Cys6 transcriptional regulator
70076			+	+							Inositol polyphosphate kinase. putative
70084			+	+	+	+	+	+			Nuclease (Borde 2007)
70090											Carbonic anhydrase
70092									1.736 down	2.636 down	unknown protein
70096									2.064 up	2.380 up	Gß-WD40 domain protein
70098									2.102 down	10.727 down	amino acid permease (GABA)
70108									1.302 down	4.649 down	MFS permease
70127											RTA1 domain protein. putative
70139											GCPR. mPR-type
70161											unknown protein
70172											MFS permease
70175											thioesterase family protein
70186									1.405 down	3.155 up	GH28 polygalacturonase/xylogalacturonan hydrolase
70191			+	+	+	+	+	+			unknown protein
70195									1.016 down	2.210 up	unknown protein
70197									46.788 down	2.467 down	Zn-dependent ß-lactamase
70201											GCN5-related acetyltransferase
70204	+	+									unknown protein
70218			+	+	+	+	+	+			unknown protein
70223			+	+	+	+	+	+			unknown protein
70251			+	+	+	+	+	+			vacuolar protein-sorting machinery class E protein HSE1
70266			+	+	+	+	+	+			CDC2_AJECA Cell division control protein 2
70305			+	+	+	+	+	+			Translation release factor eRF3 in other fungi.
70310				+							unknown protein

70311						+						flavoprotein monooxygenase
70316												NAD-dependent epimerase/dehydratase family
70319				+	+	+	+	+	+			MFS permease
70323				+	+	+	+	+	+			MFS monocarboxylic acid transporter. putative
70327										1.369 up	3.210 up	unknown protein
70329	+	+	+									unknown protein
70334										2.761 down	8.772 down	short chain dehydrogenase/reductase
70339										1.120 down	2.200 up	cytochrome P450 monoounknown proteingenase (trichothecene C-15 hydrounknown pro
70341												GH75 chitosanase
70349										3.978 down	8.651 down	MFS permease LIZ1
70351						+						Zn2Cys6 transcriptional regulator
70355				+		+	+		+	5.994 down	18.605 down	SAM-dependent methylransferase
70365										16.042 down	10.414 down	unknown protein
70373										1.837 down	6.019 down	unknown protein
70375										3.347 down	12.561 down	amidase
70377												unknown protein
70383										1.814 down	4.805 down	succinate semialdehyde dehydrogenase. NADP
70397												alanyl-transfer RNA synthetase.
70400										1.135 up	3.069 up	unknown protein
70414										1.160 down	3.363 down	Zn2Cys6 transcriptional regulator
70429												Short-chain dehydrogenase/reductase
70439				+	+	+	+	+	+	1.997 up	2.507 up	NADH-ubiquinone oxidoreductase
70452												flavin containing amine oxidoreductase

70488		+	+						unknown protein
70491							70.700 down	2.492 down	esterase/lipase
70500									unknown protein. 6TM
70511	+	+	+	+	+	+			peroxisomal carrier protein
70517	+	+	+	+	+	+			Forkhead
70520									short chain dehydrogenase/reductase
70527									lysophospholipase
70532	+	+	+	+	+	+			acyltransferase. putative
70542									ß-glycosidase (endo-beta-1.3(4)-β-D-glucanase)
70546	+	+	+	+	+	+			Poly(A) polymerase. RNA-binding region
70547	+	+	+						Zn2Cys6 transcriptional regulator
70548	+	+	+	+	+	+			Guanine nucleotide exchange factor for Ras-likeGTPases (RasGEF)
70560	+	+	+	+	+	+			Calpain-like protease palB/rim-13. putative
70570							1.047 up	3.243 down	Zn2Cys6 transcriptional regulator
70577	+	+	+	+	+	+			C2H2 transcriptional regulator
70599							1.258 up	2.636 up	unknown protein
70600							17.584 down	7.834 down	catalase. large subunit type
70608							32.737 down	13.100 down	unknown protein. HHE domains
70630							1.033 down	2.060 down	homoserine acetyltransferase family protein
70631							1.175 down	2.285 up	dipeptidyl peptidase 5
70639									unknown protein with WD repeats
70644									Flippase
70646									CCD1
70703	+	+	+	+	+	+			CDP-alcohol phosphatidyltransferase
70736	+	+	+	+	+	+			Arv1-like family protein

70750				+	+	+	+	+	+			ATP synthase subunit H. putative
70772				+	+	+	+	+	+			GPI inositol deacylase of the ER Bst1
70778	+	+			+							DNA mismatch repair protein PMS1
70795				+	+	+	+	+	+			translation factor eIF2
70800						+						elastinolytic metalloproteinase
70803										2.003 down	4.754 up	bifunctional catalase/peroxidase
70806												SSCRP
70807												unknown protein
70808												unknown protein
70811				+	+	+	+	+	+			cell division control protein Cdc4. putative
70816												Asparaginase_
70823				+	+	+	+	+	+			GPI transamidase component GPI16
70829										1.944 down	6.140 down	MFS multidrug transporter. putative
70830										1.280 up	2.310 down	MFS permease
70837										1.519 up	2.084 up	E3 ubiquitin ligase
70838												unknown protein
70840				+	+	+	+	+	+			SSCRP
70842										2.181 down	5.192 down	Cytochrome P450 CYP2 subfamily
70845										3.171 up	5.855 up	GH55 β-1.3-glucanase
70847	+	+	+									unknown protein
70855										1.629 up	2.052 up	unknown protein
70859				+		+	+		+	4.320 down	5.344 down	amidase
70860												allantoate permease. 10 TM domains
70861						+						unknown protein
70875				+	+	+	+	+	+			serine/threonine-protein kinase

70894					+	+				5.560 down	3.395 down	unknown protein
70907						+				3.594 up	4.621 up	Ankyrin
70910										2.008 down	11.340 down	GMC oxidoreductase
70912												short chain dehydrogenase/reductase
70918						+				1.053 down	8.118 up	unknown protein
70919										3.550 up	3.373 up	SSCRP
70921										1.347 down	3.785 up	unknown protein
70922												unknown protein
70923										1.543 up	2.682 down	unknown protein. only in fungi
70927										1.116 up	3.319 up	unknown protein
70932										1.280 down	2.064 down	MFS permease
70933												MFS permease
70934	+	+	+									oligopeptide transporter
70943				+	+	+	+	+	+	1.137 down	2.022 down	Histidine kinase. part of a two component signal transduction system
70949												Thiamine pyrophosphate-requiring enzyme / benzoyl formate decarboxylase
70956										4.615 down	3.284 down	cytochrome P450 monooxygenase
70960												ADA. Adenosine deaminase
70961										2.050 down	2.579 down	dienelactone hydrolase
70962										1.001 up	4.010 up	tripeptide peptidase
70967												unknown protein
70972												unique protein
70973	+	+	+							1.142 down	2.000 up	4-aminobutyrate aminotransferase

70984												cytochrome P450 protein. class I
70988												Flavin-containing monooxygenase
70991										4.481 up	3.539 up	unknown protein. ThiJ/PfpI domain
70994												dipeptidyl peptidase 5
70996										1.238 up	2.219 up	SSCRP
70998												Amino acid transporters
71005												NRPS
71008												siderophore transporter
71010										2.126 down	4.473 down	MDR-type ABC transporters
71019				+	+	+	+	+	+			unknown protein
71021				+	+	+	+	+	+			bem46 family protein
71029	+	+		+	+	+	+	+	+	3.606 up	9.178 up	Ctr copper transporter. putative
71034												unknown protein
71037				+	+	+	+	+	+			nonselective cation channel. putative
71039												-HAO-hydroxyanthranilic acid dioxygenase
71050												SAM-dependent methyltransferase MTQ2
71059										1.410 down	2.224 down	MFS permease
71071												unknown protein. only in Gibberella. A. fumigatus and A. nidulans
71072										4.958 down	3.818 down	Gluconate kinase
71076										1.093 up	3.337 up	estrase/lipase
71077												unknown protein
71078										1.038 up	3.064 up	Ca/CaM-dependent kinase-1
71080										1.325 down	3.746 down	Zn2Cys6 transcriptional regulator
71092										2.724 up	5.525 up	phytase
71094										4.531 up	2.569 down	Intradiol ring-cleavage dioxygenase
71095										1.300 up	3.922 up	Aldehyde dehydrogenase

71101										1.010 up	5.575 up	enoyl-CoA hydratase/isomerase
71103										1.281 up	3.159 up	dynamin GTPase. putative
71117				+	+	+	+	+	+			a-glycosyltransferases
71119				+	+	+	+	+	+			capsule polysaccharide biosynthesis protein. putative
71123										1.810 up	8.852 up	unknown protein
71125	+									1.057 up	3.678 up	unknown protein
71126		+										
71146	+		+	+		+				1.114 up	3.012 up	unknown protein
71154	+		+	+		+				1.158 up	2.392 up	unknown protein
71166										1.061 up	5.009 up	unknown protein
71167	+			+		+				1.202 down	3.268 up	SSCRP
71170	+	+	+									unknown protein. only in Gibberella
71173	+	+	+							1.536 up	3.313 up	unknown protein
71177	+		+	+		+						unique protein
71180	+		+	+		+				1.436 up	3.483 up	unknown protein
71259				+	+	+	+	+	+			triacylglycerol lipase
71284												unknown protein
71304				+	+	+	+	+	+			ribosomal protein L39 from yeast and fungal species.
71315				+	+	+	+	+	+			protein kinase. related to S. cerevisiae Cla4p. a member of the PAK family
71326												Esterase;too long in N-term; 214 was better
71343				+	+	+	+	+	+			cytochrome oxidase c subunit VIb
71344				+	+	+	+	+	+			unknown protein
71363				+	+	+	+	+	+			translation elongation factor 3-like protein
71371				+	+	+	+	+	+			Acetyltransf_Acetyltransferase (GNAT) family
71374				+	+	+	+	+	+			Membrane coat complex Retromer. subunit Vps35
71380				+	+	+	+	+	+			HMG-CoA reductase.
71390										1.059 up	2.255 up	MRSP1/expansin-like
71394												GH79 β-glucuronidase

71399							2.328 down	2.149 down	endo-1.3-β-glucanase
71410	+	+	+	+	+	+			translation initiation factor eIF4-gamma/eIF5/eIF2-epsilon.
71441	+	+	+	+	+	+			unknown protein
71468	+	+	+	+	+	+			replication factor A. large subunit
71496									Zinc-binding dehydrogenase
71532							13.909 down	3.294 down	GH71 α-1 3-glucanase
71554							1.084 down	3.111 up	GH5 β-1.3-mannanase/endo-β-1.4-mannosidase
71556							3.190 down	3.546 down	unknown protein
71559	+	+	+	+	+	+			Translation initiation factor eIF-5A gene based on homologyToThe corresponding genes i
71563	+	+	+	+	+	+			Chitin synthase
71566			+						phytase
71649									tRNA-dihydrouridine synthase.
71655	+	+	+	+	+	+			sterol delta(24(28))- reductase.
71676	+	+	+	+		+			ureidoglycolate hydrolase
71689		+							Zn2Cys6 transcriptional regulator
71759	+	+	+	+	+	+			tyrosyl-tRNA synthetase. class Ib.
71783	+	+	+	+	+	+			Aspartyl-tRNA synthetases
71791	+	+	+	+	+	+			UTP14. encoding a component of the SSU processome
71817									unknown protein
71823									Zn2Cys6 transcriptional regulator
71970	+	+	+	+	+	+			phosphatidylinositol-specific phospholipase C. Y domain. putative
71994	+	+	+	+	+	+			Ubiquitin-conjugating enzyme Ubc1
72012	+	+	+	+	+	+			GT8 glycogenin
72042	+	+	+	+	+	+			unknown protein
72057			+						Zn2Cys6 transcriptional regulator
72071							1.184 up	3.438 up	Carbohydrate-Binding Module Family 13

72072							3.402 down	2.039 down	CE1 esterase (PHB?)
72076									Zn2Cys6 transcriptional regulator
72086									Vacuolar carboxypeptidase Cps1
72091									unknown protein
72108	+	+	+						DNA repair protein
72137	+	+	+	+					cytosceleton binding protein
72158	+	+	+	+	+	+			Rad9
72183							3.161 up	2.115 down	SSCRP
72231	+	+	+	+	+	+			unique protein
72259	+	+	+	+					RgsA. regulator of G-protein signaling
72287	+	+	+	+	+	+			tRNA (uracil-5-)-methyltransferase/TrmA.
72321									subtilisin-like serine protease
72339		+			+				GH18. chitinase CHI18-9
72379	+	+	+						conidiospore surface protein cmp1
72383	+	+	+	+	+	+			galactose permease
72408									kinesin-like protein
72459	+	+	+	+	+	+			Phosphatidylinositol-4-phosphate 5-kinase. mss4
72488	+		+				1.527 up	2.387 down	GH95 α-L-fucosidase
72521	+	+	+	+	+	+			transcription elongation factor SPT4
72524									bZIP transcription factor
72526									GH67 α-Glucuronidase GLR1
72549									phenylacrylic acid decarbounknown proteinlase
72567			+				15.565 down	4.797 up	GH6 Cellobiohydrolase CEL6A/CBH2
72568									GH79 ß-glucuronidase
72581							2.158 down	5.960 up	unknown protein

72602				+	+	+	+	+	+	1.905 down	2.632 down	ribosomal protein P2.
72605										1.190 down	2.628 up	GPCR. secretin like
72606				+	+	+	+	+	+			ubiquitin-activating enzyme UBA1
72611				+	+	+	+	+	+	1.386 down	5.511 down	Zn2Cys6 transcriptional regulator
72612						+				2.100 down	3.496 down	acetylornithine deacetylase
72615				+	+	+	+	+	+			LysU Lysyl-tRNA synthetase (class II)
72632										5.777 down	12.000 down	GH27 α-galactosidase AGL1
72643												arginosuccinate synthetase
72661				+								unknown protein
72685				+	+	+	+	+	+			6-phosphogluconate dehydrogenase. decarboxylating
72694				+	+	+	+	+	+			transferase hexapeptide domain protein
72704												GH27 α-galactosidase AGL3
72780	+	+	+									bZIP transcription factor
72788												GT 31 glycosyltransferase
72796				+	+	+	+	+	+			unknown protein with Nif domain
72800				+	+	+	+	+	+			unknown protein
72859				+	+	+	+	+	+			unknown protein UPF0023
72896				+	+	+	+	+	+	1.277 up	2.294 up	Dolichol-phosphate (beta-D) mannosyltransferase 2
72907					+					1.083 down	3.047 down	unknown protein with WSC domain
72918				+	+	+	+	+	+			ribosomal protein L19
72922										10.758 down	7.407 down	amino acid permease (GABA)
72941				+	+	+	+	+	+			unknown protein
72993												Zn2Cys6 transcriptional regulator
73005										1.061 up	3.560 up	GH79 β-glucuronidase

73016										1.132 up	5.225 up	aryl-alcohol oxidase
73023										1.013 up	2.949 up	unknown protein
73024										1.673 up	5.217 up	unknown protein
73039										1.333 down	3.926 down	APHPhosphotransferase enzyme family aligned
73048					+	+				1.975 up	2.846 up	unknown protein
73101										1.099 down	2.796 up	glucan endo-1.3-1.4-β-D-glucosidase
73102										1.966 down	2.636 down	GH39 β-xylosidase
73103	+		+							1.100 up	2.797 up	ribosomal protein P2.
73104										1.576 down	2.634 down	Zn2Cys6 transcriptional regulator
73110	+	+	+									Acetyltransf_Acetyltransferase (GNAT) family
73119										1.119 up	3.672 up	unknown protein
73134												GT20 trehalose-6-phosphate synthase/trehalose phosphatase
73173										1.500 up	3.381 up	HFB1
73179										3.182 up	3.464 up	GH71 α-1 3-glucanase
73190				+	+	+			+			Rev3p of Saccharomyces cerevisiae. the catalytic subunit of DNA polymerase zeta. a DNA
73231				+	+	+	+	+	+			ribosomal protein L34.
73248										2.266 down	2.413 down	GH55 exo-1.3-β-glucanase
73250										1.239 down	8.723 up	Urea transporter
73256												GH81 endo-1.3-β-glucanase
73344										1.136 up	3.532 up	Cytochrome P450 CYP3/CYP5/CYP6/CYP9 subfamilies
73417				+	+	+	+	+	+	1.432 down	2.331 down	bZIP transcriptional regulator MeaB
73501				+	+	+	+	+	+			unknown protein
73504					+							unknown protein
73516				+	+	+	+		+	1.590 down	2.049 down	Grg1 Glucose repressible protein

73517	+	+	+	+	+	+			unknown protein
73519	+	+	+	+	+	+			unknown protein. 4 TM
73523	+	+	+						unknown protein with WSC domains
73525	+	+	+	+	+	+			importin ß KapE
73536			+				1.012 up	10.116 up	NADP-glutamate dehydrogenase
73539	+	+	+	+	+	+			unknown protein
73559	+	+	+						Zn2Cys6 transcriptional regulator
73560	+	+	+	+	+	+			UTP20. snoRNA-binding protein and member of the SSU processome.
73564	+	+	+	+	+	+			20S proteasome alpha-type subunit Pre10
73570	+	+	+	+	+	+			unknown protein
73571	+	+	+	+	+	+			Cytochrome c1. heme protein. mitochondrial
73574	+	+	+	+	+	+			19S regulatory particle ATPase Rpt1
73580	+	+	+		+				unknown protein
73587			+						unknown protein
73594							1.712 up	2.329 up	unknown protein
73604	+	+	+	+	+	+			phytase
73618									PKS
73621									PKS
73623							1.317 down	2.574 up	flavoprotein monooxygenase
73631									isoamyl alcohol oxidase
73632							6.821 down	15.028 up	CE5 acetyl xylan esterase AXE1
73638			+				16.794 down	24.546 up	CIP1
73643							25.659 down	22.910 up	GH61 polysaccharide monooxygenase CEL61a
73646	+	+	+	+	+	+			DNA replication licensing factor mcm4
73647	+	+	+	+	+	+			Vesicle coat complex COPI. epsilon subunit
73654			+						BZIP transcriptional regulator
73665	+	+	+						hexokinase

73678	+	+	+	+	+	+			calnexin. high identity with A. niger clxA
73689	+	+	+	+	+	+			C2H2 transcriptional regulator
73690	+	+	+	+	+	+			Cytosolic Fe-S cluster assembly factor NAR1. putative
73697	+	+	+	+	+	+			unknown protein
73706	+	+	+	+	+	+			unknown protein
73708	+	+	+	+	+	+			unknown protein
73722		+							unknown protein
73733	+	+	+	+	+	+			unknown protein
73749	+	+	+	+	+	+			unknown protein
73760	+	+	+	+	+	+			unknown protein
73764	+	+	+	+	+	+			nucleoside transporter
73765	+	+	+	+	+	+			unknown protein
73769	+	+	+	+					Unknown protein with WD40 repeats and a LisH domain which mediation protein dimeris
73774	+	+	+	+		+			acetyl-CoA synthase
73778	+	+	+	+	+	+			Rfc5p of Saccharomyces cerevisiae.
73783	+	+	+	+	+	+	1.032 up	2.759 up	mitochondrial co-chaperone GrpE . putative
73790	+	+	+	+	+	+			Mitochondrial ATP synthase epsilon chain domain-containing. putative
73792	+	+	+				1.145 up	2.298 down	Zn2Cys6 transcriptional regulator
73809	+	+	+	+	+	+			metallopeptidase. Ste23
73815	+	+	+	+	+	+	1.436 down	2.114 down	unknown protein
73818									Catalase
73823	+	+	+	+	+	+			SCF complex subunit Skp1
73829									unknown protein
73832	+	+	+	+	+	+			transcription factor SipA3
73837	+	+	+	+	+	+			Peptide chain release factor eRF/aRF subunit 1 (eRF1).
73840	+	+	+	+	+	+			unknown protein
73842	+	+	+	+	+	+			adenosine_kinase
73846	+	+	+	+	+	+			NADH-ubiquinone oxidoreductase Complex1 subunit

73853	+	+	+	+	+	+				NADH-cytochrome b5 reductase
73862	+	+	+	+	+	+				methionine aminopeptidase
73873		+								ProB Glutamate -kinase [Amino acid transport and metabolism]
73876	+	+	+	+	+	+				Mitochondrial inner membrane protease atp-23
73881	+	+	+	+	+	+				unknown protein
73895	+	+	+	+	+	+				RNA-binding protein with KH domain
73897										Trypsin-like protease
73903	+	+	+	+	+	+				6-phosphogluconolactonase
73904		+					4.345 up	3.426 up		unknown protein
73912	+	+	+	+	+	+	1.438 up	2.263 up		unknown protein
73918										NOL1/NOP2/sun domain protein
73924							2.517 up	2.535 up		MDR-type ABC transporters
73933	+	+	+	+	+	+				unknown protein
73934	+	+	+	+	+	+				IlvH Acetolactate synthase small (regulatory) subunit
73937	+	+	+							Ornithine carbamoyltransferase OTC/ARG3
73940	+	+	+	+	+	+				unknown protein
73961	+	+	+	+		+				unknown protein
73963	+	+	+	+	+	+				cytokinesis regulator. putative
73967	+	+	+	+	+	+				cofilin
73975	+	+	+	+	+	+				GYF domain-containing protein
73985	+	+	+	+	+	+				Unknown protein
73997	+	+	+	+	+	+				Acyl CoA binding protein. putative
74017		+					1.207 down	2.458 down		unknown protein
74020	+	+	+	+						Orotidine 5'-phosphate decarboxylase
74026	+	+	+	+	+	+				CDP-alcohol phosphatidyltransferase
74030	+	+	+	+	+	+				Protein phosphatase 2C
74041	+	+	+	+	+	+				Aspartate/tyrosine/aromatic aminotransferase
74047	+	+	+	+	+	+				Ribonucleotide reductase large subunit
74054	+	+	+	+	+	+	1.020 up	2.139 up		Exocyst component Sec6

74057	+	+	+	+	+	+			Ca2+ permeable channel. related to N. crassa NCU07605.1
74060	+	+	+	+	+	+	1.822 up	10.096 up	SSCRP
74070	+	+	+	+	+	+			Amino acid transporter
74080	+	+	+	+	+	+			Pantoate_ligasePantoate-beta-alanine ligase
74086									unknown protein
74091	+	+	+						Zn2Cys6 transcriptional regulator
74099	+	+	+	+	+	+			unknown protein
74103									glutathione-S-transferase theta
74118	+	+	+	+	+	+			unknown protein
74122	+	+	+	+	+	+			GAL1 Galactokinase
74123	+	+	+	+	+	+			acetolactate synthase
74129		+							unknown protein
74156									aspartyl protease
74158	+	+	+	+	+	+			ribosome biogenesis protein Ssf2. putative
74162	+	+	+	+	+	+	1.277 down	2.200 down	Helicase. C-terminal
74163	+	+	+	+	+	+			unknown protein
74168	+	+	+	+	+	+			Dolichyl-phosphate mannosyltransferase
74172									unknown protein
74177	+	+	+	+	+	+			coiled-coil domain-containing protein. putative
74187									AAA+-type ATPase
74194	+	+	+	+	+	+	3.702 down	3.064 down	mannitol dehydrogenase LXR1
74198									GH92 α-1.2-mannosidase
74214							2.813 down	2.706 down	unknown protein
74215							1.792 up	2.347 down	RTA1-like protein. 7 TM. responds to xenobiotic stimuli
74223									GH11 endo-ß-1.4-xylanase XYN1
74228							2.632 up	2.849 up	oleate δ-12 desaturase
74233	+	+	+	+	+	+			TPA: decapping enzyme Dcp2

74235	+	+	+	+	+	+			ribonucleotide reductase
74241	+	+	+	+	+	+			nucleolysin TIA-1
74252	+	+	+						Translation initiation factor 6 (eIF6) by homologyToThe corresponding protein in other eu
74258	+	+	+	+	+	+			beta-subunit of phenylalanyl-tRNA synthetase
74267	+	+	+	+	+	+			Delta subunit. ret2. of the coatomer complex (COPI). which coats Golgi-derived transport
74278	+	+	+	+	+	+	2.893 down	2.268 down	Pyridine nucleotide-disulphide oxidoreductase. class-II
74282									QI74 orthologue
74289									unknown protein
74318	+	+	+	+	+	+			importin ß KapB
74323									unknown protein
74346	+	+	+	+	+	+			Translation elongation factor precursor from Aspergillus fumigatus.
74351	+	+	+		+	+			Flavin-containing monooxygenase Fmo1
74356	+	+	+	+	+	+			tRNA (cytosine-5-)-methyltransferase NCL1
74364			+						unknown protein. 1 TM secreted
74366									nitrilase
74374	+	+	+	+	+	+			rRNA-processing protein FCF1
74375	+	+	+	+	+	+			nitrogen metabolite repression regulator NmrA
74379							1.175 up	2.035 up	ERG2 C-8 sterol isomerase; catalyzes the isomerization of the delta-8 double bond to the d
74381	+	+	+	+	+	+			INO80 chromatin remodeling complex Ies1
74386	+	+	+	+	+	+			Vacuolar protein sorting-associated protein 68
74390	+	+	+	+	+	+			unknown protein
74397	+	+	+	+	+	+			unknown protein
74400	+	+	+	+	+	+			glycogen synthase kinase 3
74406	+	+	+	+	+	+			unknown protein
74409	+	+	+	+	+	+			Ribosomal protein L5 domain.
74421	+	+	+	+	+	+	1.164 up	2.954 up	unknown protein
74437									unknown protein
74442	+	+	+	+	+	+			unknown protein
74444									DNA-directed RNA polymerase II

74449	+	+	+	+	+	+			unknown protein
74453		+							3-hydroxyacyl-CoA dehydrogenase
74462	+	+	+	+	+	+			Signal peptidase complex subunit Spc3
74476							1.512 up	2.470 down	unknown protein
74477									unknown protein
74480	+	+	+	+	+	+			UTP13. encoding a component of the SSU processome
74486	+	+	+	+	+	+	1.513 up	2.165 up	unknown protein
74491									unknown protein
74498	+	+	+	+	+	+			translation initiation factor 2 beta subunit (Interpro motif IF5).
74502	+	+	+	+	+	+	1.539 down	2.020 down	Aldehyde dehydrogenase
74505		+							UDP-N-acetylglucosamine transferase subunit
74508	+	+	+	+	+	+			unknown protein
74515	+	+	+	+	+	+	2.508 down	2.449 down	unknown protein
74517	+	+	+	+	+	+			Kex1p involved in alpha factor processing
74531	+	+	+	+	+	+			transcriptional regulator ASM-1. involved in fruiting body development
74534	+	+	+	+	+	+			unknown protein
74559	+	+	+	+	+	+			autophagocytosis protein Aut1. putative
74563							1.067 down	3.411 down	unknown protein
74568	+	+	+	+	+	+			unknown protein
74570	+	+	+	+	+	+			unknown protein
74576	+	+	+	+	+	+	1.033 down	2.544 down	BZIP transcriptional regulator
74580	+		+						unknown protein. 3 TM domains
74601	+	+	+	+	+	+			OPT family small oligopeptide transporter
74622							1.068 up	2.317 up	unknown protein
74624	+	+	+	+	+	+			unknown protein
74635	+	+	+	+	+	+			unknown protein

74638									unknown protein
74681		+							unknown protein
74682	+	+	+	+	+	+			unknown protein
74693									leucine carboxyl methyltransferase. putative
74701							1.043 down	2.336 down	GTP-binding protein involved in protein synthesis
74711									Erg8p of Saccharomyces cerevisiae; phosphomevalonate kinase
74725	+	+	+	+	+	+			aspartate/other aminotransferase
74731	+	+	+	+	+	+			beta-adaptin. apl1. large subunit of the adaptor protein complex (AP-2) of clathrin-coated
74745									unknown protein
74751									unknown protein
74765									Bromodomain containing protein
74771	+	+	+	+	+	+			unknown protein
74774	+	+	+	+	+	+			Translation initiation factor 3. subunit g (eIF-3g); homologue of yeastTIF35.
74782	+	+	+	+	+	+			survival factor 1
74795	+	+	+	+	+	+			unknown protein
74797	+	+	+	+	+	+			nuclear segregation protein (Bfr1). putative
74804									Tubulin cofactor D
74807			+						GH76 GPI-anchored α-1.6-mannanase
74818	+	+	+	+	+	+			ATP-dependent RNA helicase DBP10
74848	+	+	+	+	+	+			Rho-type GTPase-activating protein
74851									unknown protein
74854	+	+	+	+	+	+			stearic acid desaturase Sde1
74857	+	+	+	+	+	+			unknown protein
74861	+	+	+	+	+	+	1.000 up	2.614 down	unknown protein
74884	+	+	+	+	+	+			serine/threonine-protein phosphatase PP2A catalytic subunit
74892	+	+	+	+	+	+			ATP synthase regulation protein NCA2. putative
74902	+	+	+	+	+	+			unknown protein
74907			+			+			unknown protein

74913	+	+	+	+	+	+			Histidinol-phosphate/aromatic aminotransferase and cobyric acid decarboxylase
74932	+	+	+	+	+	+			unknown protein
74933	+	+	+	+	+	+			unknown protein
74943	+	+	+	+	+	+	1.760 up	2.763 up	Diphthine synthase
74946	+	+	+	+	+	+			unknown protein
74947	+	+	+	+	+	+			glycosylphosphatidylinositol transamidase
74953									MFS permease
74962	+	+	+	+	+	+			unknown protein with WD40 repeats
74971	+	+	+		+		1.002 down	2.104 down	unknown protein
74983	+	+	+	+	+	+			Isocitrate dehydrogenase. subunit 2. NAD-dependent. mitochondrial
74987	+	+	+	+	+	+			unknown protein
74989	+	+	+	+	+	+			unknown protein
74997	+	+	+	+	+	+			unknown protein
75000	+	+	+	+	+	+			3-isopropylmalate dehydratase
75001	+	+	+	+	+	+			neuronal calcium sensor NCS1. regulates sporulation and confers calcium tolerance
75009	+	+	+	+	+	+			ribonuclease HII
75012	+	+	+	+	+	+			unknown protein
75015									GH27 α-galactosidase
75021	+	+	+	+	+	+			MFS permease
75024	+	+	+	+	+	+			vacuolar ATP synthase 98 kDa subunit
75027	+	+	+	+	+	+	2.399 down	2.514 down	unknown protein
75032	+	+	+	+	+	+			unknown protein
75036	+	+	+	+	+	+	1.618 down	3.168 down	GH63 α-glucosidase
75045	+	+	+	+	+	+			unknown protein
75056									uridine kinase
75063	+	+	+	+	+	+			DNA replication licensing factor mcm6
75069	+	+	+						DNA polymeraseDelta. subunit 3

75072				+	+	+	+	+	+			Snf7 family protein
75074				+	+	+	+	+	+			TRAPP complex component Gsg1
75078												unknown protein
75079				+	+	+	+	+	+			5-phospho-ribosyl-1(alpha)-pyrophosphate synthetase
75102				+	+	+	+	+	+			XPA binding protein 2 (XAB2) involved in pre-mRNA splicing. transcription. and transcripti
75105												unknown protein
75116				+	+	+	+	+	+			Sur4p fatty acid elongase ofSaccharomyces cerevisiae
75128	+	+	+	+	+	+	+	+	+			DEAD/DEAH box helicase. putative
75139				+	+	+	+	+	+			unknown protein
75146				+	+	+	+	+	+			unknown protein
75152				+		+						unknown protein
75156										1.519 down	3.506 up	unknown protein
75159				+	+	+	+	+	+			PepN Aminopeptidase N
75165				+	+	+			+			MFS permease
75169				+	+	+	+	+	+			phosphoserine phosphatase
75175				+	+	+	+	+	+			acyl-CoA dehydrogenase
75184				+	+	+	+	+	+			topisomerase II associated protein (Pat1). putative
75189				+	+	+	+	+	+			Beta-adaptin. apl6. of the AP-3 adaptor protein complex of clathrin-coated vesicles. involv
75195				+	+	+	+	+	+			Bem1p. scaffold protein for complexes that include Cdc24p.Ste5p.Ste20p and Rsr1p
75196				+	+	+	+	+	+			vacuolar protein sorting protein (Vps36). putative
75200				+	+	+	+	+	+			unknown protein
75204												Zn-finger protein AN1
75205												Rad4p
75210				+	+	+	+	+	+			RhoGEF domain-containing protein
75226				+	+	+	+	+	+			dihydroorotate dehydrogenase
75230												unknown protein
75235				+	+	+	+	+	+			unknown protein
75247										1.176 down	2.611 up	AAA+-type ATPase

75258	+	+	+	+	+	+			Peptidase C48. SUMO/Sentrin/Ubl1
75260							1.954 down	2.455 down	RNA polymerase H/23 kD subunit
75262									unknown protein
75271	+	+	+	+	+	+	1.311 down	3.421 up	pyridoxine biosynthesis protein PDX1
75280	+	+	+	+	+	+			unknown protein
75290							4.744 up	2.431 down	dynamin GTPase
75294	+	+	+	+	+	+	1.696 down	2.545 up	BCAT_beta_family
75295	+	+	+	+	+	+	1.935 down	2.580 down	Trehalose-6-phosphate synthase component TPS1 and related subunits
75304	+	+	+	+	+	+			Isopentenyl-diphosphate delta isomerase 2
75311									phospholipase C. related to N. crassa phospholipase C
75316	+	+	+	+	+	+			unknown protein
75324	+	+	+	+	+	+			prolyl-tRNA synthetase. class IIa.
75334	+	+	+	+	+	+			unknown protein
75335	+	+	+	+	+	+			unknown protein
75336	+	+							GT alpha 1.2 mannosyltransferase
75342	+	+	+	+	+	+			unknown protein
75347	+	+	+	+	+	+			Calcium ion pump. ATPase
75361	+	+					1.483 down	4.283 down	unknown protein
75368	+	+	+	+	+	+			3-ketoacyl-CoA thiolase
75380									unknown protein
75383	+	+	+						short chain dehydrogenase/reductase
75385									unknown protein
75394									unknown protein
75397	+	+	+	+	+	+			Vacuolar protein sorting-associated protein Vsp9

75403				+	+	+				1.074 up	2.159 down	CsdB Selenocysteine lyase
75409				+	+	+	+	+	+	1.811 down	2.064 down	Phospholipid-translocating P-type ATPase. flippase; putative
75414												TrpD Anthranilate phosphoribosyltransferase
75418				+	+	+	+	+	+			C2H2 transcriptional regulator
75421				+	+	+	+	+	+			Dolichyl-P-mannose:protein O-mannosyl transferase
75424				+	+							Mitochondrial Matrix Factor
75428				+	+	+	+	+	+			RNA binding protein
75430				+	+	+	+	+	+			Amino acid permease
75434				+	+	+	+	+	+			unknown protein
75440				+	+	+	+	+	+			peroxisomal membrane protein (PmpP24)
75444				+	+	+	+	+	+			signalosome subunit 4 (CsnD)
75447												unknown protein
75450												unknown protein
75468				+	+	+	+	+	+			Cytochrome P-450 lanosterol demethylase; ergosterol biosynthetic pathway
75470										1.594 up	2.271 up	unknown protein
75472				+	+	+	+	+	+			transcriptional regulator. unknown
75475				+	+	+	+	+	+			Sulphate permease
75506				+	+	+	+	+	+			Inosine monophosphate cyclohydrolase
75514				+	+	+	+	+	+			amino acid transporter ARG13
75518				+	+	+						REV1 deoxycytidyl transferase involved in DNA translesion synthesis
75529				+	+	+	+	+	+			deoxyuridine 5'-triphosphate nucleotidohydrolase
75534				+	+	+	+	+	+			RNA-binding protein with KH domain
75535												sphingomyelinase family protein. putative
75547				+	+	+	+	+	+			GTP binding nuclear protein Ran. member of the superfamily of RAS small GTPases. relate
75551				+	+	+	+	+	+			ATP-NAD kinase
75564				+	+	+	+	+	+			Ribosomal protein (60S) L11
75566				+	+	+	+	+	+			unknown protein
75568	+	+	+	+	+	+	+	+	+			thioredoxin. putative

75580	+	+	+	+	+	+			unknown protein
75589	+	+	+	+	+	+			hydroxymethylglutaryl CoA synthase
75593	+	+	+	+	+	+			peroxisomal membrane anchor protein (Pex14). putative
75603	+	+	+	+	+	+			patched sphingolipid transporter
75604	+	+	+	+	+	+			unknown protein
75609	+	+	+	+	+	+	1.178 up	2.180 up	Rpl18 gene encoding 60sRibosomal protein L18.
75620	+	+	+	+	+	+			Bromodomain transcription factor
75646									HNRNP arginine N-methyltransferase
75648									unknown protein
75655	+	+							Component of oligomeric golgi complex Cog8
75662	+	+	+	+	+	+			t-SNARE Sec9; secretory vesicles-plasma membrane
75664	+	+	+	+	+	+			exhibits similarity to ATP-dependent RNA helicases.
75672	+	+	+	+	+	+			NsdC
75686	+	+	+						mitochondrial distribution and morphology protein
75687	+	+	+	+	+	+			eukaryotic translation initiation factor 3 subunit EifCj. putative
75689									Arginyl-tRNA ligase.
75704	+	+	+	+	+	+	1.867 up	2.085 up	Adenosine 5'-phosphosulfate kinase
75707	+	+	+	+	+	+			V-ATPase subunit C
75713	+	+	+	+		+	6.996 down	2.139 down	Cytochrome P450 CYP2 subfamily
75736	+	+	+	+	+	+			pre-mRNA splicing factor. putative
75739	+	+	+	+	+	+			pre-mRNA-splicing factor clf1
75742		+							unknown protein
75753	+	+	+	+	+	+			monothiol glutaredoxin-4
75758	+	+	+	+	+	+	1.187 up	2.014 down	unknown protein
75764	+	+	+	+	+	+			unknown protein
75769	+	+	+	+	+	+			glucose-6-phosphate dehydrogenase
75794	+	+	+	+	+	+			unknown protein
75838	+	+	+	+	+	+			Aldo_ket_redAldo/keto reductase family

75845	+	+	+	+	+	+			NifS Cysteine sulfinate desulfinase/cysteine desulfurase and related enzymes
75854	+		+	+		+			unknown protein
75859	+	+	+	+	+	+			anion-transporting ATPase
75872	+	+	+	+	+	+			MAPKK. MAP kinase kinase. involved in induction of cytokinesis and appressorium format
75878	+	+	+	+	+	+			cAMP dependent protein kinase. protein kinase A. regulatory subunit
75879	+	+	+	+	+	+			unknown protein
75884	+	+	+	+	+	+	3.472 down	7.271 down	unknown protein
75886									Cytochrome P450
75890	+	+	+	+	+	+			C1 tetrahydrofolate synthase. putative
75918	+	+	+	+	+	+			unknown protein
75921	+	+	+	+	+	+			tRNA-dihydrouridine synthase.
75923	+	+	+				2.091 down	3.079 down	unknown protein
75934	+	+	+	+	+	+			E3-binding protein
75935	+	+	+	+	+	+			unknown protein
75937									unknown protein
75939	+	+	+	+	+	+			unknown protein
75949	+	+	+	+	+	+			G-protein gamma subunit
75954	+	+	+	+	+	+			translation initiation factor eIF3 subunit.
75965	+	+	+	+	+	+			Pre-mRNA-splicing factor RSE1
75972	+	+	+	+	+	+			lectin family integral membrane protein. putative
75975	+	+	+	+	+	+			LMBR1 domain-containing protein
75985	+	+	+	+	+		2.407 up	3.846 up	Isochorismatase hydrolase
75989		+							unknown protein
75992									hydroxyethylthiazole kinase
75995	+	+	+	+	+	+	1.266 up	2.204 up	ssDNA binding protein
75998	+	+	+	+	+	+			unknown protein
76009	+	+	+	+	+	+			unknwon zinc finger protein
76010	+	+	+	+	+	+			20S proteasome. alpha subunit Pre6

76013									FAD linked oxidase domain-containing protein
76018	+	+	+						CysKCysteine synthase
76029	+	+	+	+	+	+			unknown protein
76034							2.795 down	2.527 down	Iron/ascorbate family oxidoreductases
76037	+	+	+	+	+	+			unknown protein
76039	+	+	+	+	+	+			Zn2Cys6 transcriptional regulator
76050	+	+	+	+	+	+			diphthamide biosynthesis protein 1
76057	+	+	+	+	+	+			Vacuolar ATP synthase subunit B
76065									unknown protein
76073		+					1.587 up	2.692 up	U6 snRNA-associated Sm-like protein LSm6
76075		+	+				3.472 up	2.873 up	unknown protein
76082	+	+	+	+	+	+			unknown protein
76083	+	+	+	+	+	+			unknown protein
76098	+	+	+	+	+	+			unknown protein
76114	+	+	+	+	+	+			short chain dehydrogenase/reductase
76117	+	+	+	+	+	+			unknown protein
76123	+	+	+	+	+	+			unknown protein
76125	+	+	+	+	+	+			unknown protein
76136	+	+	+				1.097 down	4.994 down	unknown protein
76141									unknown protein. only present in ascomycota
76151							1.358 up	2.492 up	GT31 ß-glycosyltransferase
76155		+	+						acid phosphatase-like protein
76159	+	+	+	+	+	+			NADH-ubiquinone oxidoreductase. chain 49kDa
76166	+	+	+	+	+	+			ATP synthase subunit 4. mitochondrial precursor
76169									NOC4. Involved inNucleolar processing of pre-18S ribosomal RNA. Has a role in theNuclea
76172	+	+	+	+	+	+			ER-derived vesicles protein Erv46
76188	+	+	+	+	+	+			cellular morphogenesis protein. putative
76191	+	+	+	+	+	+			splicing factor 3B subunit 1. putative

76200	+	+	+		+		1.138 up	2.411 up	unknown protein
76204									NADH:flavin oxidoreductase/12-oxophytodienoate reductase
76210							14.836 down	10.988 up	GH62 α-L-arabinofuranosidase ABF2
76215									sulfide:quinone oxidoreductase/flavo-binding protein
76218									zinc containing alcohol dehydrogenase superfamily
76220							1.205 down	2.030 down	Zn2Cys6 transcriptional regulator
76227									GH3 β-glucosidase CEL3e
76230									Flavin-containing monooxygenase
76235									MFS permease
76238	+	+	+	+	+	+	38.819 down	5.536 down	plasma membrane H+ ATPase
76247									O-methyltransferase family protein
76249	+	+	+	+	+	+			unknown protein
76250		+	+						unknown protein
76266	+	+	+	+	+	+			GH16 cell wall glucanosyltransferase
76269									unknown protein
76288	+	+	+	+			1.806 down	2.610 down	short chain dehydrogenase/reductase
76291	+	+	+	+	+	+			eRF1 (translation factor pelota).
76311	+	+	+	+	+	+			sodium/hydrogen antiporter
76324	+	+	+	+	+	+			acetoacetyl-CoA synthase
76329	+	+	+	+	+	+			Ran-specific GTPase-activating protein 1. putative
76330									unknown protein
76336	+	+	+	+	+	+			GTPase FZO1. transmembrane location
76340	+	+	+	+	+	+			unknown protein
76359									unknown protein. only present in ascomycota
76360							1.105 down	5.019 up	unknown protein. 1 TM domain
76363									Serine dehydratase

76366							29.853 down	3.451 down	NADH:flavin oxidoreductase/12-oxophytodienoate reductase
76374									AAA ATPase
76381		+	+						urate oxidase
76393	+	+	+	+	+	+			chaperone protein dnaJ 2
76421							1.382 down	2.333 down	aromatic ring-opening dioxygenase LigB subunit. putative
76451									C2H2 transcriptional regulator
76453	+	+	+		+		1.175 up	2.179 up	MDR-type ABC transporters
76481	+	+	+	+	+	+			Pre-mRNA-splicing ATP-dependent RNA helicase PRP28
76491	+	+	+	+	+	+			Regulator of Rac1. required for phagocytosis and cell migration
76502	+	+	+	+	+	+			unknown protein
76505	+	+	+	+	+	+			C2H2 transcriptional regulator
76514	+	+	+	+	+	+			Eukaryotic-like DNA topoisomerase I
76515	+	+	+	+	+	+			Vacuolar protein sorting-associated protein Vps33
76522	+	+	+	+	+	+			Calcium/calmodulin-dependent protein kinase
76532	+	+	+	+	+	+	1.535 up	6.009 up	ribosomal protein (60S) P0
76539	+	+	+						unknown protein
76540	+	+	+	+	+	+			C2 domain protein (=Ca2+-dependent membrane-targeting module)
76543	+	+	+	+	+	+	1.052 down	2.013 down	ARF GTPase activator (Csx2). putative
76551	+	+	+	+	+	+			uridylate kinase
76557	+	+	+	+		+			Calcium transporter
76564	+	+	+	+	+	+			FSO1 protein
76571	+	+	+	+	+	+			unknown protein
76590	+	+	+						Zn2Cys6 transcriptional regulator PRO1. involved in fruiting body development
76601							100.220 down	3.183 down	Sulfite oxidase. molybdopterin-binding component
76617	+	+	+	+		+			FAD-binding dehydrogenase
76620	+		+				2.041 down	2.566 down	Glycerol-3-phosphate dehydrogenase

76633		+					1.083 up	4.622 down	MDR multidrug transporter
76641									MFS permease
76646	+	+	+	+	+	+			unknown protein
76652									flavin-binding monooxygenase. putative
76658	+	+	+	+	+	+			unknown protein
76659							1.222 up	5.631 up	unknown protein
76672							1.287 down	27.778 up	GH3 β-glucosidase BGL1/CEL3a
76677		+					1.132 down	2.490 down	Zn2Cys6 transcriptional regulator
76682							1.259 up	4.843 up	PDR-type ABC transporters
76690									unknown protein
76696			+						Flavin-containing monooxygenase
76700	+	+	+	+	+	+			GH17 glucan 1.3 β-glucosidase/ glucan endo-1.3-β-glucosidase
76701	+	+	+	+	+	+			peroxisomal membrane protein pex16
76705	+	+	+	+	+	+	1.082 up	2.325 down	Zn2Cys6 transcriptional regulator
76706	+	+	+	+	+	+			fungal specific transcription factor domain-containing protein
76710	+	+	+	+	+	+			DNA-directed RNA polymerase III subunit RPC1
76713							1.567 down	3.232 down	unknown protein
76722		+	+						flavohemoglobin
76734	+	+	+						unknown SWIRM domain protein
76740	+	+	+	+	+	+	1.492 up	3.307 up	zuotin
76744	+	+	+	+	+	+			acetyl-transferring pyruvate dehydrogenase. E1 component beta subunit
76752	+	+	+	+	+	+			unknown protein
76758									maltose permease
76763									PTH11 GPCR
76766	+	+	+	+	+	+	1.542 down	2.240 down	xanthine/uracil permease

76771	+	+	+	+	+	+			unknown protein
76775			+						MFS permease
76784	+	+	+						sorbitol dehydrogenase
76795	+	+	+	+	+	+			pre-mRNA splicing factor. putative
76800									MFS permease
76817	+	+	+	+	+	+			GATA type transcriptional regulator
76820	+	+	+	+		+			unknown protein
76822							6.287 up	2.302 down	unknown protein
76828	+	+	+	+	+	+	1.334 up	2.039 up	exosome complex exonuclease RRP40. putative
76833	+	+	+	+	+	+			rRNA assembly protein Mis3
76845	+	+	+	+	+	+			unknown protein
76852									GH2 β-galactosidase/β-glucuronidase
76859	+	+	+	+	+	+			Importin ß KapD
76862	+	+	+	+	+	+			palmitoyltransferase PFA5. putative
76872	+	+	+	+	+	+			Zn2Cys6 transcriptional regulator
76875	+	+	+	+	+	+			unknown protein
76880			+						GTPase Rsr1 (A. fumigatus)
76887	+	+	+	+	+	+			aspartyl protease
76897	+	+	+	+	+	+	1.363 down	2.796 down	MFS permease
76905	+	+	+	+	+	+			unknown protein
76906	+	+	+	+	+	+			unknown protein
76910							2.344 down	2.174 down	monocarboxylate transporter
76919	+	+	+	+	+	+			calcium-spray protein
76927	+	+	+	+	+	+			translation initiation factor SUI1.
76930	+	+	+	+	+	+			complex I intermediate-associated protein 30
76939	+	+	+	+	+	+			Ribosomal protein S9. S4 family
76949	+	+	+			+			unknown protein

76954									Alkaline phosphatase
76960	+	+	+	+	+	+			unknown protein
76971	+	+	+	+		+			SSCRP
76986		+					1.119 down	2.765 down	unknown protein
76992									MFS permease
76995									unknown protein
77000	+	+	+	+	+	+			Haloacid dehalogenase-like protein
77007	+	+	+	+	+	+			pre-mRNA-splicing factor CWC2
77009		+							GTP binding protein (Gtp1). putative
77014	+	+	+	+	+	+			Guanosine diphosphatase. transport of GDP-mannose into the Golgi lumen
77025	+	+	+	+	+	+			P-type ATPase with putative aminophospholipid translocase activity. neo1
77031	+	+	+	+	+	+			Rho-GTPase effector BNI1 and related formins
77038	+	+	+	+	+	+			R-SNARE Sec22; anterograde/retrograde transport ER-Golgi
77056	+	+	+	+	+	+			vacuolar protein sorting 55
77084	+	+	+	+					cell wall biogenesis protein phosphatase Ssd1. putative
77086	+	+	+						ATP dependent DNA ligase domain-containing protein
77093			+						acid sphingomyelin phosphodiesterase (probably vacuolar)
77098	+	+	+	+	+	+			unknown protein
77099	+	+	+	+	+	+			unknown protein
77119	+	+	+	+	+	+			ornithine decarboxylase
77122	+	+	+	+	+	+			mitochondrial presequence protease CYM1
77135	+	+	+	+	+	+			Protein phosphatase 2A. regulatory B subunit. B56
77138	+	+	+	+	+	+	1.151 up	2.395 up	unknown protein
77142	+	+	+	+	+	+			TATA-binding protein interacting (TIP20) . putative
77154	+	+	+	+	+	+			homeobox transcriptional regulator
77167	+	+	+	+	+	+	1.011 down	3.488 down	unknown protein
77188	+	+	+	+	+	+			60S ribosomal protein L1
77191	+	+	+			+			BZIP transcriptional regulator

77193	+	+	+	+	+	+			unknown protein
77202							6.662 down	4.546 down	short chain dehydrogenase/reductase
77209	+	+	+	+	+	+			splicing factor 3a
77215	+	+	+	+	+	+			unknown protein
77218	+	+	+	+	+	+			outer membrane protein TOM13
77222	+	+	+	+	+	+			unknown protein
77227	+	+	+	+	+	+			UDP-glucose ceramide glucosyltransferase
77234	+	+	+	+	+	+			unknown protein
77242	+	+	+						glutamate N-acetyltransferase precursor
77251	+	+	+	+	+	+			histone acetylase complex subunit Paf400. putative
77254									unknown protein
77266									Thymidylate kinase
77279	+	+	+	+	+	+			SNF4
77281	+	+	+	+	+	+			importin α KapA
77283	+	+	+	+	+	+			GT2 glycosyltransferase
77284	+	+	+	+	+	+			GH12 endo-ß-1.4-glucanase
77288									Thioredoxin reductase
77291							1.359 up	2.532 down	Zn2Cys6 transcriptional regulator
77299							3.682 down	2.262 up	GH2 Exo-β-D-glucosaminidase GLS93
77309	+	+	+	+	+	+			S-adenosylmethionine decarboxylase
77311	+	+	+	+	+	+			unknown protein
77330	+	+	+	+	+	+			26S proteasome regulatory complex subunit Rpn9
77336	+	+	+	+	+	+			aconitate hydratase
77339	+	+	+	+	+	+			unknown protein
77353	+	+	+	+	+	+			NADH-ubiquinone oxidoreductase 40 kDa subunit-like protein
77364	+	+	+	+	+	+			unknown protein
77373	+	+	+	+	+	+			dihydrolipoyl dehydrogenase (E3 component of both 2-oxoglutarate dehydrogenase and p

77380		+	+						RNA binding protein
77404	+	+	+	+	+	+			importin ß KapI
77412	+	+	+	+	+	+			unknown protein
77423							2.978 down	5.195 down	Jacalin-like lectin
77425	+	+	+	+	+	+			unknown protein
77440	+	+	+	+	+	+			unknown protein
77441	+	+	+	+	+	+			Vacuolar sorting protein Vps74
77447	+	+	+	+	+	+			unknown protein
77456	+	+	+	+	+	+			unknown protein
77466	+	+	+	+	+	+			unknown protein
77473									DNA photolyase. N-terminal. class 1. FAD-binding
77476	+	+	+	+	+	+			unknown protein
77478	+	+	+	+	+	+			kinesin-like protein
77481	+	+	+						D-xylulose 5-phosphate/D-fructose 6-phosphate phosphoketolase
77485	+	+	+	+	+	+			T-complex protein 1
77495	+	+	+	+	+	+	1.182 up	2.172 up	eukaryotic translation initiation factor 3 subunit 11.
77505	+	+	+	+	+	+			unknown protein
77506	+	+	+	+	+	+	1.173 down	2.078 down	GH5 β-glycosidase CEL5d
77512									cytochrome P450
77513	+	+	+						Zn2Cys6 transcriptional regulator
77517	+	+	+	+	+	+	1.129 up	3.423 up	MFS hexose transporter
77539	+	+	+	+	+	+			glucosidase 2 subunit beta
77541	+	+	+	+	+	+			actin-like protein
77547							2.598 down	6.449 down	GT1 glycosyltransferase
77552									Inorganic phosphate transporter
77557	+		+			+	7.316 down	5.660 down	GT glycosyltransferases not yet assigned to a family. 3 TMs
77570	+	+	+	+	+	+			Vesicle coat complex COPII. Sfb3

77577	+	+	+	+	+	+			unknown protein
77579	+	+	+	+	+	+			aspartyl protease
77583	+	+	+	+	+	+			SEC14. phosphatidylinositol/phosphatidylcholine transfer protein
77587	+	+	+	+	+	+			19S regulatory particle ATPase Rpt3
77591	+	+	+	+	+	+			26S proteasome regulatory complex subunit Rpn3
77593		+					1.259 down	3.284 down	unknown protein
77602	+	+	+	+	+	+	1.503 down	2.212 down	A.a-trehalose-phosphate synthase
77609	+	+	+	+	+	+			AT-rich interaction region
77613	+	+	+	+	+	+			eukaryotic translation initiation factor 3 subunit E
77616									aminoacyl-tRNA synthetase and proteasome regulatory subunit.
77629	+	+	+	+	+	+	1.847 up	2.756 up	unknown protein
77634	+	+	+	+	+	+			N-acetylglucosaminyl transferase component Gpi1
77642	+	+	+	+	+	+			nuclear migration protein NudF
77647	+	+	+	+	+	+			threonine aldolase
77656	+	+	+	+	+	+			phosphoglycerate mutase
77661	+	+	+	+	+	+			ounknown proteinsterol binding protein
77667	+	+	+	+	+	+			RING-5
77672	+	+	+	+	+	+			effector of Sec4; Sro7 / Sro77
77685	+	+	+	+	+	+			SAGA complex bromodomain subunit Spt7. putative
77689	+	+	+	+	+	+			PTPc. Protein tyrosine phosphatases (PTP)
77699									unknown protein
77703			+						PDR-type ABC transporters
77706	+	+	+	+	+	+			unknown protein
77713	+	+	+	+	+	+			unknown protein
77714		+							biotin-protein ligase
77732	+	+	+	+	+	+			Ubiquitin conjugating enzyme Ubc6
77736	+	+	+	+	+	+			unknown protein
77739	+	+	+	+	+	+			HgmAhomogentisate .-dioxygenase

77743							1.852 up	3.027 down	unknown protein
77744	+	+	+	+	+	+			unknown protein
77747	+	+	+	+	+	+			RNA-binding protein Vip1
77749	+	+	+	+	+	+			fumarylacetoacetase
77758	+	+	+	+	+	+			cytochrome c heme lyase. putative
77764	+	+	+	+	+	+			histidine kinase PHY1p. phytochrome-like
77767	+	+	+	+		+			Taurine catabolism dioxygenase TauD. putative
77770									Zinc-containing alcohol dehydrogenase
77780		+	+						unknown protein
77785									MFS permease
77795	+	+	+	+	+	+			LIMPET. E3-ubiquitin ligase SCON-2
77806	+	+	+						Alcohol dehydrogenase. class V
77811	+	+	+	+	+	+			AMP-dependent synthetase and ligase
77829	+	+	+	+	+	+			unknown protein
77836									unknown protein
77848	+	+	+	+	+	+			unknown protein
77850	+	+	+	+	+	+			unknown protein
77870	+	+	+	+	+	+			peroxin 8. putative
77878							1.096 down	3.446 up	C2H2 transcriptional regulator
77887	+	+							unknown protein
77906	+	+	+	+	+	+			unknown protein
77915	+	+	+	+	+	+	1.061 up	2.019 up	unknown protein
77932	+	+	+	+	+	+	1.446 up	3.282 up	Ribosomal protein L30.
77942	+	+	+						GH72 β-1 3-glucanosyltransferase
77955	+	+	+	+	+	+			cytochrome b5-like Heme/Steroid binding domain-containing protein
77969	+	+	+						chromate transporter
77974	+	+	+	+	+	+			half-sized ABC transporter
77979	+	+	+	+	+	+	1.525 up	2.412 up	SmallSubunit. aps3. of the adaptor protein complex AP-3 of clathrin-coated vesicles; invol

77989	+	+	+	+	+	+			Csh7. an ER membrane chaperone protein
77991	+	+	+	+	+	+			RNA polymerase TFIIH complex subunit Ssl1. putative
77998	+	+	+						unknown protein
78004	+	+	+	+	+	+			triose phosphate/3-phosphoglycerate/phosphate translocator
78010	+	+	+	+	+	+	1.096 down	2.423 down	ankyrin containing protein
78012	+	+	+	+	+	+	1.359 down	2.109 down	Sensory transduction histidine kinase
78027	+	+	+	+	+	+			unknown protein. only in fungi
78037							1.220 down	3.040 down	unknown protein
78042	+	+	+	+	+	+	1.091 down	2.296 down	unknown protein
78044	+	+	+	+	+	+			unknown protein
78046	+	+	+	+	+	+			eukaryotic translation initiation factor 3
78049									elongation factor Tu (G).
78054									Vacuolar protein sorting-associated protein Vps16
78061	+	+	+	+	+	+			unknown protein
78062	+	+	+	+	+	+			rRNA biogenesis protein RRP5
78072									Amidase
78095	+	+	+						unknown protein
78109	+	+	+	+	+	+			pre-mRNA processing splicing factor 8
78117	+	+	+	+	+	+			unknown protein
78130	+	+	+	+	+	+			C-5 sterol desaturase
78158	+	+	+	+	+	+			importin ß KapL
78162	+	+	+	+	+	+			unknown protein
78176	+	+	+	+	+	+			ß-1.3-glucan synthase
78189	+	+	+	+	+	+			Component of oligomeric golgi complex Cog5
78191	+	+	+	+		+			unknown protein
78223									Cytochrome P450 CYP3/CYP5/CYP6/CYP9 subfamilies
78226			+						unknown protein

78233	+	+	+	+	+	+			40s ribosomal protein S21 based on homolgoy to the corresponding protein in Giberella z
78242	+	+	+	+	+	+			unknown protein
78248									short chain dehydrogenase/reductase
78264	+	+	+	+	+	+	1.611 up	2.211 up	Peptide methionine sulfoxide reductase
78268	+	+	+	+	+	+			unknown protein
78274	+	+	+	+	+	+	1.231 down	2.084 up	phosphatidylinositol-3-phosphate 5-kinase; fab1
78288	+	+	+	+	+	+			unknown protein
78301	+	+	+	+	+	+	1.228 down	2.251 down	cation diffusion facilitator family transporter
78306	+	+	+	+	+	+			thiosulfate sulfurtransferase
78309									unknown protein
78314	+	+	+	+	+	+			RgsB. regulator of G-protein signaling
78320									Sulfatase
78322	+	+	+	+	+	+			unknown protein
78340									unknown protein
78343	+	+	+	+	+	+			unknown protein
78347		+							3-deoxy-7-phosphoheptulonate synthase
78357	+	+	+	+	+	+	1.122 down	2.796 down	NADH-quinone oxidoreductase. putative
78373	+	+	+	+	+	+			half-sized ABC transporter
78377	+	+	+	+	+	+			GTP-binding protein. HSR1-related. member ofGTP1/OBG family
78387									Cytochrome P450 CYP3/CYP5/CYP6/CYP9 subfamilies
78391							1.462 down	4.460 down	unknown protein
78401	+	+	+	+	+	+			unknown protein
78409	+	+	+	+	+	+			ThrC Threonine synthase
78415	+	+	+	+	+	+			unknown protein
78421	+	+	+	+	+	+			unknown protein
78423	+	+	+	+	+	+			26S proteasome regulatory complex subunit Rpn2
78437	+	+	+	+	+	+			unknown protein

78439	+	+	+	+	+	+			pyruvate kinase
78445									Zn2Cys6 transcriptional regulator
78448									unknown protein
78462									L-carnitine dehydratase/alpha-methylacyl-CoA racemase
78463									unknown protein
78465							1.324 up	2.856 up	siderophore transporter
78476									unknown protein
78482	+	+	+	+	+	+			acyl carrier protein precursor. mitochondrial [NADH-ubiquinone oxidoreductase subunit]
78496			+						Flavin-containing monooxygenase Fmo1 like
78499			+						PTH11 GPCR
78542									cytosolic phospholipase A2. putative
78543	+	+	+	+	+	+			RNA-binding La domain protein
78552							1.082 down	2.510 down	Aromatic L-amino acid decarbounknown proteinlase
78555	+	+	+	+	+	+			Esterase of the alpha-beta hydrolase superfamily (Neuropathy targetEsterase). contains c
78560	+	+	+	+	+	+			unknown protein
78561		+							Molybdenum cofactor biosynthesis protein
78576		+							unknown protein
78582	+	+	+	+	+	+			ferrochelatase
78585							39.746 down	4.811 down	MFS permease
78591	+	+	+	+	+	+			fatty acid synthase beta subunit [Includes: 3-hydroxypalmitoyl-[acyl-carrier-protein] dehy
78611	+	+					1.873 down	4.125 down	phospholipase D
78626	+	+	+	+	+	+			unknown protein
78639		+	+				4.778 up	2.880 up	Serine/threonine protein kinase
78641	+	+	+	+	+	+			F-box and WD domain-containing protein
78645	+	+	+	+	+	+			unknown protein. 2 TM
78650	+	+	+	+	+	+			C2H2 transcriptional regulator
78661	+	+	+	+	+	+			ribosomal protein S5. MRPS5.

78679				+	+	+	+	+	+	1.345 up	2.057 up	mitochondrial carrier protein
78681				+	+	+	+	+	+			SSCRP
78683				+	+	+	+	+	+			aldehyde dehydrogenase
78685				+	+	+						unknown protein
78688				+	+	+	+	+	+	1.703 down	2.746 down	Winged helix repressor DNA-binding
78690				+	+	+	+	+	+			unknown protein
78693												tRNA intron endonuclease. N-terminal.
78695				+	+	+	+	+	+			unknown protein
78706				+	+	+	+	+	+			unknown protein
78713										3.003 down	4.203 down	GH72 ß-(1-3) glucanosyltransferase
78725												unknown protein
78726				+	+	+	+	+	+			DNA-directed RNA polymerase. 30-40 kDa subunit
78732				+	+	+	+	+	+			6-phosphofructo-2-kinase
78738				+	+	+	+	+	+			sodium/hydrogen exchanger family protein
78740				+	+	+	+	+	+	1.037 up	2.234 up	v-SNARE Bet1. ER-Golgi complex
78753				+	+	+	+	+	+			Nucleoporin interacting component
78757				+	+	+	+	+	+			plasma membrane H+ ATPase
78765				+	+	+	+	+	+			mitochondrial DNA replication protein (Yhm2). putative
78768				+	+	+	+	+	+			unknown protein
78773	+	+	+	+	+	+	+	+	+	1.069 down	2.061 down	long-chain-fatty-acid--CoA ligase 2
78783				+	+	+	+	+	+			kinetochore protein SPC25
78792										1.875 down	4.111 down	unknown protein
78797										1.085 down	3.513 down	xanthine dehydrogenase
78799				+	+	+	+	+		1.076 down	3.434 down	Zn2Cys6 transcriptional regulator
78806				+	+	+	+	+	+			Ubiquitin chain assembly factor (E4)

78811	+	+	+	+	+	+	1.201 down	2.151 down	unknown protein
78817	+	+	+	+	+	+			19S regulatory particle ATPase Rpt6
78823	+	+	+	+	+	+			unknown protein
78828		+					2.840 up	2.953 up	carboxylesterase type B
78833									MFS permease (fucose permease)
78836	+	+	+	+	+	+			mitochondrial phosphate carrier
78843									unknown protein
78852	+	+	+	+	+	+			Esterase/lipase/thioesterase
78864							1.190 up	2.701 up	GcvTGlycine cleavage system T protein
78866	+	+	+	+	+	+	1.009 up	2.054 down	unknown protein
78870	+	+	+	+	+	+			unknown protein
78873	+	+	+	+	+	+			unknown protein
78882	+	+	+	+	+	+			20S proteasome beta-type subunit Pre7
78886	+	+	+	+	+	+			unknown protein
78895	+	+	+	+	+	+	1.006 up	2.724 down	Zn2Cys6 transcriptional regulator
78909	+	+	+	+	+	+			serine/threonine protein kinase cot1
78925	+	+	+	+	+	+			20S proteasome beta-type subunit Pre4
78929	+	+	+	+	+	+			Beta_HSD-beta hydroxysteroid dehydrogenase/isomerase family
78935									ubiquitin-conjugating enzyme
78939	+	+	+	+	+	+			pre-mRNA splicing helicase
78953	+	+	+	+	+	+			unknown protein with CORD and CS domain
78956									unknown protein
78958	+	+	+	+	+	+			Autophagy-related protein 22
78960	+	+	+	+	+	+			unknown protein
78961	+	+	+	+	+	+			F-actin capping protein. beta subunit domain
78966	+	+	+	+	+	+			unknown protein
78968	+	+	+	+	+	+			unknown protein

78970	+	+	+	+	+	+	2.472 down	4.870 down	mitochondrial (phosphate) carrier
78984	+	+	+	+	+	+	1.304 down	2.108 down	unknown protein
78988	+	+	+		+	+			Gß-WD40 domain protein
79010	+	+	+						lipase. putative
79014	+	+	+	+	+	+			Vacuolar ATP synthase 16 kDa proteolipid subunit
79019	+	+	+	+	+	+			Phospholipase D
79031	+	+	+	+	+	+			GTP-binding protein
79034	+	+	+	+	+	+	1.463 up	2.784 up	ABC1 family protein
79037	+	+	+	+	+	+			asparagine synthase
79044		+							GH47 α-1.2-mannosidase
79059	+	+	+	+	+	+	1.057 up	2.079 up	Myo-inositol-1-phosphate synthase. associated to cellulase signal transduction (PMID: 15
79075	+	+	+	+	+	+			unknown protein of ORM1 family
79085	+	+	+	+	+	+			unknown protein
79089	+	+	+	+	+	+			unknown protein
79104	+	+	+	+	+	+			Epsilon subunit of the coatomer complex. sec28
79106	+	+	+	+	+	+	1.328 up	2.075 up	40s ribosomal protein L44e by homology to the corresponding protein of several yeasts ar
79109	+	+	+	+	+	+			unknown protein
79111									unknown protein
79116									MFS permease
79119	+	+	+	+	+	+			unknown protein
79124	+	+	+	+	+	+			kinesin-like protein. a kinesin-3 family member.
79129	+	+	+						unknown protein
79153							1.159 down	2.901 down	HyuA. N-methylhydantoinase A/acetone carboxylase. beta subunit
79166	+	+	+	+	+	+			SCP-2 sterol transfer family protein
79169	+	+	+	+	+	+			K+ channel protein
79171	+	+	+	+	+	+			Casein kinase 1Cki1. regulator of phosphatidylinositol 4-phosphate 5-kinase
79174	+	+	+	+	+	+			Emp24/gp25L/p24 family protein Erp1

79187	+	+	+	+	+	+	2.070 up	2.371 up	DNA polymerase alpha-primase complex. polymerase-associated subunit B
79202		+	+				2.388 down	3.461 up	MFS permease. associated with cellulose signalling
79222							13.373 down	4.677 down	unknown protein
79225	+	+	+	+	+	+			DNA-directed RNA polymerase. beta subunit
79229	+	+	+	+	+	+	1.497 up	2.508 up	elongation factor G (Tu) and the LepA membrane protein family.
79235	+		+	+					unknown protein
79237	+	+	+				1.407 down	3.081 down	unknown protein
79238									unknown protein
79244		+	+						unknown protein
79258	+	+	+	+	+	+			phospholipid-translocating P-type ATPase
79259	+	+	+	+					unknown protein
79262	+	+	+	+	+	+			ATP synthase subunit E. putative
79271	+	+	+	+		+	1.162 down	3.028 down	malate synthase
79287	+	+	+	+	+	+			unknown protein
79295	+	+	+	+	+	+			ER membrane proteins involved in ER exit of secreted proteins.
79300	+	+	+	+	+	+			unknown protein
79304	+	+	+						DNA repair endonuclease Rad2p of Saccharomyces cerevisiae and ERCC5 (XPG) of Homo s
79315	+	+	+	+	+	+			RNA polymerase Rpb1
79324	+	+	+	+	+	+			nucleoside-diphosphate-sugar epimerase. putative (check!)
79326	+	+	+	+	+	+			unknown protein
79329	+	+	+						MFS permease
79334	+	+	+	+	+	+			NADH-cytochrome b5 reductase. putative
79344	+	+	+	+	+	+			unknown protein
79345							3.286 up	3.544 up	unknown protein. Mpv17/PMP22 family
79359	+	+	+	+	+	+			unknown protein
79361		+	+				2.257 up	2.408 up	unknown protein
79373	+	+	+	+	+	+			adenine phosphoribosyltransferase

79377	+	+	+	+	+	+			Sin3 complex subunit Stb2 based on homology to the corresponding proteins of Aspergillu
79381	+	+	+	+	+	+			RMD5. regulator of gluconeogenesis
79395	+	+	+	+	+	+			unknown protein
79396	+	+	+	+	+	+	2.453 down	2.093 down	GT2 ß-glycosyltransferase. related to hyaluronan synthases
79398	+	+	+	+	+	+			Ca2+ transporter
79405	+	+	+	+	+	+			HET-containing protein. unknown
79414	+	+	+	+	+	+			transcriptional regulator NGG1. histone acetyltransferase subunit-3 like
79419	+	+	+	+	+	+			Polyubiquitin-binding protein Dsk2
79426							1.416 up	2.139 up	unknown protein
79430									unknown protein
79439	+	+	+	+	+	+			acetyl-CoA acetyltransferase
79441	+	+	+	+	+	+			histone deacetylase complex subunit
79448	+	+	+	+	+	+			HFBs
79459	+	+	+	+	+	+			Myristoyl-CoA:protein N-myristoyltransferase (nmt)
79461	+	+	+	+	+	+	1.603 down	2.087 down	unknown protein
79464	+	+	+	+	+	+			unknown protein
79466	+	+	+	+	+	+			unknown protein
79485	+	+	+	+	+	+			peptidase (PNG1)
79487	+	+	+	+	+	+	1.260 up	2.392 up	Mannose-P-dolichol utilization defect 1 protein
79490	+	+	+	+	+	+			unknown protein
79495	+	+	+	+	+	+			Isopropylmalate/homocitrate/citramalate synthases
79498		+	+						NoxA
79501	+	+	+						unknown protein
79503	+	+	+	+	+	+			casein kinase II. alpha subunit
79510	+	+	+	+	+	+			MRP-type ABC transporter
79535	+	+	+	+	+	+			serine/threonine protein phosphatase
79545	+	+	+	+	+	+			Dioxygenases related to -nitropropane dioxygenase
79560	+	+	+	+	+	+			unknown protein

79565	+	+	+	+	+	+	1.870 up	2.302 up	Thioredoxin reductase TrxB
79568	+	+	+	+	+	+			UDP-N-acetylglucosamine pyrophosphorylase
79598	+	+	+	+	+	+			unknown protein
79599	+	+	+	+	+	+			Ca2+ transporter
79602							1.010 up	4.513 down	GH81 endo-1.3-β-glucanase
79606							1.639 down	2.177 down	GH115 xylan-α-1.2-glucuronidase or α-(4-O-methyl)-glucuronidase
79619	+	+	+	+	+	+			unknown protein
79623	+	+	+	+	+	+			pre-rRNA-processing protein TSR2 . putative
79627	+	+	+	+	+	+			unknown protein
79644	+	+	+				1.424 down	2.247 up	metal ion transporter SMF2
79661							1.018 up	2.319 down	unknown protein
79669									GH3 β-N-acetylglucosaminidase
79671									N-acetyl-glucosamine-6-phosphate deacetylase
79673	+	+	+	+	+	+			PhoG
79677									unknown protein
79686	+	+	+	+	+	+			Unknown protein with RNA binding domains
79690	+	+	+	+	+	+			Electron transfer flavoprotein-ubiquinone oxidoreductase
79694	+	+	+	+	+	+			fructosyl amino acid oxidases
79708		+							unknown protein
79725							1.094 down	3.115 down	Zn2Cys6 transcriptional regulator
79726							7.847 up	9.055 up	unknown protein
79737									Maleylacetoacetate isomerase. putative
79738									Zinc-binding oxidoreductase
79741	+	+	+	+		+	1.807 down	2.212 up	ER-bound Farnesyl-diphosphate farnesyltransferase
79746	+	+	+	+	+	+			has high homology to microtubule associated proteins.

79749		+	+	+	+	+	+			G-patch domain-containing protein
79756		+	+	+	+	+	+	1.166 up	2.416 down	F-Box/WD40 repeat protein FWD-1. targets the clock component FRQ for degradation
79761				+	+					unknown protein
79769		+	+	+	+	+	+			Cytochrome c and c1 heme-lyase. shares homology to Protein prenyltransferase. alpha su
79774		+	+	+	+	+	+			unknown protein
79779								1.626 up	5.436 up	unknown protein
79782		+	+	+	+	+	+			BioF -keto--aminopelargonate synthetase and related enzymes
79797		+	+	+	+	+	+			unknown protein
79807		+	+	+	+	+	+	1.096 down	2.083 down	Aspartictype endopeptidase ctsD
79813										flavoprotein monooxygenases
79816								1.212 down	3.680 up	unknown protein; secreted
79817		+	+	+	+	+	+			unknown protein
79823		+						1.320 down	2.100 down	DCL2. Dicer-like protein. involved in quelling
79825		+	+	+	+	+	+			20S proteasome beta-type subunit Pre8
79828		+	+	+	+	+	+			unknown protein. 3 TM
79832		+	+	+	+	+	+			GT α-1.6-mannosyltransferase
79835		+	+	+	+	+	+			GadB Glutamate decarboxylase and related PLP-dependent proteins
79843										hsp70 protein
79850	+	+	+	+	+	+	+			Phosphotyrosyl phosphatase activator. PTPA
79854		+	+	+	+	+	+			CCR4-NOT core complex subunit Not4
79871		+	+	+	+	+	+			Zn2Cys6 transcriptional regulator
79884		+	+	+	+	+	+			Neddylin (ubiquitin-like protein).
79891		+	+	+	+	+	+			Vesicle coat complex COPII. Sec13
79895		+	+	+	+	+	+			serine/threonine protein kinase kin1 involved in cellular morphogenesis
79919		+	+	+	+	+	+			unknown protein
79921			+	+						GH92 α-1.2-mannosidase
79931		+	+	+						unknown protein

79933	+	+	+	+	+	+	1.273 up	3.151 up	mitochondrial ribosomal protein MRPL6.
79946	+	+	+	+	+	+			multisynthetase complex auxiliary component p43
79947	+	+	+	+	+	+	1.257 down	2.543 up	DNA-dependent RNA polymerase
79955	+	+	+	+	+	+			UDP glucose-4-epimerase
79960			+	+		+	3.156 up	2.894 up	GH47 α-1.2-mannosidase
79972	+	+	+	+	+	+			unknown protein
79974	+	+	+	+	+	+			Palmitoyltransferase ERF2
79990									unknown protein
79993	+	+	+	+	+	+			T-complex protein 1
80003	+	+	+						Phosphoglycerate dehydrogenase and related dehydrogenases
80005	+	+	+	+	+	+			unknown protein
80007	+	+	+	+	+	+			Like Rad54; interacts with Dmc1
80019							1.739 up	2.366 up	short-chain dehydrogenase/reductase
80022	+	+	+	+	+	+			unknown protein
80025	+	+	+	+	+	+			ribosomal protein S17e. and it shares extensive amino acid sequence identity with ribosor
80026			+				2.194 up	3.143 up	MFS permease
80028									MRP-type ABC transporter
80034							1.226 up	2.088 down	DadA Glycine/D-amino acid oxidases (deaminating) [Amino acid transport and metabolism
80042	+	+	+	+	+	+			unknown protein. 1 TM
80048	+	+	+	+	+	+			phosducin like protein class II
80052	+	+	+	+		+			unknown protein
80056							2.072 up	3.390 up	RNA 3'-terminal phosphate cyclase . putative
80058							11.514 down	4.257 down	MFS permease
80073	+	+	+	+	+	+			unknown protein
80086			+						MFS peptide transporter
80091									quinate permease
80096	+	+	+	+			1.083 down	2.464 down	unknown protein

80105							1.011 up	2.166 down	unknown protein
80115									unknown protein
80122									unknown protein
80125	+	+	+	+	+	+			GPCR. related to N. crassa Stm1-like GPR-5
80137							1.188 down	2.247 down	unknown protein
80139									Zn2Cys6 transcriptional regulator
80142	+	+	+	+	+	+			HSP104 and related ATP-dependent Clp proteases
80149	+	+	+						unknown protein.
80151									PheA Prephenate dehydratase
80159	+	+	+	+	+	+			DNA-directed DNA polymerase B
80167	+	+	+	+	+	+	1.362 up	2.118 up	histone H4 variant
80168	+	+	+	+	+	+			unknown protein
80181	+	+	+	+	+	+			unknown protein
80186	+	+	+	+	+	+			MAPKK. hypothetical MAP kinase kinase Pbs2p
80187	+	+	+	+	+	+			unknown protein. 8TM (auxin efflux carrier)
80191	+	+	+	+	+	+			steryl ester lipase TPL1
80200	+	+	+	+	+	+			transcription factor (Snd1/p100)
80206	+	+	+	+	+	+			unknown protein
80211	+	+	+	+	+	+	1.450 up	6.721 up	ribosomal protein L14.
80216	+	+	+	+	+	+			ATP synthase D chain. mitochondrial
80231	+	+	+	+	+	+			glucokinase
80234	+	+	+	+	+	+			unknown protein
80237									intermembrane peptidase
80240									GH35 β-galactosidase BGA1
80243	+	+	+	+	+	+			SRP receptor. alphaSubunit
80245	+	+	+	+	+	+			Membrane protein. yif1. involved in biogenesis of COPII transport vesicles
80252									unknown protein. in Sordariomycetes
80265	+	+	+	+	+	+			unknown protein

80268	+	+	+	+	+	+			fumarate lyase
80273									phosphatidylinositol polyphosphate phosphatase
80277	+	+	+	+	+	+			Rho-guanyl nucleotide exchange factor
80282	+	+	+	+	+	+			unknown protein
80288	+	+	+	+	+	+	1.203 up	2.718 up	cytochrome c oxidase polypeptide V
80291							1.110 down	2.580 down	Zn2Cys6 transcriptional regulator
80292	+	+	+	+	+	+			DSPc. Dual specificity phosphatases (DSP)
80310	+	+	+	+	+	+			vacuolar protein sorting protein Vps66. putative
80312	+	+	+	+	+	+			unknown protein
80326	+	+	+	+	+	+			19S regulatory particle ATPase Rpt2
80332	+	+	+	+	+	+			mitochondrial carrier protein. putative
80335									unknown protein
80339	+	+	+	+	+	+			unknown protein
80340		+	+		+		5.037 up	6.295 up	GT α-1.6-mannosyltransferase
80359	+	+	+	+	+	+			unknown protein
80365	+	+	+	+	+	+	1.112 up	2.200 down	unknown protein
80398	+	+	+	+	+	+			importin ß KapC
80400	+	+	+	+	+	+			Ubiquitin C-terminal hydrolase
80403	+	+	+	+	+	+			unknown protein
80415	+	+	+	+	+	+			vacuolar sorting protein. vps3. and TGF beta receptor associated protein 1
80425	+	+	+	+	+	+	1.363 down	2.221 down	unknown protein
80432	+	+	+	+	+	+			half-sized ABC transporter
80437	+	+	+						unknown protein
80441	+	+	+	+	+	+	1.469 up	2.322 up	unknown protein
80447	+	+	+	+	+	+			calmodulin is involved in control of enzymes by Ca(2+). stimulates many protein kinases a
80475									unknown protein
80484	+	+	+	+	+	+			unknown protein

80498	+	+	+	+	+	+			Nucleosome assembly protein NAP-1
80499									POS9-activating factor FAP7
80509	+	+	+	+	+	+			Cyclopropane-fatty-acyl-phospholipid synthase
80515	+	+	+	+	+	+			60S ribosome biogenesis protein Mak11. putative
80523	+	+	+				1.208 down	2.604 down	pH-response regulator protein palC
80528							3.962 up	2.091 up	Gß-WD40 protein
80538	+	+	+	+	+	+			Eukaryotic translation initiation factor 3 subunit H (eIF3h). putative
80553	+	+	+	+	+	+	1.083 down	2.260 down	ADP-ribosylation factor like protein
80578									unknown protein
80579	+	+	+	+	+	+			unknown protein
80584	+	+	+	+	+	+			Oligosaccharyltransferase. beta subunit
80586	+	+	+	+	+	+			unknown protein
80592	+	+	+	+	+	+			unknown protein
80593	+	+	+	+	+	+			unknown protein
80594	+	+	+	+	+	+			unknown protein
80597	+	+	+	+	+	+			unknown protein
80605	+	+	+	+	+	+			unknown protein
80607									short chain dehydrogenase/reductase
80621	+	+	+	+	+	+			2-methylcitrate synthase
80627	+	+	+	+	+	+			unknown protein
80636							1.232 down	2.196 down	unknown protein
80639							2.616 up	4.050 up	iron transporter
80645							1.004 down	3.596 down	Amidase
80654							11.701 down	3.854 down	UDP-glucuronosyl and UDP-glucosyl transferase
80659							97.770 down	5.813 down	alcohol oxidase AOX1

80668	+	+	+	+	+	+	1.769 down	3.231 down	Zn2Cys6 transcriptional regulator
80672	+	+	+	+	+	+			cation efflux family protein. putative
80680	+	+	+	+	+	+			unknown protein
80685							1.314 up	4.278 down	unknown protein
80691	+	+	+	+	+	+			unknown protein
80695	+	+	+	+	+	+	1.119 up	2.036 down	half-sized ABC transporter
80709	+	+	+	+	+	+			pre-mRNA-splicing factor CWC21. putative
80713	+	+	+	+	+	+			GTPase acitvating protein for Rho subfamily of RAS smallGTPases. related to S. cerevisiae
80714							1.174 up	2.611 down	Dolichyl-phosphate-mannose a-mannosyltransferase
80716	+	+	+	+	+	+			ornithine aminotransferase
80725	+	+	+	+	+	+			unknown protein
80732	+	+	+	+	+	+			histone H3 lysine 36 (K36) methyltransferase
80733							1.493 up	2.345 up	unknown protein
80741									unknown protein
80743	+	+	+	+	+	+			unknown protein
80746									aryl-alcohol oxidase
80748	+	+	+	+	+	+			RNA polymerase I specific transcription stimulatory factor composed of Uaf30p
80756									unknown protein
80758	+	+	+	+	+	+	1.095 down	2.026 down	serine/threonine protein kinase nrc2
80761	+	+	+	+	+	+			seryl-tRNA synthetase. class IIa.
80762	+	+	+	+	+	+			ubiquitin carboxyl-terminal hydrolase. family 1
80767	+	+	+	+	+	+			MFS permease
80771	+	+	+	+	+	+			unknown protein
80775	+	+	+	+	+	+			calcium dependent mitochondrial carrier protein. putative
80778									alpha/beta hydrolase
80784	+	+	+	+	+	+			unknown protein

80788				+	+	+	+	+	+			Peroxisomal membrane anchor protein (Pex14p). putative
80792	+	+	+	+	+	+	+	+	+			brix domain-containing protein
80797												histone deacetylase
80806				+	+	+	+	+	+			unknown protein
80807						+						unknown protein
80813					+	+						unknown protein
80820				+	+	+	+	+	+			HisG ATP phosphoribosyltransferase
80825				+	+	+	+	+	+			unknown protein
80833												GH18 chitinase Chi18-5
80835				+	+	+	+	+	+			SWI/SNF complex protein
80843				+	+	+	+	+	+	1.043 down	2.809 up	26S proteasome regulatory complex subunit Rpn8
80854				+	+	+	+	+	+			ribosomal protein MRPL24.
80863				+	+	+						unknown protein
80867				+	+	+	+	+	+			unknown protein
80870				+	+	+	+	+	+			unknown protein
80871										1.732 down	3.483 down	unknown protein
80872				+	+	+	+	+	+			phosphoribosylamine-glycine ligase
80875				+	+	+	+	+	+	1.465 up	4.312 down	MFS permease
80879				+		+	+					MFS H+/oligopeptide transporter
80881				+	+	+	+	+	+			GDP-forming succinate-CoA ligase. beta subunit
80890				+	+	+	+	+	+			3' exoribonuclease
80898				+	+	+	+	+	+			Secretion related small GTPase Rab1/Ypt1
80904				+	+	+	+	+	+			unknown protein
80911				+	+	+						tripeptide peptidase
80920				+	+	+			+			ADH1
80922				+	+	+	+	+	+			unknown protein
80932				+	+	+	+	+	+			Cytochrome c oxidase assembly protein CtaG/Cox11

80941	+	+	+						TRNA pseudouridine synthase.
80956	+	+	+	+	+	+			RNA recognition protein. RNP-1
80958	+	+	+	+	+	+			phosphatidylserine decarboxylase proenzyme 2 precursor
80961	+	+	+	+	+	+			unknown protein
80973									PDR-type ABC transporters
80980							5.500 up	4.778 up	peptidyl arginine deiminase
80990	+	+	+	+	+	+			unknown protein
80994	+	+	+	+	+	+			N-acetylglucosamine-phosphate mutase
80997	+	+	+	+	+	+			HNRNP arginine N-methyltransferase
81001	+	+	+	+	+	+			Rps8 gene based on similarity to NSA2 gene of yeast that confersResistance to killer toxin.
81004							1.369 down	2.202 down	aspartyl protease
81007	+	+	+	+	+	+			serine/threonine-protein kinase RIO1
81014		+							NRPS
81019		+					1.642 down	5.665 down	Zinc-containing alcohol dehydrogenase
81022							1.047 up	3.411 down	allantoate permease. 10 TM domains
81043	+	+	+	+	+	+			DNA directed RNA polymerase II 15 kDa subunit. putative
81049									C-14 sterol reductase
81058	+	+	+	+	+	+	2.175 down	3.544 down	peptidase family S58
81070	+	+	+	+	+	+			amidase
81082							11.207 up	25.313 up	aquaglyceroporin
81087			+				10.395 down	3.035 up	aminopeptidase Y
81089	+	+	+	+	+	+			CysK. Cysteine synthase; aa370-414 cd02205. CBS domain
81093	+	+	+	+	+	+			unknown protein
81096							1.024 up	14.686 up	ferric reductase
81097	+	+	+	+	+	+			unknown protein with TLC domain
81102	+		+	+					unknown protein

81105	+	+	+	+	+	+			unknown protein
81109	+	+	+	+	+	+			unknown protein
81110	+	+	+	+	+	+			acetyl-CoA carboxylase
81115	+	+	+	+	+	+			Peptidase D
81122									unknown protein. 4 TM. only in Sordariomycetes
81125	+	+	+	+	+	+	2.107 down	2.495 down	Amino acid permease
81136	+	+	+	+	+	+			serine/threonine phosphatase 2 C. PTC2
81139	+	+	+	+	+	+			ribosomal protein S4.
81140	+	+	+	+	+	+			Ribosomal protein S18.
81148		+							short chain dehydrogenase/reductase
81149	+	+	+	+	+	+			aquaglyceroporin
81150							1.513 up	2.272 down	unknown protein
81164	+	+	+	+	+	+			Protein phosphatase type 2C Ptc2
81174	+	+	+	+	+	+			unknown protein
81183	+	+	+	+	+	+	1.134 down	2.366 down	unknown protein
81188	+	+	+						HisF Imidazoleglycerol-phosphate synthase
81211	+	+	+	+	+	+			GT α-1.6-mannosyltransferase
81214	+	+	+	+	+	+			unknown protein
81219		+							Serine/threonine protein kinase
81226	+	+	+	+	+	+			unknown protein
81239									GDP-mannose transporter
81259	+	+	+	+	+	+			unknown protein
81260	+	+	+	+	+	+			adenylate kinase
81263	+	+	+	+	+	+			ER lumen protein retaining receptor Erd2
81271	+	+	+	+	+	+	2.399 up	3.113 up	xylitol dehydrogenase XDH1
81275	+	+	+				2.360 down	3.247 down	phytanoyl-CoA dioxygenase family protein
81291	+	+	+	+	+	+			unknown protein

81296	+	+	+	+	+	+			SSCRP
81303	+	+	+	+	+	+			fumarate reductase
81328		+							unknown protein. only in fungi
81329	+	+	+	+	+	+			unknown protein
81331		+	+						SSCRP
81339	+	+	+	+	+	+			glycine dehydrogenase
81343	+	+	+	+	+	+			unknown protein
81348	+	+	+	+	+	+			amino acid transporter
81362	+	+	+	+	+	+	1.290 up	2.199 up	Lipoate synthase
81383							3.063 up	6.292 up	GPCR . contains RGS domain
81389	+	+	+	+	+	+	3.699 down	4.788 down	phosphate/H+ symporter
81405	+	+	+	+	+	+			unknown protein
81410	+	+	+	+		+			unknown protein
81416	+	+	+	+	+	+			unknown protein
81420	+	+	+	+			2.875 down	2.643 down	Argonaute siRNA chaperone (ARC) complex subunit
81423	+	+	+	+	+	+	1.091 down	2.463 up	unknown protein
81430	+	+	+	+	+	+			Cation transporting ATPase
81442									Amino acid transporters
81447	+	+	+	+	+	+			GTP-binding protein YchF
81450	+	+	+	+	+	+			Mitochondrial import inner membrane translocase. subunit Tim44
81454	+	+	+	+	+		1.024 down	2.735 down	unknown protein
81457	+	+	+	+	+	+			unknown protein
81460		+							unknown protein. secreted
81465	+	+	+	+	+	+			unknown protein
81473	+	+	+	+	+	+	1.282 up	2.031 up	caffeine-induced death protein 2
81511							1.824 down	2.453 down	formamidase

81517				+	+	+	+	+	+			unknown protein
81522				+	+	+	+	+	+			Selenocysteine lyase-like protein
81525												isoflavone reductase
81536				+	+	+	+	+				cation transporting ATPase
81541				+		+						unknown protein
81546				+	+	+	+	+	+			sulfate transporter
81553												short chain dehydrogenase/reductase
81565				+	+	+	+	+	+			C2HC5 finger protein
81570	+	+	+	+	+	+	+	+	+			Homoaconitase catalytic domain
81576				+	+	+	+		+			Assimilatory sulfite reductase.Alpha subunit
81586					+							unknown protein
81591												unknown protein
81593				+	+	+	+	+	+			MFS permease
81598												GH18. chitinase CHI18-7
81599				+	+	+	+	+	+			pre-rRNA processing nucleolar protein Sik1. putative
81609				+	+	+				1.338 down	2.033 down	GH15
81616				+		+	+					unknown protein
81623										1.859 down	2.465 down	unknown protein. only in Hypocreaceae
81630				+	+	+	+	+	+			unknown protein
81646				+	+	+	+	+	+	1.513 down	2.238 down	NADPH oxidase
81649												Flavin-containing monooxygenase
81652												unknown protein
81659										10.047 down	3.646 down	unknown protein
81661				+	+	+	+	+	+			GT ß-glycosyltransferases
81668												unknown protein
81670				+	+	+						MFS permease

81676							1.496 down	5.583 down	salicylate hydroxylase
81679		+	+						Flavoprotein monooxygenase. putative
81688	+	+	+	+	+	+			unknown protein
81690	+	+	+	+	+	+			ß-arrestin protein. shares similarity with Aspergillus idulans CreD. possible inhibitor of G-p
81700	+	+	+	+	+	+	1.144 down	2.691 down	mannose-6-phosphate isomerase
81713	+	+	+	+	+	+			ribosomal protein S24e. Homologue of yeast RPS24a/b.
81716	+	+	+	+	+	+			peptidyl-prolyl cis-trans isomerase
81720							1.222 down	2.629 down	Ume5 gen
81730	+	+	+	+	+	+	1.224 up	2.100 up	glutaredoxin
81742	+	+	+	+	+	+			RNA polymerase I subunit Rpa43. putative
81756									unknown protein
81757	+	+	+	+	+	+			unknown protein
81763	+	+	+	+	+	+			unknown protein
81774	+	+	+	+	+	+	1.141 down	2.798 down	unknown protein
81778									glutaminase A
81779	+	+	+	+	+	+			ribosomal protein MRPL20.
81783	+	+	+	+	+	+			unknown protein
81785	+	+	+	+	+	+			GTPase activating factor (RasGAP) related to S. cerevisiae Bud2
81797	+	+	+	+	+	+			Sec1 family member sly1
81803	+	+	+	+	+	+			mitochondrial protein sorting (Msf1)
81804	+	+	+	+	+	+			methionine aminopeptidase
81819									unknown protein
81822	+	+	+	+	+	+			mitochondrial leucyl-tRNA synthetase.
81824	+	+	+	+	+	+			MTHFRMethylenetetrahydrofolate reductase
81843		+					2.746 down	9.875 down	Alkaline phosphatase
81855	+	+	+	+	+	+			pyruvate dehydrogenase kinase. putative

81859				+	+	+	+	+	+			Ubiquitin-specific protease
81861				+	+	+	+	+	+			protein kinase BUR1
81865				+	+	+	+	+	+			unknown protein
81882				+	+	+	+	+	+			unknown protein
81884				+	+	+	+	+	+			Translocon-associated protein (TRAP). alpha subunit
81889				+	+	+	+	+	+			unknown protein
81896												aspartate kinase
81906				+	+	+	+	+	+	1.461 up	2.310 up	unknown protein
81911				+	+	+	+	+	+	1.107 down	2.299 down	unknown protein
81920				+	+	+	+	+	+	1.128 up	2.336 up	SSCRP
81925				+	+	+	+	+	+			unknown protein
81930				+	+	+	+		+			HAM1-like protein. probably related to DNA-repair
81939				+	+	+	+	+	+			unknown protein
81949	+	+	+	+	+	+	+	+	+			Winged helix repressor DNA-binding
81955										1.008 up	2.970 up	nitrate reductase
81964												PKS
81972				+	+	+	+	+	+			phosphatidylinositol transporter. putative
81979										9.476 down	5.025 down	glutathione-S-transferase
81984				+	+	+	+	+	+			palmitoyl protein thioesterase
81990				+	+	+	+	+	+			unknown protein
82001				+	+	+	+	+	+			unknown protein
82014												Aa_transTransmembrane amino acid transporter protein
82017												siderophore transporter
82026												trans-aconitate methyltransferase
82032						+						unknown protein
82037				+	+	+	+	+	+	1.163 up	2.719 down	Inorganic phosphate transporter
82039												D-aminoacylase. putative

82041												PTH11 GPCR
82049										1.577 up	7.546 up	unknown protein
82068												unknown protein
82074												unknown protein
82085				+	+	+	+	+	+			glutaredoxin Grx1. putative
82087				+	+	+	+	+	+			unknown protein
82095										1.055 up	2.518 up	ammonium transporter. high affinity
82100				+	+	+	+	+	+			PhosphatidylinositolPolyphosphatePhosphatase
82105				+						2.755 down	4.288 down	PDR-type ABC transporters
82123				+	+	+	+	+	+			unknown protein
82125				+	+	+	+	+	+			mediator of RNA polymerase II transcription subunit 7. putative
82133				+	+	+	+	+	+			cytochrome P450. putative
82137												unknown protein
82141						+						serine carboxypeptidase S28. putative
82145	+	+			+	+	+	+	+			GDP-fucose transporter (putative)
82146						+						unknown protein
82150				+	+	+	+	+	+			phosphate transporter (Pho88). putative
82153				+	+	+	+	+	+			unknown protein
82172												lipoate-protein ligase A. putative
82175				+	+	+	+	+	+			serine/threonine protein kinase
82177												3-oxo-5-alpha-steroid 4-dehydrogenase
82197				+	+	+	+	+	+			CN_hydrolaseCarbon-nitrogen hydrolase
82204					+					1.113 down	2.439 down	ammonium transporter. high affinity
82208												PKS
82214												unknown protein
82227												GH3 β-glucosidase CEL3c
82235				+	+	+				1.174 up	2.844 up	GH31 α-glucosidase
82241												Zn2Cys6 transcriptional regulator

82246								1.358 up	2.245 up	GCPR. mPR-type
82260		+	+	+	+	+	+			unknown protein
82280		+	+	+	+	+	+			mitochondrial 2-oxoglutarate/2-oxoadipate transporter-like protein
82286		+	+	+	+	+	+	1.478 up	2.044 up	unknown protein
82296										MFS permease
82309								1.390 down	5.190 down	MFS permease
82317										Unknown protein
82321								122.123 down	2.981 down	Aquaporin
82326		+	+	+	+	+	+			phosphoglycerate mutase family protein
82327								4.846 down	5.701 down	PDR-type ABC transporters
82334		+	+	+	+	+	+			tyrosyl-tRNA synthetase. class Ib.
82351		+	+	+	+	+	+			MAPK2. mitogen activated protein kinase 2. probably involved in stress response
82356		+	+	+	+	+	+			unknown protein
82360		+	+	+	+	+	+			lipase/serine esterase. putative
82374		+	+	+	+	+	+	2.028 down	4.120 down	unknown protein
82380		+	+	+	+	+	+			initiation factor 2B.
82385		+	+	+	+	+	+			methionyl-tRNA synthetase.
82403		+	+	+	+	+	+	1.198 down	3.822 down	Acyl-CoA dehydrogenase
82409		+	+	+	+	+	+			Amidohydrolase
82425		+	+	+	+	+	+	1.070 up	2.685 up	unknown protein
82434		+	+	+	+	+	+			Unknown protein with TPR structural motif.
82442			+							Several tryptophanyl-tRNASynthetases from other fungi.
82451		+	+	+	+	+	+			1.2-dihydroxy-3-keto-5-methylthiopentene dioxygenase . putative
82452		+	+	+	+	+	+			Aminopeptidase P
82458		+	+	+	+	+	+			Peptidase C19. ubiquitin carboxyl-terminal hydrolase 2
82469	+									unknown protein

82475	+	+	+	+	+	+			Ras GTPase-activating protein with distant similarity to human neurofibromin. possibly inv
82476	+	+	+	+	+	+			Exocyst component Sec15
82499	+	+	+	+	+	+			Integral membrane protein. putative
82510	+	+	+	+	+	+			histone H4 variant
82512	+	+	+	+	+	+			26S proteasome regulatory complex subunit Rpn4
82516	+	+	+	+	+	+			Pentafunctional Aromatic Polypeptide
82519	+	+	+	+	+	+			unknown protein
82523	+	+	+	+	+		1.146 down	2.139 down	unknown protein
82529	+	+	+	+	+	+			membrane-bound O-acyltransferase domain-containing protein
82531	+	+	+	+	+	+			GTPase-activating protein gyp10. putative
82534	+	+	+	+	+	+			heat shock protein (Hsp70 chaperone Hsp88)
82539	+	+	+	+	+	+			nonsense-mediated mRNA decay factor (Upf2). putative
82544	+	+	+						Calcium transporter
82547	+	+	+	+	+	+			cystathionine beta-lyase
82551	+	+	+	+	+	+			GT α-1.6-mannosyltransferase
82560	+	+	+	+	+	+	2.091 up	2.857 up	Ribokinase
82562	+	+	+	+	+	+			unknown protein
82568	+	+	+	+	+	+			AAA ATPase
82577	+	+	+	+	+	+			ArgE. Acetylornithine deacetylase/Succinyl-diaminopimelate desuccinylase and related de
82591	+	+	+	+	+	+	3.120 down	3.377 down	unknown protein with oxidoreductase domain
82599	+	+	+	+		+			unknown protein
82601	+	+	+	+	+	+			AldedhAldehyde dehydrogenase family
82606	+	+	+	+	+	+	1.068 up	2.174 up	Mn2+ homeostasis protein Per1
82613	+	+	+	+	+	+			translation elongation factor 1 beta by homology to the corresponding gene in other fung
82616	+		+				5.179 down	4.507 down	GH5 membrane bound endoglucanase CEL5b
82619							1.131 up	9.196 down	arginosuccinate synthetase
82623									tripeptide peptidase

82626							1.093 up	3.230 down	half-sized ABC transporter
82628							4.132 up	2.764 up	acetyltransferase SidF
82633			+				3.948 down	4.784 down	GH72 β-1 3-glucanosyltransferase
82651	+	+	+	+	+	+			Cullin
82662							75.475 down	4.705 down	Epl1/Sm1
82667	+	+	+	+	+	+			ThrB Homoserine kinase
82668	+	+	+	+	+	+			nuclear pore complex protein sonA
102377	+	+	+	+	+	+			unknown protein
102378	+	+	+	+	+	+			kinesin-like protein. a kinesin-3 family member.
102379		+							unknown protein
102381	+	+	+	+	+	+			Calcium-binding EF-hand
102382									hydroxyacylglutathione hydrolase
102383							1.548 down	4.029 down	unknown protein
102385							1.011 down	2.671 up	unknown protein
102386							1.618 up	2.391 up	unique protein
102401	+	+	+	+	+	+			DNA polymeraseDelta. small regulatory subunit
102403									unknown protein
102411							1.522 down	2.640 down	unknown protein
102414	+	+	+						unknown protein
102416							2.132 down	2.163 down	amino acid permease PotE
102437									unique protein. secreted
102441		+							unknown protein
102444	+	+	+	+	+	+			unknown protein
102451	+	+	+	+	+	+			unknown protein

102454				+	+	+	+	+	+	1.741 up	2.160 up	unknown protein
102458				+	+	+	+	+	+			QDE3. RecQ helicase. essential for quelling
102461				+	+	+	+	+	+			unknown protein
102464				+	+	+	+	+	+			bHLH transcriptional regulator
102467										1.499 down	3.218 up	unknown protein
102468												unknown protein
102476				+	+	+	+	+	+			unknown protein
102482												unknown protein
102487												Cytochrome P450 CYP2 subfamily
102489												unknown protein
102492												Short-chain dehydrogenase/reductase
102497				+	+	+	+	+	+			Zn2Cys6 transcriptional regulator
102499						+				1.036 down	6.618 up	Zn2Cys6 transcriptional regulator
102500										1.148 down	3.845 up	MRSP1/expansin-like
102504												unknown protein
102512				+	+	+	+	+	+			unknown protein
102521				+	+	+						unknown protein
102554				+	+	+	+	+	+			small nuclear ribonucleoprotein Lsm8
102555					+							inosine-uridine preferring nucleoside hydrolase
102559				+	+	+	+	+	+			integral membrane protein
102561												FAD binding domain-containing protein
102562												unknown protein. Duf899 domain
102567	+	+	+							1.516 up	3.056 up	unknown protein
102572				+	+				+			Peptidyl-prolyl cis-trans isomerase PIN4 (EC 5.2.1.8) (PPIase PIN4)
102579				+	+	+	+	+	+			E3 ubiquitin-protein ligase BRE1 (EC 6.3.2.-) [BRE1]
102581				+	+	+	+	+	+			Ubiquitin binding protein Rad23
102582				+	+	+	+	+	+			unknown protein

102593	+	+	+	+	+	+	1.550 up	2.534 up	Mitochondrial ribosomal protein L17
102599		+							unknown protein
102603	+	+	+	+	+	+			Pre-mRNA-splicing factor ini1
102607	+	+	+	+	+	+			unknown protein
102612	+	+	+	+	+	+			inositol-pentakisphosphate 2-kinase
102616	+	+	+	+	+	+			transcriptional corepressor Cyc8
102617	+	+	+	+	+	+			unknown protein
102619									unknown protein
102627	+	+	+	+	+	+			unknown protein
102637							1.847 up	3.624 up	unique protein
102640	+	+	+	+	+	+			unknown protein
102641	+	+	+	+	+	+			unknown protein
102648	+	+	+	+	+	+			unknown protein
102650	+	+	+	+	+	+			unknown protein
102652	+	+	+	+	+	+			unknown protein
102655	+	+	+	+	+	+			cAMP phosphodiesterase PDE2. high affinity
102659	+	+	+	+	+	+			GTPase with a role in regulation of membrane traffic. arl1; G protein of the Ras superfami
102668	+	+	+	+	+	+			unknown protein
102672	+	+	+	+	+	+			unknown protein
102673	+	+	+		+				unknwon zinc finger protein
102676									DNA-directed RNA polymerase. 13 to 16 kDa subunit
102677									unknown protein
102680							1.058 down	2.377 up	unique protein
102686									SSCRP
102694	+	+	+	+	+	+			Cohesin (Denison. Kafer et al. 1993)
102700	+	+	+	+	+	+			glycyl-tRNA synthase. alpha 2 dimer.
102705	+	+	+	+	+	+			unknown protein
102711	+	+	+	+	+	+			serine/threonine-protein kinase. putative
102723									TBC domain protein (GAP)

102735		+							unknown protein. WSC domain
102737	+	+	+						VosA ?
102738	+	+	+		+		4.027 down	2.121 down	unknown protein
102743	+	+	+		+				unknown protein. only in Sordariomycetes
102744	+	+	+	+	+	+			TrpE Anthranilate/para-aminobenzoate synthases component I
102763		+	+						unknown protein
102766									short chain dehydrogenase/reductase
102769	+	+	+	+	+	+			unknown protein
102772									unknown protein
102773							1.674 up	6.196 up	unknown protein
102774							1.389 up	5.248 up	unknown protein
102775									unknown protein
102776		+	+		+				unknown protein
102778									unknown protein
102779							2.439 up	2.160 up	unknown protein
102780							1.340 up	4.147 up	unknown protein
102785									unknown protein
102787							1.879 up	2.600 up	unique protein
102788							1.186 up	3.375 up	unique protein
102789									unique protein
102794	+	+	+	+		+			RNA recognition domain protein
102799									unknown protein
102813	+	+	+	+	+	+			dynactin ro-3
102816	+	+	+	+	+	+	1.513 down	2.344 down	Zn2Cys6 transcriptional regulator
102820									ferrooxidoreductase
102830	+	+	+		+		4.386 down	2.858 down	unknown protein
102836									unknown protein

102837									unknown protein with CFEM domain
102846							1.230 down	2.239 down	unknown protein
102850							1.029 up	2.222 down	unique protein
102851							4.207 up	9.908 up	SSCRP
102855									SAM methyltransferase TRM9 (tRNA carboxylmethyl transferase).
102863							1.103 down	2.858 down	unknown protein
102864									unknown protein
102870	+	+	+	+	+	+			unknown protein
102871		+							unique protein
102876							1.040 up	2.045 down	unique protein
102884									unique protein
102887	+	+	+				1.447 up	3.225 up	unique protein
102890									unknown protein
102892	+	+	+	+	+	+	1.165 down	2.607 down	unknown protein
102901	+	+	+	+	+	+			unknown protein
102904			+						unknown protein
102906							2.927 down	4.337 down	unique protein
102908	+	+	+						SSCRP
102909									GH2 protein
102910	+	+	+	+	+	+			unknown protein
102920	+	+	+	+		+	4.625 down	6.239 down	C2H2 transcriptional regulator
102927							1.412 up	2.592 up	Serine/threonine protein kinase
102933							1.831 up	3.914 down	unknown protein. only in Gibberella
102935							1.124 up	2.963 up	unknown protein

102936										1.234 up	3.549 up	unknown protein
102938												SSCRP
102941				+	+	+	+	+	+			unknown protein
102947												unknown protein
102952												unknown protein
102953										3.052 down	4.708 down	dityrosine transporter. required for spore wall synthesis
102957												2-oxoglutarate-dependent ethylene/succinate-forming enzyme. putative
102960				+	+	+	+	+		1.842 up	2.297 up	unique protein. 2 TM
102964				+	+	+		+				ribosomal protein S2.
102966						+						unknown protein
102970	+	+	+									unique protein
102973				+	+	+	+	+	+			unknown protein
102976				+	+	+	+	+	+	1.588 up	2.411 up	unknown protein
102980				+	+	+	+	+	+			ubiquitin-conjugating enzyme. putative
102985				+	+	+	+	+	+	1.861 down	3.234 down	unknown protein
102989												unknown protein
102996												unknown protein
102998												ADP-ribosylglycohydrolase-like protein
102999										6.126 up	11.491 up	unknown protein
103002						+						unique protein
103006												unknown protein. only in Hypocreaceae
103009												unknown protein
103012												taurine catabolism dioxygenase. alpha-ketoglutarate dependent
103015										8.856 down	10.365 down	unknown protein
103016										1.057 down	2.695 up	unique protein
103023												unknown protein
103028				+	+	+	+	+	+			unique protein

103031							unknown protein
103032					1.208 up	3.405 up	unknown protein
103033							PL7 alignate lyase
103034					3.507 down	9.746 down	Zn2Cys6 transcriptional regulator
103039							peptidase S41
103041					4.814 down	6.212 up	acetamidase
103043	+	+	+		1.105 up	3.003 up	unknown protein
103044	+	+	+		1.148 up	2.795 up	unique protein
103045	+	+	+				
103048							unique protein
103049					2.382 down	2.468 up	GH28 endo-polygalacturonase
103050							unknown protein
103059					2.053 down	2.031 up	unknown protein
103060							unknown protein
103061					1.298 up	4.189 up	unknown protein
103062							unique protein
103063					1.738 down	3.827 up	unknown protein
103064					6.552 down	2.463 up	unknown protein
103065					1.378 up	3.061 up	unknown protein
103073				+	1.205 up	2.041 up	unknown protein
103080							unknown protein
103103							GCN5-N-Acetyltransferase
103108							phytanoyl-CoA dioxygenase family protein
103112					2.549 up	4.117 up	unknown protein
103113							short chain dehydrognease/reductase

103114							1.079 up	3.106 up	unknown protein
103119							1.115 up	4.041 up	membrane dipeptidase GliJ
103121							1.200 up	4.041 up	unique protein
103122							1.000 down	3.653 up	Zn2Cys6 transcriptional regulator
103127									unknown protein
103129							1.111 up	2.343 up	unknown protein
103130							1.315 up	2.650 up	unique protein
103131									unknown protein. 1 TM
103132	+	+	+	+	+	+			acetylglutamate kinase ARG6
103133									unknown protein
103134									unknown protein only present with low similarity in Neurospora
103135							1.070 up	16.190 up	SSCRP
103136							1.614 up	2.455 up	Glycolate oxidase
103138							1.029 down	2.460 down	Zn2Cys6 transcriptional regulator
103143									unknown protein
103145							3.357 down	3.491 down	unique protein
103147	+	+	+	+		+	1.749 down	5.977 down	unknown protein
103149							18.085 down	6.549 down	AAA ATPase
103153									unique protein
103155							1.153 up	3.630 up	unknown protein
103156							19.725 down	3.204 down	unknown protein
103157							1.024 up	4.853 down	unknown protein
103158							2.610 down	7.664 down	Zn2Cys6 transcriptional regulator
103162									Dihydrolipoamide acetyltransferase

103167				+	+	+	+	+	+			unknown protein
103172										5.905 down	2.021 down	unique protein
103174										1.007 up	4.014 up	SSCRP
103175										1.476 up	3.592 up	epoxide hydrolase. putative
103176										1.004 up	2.534 up	unknown protein
103177										2.072 up	2.395 up	glutathionine S-transferase
103179	+											MFS permease
103180			+			+						unknown protein
103186										1.367 down	2.113 up	unknown protein
103189										3.073 down	2.586 down	HET protein
103191	+	+	+									unknown protein
103193										10.151 down	7.186 down	ankyrin
103205										5.009 up	4.748 up	unknown protein
103209				+	+	+	+	+	+			unknown protein
103215				+		+				1.392 down	3.073 down	Cytochrome P450 CYP4/CYP19/CYP26 subfamilies
103222				+	+	+	+	+	+	1.518 up	2.257 up	Ribosomal protein S29 (S14 family) by homology to the corresponding protein of N. crassa
103230				+	+	+	+		+	1.127 down	4.801 down	Zn2Cys6 transcriptional regulator
103236										1.562 down	2.773 up	unknown protein
103252				+	+	+			+			unknown protein
103273				+	+	+						RNA polymerase II Elongator subunit
103275												C2H2 transcriptional regulator
103276												unique protein
103282					+							unknown protein
103286					+					1.300 up	4.691 up	unique protein

103291									unknown protein
103302		+							unknown protein
103303									unknown protein
103311	+	+	+	+	+	+			chromatin assembly factor 1 subunit C
103312									unknown protein
103321	+	+	+	+	+	+			U4/U6.U5 tri-snRNP-associated protein
103322	+	+	+	+	+	+			translation initiation factor 4e [Neurospora crassa].
103330									
103335							1.274 up	5.229 up	unknown protein
103336									unknown protein
103348									exosome complex exonuclease RRP4
103350	+	+	+	+	+	+			unknown protein
103356	+	+	+	+	+	+	1.124 down	2.175 down	Vacuolar transporter chaperone
103364									unknown protein
103366									unknown protein
103372	+	+	+	+	+	+	1.646 down	2.811 down	BZIP transcriptional regulator
103376	+	+	+	+	+	+			unknown protein
103393									SSCRP
103394							1.079 down	3.209 up	unknown protein
103395							1.010 up	3.104 up	unknown protein
103403							1.289 up	3.154 up	unknown protein
103411									unknown protein
103421									unknown protein. only in Hypocreaceae
103423							1.092 down	2.619 up	SSCRP
103437									unknown protein
103438									unique protein
103442									unique protein

103446									unknown protein
103447							1.104 up	2.307 down	unknown protein
103451									GDP-forming succinate-CoA ligase. beta subunit
103455							3.006 up	3.367 up	unknown protein
103458									GH25 N.O-diacetylmuramidase
103459									unique protein
103463									unknown protein
103470									unknown protein
103482		+					1.723 down	4.604 down	transcriptional activator with ariadne RING finger
103487									glutathione S transferase
103498	+	+	+	+	+	+			unknown protein
103506	+	+	+	+	+	+	1.748 down	2.197 down	unknown protein
103517	+	+	+	+					pyrimidine 5'-nucleotidase. putative
103537							21.752 up	15.724 up	GCN5-N-acetyltransferase
103555	+	+	+	+	+	+			unknown protein
103560									unknown protein
103563									riboflavin biosynthesis protein Rib7
103576									unknown protein
103579		+							unique protein. secreted. 1 TM
103594									unknown protein
103597									unknown protein
103599	+	+	+	+	+	+			ESF2. involved in pre-18S rRNA processing.
103613									unique protein
103614									unique protein
103618	+	+	+	+	+	+			t-SNARE Ufe1; ER membrane fusion and vesicular traffic
103625	+	+	+	+	+	+			proteasome maturation factor UMP1. putative
103651							1.319 up	2.880 up	unknown protein

103653							1.046 down	2.489 up	unique protein
103655							1.654 up	2.620 up	rad55
103660									unique protein
103668							1.348 down	2.508 down	unique protein
103669	+	+	+	+	+	+			α/ß hydrolase lipase
103671							1.602 down	3.810 down	unknown protein
103683							1.654 up	2.075 down	unique protein
103694	+	+	+	+	+	+	1.135 down	6.223 down	PTH11 GPCR
103695									unknown protein
103703	+	+	+	+	+	+			Pex2/Pex12 amino terminal region
103712									unknown protein
103715							1.364 up	3.923 up	unique protein
103720	+	+	+	+	+	+			unknown protein
103725									unknown protein
103739									Hsp27-ERE-TATA-binding protein/Scaffold attachment factor (SAF-B)
103740									unknown protein
103745	+	+	+	+	+	+			unknown protein
103747	+	+		+	+	+			unknown protein
103751									Zn2Cys6 transcriptional regulator
103754		+	+						serine/threonine protein kinase. PAK/STE20 subfamily
103755									unique protein
103756	+	+	+	+	+	+			ATP-dependent protease La
103761									unknown protein
103771									unknown protein
103772									unknown protein
103774									unique protein

103778								1.045 down	6.708 up	unique protein
103798		+	+	+	+	+	+	2.520 down	3.567 down	SSCRP
103799		+		+				1.497 down	2.834 down	unique protein
103801										unknown protein
103804								1.300 down	2.422 down	SWI-SNF chromatin-remodeling complex protein
103812		+	+	+	+					unknown protein
103813										Serine/threonine protein kinase
103822										unknown protein
103825										CE16 acetyl esterase
103827										unique protein
103844		+	+	+						unknown protein
103850			+	+						Beta-1.4-mannosyltransferase
103852	+							1.057 up	2.304 down	unknown protein
103853		+	+	+	+	+	+			Mitochondrial carnitine/acyl carnitine carrier
103861		+	+	+						unknown protein
103863								1.224 up	2.825 up	unknown protein
103865		+	+	+	+	+	+			unknown protein
103866										unique protein
103886		+	+	+	+	+	+	1.097 down	2.746 down	unknown protein
103898								2.071 up	2.304 up	unknown protein
103900								1.039 up	3.474 up	unknown protein
103907								1.380 up	3.721 down	unknown protein
103917		+	+	+	+	+	+			unknown protein
103920				+						unknown protein. only in Magnaporthe

103930										1.251 down	2.725 down	unknown protein
103937				+	+	+	+	+	+			unknown protein
103947				+	+	+	+	+	+			unknown protein
103949				+	+	+	+	+	+	1.170 up	2.376 up	unknown protein
103951												PLP dependent enzymes class III
103959						+				1.069 down	2.748 up	unique protein
103960					+	+				2.353 down	3.107 down	nitrilase
103973				+	+	+	+	+	+	1.398 up	4.665 up	unknown protein
103993					+							RNA exonuclease 4
103995				+	+	+	+	+	+			unknown protein
104000				+	+	+	+	+	+	1.918 up	2.158 up	DNA repair protein rad18
104004				+	+	+	+	+	+			transcriptional regulator. unknown
104015				+	+	+	+	+	+			RNA polymerase Rpb8
104016					+					1.120 up	2.070 down	unknown protein
104021						+						unknown protein
104046	+	+	+							1.311 up	2.853 up	unique protein
104050										1.559 down	14.834 up	SSCRP
104054										1.043 up	3.720 up	unknown protein
104059										1.067 down	3.602 up	short chain dehydrognease/reductase
104060										1.197 down	3.301 up	short chain dehydrognease/reductase
104061												unknown protein
104064										1.057 down	3.660 up	unknown protein
104067										1.162 down	6.266 up	unknown protein

104071							1.019 down	2.566 up	unknown protein
104072									xylose transporter
104073									aspartate racemase
104075							1.213 down	2.653 down	Zn2Cys6 transcriptional regulator
104077							1.291 down	12.210 down	Amino acid transporter PotE
104079							1.112 down	3.604 up	MRSP1/expansin-like
104081							1.026 down	2.959 up	coenzyme F420-dependent N5.N10-methylene tetrahydromethanopterin reductase
104084									unknown protein
104089									unknown protein
104097			+						unknown protein
104102									unknown protein
104103									short chain dehydrogenase/reductase
104104									Zn2Cys6 transcriptional regulator
104106							1.073 down	2.488 up	ADP/ATP carrier protein
104109							1.121 up	2.331 up	unique protein
104115			+						Cytochrome P450 CYP2 subfamily
104116									Zinc-containing alcohol dehydrogenase
104117									unknown protein
104118							1.156 up	5.813 up	unique protein
104121	+	+	+	+	+	+			GT α-1.3-mannosyltransferase
104132	+	+	+	+	+	+			unknown protein
104135									Thioredoxin. putative
104137		+							unknown protein
104140	+	+	+	+	+	+	2.606 up	2.557 up	3-hydroxi-isobutyrate dehydrogenase
104141	+	+	+	+	+	+			transcriptional regulator APSES type

104152		+	+	+	+	+	+			unknown protein
104156	+									unknown protein
104157		+	+	+	+	+	+	1.015 down	2.282 up	unique protein
104159		+	+	+	+	+	+			MUS26 of Neurospora crassa.MUS26 is a probable homolog of Rev7p of Saccharomyces c
104161		+	+	+	+	+	+			importin ß KapG
104168								2.519 up	2.197 up	unknown protein
104171										unknown protein
104173								1.554 down	2.475 up	unique protein
104174										unknown protein
104175								1.211 up	2.594 up	unique protein
104179										short chain dehydrogenase/reductase
104180								9.686 down	2.395 down	Ankyrin
104181								3.106 up	4.664 up	SSCRP
104182		+	+					2.242 down	7.991 down	Zn2Cys6 transcriptional regulator
104197										unknown protein
104199										unknown protein
104200								3.939 up	5.335 up	unknown protein. F-box
104201								1.064 down	2.585 up	unknown protein
104202										unique protein
104203										unknown protein
104206								1.211 up	2.035 up	SSCRP
104207										unknown protein
104209								1.146 up	3.511 up	unknown protein
104211				+	+		+	15.509 down	24.344 up	FKBP-type peptidyl-prolyl cis-trans isomerase

104215							1.454 down	3.289 down	unknown protein
104217									unknown protein
104219									unknown protein
104220									unique protein
104222									GCN5-related acetyltransferase
104227							1.120 down	21.005 up	SSCRP
104228							1.048 down	2.701 up	unique protein
104230	+	+	+	+	+	+			unknown protein
104231	+	+	+	+	+	+	1.087 down	2.094 down	unknown protein
104239	+	+	+	+	+	+			unknown protein
104243									unknown protein
104251									unknown protein
104260							1.303 up	3.683 up	unknown protein
104261							1.323 up	6.012 up	GCN5-related N-acetyltransferase
104263	+	+		+	+	+			unique protein
104272									unknown protein
104276							1.269 up	2.652 down	unknown protein
104277				+			1.942 down	2.882 down	cell wall protein. distantly related to A. niger CwpA.
104286							1.065 up	2.845 up	unique protein
104288									phosphatidylethanolamine-binding protein
104289							1.048 down	2.528 up	unique protein
104291			+						unknown protein
104292							1.803 up	2.411 up	mating type pheromone precursor. alpha type
104293							1.430 up	4.472 up	HFB5

104294										1.215 up	5.465 up	unknown protein
104295						+				5.144 down	5.720 up	unknown protein
104301												unknown protein
104302												unknown protein
104304										2.406 down	3.822 down	unknown protein
104310				+	+							unknown protein
104313					+							unknown protein
104318					+							unknown protein
104320												MFS permease
104322				+	+	+	+		+			unique protein
104325				+	+	+	+	+	+			unknown protein
104333												unknown protein
104334										1.156 down	2.076 down	unknown protein
104335										1.256 up	2.224 up	unknown protein
104336										1.324 down	3.040 up	unique protein
104343	+	+	+									
104351					+		+			2.431 up	2.871 up	unique protein
104353										1.651 up	2.210 up	unknown protein. contains F-box
104354										1.047 up	2.685 up	SSCRP
104356												unknown protein
104359												unknown protein
104364				+	+	+	+	+	+			MAPK mitogen activated protein kinase STE20
104368										1.153 up	3.884 up	unknown protein
104369										1.052 down	2.744 up	unknown protein
104370				+	+	+	+	+	+			unknown protein
104372												ankyrin repeat protein. 1 TM

104373										1.217 up	4.060 up	MRSP1/expansin-like
104377										1.198 down	4.890 down	unknown protein
104380										2.130 down	5.726 down	Zn2Cys6 transcriptional regulator
104382												unknown protein
104390										3.773 up	2.563 down	glutathione S-transferase
104393												SAM-dependent methyltransferase
104394												esterase
104395										1.098 up	2.721 down	unknown protein
104399	+	+	+							1.149 up	2.696 up	unique protein
104400												lipase/esterase (α/ß fold)
104401				+	+	+	+	+	+			HFBs
104413												unknown protein
104415												unknown protein
104418												Kynurenine 3-monooxygenase and related flavoprotein monooxygenases
104419				+	+		+	+	+	1.090 down	4.138 up	unique protein. secreted
104421										1.220 up	4.106 up	unknown protein
104422										1.407 down	9.137 up	unknown protein
104423										1.648 down	4.053 up	MRSP1/expansin-like
104425												unknown protein
104435	+	+	+									unknown protein
104437				+	+	+		+		3.382 up	2.494 up	unknown protein
104438												FAD-binding. oxidoreductase
104448				+	+	+						unknown protein
104451					+							retrograde regulation protein 2

104454												unknown protein
104455												unique protein
104458												SSCRP
104461						+						Epoxide hydrolase
104463					+							unknown protein
104469				+	+	+	+	+	+			unknown protein
104474				+	+	+	+	+	+			kinesin-like protein
104490				+	+	+	+	+	+	1.325 up	3.345 up	v-SNARE Gos1. Golgi transport
104491				+	+	+	+	+	+			60S ribosomal protein L35 (L29 family) by homology with the corresponding protein in oth
104494				+	+	+	+	+	+			unknown protein
104495				+	+	+	+	+	+			Pre-mRNA-processing ATP-dependent RNA helicase PRP5
104501	+	+	+							1.112 up	3.236 up	unique protein
104511	+	+	+	+	+	+	+	+	+	1.060 down	2.225 up	unique protein
104513					+					1.188 down	2.135 down	Zn2Cys6 transcriptional regulator
104514												unknown protein
104533				+	+	+	+	+		1.025 down	3.190 up	unique protein
104535				+	+	+	+	+	+			unknown protein
104541				+	+	+	+	+	+			unknown protein
104547												unknown protein
104549						+						MFS permease
104551												unknown protein
104556										1.223 up	12.129 up	unknown protein
104557										6.959 down	3.565 down	short-chain dehydrogenase/reductase
104573				+	+	+	+	+	+			unknown protein
104576	+	+	+							1.023 up	2.780 up	unique protein
104577	+	+	+	+	+	+	+	+	+	1.095 up	2.664 up	unique protein

104584				+	+	+	+		+			Epl1/Sm1
104585										1.082 down	2.283 up	unknown protein
104592										1.093 down	2.779 up	unknown protein
104593										2.936 down	2.909 down	unknown protein with ankyrin. Leu-zipper and WD40
104595				+	+	+	+	+	+	1.134 up	2.185 up	Ribosomal protein L7Ae/L30e/S12e/Gadd45. Homologue of yeast SNU13.
104599				+	+	+	+	+	+	3.147 down	5.473 down	Mandelate racemase/muconate lactonizing enzyme
104601										1.094 up	2.201 down	unknown protein
104606				+	+	+	+	+	+			Iron-containing alcohol dehydrogenase
104609												UbiE/COQ5 ubiquinone methyltransferase
104617				+	+	+				1.218 down	2.670 down	
104623	+		+	+	+	+	+	+	+			unknown protein
104639				+	+	+	+	+	+			Transport protein Uso1
104643				+	+	+	+	+	+			unknown protein
104651				+	+	+	+	+	+			unknown protein
104659												unknown protein
104661												unknown protein
104664				+	+	+	+	+	+			Mitochondrial outer membrane and cell wall localized SUN family member required for m
104683				+	+	+	+	+	+			AAA ATPase
104684				+	+	+						unknown protein
104691				+	+	+	+	+	+			unknown protein
104695				+	+	+	+		+	1.708 down	2.243 up	unique protein
104704				+	+	+	+	+	+			60S ribosomal protein RML2. mitochondrial precursor from Ashbya gossypii.
104707												unknown protein
104715	+	+	+							1.005 down	2.905 up	unique protein

104716	+	+	+							1.076 up	3.999 up	unique protein
104721				+	+	+						Late Golgi transport protein Sft2
104741										1.902 down	4.389 down	unknown protein
104744					+	+				1.111 up	2.282 up	cytochrome c oxidase assembly protein COX19. putative
104750					+							unique protein
104762				+	+	+	+	+	+	1.727 up	2.041 up	unknown protein
104785				+	+	+						unknown protein with SacI domain
104786				+	+	+	+	+	+			unknown protein
104790				+	+	+	+	+	+			Tubulin gamma chain
104795										1.407 up	3.245 up	unique protein
104797												GH3 β-glucosidase BGL3j
104803				+	+	+	+	+	+			carbon-nitrogen hydrolase
104807				+	+	+	+	+	+			unknown protein
104816												MFS permease
104820										1.371 down	2.073 down	unknown protein
104831				+	+	+	+	+	+			unknown protein
104838					+							unknown protein
104842						+				1.005 down	2.241 down	transthyretin-like protein
104856				+	+	+	+	+	+			unknown protein
104867												unique protein
104878				+	+	+	+	+	+			unknown protein
104879				+	+	+	+					unknown protein
104890												4-nitrophenylphosphatase
104898				+	+	+	+	+	+			unknown protein
104907					+							unique protein
104911				+		+			+	1.099 down	2.097 down	unknown protein
104923										1.072 up	3.090 up	unique protein

104925							1.743 up	2.802 down	unknown protein
104927							3.427 up	4.123 up	unique protein
104939									unique protein
104942	+	+	+	+	+	+			unknown protein
104952		+	+	+	+	+			unknown protein
104958	+	+	+		+				CTD kinase subunit gamma. putative
104968							1.229 down	2.768 down	unknown protein
104972	+	+	+		+	+	1.330 down	2.083 down	glutathione synthase
104978	+	+	+	+	+	+			unknown protein
104994									Zn2Cys6 transcriptional regulator
105003							4.968 down	4.113 down	unknown protein
105011									Phosphoribosylformimino--aminoimidazole carboxamide ribonucleotide (ProFAR) isomera
105019									Heat shock protein DnaJ
105026	+	+	+	+	+	+			Nucleolar GTPase/ATPase p130
105027							1.093 up	2.231 up	unknown protein
105031									RecQ subfamily of DNA helicases
105035	+	+	+	+	+	+			3-oxo-5-alpha-steroid 4-dehydrogenase
105051		+							unknown protein
105054	+	+	+	+	+	+			unknown protein
105055	+	+	+	+	+	+			Ptpl Protein tyrosine phosphatase-like protein
105062									SSCRP
105070									MetC Cystathionine beta-lyases/cystathionine gamma-synthases
105072	+	+	+	+	+	+			CE4 polysaccharide deacetylase
105077	+	+	+	+	+	+			pre-mRNA splicing factor ATP-dependent RNA helicase prp16
105079	+	+	+	+	+	+			unknown protein
105081	+	+	+	+	+	+			peflin
105092									unknown protein

105106										1.255 up	6.057 down	unique protein
105133				+	+	+	+	+	+	1.460 up	2.238 up	unknown protein
105143	+	+	+							1.323 up	5.834 up	unique protein
105147				+	+	+	+	+	+			unknown protein
105150												unique protein
105154										1.002 down	2.745 up	unknown protein
105155										1.334 up	4.191 up	unknown protein
105156										1.034 up	10.673 up	unique protein
105157										1.011 up	4.445 up	unknown protein
105158										1.390 up	2.330 down	unknown protein
105165												unique protein
105167						+				1.123 up	2.845 up	unique protein
105171										1.023 up	4.463 up	unknown protein
105173										1.076 up	3.365 up	unknown protein
105174												unique protein
105176												unique protein
105187										1.130 down	3.887 up	unknown protein
105189				+	+	+	+	+	+			20S proteasome beta-type subunit Pup1
105190				+	+	+	+	+	+			unknown protein
105191				+	+	+	+	+	+			DNA-directed RNA polymerase. 30-40 kDa subunit
105192				+	+	+	+	+	+			NEDD8-activating enzyme E1 catalytic subunit
105196					+	+						unknown protein
105202				+	+	+	+	+	+			Arp2/3 complex. 34kDa subunit p34-Arc
105206										1.399 up	3.470 up	unique protein
105216	+	+	+									unknown protein
105220										5.424 down	3.525 down	unknown protein

105221							1.650 down	2.028 up	unknown protein
105222							1.081 up	3.648 up	unique protein
105223							1.179 down	4.591 down	unknown protein
105224									PTH11 GPCR
105233							1.129 down	2.785 up	unique protein
105237									SSCRP
105238									Taurine catabolism dioxygenase TauD/TfdA
105239							1.161 down	2.977 down	Zn2Cys6 transcriptional regulator
105242									SAM-dependent methyltransferases
105246							1.488 up	3.805 up	GH23 exo-ß-1.3-glucanase. distantly related
105247							1.196 up	4.006 up	dipeptidyl peptidase 5
105251							1.185 down	2.724 down	unknown protein
105255							1.609 down	5.298 down	Zn2Cys6 transcriptional regulator
105260	+	+	+	+		+	2.886 down	12.720 down	MFS permease
105263							1.364 down	3.222 down	Zn2Cys6 transcriptional regulator
105269							1.661 down	2.011 down	Zn2Cys6 transcriptional regulator
105275									unknown protein. secreted. contains parallel beta-helix repeat
105279							1.861 down	2.277 up	Leucine aminopeptidase 1
105282									short chain dehydrogenase/reductase
105286	+						1.261 down	2.546 up	unknown protein
105287	+								unknown protein

105288												unknown protein
105290					+					1.631 down	2.336 up	unknown protein
105291	+	+	+	+	+							unknown protein
105311										1.576 up	4.079 up	SSCRP
105313												unique protein
105315				+	+	+	+	+	+			ribosomal protein L15
105328												unknown protein
105330										1.687 down	3.985 up	unknown protein
105336										1.210 up	2.696 up	unknown protein
105338										1.199 up	4.693 up	unknown protein
105342												Iron/ascorbate family oxidoreductases
105346												unknown protein
105349				+	+	+	+	+	+			unique protein
105351				+	+	+	+	+	+			cell cycle control protein cwf8
105356												unknown protein
105363										5.675 down	7.165 down	aldolase/citrate lyase family protein
105379												isochorismatase family protein
105381										1.324 up	2.320 up	unknown protein
105383										1.162 up	3.994 up	unknown protein
105385										1.030 down	2.463 up	unknown protein
105386										1.081 down	2.736 down	unknown protein
105389												unknown protein. 5 TM
105391										1.189 up	4.408 up	unknown protein
105393										1.500 down	2.832 up	unknown protein
105401				+	+	+	+	+	+			unknown protein. secreted

105405							1.174 down	2.990 up	unique protein
105406			+						unique protein
105407	+	+	+	+	+	+			unknown protein
105408	+	+	+	+	+	+			unique protein
105409	+	+	+	+	+	+			regulator of MAPKKK STE50
105420									unknown protein
105423	+	+	+	+	+	+			unknown protein
105432	+	+	+	+	+	+			unknown protein with RING finger SH3 domain
105444							1.549 down	2.658 up	SSCRP
105445							1.048 up	3.096 up	unknown protein. only in Neurospora and A. fumigatus
105447							1.074 down	4.082 up	unknown protein
105448							1.215 up	5.686 up	CBM 13
105449							1.020 up	3.699 up	Cyclin C-dependent kinase CDK8
105454									SSCRP
105455							3.385 down	2.493 down	unknown protein
105457							1.195 up	2.697 up	unknown protein
105458									C2H2 transcription factor
105465									unknown protein
105466									unknown protein
105467							1.053 up	3.546 up	unknown protein
105475	+	+	+	+	+	+			Zn2Cys6 transcriptional regulator
105479	+	+	+	+	+	+			ribosomal protein L23
105483									unique protein
105488		+					1.162 up	2.099 up	unknown protein
105504	+	+	+	+	+	+			Alkaline phosphatase. putative
105514							23.554 down	2.801 down	unique protein

105515							2.915 up	5.259 up	unique protein
105518							2.552 down	6.245 down	K+ channel
105520									Zn2Cys6 transcriptional regulator
105524									unique protein
105532							1.695 down	2.322 up	unique protein
105533	+								SSCRP
105535	+	+	+	+	+	+			unknown protein
105537							1.051 up	2.108 down	unknown protein
105538									unknown protein
105540							1.033 down	2.368 up	unique protein
105553							1.363 up	8.518 up	unique protein
105556							1.445 up	4.205 up	unique protein
105557	+	+	+	+	+	+			GT32 α-glycosyltransferase
105565									MFS permease
105589	+	+	+	+	+	+			mitochondrial ATPase inhibitor. putative
105593	+	+	+	+	+	+			unknown protein
105597	+	+			+		1.101 up	2.615 down	unique protein
105613			+						unknown protein
105617									unknown protein
105623	+	+	+	+	+	+			ATP synthase subunit gamma precursor
105628			+						unknown protein
105631			+						unknown protein
105636		+	+						unknown protein
105643							1.135 down	2.688 down	myb transcriptional regulator
105647	+	+	+	+	+	+			unknown protein

105650												unknown protein
105652										1.273 up	3.408 up	unknown protein
105676				+	+	+	+	+	+	2.252 up	2.303 up	DNA replication licensing factor mcm7
105682												unknown protein
105692				+	+	+	+	+	+			Golgi reassembly stacking protein GRASP65
105701												unknown protein
105707						+				1.072 up	3.169 up	unknown protein
105709												unknown protein
105710												unique protein
105718										1.979 down	7.431 down	unknown protein
105722	+	+	+							17.360 down	4.027 down	unique protein
105729										1.012 up	2.767 up	unknown protein
105748												unique protein
105752										1.018 up	11.330 up	C4-dicarboxylate transporter/malic acid transport protein
105763				+	+			+		1.649 up	4.233 down	HFBs
105765										1.009 up	4.102 down	unknown protein
105768										1.049 down	2.085 up	Cytochrome P450
105771				+	+	+	+		+			unknown protein
105775	+	+	+							2.536 down	2.195 down	unknown protein
105784										1.815 down	2.811 down	Zn2Cys6 transcriptional regulator
105785										1.423 up	4.402 up	unknown protein
105787												unknown protein
105788												aspartyl protease
105798										1.120 up	2.659 up	MFS permease

105804							1.193 down	3.352 up	PKS
105805							1.272 up	4.632 up	Zn2Cys6 transcriptional regulator
105806							1.436 down	3.782 up	unknown protein
105808									unknown protein
105810	+	+	+	+	+	+			Peptidase M4. thermolysin. putative
105816									unknown protein
105820							1.237 up	2.642 up	unknown protein
105823							27.139 down	7.263 down	FAD binding domain protein
105832	+	+	+	+	+	+	1.403 down	2.434 down	phosphoribosylaminoimidazole carbounknown proteinlase
105834							1.110 up	3.049 down	Zn2Cys6 transcriptional regulator
105838							1.160 down	2.210 up	glyoxalase
105840			+				9.559 down	2.071 up	unknown protein. 2 TMs
105843									unknown protein
105844	+	+		+		+			SSCRP
105847									unique protein
105848									unknown protein
105849							1.424 down	4.587 down	Zn2Cys6 transcriptional regulator
105851							1.232 down	2.375 down	GMC oxidoreductase family protein
105854		+	+						unknown protein
105860									unknown protein
105863							1.395 up	2.300 down	unknown protein
105864									PKS-NRPS

105866							1.135 down	2.615 down	unique protein
105867							1.058 up	3.986 up	unique protein
105868									unknown protein
105870									Aquaporin (major intrinsic protein family)
105874							1.051 up	2.636 down	FAD binding domain-containing protein
105880									Zn2Cys6 transcriptional regulator
105882									unknown protein
105884							1.120 up	3.214 up	short chain dehydrogenase/reductase
105888							1.847 up	4.222 up	short chain dehydrogenase/reductase
105891	+	+	+	+	+	+			serine-threonine kinase receptor-associated protein
105894							1.149 up	9.027 up	unknown protein
105904									unknown protein
105911									unknown protein
105912							1.082 down	2.348 up	unknown protein
105917	+	+	+	+	+	+			unknown protein
105924									GT1 ß-glycosyltransferase
105931									GH20 N-acetyl-β-hexosaminidase
105938	+	+	+	+	+	+			pre-mRNA splicing factor slt11
105956									GH13 α-amylase
105968	+	+	+	+	+	+			Iron/ascorbate family oxidoreductases
105969									unknown protein
105970									short chain dehydrogenase/reductase
105975									unknown protein
105977							1.379 up	2.139 up	unknown secreted protein. 3 TM
105978							1.676 up	2.003 down	ARO8. Transcriptional regulators containing a DNA-binding HTH domain and an aminotran
105979									Zn2Cys6 transcriptional regulator
105980									Zn2Cys6 transcriptional regulator

105983										1.085 up	2.706 up	unknown protein
105984				+	+	+						SSCRP
105989										2.653 up	2.578 up	Zn2Cys6 transcriptional regulator
105994				+	+	+	+	+	+			unknown protein
106009										1.133 down	2.063 down	Zn2Cys6 transcriptional regulator
106014				+	+	+	+	+	+			Sorting Nexin Snx3
106016												unknown protein
106018				+	+	+	+	+	+	2.107 up	2.312 up	unknown protein
106023				+	+	+						unknown protein
106024					+					1.429 up	3.875 up	unknown protein
106029				+	+	+				1.994 down	4.616 down	MFS permease
106035				+		+						unknown protein
106037				+	+	+	+	+	+			unknown protein
106039				+	+	+	+	+	+			Ribosomal protein S28 based on homology to the corresponding protein of Neurospora cr
106041	+	+	+							1.216 up	3.105 up	unique protein
106043				+	+	+				3.842 down	4.265 down	unknown protein. GFA-domain
106048				+	+	+	+	+	+			C2H2 transcriptional regulator
106049												unknown protein. only in Hypocreaceae
106051				+	+	+	+	+				unknown protein
106055												unknown protein
106064					+							C2H2 transcriptional regulator
106067				+	+	+	+	+	+	1.540 down	3.028 up	Chaperonin Cpn10
106081				+	+	+	+	+	+			unknown protein
106082				+						1.358 down	2.813 down	Epl1/Sm1
106089										1.823 down	5.089 down	unique protein

106107							1.429 up	2.523 up	unknown protein
106108	+	+	+	+	+	+			unknown protein
106111	+	+	+						unique protein
106113	+	+	+	+	+	+			Vesicle coat complex COPI. gamma subunit
106116							2.227 up	3.324 up	unknown protein
106118							1.221 down	2.479 down	Inorganic phosphate transporter
106120			+				19.812 up	13.457 up	esterase/lipase
106129	+	+	+				1.554 down	3.547 down	unknown protein. only present in ascomycota
106130	+	+	+	+	+	+	1.346 down	2.843 down	unknown protein
106131									unknown protein
106138							2.081 down	3.340 down	SNF2 family DNA-dependent ATPase
106145	+	+	+	+	+	+			unknown protein
106147	+	+	+	+	+	+			unknown protein
106148		+							unknown protein
106150	+	+	+	+		+			Coenzyme Q (ubiquinone) biosynthesis protein Coq4. putative
106151							1.136 up	7.677 up	unknown protein
106152							1.488 up	2.079 up	unknown protein
106154		+					1.368 up	2.699 down	unknown protein
106155		+							unknown protein
106160									unknown protein. secreted
106161									Ankyrin
106164		+	+		+		6.740 down	2.623 up	short chain dehydrogenase/reductase
106168							1.325 up	3.414 up	SSCRP
106171							2.821 down	3.232 up	HET protein

106178												unknown protein
106181					+					1.265 up	11.629 down	unique protein
106205				+	+	+	+	+	+			ubiquitin carboxyl-terminal hydrolase 2. putative
106219										1.617 up	3.010 up	unknown protein
106223				+	+	+	+		+	2.658 down	6.040 down	unique protein
106229				+	+	+						unique protein
106236				+	+	+	+	+	+			unique protein
106237				+	+	+	+	+	+			unknown protein
106242										1.424 down	2.404 down	unique protein
106244												Zn2Cys6 transcriptional regulator
106245										1.665 down	4.117 down	catalase
106248						+				1.696 up	3.534 up	monosaccharide transporter (galactose permease ?)
106249				+	+	+	+	+	+			unknown protein
106250				+	+	+	+	+	+			Glutamine amidotransferase. class-II
106251												unique protein
106252	+		+									unknown protein
106253	+	+	+									unique protein
106258												unknown protein
106259				+								Zn2Cys6 transcriptional regulator
106267	+		+							1.081 up	3.031 up	unique protein
106270										46.214 down	6.817 down	unknown protein
106272				+	+	+	+	+	+	1.455 down	2.574 down	PKS
106274				+	+	+	+	+	+			unique protein
106276					+					1.087 down	3.132 down	initiator tRNA phosphoribosyl transferase.

106283	+	+	+	+	+	+			unknown protein
106287	+	+	+	+	+	+			unknown protein
106294	+	+	+						unknown protein. only in Magnaporthe. Neurospora and Chaetomium
106296									unknown protein
106297							1.393 up	2.036 down	Amino acid transporters
106306	+	+	+	+	+	+			unknown protein
106308	+	+	+	+	+				unknown protein
106309	+	+	+	+	+	+			unknown protein
106314							1.306 up	6.595 down	Ankyrin
106315							2.271 up	4.033 up	serine protease
106321	+	+	+	+	+	+			unknown protein
106330			+						MFS permease
106331	+	+	+	+	+	+			TRAPP complex component Trs120
106337	+	+	+	+	+	+			unknown protein
106340									unique protein
106342	+	+	+	+	+	+			unknown protein
106353									unknown protein
106356							1.240 down	2.196 down	unknown protein
106357	+	+	+	+	+	+			unknown protein
106362									unknown protein
106370	+	+	+	+	+	+			unknown protein
106371							8.617 down	2.322 down	SSCRP
106378									unknown protein
106391							1.062 down	3.506 down	unknown protein
106405							1.978 up	3.158 up	extracellular lipase-like protein
106429		+							unknown protein

106437										1.356 down	2.581 down	unknown protein
106444												unique protein
106445										1.126 down	6.337 up	unique protein
106450												unique protein
106452										3.258 down	4.639 down	unknown protein
106453										1.138 up	3.756 up	SSCRP
106457												unknown protein
106467												unknown protein
106470	+	+	+							1.047 down	2.278 up	unique protein
106471	+	+	+	+	+	+	+	+	+			unknown protein
106473				+	+	+	+	+	+			TATA box binding protein associated factor (TAF)
106475												unknown protein
106476												Zn2Cys6 transcriptional regulator
106479										1.156 up	3.527 up	unknown protein
106480										2.327 up	6.820 up	flavoprotein monooxygenases
106482												unique protein
106484												SSCRP
106486										1.074 up	3.471 up	unique protein
106487												Protein kinase
106488										1.475 down	3.615 down	unknown protein
106490										1.188 up	2.925 up	unknown protein. 3 TM. only in Neurospora and Chaetomium
106491										2.986 down	5.048 down	unknown protein
106492										1.077 up	3.370 up	SSCRP
106493										1.085 up	2.974 up	unknown protein
106494												unknown protein

106499							1.377 down	2.433 up	unknown protein
106512									unique protein
106516									short chain dehydrognease/reductase
106531	+	+	+	+	+	+			GTP-binding protein GTR1
106533	+	+	+	+	+	+			Rab GTPase interacting factor Yip1
106537									unique protein
106538							7.440 up	10.140 up	HFB4
106539									unknown protein
106554									SSCRP
106556							1.458 down	3.185 up	unknown protein
106564	+	+	+				1.032 up	2.475 up	unique protein
106569		+	+						unknown protein
106570	+	+	+	+	+	+			unknown protein
106575			+						GH79 β-glucuronidase
106576									unknown protein. 2 TM
106583							1.121 down	3.225 up	unique protein
106584									unique protein
106586	+	+	+						unknown protein
106591	+					+			unique protein
106595	+	+	+	+	+	+			Signal recognition particle. subunit Srp54
106603	+	+	+	+	+	+			Ras small GTPase.Rho Type;Related to S. pombe Septum promoting GTP binding protein 1
106604							1.092 up	2.819 up	unique protein
106605		+	+						unknown protein
106606									unknown protein
106607									unknown protein
106615									SSCRP
106617	+	+	+	+	+	+			NADH:ubiquinone oxidoreductase 17.2 kD subunit

106623									unknown protein
106624									unique protein
106625									unknown protein
106626									unknown protein. SWIFT domain
106627									unknown protein
106636									unknown protein
106640	+	+	+	+	+	+			translation initiation factor 3.
106641	+	+	+	+	+	+			unknown protein
106642	+	+	+	+	+	+			unknown protein
106645									unknown protein
106646			+						unknown protein
106647									unique protein
106649									3-hydroxyacyl-CoA dehydrogenase. NAD binding domain. putative
106654							1.352 down	4.265 down	Zn2Cys6 transcriptional regulator
106657							1.546 down	2.603 down	Zn2Cys6 transcriptional regulator
106659							1.329 down	5.170 down	unknown protein
106660									unknown protein
106661							1.727 down	2.889 up	aspartyl protease
106662							1.038 up	2.307 up	SSCRP
106676									unknown protein. only in Gibberella
106677							3.067 down	3.773 down	Zn2Cys6 transcriptional regulator
106680	+	+	+	+	+	+			Nop10p family Nucleolar RNA-binding protein
106684							1.611 up	3.536 up	unknown protein
106686			+				2.277 down	4.800 up	unknown protein

106695							8.454 down	2.670 down	Sulfite oxidase. molybdopterin-binding component
106697							2.150 down	3.088 down	esterase/lipase. HGT
106706							1.414 down	7.936 down	Zn2Cys6 transcriptional regulator
106720									Zn2Cys6 transcriptional regulator
106730	+	+	+	+	+	+			vacuole-associated enzyme activator complex component (Vac14)
106731	+	+	+	+					unknown protein
106743	+	+	+						unknown protein
106744	+	+	+	+	+	+			unknown protein
106755		+							unknown protein
106761	+	+	+	+	+	+			autophagy-related protein 9 . putative
106769	+	+	+	+	+	+			DNA repair protein Rad18
106781									unknown protein
106797									unknown protein
106798			+				3.762 up	4.706 up	GCN5-N-acetyltransferase
106801							1.007 up	2.007 down	unknown protein
106812	+	+	+	+	+	+	1.106 up	4.957 up	unique protein
106818	+	+	+	+	+	+			unknown protein
106821									unknown protein
106828							1.297 up	2.124 down	unknown protein
106829									GCN5-N-acetyltransferase
106834	+	+	+	+	+	+			unknown protein
106868									unknown protein
106869							1.737 up	3.537 up	unknown protein
106874	+	+	+	+	+	+			unknown protein
106877									unknown protein
106879									unknown protein. secreted

106885												acyl-CoA dehydrogenase
106895												unique protein
106897	+	+	+							1.367 up	2.386 up	unique protein
106900												unique protein
106928										1.027 up	4.367 up	Na/K ATPase alpha 1 subunit.
106936												ferric reductase
106939										4.556 up	2.534 up	protein kinase
106947										1.833 up	3.657 up	unique protein
106951				+	+	+	+	+	+			unknown protein
106958				+	+	+				1.115 up	2.764 down	unique protein
106960										1.109 down	4.269 up	unknown protein
106962				+	+	+	+	+	+			RNA helicases and translation initiation factor eIF4a subunit. Homologue of yeast TIF2.
106981				+	+	+	+	+	+			unknown protein
106982				+	+	+	+	+	+			unknown protein
106998										1.098 down	2.488 up	unknown protein
107002										1.448 down	3.478 down	unknown protein
107003										1.757 down	4.165 up	unknown protein
107005				+	+	+	+	+	+			Guanine nucleotide exchange factor
107007										1.101 down	2.283 up	SSCRP
107008										1.208 up	4.930 up	unknown protein
107011				+	+	+	+	+	+			unknown protein
107012				+	+	+						unknown protein
107014												unknown protein
107031										1.255 down	2.560 up	unique protein

107032		+							unknown protein
107035	+	+	+	+		+			RAS small GTPase.Ras subfamily
107037							2.634 up	3.940 up	unknown protein
107042							2.094 up	2.118 up	PTH11 GPCR
107045	+	+	+	+	+	+			FHA domain protein SNIP1
107055	+	+	+	+	+	+	1.572 down	6.580 down	unknown protein
107058		+							unknown protein
107068									Suppressor of meioticSilencing
107070							1.036 down	2.653 up	unique protein
107071							1.412 up	3.767 up	HET-domain protein. unknown unknown
107072									unknown protein
107073			+						Ca2+/calmodulin-dependent protein kinase. EF-Hand protein superfamily
107076									unique protein
107078	+	+	+	+	+	+			unknown protein
107082	+	+	+	+	+	+			unknown protein
107107							1.193 down	2.243 up	unknown protein
107111	+	+	+	+	+	+	1.258 down	3.064 down	unknown protein
107112			+				1.851 up	2.498 up	unique protein
107123	+	+	+	+	+	+	1.913 up	2.239 up	mitochondrial import inner membrane translocase subunit tim16
107124	+	+	+	+	+	+			GTP-binding protein
107131	+	+	+	+	+	+	1.657 up	2.838 up	unknown protein
107132	+	+	+	+	+	+			unknown protein
107137	+	+	+	+	+	+	1.151 down	2.205 down	sterol desaturase family
107140	+	+	+	+	+	+			unknown protein
107141									unique protein
107142							1.980 up	2.709 up	neutral protease 2

107144												unknown protein
107146							+			1.009 up	2.363 up	unknown protein
107151				+	+	+	+	+	+			unknown protein
107158				+	+	+	+	+	+			unknown protein
107168					+							unknown protein
107172												2.2-dialkylglycine decarboxylase. AIB forming ?
107185												unknown protein
107187					+							unknown protein
107194				+	+	+	+	+	+	2.057 up	2.995 up	unique protein
107197	+	+	+									unknown protein
107202						+				3.857 down	2.407 down	unknown protein
107205				+	+	+	+	+	+			inositol phospholipid synthesis protein Scs3p. putative
107206												ATP adenylyltransferase. putative
107207										1.338 up	2.270 up	HMG box-containing protein
107208										1.057 up	2.665 up	unique protein
107217										6.515 down	2.752 down	unknown protein
107218												unique protein
107229												unique protein
107239				+	+	+	+	+	+	2.637 up	2.160 up	microtubule protein BIM1. possibly involved in mating. karyogamy or meiosis
107244				+	+	+	+	+	+			unknown protein
107245				+	+							unknown protein
107253												unknown protein
107255												ADP-ribosylation factor-like protein 2. Ras small GTPase
107258				+	+	+	+	+	+	1.087 up	2.331 down	1-acyl-sn-gylcerol-3-phosphate acyltransferase (Lysophosphatidic acid acyltransferase)
107260				+	+	+	+	+	+	1.017 down	2.150 down	unknown protein
107263				+	+	+	+	+	+			phospholipase of papatin-family

107268										1.302 down	2.148 down	Esterase/lipase/thioesterase
107278												unique protein
107279												unknown protein
107282				+	+	+	+	+	+			unknown protein
107285	+	+	+									
107294										1.177 down	2.568 down	RTA1-like protein. 7 TM. responds to xenobiotic stimuli
107297										3.011 down	11.076 down	unique protein
107298												unique protein
107300				+	+	+	+	+	+			unique protein
107306				+	+	+				1.201 up	2.031 down	unique protein
107323												short chain dehydrogenase/reductase
107325				+	+	+	+	+	+			unknown protein
107329										1.361 down	3.984 up	methionine synthase
107335				+	+	+	+	+	+			Ribosomal protein L10.
107338												unique protein
107339												unique protein
107340				+	+	+	+	+	+	2.102 down	5.791 down	unknown protein
107346	+	+	+									unknown protein
107347	+	+	+							2.995 up	2.418 up	SSCRP
107349										1.248 up	2.496 up	unknown protein
107357												unique protein
107358										1.150 down	2.227 up	unique protein
107360												unknown protein
107369				+	+	+	+	+	+	2.688 down	3.284 down	guanine nucleotide exchange factor-like protein

107374									unknown protein
107383									arginine N-methyltransferase skb1
107385	+	+	+	+	+	+			ATP-dependent helicase Prp16
107386							1.032 up	2.794 up	unknown protein. only in Gibberella and Neurospora
107389	+	+	+	+	+	+			unknown protein
107402	+	+	+	+	+	+			guanylate kinase
107420	+	+	+	+	+	+			unknown protein
107436	+	+	+	+	+	+			unknown protein
107445	+	+	+	+	+	+	1.087 up	3.595 up	unknown protein
107460	+	+	+	+	+	+			unknown protein
107463	+	+	+	+	+	+	2.112 down	2.059 down	exoribonuclease Dhp1
107464	+	+	+	+	+	+	1.179 up	11.473 up	unknown protein
107469									unique protein
107475							1.673 down	3.812 down	unknown protein
107479									ZIP zinc/iron transporter
107483	+	+	+	+	+	+	1.037 up	2.775 down	unknown protein
107488							1.111 up	2.913 up	unknown protein
107490	+	+	+	+	+	+			unknown protein
107494							8.068 down	4.350 down	unique protein
107495							25.930 down	2.009 down	unknown protein
107502									unknown protein
107503	+	+	+	+	+	+			unknown protein
107507							8.541 up	3.427 up	unknown protein
107509		+	+	+	+	+			autophagy protein 16. putative
107513							1.801 up	2.031 up	unique protein
107523									unknown protein

107524	+	+	+				1.048 down	2.835 down	unique protein
107525									unknown protein. only in fungi
107526							1.014 down	2.315 up	short chain dehydrognease/reductase
107541									myb transcriptional regulator
107547									unknown protein
107551	+	+	+	+	+	+			unknown protein
107552	+	+	+	+	+	+			unknown protein
107554	+	+	+	+		+	1.815 down	2.036 down	glutathione transferase
107564	+	+	+	+	+	+	2.034 up	2.146 up	Sin3-associated polypeptide Sap18
107567	+	+	+	+	+	+			unknown protein
107571	+	+	+	+	+				unknown protein
107577	+	+	+	+	+	+			unknown protein
107591	+	+	+	+	+	+			unknown protein
107595							1.072 up	2.779 up	unique protein
107596									unique protein
107601	+	+	+	+	+	+	1.169 down	2.275 up	unknown Zn-finger protein. Tim10/DPP type
112695							1.011 up	2.453 up	SSCRP
119534	+	+	+	+	+	+	1.220 down	2.526 down	unique protein
119548	+	+	+	+	+	+			mitotic checkpoint protein BUB3
119551	+	+	+	+	+	+			mitochondrial carrier protein LEU5
119552	+	+	+	+	+	+	1.189 up	2.725 up	unique protein
119554	+	+	+	+	+	+			rRNA processing protein Bystin
119555									unknown protein
119556							2.752 down	2.620 down	unknown protein. secreted

119568	+	+	+	+	+	+	1.206 down	3.590 down	unknown protein
119576									unknown protein. only present in ascomycota and Streptomyces
119578	+	+	+	+	+	+			Cargo transport protein Erv29
119592	+	+	+	+	+	+			High affinity Ca2+/Mn2+ P-type ATPase (Golgi)
119593	+	+	+	+	+	+			Has homology to tubuling binding cofactor A.
119600	+	+	+	+	+	+			Oligosaccharyltransferase. alpha subunit
119603									methylthioribulose-1-phosphate dehydratase
119607							1.762 down	2.907 down	unknown protein
119609	+	+	+	+	+	+			Phospho-2-dehydro-3-deoxyheptonate aldolase
119614	+	+	+	+	+	+			CAMP dependent serine/threonine protein kinase related to S.Cerevisiae Sch9
119616									Serine/threonine protein kinase
119619							4.165 down	2.780 down	unknown protein
119620							1.343 down	2.295 down	Zn2Cys6 transcriptional regulator
119623			+						tRNA pseudouridine synthase D.
119624									unknown protein
119626	+	+	+	+	+	+			40s ribosomal protein S22 (S8 family) by homology to the corresponding protein of N. cras
119633	+	+	+	+	+	+			unknown protein
119638	+	+	+	+	+	+			unknown protein
119642	+	+	+	+	+	+			unknown protein
119645	+	+	+						unknown protein
119651	+	+	+	+	+	+			ribosomal protein L16
119658	+	+	+	+	+	+			unknown protein
119664	+	+	+	+	+	+			ubiquitin carboxyl-terminal hydrolase. putative
119679		+							calcium/calmodulin dependent protein kinase C. related to E. nidulans cmkC
119681	+	+	+	+	+	+			Component. Pan1. of actinCytoskeleton-regulatoryComplex Pan1p-Sla1p-End3p. involved
119690	+	+	+	+	+	+			unknown protein
119694	+	+							unknown protein

119695									RNA:NAD 2'-phosphotransferase TPT1.
119696	+	+	+	+	+	+			lipase. extracellular
119697	+	+	+	+	+	+			Tyrosine specific and dual specificity protein phosphatase. containsTonB box. N-terminal
119704	+	+	+	+	+	+			unknown protein
119707	+	+	+	+	+	+			calponin-like actin binding domain
119710	+	+	+				3.821 down	4.284 down	MFS multidrug transporter
119721	+	+	+	+	+	+			U4/U6.U5 tri-snRNP-associated protein snu66
119723	+	+	+	+	+	+	1.044 down	2.050 down	NSF attachment protein Sec17/alpha-SNAP
119724	+	+	+	+	+	+			unknown protein
119731	+	+	+	+	+	+			heat shock protein hsp60 mitochondrial precursor protein
119732									unknown protein
119735	+	+	+	+	+	+			Glyceraldehyde-3-phosphate dehydrogenase. isozyme 2
119739	+	+	+	+	+	+			unknown protein
119742	+	+	+	+	+	+			Vesicle coat complex COPII. Sec31
119749	+	+	+	+	+	+			Signal recognition particle. subunit Srp19
119757	+	+	+	+	+	+			RNA splicing factor Pad-1
119759	+	+	+	+	+	+			BZIP transcriptional regulator
119762	+	+	+	+	+	+			key endocytic protein. Ede1. involved in a network of interactions with other endocytic pr
119764	+	+	+						unknown protein
119767									alpha-tubulin cofactor B.
119768	+	+	+	+	+	+			myb transcriptional regulator
119779									unknown protein
119788	+	+	+	+	+	+			saccharopine dehydrogenase
119789	+	+	+	+	+	+	1.369 down	2.762 down	MFS permease
119790									myo-inositol oxygenase .
119792	+	+	+	+	+	+			unknown protein
119796									unknown protein

119797	+	+	+	+	+	+			SSCRP
119800	+	+	+	+	+	+	1.953 up	2.000 up	unknown protein
119803		+							Meiosis-specific protein. required for spore wall formation during sporulation
119805	+	+	+	+		+	1.232 up	2.096 up	HFBs
119806	+	+	+				1.723 up	2.677 up	unknown protein. only in Gibberella. Magnaporthe and Chaetomium
119812	+	+	+	+	+	+			unknown protein
119813	+	+	+	+	+	+			unknown protein
119815	+	+	+	+	+	+			unknown protein
119816	+	+	+	+	+	+	1.562 up	2.392 up	unknown protein
119819		+							GCPR. mPR-type
119822	+	+	+	+	+				Serine/threonine protein kinase
119823							1.635 down	2.686 down	SAM-dependent methyltransferases
119825							1.242 up	3.237 up	unknown protein
119826							2.461 down	18.872 down	C2H2 transcriptional regulator
119830	+	+	+	+	+	+			kinesin-like protein
119839							1.805 down	2.966 up	cyanamide hydratase
119844									unknown protein
119845	+	+	+	+	+	+			mitochondrial oxaloacetate/sulfate/thiosulfate transporter-like protein
119848	+	+	+	+	+	+			DEAD helicases superfamily protein (Aquarius). putative
119850	+	+	+	+	+	+			unknown protein
119854	+	+	+	+	+	+			myotubularin (protein phosphatase)
119855	+	+	+	+	+	+			40s ribosomal protein
119856	+	+	+	+	+	+	1.007 down	2.034 down	E3 ubiquitin-protein ligase/Putative upstream regulatory element binding protein
119857							21.231 up	8.567 up	unique protein
119859									GH18. chitinase CHI18-13
119860							1.423 up	2.222 down	unknown protein

119864							9.027 down	4.933 down	unknown protein
119867									unknown protein
119871	+	+	+	+	+	+			Ras GTPAse of Rho subfamily
119875									acyl-CoA thioesterase
119876	+	+	+				1.103 up	2.027 up	aspartyl protease
119879	+	+	+	+	+	+			Rab geranylgeranyl transferase escort protein. putative
119881	+	+	+	+	+	+			unknown protein
119887	+	+	+	+	+	+			unknown protein
119890									Protein disulfide isomerase TigA
119895	+	+	+	+	+	+	1.040 up	2.374 down	unknown protein
119896	+	+	+	+	+	+	1.306 down	3.683 down	malic enzyme
119898	+	+	+	+	+	+			ribosome biogenesis GTPase Lsg1
119902	+	+	+	+		+	4.795 down	2.418 down	unique protein
119903	+	+	+	+		+			Der1. and ER membrane protein involved in ER-associated protein degradation.
119924	+	+	+	+	+	+			Hsp70 nucleotide exchange factor FES1. putative
119931	+	+	+	+	+	+			fatty acid elongase
119933	+	+	+	+	+	+			unknown protein
119940	+	+	+						unique protein
119943	+	+	+	+	+	+			unknown protein
119947	+	+	+	+		+			unknown protein
119954	+	+	+	+	+	+			porphobilinogen synthase-like protein
119956	+		+	+		+			unknown protein
119960							1.254 up	2.295 up	unique protein
119963	+		+	+		+	1.202 up	2.031 up	HFBs
119965	+	+	+	+	+	+			unknown protein
119972	+	+	+	+	+	+			unknown protein
119974									zinc-binding dehydrogenase. putative

119975	+	+	+	+	+	+			SSCRP
119980	+	+	+	+	+	+			unknown protein
119981	+	+	+	+	+	+			unknown protein. vacuolar membrane
119983	+	+	+	+	+	+			unknown protein
119986	+	+	+	+	+	+			Zn2Cys6 transcriptional regulator
119989							1.034 down	2.015 up	HFB2
119991	+	+	+	+	+	+			Unknown protein with a putative zinc finger binding motif
119999	+		+						Myb-DNA binding domain protein
120000									unknown protein
120004	+	+	+	+	+	+			ankyrin repeats
120008							2.084 up	2.017 up	GMC oxidoreductase
120012	+	+	+	+	+	+			60S ribosomal protein L24
120017	+	+	+	+		+			oligopeptide transporter
120020	+	+	+	+	+	+			ribosomal protein L37e from several filamentous fungi and yeasts.
120030									unique protein
120031							2.450 up	3.009 up	unknown protein
120035	+	+	+						unknown protein
120044	+	+	+	+	+	+			GDP dissociation inhibitor Gdi1
120051	+	+	+	+	+	+			Signal recognition particle. subunit Srp68
120053	+	+	+	+	+	+			Hsp70 family protein
120057	+	+	+	+	+	+			unknown protein
120058	+								L-2-hydrounknown proteinglutarate dehydrogenase
120059	+	+	+		+	+			unknown protein
120060	+	+	+	+	+	+	1.112 down	2.008 down	unknown protein with pleckstrin like domain
120064	+	+	+	+	+	+			DEAD box helicase Hel1
120067	+	+	+	+	+	+			Cue1 domain protein involved in ER-associated protein degradation. Distantly related to [illegible]
120070	+	+	+	+	+	+			unknown protein
120075	+	+	+	+	+	+			Ubiquitin-conjugating enzyme (E2)

120079	+	+	+	+	+	+	1.595 down	2.608 down	mitochondrial acetoacetyl-CoA thiolase-like protein
120081	+	+	+	+	+	+			NMD3 family protein (nonsense-mediated mRNA decay protein)
120086	+	+	+	+	+	+			unknown protein
120088	+	+	+	+		+	1.302 up	3.685 up	cytosolic asparaginyl-tRNA synthetase. required for protein synthesis. catalyzes the specif
120106	+	+	+	+	+	+	1.999 up	2.648 up	Isopentenyl diphosphate:dimethylallyl diphosphate isomerase
120110	+	+	+	+	+	+			unknown protein
120113							1.444 down	2.062 down	unknown protein
120114		+	+						ABC transporter
120117	+	+	+	+	+	+			C2H2 transcriptional regulator
120120			+						GCN5-related N-acetyltransferase
120124			+						unknown protein
120125	+	+	+	+	+	+	1.277 down	2.433 down	unknown protein
120127	+	+	+	+	+	+			transcriptional regulator GATA-type zinc finger protein ASD-4 (ascospore development pr
120140									metallophosphoesterase domain-containing protein
120142	+	+	+	+	+	+			unknown protein
120143	+	+	+	+	+	+			chaperone DnaJ
120144	+	+	+	+	+	+			Heat shock protein DnaJ
120147	+	+	+	+	+	+			deounknown proteinhypusine hydrounknown proteinlase
120150	+	+	+	+	+	+			RAS like small GTPase. Ras subfamily
120152	+	+	+	+	+	+			unknown protein
120153	+	+	+	+	+	+			26S proteasome regulatory complex subunit Rpn1
120154	+	+	+	+	+	+			NADPH cytochrome P450 oxidoreductase
120156	+	+	+	+	+	+			cell morphogenesis protein PAG1
120160	+	+	+	+	+	+	1.428 up	2.378 up	unknown protein
120172	+	+	+	+	+	+	1.285 down	2.139 down	flavodoxin and radical SAM domain protein
120173	+	+	+	+	+	+			Protein import receptor MAS20
120176	+	+	+	+	+	+			unknown protein

120184	+	+	+	+	+	+			v-SNARE Ykt6; trafficking to and within Golgi. endocytic trafficking to vacuole. vacuolar fus
120189	+	+	+	+		+			HFBs
120193									unknown protein
120195	+	+	+						unknown protein
120198	+	+	+	+	+	+	1.323 down	2.604 down	glycosyl transferase. family 35. glycogen phosphorylase 1
120206	+	+	+	+	+	+			unknown protein
120215		+							unknown protein
120219	+	+	+	+	+	+			Chorismate_mutChorismate mutase aligned
120223	+	+	+	+	+	+			unknown protein
120224									C2H2 transcriptional regulator
120228									Zn2Cys6 transcriptional regulator
120229							4.392 down	67.461 up	GH10 endo-ß-1.4-xylanase XYN3
120231									NACHT domain WD40 repeat-containing protein
120235	+	+	+	+	+	+			Elongation factor 2
120236	+	+	+	+	+	+			unknown protein
120238	+	+	+	+	+	+			GPCR. related to NCU07701 (Neurospora)
120248							2.296 down	2.051 down	unknown protein
120257	+	+	+						unknown protein
120260	+	+	+	+	+	+	1.219 up	2.267 up	ribosomal protein L24e. putative
120267	+	+	+						ribose-5-phosphate isomerase
120272	+	+	+	+	+	+	1.138 down	2.083 down	64 kDa mitochondrial NADH dehydrogenase. putative
120276	+	+	+	+	+	+			unknown protein
120288	+	+	+						short chain dehydrogenase/reductase
120290	+	+	+	+	+	+			thiamine pyrophosphokinase
120294	+	+	+	+	+	+	1.606 up	2.216 up	unknown protein
120300	+	+	+	+	+	+			Electron transfer flavoprotein. alpha subunit

120311							3.486 down	4.803 up	unique protein
120312							17.338 down	15.462 up	GH5 endo-β-1.4-glucanase EGL2/CEL5a
120313									unknown protein
120318									unknown protein
120320									unknown protein
120326							1.472 down	4.129 down	unknown protein
120332	+	+	+	+	+	+			unknown protein
120337	+	+	+	+	+	+			kinetochore protein NUF2
120339									Histone acetyltransferase (MYST family)
120351	+	+	+	+	+	+	1.691 up	2.020 up	unknown protein
120354	+	+	+	+	+	+			unknown protein
120357									Zinc-binding oxidoreductase
120359									unknown protein
120362									unique protein
120363							1.151 up	2.172 down	C2H2 transcriptional regulator
120365							2.044 down	2.434 down	BZIP transcriptional regulator
120370							1.519 up	2.024 up	unknown protein
120371							161.771 down	8.375 down	Catalase
120376	+	+	+			+			Proteasome component Ecm29
120378	+	+	+	+	+	+			isocitrate dehydrogenase subunit IDH1. NAD-specific
120381							2.719 up	6.564 down	unique protein
120384	+	+	+	+	+	+			unknown protein
120385	+	+	+	+	+	+			unknown protein
120388	+	+	+	+	+	+			TRAPP complex component Trs130

120391	+	+	+	+	+	+			unknown protein
120394	+	+	+	+	+	+			T-complex protein 1
120402	+	+	+	+	+	+			tubulin-tyrosine ligase
120404	+	+			+				SIR2 protein
120407	+	+	+	+	+	+			unknown protein
120411	+	+	+	+	+	+			unknown protein
120412	+	+	+	+	+	+			ARF-GAP effector
120414	+	+	+	+	+	+			unknown protein
120415							4.687 up	2.154 up	unknown protein
120416	+	+	+	+	+	+			unknown protein
120418	+	+	+	+	+	+			unknown protein
120420	+	+	+	+	+	+			unknown protein
120424									Glyoxalase I
120428	+	+	+	+	+	+			C2H2 transcriptional regulator
120430	+	+	+	+		+			Zn2Cys6 transcriptional regulator
120432	+	+	+	+	+	+			unknown protein
120447	+	+	+	+	+	+			Rad24. involved inReplication checkpoint control
120451	+	+	+	+	+	+	1.872 up	3.275 up	unknown protein
120453	+	+	+						SSCRP
120458	+	+	+	+	+	+			unknown protein
120473	+	+	+	+	+	+	1.170 up	2.137 up	Dihydrolipoamide transacylase (alpha-keto acid dehydrogenase E2 subunit)
120475	+	+	+	+	+	+	1.685 down	2.386 down	C2H2 transcriptional regulator
120479	+	+	+						SSCRP
120482	+	+	+	+	+	+			unknown protein
120484	+	+	+	+	+	+			Unknown coiled-coil protein
120485	+	+	+	+	+	+			unknown protein
120486	+	+	+	+	+	+	1.624 up	4.346 up	auxiliary protein of DNA polymerase delta
120489							2.331 down	2.972 down	unknown protein

120498										Phosphotyrosyl phosphatase activator. PTPA
120503		+	+	+	+		+			unique protein
120504			+	+				2.557 down	9.472 up	unique protein
120510		+	+	+	+	+	+			unknown protein
120516		+	+	+	+	+	+			unknown protein
120524		+	+	+			+			unknown protein
120529		+	+	+	+	+	+			unknown protein
120534	+	+	+	+	+	+	+			Ca2+-binding actin-bundling protein (fimbrin/plastin). EF-Hand protein superfamily
120535	+	+	+	+	+	+	+			unknown protein
120539		+	+	+	+	+	+			Adenylate kinase
120540		+	+	+	+	+	+			Ribosomal protein S23
120545		+	+	+	+	+	+			protein phosphatase 2A regulatory B subunit
120556										Mitochondrial substrate carrier
120557		+	+	+	+	+	+			unknown protein
120558										unknown protein
120566		+	+	+	+		+			unknown protein
120568		+	+	+	+	+	+			enolase
120570		+	+	+	+	+	+			tRNA splicing endonuclease SEN15 of yeast.
120571		+	+	+	+	+	+	1.596 up	5.082 up	pre-rRNA-processing protein PNO1
120577		+	+	+	+	+	+			unique protein
120579		+	+	+	+	+	+			Unknown protein
120583		+	+	+	+	+	+			unknown protein
120597		+	+	+	+	+	+	2.887 down	8.540 down	C2H2 transcriptional regulator
120600		+	+	+	+	+	+			unknown protein
120605		+	+	+	+	+	+			protein kinase Rim15. response regulator receiver p
120607		+	+	+	+	+	+			eukaryotic translation initiation factor 3. subunit 7.
120609										unknown protein
120610		+	+	+	+	+	+			Unknown protein

120618		+	+	+	+	+	+			mediator of RNA polymerase II transcription subunit 14. putative
120621		+	+	+	+	+	+			ribosomal protein L37 of the large (60S) ribosomal subunit. Belongs to the same family as
120623		+	+	+	+	+	+	2.154 down	2.316 down	unknown protein
120627		+	+	+	+	+	+			Ca-ATPases
120635		+	+	+	+	+	+			transketolase-like protein
120646		+	+	+	+	+	+			Spermine/spermidine synthase
120648		+	+	+	+	+	+			PhyHPhytanoyl-CoA dioxygenase (PhyH)
120650	+	+	+	+	+	+	+			20S proteasome alpha subunit Scl1
120654		+	+	+	+	+	+			unknown protein
120661		+	+	+	+	+	+			aps1. subunit of the adaptor protein complex AP-1
120675		+	+	+	+	+	+			ArgE
120676		+	+	+	+	+	+			GH37 α.α-trehalase
120688		+	+	+	+	+	+	1.495 up	2.635 up	unique protein
120689										unknown protein
120696										alcohol dehydrogenase
120697								3.428 up	4.518 up	SSCRP
120698		+	+	+	+		+			C2H2 transcriptional regulator
120702								1.202 up	4.743 up	unknown protein
120705										unknown protein
120712										3-oxoacyl-(acyl carrier protein) synthase
120715								2.085 down	2.028 down	Zn2Cys6 transcriptional regulator
120722		+	+	+	+	+	+			Serine/threonine protein phosphatases
120730		+	+	+	+	+	+			unknown protein
120733		+	+	+	+	+	+	1.470 up	3.649 up	mitochondrial import inner membrane translocase subunit TIM13 / Zn-finger. Tim10/DDP
120737		+	+	+	+	+	+			histone acetyltransferase type B catalytic subunit
120747										unknown protein
120749		+	+	+		+		2.428 down	2.475 up	GH1 ß-glucosidase BGL2/CEL1a

120751	+	+	+	+	+	+	2.015 down	2.226 down	unknown protein
120752							1.396 down	3.362 down	K+ transporter Trk1
120753	+	+	+	+	+	+			DNA repair helicase RAD25
120765									enoyl-CoA hydratase/isomerase
120767	+	+	+	+	+	+			unknown protein
120774	+	+	+						unknown protein
120779	+	+	+	+	+	+			unknown protein
120781	+	+	+	+	+	+			40s ribosomal protein S6
120784	+	+	+	+	+	+	1.608 down	2.766 down	cell wall mannoprotein
120786	+	+	+	+	+	+			HMGL-like
120789	+	+	+	+	+	+			Tubulin alpha chain 2
120791	+	+	+	+	+	+	1.410 up	2.105 up	unknown protein
120794	+	+	+	+	+	+			unknown protein
120799	+	+	+	+	+	+			unknown protein
120801	+	+	+	+	+	+			RNA recognition domain protein
120803		+	+						unknown protein
120805	+	+	+	+	+	+			unknown protein
120806	+	+	+	+	+	+			protein kinase
120807	+	+	+	+	+	+			GT66. STT3 subunit of ER oligosaccharyltransferase
120813		+							CAP-Gly domain aligned
120819	+	+	+	+	+	+			unknown protein
120821	+	+	+	+	+	+			phytase
120823		+	+				1.409 up	2.117 up	cell wall mannoprotein
120826	+	+	+	+					unknown protein
120828	+	+	+	+	+	+	1.240 up	2.442 up	unknown protein
120830	+	+	+	+	+	+			Tubulin alpha chain 1
120833	+	+	+	+	+	+			40s ribosomal protein S15 based on homology to corresponding proteins in other fungi.

120836	+	+	+	+	+	+			unknown protein
120837	+	+	+		+				unknown protein. 1 TM domain
120851									HET domain protein. related to N. crassa pin-c3
120860									germinal center kinase. related to S. cerevisiae Kic1
120864	+	+	+	+	+	+			DIP2. encoding a nucleolar protein. specifically associated with the U3 snoRNA of the SSU
120868	+	+	+	+	+	+			Ribosomal protein S13 (S15 family) based on homology to the corresponding protein in Gi
120872							2.434 down	3.420 down	GCN5 N-acetyltransferase
120873							1.069 down	11.000 up	GH71 α-1 3-glucanase
120877									Zn-dependent ß-lactamase
120879									unique protein
120884									Formyl_trans_NFormyl transferase
120889							1.271 up	2.110 down	cytochrome P450. putative
120892	+	+	+	+	+	+			unknown protein
120893	+	+	+	+	+	+			ATPase. AAA family protein. putative
120897	+	+	+	+	+	+			BRCT domain protein Rad4/Cut5 of Schizosaccharomyces pombe. a protein involved in DN
120899	+	+	+	+	+	+			unknown protein
120908	+	+	+	+	+	+			myb transcriptional regulator
120911	+	+	+	+	+	+	3.158 down	8.588 down	short chain dehydrogenase/reductase
120912		+							unknown protein
120915	+	+	+	+	+	+			unknown protein
120917	+	+	+	+	+	+			unknown protein
120918	+	+	+	+	+	+			unknown protein
120922	+	+	+	+	+	+			thioredoxin
120923									GT32 α-glycosyltransferase
120926							4.050 down	2.468 up	unique protein
120927							1.963 up	6.440 up	ankyrin containing protein

120928									unknown protein
120929	+	+	+	+	+	+			unknown protein
120931	+	+	+	+	+	+	7.346 up	12.502 up	unknown protein
120937	+	+	+	+	+	+			unknown protein
120940	+	+	+	+	+	+			Vacuolar protein sorting-associated protein Vps2
120943	+	+	+	+	+	+			NAD-dependent glutamate dehydrogenase
120952	+	+	+	+	+	+			Glutamine Phosphoribosylpyrophosphate amidotransferase PurF
120953									unknown secreted protein
120961			+				40.524 down	21.244 up	GH61 polysaccharide monooxygenase CEL61b
120968							2.928 down	13.631 up	Copper chaperone for superoxide dismutase
120969	+	+	+	+	+	+	1.548 up	2.148 up	NADPH oxidase regulator NoxR
120975									unknown protein
120978	+	+	+	+	+	+	1.121 up	2.011 up	large ribosomal subunit (protein L34e).
120980									unknown protein
120981	+	+	+	+	+	+	1.324 down	3.117 down	magnesium and cobalt transporter CorA
120983	+	+	+	+	+	+			uracil phosphoribosyl transferase
120985	+	+	+						unknown protein
120988									Zn2Cys6 transcriptional regulator
120993	+						1.306 down	3.637 up	unknown protein
120994	+	+	+	+	+	+			T-complex protein 1
120998	+	+	+	+	+	+			Carboxypeptidase Y homolog
121000									Tryptophanyl-tRNA synthetase. class I of Neurospora crassa
121003	+	+	+	+	+	+			Ribosomal protein S18. S13 family
121004	+	+	+	+	+	+			unknown protein
121009	+	+	+	+	+	+			20S proteasome beta-type subunit Pre2
121011	+	+	+	+	+	+			Unknown protein with Zn-finger. FYVE type domain.
121014		+		+					unknown protein

121019	+	+	+	+	+	+			Succinate dehydrogenase
121022	+	+	+	+	+	+			unknown protein
121023	+	+	+	+	+	+			eIF2 gamma subunit.
121028	+	+	+	+	+	+			14-3-3 protein
121031	+	+	+	+	+	+			valyl-tRNA synthetase. mitochondrial precursor.
121033	+	+	+	+		+			unique protein
121041									unknown protein
121042							2.457 up	7.562 up	unknown protein
121048	+	+	+	+	+	+			ubiquitin conjugating enzyme
121051	+	+	+	+	+	+			histidyl-tRNA synthetase. class IIa. from other fungi.
121056	+	+	+	+	+	+			unknown protein
121058							1.119 up	2.244 up	unknown protein
121061	+	+	+	+	+	+			checkpoint kinase 2-like protein
121065									unknown protein
121068	+	+	+	+	+	+			Phosphomannomutase (SEC53)
121071	+	+	+	+	+	+			unknown protein
121073	+	+	+	+	+	+			unknown protein
121074	+	+	+		+				homeobox transcriptional regulator
121080	+	+	+	+	+	+			Heteromeric CCAAT factors
121082	+	+	+	+	+	+			unique protein
121087	+	+	+	+	+	+			unknown protein
121088	+	+	+	+	+	+			unknown protein. secreted 1 TM
121089	+	+	+	+	+	+			unknown protein. only present in ascomycota and cow
121091	+	+	+	+	+	+			unknown protein
121095	+	+	+	+	+	+	1.186 down	2.528 down	cell cycle control protein (Cwf19). putative
121098	+	+	+	+	+	+			Multicopper oxidases
121107	+		+	+		+	1.213 up	2.152 down	Zn2Cys6 transcriptional regulator
121111									unknown protein

121121							1.399 up	2.196 up	Zn2Cys6 transcriptional regulator
121125	+	+	+	+	+	+			unknown protein
121126									unknown protein
121127							17.003 down	4.756 up	GH3 β-xylosidase BXL1
121130							1.021 up	2.166 down	Zn2Cys6 transcriptional regulator
121133	+	+	+	+		+			Aspartic peptidaseA1
121135							2.155 up	2.695 up	SSCRP
121136									unique protein
121138									Zn2Cys6 transcriptional regulator
121139									Amino acid transporters
121143	+	+	+	+	+	+			Phenylalanyl-tRNA synthetase
121146									unknown protein
121148	+	+	+	+	+	+			C2H2 transcriptional regulator
121154	+	+	+	+	+	+			Amidase
121156		+							Copper amine oxidase
121158	+	+	+	+	+	+			Serine/Threonine protein kinase
121163	+			+		+			unknown protein
121164	+	+	+						Zn2Cys6 transcriptional regulator
121166	+	+	+	+	+	+			SAM-dependent methyltransferases
121169	+	+	+	+	+	+			Vacuolar protein sorting-associated protein Vps46
121171	+	+	+	+	+	+	2.084 up	2.196 up	replication fork protection component Swi3
121172	+	+	+	+	+	+			unknown protein. 2 TM. related to N. crassa NADH-ubiquinone oxidoreductase
121175	+	+	+	+	+	+			unknown protein
121177									SSCRP
121178	+	+	+	+	+	+			unknown protein
121185									unknown protein
121187	+	+	+	+	+	+	2.968 down	2.772 down	unknown protein

121189									unknown protein
121191	+	+	+	+	+	+			Acetyltransf_Acetyltransferase (GNAT) family
121196	+	+	+	+	+	+			SWI-SNF chromatin-remodeling complex protein
121203	+	+	+	+	+	+	1.527 down	2.032 down	unknown protein
121206	+	+	+	+	+	+			unknown protein
121214									unknown protein
121219	+	+	+	+	+	+			ribosomal protein L11
121223	+	+	+	+	+	+			Secretion related small GTPase Sec4
121226	+		+				2.555 up	2.130 up	unknown protein
121227	+	+	+	+	+	+			GT2 dolichyl-phosphate ß-glucosyltransferase
121230	+	+	+	+	+	+	1.100 up	2.390 up	unique protein
121232	+		+						unknown protein
121238	+	+	+	+	+	+			ubiquitin carboxyl-terminal hydrolase
121239	+	+							unknown protein
121240	+								phosphoglycerate mutase
121247	+	+	+	+	+	+			unknown protein
121248	+	+	+	+	+	+			ATP12 chaperone . putative
121251			+						cell wall protein (Metarhizium adhesion Mad1 ?)
121252	+	+	+	+	+	+			Zn2Cys6 transcriptional regulator
121255	+	+	+	+	+	+			2-methylcitrate dehydratase-like protein
121262	+	+	+	+	+	+			unknown protein
121275	+	+	+	+	+	+			cytochrome c oxidase subunit VIa
121276	+	+	+	+	+	+			sla2. adaptor protein that links actin to clathrin
121278	+	+	+	+	+	+			unknown protein
121284	+	+	+	+	+	+			unknown protein
121285	+	+	+	+	+	+			unique protein. 1 TM
121289	+	+	+	+	+	+			unknown protein
121292	+	+	+	+	+	+			Emp24/gp25L/p24 family protein Erp3

121294	+	+					1.670 down	2.337 down	glucan endo-1.3(4)-β-D-glucosidase
121295	+	+	+	+	+	+	1.210 down	2.214 down	Dolichol kinase Sec59
121297	+	+	+	+	+	+			unknown protein
121298	+	+	+	+	+	+			formate dehydrogenase
121304	+	+	+	+	+	+			unknown protein
121306	+	+	+		+				Aspartyl protease
121308	+	+	+				1.666 up	2.084 up	PutA delta-1-pyrroline-5-carboxylate dehydrogenase
121310	+	+	+	+	+	+			Zn2Cys6 transcriptional regulator
121312	+	+	+	+	+	+			SSCRP
121315							1.335 up	3.384 up	unknown protein
121319	+	+	+	+	+	+			unknown protein
121325									unknown protein
121336									unique protein
121337							1.552 down	2.022 up	unknown protein
121338		+				+			unknown protein
121343	+	+	+	+	+	+			20S proteasome alpha-type subunit Pre5
121345									SerC Phosphoserine aminotransferase
121349	+	+	+	+	+	+			GTPase activating protein for Rab family members of Ras smallGTPases. related to S. cerev
121350									unknown protein
121351	+	+	+	+	+	+			GH31 Glucosidase II alpha subunit GLS2
121355									GH18 chitinase CHI18-rel2
121359	+	+	+	+	+	+			Ubiquitin associated protein probably involved in cell signalling via protein kinases and the
121362	+	+	+	+	+	+			unknown protein
121363	+	+	+	+	+	+			RNA export mediator Gle1. putative
121367	+	+	+	+	+	+			vacuolar ATP synthase catalytic subunit A
121372	+	+	+	+	+	+	1.084 up	2.660 up	60S ribosomal protein L38 by homology with corresponding proteins in other fungi and ye
121374	+	+	+	+	+	+			mevalonate kinase; ergosterol biosynthetic pathway

121381	+	+	+	+	+	+			Rad16
121396									unique protein
121397	+	+	+	+	+	+	1.036 down	2.050 up	Sec61alpha subunit
121399	+	+	+	+	+	+			unknown protein
121405							4.002 down	10.879 down	4-aminobutyrate aminotransferase
121408	+	+	+	+	+	+			ribosomal protein L27 domain. Shows amino acid sequence similarity to S. cerevisiae mito
121411	+	+	+	+	+	+			unknown protein
121412	+	+	+	+	+	+	4.497 up	2.811 up	Zn2Cys6 transcriptional regulator
121415							11.737 up	29.400 up	Zn2Cys6 transcriptional regulator
121416									unknown protein
121417							1.340 down	2.317 up	unknown protein with fasciclin domain
121418									lipase G-D-S-L
121420	+	+	+	+	+	+			glutamyl-tRNA synthetase. class Ic.
121427									unknown protein
121431	+	+	+	+	+	+			unknown protein
121436	+	+	+	+	+	+			Mitochondrial inner membrane insertase
121439	+	+	+				3.040 down	2.622 down	unknown protein
121441	+	+			+		1.208 up	3.323 down	carbounknown proteinlic acid transporter
121445									unknown protein
121449	+	+	+	+	+	+			IGPDImidazoleglycerol-phosphate dehydratase
121453	+	+	+	+	+	+			translation initiation factor eIF2B subunit (Gcd2p).
121464	+	+	+	+	+	+	1.181 up	3.226 up	GcvHGlycine cleavage system H protein (lipoate-binding)
121468	+	+	+	+	+	+			unknown protein
121469	+	+	+	+	+	+			ribosomal protein L4/L1e
121471	+	+	+	+	+	+			SSCRP

121474							1.420 down	4.098 down	Zn2Cys6 transcriptional regulator
121475	+								unique protein
121478									short chain dehydrognease/reductase
121482									MFS permease
121486							12.952 down	7.206 down	unknown protein
121491									GT4 α.α'-trehalose phosphorylase/synthase
121495							1.677 down	9.463 up	Vacuolar proteinase B (yscB). a serine protease of the subtilisin family
121498							12.234 down	2.704 down	Phosphatidylserine decarboxylase
121499									unknown protein
121500									metallophosphoesterase
121504	+	+	+	+	+	+			GPI transamidase component GAA1
121506	+	+	+	+	+	+			mitochondrial substrate carrier. comprises DEAD/DEAH box helicase domain
121516	+	+	+	+	+	+	1.279 up	2.128 up	histone H2B
121522	+	+	+	+	+	+			histone H2A
121529	+	+	+	+	+	+			Mitochondrial carrier protein
121533	+	+	+	+	+	+			unknown protein
121534									pyruvate decarboxylase
121539	+	+	+	+	+	+			MAPK1. mitogen activated protein kinase 1 involved in biocontrol activity in H. virens
121544									acetyltransferase
121546	+	+	+						acyltransferases that catalyze diacylglycerol esterification; phospholipid:diacylglycerol acy
121550	+	+	+	+	+	+			PssA Phosphatidylserine synthase
121553	+	+	+						unknown protein
121556									unknown protein
121558									unknown protein
121559	+	+	+	+	+	+			unknown protein
121569	+	+	+	+	+	+			Unknown protein
121573	+	+	+	+	+	+			DNA primase. small subunit

121579	+	+	+	+	+	+	1.519 up	2.047 up	unknown protein
121582	+	+	+	+	+	+			MRP4 encoding mitochondrial ribosomal protein of the small subunit.
121584	+	+	+	+	+	+			C2H2 transcriptional regulator
121588	+	+	+	+	+	+			beta-adaptin. apl2. large subunit of the adaptor protein (AP-1) complex of clathrin-coated
121593	+	+	+	+	+	+			unknown protein
121594							5.975 down	2.831 down	unique protein
121597	+	+	+	+	+	+			unique protein
121602			+				1.168 down	2.527 down	Zn2Cys6 transcriptional regulator
121605			+						unknown protein
121606	+	+	+	+	+	+			unknown protein
121608							2.331 down	7.628 down	MFS permease
121610	+	+	+	+	+	+			TOR kinase
121620			+						NMT1 thiamine biosynthesis protein
121629	+	+	+	+	+	+			acyl-CoA dehydrogenase
121630	+	+	+	+	+	+			member of WD40 repeat superfamily
121638	+	+	+				2.859 down	2.162 down	unique protein
121639	+	+	+	+	+	+			unknown protein
121646	+	+	+	+	+	+			vacuolar import and degradation protein 27
121647			+						unknown protein
121652	+	+	+	+	+	+			Vacuolar assembly protein Vps41
121653	+					+			unknown protein
121654	+	+	+	+	+	+			UTP6. a Component of the SSU processome
121656	+	+	+	+	+	+			unknown protein
121660	+	+	+						unknown protein
121661	+	+	+	+	+	+			aldose-1-epimerase
121664							1.022 down	2.411 up	Glutamate decarboxylase and related proteins

121670	+	+	+	+	+	+			Frequency clock protein FRQ-1
121681									unknown protein
121682			+						Zn2Cys6 transcriptional regulator
121684	+	+	+	+	+	+			calcium/calmodulin dependent protein kinase 2
121686	+	+	+	+	+	+			Serine hydroxymethyltransferase
121689									unknown protein
121693							2.324 down	2.982 down	Glutathione-dependent formaldehyde-activating. GFA
121702									unknown protein
121706	+	+	+	+	+	+			DAHP synthase ARO4
121717	+	+	+	+	+	+			unknown protein
121725	+	+	+	+	+	+			splicing factor SPF30.
121732									unknown protein
121733	+	+	+	+	+	+			delta(14)-sterol reductase
121735			+				3.392 up	5.256 up	GH3 β-glucosidase CEL3b
121739									SSCRP
121741	+	+	+	+	+	+	1.323 up	2.025 up	Ribosomal protein S7
121743	+	+	+	+	+	+	1.088 up	2.331 down	Mitochondrial succinate/fumarate antiporter
121746							1.952 up	3.896 up	GH55 exo-1 3-β-glucanase GLUC78
121750	+	+	+	+	+	+			unknown protein
121754	+	+	+	+	+	+			Translocation protein Sec63
121757	+	+	+	+	+	+			C2H2 transcriptional regulator
121764	+	+	+	+	+	+			unknown protein
121766	+	+	+	+	+	+			Telomere and ribosome associated protein
121773	+	+	+	+	+	+	1.896 up	2.074 down	unknown protein
121774	+	+	+	+	+	+			Guanine nucleotide exchange factor Sec12
121777	+	+	+	+	+	+			unknown protein
121785	+						19.861 down	2.194 down	ATP-dependent RNA helicase

121786									unknown protein
121789	+	+	+	+	+	+			triose-phosphate isomerase-like protein
121793	+	+	+	+	+	+			unknown protein
121794	+	+	+						Zn2Cys6 transcriptional regulator
121800	+	+	+				1.748 down	2.510 down	stomatin-like protein
121801	+	+	+	+	+	+			NOP58; component of the SSU processome.
121804	+	+	+	+	+	+	1.225 up	2.470 up	Ribosomal protein S15.
121814	+	+	+	+	+	+			alanine-glyoxylate aminotransferase
121818			+						unknown secreted protein
121820	+	+	+	+	+	+			Methionine_syntMethionine synthasevitamin-B independent
121824	+	+	+	+	+	+	1.118 down	2.540 up	ATP citrate lyase. alpha subunit
121826	+	+	+	+	+	+			ATP citrate lyase. beta subunit
121834	+	+	+	+	+	+			adenine nucleotide translocator
121837	+	+	+	+	+	+			Ribosomal protein S5. S7 family
121839	+		+						HSP70
121843	+		+						C6HC zinc finger protein
121850	+	+	+	+	+	+			hexose transporter
121856	+	+	+	+		+			4-hydroxyphenylpyruvate dioxygenase
121864	+	+	+	+	+	+	1.059 down	3.088 down	unknown protein
121868	+	+	+	+	+	+			Phosphoglycerate/bisphosphoglycerate mutase
121870	+	+	+	+	+	+	3.727 down	2.435 down	unknown protein
121875									Zn2Cys6 transcriptional regulator
121877	+	+	+			+	4.705 down	3.710 down	epoxide hydrolase
121878	+	+	+	+	+	+			40s ribosomal protein S19e by homology to the corresponding proteins of G. zeae and E. r
121883							6.827 up	4.673 up	unique secreted protein
121889	+	+	+	+	+	+			unknown protein

121890	+	+	+	+	+	+			mitochondrial processing peptidase. α-subunit
121894	+	+	+	+		+			Zn2Cys6 transcriptional regulator
121901	+	+	+	+	+	+			eukaryotic translation initiation factor 3 subunit 8. N-terminal.
121903	+	+	+	+	+	+			iron-sulfur cluster assembly accessory protein. putative
121904	+	+	+	+	+	+			unknown protein
121906	+	+	+	+	+	+	1.414 up	2.027 up	40s ribosomal protein S14 (S11 family).
121915	+	+	+	+	+	+			Translationally-controlled tumor protein homolog
121920	+	+	+	+	+	+			GT57 dolichyl-phosphate-glucose a-glucosyltransferase Alg8
121940	+	+	+	+	+	+			Tup1
121942	+	+	+	+	+	+			Aa_transTransmembrane amino acid transporter protein
121944	+	+	+	+	+	+	1.128 down	2.144 down	serine/threonine protein kinase
121948	+	+	+	+	+	+	1.810 up	3.070 up	signal peptidase spc12
121950	+	+	+	+	+	+			unknown protein
121955	+	+	+	+	+	+			Emg1p. a protein required for the maturation of the 18S rRNA and for 40S ribosome prod
121960	+	+	+	+	+	+			unknown protein
121962	+	+	+	+	+	+			Blue light regulator 1 BRL1
121966	+	+	+	+	+	+			Vesicle coat complex COPI. alpha subunit
121968	+	+	+	+	+	+	1.714 down	2.219 down	serine peptidase S28
121975	+	+	+	+	+	+			Clathrin associated epsin 2A
121977	+	+	+	+	+	+			CytoplasmicCyclophilin
121981	+	+	+	+	+	+			26S proteasome regulatory complex subunit Rpn11
121986			+						MFS permease
121989	+	+	+	+	+	+	1.126 down	2.243 down	oxalate decarboxylase
121990									PTH11 GPCR
121993	+	+	+	+	+	+			unknown protein
122001	+	+	+	+	+	+			ribosomal protein L14b/L23e.
122007			+				2.737 up	4.789 up	unique protein

122010									LdhA Lactate dehydrogenase and related dehydrogenases
122013	+	+	+						MFS permease
122015	+	+	+	+	+	+			Serine/threonine protein kinase required for receptor-mediated endocytosis
122023	+	+	+	+	+	+			unknown protein
122031	+	+	+	+		+			SAM-dependent methyltransferase
122036	+	+	+	+	+	+			Unknown protein
122040	+	+	+	+	+	+	1.178 up	2.036 up	ribosomal protein L31e.
122043	+	+	+						unknown protein
122047		+							cell wall protein. Ser/Thr-rich.
122048	+	+	+	+	+	+	1.382 up	3.580 up	Sec61 beta subunit
122050	+	+	+	+	+	+			unknown protein
122064	+	+	+	+	+	+			myosin heavy chain
122065	+	+	+	+	+	+			unknown protein
122067	+	+	+	+	+	+			unknown protein
122069									SSCRP
122070	+	+	+	+	+	+			pre-mRNA-processing factor 17
122071	+	+	+	+	+	+			ER-derived vesicles protein Erv14
122074			+						flavoprotein monooxygenases
122075	+	+	+	+	+	+			unknown protein
122076	+	+	+						aspartyl protease
122079		+	+			+			short chain dehydrogenase/reductase
122081			+				3.019 down	33.524 up	GH7 Endo-β-1.4-glucanase EGL1/CEL7b
122083									peptidase family M28
122084									unknown protein
122087	+	+	+	+		+			unknown protein
122089	+		+	+		+	7.444 down	3.053 down	unknown protein
122091	+	+	+	+	+	+	1.767 down	2.592 down	phospholipase of papatin-family

122095	+								unknown protein. secreted. only present in ascomycota
122096							1.613 up	7.382 up	unknown protein
122102	+	+	+	+	+	+	1.067 down	2.049 down	unknown protein
122103									unknown protein
122104		+					1.447 down	2.368 down	Xanthine/uracil permease family
122108	+	+	+	+	+	+			unknown protein. 1TM. only in Gibberella and Chaetomium
122110									4-coumarate:coenzyme A ligase
122113	+	+	+	+	+	+	1.257 up	2.126 up	unknown protein
122124									AAA ATPase
122127							1.574 up	2.024 up	SSCRP
122131	+	+	+	+	+	+			PCMTProtein-L-isoaspartate(D-aspartate) O-methyltransferase
122134				+					unknown protein
122139	+	+	+	+	+	+			unknown protein
122140				+			1.085 down	2.592 down	unique protein
122141	+	+	+	+	+	+			Ribosomal protein L28 (L29/L15. rp44. YL24 family). Homologue of S. cerevisiae RPL28.
122144	+	+	+	+	+	+			unknown protein
122146									unknown protein
122147									unique protein
122153									MFS permease
122160		+			+		1.845 up	4.787 down	Heat shock proteinwith DnaJ domain
122166	+	+	+	+	+	+			ERG5 C-22 sterol desaturase. a cytochrome P450Enzyme that catalyzes the formation of t
122168	+	+	+	+	+	+			unknown protein
122169	+	+	+	+	+	+	1.214 down	2.011 down	Histidine kinase. part of a two component signal transduction system
122172	+	+	+	+	+	+			GT 2. chitin synthase
122175	+	+	+	+	+	+			ribosome biogenesis protein Urb1 . putative
122176			+						unknown protein

122178	+	+	+	+	+	+			unknown protein
122180	+	+	+	+	+	+			unknown protein
122187									unknown protein
122193	+	+	+	+	+	+			Serine/threonine protein kinase. vps15. involved in vacuolar protein sorting
122197	+	+	+						unknown protein
122198	+	+	+	+	+	+			unique protein
122199	+	+	+	+	+	+			cell division control protein 3
122208	+	+	+	+	+	+			Zn2Cys6 transcriptional regulator
122210	+	+	+	+	+	+			ribosomal protein S4.
122212	+	+	+	+	+	+	1.276 down	2.188 down	vacuolar protein-sorting protein BRO1
122220									HET protein
122226	+	+	+	+	+	+			Carbamoyl-phosphate synthase. small chain
122230	+	+	+	+	+	+			unknown protein
122233	+	+	+	+	+	+			Iso_dhIsocitrate/isopropylmalate dehydrogenase
122240	+	+	+	+	+	+	1.159 down	2.210 down	Carnitine o-acyltransferase
122242		+	+						unknown protein. 1 TM
122255	+	+	+	+	+	+			unknown protein
122262									unknown protein
122271									Zn2Cys6 transcription regulator. C. albicans Fcr1
122275									Phenylalanyl-tRNA synthetase
122278	+	+	+	+	+	+	1.099 down	2.065 up	U3 small nucleolar RNA associated protein (SOF1)
122283	+	+	+	+	+	+			SWI-SNF chromatin-remodeling complex protein
122284	+	+	+	+	+	+	1.420 down	2.615 down	vel1. velvet protein
122287	+	+	+	+	+	+			glutamate synthase (NADPH). PUBMED 15846337
122293							1.035 down	2.797 down	unknown protein
122296	+	+	+	+	+	+			citrate (Si)-synthase

122299	+	+	+	+	+	+			unknown protein
122301	+	+	+	+	+	+			Cystathionine beta-lyases/cystathionine gamma-synthases
122304	+	+	+	+	+	+			unknown protein
122314									unknown protein
122324									SSCRP
122349	+	+	+	+	+	+			unknown protein
122350	+	+	+	+	+	+	1.366 down	3.530 down	Glutamate decarboxylase
122363	+		+				2.768 down	2.497 up	Hsp26/Hsp42
122366	+	+	+	+	+	+	1.161 down	2.117 down	unknown protein
122371	+	+	+	+	+	+			bHLH transcriptional regulator
122374							5.802 up	7.071 up	MRSP1/expansin-like
122375									MRSP1/expansin-like
122376			+						unknown protein
122378									Ribonuclease II
122381	+	+	+	+	+	+			unknown protein
122384	+	+	+	+	+	+			unknown protein
122385	+	+	+	+	+	+			glucosamine 6-phosphate synthetase
122387	+	+	+	+	+	+			transcriptional regulator HMG type
122392	+	+	+	+	+	+			Rfc2p of Saccharomyces cerevisiae.
122396	+	+	+	+	+	+			Nuclear transport regulator Npl4
122405	+	+	+	+	+	+			Ubiquitin-specific protease involved in catabolite repression. Appears to be be part of a co
122410	+	+	+	+	+	+			Exocyst component Sec10
122415	+	+	+	+	+	+			Protein disulfide isomerase 1
122416	+								NADH:flavin oxidoreductase/NADH oxidase
122419	+	+	+	+					Phosphatidylinositol 4.5-bisphosphate 5-phosphatase
122422		+	+				6.607 up	4.563 up	SSCRP
122428		+							unknown protein

122431	+	+	+	+	+	+			Pex19 (peroxin). a 40 kDa farnesylated protein associated with peroxisomes.
122447	+	+	+	+	+	+			Rad52
122448							1.273 up	7.482 up	C2H2 transcription factor
122455	+	+	+	+	+	+			unknown protein
122457	+	+	+	+	+	+			transcriptional activator Helix-turn-helix type 3
122468	+	+	+	+	+	+			GadB Glutamate decarboxylase and related PLP-dependent proteins
122476	+	+	+						ATG5 protein
122480	+	+	+	+	+	+			unknown protein
122487									DNAse
122495									GH76 α-1.6-mannanase
122497	+	+	+						unknown protein
122499							1.816 down	2.904 up	unknown protein
122500									Histone tail methylase containing SET domain
122501									unknown protein
122505							2.914 down	4.134 down	aryl-alcohol dehydrogenase
122506									unique protein
122511		+	+						glucan endo-1.3(4)-β-D-glucosidase
122523	+	+	+	+	+	+			Zn2Cys6 transcriptional regulator
122526	+	+	+	+	+	+	1.039 up	5.874 up	unknown protein
122527	+	+	+						unknown proteinwith Duf718 domain
122529	+	+	+	+	+	+	2.395 up	2.493 up	S1/P1 Nuclease
122531	+	+	+	+	+	+			AsdAspartate-semialdehyde dehydrogenase
122533	+	+	+	+	+	+			AldedhAldehyde dehydrogenase family
122541	+	+	+	+	+	+	1.175 down	2.915 down	C2H2 transcriptional regulator
122546	+	+	+	+	+	+			Vacuolar protein sorting-associated protein Vps27
122551	+	+	+	+	+	+			mpg1 encoding mannose-1-phosphate guanyltransferase involved in the O-glycosylation p
122552	+	+	+	+	+	+			dynein light chain

122556		+					7.167 up	13.985 up	Short-chain dehydrogenase/reductase
122565	+	+	+		+				CysKCysteine synthase
122567	+	+	+	+	+	+	1.795 down	2.102 down	unknown protein
122569									unknown protein
122571									unknown protein
122572	+	+	+	+	+	+	1.105 down	2.299 up	hsp70 family protein
122576		+							unknown protein
122579			+				5.546 up	4.905 up	unknown protein. 4TM
122582	+		+						unknown protein
122584	+	+	+	+	+	+			helix-turn-helix transcription factor
122587	+	+	+	+	+	+			unknown protein
122590	+	+	+	+	+	+			unknown protein. unique in fungi. 1TM
122592	+	+	+	+	+	+			PhosphoinositidePhosphatase. sac1. involved inProtein trafficking and secretion
122593	+	+	+	+	+	+			Secretion related GTPase Rab5/Ypt51
122614	+	+			+		2.211 down	3.394 down	unique protein
122628	+	+	+	+	+	+			unknown protein
122629							4.133 up	3.481 up	unique protein
122630	+	+	+		+	+			unknown protein
122641	+	+	+	+	+	+			nicotinate phosphoribosyltransferase
122644	+	+	+	+	+	+			CDP-alcohol phosphatidyltransferase
122646	+	+	+						Alpha/beta hydrolase
122653	+	+	+	+	+	+			squalene synthase
122656	+	+	+	+	+	+			unknown protein
122657	+	+	+	+	+	+			unknown protein
122659	+	+	+	+	+	+			UTP4. a component of the SSU processome
122662	+	+	+	+	+	+			unknown protein
122666	+	+	+	+		+			unknown protein

122679	+	+	+	+	+	+			unknown protein
122680	+	+	+	+	+	+			C2H2 transcriptional regulator
122684	+	+	+	+	+	+			transcriptional regulator. unknown
122689	+	+	+						unknown protein
122692	+	+	+	+	+	+			unknown protein
122696	+	+	+	+	+	+			unknown protein
122697	+	+	+	+	+	+			ribosomal protein L36
122701	+	+	+	+	+	+			actin-like protein
122703	+	+	+	+	+	+			mitochondrial-processing peptidase subunit beta
122708	+	+	+	+	+	+			Late Golgi protein sorting complex. subunit Vps53
122717	+	+	+	+	+	+			ubi4 ; polyubiquitin
122724									unknown protein. only in neurospora and Chaetomium
122730	+	+	+	+	+	+			Esterase/lipase/thioesterase superfamily
122734	+	+	+						unique protein
122735	+	+	+	+	+	+			protein kinase
122736	+	+	+	+	+	+			tagatose bisphosphate aldolase
122743	+	+	+	+	+	+			F-acting capping protein. alpha subunit
122745	+	+	+	+	+	+	1.845 up	3.234 up	2-oxoisovalerate dehydrogenase subunit beta. putative
122756	+	+	+	+	+	+			unknown protein
122767	+	+	+	+	+	+			N-terminal cooper fist DNA-binding domain-containing protein
122774	+	+	+	+	+	+			2-nitropropane dioxygenase
122778							7.686 down	2.730 down	short chain dehydrogenase/reductase
122780							4.450 up	6.086 up	GH28 exo-rhamnogalacturonase RGX1
122783	+								Zn2Cys6 transcriptional regulator
122792									unknown protein
122794	+	+	+	+	+	+			unique protein
122795			+				1.517 down	2.720 down	PTH11 GPCR
122811	+	+	+	+	+	+			glutamine synthetase

122813								2.128 up	3.983 up	unique protein
122817			+	+						unknown protein
122819		+	+	+	+	+	+	1.837 up	3.820 up	DNA replication complex GINS protein PSF3
122820								22.692 down	3.281 down	Kynurenine aminotransferase. glutamine transaminase K
122823		+								unknown protein
122824				+				10.764 down	35.206 up	PTH11 GPCR
122825								2.051 down	3.847 down	unknown protein
122828										nicotinamide riboside kinase 1. putative
122833		+	+	+	+	+	+			unknown protein
122838		+	+	+	+	+	+			unknown protein
122841	+	+	+	+	+	+	+			Exocyst component Sec3
122846		+	+	+	+	+	+			ATP-dependent RNA helicase.
122855		+	+	+	+	+	+			unknown protein
122856				+						unknown protein
122857		+	+	+	+	+	+			prohibitin-2
122860										unknown protein
122864		+	+	+	+	+	+			pre-mRNA splicing factor
122868		+	+	+	+	+	+			ThrA Homoserine dehydrogenase
122870		+	+	+	+	+	+	2.089 up	3.578 up	cell wall protein. CwpA
122872		+	+	+	+	+	+			Ubiquitin conjugating enzyme variant Mms2. a protein required for theUbc13-dependent
122874			+	+						unknown protein
122879		+	+	+	+	+	+			bHLH transcriptional regulator
122882		+	+	+	+	+	+			Hsp90 co-chaperone AHA1
122886		+	+	+	+	+	+			Tubulin beta chain 2
122889								36.228 down	2.689 down	unknown protein
122897										unknown protein
122898		+	+	+	+	+	+			unknown protein

122900	+	+	+	+	+	+			ribosomal protein RSM22.
122903	+	+	+	+	+	+			unknown protein
122909	+	+	+	+	+	+			translation elongation factor eEF-3 homologue YPL226w of Saccharomyces cerevisiae.
122914	+	+	+	+	+	+			unknown protein
122920	+	+	+	+	+	+			Molecular chaperone Bip
122929	+	+	+	+	+	+			unknown protein
122931									unknown protein
122941							1.806 down	3.349 up	unique secreted protein with CFEM domain
122943	+	+	+						SWI-SNF chromatin-remodeling complex protein
122948									multicopper oxidase
122952	+	+	+	+		+			unknown protein
122956	+	+	+	+	+	+			Ribosomal protein S27 based on homology to the corresponding protein of N. crassa.
122960	+	+	+	+	+	+			Amidase
122963	+	+	+	+	+	+			Cu_amine_oxidCopper amine oxidaseenzyme domain aligned
122971	+	+	+	+	+				unknown protein. only in Chaetomium. A. oryzae. fumigatus and Magnaporthe
122972									Cation transporting ATPase
122974							1.233 down	2.272 down	unknown protein
122975									unique protein with collagen triple helix repeat
122987	+	+	+	+	+	+			pyruvate dehydrogenase. E1 component alpha subunit
122988	+	+	+	+	+	+			MRP-type ABC transporter
122992							2.333 down	2.545 up	GT 31 ß-glycosyltransferase
122993		+							GT2 polysaccharide-forming ß-glycosyltransferase; distantly animal hyaluronan synthase
122994	+	+	+	+	+	+	1.279 down	2.043 down	unknown protein
122995							1.115 up	2.386 down	unknown protein
122998									unknown protein
123008									unknown protein

123009			+				2.980 up	2.582 up	glutamine synthetase
123013	+	+	+	+	+	+			Phosphatidylinositol 4-kinase
123014	+	+	+	+	+	+			unknown protein
123015	+	+	+	+	+	+			Secretion related GTPase Rab5/Ypt51
123019							1.298 down	2.272 down	unknown protein
123026	+	+	+	+	+	+			transaldolase
123029	+	+	+	+	+	+	1.339 down	6.480 down	Cu2+/Zn2+ superoxide dismutase SOD1
123030	+	+	+	+	+	+			unknown protein
123039		+	+				1.031 down	2.278 up	HFBs
123047	+	+	+	+	+	+			unknown protein
123056	+	+	+	+	+	+			hex1. encodes the major protein of the Woronin body forming a plug between fungal com
123066	+	+	+	+	+	+			unknown protein
123071	+	+	+	+	+	+			ATP synthase beta chain. mitochondrial precursor. associated to cellulase signal transduct
123079							7.591 up	9.716 up	short chain dehydrogenase/reductase
123084							1.995 down	2.052 down	chloroperoxidase
123086							1.367 up	190.175 up	SSCRP
123090	+	+	+	+	+	+			unknown protein
123095							2.880 up	2.588 down	unique protein
123111	+	+	+	+	+	+			Sorting nexin-1-like protein Vps5
123113	+	+	+	+	+	+			unknown protein
123114	+	+	+	+	+	+			Heat shock protein 90
123118	+	+	+	+	+	+			DnaJ-like protein JJ3. involved inDiphthamide biosynthesis.
123120							1.200 up	2.241 up	unknown protein. 8 TM
123124	+	+	+	+	+	+			unknown protein
123125	+	+	+	+	+	+			unknown protein

123126												unknown protein
123130				+	+	+	+	+	+			unique protein
123131				+	+	+	+		+			endonuclease/exonuclease/phosphatase. putative
123134				+	+	+						unknown protein
123144				+	+	+	+	+	+			unknown protein
123145				+	+	+	+	+	+			Ribosomal protein L17
123149										2.559 down	3.075 down	unknown protein
123152				+	+	+	+	+	+			Cation efflux protein
123155				+	+	+	+	+	+			Clathrin lightChain.Clc1. vesicleCoat protein
123158				+	+	+	+	+	+			t-SNARE Tlg2. endosome-derived vesicles-late Golgi
123163				+	+	+	+	+	+			γ-adaptin. apl4. large subunit of the adaptor protein (AP) complex of clathrin-coated vesic
123164				+	+	+	+	+	+			Signal recognition particle. subunit Srp72
123173				+	+	+	+	+	+			GT41 UDP-N-acetylglucosamine:peptide N-acetylglucosaminyltransferases
123174				+	+	+	+	+	+			Phosphoglycerate dehydrogenase and related dehydrogenases; RibA GTP cyclohydrolase I
123176				+	+	+	+	+	+			60S ribosomal protein L12 (L11 family).
123179				+	+	+			+			GTP cyclohydrolase
123180				+	+	+	+	+	+	1.257 up	2.173 up	Seryl-tRNASynthetase. class IIa.
123183				+	+	+	+	+	+			Ca2+ transporter. putative
123185				+	+	+	+	+	+			myosin heavy chain
123186				+	+	+						unknown protein
123188	+	+	+									unknown protein
123191				+	+	+	+	+	+	1.029 down	2.562 up	unknown protein
123196												unknown protein
123198				+	+	+	+	+	+			succinate dehydrogenase
123199										3.404 up	5.813 up	SSCRP
123202				+	+	+	+	+	+			60s ribosomal protein rla2
123204					+	+		+		3.159 up	2.647 up	lipase/esterase

123205							1.029 down	2.750 up	unknown protein
123207							3.296 up	2.828 up	unknown protein
123213	+	+	+	+	+	+			cell wall protein. Ser/Thr-rich.
123220	+	+	+	+	+	+			unknown protein
123223	+	+	+						threonine aldolase GLY1
123226	+	+	+	+	+	+	3.145 down	8.238 down	GH37 α.α-trehalase
123227							1.187 down	2.591 down	unknown protein
123228	+	+	+	+	+	+			clathrin heavy chain. chc1. vesicle coat protein
123232							15.789 down	11.782 up	GH12 endo-ß-1.4-glucanase
123234									Subtilisin like protease (SUB9)
123236							1.222 up	3.011 up	SSCRP
123238			+						epoxide hydrolase
123241	+		+						MFS permease
123244	+	+	+	+	+	+			Serine proteinase Sub8
123251									flavohemoglobin
123255	+	+	+	+	+	+			unknown protein
123256		+					2.168 up	2.251 up	unknown protein
123258	+	+	+	+	+	+			rho3
123260									unknown protein
123261									unknown protein
123262							1.494 down	4.007 down	unique protein. HTG. amidase domain. 1 TM
123265	+	+	+	+	+	+	1.801 up	2.330 up	unknown protein
123274							5.664 down	4.439 down	Aldehyde dehydrogenase
123278							1.875 up	3.054 up	unknown protein
123279	+	+	+	+	+	+			a-L-arabinofuranosidase I (Abf1) (EC 3.2.1.55)

123282										2.097 down	3.305 down	unknown protein
123283												GH54 α-L-arabinofuranosidase I ABF1
123288					+							xylulokinase
123290				+	+	+	+	+	+			sodium/hydrogen exchanger
123293				+	+	+	+	+	+			MDR-type ABC transporters
123296				+	+	+	+	+	+			unknown protein
123302				+	+	+	+	+	+			G-protein alpha subunit 1 GNA1
123313				+	+	+	+	+	+			Serine/threonine protein kinase
123321				+	+	+	+	+	+			unknown protein
123324				+	+	+	+	+	+			unknown protein
123325				+	+	+	+	+	+	2.108 up	2.548 up	zinc transporter
123327												SWI-SNF chromatin-remodeling complex protein
123343				+	+	+	+	+	+			unknown protein
123344				+	+	+	+	+	+			histidine phosphotransferase. tripartite hybrid signal transduction histidine kinase
123354						+						unknown protein
123363				+	+	+	+	+	+			unknown protein
123364				+	+	+	+	+	+			Protein involved in ER to Golgi transport Sly41
123368				+	+	+	+	+	+			GH13 1.4-α-glucan branching enzyme
123382				+	+	+	+	+	+			Coproporphyrinogen III oxidase
123396										1.023 down	3.179 up	unknown protein
123408				+	+	+	+	+	+			Actin-like protein 3
123422				+	+	+	+	+	+			GAP Gyp1
123426				+	+	+	+	+	+			unknown protein
123429				+	+	+	+	+	+			unique protein
123431												thymine diounknown proteingenase
123437					+	+						unknown protein
123441	+	+	+							2.384 up	2.308 up	unknown protein
123445												Zn2Cys6 transcriptional regulator

123455							1.035 down	2.549 down	unknown protein
123456	+	+	+						GH65 α.α-trehalase
123457	+	+	+	+	+	+			CTP synthase
123459									high affinity nickel permease
123460									unknown protein
123468									IMP dehydrogenase
123471	+	+	+	+	+	+			Subtilisin like protease. related to S. cerevisiae Kex2 protease
123473							3.810 down	14.364 down	MFS permease
123475	+	+	+	+	+	+	1.527 down	3.709 down	cell wall Thr-rich mannoprotein. Distantly related to S. cerevisiae Dan4p.
123476			+				1.094 down	5.959 up	unknown protein
123493	+	+	+	+	+	+	1.073 down	3.276 down	Ubiquitin-protein ligase (E3)
123499									HET-s/LopB domain protein
123502	+	+	+	+	+	+			CBM 21
123508									unknown protein
123509							1.506 up	3.955 down	Zn2Cys6 transcriptional regulator
123510							1.330 down	8.529 down	Zn2Cys6 transcriptional regulator
123538	+	+	+	+	+	+			GH72 β-1 3-glucanosyltransferase
123539									unknown protein. only in Gibberella and Arabidopsis
123540	+	+	+	+	+	+			unknown protein
123541	+		+	+	+	+			mitochondrial carrier protein
123543			+						unknown protein
123550									Indoleamine 2.3-dioxygenase-like protein
123551	+	+	+	+	+	+			unknown protein
123553	+	+	+	+			2.779 up	2.147 up	short chain dehydrogenase/reductase

123554	+	+	+	+	+	+			OTU-like cysteine protease. putative
123556	+	+	+	+	+	+			unknown protein
123559	+	+	+	+	+	+			ubiquitin-conjugating enzyme
123561	+	+	+	+	+	+			Kex2 protease to S. cerevisiae Kex2 protease
123562	+	+	+	+	+	+			Sorting nexin.Snx4; involved in maturation of the vacuolar aminopeptidase I in yeast
123571	+	+	+	+	+	+			unknown protein
123572	+	+	+			+	2.634 down	2.278 down	Phospholipase A2
123577	+	+	+	+	+	+			Endoplasmatic reticulum oxidising protein Ero1
123588	+	+	+	+	+	+			electron transport protein. probably involved in cytochrome C assembly
123598	+	+	+	+	+	+			nuclear pore protein
123604	+	+	+	+	+	+	1.224 up	2.042 up	Rps24 (family S16) by homology with similar proteins in other fungi.
123608							1.373 up	2.603 up	unique protein
123611	+	+	+	+	+	+			amino acid permease
123614	+	+	+	+	+	+			unknown protein
123616			+				1.984 up	2.365 up	short unique protein
123618	+	+	+	+	+	+			HD family hydrolase. putative
123619	+	+	+	+	+	+			SAGA complex subunit (Ada2)
123627	+	+	+	+	+	+			short chain dehydrogenase/reductase
123629	+	+	+	+	+	+			SSCRP
123631	+	+	+	+	+	+			Saccharopine dehydrogenase
123633									homoserine o-acetyltransferase
123636	+	+	+	+	+	+	1.722 down	2.658 down	unknown protein
123639							5.100 up	2.625 up	GH64 endo-1.3-β-glucanase
123648									unknown protein
123649	+						1.749 down	3.910 down	unique protein
123650	+	+	+	+		+			unique protein
123655	+	+	+	+	+	+			Protein involved in regulation of arginine-responsive genes

123658									unique protein
123659	+		+	+		+	20.748 down	5.487 down	cell wall protein. istantly related to S. cerevisiae Pir3p.
123663	+	+	+	+	+	+			unknown protein
123668									N-acetyltransferase activity
123672	+	+	+	+	+	+			Serine/Threonine protein kinase
123673									Ankyrin
123674									unique protein
123675									unknown protein
123680	+	+	+	+	+	+			unknown protein
123681	+	+	+	+	+	+			short chain dehydrogenase/reductase
123686	+	+	+	+	+	+			unknown protein
123687	+	+	+	+		+			unknown protein
123690	+	+	+	+	+	+			protein kinase. mago nashi protein
123695	+	+	+	+	+	+	1.705 up	2.807 up	unknown protein
123697	+	+	+	+	+	+	2.644 down	5.059 down	Unknown protein
123699	+	+	+	+	+	+			Zn2Cys6 transcriptional regulator
123702	+	+	+						MFS permease
123705	+	+	+	+	+	+			UDP-glucose-6-dehydrogenase
123710									unknown protein
123713			+						transcriptional regulatorMedA. involved in fruiting body development
123714	+	+	+	+	+	+			carbonic anhydrase
123717	+	+	+	+	+	+			Zn2Cys6 transcriptional regulator
123718			+						amino acid transporter. neutral 11 TM
123720	+	+	+	+	+	+			3-ketoacyl-CoA thiolase-like protein
123723							1.085 down	2.645 down	Arylacetamide deacetylase
123726									glucan endo-1.3(4)-β-D-glucosidase
123729	+	+	+	+	+	+			malate dehydrogenase

123732							3.583 down	4.606 down	unique protein
123733									HET protein
123735									heavy metal translocating P-type ATPase. putative
123738	+	+	+	+	+	+			Arginase
123740	+	+	+	+	+	+	1.430 down	2.305 down	unknown protein
123753	+	+	+	+	+	+			ubiquitin/small ribosomal subunit protein 31 fusion protein
123758	+	+	+	+	+	+			unknown protein
123767	+	+	+	+	+	+			Vacuolar protein sorting-associated protein Vps4
123771	+	+	+	+	+	+			GCD. Glutaryl-CoA dehydrogenase
123773	+	+	+	+	+	+			Ubiquitin-conjugating enzyme
123776	+	+	+	+	+	+			unknown protein
123777									unique protein
123779							1.577 up	2.671 down	unique protein
123780	+	+	+	+	+	+			unknown protein
123786									NRPS
123787									Zinc-containing alcohol dehydrogenase
123795	+	+	+	+	+	+			allantoinase
123797		+	+						unique protein
123801	+	+	+	+	+	+			aspartyl-tRNA synthetase. class IIb.
123805	+	+	+	+	+	+	1.360 down	2.531 down	DHBP_synthase.-dihydroxy--butanone -phosphate synthase
123806	+						3.020 down	3.750 down	GPCR. secretin like
123809	+	+	+	+	+	+			MFS permease
123818			+				23.487 down	2.491 up	GH11 endo-ß-1.4-xylanase XYN2
123820	+	+	+	+	+	+			BCAT_beta_family

123827	+	+	+	+	+	+	1.521 down	2.737 down	bifunctional catalase/peroxidase
123831	+	+	+	+	+	+			Serine/Threonine protein kinase
123832	+	+	+	+	+	+			Molecular chapeone DnaJ superfamily
123837	+	+	+	+	+	+			actin-like protein. centractin
123842	+	+	+	+	+	+			unknown protein
123850	+	+	+	+	+	+	1.270 up	2.171 up	60s acid ribosomal protein P1 based on homology to corresponding proteins in fungi and v
123860	+	+	+	+	+	+			CCR4-NOT transcription complex subunit 3
123865							5.058 up	2.021 up	Peptidase S8 and S53. subtilisin. kexin. sedolisin
123881	+	+	+	+	+	+			Zn2Cys6 transcriptional regulator
123882	+	+	+	+	+	+			unknown protein
123884									unknown protein
123888	+	+	+	+	+	+	2.767 down	5.528 down	unknown protein
123890	+	+	+	+	+	+			CDC3. Sporulation-specific
123900	+	+	+	+	+	+			ribosomal protein S9.
123902	+	+	+	+	+	+			elongation factor 1. gamma chain.
123903	+	+	+	+	+	+			cell cycle control protein cwf16
123911		+							unknown protein
123914									unique protein
123920	+	+	+	+	+	+			Rho-GTPase activating protein. distantly related to Rga8
123922	+	+	+	+	+	+			Peptidyl-prolyl cis-trans isomerase. cyclophilin type
123928	+	+	+	+	+	+			unknown protein
123934	+	+	+	+	+	+			MGM1 protein. mitochondrial precursor
123940							3.232 down	40.577 up	GH115 methylglurunoyl esterase CIP2
123946									dehydrogenase associated with cellulase signal transduction (PMID: 15288024)
123951	+	+	+	+	+	+			unknown ER membrane protein
123955							18.904 up	8.468 up	Epl1/Sm1
123962	+	+	+	+	+				unique protein

123963				+	+	+	+	+	+			unknown protein
123965				+	+	+	+	+	+			unknown protein
123967										1.272 up	2.252 up	HFB3
123968					+	+				3.090 up	2.561 up	unknown protein
123969				+	+	+	+	+	+			unknown protein
123974				+	+	+	+	+	+			Vesicle coat complex COPI. beta` subunit
123976												unique protein
123978												GMC methanol oxidase
123979												peptide transporter MTD1
123987												unknown protein
123989										6.841 down	2.485 up	GH7 Cellobiohydrolase CBH1/CEL7a
123992										3.714 down	3.757 up	swollenin
123999	+	+	+		+					2.266 up	2.361 up	NADH:flavin oxidoreductase/NADH oxidase
124000				+	+	+	+		+			unknown protein
124001												Protein phosphatase 2C/pyruvate dehydrogenase (lipoamide) phosphatase
124002												Sexual differentiation process protein ISP4
124007												unknown protein
124010				+	+	+	+	+	+			60S ribosomal protein L16 (L13 family).
124016				+	+	+						GH36 α-galactosidase AGL2
124022										8.500 down	2.036 up	Zn2Cys6 transcriptional regulator
124030										1.742 down	2.166 down	unknown protein with TIM barrel
124031				+	+	+	+	+	+			20S proteasome beta-type subunit Pre9
124040												unknown protein
124043										1.241 up	8.695 up	GH18. chitinase CHI18-14
124051						+						alpha/beta hydrolase
124052				+	+	+	+	+	+			histone 2A
124056				+		+	+		+			phosphatidic acid phosphatase

124058				+	+	+	+	+	+			unknown protein
124059						+				6.566 down	2.088 up	SSCRP
124061				+	+	+	+	+	+			unknown protein
124065										1.804 down	6.623 up	unknown protein. only in bacteria; contains large nuclear transport factor 2 domain
124079										1.365 up	2.734 up	Multicopper oxidases
124083												Epl1/Sm1
124084						+						unknown protein
124092												unknown protein
124094				+	+	+	+	+	+			unknown protein
124097				+	+	+	+	+	+			phenazine biosynthesis protein phzF
124101				+	+	+	+	+	+			Nuclear transport factor 2
124104				+	+	+	+	+	+	1.247 down	2.473 down	unknown protein
124110				+	+	+	+	+	+			Ribosomal protein L8
124113					+	+						PTH11 GPCR
124115				+	+	+	+	+	+	1.640 down	4.849 down	phosphoenolpyruvate carboxykinase AcuF
124116										1.254 up	2.082 down	unknown protein
124117				+	+	+	+	+	+			casein kinase II. beta (regulatory) subunit
124119	+	+	+	+	+	+	+	+	+	1.157 down	3.788 down	unique protein
124132				+	+	+	+	+	+			pH-response regulator protein palA/RIM20
124134												unknown protein
124136												short chain dehydrogenase/reductase
124141												unknown protein
124148				+	+	+	+	+	+			ubiquinol-cytochrome-C oxidoreductase complex III subunit VIII. 11kD protein of the Ucr[illegible]
124149				+	+	+	+	+	+			ribosomal protein L7AE domain.

124157	+	+	+	+	+	+	1.051 up	3.312 down	unknown protein
124158	+	+	+	+	+	+			N-acetyltransferase complex ARD1 subunit
124169									unknown protein
124170							1.352 down	3.206 down	arsenite methyltransferase
124172	+	+	+	+	+	+			protein kinase NPKA [E. nidulans]. probably involved in DNA damage signal transduction
124173	+	+	+	+	+	+			SSCRP
124174	+	+	+	+	+	+			Peptidyl-prolyl cis-trans isomerase. cyclophilin type
124175									GH64 endo-1.3-β-glucanase
124177	+	+	+	+	+	+	1.683 down	2.586 down	unknown protein
124179	+	+	+	+	+	+			cyclin-dependent protein kinase . putative
124181									Unknown protein with Tubulin_FtsZ domain.
124187	+	+	+	+	+	+	1.166 up	2.551 up	ER-associated protein degradation
124195	+	+	+	+	+	+			FeS cluster assembly scaffold IscU
124198									unknown protein
124202	+	+	+	+	+	+			unknown protein
124205	+	+	+	+		+			RNA polymerase III transcription initiation factor complex (TFIIIC) subunit part of the TauA
124206	+	+	+	+	+	+			ribosomal protein S10
124210	+	+	+	+	+	+	1.144 up	2.673 up	histone H3
124222	+	+	+	+	+	+	1.336 down	4.232 down	CaaX-protease. related to E. nidulans rce1. involved in signal transduction
124223	+	+	+	+	+	+			unknown protein
124228							1.455 down	3.406 down	GT2 chitin synthase
124234	+	+	+	+	+	+			adenylate cyclase associated protein (CAP)
124246	+	+	+	+	+	+	1.375 up	2.022 up	unknown protein
124249		+	+				2.271 up	3.665 up	unknown protein
124256		+	+				1.855 up	4.359 up	phospholipase-like protein
124259									SSCRP

124260							1.123 down	2.464 down	Zn2Cys6 transcriptional regulator
124277									SSCRP
124278	+	+	+	+	+	+	1.907 up	2.834 up	unknown protein with EXS domain
124282		+							unknown protein. SET and MYND domain
124283							3.244 up	7.355 up	unknown protein. SET and MYND domains
124285	+	+	+	+	+	+			small nuclear ribonucleoprotein Sm D1
124286		+					1.274 down	2.340 down	Heteromeric CCAAT factors
124288									unknown protein
124293	+	+	+	+	+	+			unknown protein
124295	+	+	+	+	+	+	3.114 down	3.010 up	SSCRP
124296							2.810 down	2.316 up	unique protein

* presence of one of the specified methylations is indicated by "+"; cells lacking + exhibit no methylation. The color code is the same as used in Supplementary Table S2.

Table S5 A few genes are associated with H3K4 methylation, which would be consistent with a positive role of LAE1 in H3K4 methylation*

**protein ID**	**H3K4me2**			**H3K4me3**			**ratio *[Δlae1]***	**ratio *[lae1OE]***	**Annotation**
	WT	*Δlae1*	*lae1OE*	WT	*Δlae1*	*lae1OE*			
47926	+	+	+	+			1,723 up	4,383 up	ABC1 family protein
72612			+				2,100 down	3,496 down	acetylornithine deacetylase
53372	+		+	+		+	2,753 down	2,231 down	acyltransferase 3
70859	+		+	+		+	4,320 down	5,344 down	amidase
81420	+	+	+	+			2,875 down	2,643 down	Argonaute siRNA chaperone (ARC) complex subunit
121785	+						19,861 down	2,194 down	ATP-dependent RNA helicase
65315	+		+				16,360 down	11,572 down	bZIP transcription factor
54703	+	+	+	+		+	2,647 down	2,513 down	C2H2 transcriptional regulator
102920	+	+	+	+		+	4,625 down	6,239 down	C2H2 transcriptional regulator
4876	+	+	+	+			2,534 down	5,033 down	catechol dioxygenase
123659	+		+	+		+	20,748 down	5,487 down	cell wall protein, istantly related to S. cerevisiae Pir3p.
75713	+	+	+	+		+	6,996 down	2,139 down	Cytochrome P450 CYP2 subfamily
120088	+	+	+	+		+	1,302 up	3,685 up	cytosolic asparaginyl-tRNA synthetase, required for protein synthesis, catalyzes the spe tRNA.
121877	+	+	+			+	4,705 down	3,710 down	epoxide hydrolase
79741	+	+	+	+		+	1,807 down	2,212 up	ER-bound Farnesyl-diphosphate farnesyltransferase
54144			+				1,097 down	3,141 up	ferric reductase
104211			+	+		+	15,509 down	24,344 up	FKBP-type peptidyl-prolyl cis-trans isomerase
2185			+				1,764 up	7,251 up	GCN5-related N-acetyltransferase
61703	+	+	+	+		+	1,316 up	2,241 up	germinal center kinase, related to S. cerevisiae Kic1
50215	+		+			+	6,380 down	2,374 up	GH16 endo-1,3-β-D-glucosidase/1,3-glucan binding protein
45717			+				4,496 down	4,485 down	GH47 α-1,2-mannosidase
82616	+		+				5,179 down	4,507 down	GH5 membrane bound endoglucanase CEL5b
82633			+				3,948 down	4,784 down	GH72 β-1 3-glucanosyltransferase
76620	+		+				2,041 down	2,566 down	Glycerol-3-phosphate dehydrogenase
123806	+						3,020 down	3,750 down	GPCR, secretin like

77557	+		+		+	7,316 down	5,660 down	GT glycosyltransferases not yet assigned to a family, 3 TMs
66888	+					11,913 down	5,286 down	GT α-1,3-mannosyltransferase
65817	+	+	+	+		5,911 down	3,410 down	GT α-1,3-mannosyltransferase CMT1
4561	+	+	+	+	+	2,718 down	2,569 down	GT α-1,6-mannosyltransferase
119963	+		+	+	+	1,202 up	2,031 up	HFBs
119805	+	+	+	+	+	1,232 up	2,096 up	HFBs
122363	+		+			2,768 down	2,497 up	Hsp26/Hsp42
105260	+	+	+	+	+	2,886 down	12,720 down	MFS permease
53475			+			2,083 down	2,851 down	MFS permease
68813			+			3,217 down	2,939 down	MFS permease
106248			+			1,696 up	3,534 up	monosaccharide transporter (galactose permease ?)
44476	+		+			4,980 down	2,986 down	MRP-type ABC transporter
73536			+			1,012 up	10,116 up	NADP-glutamate dehydrogenase
82105	+					2,755 down	4,288 down	PDR-type ABC transporters
64959	+	+		+	+	13,328 down	2,437 down	phosphatidyl synthase
3568	+	+	+	+	+	5,337 down	3,682 down	phospholipase A2
123572	+	+	+		+	2,634 down	2,278 down	Phospholipase A2
44278	+	+	+	+	+	2,647 down	4,813 down	Rab geranyl transferase escort protein
70355	+		+	+	+	5,994 down	18,605 down	SAM-dependent methylransferase
60758	+		+	+	+	12,719 down	5,079 down	SAM-dependent methyltransferase
4442	+					2,342 down	3,378 down	SAM-dependent methyltransferase
3055	+			+	+	22,757 down	2,485 down	short chain dehydrogenase/reductase
123616			+			1,984 up	2,365 up	short unique protein
71167	+		+			1,202 down	3,268 up	SSCRP
53366	+	+	+	+	+	1,353 up	2,151 up	translation initiation protein Sua5p.
106223	+	+	+	+	+	2,658 down	6,040 down	unique protein
119902	+	+	+	+	+	4,795 down	2,418 down	unique protein
43392	+		+		+	1,651 up	5,859 up	unique protein
104695	+	+	+	+	+	1,708 down	2,243 up	unique protein
105167			+			1,123 up	2,845 up	unique protein

107112			+			1,851 up	2,498 up	unique protein
4851	+		+	+	+	194,650 down	3,832 down	unknown protein
122089	+		+	+	+	7,444 down	3,053 down	unknown protein
65522	+		+			10,051 down	4,155 down	unknown protein
59940	+	+	+	+	+	3,732 down	3,082 down	unknown protein
60616	+	+	+	+	+	4,708 down	3,985 down	unknown protein
3488	+	+	+	+		2,876 down	2,578 down	unknown protein
107202			+			3,857 down	2,407 down	unknown protein
54622	+		+			2,553 down	4,686 up	unknown protein
43199	+		+			1,442 up	3,792 up	unknown protein
43302	+		+			1,007 down	5,446 up	unknown protein
71146	+		+			1,114 up	3,012 up	unknown protein
71154	+		+			1,158 up	2,392 up	unknown protein
120993	+					1,306 down	3,637 up	unknown protein
43083	+	+	+	+		1,236 down	3,216 up	unknown protein
105707			+			1,072 up	3,169 up	unknown protein
50996	+	+		+	+	4,528 down	7,482 down	unknown protein, C2 domain
49928			+			1,144 down	7,578 up	unknown protein, only in Sordariomycetes
102499			+			1,036 down	6,618 up	Zn2Cys6 transcriptional regulator

* genes are sorted alphabetically according to gene anniotation; other abbreviations and the color code are used as in Supplementary Table S3
