# Supplementary material for: Functional Analyses of Trichoderma reesei LAE1 Reveal Conserved and Contrasting Roles of This Regulator
Source: G3 (Bethesda). 2013 Feb 1;3(2):369–78. doi: 10.1534/g3.112.005140 (PMC3564997; doi:10.1534/g3.112.005140)
Supplement: Supporting Information [file supp_3.2.369_TableS1.pdf]

Table S1 Primers used for qPCR in this study

Gene	sequence (primer forward 5´→3´)	sequence (primer reverse 5´→3´)	primer efficiency (%)	Tm (°C)
Gene	primer efficiency (%)	Tm (°C)	100	57.2
vel1	AGCCTTATGTGCCTCACT	GCAGGAGCAGAGTAGTTG	100	57.2
lae1	ACTGGAGATTGACTGGATGC	TTCTGCGTCTGGTAGCCTC	100	62.9
Trire2:23171	GGTGGCTGTAGATGTGTT	TGAGAGTCGTCGTTGAAG	100	57.2
Trire2:112083	GGTGAAGAGATACGGTAT	TGAGTTGGAGTAGATGTC	100	52.6
Trire2:109101	GAATCCAGTGGCATTCTATC	CGACCCTCAATAATATCATACC	100	57.1
Trire2:69187	GCCTCTACCAATGAACTC	CCGAGACAGGACATAATC	97.2	56.1
Trire2:107071	CGATTGCCGTCTACGATAT	GCTCAGTTGTCCAGTCAT	100	56.1
Trire2:111494	ATCAACCAGACAGACAAC	AGATATAGACTTGCTCACATAG	95	52.6
Trire2:122824	CGTATGGCACCATCATTGG	CTGGGAAGACTCGTCGGAG	100	64
Trire2:39587	CTTGACAACCCGCCAAACAG	CGATGGAGTAAAAGACACCG	87.5	62
Trire2:82041	CTCATCGTCTTCCCCATTC	CATGCTTGTTGTTGTGCCCTG	100	62
Trire2:69904	CGGGAGACATTTCGCCTTTATC	CAGAAGCAGGTCGCAGAGTAG	86	64
Trire2:5647	CTACAACCCCGATAACCTC	CGTGGAAGATGCGGTAGTAC	98.5	60
Trire2:110744	CCTCTACGCCTCGTCAATC	CAATGGCGGAGAAGAAGAAGC	85	62
Trire2:110339	GATGCCGCTGATTGCCAC	CGAGATCGGTGATGATGTCG	91	65
Trire2:62462	CACGAACCCAACGCTCCAAC	CGAAGTATGCCCACTCCAG	95.5	63
Trire2:121990	CTCAAGGTAGTTGCGGTG	CGTCATCTGGTGGCTGTTG	90	60
Trire2:76763	CGGCAGAGGTTGAGCAATAC	CGGTAACACGGAGGCGTTTC	89	64
Trire2:105224	CACCGCTTGCCATCATCTTC	CAAGAGGAATCCCAAGACAC	100	61
sar1	TGGATCGTCAACTGGTTCTACGA	GCATGTGTAGCAACGTGGTCTTT	64	64
