# Supplementary material for: Functional Analyses of Trichoderma reesei LAE1 Reveal Conserved and Contrasting Roles of This Regulator
Source: G3 (Bethesda). 2013 Feb 1;3(2):369–78. doi: 10.1534/g3.112.005140 (PMC3564997; doi:10.1534/g3.112.005140)
Supplement: Supporting Information [file supp_3.2.369_TableS2.pdf]

Table S2 Genes differentially expressed in T. reesei strains modulated in lae1 function*

SEQ_ID	wild-type	S.D.	*Δlae1*	S.D.	*lae1OE*	S.D.	ratio *[Δlae1]*	ratio *[ae1OE]*	p-value	F- value	
1959	6.55	0.23	6.99	0.07	8.78	0.15	1.359 up	4.687 up	0.00	166.52	Haloacid dehalogenase-like hydrolase
1977	12.71	0.03	12.57	0.04	11.58	0.14	1.106 down	2.191 down	0.00	89.50	Peptidase_M28
1993	12.52	0.05	12.44	0.14	11.27	0.09	1.057 down	2.381 down	0.00	151.74	γ-glutamyl phosphate reductase GPR
2033	4.81	0.06	3.30	0.04	5.95	0.13	2.855 down	2.205 up	0.00	404.26	unknown protein
2036	6.24	0.02	6.11	0.09	7.75	0.16	1.090 down	2.856 up	0.00	142.94	cysteine peptidase
2071	9.68	0.07	10.58	0.09	10.86	0.09	1.875 up	2.266 up	0.00	123.87	unknown protein. 5 TM
2076	9.84	0.10	6.09	0.11	6.75	0.45	13.496 down	8.504 down	0.00	67.36	malate permease
2091	12.79	0.03	12.11	0.17	11.37	0.08	1.604 down	2.676 down	0.00	136.90	6-phosphofructokinase
2096	9.25	0.02	9.96	0.15	7.47	0.31	1.637 up	3.429 down	0.00	76.37	Zn2Cys6 transcriptional regulator
2125	12.16	0.01	12.35	0.05	11.13	0.10	1.146 up	2.029 down	0.00	188.64	Fructose-2.6-bisphosphatase
2148	13.15	0.02	12.06	0.21	11.74	0.24	2.133 down	2.651 down	0.00	30.59	Zn2Cys6 transcriptional regulator
2185	5.85	0.02	6.67	0.15	8.71	0.36	1.764 up	7.251 up	0.00	76.38	GCN5-related N-acetyltransferase
2223	8.84	0.12	9.08	0.13	10.23	0.22	1.178 up	2.618 up	0.00	45.43	succinate-CoA ligase. alpha subunit
2316	12.36	0.10	10.37	0.27	10.33	0.10	3.965 down	4.082 down	0.00	141.24	unknown protein with caleosin domain
2322	9.61	0.01	9.18	0.14	8.22	0.17	1.344 down	2.612 down	0.00	65.81	unknown protein
2358	9.66	0.06	9.32	0.03	8.12	0.28	1.269 down	2.915 down	0.00	40.58	unknown protein with HIT domain
2392	14.29	0.09	14.02	0.04	12.19	0.23	1.209 down	4.288 down	0.00	117.02	peroxisomal hydratase-dehydrogenase-epimerase
2399	11.59	0.01	11.51	0.10	9.90	0.08	1.056 down	3.210 down	0.00	457.97	unknown protein
2489	6.70	0.04	5.83	0.06	8.45	0.38	1.820 down	3.356 up	0.00	59.33	Ribonuclease T2
2492	10.31	0.17	9.44	0.02	8.18	0.24	1.832 down	4.378 down	0.00	78.04	Phosphatidylinositol-4-phosphate 5-kinase
2499	7.61	0.09	7.85	0.04	5.36	0.27	1.182 up	4.741 down	0.00	122.47	unknown protein
2517	4.72	0.08	4.23	0.05	5.82	0.41	1.399 down	2.149 up	0.01	18.41	Flavoprotein monooxygenase. putative
2526	13.57	0.02	13.29	0.14	12.56	0.14	1.215 down	2.010 down	0.00	50.83	Protein kinase C
2570	3.28	0.07	3.82	0.24	4.83	0.08	1.448 up	2.929 up	0.00	105.25	short chain dehydrogenase/reductase
2687	12.56	0.07	10.54	0.17	10.90	0.10	4.071 down	3.173 down	0.00	197.49	AAA ATPase
2703	11.87	0.14	11.97	0.04	10.62	0.24	1.074 up	2.372 down	0.00	41.84	unknown protein

2730	13.28	0.05	13.24	0.00	12.16	0.20	1.024 down	2.171 down	0.00	47.38	unknown protein
2745	12.92	0.02	12.76	0.13	11.80	0.12	1.115 down	2.175 down	0.00	91.93	fumarate hydratase-like protein
2835	11.99	0.01	11.83	0.01	10.80	0.21	1.112 down	2.275 down	0.00	44.84	unknown protein
3043	3.23	0.03	3.71	0.18	5.11	0.18	1.393 up	3.664 up	0.00	105.89	SSCRP
3049	13.72	0.39	12.80	0.01	10.85	0.08	1.898 down	7.341 down	0.00	181.03	methionine aminopeptidase
3055	12.48	0.15	7.97	0.14	11.17	0.12	22.757 down	2.485 down	0.00	651.11	short chain dehydrogenase/reductase
3094	5.60	0.12	5.26	0.11	7.17	0.67	1.265 down	2.986 up	0.02	11.46	GH30 glucan endo 1.6-β-glucanase
3121	3.35	0.46	3.82	0.25	4.97	0.12	1.379 up	3.066 up	0.00	31.51	cytochrome P450 oxidoreductase OrdA-like
3196	12.47	0.11	12.28	0.05	10.80	0.16	1.138 down	3.171 down	0.00	131.28	GH38 α-mannosidase
3262	3.61	0.03	4.01	0.01	5.31	0.21	1.315 up	3.244 up	0.00	86.89	succinate semialdehyde dehydrogenase (NADP)
3267	9.12	0.02	10.14	0.06	7.30	0.31	2.019 up	3.543 down	0.00	100.17	MRSP1/expansin-like
3350	11.77	0.18	12.11	0.24	10.76	0.31	1.265 up	2.002 down	0.01	18.73	phosphopantothenate-cysteine ligase
3364	11.25	0.06	10.43	0.11	9.12	0.17	1.768 down	4.386 down	0.00	156.98	Zinc-containing alcohol dehydrogenase
3422	6.69	0.30	6.91	0.15	8.76	0.41	1.166 up	4.207 up	0.00	31.17	unknown protein with oxidoreductase domain
3462	12.11	0.15	12.22	0.06	11.06	0.24	1.083 up	2.060 down	0.00	30.58	UreF urease accessory protein
3481	10.81	0.10	10.51	0.24	9.67	0.22	1.236 down	2.211 down	0.01	23.75	unknown protein
3488	11.29	0.09	9.77	0.05	9.93	0.21	2.876 down	2.578 down	0.00	55.13	unknown protein
3501	9.18	0.12	8.88	0.13	8.15	0.24	1.227 down	2.035 down	0.01	19.64	unknown protein
3529	10.62	0.10	11.31	0.32	11.99	0.16	1.613 up	2.586 up	0.00	33.87	unknown protein
3568	10.27	0.07	7.85	0.24	8.39	0.27	5.337 down	3.682 down	0.00	62.06	phospholipase A2
3579	7.68	0.00	7.74	0.19	9.71	0.27	1.046 up	4.104 up	0.00	79.80	ATP-dependent RNA helicases.
3596	7.86	0.25	7.31	0.09	8.90	0.43	1.470 down	2.050 up	0.01	14.87	uroporphyrinogen synthase
3605	11.24	0.02	10.78	0.07	9.96	0.17	1.377 down	2.431 down	0.00	67.96	Zn2Cys6 transcriptional regulator
3835	10.40	0.27	10.40	0.01	8.73	0.33	1.002 down	3.172 down	0.00	34.15	3-ketoacyl-CoA thiolase
3889	13.56	0.12	13.42	0.01	12.31	0.33	1.099 down	2.382 down	0.01	21.19	unknown protein
3909	9.25	0.02	10.02	0.11	10.58	0.44	1.710 up	2.528 up	0.03	10.22	Mitochondrial ribosomal protein L43
3914	10.64	0.03	7.12	0.09	6.78	0.89	11.485 down	14.492 down	0.01	22.27	Endoplasmic reticulum protein EP58
4040	11.23	0.04	10.80	0.00	10.10	0.21	1.353 down	2.187 down	0.00	35.16	RNA polymerase Rpc34 subunit
4064	11.58	0.07	11.92	0.04	13.18	0.09	1.267 up	3.025 up	0.00	345.65	Threonine dehydratase
4069	11.64	0.03	11.63	0.09	10.33	0.29	1.008 down	2.480 down	0.00	32.07	AMP-dependent synthetase and ligase

4109	9.86	0.05	10.13	0.00	8.71	0.20	1.204 up	2.228 down	0.00	68.66	Aspartate/tyrosine/aromatic aminotransferase
4114	6.26	0.36	3.21	0.04	5.03	0.19	8.272 down	2.337 down	0.00	96.40	FAD linked oxidase
4124	6.34	0.08	10.60	0.10	9.84	0.11	19.177 up	11.315 up	0.00	1042.30	myb transcriptional regulator
4171	9.30	0.15	6.01	0.14	7.97	0.19	9.788 down	2.519 down	0.00	177.10	calcium transporter
4240	11.22	0.06	8.06	0.05	8.02	0.08	8.960 down	9.188 down	0.00	1420.23	stress response protein Rds1; secreted
4308	12.63	0.13	11.85	0.03	11.62	0.17	1.717 down	2.021 down	0.00	32.90	Aminopeptidase N
4428	10.26	0.15	9.95	0.05	9.19	0.25	1.234 down	2.098 down	0.01	20.69	unknown protein. TPR domain
4442	8.19	0.00	6.96	0.11	6.43	0.27	2.342 down	3.378 down	0.00	43.60	SAM-dependent methyltransferase
4479	11.44	0.06	12.11	0.22	12.65	0.27	1.597 up	2.318 up	0.01	18.26	small nuclear ribonucleoprotein Sm D2
4494	12.22	0.06	11.93	0.08	10.60	0.35	1.224 down	3.085 down	0.00	29.62	unknown protein
4517	5.19	0.02	6.20	0.13	9.04	0.17	2.027 up	14.425 up	0.00	577.36	Cytochrome P450 CYP4/CYP19/CYP26 subfamilies
4561	9.77	0.08	8.32	0.02	8.41	0.19	2.718 down	2.569 down	0.00	64.60	GT α-1.6-mannosyltransferase
4626	13.27	0.26	11.88	0.16	10.95	0.44	2.627 down	5.010 down	0.01	26.50	unknown protein. C2 domain
4677	11.12	0.05	11.06	0.06	9.69	0.18	1.045 down	2.710 down	0.00	92.34	unknown protein
4726	12.92	0.10	12.89	0.11	11.52	0.24	1.022 down	2.629 down	0.00	47.56	Cytochrome P450 monooxygenase
4737	10.89	0.06	9.58	0.02	9.10	0.23	2.471 down	3.457 down	0.00	68.07	arylamine N-acetyltransferase 1
4748	12.15	0.14	12.09	0.11	11.00	0.16	1.042 down	2.213 down	0.00	57.75	C2H2 transcription factor
4847	10.92	0.10	10.62	0.14	9.76	0.10	1.229 down	2.232 down	0.00	83.73	unknown protein
4851	11.76	0.07	4.15	0.23	9.82	0.18	194.650 down	3.832 down	0.00	1034.06	unknown protein
4875	11.39	0.14	10.78	0.01	9.28	0.23	1.521 down	4.324 down	0.00	98.04	carboxy-cis.cis-muconate cyclase
4876	12.36	0.17	11.02	0.00	10.03	0.30	2.534 down	5.033 down	0.00	61.72	catechol dioxygenase
4905	6.27	0.39	8.16	0.27	7.78	0.52	3.713 up	2.852 up	0.03	10.27	unknown protein
4921	12.88	0.17	12.46	0.04	11.80	0.25	1.338 down	2.114 down	0.01	19.91	C2H2 transcriptional regulator
4941	12.34	0.04	11.96	0.00	9.37	0.39	1.301 down	7.840 down	0.00	83.15	phenylacetyl-CoA ligase.
4945	11.80	0.15	11.97	0.02	10.80	0.07	1.121 up	2.001 down	0.00	152.39	MAPKKK mitogen activated protein kinase kinase kinase Ste11
4950	9.73	0.04	9.36	0.00	8.62	0.21	1.291 down	2.153 down	0.00	35.56	urea transporter
4952	6.59	0.24	5.49	0.12	8.11	0.17	2.137 down	2.872 up	0.00	157.24	unknown protein
4999	9.24	0.39	13.25	0.25	12.64	0.23	16.108 up	10.569 up	0.00	133.02	cytochrome P450 monooxygenase
5000	5.37	0.36	6.35	0.04	7.11	0.55	1.974 up	3.348 up	0.03	9.95	MFS permease
5026	8.75	0.02	10.14	0.03	10.74	0.12	2.634 up	3.969 up	0.00	288.54	SSCRP

5063	12.65	0.04	11.93	0.09	11.16	0.25	1.647 down	2.797 down	0.00	39.15	unknown protein
5072	8.79	0.05	9.00	0.08	9.98	0.12	1.160 up	2.286 up	0.00	113.58	unknown protein
5084	11.91	0.06	9.34	0.08	7.62	0.51	5.928 down	19.550 down	0.00	79.21	conidiation-specific protein 10
5182	12.58	0.03	9.89	0.02	10.43	0.15	6.435 down	4.438 down	0.00	311.80	iron-dependent peroxidase
5196	11.13	0.00	11.14	0.03	9.23	0.28	1.006 up	3.749 down	0.00	78.40	C-type cyclin
5275	12.75	0.11	12.79	0.03	10.89	0.06	1.024 up	3.633 down	0.00	800.28	unknown protein with SET domain
5296	10.85	0.09	10.83	0.03	9.75	0.03	1.019 down	2.155 down	0.00	510.87	DHQase_I. Type I 3-dehydroquinase
5308	5.13	0.34	5.20	0.68	6.49	0.42	1.050 up	2.558 up	0.04	7.82	unknown protein
5319	10.23	0.01	9.42	0.04	8.19	0.09	1.747 down	4.094 down	0.00	585.84	unknown protein
5324	10.79	0.14	11.26	0.10	12.02	0.24	1.384 up	2.343 up	0.01	26.97	Ribosomal protein L14b
5330	4.77	0.01	4.76	0.25	5.91	0.25	1.011 down	2.195 up	0.01	26.91	glutathione S-transferase. putative
5366	11.28	0.01	11.29	0.01	9.92	0.63	1.008 up	2.555 down	0.04	7.87	O-methyltransferase. putative
5369	3.51	0.29	3.19	0.36	6.05	1.06	1.250 down	5.811 up	0.03	10.22	Metallocarboxypeptidase. putative
5530	12.84	0.14	12.11	0.14	11.36	0.21	1.651 down	2.780 down	0.00	42.34	unknown protein
5612	9.58	0.08	8.56	0.04	8.06	0.40	2.028 down	2.858 down	0.01	15.90	MFS permease
5651	8.16	0.04	6.47	0.38	6.42	0.29	3.222 down	3.328 down	0.01	27.94	unknown protein
5656	10.62	0.09	10.70	0.15	9.39	0.25	1.057 up	2.348 down	0.00	36.99	MFS permease
5675	12.39	0.08	12.21	0.12	11.31	0.10	1.130 down	2.106 down	0.00	98.52	C2H2 transcriptional regulator
5737	10.10	0.02	10.30	0.01	8.96	0.18	1.148 up	2.194 down	0.00	78.72	tyrosine/serine protein phosphatase. putative
5776	13.87	0.02	12.85	0.02	12.56	0.18	2.029 down	2.478 down	0.00	61.23	glucose-6-phosphate isomerase
5807	8.79	0.18	6.94	0.13	7.18	0.50	3.606 down	3.055 down	0.02	13.68	GH95 α-L-fucosidase
5818	9.88	0.07	8.82	0.05	8.84	0.10	2.086 down	2.049 down	0.00	117.46	DSBA oxidoreductase
5836	10.74	0.03	12.11	0.23	11.79	0.42	2.584 up	2.073 up	0.03	9.20	GH2 β-mannosidase
5888	4.26	0.17	3.77	0.42	5.31	0.25	1.400 down	2.069 up	0.01	22.83	fatty-acid amide hydrolase. putative
5889	10.37	0.45	9.36	0.23	9.26	0.33	2.014 down	2.156 down	0.04	7.69	amidase
5890	4.07	0.13	3.83	0.26	5.61	0.06	1.177 down	2.908 up	0.00	146.41	MFS permease
5912	11.49	0.12	11.19	0.13	10.44	0.18	1.225 down	2.059 down	0.00	34.11	unknown protein
5942	11.37	0.21	10.49	0.13	9.50	0.26	1.845 down	3.665 down	0.00	46.16	unknown protein
5970	11.51	0.07	9.33	0.06	8.77	0.16	4.533 down	6.710 down	0.00	286.94	Amidase
6085	3.77	0.07	5.01	0.27	4.80	0.05	2.362 up	2.041 up	0.00	55.44	Enoyl-CoA hydratase/isomerase. putative

6103	3.80	0.02	4.04	0.32	4.94	0.11	1.179 up	2.196 up	0.00	37.99	MFS multidrug transporter. putative
21152	11.01	0.14	11.46	0.12	12.59	0.29	1.364 up	2.991 up	0.00	33.69	unknown protein
21170	13.33	0.03	13.86	0.30	14.54	0.07	1.440 up	2.300 up	0.00	47.41	Ribosomal protein S12
21221	11.39	0.17	11.45	0.21	10.36	0.08	1.044 up	2.038 down	0.00	60.93	unknown protein
21288	12.68	0.04	12.73	0.04	11.55	0.20	1.035 up	2.182 down	0.00	53.68	Vacuolar segregation protein Pep7
21342	12.35	0.12	12.43	0.08	11.21	0.18	1.056 up	2.204 down	0.00	61.79	TUL1 Golgi-localized RING-finger ubiquitin ligase (E3)
21396	11.40	0.03	10.81	0.04	10.22	0.16	1.497 down	2.263 down	0.00	61.24	sugar isomerase
21412	13.97	0.17	12.89	0.11	12.97	0.12	2.118 down	2.000 down	0.00	50.22	unknown protein
21415	12.60	0.19	11.61	0.08	10.46	0.25	1.989 down	4.412 down	0.00	71.00	unknown protein
21416	13.17	0.08	12.41	0.03	11.47	0.13	1.698 down	3.251 down	0.00	165.59	unknown protein
21422	12.80	0.02	12.78	0.03	11.15	0.08	1.013 down	3.152 down	0.00	594.42	MFS permease
21461	12.27	0.07	11.92	0.15	11.27	0.25	1.270 down	2.003 down	0.01	17.47	ATP-dependent protease La. putative
21498	11.25	0.18	12.40	0.20	12.91	0.03	2.221 up	3.163 up	0.00	120.28	unknown protein
21505	12.87	0.18	12.46	0.11	11.57	0.15	1.330 down	2.467 down	0.00	54.90	G-protein alpha subunit 3 GNA-3
21571	13.26	0.11	12.93	0.09	12.24	0.21	1.253 down	2.019 down	0.01	25.09	unknown protein
21595	10.53	0.14	7.93	0.13	8.56	0.17	6.057 down	3.914 down	0.00	150.98	MFS permease
21758	14.86	0.16	15.08	0.02	13.36	0.20	1.165 up	2.832 down	0.00	92.43	isocitrate lyase
21817	12.81	0.05	11.87	0.19	11.47	0.18	1.918 down	2.520 down	0.00	43.31	unknown protein
21836	13.96	0.01	13.13	0.01	11.84	0.16	1.775 down	4.346 down	0.00	202.44	phosphoglucomutase/phosphomannomutase
21960	11.54	0.12	11.83	0.07	13.05	0.52	1.223 up	2.842 up	0.02	11.40	phospholipase C
22030	12.46	0.02	12.51	0.09	11.17	0.14	1.033 up	2.442 down	0.00	135.52	unknown protein
22035	12.17	0.07	11.40	0.19	10.92	0.14	1.702 down	2.376 down	0.00	49.85	unknown protein
22064	13.86	0.03	13.07	0.01	12.20	0.07	1.726 down	3.163 down	0.00	553.67	DNAJ heat shock family protein
22076	11.93	0.13	12.30	0.19	13.11	0.16	1.294 up	2.266 up	0.00	40.46	lysine decarboxylase
22093	12.88	0.10	11.88	0.05	10.15	0.19	2.004 down	6.633 down	0.00	230.35	Protein farnesyltransferase. alpha subunit
22129	10.92	0.02	12.68	0.01	12.59	0.68	3.396 up	3.195 up	0.04	7.90	GH61 polysaccharide monooxygenase
22168	10.72	0.12	9.71	0.14	9.61	0.25	2.016 down	2.163 down	0.01	19.84	unknown protein
22201	11.38	0.02	11.09	0.29	9.94	0.28	1.223 down	2.717 down	0.01	26.99	unknown protein
22251	10.55	0.04	10.81	0.01	12.58	0.27	1.199 up	4.105 up	0.00	84.11	Mitochondrial carnitine-acylcarnitine carrier protein
22277	9.52	0.03	11.02	0.31	10.63	0.27	2.830 up	2.159 up	0.01	19.53	sideroflexin-1

22283	13.00	0.24	12.76	0.10	11.91	0.39	1.179 down	2.130 down	0.03	9.31	unknown protein
22332	11.84	0.08	12.72	0.13	13.04	0.17	1.838 up	2.292 up	0.00	43.15	small nuclear ribonucleoprotein E
22351	7.22	0.12	8.32	0.21	9.24	0.30	2.142 up	4.051 up	0.00	40.89	legume-like lectin
22386	12.03	0.16	12.13	0.08	14.74	0.11	1.066 up	6.519 up	0.00	520.33	Epl1/Sm1
22388	11.31	0.04	11.39	0.04	10.28	0.19	1.051 up	2.048 down	0.00	48.99	unknown protein
22426	11.61	0.01	11.09	0.16	10.30	0.11	1.434 down	2.484 down	0.00	97.28	unknown protein
22459	11.26	0.03	12.76	0.17	12.47	0.11	2.822 up	2.322 up	0.00	106.23	carboxypeptidase A
22464	12.04	0.16	11.48	0.06	9.07	0.15	1.475 down	7.818 down	0.00	385.49	Bifunctional P-450:NADPH-P450 reductase
22512	8.85	0.04	10.54	0.16	11.74	0.18	3.243 up	7.442 up	0.00	221.40	short chain dehydrogenase/reductase
22532	14.25	0.09	14.09	0.03	13.22	0.27	1.118 down	2.049 down	0.01	20.57	BZIP transcriptional regulator
22559	13.28	0.01	12.81	0.09	12.16	0.24	1.385 down	2.173 down	0.01	24.45	unknown protein
22625	9.72	0.10	10.32	0.04	10.92	0.15	1.520 up	2.304 up	0.00	64.87	Molecular chaperone (DnaJ superfamily)
22654	12.94	0.03	12.11	0.17	10.77	0.19	1.783 down	4.500 down	0.00	119.71	monodehydroascorbate reductase
22667	9.38	0.13	9.93	0.21	10.51	0.07	1.465 up	2.189 up	0.00	57.21	pre-rRNA-processing protein IPI1. putative
22705	11.22	0.00	11.45	0.06	10.05	0.14	1.175 up	2.255 down	0.00	141.99	urease
22707	11.84	0.03	12.08	0.00	12.99	0.09	1.183 up	2.228 up	0.00	226.72	unknown protein
22718	12.11	0.20	12.57	0.01	13.13	0.05	1.375 up	2.028 up	0.00	79.01	Glutaminylpeptide cyclotransferase
22741	10.19	0.17	9.78	0.14	8.85	0.40	1.330 down	2.539 down	0.02	13.17	unknown protein
22785	12.51	0.07	12.29	0.16	10.86	0.13	1.166 down	3.150 down	0.00	153.37	C2H2 transcriptional regulator (amdA ?)
22830	11.16	0.04	10.51	0.06	9.64	0.04	1.569 down	2.876 down	0.00	893.43	Glutathione S-transferase
22841	10.29	0.13	10.40	0.15	11.65	0.34	1.082 up	2.560 up	0.01	22.40	unknown protein
22875	11.95	0.01	11.88	0.02	10.21	0.09	1.047 down	3.346 down	0.00	648.36	unknown protein
23059	12.83	0.65	13.38	0.17	14.35	0.18	1.461 up	2.879 up	0.01	15.66	Ribosomal protein L24/L26
23083	10.60	0.07	11.90	0.08	12.20	0.54	2.470 up	3.030 up	0.03	9.94	Flavoprotein monooxygenase
23115	10.04	0.06	9.14	0.22	11.80	0.38	1.866 down	3.393 up	0.00	53.89	expansin
23124	12.66	0.25	12.89	0.13	11.55	0.17	1.169 up	2.158 down	0.00	47.20	unknown protein
23146	11.72	0.00	11.73	0.01	13.04	0.20	1.007 up	2.499 up	0.00	72.73	unknown protein
23152	10.85	0.00	10.66	0.02	9.75	0.21	1.145 down	2.143 down	0.00	40.13	unknown LMBR1 domain protein
23171	10.52	0.24	12.38	0.07	13.83	0.21	3.634 up	9.894 up	0.00	191.65	NRPS
23193	12.20	0.02	11.71	0.13	13.41	0.17	1.403 down	2.313 up	0.00	110.62	importin ß KapJ

23271	12.72	0.00	13.21	0.08	14.16	0.15	1.398 up	2.713 up	0.00	105.35	ER-derived vesicles protein Erv41
23462	11.90	0.05	11.22	0.12	10.57	0.19	1.605 down	2.515 down	0.00	46.94	unknown protein
23475	13.71	0.01	13.03	0.09	12.61	0.04	1.594 down	2.142 down	0.00	306.64	Peptidase family M28 protein
24586	4.43	0.40	4.74	0.08	5.89	0.40	1.236 up	2.736 up	0.02	13.45	NRPS
25159	13.46	0.07	12.51	0.12	12.41	0.10	1.924 down	2.060 down	0.00	75.53	PTPc. Protein tyrosine phosphatases (PTP)
25739	12.09	0.10	11.93	0.02	10.89	0.10	1.120 down	2.296 down	0.00	149.52	CorA family metal ion transporter [Metarhizium anisopliae ARSEF 23]
26187	3.23	0.03	3.08	0.01	5.63	0.16	1.113 down	5.270 up	0.00	392.37	unknown protein
26255	8.74	0.01	8.55	0.12	7.47	0.42	1.141 down	2.413 down	0.02	12.86	Zn2Cys6 transcriptional regulator
26642	11.03	0.16	9.61	0.09	9.31	0.48	2.673 down	3.309 down	0.02	13.85	MFS permease
26787	11.81	0.04	11.87	0.31	10.76	0.32	1.036 up	2.073 down	0.02	14.26	ADP-ribosylation factor-binding protein Gga2
26871	10.34	0.02	9.90	0.04	9.33	0.29	1.361 down	2.021 down	0.02	14.61	Zn2Cys6 transcriptional regulator
27042	10.63	0.02	9.74	0.10	9.50	0.17	1.852 down	2.192 down	0.00	45.57	unknown protein
27181	13.84	0.08	12.39	0.04	9.65	0.59	2.740 down	18.345 down	0.00	62.51	MFS permease
27219	3.82	0.46	4.79	0.01	5.25	0.11	1.966 up	2.694 up	0.01	27.28	GH27 α-galactosidase
27422	5.33	0.09	6.43	0.15	6.40	0.18	2.149 up	2.109 up	0.00	35.03	RNA helicase DEAD/DEAH box
27697	8.96	0.01	9.13	0.03	7.37	0.38	1.122 up	3.020 down	0.00	32.84	Amidase
27770	10.59	0.03	11.12	0.02	7.86	0.41	1.439 up	6.637 down	0.00	90.90	MFS permease
27939	10.61	0.02	10.75	0.02	8.99	0.22	1.098 up	3.071 down	0.00	96.69	unknown protein
27983	3.59	0.09	3.24	0.25	5.32	0.50	1.272 down	3.331 up	0.01	22.77	PTH11 GPCR
27992	10.75	0.02	12.75	0.07	12.44	0.06	4.000 up	3.240 up	0.00	713.53	PTH11 GPCR
28036	8.49	0.09	6.27	0.35	6.63	0.10	4.652 down	3.610 down	0.00	95.29	MFS permease
29275	6.62	0.29	7.00	0.01	8.31	0.08	1.297 up	3.219 up	0.00	112.54	unknown protein
29439	10.95	0.03	10.09	0.01	9.73	0.25	1.808 down	2.325 down	0.01	25.79	unknown protein
29642	3.90	0.20	4.46	0.17	5.90	0.36	1.468 up	3.998 up	0.00	34.58	Phosphatidylserine decarboxylase
29710	10.58	0.30	10.13	0.21	8.79	0.12	1.362 down	3.454 down	0.00	72.03	unknown protein
29993	10.37	0.11	9.77	0.11	8.58	0.15	1.516 down	3.465 down	0.00	135.07	unknown protein
30084	7.89	0.03	9.33	0.18	9.10	0.20	2.706 up	2.313 up	0.00	40.89	unknown protein
30166	10.42	0.18	9.65	0.07	7.66	0.21	1.698 down	6.766 down	0.00	171.73	unknown protein
30274	8.21	0.35	8.41	0.22	9.85	0.33	1.148 up	3.126 up	0.01	24.33	unknown protein
30465	3.67	0.05	3.03	0.21	5.06	0.23	1.557 down	2.611 up	0.00	78.07	unknown protein. WD-repeats

30578	9.20	0.05	8.57	0.06	7.03	0.26	1.547 down	4.472 down	0.00	85.87	HhH-GPD family base excision DNA repair protein
30759	10.11	0.07	9.19	0.04	8.70	0.53	1.892 down	2.654 down	0.04	7.88	zinc containing alcohol dehydrogenase superfamily
30776	3.14	0.04	3.19	0.02	4.33	0.07	1.036 up	2.281 up	0.00	424.55	alpha-ketoglutarate-dependent sulfonate diounknown proteingenase
31134	12.64	0.16	12.48	0.05	9.83	0.16	1.116 down	7.005 down	0.00	363.54	isoprenylcysteine carboxyl methyltransferase
31210	11.81	0.20	11.86	0.01	9.69	0.24	1.033 up	4.325 down	0.00	103.75	unknown protein
31248	12.02	0.17	11.60	0.03	10.35	0.10	1.341 down	3.187 down	0.00	183.47	Ribonuclease. T2 family
31367	11.81	0.05	11.60	0.01	10.61	0.24	1.156 down	2.285 down	0.00	34.13	unknown protein
31447	4.70	0.12	5.60	0.09	6.22	0.23	1.867 up	2.863 up	0.00	42.40	unknown protein
31481	9.29	0.08	10.55	0.01	10.35	0.09	2.395 up	2.074 up	0.00	163.52	chaperone protein dnaJ 6
32087	5.26	0.52	5.69	0.05	7.28	0.51	1.346 up	4.040 up	0.01	15.86	short chain dehydrogenase/reductase
32212	2.69	0.23	2.47	0.35	4.08	0.05	1.166 down	2.618 up	0.00	63.62	SSCRP
32243	11.55	0.17	11.04	0.25	9.94	0.55	1.428 down	3.056 down	0.03	9.85	Alpha/beta hydrolase
32261	12.43	0.11	11.56	0.12	11.01	0.32	1.832 down	2.678 down	0.01	20.83	unknown protein
32364	12.01	0.05	11.34	0.07	9.30	0.36	1.586 down	6.507 down	0.00	72.82	GDSL lipase
32402	2.80	0.15	2.79	0.22	4.48	0.08	1.009 down	3.214 up	0.00	154.54	glutathione S-transferase GliG. putative
32478	3.09	0.05	3.22	0.01	4.80	0.25	1.093 up	3.281 up	0.00	72.21	unknown protein
32639	5.29	0.29	5.84	0.25	7.57	0.95	1.471 up	4.886 up	0.05	7.36	unknown protein
32747	3.38	0.04	3.89	0.49	4.90	0.08	1.420 up	2.856 up	0.00	32.83	unknown protein
32798	12.43	0.04	12.70	0.14	14.92	0.08	1.207 up	5.624 up	0.00	659.82	unknown protein
32849	11.61	0.02	11.06	0.15	10.24	0.17	1.465 down	2.581 down	0.00	63.16	unknown protein
33029	7.88	0.41	7.88	0.03	10.07	0.83	1.001 up	4.594 up	0.02	10.76	unknown protein
33058	9.03	0.05	10.14	0.27	10.35	0.35	2.148 up	2.491 up	0.02	13.13	unknown protein
33207	10.47	0.09	10.67	0.07	9.32	0.18	1.147 up	2.231 down	0.00	72.10	cation transport protein ChaC. putative
33342	9.37	0.13	9.91	0.17	12.29	0.10	1.461 up	7.618 up	0.00	488.74	Oligosaccharyltransferase. epsilon subunit
33359	10.04	0.17	10.85	0.20	12.06	0.51	1.743 up	4.041 up	0.01	17.30	translation initiation factor SUI1 and density-regulated protein.
33371	4.62	0.07	4.73	0.18	5.98	0.47	1.078 up	2.569 up	0.02	12.14	phosphoglycerate mutase family protein
33387	12.86	0.03	12.36	0.05	11.04	0.32	1.411 down	3.518 down	0.00	41.94	GCN5-related N-acetyltransferase. putative
33723	4.77	0.32	5.75	0.18	6.27	0.45	1.977 up	2.839 up	0.03	10.28	ankyrin repeat-containing protein. putative
33949	4.97	0.36	5.20	0.10	6.31	0.19	1.178 up	2.535 up	0.00	31.05	NADH:cytochrome b5 reductase (CBR). putative
33960	12.26	0.17	12.87	0.26	13.60	0.24	1.529 up	2.528 up	0.01	23.26	prefoldin subunit 6. putative

34183	8.03	0.18	8.59	0.12	9.05	0.38	1.467 up	2.019 up	0.05	7.22	unknown protein
34197	11.81	0.14	11.98	0.07	10.28	0.09	1.125 up	2.879 down	0.00	259.52	BolA-like protein
34252	13.07	0.11	12.27	0.04	10.55	0.16	1.742 down	5.722 down	0.00	260.43	unknown protein
34280	10.49	0.04	11.50	0.13	11.52	0.34	2.015 up	2.031 up	0.03	10.46	unknown protein
34312	4.12	0.03	4.42	0.24	6.78	0.34	1.230 up	6.305 up	0.00	78.81	conidiation protein 6
34327	11.56	0.06	12.33	0.14	12.66	0.25	1.702 up	2.138 up	0.01	18.78	NADH-ubiquinone oxidoreductase B12 subunit. putative
34413	5.09	0.44	5.85	0.66	8.10	0.59	1.686 up	8.071 up	0.01	21.48	unique protein. secreted
34493	14.80	0.13	14.87	0.15	11.84	0.23	1.048 up	7.778 down	0.00	219.01	a-type peptide pheromone precursor hpp1
34726	4.50	0.23	5.48	0.01	5.92	0.09	1.973 up	2.671 up	0.00	82.70	Guanine nucleotide exchange factor for Ras-likeGTPases (RasGEF)
35109	3.67	0.12	3.05	0.17	7.77	0.74	1.535 down	17.094 up	0.00	57.40	unknown protein. contains WD repeats
35137	3.77	0.32	3.26	0.05	4.93	0.20	1.427 down	2.234 up	0.00	46.93	unknown protein
35534	5.06	0.27	5.48	0.41	6.25	0.20	1.338 up	2.277 up	0.02	14.30	unknown protein
35726	4.52	0.09	4.68	0.14	5.99	0.23	1.121 up	2.775 up	0.00	50.94	Subtilisin like protease
35777	13.64	0.03	13.19	0.06	12.38	0.31	1.359 down	2.383 down	0.01	20.49	K(+)/H(+) antiporter 1
36006	12.77	0.10	12.10	0.24	11.38	0.15	1.591 down	2.621 down	0.00	48.86	Serine/Threonine protein kinase
36159	10.53	0.11	10.26	0.10	9.37	0.15	1.202 down	2.237 down	0.00	58.27	unknown protein
36335	10.29	0.02	10.74	0.11	7.33	0.60	1.362 up	7.762 down	0.00	47.37	Zn2Cys6 transcriptional regulator
36468	11.21	0.07	10.31	0.03	9.22	0.26	1.872 down	3.992 down	0.00	64.80	Zn2Cys6 transcriptional regulator
36530	10.15	0.11	10.03	0.08	8.99	0.26	1.084 down	2.227 down	0.01	26.71	unknown protein
36608	10.78	0.01	11.51	0.14	9.67	0.20	1.657 up	2.149 down	0.00	83.69	unknown protein
36703	10.56	0.02	10.15	0.17	9.20	0.32	1.326 down	2.569 down	0.01	21.38	Zn2Cys6 transcriptional regulator
36913	7.61	0.21	8.22	0.08	6.53	0.35	1.525 up	2.103 down	0.01	24.52	Zn2Cys6 transcriptional regulator
37316	3.48	0.27	3.46	0.09	5.23	0.08	1.014 down	3.350 up	0.00	148.72	unknown protein
37525	10.94	0.01	13.30	0.00	12.93	0.08	5.125 up	3.989 up	0.00	950.42	GPCR . contains RGS domain
37783	11.84	0.14	9.91	0.07	8.85	0.47	3.819 down	7.917 down	0.00	42.63	unknown protein
37950	2.71	0.06	2.98	0.31	4.93	0.07	1.199 up	4.659 up	0.00	188.34	unknown protein
38048	9.31	0.05	9.83	0.13	7.98	0.34	1.436 up	2.503 down	0.00	36.14	unknown protein
38080	12.06	0.15	12.08	0.00	10.74	0.21	1.013 up	2.511 down	0.00	57.90	C2H2 transcriptional regulator
38603	11.37	0.03	11.59	0.08	10.11	0.20	1.162 up	2.404 down	0.00	73.87	MFS permease
38640	12.93	0.03	13.01	0.12	11.64	0.27	1.062 up	2.448 down	0.00	37.03	unknown protein

38833	11.96	0.05	11.25	0.05	10.28	0.29	1.641 down	3.197 down	0.00	38.74	unknown protein
38863	11.04	0.02	10.71	0.12	9.97	0.08	1.257 down	2.096 down	0.00	127.44	unknown protein
39221	8.88	0.07	8.92	0.01	7.78	0.54	1.032 up	2.130 down	0.05	7.11	Zn2Cys6 transcriptional regulator
39590	8.89	0.10	9.33	0.03	10.62	0.25	1.352 up	3.305 up	0.00	60.38	Membrane protein Erj5 with DnaJ domain
39606	10.26	0.28	9.65	0.21	9.02	0.34	1.531 down	2.360 down	0.02	11.04	unknown protein
39755	11.04	0.13	10.17	0.04	9.56	0.35	1.818 down	2.781 down	0.01	19.09	GH16 glucan endo-1.3(4)-β-D-glucosidase
40290	8.98	0.12	9.34	0.19	10.29	0.35	1.281 up	2.479 up	0.01	15.93	unknown protein
40346	10.31	0.16	11.29	0.03	11.51	0.27	1.959 up	2.292 up	0.01	19.65	formamidopyrimidine-DNA glycosylase
40775	11.18	0.01	9.90	0.07	9.72	0.17	2.429 down	2.752 down	0.00	79.10	RTA1 protein. 7 TNM. responds to xenobiotic stimulus
40808	5.85	0.37	9.25	0.13	4.52	0.14	10.571 up	2.515 down	0.00	360.37	short-chain dehydrogenase/reductase
40943	11.97	0.09	11.70	0.06	10.15	0.30	1.207 down	3.540 down	0.00	52.12	unknown protein
41001	7.83	0.10	8.01	0.16	9.03	0.29	1.137 up	2.303 up	0.01	21.33	unknown protein with Nif domain
41171	10.29	0.24	10.00	0.01	8.18	0.40	1.220 down	4.325 down	0.00	36.03	unknown protein
41260	3.05	0.10	3.14	0.08	4.52	0.12	1.057 up	2.770 up	0.00	173.50	PTH11 GPCR
41325	7.93	0.16	8.63	0.05	6.86	0.35	1.625 up	2.104 down	0.00	28.80	RTA1 like protein; 7 TM
41425	8.87	0.09	8.75	0.14	7.08	0.37	1.086 down	3.458 down	0.00	34.34	unknown protein
41428	13.26	0.01	13.22	0.09	11.97	0.26	1.027 down	2.445 down	0.00	37.50	Tyrosine specific protein phosphatase and dual specificity protein phosphatase
41504	9.95	0.06	10.03	0.07	11.04	0.20	1.054 up	2.124 up	0.00	44.04	phosphopantetheinyl transferase. putative
41573	13.52	0.14	13.15	0.02	12.46	0.31	1.293 down	2.085 down	0.02	13.70	BZIP transcriptional regulator
41590	11.48	0.18	10.46	0.05	9.66	0.31	2.028 down	3.551 down	0.00	35.28	iron transporter
41617	11.82	0.06	3.28	0.04	14.25	0.38	372.860 down	5.394 up	0.00	928.13	lae1
41699	6.56	0.05	7.81	0.06	8.35	0.40	2.380 up	3.449 up	0.01	21.86	HET protein
41761	13.74	0.14	13.63	0.15	12.60	0.20	1.077 down	2.205 down	0.00	37.38	iron transporter
41768	6.05	0.27	6.28	0.16	7.87	0.65	1.172 up	3.543 up	0.03	10.68	GH16 cell wall glucanosyltransferase
41942	10.25	0.17	11.08	0.15	9.14	0.18	1.770 up	2.165 down	0.00	92.84	unknown protein
42043	2.67	0.13	2.75	0.06	4.48	0.16	1.059 up	3.502 up	0.00	163.43	unknown protein
42152	8.71	0.03	8.13	0.05	6.34	0.24	1.491 down	5.173 down	0.00	132.06	GH75 chitosanase
42181	9.67	0.13	9.90	0.02	10.99	0.35	1.175 up	2.499 up	0.01	18.87	unknown protein
42264	9.50	0.13	10.25	0.19	6.36	0.45	1.681 up	8.807 down	0.00	97.08	3'-5' exonuclease
42326	11.68	0.10	12.17	0.02	10.64	0.42	1.404 up	2.046 down	0.01	16.18	unknown protein

42355	9.31	0.05	9.83	0.13	8.20	0.21	1.434 up	2.154 down	0.00	69.25	unknown protein
42449	5.86	0.14	6.26	0.13	7.29	0.36	1.313 up	2.689 up	0.01	19.17	N-acetyltransferase activity
42571	6.46	0.02	6.20	0.08	7.51	0.27	1.196 down	2.069 up	0.00	32.21	unknown protein
42848	14.26	0.06	12.98	0.04	12.91	0.31	2.421 down	2.534 down	0.01	22.53	unknown protein
42866	12.18	0.05	10.63	0.11	10.57	0.25	2.932 down	3.059 down	0.00	45.77	thioesterase family protein
42919	13.57	0.01	13.88	0.04	14.71	0.12	1.237 up	2.199 up	0.00	118.71	ubiquitin fusion protein
42942	2.23	0.02	2.11	0.05	3.71	0.10	1.087 down	2.798 up	0.00	338.13	unknown protein
43076	12.32	0.08	12.41	0.03	10.92	0.32	1.070 up	2.626 down	0.00	33.19	unknown protein
43083	10.26	0.14	9.95	0.15	11.94	0.43	1.236 down	3.216 up	0.00	29.07	unknown protein
43115	9.52	0.01	11.05	0.12	10.65	0.14	2.885 up	2.190 up	0.00	88.85	SSCRP
43161	8.68	0.13	9.13	0.08	10.36	0.32	1.364 up	3.214 up	0.00	33.29	Carboxylesterase and related proteins
43199	4.03	0.12	4.55	0.35	5.95	0.18	1.442 up	3.792 up	0.00	61.63	unknown protein
43225	11.51	0.14	12.52	0.20	13.24	0.18	2.007 up	3.317 up	0.00	65.31	U6 small nuclear ribonucleoprotein (Lsm3). putative
43236	12.29	0.14	12.83	0.09	13.40	0.21	1.449 up	2.153 up	0.01	25.68	unknown protein
43269	9.43	0.27	11.10	0.33	10.88	0.37	3.176 up	2.723 up	0.01	14.82	bHLH transcriptional regulator
43302	3.68	0.37	3.67	0.17	6.12	0.43	1.007 down	5.446 up	0.00	42.46	unknown protein
43392	5.42	0.08	6.15	0.25	7.97	0.55	1.651 up	5.859 up	0.01	26.27	unique protein
43401	8.59	0.30	9.06	0.33	12.84	0.23	1.387 up	19.063 up	0.00	229.96	unknown protein
43427	2.69	0.17	2.94	0.07	4.25	0.18	1.188 up	2.936 up	0.00	77.73	unknown protein
43701	7.86	0.08	8.85	0.09	10.05	0.36	1.993 up	4.588 up	0.00	41.84	MFS multidrug transporter
43893	12.80	0.21	12.25	0.15	11.56	0.10	1.466 down	2.366 down	0.00	56.48	unknown protein
43974	10.19	0.05	10.87	0.21	11.31	0.26	1.608 up	2.180 up	0.01	16.78	UDP-galactose transporter
44117	13.34	0.15	12.83	0.10	12.00	0.07	1.424 down	2.529 down	0.00	145.01	Heterokaryon incompatibility protein HEC-C
44175	13.28	0.05	10.77	0.02	9.83	0.13	5.701 down	10.981 down	0.00	769.90	MFS H+ sugar transporter
44251	9.96	0.05	9.85	0.10	11.30	0.28	1.078 down	2.544 up	0.00	39.80	GTPase-activating protein Msb3 (Sec4/Rabs)
44278	15.15	0.02	13.74	0.04	12.88	0.20	2.647 down	4.813 down	0.00	140.75	Rab geranyl transferase escort protein
44362	9.48	0.01	12.44	0.06	12.54	0.12	7.751 up	8.294 up	0.00	779.09	unknown protein
44366	9.94	0.00	8.74	0.03	8.82	0.29	2.297 down	2.179 down	0.01	19.55	Glycosylphosphatidylinositol-specific phospholipase C
44459	10.42	0.09	11.47	0.02	12.13	0.14	2.076 up	3.266 up	0.00	143.38	Rfc4p (replication factor C) subunit 4
44476	11.26	0.00	8.94	0.05	9.68	0.40	4.980 down	2.986 down	0.00	30.16	MRP-type ABC transporter

44640	12.15	0.16	13.24	0.01	13.55	0.06	2.128 up	2.630 up	0.00	191.12	unknown protein
44878	12.56	0.08	12.19	0.05	11.29	0.18	1.298 down	2.413 down	0.00	59.01	unknown protein
45250	11.95	0.01	12.52	0.01	14.00	0.13	1.483 up	4.138 up	0.00	335.30	oleate-delta12-desaturase
45512	10.58	0.03	10.97	0.21	11.72	0.30	1.314 up	2.214 up	0.01	15.24	unknown protein
45573	11.23	0.08	11.64	0.03	12.32	0.09	1.329 up	2.118 up	0.00	128.61	PTH11 GPCR
45598	13.30	0.36	12.26	0.04	12.30	0.24	2.064 down	2.003 down	0.02	12.83	unknown protein
45675	12.45	0.02	11.88	0.09	10.71	0.14	1.482 down	3.326 down	0.00	164.98	unknown protein
45717	13.08	0.13	10.91	0.08	10.92	0.27	4.496 down	4.485 down	0.00	75.32	GH47 α-1.2-mannosidase
46057	11.12	0.13	11.27	0.09	12.12	0.19	1.111 up	2.011 up	0.00	33.62	unknown protein
46128	8.24	0.15	6.81	0.08	7.16	0.30	2.682 down	2.114 down	0.01	19.61	AAA ATPase
46320	11.98	0.13	12.50	0.21	13.09	0.17	1.441 up	2.162 up	0.00	29.50	small nuclear ribonucleoprotein LSM2
46443	10.34	0.00	11.04	0.11	9.05	0.38	1.623 up	2.437 down	0.00	31.96	GT α-1.6-mannosyltransferase
46446	12.63	0.09	12.41	0.01	11.49	0.06	1.162 down	2.194 down	0.00	266.99	Thioredoxin binding protein TBP-2
46545	7.01	0.05	8.22	0.21	11.21	0.38	2.305 up	18.397 up	0.00	143.43	Prephenate dehydrogenase
46763	12.42	0.12	12.78	0.05	10.56	0.27	1.281 up	3.639 down	0.00	89.64	endonuclease/exonuclease/phosphatase family protein
46816	8.71	0.07	7.63	0.28	10.41	0.23	2.115 down	3.253 up	0.00	116.09	GH3 β-glucosidase CEL3d
47077	10.59	0.12	9.60	0.11	9.58	0.15	1.986 down	2.020 down	0.00	39.58	diounknown proteingenase
47136	10.93	0.07	11.02	0.04	12.18	0.23	1.064 up	2.384 up	0.00	42.62	Glutathione peroxidase
47268	11.21	0.06	12.44	0.02	12.76	0.29	2.346 up	2.922 up	0.00	32.26	GH3 β-glucosidase BGL3i
47286	13.64	0.10	12.87	0.05	12.40	0.32	1.704 down	2.369 down	0.01	15.76	unknown protein
47315	10.91	0.01	9.74	0.14	9.62	0.27	2.264 down	2.449 down	0.01	25.47	P-type ATPase
47330	10.67	0.11	11.33	0.08	12.72	0.20	1.578 up	4.142 up	0.00	112.69	SSCRP
47479	11.08	0.18	11.38	0.08	9.37	0.37	1.229 up	3.275 down	0.00	39.96	Zn2Cys6 transcriptional regulator
47510	11.79	0.13	12.33	0.25	12.95	0.34	1.451 up	2.230 up	0.02	11.02	Complex 1 LYR protein
47603	10.71	0.13	10.28	0.08	11.77	0.34	1.351 down	2.082 up	0.01	23.36	succinyl-CoA:3-ketoacid-coenzyme A transferase subunit A
47733	11.87	0.05	13.84	0.09	14.21	0.18	3.912 up	5.034 up	0.00	178.55	SSCRP
47795	11.62	0.07	12.11	0.26	12.64	0.24	1.410 up	2.027 up	0.01	14.79	ribosomal protein S10.
47814	12.94	0.00	12.89	0.09	11.64	0.34	1.035 down	2.472 down	0.01	23.69	prenylcysteine lyase. putative
47926	9.50	0.17	10.29	0.12	11.64	0.29	1.723 up	4.383 up	0.00	57.08	ABC1 family protein
48080	11.52	0.05	11.72	0.13	7.84	0.25	1.148 up	12.864 down	0.00	341.98	unknown protein

48211	13.39	0.08	12.47	0.06	11.19	0.14	1.886 down	4.585 down	0.00	236.39	unknown protein. intracellular
48280	9.49	0.34	10.03	0.48	13.57	0.76	1.455 up	16.937 up	0.00	34.96	unknown protein
48295	10.86	0.07	11.76	0.06	12.41	0.39	1.862 up	2.929 up	0.01	17.67	unknown protein
48366	12.79	0.28	13.14	0.03	13.87	0.08	1.269 up	2.111 up	0.00	44.92	26S proteasome regulatory complex subunit Rpn12
48438	12.23	0.05	11.88	0.13	10.48	0.17	1.270 down	3.364 down	0.00	125.44	Zn2Cys6 transcriptional regulator
48792	11.02	0.05	12.15	0.27	12.80	0.15	2.193 up	3.449 up	0.00	73.33	amidase
48951	12.71	0.21	12.37	0.04	11.56	0.10	1.266 down	2.215 down	0.00	66.59	unknown protein
49048	10.91	0.13	9.81	0.07	8.30	0.24	2.152 down	6.111 down	0.00	126.53	RNA-dependent RNA-polymerase
49081	8.73	0.08	7.12	0.10	13.14	0.31	3.051 down	21.255 up	0.00	477.21	GH74 Xyloglucanase CEL74a
49112	12.14	0.10	11.14	0.04	10.64	0.17	2.004 down	2.831 down	0.00	76.38	Glutamate-1-semialdehyde aminotransferase
49149	10.33	0.01	11.20	0.27	11.93	0.26	1.817 up	3.015 up	0.00	32.01	Translocation protein Sec66
49193	12.94	0.04	13.29	0.14	13.99	0.33	1.274 up	2.066 up	0.02	11.75	GH17 glucan 1.3-β-glucosidase
49295	12.23	0.14	12.05	0.02	11.22	0.11	1.128 down	2.004 down	0.00	80.42	unknown protein
49366	12.46	0.18	9.16	0.14	10.27	0.16	9.869 down	4.569 down	0.00	223.32	unknown protein
49517	10.83	0.07	11.38	0.22	12.22	0.17	1.469 up	2.630 up	0.00	49.67	unknown protein
49589	13.23	0.17	12.76	0.00	6.17	0.46	1.387 down	133.610 down	0.00	350.57	unknown protein
49753	11.05	0.14	10.40	0.04	9.40	0.12	1.570 down	3.133 down	0.00	146.60	L-arabinitol 4-dehydrogenase
49832	11.96	0.07	11.75	0.10	10.63	0.12	1.155 down	2.510 down	0.00	137.20	QDE2. Argonaute-like protein. essential for quelling
49864	11.99	0.14	12.39	0.15	13.11	0.12	1.322 up	2.180 up	0.00	55.05	unknown protein
49928	8.73	0.03	8.54	0.07	11.65	0.27	1.144 down	7.578 up	0.00	197.05	unknown protein. only in Sordariomycetes
49976	8.40	0.20	4.56	0.03	13.34	0.39	14.409 down	30.525 up	0.00	558.57	GH5 endo-β-1.4-glucanase EGL5/CEL45a
50077	10.12	0.18	11.21	0.05	11.34	0.25	2.121 up	2.333 up	0.01	23.77	Amino acid transporters
50104	9.38	0.19	10.19	0.14	10.41	0.38	1.752 up	2.040 up	0.05	7.43	phosphatidyl synthase (A. fumigatus)
50215	11.33	0.10	8.66	0.02	12.58	0.30	6.380 down	2.374 up	0.00	186.73	GH16 endo-1.3-β-D-glucosidase/1.3-glucan binding protein
50249	13.26	0.14	12.96	0.00	12.18	0.09	1.231 down	2.103 down	0.00	104.05	peptidase family M3
50323	7.59	0.00	8.75	0.13	9.44	0.45	2.246 up	3.620 up	0.01	18.56	OOC1
50429	9.35	0.22	10.04	0.12	11.16	0.27	1.620 up	3.515 up	0.00	41.38	unknown protein
50516	12.60	0.02	13.30	0.05	13.63	0.04	1.621 up	2.032 up	0.00	505.61	ATPase. V-type
50583	10.25	0.14	9.61	0.08	8.30	0.13	1.556 down	3.869 down	0.00	196.00	Guanine deaminase (GDEase). an aminohydrolase responsible for the conversion ofGu

50593	10.16	0.18	11.63	0.10	11.67	0.04	2.758 up	2.845 up	0.00	182.36	5' nucleotidase
50616	10.40	0.42	10.56	0.19	7.36	1.34	1.118 up	8.243 down	0.04	8.71	Coenzyme A transferase
50625	13.23	0.01	12.96	0.24	12.22	0.16	1.201 down	2.018 down	0.00	30.11	unknown protein
50786	11.63	0.12	13.23	0.26	13.14	0.16	3.020 up	2.845 up	0.00	56.08	Flavin-linked sulfhydryl oxidase
50996	10.82	0.02	8.64	0.14	7.91	0.51	4.528 down	7.482 down	0.00	35.39	unknown protein. C2 domain
51130	11.66	0.13	11.57	0.22	12.78	0.36	1.063 down	2.182 up	0.01	15.22	cytochrome c
51375	11.81	0.01	12.78	0.12	13.40	0.14	1.968 up	3.018 up	0.00	118.93	unknown protein
51407	11.59	0.12	10.78	0.03	10.46	0.21	1.753 down	2.200 down	0.00	29.70	unknown protein
51415	13.00	0.11	12.12	0.07	11.76	0.40	1.841 down	2.361 down	0.03	10.32	unknown protein
51426	12.55	0.12	12.88	0.27	14.01	0.13	1.256 up	2.757 up	0.00	61.75	Ribosomal protein L30 based on homology with similar proteins in other fungi.
51558	12.02	0.05	11.60	0.12	10.63	0.19	1.335 down	2.630 down	0.00	61.53	unknown protein
51603	13.58	0.00	13.45	0.01	12.39	0.11	1.097 down	2.285 down	0.00	180.82	unknown protein
51654	8.76	0.02	8.46	0.13	7.44	0.48	1.231 down	2.496 down	0.03	10.15	unknown protein
51846	12.50	0.01	12.35	0.22	11.27	0.20	1.113 down	2.358 down	0.00	42.05	Zn2Cys6 transcriptional regulator
52073	13.53	0.22	12.28	0.03	12.07	0.18	2.362 down	2.743 down	0.00	48.70	sulfatase
52222	8.32	0.10	7.53	0.05	6.75	0.27	1.733 down	2.984 down	0.00	35.59	ankyrin repeat domain-containing protein
52315	3.50	0.18	3.45	0.42	14.38	0.06	1.038 down	1882.167 up	0.00	2729.49	copper transporter Ctr
52476	7.53	0.13	12.78	0.18	12.10	0.77	38.161 up	23.863 up	0.00	48.07	unknown protein
52499	11.43	0.00	11.38	0.21	9.95	0.13	1.035 down	2.786 down	0.00	112.08	C2H2 transcriptional regulator
52505	11.34	0.02	11.74	0.00	12.37	0.33	1.315 up	2.034 up	0.02	11.51	unknown protein
52875	12.72	0.10	12.12	0.13	10.79	0.35	1.522 down	3.806 down	0.00	35.41	Zn2Cys6 transcriptional regulator
53004	12.10	0.01	12.24	0.11	10.89	0.18	1.103 up	2.307 down	0.00	71.27	protein tyrosine phosphatase activity. M phase of mitotic cell cycle
53029	3.87	0.59	3.71	0.11	4.88	0.22	1.115 down	2.011 up	0.02	11.81	ß-lactamase-like protein
53053	10.19	0.17	12.43	0.03	12.59	0.08	4.738 up	5.307 up	0.00	420.23	unknown protein
53079	10.68	0.16	8.65	0.15	8.21	0.83	4.105 down	5.546 down	0.03	9.83	NADP/FAD dependent oxidoreductase
53153	9.26	0.01	9.14	0.04	7.89	0.23	1.092 down	2.593 down	0.00	55.17	unknown protein
53168	2.80	0.05	2.77	0.05	4.64	0.46	1.024 down	3.572 up	0.01	26.35	Cytochrome P450. putative
53186	5.71	0.05	6.10	0.05	7.82	0.51	1.317 up	4.326 up	0.01	24.10	PL20 glucuronan lyase A
53282	7.05	0.42	6.98	0.06	8.29	0.42	1.047 down	2.361 up	0.02	11.29	NAD(P)H-dependent FMN reductase LOT6. putative
53314	6.12	0.37	6.71	0.09	7.21	0.15	1.507 up	2.126 up	0.01	18.31	unknown protein

53334	8.51	0.08	9.13	0.01	9.60	0.22	1.530 up	2.122 up	0.01	26.49	unknown protein
53360	9.98	0.06	9.02	0.07	8.60	0.34	1.942 down	2.594 down	0.01	17.39	SSCRP
53366	9.94	0.15	10.38	0.03	11.05	0.04	1.353 up	2.151 up	0.00	150.61	translation initiation protein Sua5p.
53372	11.26	0.14	9.80	0.18	10.10	0.16	2.753 down	2.231 down	0.00	46.37	acyltransferase 3
53373	13.32	0.11	13.00	0.22	11.54	0.16	1.248 down	3.435 down	0.00	99.03	CoA-transferase family III
53378	3.49	0.28	4.28	0.23	5.21	0.25	1.733 up	3.298 up	0.00	32.90	siderophore transporter
53401	10.03	0.03	10.29	0.00	8.59	0.30	1.200 up	2.706 down	0.00	47.28	unknown protein
53428	9.57	0.18	9.58	0.10	6.38	0.45	1.005 up	9.099 down	0.00	77.20	SAM-dependent metyhltransferase
53452	2.22	0.03	2.32	0.14	3.99	0.09	1.070 up	3.399 up	0.00	361.91	unknown protein
53475	11.33	0.05	10.27	0.04	9.82	0.09	2.083 down	2.851 down	0.00	271.95	MFS permease
53500	10.94	0.05	10.92	0.01	8.30	0.28	1.012 down	6.223 down	0.00	146.60	unknown protein
53501	11.71	0.06	10.77	0.27	10.64	0.24	1.921 down	2.097 down	0.01	16.52	unknown protein
53525	9.00	0.07	9.30	0.09	10.09	0.14	1.224 up	2.126 up	0.00	67.53	unknown protein (tyrosin phosphatase domain)
53542	13.99	0.00	13.66	0.07	12.53	0.04	1.254 down	2.746 down	0.00	877.04	GH76 α-1.6-mannanase
53561	10.83	0.02	9.51	0.01	8.54	0.38	2.483 down	4.876 down	0.00	40.70	unknown protein
53596	9.97	0.11	10.38	0.08	11.00	0.09	1.330 up	2.044 up	0.00	89.95	unknown protein
53611	10.79	0.11	9.72	0.15	8.52	0.25	2.105 down	4.822 down	0.00	79.84	MFS permease
53615	10.94	0.01	10.47	0.03	9.55	0.18	1.383 down	2.620 down	0.00	73.72	unknown protein with WD repeats
53665	4.80	0.26	5.21	0.10	6.81	0.09	1.329 up	4.049 up	0.00	165.54	unknown protein
53672	12.28	0.01	11.49	0.04	10.83	0.17	1.729 down	2.741 down	0.00	83.44	unknown protein
53673	12.24	0.04	11.36	0.05	13.29	0.27	1.835 down	2.072 up	0.00	57.85	NADP:D-xylose dehydrogenase [Hypocrea jecorina]
53701	10.67	0.05	10.87	0.22	11.94	0.23	1.149 up	2.411 up	0.00	33.28	unknown protein
53747	8.19	0.16	8.74	0.04	6.66	0.43	1.468 up	2.879 down	0.00	29.88	unknown protein
53777	11.75	0.01	10.36	0.21	8.39	0.36	2.621 down	10.284 down	0.00	93.14	unknown protein
53824	11.12	0.08	7.92	0.11	9.00	0.62	9.191 down	4.343 down	0.01	23.12	unknown secreted protein
53859	12.24	0.04	12.01	0.04	10.56	0.14	1.176 down	3.203 down	0.00	196.84	C-4 sterol methyl oxidase
53862	3.54	0.16	4.12	0.05	5.31	0.20	1.501 up	3.430 up	0.00	79.72	unknown protein with WD repeats
53863	6.54	0.12	6.23	0.04	7.93	0.35	1.246 down	2.616 up	0.00	31.97	Flavoprotein monooxygenase
53868	9.12	0.12	8.15	0.07	7.44	0.17	1.960 down	3.207 down	0.00	86.72	NADH:flavin oxidoreductase/NADH oxidase
53888	9.70	0.14	8.72	0.09	8.29	0.08	1.981 down	2.663 down	0.00	137.85	pirin

53903	9.91	0.02	8.92	0.08	6.84	0.53	1.980 down	8.402 down	0.00	42.33	MFS permease
53939	11.24	0.09	11.55	0.22	10.17	0.02	1.238 up	2.099 down	0.00	128.68	DNA polymerase y family member involved in translesion synthesis during DNA repair
53956	12.23	0.02	12.53	0.10	11.14	0.24	1.231 up	2.129 down	0.00	43.55	unknown protein
53961	11.44	0.11	7.67	0.05	8.37	0.34	13.697 down	8.413 down	0.00	116.41	Aspartyl protease
53964	3.69	0.26	4.24	0.10	6.28	0.45	1.471 up	6.049 up	0.00	39.50	oxalate decarboxylase
53989	11.09	0.15	10.71	0.13	9.71	0.30	1.298 down	2.593 down	0.01	24.27	pfkB family kinase. putative
54048	10.23	0.05	8.90	0.19	7.82	0.34	2.510 down	5.314 down	0.00	50.05	unknown protein
54052	7.02	0.67	8.60	0.28	11.25	0.45	2.977 up	18.694 up	0.00	57.95	unknown protein
54089	5.43	0.36	5.88	0.11	7.38	0.32	1.367 up	3.852 up	0.00	34.61	unknown protein
54128	10.79	0.16	10.71	0.12	9.48	0.24	1.056 down	2.477 down	0.00	38.54	IlvA Threonine dehydratase
54144	9.46	0.05	9.33	0.10	11.11	0.08	1.097 down	3.141 up	0.00	470.57	ferric reductase
54160	3.12	0.07	2.99	0.04	4.95	0.13	1.095 down	3.539 up	0.00	298.65	GliI [Aspergillus fumigatus]
54166	12.55	0.13	12.31	0.09	11.01	0.30	1.179 down	2.905 down	0.00	34.39	Cytochrome P450
54179	4.69	0.27	4.79	0.03	6.19	0.08	1.067 up	2.827 up	0.00	119.04	glutathione S-transferase
54202	11.23	0.09	11.09	0.10	10.04	0.18	1.098 down	2.275 down	0.00	57.10	unknown protein. contains DUF155 domain
54226	10.39	0.14	9.91	0.05	11.70	0.19	1.394 down	2.478 up	0.00	96.21	unknown protein
54227	9.66	0.05	6.46	0.02	6.73	0.50	9.221 down	7.643 down	0.00	45.89	short-chain dehydrogenase/reductase
54230	9.27	0.03	8.44	0.09	7.60	0.56	1.777 down	3.192 down	0.03	10.05	NACHT domain WD40 repeat-containing protein. related to HET
54239	11.12	0.04	8.87	0.13	9.37	0.34	4.733 down	3.356 down	0.00	40.13	Multicopper oxidases
54285	11.88	0.08	11.57	0.01	10.37	0.11	1.239 down	2.840 down	0.00	219.80	unknown protein
54335	12.53	0.15	12.12	0.02	11.08	0.22	1.329 down	2.742 down	0.00	47.59	unknown protein
54349	11.44	0.02	12.16	0.12	13.12	0.31	1.637 up	3.184 up	0.00	32.35	small nuclear ribonucleoprotein F
54352	10.88	0.20	9.54	0.12	7.14	0.38	2.527 down	13.359 down	0.00	106.81	unknown protein.
54366	8.59	0.19	8.36	0.04	7.03	0.26	1.169 down	2.940 down	0.00	44.69	ceramidase. nonlysosomal
54426	6.54	0.18	6.74	0.11	7.84	0.26	1.145 up	2.457 up	0.00	29.53	unknown protein
54444	10.03	0.21	10.02	0.12	8.44	0.40	1.005 down	3.004 down	0.01	23.57	unknown protein
54461	3.62	0.44	3.54	0.08	6.57	1.36	1.056 down	7.740 up	0.04	7.78	MFS permease
54462	4.15	0.36	4.80	0.02	6.02	0.11	1.567 up	3.654 up	0.00	78.93	unknown protein
54469	11.85	0.02	11.62	0.10	10.45	0.16	1.174 down	2.646 down	0.00	100.97	Vacuolar transporter chaperone 4 (vtc4). putative
54550	9.43	0.02	9.43	0.01	8.33	0.10	1.001 down	2.149 down	0.00	185.46	short chain dehydrognease/reductase

54611	12.45	0.17	12.57	0.09	14.11	0.18	1.086 up	3.175 up	0.00	99.58	unknown protein
54622	6.21	0.10	4.86	0.06	8.44	0.37	2.553 down	4.686 up	0.00	111.46	unknown protein
54633	13.01	0.02	12.59	0.01	11.88	0.35	1.339 down	2.183 down	0.02	12.87	GT ß-glycosyltransferases
54659	12.91	0.18	12.52	0.08	11.06	0.56	1.315 down	3.606 down	0.02	14.13	unknown protein
54667	9.67	0.07	12.33	0.13	13.25	0.31	6.311 up	11.946 up	0.00	141.91	acyl-CoA synthetase
54669	4.89	1.18	5.53	0.10	8.14	0.28	1.552 up	9.480 up	0.01	26.79	Golgi matrix protein. rud3. involved in the structural organization of the cis-Golgi
54674	11.59	0.00	12.05	0.08	10.56	0.29	1.375 up	2.033 down	0.00	31.52	unknown protein
54676	9.75	0.17	9.97	0.25	10.90	0.46	1.169 up	2.232 up	0.04	7.58	COX17. cytochrome C oxidase assembly protein
54694	11.81	0.02	9.73	0.09	8.39	0.30	4.224 down	10.692 down	0.00	136.18	aryl-alcohol dehydrogenases
54703	11.98	0.01	10.57	0.00	10.65	0.21	2.647 down	2.513 down	0.00	50.71	C2H2 transcriptional regulator
54723	2.95	0.14	2.91	0.03	4.38	0.03	1.025 down	2.698 up	0.00	460.57	SSCRP
54761	5.79	0.13	3.84	0.08	7.53	1.13	3.862 down	3.324 up	0.02	11.89	quercetin 2.3-diounknown proteingenase
54768	11.25	0.09	13.93	0.07	13.34	0.17	6.432 up	4.254 up	0.00	217.38	unknown protein
54784	2.85	0.07	2.92	0.06	4.70	0.10	1.049 up	3.593 up	0.00	423.61	unknown protein
54789	4.33	0.17	4.88	0.34	5.96	0.55	1.464 up	3.105 up	0.03	9.66	short chain dehydrogenase/reductase
54790	8.87	0.16	8.39	0.00	7.16	0.25	1.395 down	3.263 down	0.00	53.39	unknown protein
54846	9.26	0.10	9.22	0.20	7.51	0.34	1.025 down	3.366 down	0.00	38.23	half-sized ABC transporter
54870	12.57	0.12	12.53	0.04	11.47	0.26	1.025 down	2.143 down	0.01	27.18	acetate—CoA ligase
54893	12.97	0.16	13.15	0.05	11.73	0.26	1.138 up	2.349 down	0.00	38.18	unknown protein
54902	7.59	0.04	7.68	0.00	5.94	0.51	1.062 up	3.137 down	0.01	18.63	unknown protein
54954	6.53	0.42	6.31	0.17	5.07	0.31	1.163 down	2.740 down	0.01	18.86	MRP-type ABC transporter
54972	8.47	0.05	6.77	0.03	5.49	0.14	3.231 down	7.847 down	0.00	508.28	MFS permease
55034	13.17	0.02	12.22	0.17	11.57	0.24	1.932 down	3.027 down	0.00	41.50	unknown protein
55036	6.89	0.06	7.93	0.17	8.88	0.29	2.055 up	3.968 up	0.00	47.40	unknown protein
55039	5.97	0.00	7.79	0.37	7.71	0.21	3.534 up	3.347 up	0.00	43.72	unknown protein
55041	10.32	0.22	10.16	0.01	9.24	0.18	1.115 down	2.113 down	0.00	33.85	unknown protein
55060	9.86	0.09	11.18	0.07	10.87	0.19	2.487 up	2.002 up	0.00	42.09	kinesin heavy chain
55105	9.04	0.07	8.08	0.16	7.75	0.13	1.935 down	2.440 down	0.00	64.85	Zn2Cys6 transcriptional regulator
55126	10.02	0.11	8.82	0.09	8.77	0.36	2.304 down	2.382 down	0.02	14.38	unknown protein
55172	9.16	0.01	9.56	0.02	10.83	0.16	1.324 up	3.179 up	0.00	141.66	unknown protein

55193	9.43	0.14	8.36	0.11	7.88	0.29	2.101 down	2.923 down	0.01	28.26	unknown protein
55213	9.00	0.04	9.09	0.08	7.70	0.35	1.066 up	2.472 down	0.01	24.21	tRNA-specific adenosine deaminase
55240	11.61	0.22	8.84	0.01	9.82	0.27	6.843 down	3.475 down	0.00	75.62	large-conductance mechanosensitive channel
55272	13.40	0.01	14.67	0.10	14.62	0.07	2.401 up	2.322 up	0.00	220.27	unknown protein
55279	11.94	0.03	11.24	0.23	10.46	0.14	1.618 down	2.786 down	0.00	67.25	unknown protein
55353	12.32	0.04	12.02	0.07	10.47	0.13	1.233 down	3.611 down	0.00	268.80	pathotenate kinase
55362	10.38	0.11	9.15	0.19	9.37	0.09	2.356 down	2.012 down	0.00	66.68	Heat shock protein 70
55374	4.87	0.02	4.49	0.28	5.94	0.07	1.298 down	2.104 up	0.00	89.51	unknown protein
55407	10.82	0.04	9.82	0.05	9.71	0.28	2.000 down	2.158 down	0.01	18.62	MFS permease
55443	9.93	0.09	11.09	0.13	11.09	0.35	2.239 up	2.233 up	0.02	12.66	unknown protein
55454	11.89	0.08	11.63	0.16	10.28	0.13	1.195 down	3.038 down	0.00	131.81	unknown protein
55566	7.77	0.00	8.12	0.06	8.83	0.27	1.272 up	2.085 up	0.01	18.95	multicopper oidase
55584	12.69	0.20	12.51	0.10	11.66	0.19	1.131 down	2.033 down	0.00	28.62	unknown protein
55589	11.34	0.08	12.27	0.22	12.87	0.19	1.915 up	2.906 up	0.00	47.10	3' exoribonuclease involved in RNA processing during translation.
55595	12.29	0.03	12.68	0.03	13.32	0.15	1.311 up	2.042 up	0.00	55.43	Ca2+ transporter
55597	12.96	0.01	12.49	0.19	11.48	0.40	1.384 down	2.787 down	0.01	16.49	unknown protein
55599	11.04	0.00	10.26	0.03	9.87	0.13	1.710 down	2.246 down	0.00	92.06	RNA 12 protein
55630	10.45	0.32	8.50	0.01	7.48	0.17	3.870 down	7.837 down	0.00	159.88	monocarboxylate transporter
55631	13.01	0.16	12.39	0.00	11.81	0.26	1.531 down	2.294 down	0.01	22.27	phospholipase
55634	7.01	0.54	6.61	0.04	5.69	0.09	1.319 down	2.506 down	0.01	21.39	MFS permease
55636	10.40	0.09	9.60	0.17	9.07	0.24	1.750 down	2.511 down	0.00	28.95	MRP-type ABC transporter
55637	9.83	0.28	9.06	0.02	8.38	0.29	1.710 down	2.742 down	0.01	21.16	Unknown protein with RNA-dependent RNA polymerase (RdRP) and DEXDc/DEAD-like
55666	3.59	0.10	3.67	0.45	5.67	0.03	1.057 up	4.246 up	0.00	96.97	unknown protein with WD repeats
55671	4.37	0.35	5.10	0.38	5.53	0.11	1.665 up	2.237 up	0.01	14.72	unknown protein
55719	5.40	0.17	6.63	0.11	6.61	0.34	2.340 up	2.304 up	0.01	14.66	Protein kinase
55782	5.69	0.49	6.05	0.27	8.13	0.31	1.283 up	5.432 up	0.00	42.42	dipeptidyl peptidase 5
55802	8.68	0.02	9.12	0.07	11.46	0.65	1.351 up	6.833 up	0.01	25.69	GH76 α-1.6-mannanase
55871	6.59	0.19	4.80	0.03	5.02	0.24	3.474 down	2.975 down	0.00	49.29	Short-chain dehydrogenase/reductase
55887	6.50	0.20	8.77	0.10	11.77	0.62	4.792 up	38.374 up	0.00	80.82	unknown protein. secreted
55999	8.14	0.04	7.07	0.10	9.36	0.25	2.100 down	2.335 up	0.00	93.12	GH27 α-galactosidase

56026	10.92	0.01	10.44	0.05	9.44	0.36	1.396 down	2.779 down	0.01	20.38	unknown protein
56064	14.14	0.12	13.91	0.12	12.81	0.11	1.179 down	2.519 down	0.00	119.36	unknown protein
56093	2.91	0.02	3.22	0.09	4.58	0.19	1.247 up	3.202 up	0.00	96.95	unknown protein
56117	12.28	0.14	12.36	0.07	10.93	0.16	1.054 up	2.548 down	0.00	92.61	unknown protein
56141	12.39	0.13	11.59	0.06	10.76	0.59	1.738 down	3.095 down	0.04	8.66	unknown protein
56211	8.69	0.12	11.09	0.18	10.98	0.17	5.270 up	4.898 up	0.00	150.26	unknown protein
56218	11.43	0.05	11.97	0.12	10.00	0.18	1.453 up	2.688 down	0.00	131.31	unknown protein
56236	6.42	0.37	6.15	0.45	7.87	0.57	1.201 down	2.746 up	0.03	9.86	SAM-dependent methyltransferases
56256	12.38	0.15	12.33	0.08	11.28	0.20	1.034 down	2.133 down	0.00	38.86	unknown protein
56259	3.29	0.24	3.79	0.37	5.09	0.24	1.422 up	3.484 up	0.00	35.31	unknown protein
56278	3.52	0.04	3.81	0.39	5.41	0.40	1.217 up	3.688 up	0.01	24.71	unknown protein. in Sordariomycetes
56289	7.17	0.07	7.66	0.06	6.05	0.49	1.404 up	2.177 down	0.02	13.76	MFS permease
56326	6.23	0.11	6.69	0.33	7.76	0.42	1.370 up	2.884 up	0.02	13.76	Zinc-containing alcohol dehydrogenase
56350	4.55	0.13	4.32	0.15	5.85	0.19	1.175 down	2.450 up	0.00	66.94	cysteine synthase. putative
56376	7.86	0.19	8.78	0.03	9.00	0.21	1.888 up	2.202 up	0.01	26.77	unknown protein
56390	13.42	0.00	12.39	0.07	11.77	0.31	2.036 down	3.134 down	0.00	31.42	unknown protein. TPR repeats
56399	12.53	0.03	11.79	0.08	10.98	0.23	1.669 down	2.925 down	0.00	50.08	unknown protein
56408	12.30	0.06	12.42	0.01	11.26	0.22	1.081 up	2.056 down	0.00	40.20	unknown protein
56426	12.17	0.01	12.01	0.23	11.13	0.14	1.116 down	2.044 down	0.00	38.92	GCPR. mPR-type
56427	9.63	0.16	7.92	0.12	8.57	0.27	3.269 down	2.089 down	0.00	30.08	Arylacetamide deacetylase
56434	5.33	0.02	4.92	0.07	7.22	0.09	1.327 down	3.728 up	0.00	790.24	FKBP-type peptidyl-prolyl isomerase. putative
56448	6.82	0.09	5.56	0.04	8.23	0.11	2.402 down	2.663 up	0.00	579.00	GH18 chitinase CHI18-11
56467	7.09	0.18	7.83	0.01	8.37	0.10	1.678 up	2.430 up	0.00	87.54	Mitochondrial deoxynucleotide carrier protein
56469	11.29	0.18	11.51	0.08	9.94	0.03	1.172 up	2.548 down	0.00	269.90	unknown protein
56499	8.67	0.23	9.48	0.11	10.24	0.41	1.754 up	2.959 up	0.01	14.90	N-acyl-phosphatidylethanolamine-hydrolyzing phospholipase D. putative
56515	9.47	0.09	8.65	0.18	6.25	0.43	1.767 down	9.316 down	0.00	67.97	Regulation of HR towards crossover
56546	10.44	0.06	12.54	0.30	12.94	0.12	4.290 up	5.674 up	0.00	158.05	unknown protein. 3TM
56572	12.24	0.02	11.77	0.10	9.98	0.23	1.383 down	4.784 down	0.00	119.48	aminotransferase. classes I and II family
56587	10.36	0.11	10.03	0.01	7.95	0.21	1.259 down	5.312 down	0.00	174.73	GCN5-related N-acetyltransferase
56625	12.23	0.08	12.37	0.13	10.90	0.24	1.101 up	2.505 down	0.00	51.32	CoA-transferase family III

56646	9.02	0.05	8.64	0.01	7.47	0.48	1.307 down	2.940 down	0.02	13.82	monoamine oxidase
56671	7.62	0.20	8.53	0.02	8.85	0.43	1.885 up	2.349 up	0.04	8.60	unknown protein
56726	9.58	0.17	9.94	0.10	11.02	0.12	1.287 up	2.717 up	0.00	103.61	Branched chain alpha-keto acid dehydrogenase complex. alpha subunit
56758	9.59	0.06	10.00	0.03	11.07	0.23	1.330 up	2.799 up	0.00	54.97	unknown protein
56819	12.28	0.08	10.93	0.05	10.51	0.40	2.546 down	3.415 down	0.01	21.51	glutathione-S-transferase
56830	11.98	0.05	10.99	0.06	10.69	0.35	1.988 down	2.437 down	0.01	15.20	dipeptidyl peptidase 5
56831	11.38	0.07	11.70	0.15	12.45	0.19	1.247 up	2.100 up	0.00	31.92	Co-chaperone Hsc20
56840	9.02	0.01	6.32	0.14	11.77	0.62	6.499 down	6.709 up	0.00	86.62	unknown protein
56853	10.35	0.02	9.27	0.15	8.38	0.58	2.107 down	3.913 down	0.02	12.60	unknown protein
56860	11.21	0.01	10.24	0.00	9.37	0.31	1.964 down	3.588 down	0.00	39.91	unknown protein
56863	13.75	0.06	11.13	0.12	12.07	0.16	6.161 down	3.218 down	0.00	178.66	unknown protein
56894	2.88	0.23	3.09	0.17	4.26	0.10	1.156 up	2.606 up	0.00	72.96	GH18 chitinase CHI18-10
56996	4.93	0.29	4.85	0.10	8.93	0.21	1.054 down	15.988 up	0.00	362.01	GH5 β-Mannanase MAN1
57002	12.41	0.18	12.07	0.24	10.88	0.24	1.266 down	2.875 down	0.00	35.74	unknown protein
57008	5.24	0.40	5.55	0.15	6.96	0.49	1.238 up	3.284 up	0.02	13.77	unknown protein
57045	7.48	0.45	6.61	0.00	6.40	0.25	1.818 down	2.101 down	0.03	9.96	unknown protein
57049	12.79	0.04	12.13	0.01	11.52	0.31	1.578 down	2.408 down	0.01	19.50	tRNA selenocysteine-associated protein 1
57101	4.84	0.35	5.41	0.36	6.73	0.15	1.475 up	3.705 up	0.00	42.76	PTH11 GPCR
57112	9.08	0.17	8.87	0.10	7.80	0.41	1.156 down	2.420 down	0.02	12.86	HET and Ankyrin domain protein
57128	12.17	0.14	11.50	0.05	10.93	0.29	1.584 down	2.356 down	0.01	18.68	GH13 glycogen debranching enyzme
57179	5.10	0.27	5.27	0.06	8.18	0.57	1.125 up	8.449 up	0.00	42.77	GH105/GH88 glycosyl hydrolase
57185	7.09	0.18	9.06	0.15	8.36	0.64	3.912 up	2.408 up	0.04	7.87	Amino acid transporters
57198	10.39	0.09	9.74	0.19	8.55	0.27	1.567 down	3.568 down	0.00	47.77	Rad10
57286	8.85	0.11	9.96	0.19	10.10	0.38	2.161 up	2.381 up	0.02	11.46	unknown protein
57322	11.88	0.01	11.87	0.00	10.31	0.48	1.005 down	2.955 down	0.01	17.36	ARO Transcriptional regulators containing a DNA-binding HTH domain and an aminotr[illegible]
57357	8.69	0.11	8.86	0.07	10.10	0.29	1.124 up	2.654 up	0.00	32.22	Glycine cleavage T protein (aminomethyl transferase). putative
57383	8.82	0.01	7.94	0.14	7.53	0.19	1.846 down	2.443 down	0.00	43.71	unknown protein
57397	6.76	0.18	7.98	0.11	8.28	0.42	2.316 up	2.850 up	0.02	13.47	unknown protein
57433	3.24	0.01	3.19	0.16	4.63	0.05	1.035 down	2.620 up	0.00	299.84	Peptidase S8 and S53. subtilisin. kexin. sedolisin
57465	12.29	0.03	12.19	0.10	11.13	0.13	1.074 down	2.235 down	0.00	104.32	Vsp9 domain protein

57488	12.93	0.18	12.73	0.02	11.70	0.26	1.149 down	2.350 down	0.01	27.75	Top3 gene
57524	12.44	0.12	12.02	0.05	11.39	0.18	1.336 down	2.065 down	0.00	35.65	amidohydrolase 2
57526	9.65	0.09	6.60	0.04	7.15	0.17	8.271 down	5.647 down	0.00	291.59	GPCR. mating type pheromone G-protein coupled receptor
57527	10.16	0.18	10.12	0.01	8.47	0.22	1.029 down	3.217 down	0.00	78.91	unknown protein
57537	11.64	0.03	12.02	0.23	12.78	0.21	1.299 up	2.213 up	0.01	27.11	unknown protein
57555	9.34	0.06	7.89	0.07	7.60	0.63	2.737 down	3.347 down	0.04	8.66	cytochrome P450 monooxygenase
57568	13.46	0.09	13.41	0.01	12.24	0.33	1.042 down	2.344 down	0.01	21.32	unknown protein
57575	4.83	0.22	5.06	0.44	6.37	0.39	1.168 up	2.904 up	0.02	14.45	unknown protein
57592	11.76	0.08	11.74	0.00	10.51	0.15	1.009 down	2.373 down	0.00	100.45	unknown protein
57603	12.83	0.22	12.30	0.03	11.76	0.08	1.449 down	2.098 down	0.00	58.36	unknown protein
57643	10.95	0.09	10.15	0.22	6.73	0.26	1.743 down	18.548 down	0.00	285.83	unknown protein
57735	12.87	0.02	12.39	0.05	11.85	0.24	1.398 down	2.025 down	0.01	20.26	GATA type transcriptional regulator
57749	10.71	0.10	9.68	0.10	8.80	0.43	2.035 down	3.764 down	0.01	21.78	MFS permease
57760	11.59	0.03	11.16	0.09	10.05	0.22	1.349 down	2.915 down	0.00	61.82	Zn2Cys6 transcriptional regulator
57860	11.60	0.02	11.40	0.05	9.77	0.15	1.153 down	3.578 down	0.00	225.07	Multicopper oxidases
57865	7.75	0.20	8.10	0.14	9.24	0.21	1.281 up	2.816 up	0.00	46.53	unknown protein
57868	8.31	0.03	9.60	0.04	9.35	0.19	2.442 up	2.062 up	0.00	42.56	Delta 1-pyrroline-5-carboxylate reductase
57870	10.16	0.03	11.42	0.18	11.80	0.28	2.398 up	3.119 up	0.00	34.63	histone H3
57891	12.23	0.02	12.12	0.09	11.10	0.11	1.078 down	2.186 down	0.00	125.15	actinin actin binding and calcium-binding EF-hand domains
57914	11.52	0.03	11.73	0.17	7.31	0.29	1.158 up	18.424 down	0.00	333.80	Esterase/lipase/thioesterase
57965	12.25	0.02	12.49	0.02	11.11	0.10	1.179 up	2.203 down	0.00	258.03	DSBA family oxidoreductase. putative
57975	7.05	0.29	7.90	0.03	8.78	0.20	1.804 up	3.326 up	0.00	52.08	phospholipase C. related to Aspergillus fumigatus phosphatidylinositol phospholipase
58016	5.03	0.31	5.53	0.33	6.67	0.09	1.417 up	3.118 up	0.00	43.99	unknown protein
58026	10.53	0.13	10.41	0.25	9.10	0.28	1.086 down	2.694 down	0.00	29.50	glutathione S-transferase domain-containing protein
58051	10.50	0.06	10.03	0.08	9.12	0.30	1.387 down	2.606 down	0.01	25.60	unknown protein
58130	12.99	0.00	12.35	0.01	11.82	0.06	1.563 down	2.246 down	0.00	384.56	bHLH transcriptional regulator
58227	5.12	0.25	6.29	0.30	7.12	0.31	2.248 up	4.000 up	0.00	30.50	GCN5-related acetyltransferase
58229	11.11	0.12	12.20	0.01	12.13	0.08	2.134 up	2.030 up	0.00	120.50	Ubiquitin-like protein (HubA)
58244	11.80	0.22	10.88	0.00	9.61	0.27	1.892 down	4.551 down	0.00	62.91	unknown protein
58264	9.47	0.00	10.27	0.15	10.83	0.09	1.747 up	2.575 up	0.00	132.85	paxU orthologue ? (indole-terpene biosynthesis?)

58267	9.48	0.08	9.99	0.11	11.07	0.12	1.428 up	3.018 up	0.00	161.89	tRNA-dihydrouridine synthase.
58285	7.34	0.13	6.36	0.25	6.28	0.24	1.981 down	2.096 down	0.01	16.42	PKS-NRPS
58296	3.94	0.02	4.09	0.05	5.36	0.34	1.107 up	2.676 up	0.01	26.87	MFS permease
58333	8.23	0.13	7.63	0.13	7.04	0.20	1.519 down	2.281 down	0.00	32.69	FAD binding protein
58366	3.84	0.14	3.48	0.11	5.40	0.33	1.280 down	2.944 up	0.00	42.64	AAA+-type ATPase
58370	10.26	0.11	9.36	0.08	9.03	0.18	1.865 down	2.339 down	0.00	43.72	mtDNA repair protein
58387	11.13	0.16	10.80	0.03	9.92	0.09	1.261 down	2.312 down	0.00	108.01	proline iminopeptidase
58412	7.15	0.34	5.26	0.33	5.35	0.15	3.697 down	3.471 down	0.00	42.07	AdhP Zn-dependent alcohol dehydrogenases
58431	3.82	0.50	4.30	0.05	5.38	0.17	1.400 up	2.947 up	0.01	27.42	unknown protein
58450	4.42	0.15	4.65	0.28	5.65	0.31	1.176 up	2.349 up	0.01	16.02	GH3 β-xylosidase XYL3b
58456	11.87	0.09	8.66	0.14	9.45	0.19	9.297 down	5.379 down	0.00	210.31	Zn2Cys6 transcriptional regulator
58479	7.77	0.19	7.26	0.02	6.02	0.29	1.418 down	3.365 down	0.00	41.47	short chain dehydrogenase/reductase
58492	9.80	0.05	10.28	0.30	10.94	0.43	1.397 up	2.204 up	0.05	7.08	unknown protein
58509	9.51	0.16	9.80	0.08	8.29	0.18	1.223 up	2.335 down	0.00	72.71	DNA ligase IV involved in non-homologous end joining of double-strand DNA breaks
58511	12.34	0.15	10.58	0.09	9.00	0.31	3.383 down	10.118 down	0.00	117.99	amino acid permease (GABA)
58521	3.48	0.09	3.69	0.07	5.32	0.33	1.160 up	3.571 up	0.00	43.52	SAM (and some other nucleotide) binding motif
58525	9.95	0.05	8.46	0.05	8.21	0.36	2.797 down	3.337 down	0.01	26.49	unknown protein
58535	3.48	0.18	4.04	0.12	6.53	0.32	1.475 up	8.270 up	0.00	108.76	unknown protein
58560	10.20	0.14	9.89	0.05	9.13	0.34	1.242 down	2.103 down	0.02	12.11	MFS monocarboxylate transporter
58563	4.47	0.16	4.48	0.16	5.52	0.17	1.004 up	2.070 up	0.00	40.40	oxalate decarboxylase
58574	5.98	0.48	6.11	0.10	7.36	0.49	1.092 up	2.601 up	0.03	9.18	unknown protein
58601	3.29	0.08	2.96	0.02	5.25	0.14	1.251 down	3.900 up	0.00	345.05	unknown protein
58603	9.61	0.06	9.71	0.04	11.84	0.19	1.070 up	4.702 up	0.00	207.27	unknown protein. contains Splecktrin motif
58634	11.64	0.04	11.05	0.06	9.92	0.15	1.499 down	3.290 down	0.00	152.86	Zn2Cys6 Fungal transcriptional regulator
58639	11.60	0.07	8.20	0.08	10.23	0.26	10.563 down	2.584 down	0.00	141.20	unknown protein
58640	9.82	0.01	10.42	0.32	11.39	0.26	1.516 up	2.972 up	0.00	29.24	unknown protein
58651	8.26	0.32	8.14	0.35	9.64	0.07	1.084 down	2.597 up	0.00	43.37	adenylosuccinate synthase
58675	7.64	0.15	6.98	0.24	5.59	0.57	1.577 down	4.152 down	0.01	15.22	short chain dehydrogenase/reductase
58689	8.40	0.18	8.05	0.11	6.96	0.28	1.277 down	2.717 down	0.00	30.31	ERG5 C-22 sterol desaturase. a cytochrome P450 enzyme that catalyzes the formation
58694	10.65	0.02	9.88	0.12	9.49	0.26	1.700 down	2.232 down	0.01	21.30	unknown protein

58699	8.44	0.04	7.69	0.03	7.15	0.15	1.682 down	2.440 down	0.00	78.86	unknown protein
58734	11.64	0.12	12.11	0.05	13.27	0.10	1.390 up	3.106 up	0.00	212.09	prefoldin subunit 2
58746	11.67	0.13	11.25	0.05	10.30	0.28	1.343 down	2.596 down	0.00	28.58	unknown protein
58823	5.99	0.02	6.60	0.18	7.41	0.48	1.531 up	2.674 up	0.03	9.91	MRSP1/expansin-like
58837	11.76	0.07	11.11	0.19	10.33	0.12	1.566 down	2.698 down	0.00	84.39	unknown protein
58848	5.62	0.12	5.23	0.14	6.97	0.44	1.314 down	2.539 up	0.01	20.27	unknown protein
58853	8.05	0.04	9.62	0.11	12.30	0.11	2.977 up	19.052 up	0.00	1288.61	Homeodomain-like
58880	3.72	0.11	3.66	0.19	6.13	0.25	1.041 down	5.309 up	0.00	122.25	MRSP1/expansin-like
58887	3.38	0.12	3.19	0.16	4.86	0.11	1.134 down	2.799 up	0.00	157.84	GH78 α-L-rhamnosidase
58897	13.35	0.04	12.76	0.08	11.55	0.08	1.497 down	3.468 down	0.00	494.37	C2H2 transcriptional regulator
58952	5.80	0.25	5.17	0.09	4.73	0.12	1.547 down	2.103 down	0.00	32.45	calcium transporting ATPase. ion pump
58990	8.94	0.13	9.51	0.06	10.01	0.23	1.491 up	2.112 up	0.01	22.78	v-SNARE Bos1. ER-Golgi
59028	6.69	0.03	7.71	0.05	9.02	0.26	2.016 up	4.997 up	0.00	92.48	unknown protein
59050	3.13	0.25	2.84	0.13	6.41	0.83	1.216 down	9.714 up	0.01	27.29	Ankyrin
59053	12.54	0.20	12.33	0.05	11.34	0.16	1.156 down	2.291 down	0.00	53.14	Adenosine/AMP deaminase
59065	4.17	0.24	4.59	0.01	5.63	0.20	1.339 up	2.752 up	0.00	47.64	unknown protein
59067	9.94	0.08	10.18	0.09	11.20	0.17	1.180 up	2.397 up	0.00	65.44	Zn2Cys6 transcriptional regulator
59073	9.73	0.06	9.58	0.00	8.58	0.24	1.110 down	2.213 down	0.00	34.08	unknown protein
59081	3.17	0.11	3.27	0.00	4.83	0.13	1.066 up	3.150 up	0.00	200.10	CsdB Selenocysteine lyase
59151	14.30	0.21	10.21	0.17	12.06	0.29	17.018 down	4.712 down	0.00	128.31	SSCRP
59188	10.60	0.15	10.93	0.19	8.50	0.22	1.257 up	4.261 down	0.00	126.19	unknown protein
59190	9.99	0.16	9.30	0.16	8.51	0.23	1.614 down	2.775 down	0.00	35.46	MFS permease
59196	7.52	0.11	8.59	0.00	9.54	0.53	2.101 up	4.067 up	0.01	16.18	short chain dehydrogenase/reductase
59234	10.43	0.02	10.02	0.27	8.45	0.49	1.330 down	3.943 down	0.01	20.24	Zn2Cys6 transcriptional regulator
59244	3.98	0.21	4.09	0.05	6.00	0.54	1.079 up	4.037 up	0.01	20.86	metalloprotease. putative
59272	9.49	0.05	9.10	0.37	7.89	0.33	1.314 down	3.044 down	0.01	22.28	MFS permease
59315	7.45	0.06	8.24	0.08	6.20	0.21	1.723 up	2.386 down	0.00	105.73	PKS
59322	9.45	0.06	8.23	0.13	6.90	0.33	2.327 down	5.863 down	0.00	63.76	Zinc-binding oxidoreductase
59333	2.96	0.12	3.24	0.16	4.97	0.46	1.215 up	4.018 up	0.01	26.13	MFS permease
59351	7.22	0.24	5.80	0.07	5.29	0.20	2.679 down	3.796 down	0.00	68.48	1-aminocyclopropane-1-carboxylate synthase

59352	10.50	0.02	9.77	0.11	8.63	0.70	1.659 down	3.656 down	0.04	8.47	cytochrome P450
59353	9.10	0.12	9.39	0.01	7.82	0.36	1.228 up	2.423 down	0.01	25.14	Zn2Cys6 transcriptional regulator
59354	9.52	0.08	8.52	0.04	7.91	0.25	2.000 down	3.047 down	0.00	45.72	Zn2Cys6 transcriptional regulator
59364	13.68	0.06	9.67	0.31	9.01	0.22	16.146 down	25.526 down	0.00	306.55	Sexual differentiation process protein ISP4
59368	8.63	0.10	11.15	0.14	13.71	0.19	5.742 up	33.950 up	0.00	640.15	unknown protein. Duf341
59372	8.22	0.02	3.77	0.10	6.25	0.09	21.869 down	3.935 down	0.00	1530.25	unknown protein
59381	11.06	0.02	10.38	0.10	8.26	0.30	1.602 down	6.982 down	0.00	110.68	SAM-dependent methyltransferases
59382	11.81	0.09	11.10	0.22	8.60	0.30	1.643 down	9.290 down	0.00	126.32	unknown protein
59391	10.26	0.06	10.21	0.05	7.97	0.53	1.035 down	4.898 down	0.00	30.35	GH27 α-galactosidase
59396	5.91	0.03	5.58	0.03	7.20	0.61	1.260 down	2.444 up	0.03	9.64	unknown protein
59482	9.72	0.01	11.14	0.01	12.11	0.51	2.690 up	5.271 up	0.01	25.01	PKS
59491	8.79	0.11	8.05	0.06	6.17	0.12	1.664 down	6.146 down	0.00	452.69	unknown protein
59508	4.71	0.08	4.18	0.40	5.92	0.32	1.443 down	2.313 up	0.01	24.23	dipeptidyl peptidase 5
59515	9.24	0.02	10.57	0.06	11.04	0.25	2.504 up	3.481 up	0.00	56.31	MFS permease
59546	11.91	0.06	11.38	0.13	10.83	0.40	1.441 down	2.105 down	0.04	8.09	Zn2Cys6 transcriptional regulator
59582	3.67	0.11	4.21	0.12	5.32	0.35	1.451 up	3.142 up	0.01	26.41	unknown protein
59584	10.15	0.01	10.87	0.19	11.58	0.53	1.644 up	2.685 up	0.04	8.09	unknown protein
59597	4.17	0.03	4.47	0.16	5.69	0.09	1.231 up	2.859 up	0.00	180.21	unknown protein
59598	11.81	0.05	10.90	0.09	9.72	0.17	1.883 down	4.278 down	0.00	161.81	unknown protein
59609	12.56	0.13	12.57	0.01	11.43	0.33	1.007 up	2.188 down	0.01	18.74	unknown protein
59628	6.60	0.38	7.02	0.28	8.66	0.29	1.342 up	4.185 up	0.00	36.47	unknown protein
59642	11.29	0.09	11.01	0.15	9.51	0.39	1.211 down	3.427 down	0.01	27.73	α/ß hydrolase lipase/epoxide hydrolase
59649	8.47	0.04	8.65	0.01	6.51	0.27	1.129 up	3.892 down	0.00	95.16	Zinc-containing alcohol dehydrogenase
59665	10.19	0.06	10.14	0.10	8.87	0.32	1.031 down	2.486 down	0.01	25.76	unknown protein
59669	10.53	0.14	10.64	0.10	9.41	0.20	1.081 up	2.171 down	0.00	44.92	extracellular salicylate hydroxylase/monooxygenase. putative
59689	9.46	0.12	9.18	0.02	8.18	0.23	1.219 down	2.438 down	0.00	39.28	GH2 β-mannosidase
59690	3.08	0.31	2.91	0.24	4.86	0.13	1.119 down	3.442 up	0.00	83.95	unknown protein
59726	2.84	0.02	2.93	0.00	5.25	0.09	1.062 up	5.297 up	0.00	1077.63	DNA photolyase. N-terminal. class 1. FAD-binding
59740	9.36	0.13	8.98	0.01	7.55	0.18	1.298 down	3.502 down	0.00	114.38	transcriptional regulator. unknown
59746	9.24	0.04	9.03	0.01	7.55	0.17	1.158 down	3.233 down	0.00	148.83	oxaloacetase-like protein

59747	13.01	0.05	12.51	0.20	11.61	0.24	1.417 down	2.643 down	0.00	32.98	Rho GTPase-activating protein involved in signal transduction mechanisms
59751	11.25	0.03	10.68	0.19	10.18	0.26	1.475 down	2.093 down	0.01	16.20	Ribonucleases P/MRP protein subunit POP1 containing protein
59763	5.92	0.03	7.41	0.01	7.98	0.19	2.804 up	4.151 up	0.00	128.55	unknown protein
59770	10.49	0.12	9.88	0.12	8.59	0.16	1.525 down	3.754 down	0.00	131.69	aryl-alcohol dehydrogenases
59778	12.93	0.13	12.79	0.02	10.38	0.31	1.104 down	5.861 down	0.00	101.79	GPCR. related to A nidulans GprC
59791	2.72	0.05	2.91	0.02	5.03	0.42	1.143 up	4.978 up	0.00	46.91	GH18. chitinase CHI18-15
59796	12.70	0.05	12.21	0.02	11.57	0.09	1.407 down	2.193 down	0.00	162.81	MFS permease
59801	7.65	0.13	7.54	0.03	9.29	0.27	1.082 down	3.116 up	0.00	62.35	unknown protein
59827	8.93	0.18	9.60	0.04	11.11	0.11	1.592 up	4.546 up	0.00	256.44	unknown protein
59833	11.55	0.02	10.42	0.02	9.97	0.26	2.185 down	2.973 down	0.00	40.48	unknown protein
59843	9.38	0.07	9.54	0.22	8.05	0.31	1.113 up	2.512 down	0.00	29.40	AMP-dependent synthetase and ligase. acetoacetyl-CoA synthase-like
59876	4.91	0.56	4.48	0.20	7.32	1.08	1.341 down	5.318 up	0.03	9.07	xenobiotic compound monoounknown proteingenase. DszA family
59887	8.48	0.38	6.05	0.32	6.37	0.16	5.385 down	4.318 down	0.00	58.83	L-lactate dehydrogenase. putative
59936	9.18	0.01	9.86	0.24	10.54	0.22	1.599 up	2.570 up	0.00	30.80	unknown protein
59940	12.05	0.09	10.15	0.17	10.43	0.51	3.732 down	3.082 down	0.02	13.95	unknown protein
59952	8.96	0.08	10.04	0.25	6.58	0.28	2.117 up	5.176 down	0.00	148.78	Amino acid transporter PotE
60004	12.02	0.20	11.60	0.23	10.61	0.28	1.338 down	2.648 down	0.01	23.70	Zn2Cys6 transcriptional regulator
60028	3.54	0.28	3.37	0.13	5.22	0.24	1.122 down	3.193 up	0.00	58.73	unknown protein
60052	10.16	0.02	9.23	0.17	8.07	0.08	1.895 down	4.237 down	0.00	310.61	short chain dehydrogenase/reductase
60116	13.65	0.08	12.95	0.13	10.50	0.54	1.630 down	8.900 down	0.00	45.58	MRP-type ABC transporter
60144	3.73	0.37	3.78	0.30	6.18	0.91	1.036 up	5.498 up	0.02	11.01	Amino acid transporters
60182	3.68	0.65	2.68	0.17	4.94	0.21	1.995 down	2.394 up	0.00	30.63	unknown protein
60187	7.16	0.03	6.88	0.01	5.97	0.14	1.217 down	2.281 down	0.00	93.56	enoyl-CoA hydratase/isomerase
60194	10.74	0.02	10.07	0.27	9.62	0.23	1.588 down	2.163 down	0.01	17.54	phosphatidylinositol-specific phospholipase C (MPLC1)
60232	10.82	0.00	11.12	0.11	9.26	0.24	1.226 up	2.958 down	0.00	83.82	unknown protein
60270	12.04	0.01	11.59	0.02	10.90	0.12	1.362 down	2.202 down	0.00	114.22	unknown protein
60282	12.04	0.01	11.64	0.14	10.70	0.20	1.318 down	2.532 down	0.00	49.07	Zn2Cys6 transcriptional regulator
60328	9.38	0.09	9.00	0.32	7.77	0.18	1.299 down	3.043 down	0.00	50.46	unknown protein
60337	6.92	0.03	4.09	0.42	5.37	0.21	7.097 down	2.932 down	0.00	63.70	unknown protein
60352	9.49	0.05	7.87	0.24	7.24	0.51	3.078 down	4.788 down	0.01	20.45	NADH-dehydrogenase (ubiquinone)

60370	11.50	0.16	9.04	0.03	9.83	0.41	5.514 down	3.175 down	0.00	29.72	unknown protein
60378	7.71	0.17	9.13	0.18	6.60	0.39	2.670 up	2.164 down	0.00	42.54	AAA ATPase
60418	4.81	0.09	5.35	0.10	6.31	0.36	1.458 up	2.832 up	0.01	20.38	Aldehyde dehydrogenase
60419	4.91	0.18	4.90	0.13	6.42	0.20	1.005 down	2.848 up	0.00	65.69	acid phosphatase. putative
60422	10.55	0.13	9.08	0.14	9.02	0.32	2.764 down	2.876 down	0.01	24.60	unknown protein
60445	11.42	0.08	9.64	0.02	8.23	0.35	3.424 down	9.081 down	0.00	90.72	unknown protein
60456	11.12	0.06	11.32	0.12	10.03	0.13	1.149 up	2.137 down	0.00	105.59	unknown protein
60489	11.14	0.02	9.73	0.12	12.34	0.39	2.666 down	2.296 up	0.00	49.05	CE5 cutinase
60490	10.90	0.10	11.05	0.13	9.80	0.15	1.104 up	2.155 down	0.00	75.00	2-nitropropane dioxygenase
60557	3.26	0.11	3.42	0.16	4.72	0.12	1.123 up	2.760 up	0.00	123.01	unknown protein
60560	7.92	0.05	2.92	0.02	6.80	0.47	31.933 down	2.174 down	0.00	110.22	unknown protein
60565	9.98	0.10	10.00	0.02	7.72	0.35	1.012 up	4.780 down	0.00	69.41	unknown protein
60578	11.71	0.14	11.53	0.08	10.52	0.15	1.131 down	2.268 down	0.00	60.74	Zn2Cys6 transcriptional regulator
60616	12.42	0.13	10.18	0.06	10.42	0.48	4.708 down	3.985 down	0.01	22.81	unknown protein
60634	11.26	0.17	11.47	0.02	10.02	0.17	1.156 up	2.372 down	0.00	81.34	transcriptional regulator. unknown
60635	5.36	0.26	4.25	0.18	10.05	1.91	2.154 down	25.709 up	0.02	12.76	GH92 α-1.2-mannosidase
60638	5.49	0.09	4.42	0.58	8.04	0.16	2.099 down	5.875 up	0.00	118.79	unknown protein
60664	12.18	0.08	11.87	0.01	11.02	0.18	1.239 down	2.244 down	0.00	51.98	unknown protein with PH domain (putative phosphatidylinositol 4.5-bisphosphate pro
60743	12.33	0.10	12.96	0.12	13.40	0.22	1.540 up	2.098 up	0.01	23.27	Dolichyl-phosphate mannosyltransferase polypeptide 3
60758	11.64	0.27	7.97	0.01	9.29	0.14	12.719 down	5.079 down	0.00	265.91	SAM-dependent methyltransferase
60761	12.98	0.07	11.97	0.27	11.15	0.31	2.016 down	3.554 down	0.00	31.87	transcriptional regulator Grainyhead/CP2
60771	11.89	0.21	12.60	0.17	13.05	0.17	1.632 up	2.232 up	0.00	28.61	prefoldin chaperone
60810	13.51	0.16	9.84	0.06	11.90	0.33	12.659 down	3.051 down	0.00	94.27	unknown protein. GPR1/FUN34/yaaH protein. 6TMs
60825	3.24	0.07	3.37	0.05	5.26	0.49	1.099 up	4.054 up	0.01	25.86	Cytochrome P450. putative
60835	11.57	0.14	11.43	0.30	10.40	0.31	1.105 down	2.255 down	0.01	15.70	PutA delta-1-pyrroline-5-carboxylate dehydrogenase
60847	13.10	0.07	12.68	0.19	12.06	0.16	1.334 down	2.048 down	0.00	33.98	Mitochondrial F1F0-ATP synthase. subunit c/ATP9/proteolipid
60850	10.28	0.15	10.08	0.08	9.16	0.34	1.142 down	2.173 down	0.02	14.53	Mitochondrial initiation factor 2 (IF-2).
60890	9.90	0.01	10.19	0.12	11.60	0.27	1.224 up	3.235 up	0.00	52.23	L-galactose dehydrogenase (L-GalDH). putative
60897	11.62	0.12	11.60	0.07	10.25	0.11	1.013 down	2.589 down	0.00	158.40	Zn2Cys6 transcriptional regulator
60931	8.79	0.05	9.21	0.12	7.76	0.24	1.335 up	2.042 down	0.00	42.29	Zn2Cys6 transcriptional regulator

60945	12.91	0.23	10.66	0.13	13.97	0.60	4.781 down	2.081 up	0.00	32.27	MFS permease
60949	8.58	0.08	9.51	0.01	7.19	0.47	1.906 up	2.613 down	0.00	29.48	unknown protein
60951	12.14	0.05	11.79	0.03	10.95	0.11	1.275 down	2.289 down	0.00	147.24	unknown protein
60981	9.70	0.05	9.64	0.06	8.29	0.26	1.043 down	2.651 down	0.00	46.87	unknown protein
60987	11.60	0.12	11.73	0.01	9.87	0.47	1.100 up	3.317 down	0.01	23.91	MRP-type ABC transporter
61020	7.06	0.14	7.86	0.13	8.28	0.17	1.740 up	2.330 up	0.00	38.37	thioesterase superfamily protein
61032	11.58	0.15	11.14	0.07	10.40	0.13	1.363 down	2.274 down	0.00	64.23	unknown protein
61055	6.01	0.25	5.13	0.02	7.78	0.95	1.851 down	3.390 up	0.03	9.56	unique protein
61066	9.13	0.18	8.61	0.03	7.74	0.33	1.434 down	2.615 down	0.01	19.18	GMC oxidoreductase
61116	12.72	0.18	12.77	0.06	11.61	0.29	1.039 up	2.148 down	0.01	22.18	ferric reductase
61121	10.07	0.00	9.86	0.09	8.80	0.23	1.160 down	2.413 down	0.00	42.56	arylsulfatase. putative
61127	13.21	0.12	12.23	0.03	11.50	0.38	1.975 down	3.274 down	0.01	21.81	Serine carboxypeptidase
61134	7.27	0.04	7.78	0.12	8.41	0.34	1.429 up	2.205 up	0.02	12.31	unknown protein
61190	9.22	0.23	10.04	0.01	10.37	0.44	1.766 up	2.218 up	0.05	7.00	BolA domain-containing protein
61222	9.09	0.08	8.89	0.16	7.39	0.34	1.149 down	3.238 down	0.00	34.24	unknown protein
61278	8.56	0.03	8.33	0.11	7.47	0.32	1.175 down	2.137 down	0.01	15.29	MFS permease
61293	9.03	0.11	8.99	0.01	7.62	0.22	1.030 down	2.666 down	0.00	60.19	dipeptidyl peptidase 5
61383	11.92	0.06	11.04	0.01	10.48	0.23	1.849 down	2.716 down	0.00	41.73	unknown protein
61441	12.38	0.07	11.75	0.04	11.21	0.18	1.542 down	2.246 down	0.00	42.19	unknown protein
61476	9.73	0.17	9.23	0.11	7.79	0.25	1.409 down	3.821 down	0.00	66.49	Zn2Cys6 transcriptional regulator
61496	10.63	0.01	10.58	0.03	12.10	0.03	1.036 down	2.757 up	0.00	2729.14	MFS permease
61504	10.43	0.23	9.36	0.21	8.53	0.56	2.093 down	3.731 down	0.02	11.56	unknown protein
61526	9.20	0.04	7.93	0.00	7.44	0.15	2.413 down	3.380 down	0.00	145.06	unknown protein
61553	9.68	0.07	7.18	0.02	7.46	0.26	5.669 down	4.651 down	0.00	96.49	D-xylulose 5-phosphate/D-fructose 6-phosphate phosphoketolase
61605	5.92	0.21	9.20	0.11	7.21	0.28	9.729 up	2.443 up	0.00	94.03	Vacuolar sorting protein VPS1. dynamin. and related proteins
61618	4.46	0.13	4.71	0.15	6.16	0.29	1.187 up	3.255 up	0.00	42.56	unknown protein
61632	11.08	0.11	12.17	0.25	12.10	0.12	2.128 up	2.019 up	0.00	33.76	unknown protein
61642	13.96	0.09	13.01	0.10	12.28	0.21	1.927 down	3.207 down	0.00	66.28	unknown protein. secreted
61703	6.94	0.05	7.33	0.02	8.10	0.26	1.316 up	2.241 up	0.01	25.10	germinal center kinase. related to S. cerevisiae Kic1
61798	11.23	0.14	11.12	0.04	12.41	0.08	1.072 down	2.271 up	0.00	198.75	unknown protein. only in ascomycota

61830	4.61	0.55	4.11	0.37	6.16	0.28	1.405 down	2.945 up	0.01	24.99	SSCRP
61858	3.03	0.06	2.61	0.04	4.52	0.20	1.334 down	2.801 up	0.00	119.91	unknown protein
61907	11.25	0.04	11.25	0.13	10.21	0.23	1.002 up	2.049 down	0.00	29.21	unknown protein
61910	9.34	0.06	9.82	0.09	8.33	0.22	1.396 up	2.016 down	0.00	52.74	Aldehyde dehydrogenase
61939	11.89	0.02	11.83	0.08	13.48	0.23	1.044 down	3.007 up	0.00	77.56	unknown protein
61948	3.91	0.31	3.44	0.27	5.61	0.21	1.383 down	3.258 up	0.00	65.12	Protein disulfide isomerase 2
61987	9.69	0.10	9.53	0.16	8.65	0.21	1.122 down	2.063 down	0.01	27.90	unknown protein
61995	12.44	0.02	12.54	0.19	13.62	0.16	1.071 up	2.258 up	0.00	56.93	unknown protein
62034	10.78	0.05	10.03	0.05	8.60	0.43	1.683 down	4.546 down	0.00	32.03	unknown protein
62086	9.38	0.10	10.02	0.14	10.73	0.20	1.555 up	2.551 up	0.00	43.23	unknown protein
62114	8.20	0.32	7.22	0.30	6.32	0.27	1.978 down	3.675 down	0.00	28.93	unknown protein
62153	10.73	0.02	10.14	0.15	9.58	0.12	1.503 down	2.219 down	0.00	69.62	malate dehydrogenase
62171	8.05	0.16	7.86	0.22	10.58	0.38	1.142 down	5.770 up	0.00	68.47	MFS permease
62181	12.10	0.07	12.00	0.01	10.69	0.12	1.073 down	2.658 down	0.00	191.50	Protein kinase. Ca2+-dependent
62213	14.16	0.01	14.13	0.03	12.27	0.10	1.021 down	3.707 down	0.00	600.43	unknown protein with fasciclin domain
62255	2.78	0.22	2.90	0.31	4.59	0.25	1.092 up	3.526 up	0.00	46.53	lipase. lipocalin related
62285	11.93	0.01	11.65	0.15	10.32	0.14	1.215 down	3.054 down	0.00	136.68	unknown protein
62323	8.77	0.16	9.14	0.06	7.62	0.39	1.296 up	2.216 down	0.01	18.98	unknown protein
62333	3.87	0.01	3.88	0.40	5.81	0.23	1.003 up	3.830 up	0.00	57.41	unknown protein
62340	9.34	0.12	10.87	0.03	10.75	0.10	2.892 up	2.669 up	0.00	172.16	unknown protein
62380	13.37	0.03	9.16	0.06	11.97	0.21	18.482 down	2.628 down	0.00	327.92	MFS permease (galactose permease ?)
62401	12.21	0.11	12.02	0.04	10.91	0.20	1.136 down	2.453 down	0.00	53.69	guanine nucleotide exchange factor synembryn. putative
62424	11.09	0.24	10.87	0.09	9.39	0.24	1.165 down	3.248 down	0.00	51.37	UDP-glucose:sterol b-glucosyltransferase
62447	12.86	0.01	12.11	0.07	10.56	0.15	1.686 down	4.938 down	0.00	276.92	unknown protein
62462	11.76	0.01	11.20	0.08	13.04	0.28	1.475 down	2.438 up	0.00	53.68	PTH11 GPCR
62477	8.96	0.04	10.13	0.17	10.32	0.27	2.257 up	2.568 up	0.01	26.63	Vacuolar protein sorting-associated protein Vps28
62480	9.47	0.08	10.22	0.32	10.50	0.16	1.686 up	2.049 up	0.01	19.05	mitochondrial tricarboxylate transporter (Ctp). putative
62484	10.98	0.03	9.45	0.01	9.00	0.28	2.877 down	3.937 down	0.00	55.93	unknown protein
62488	10.02	0.01	11.62	0.09	11.50	0.18	3.022 up	2.780 up	0.00	79.12	MFS permease
62502	12.31	0.05	12.66	0.08	10.98	0.14	1.270 up	2.522 down	0.00	180.09	MFS permease

62556	10.86	0.07	9.78	0.07	9.20	0.34	2.118 down	3.153 down	0.01	25.49	unknown protein
62611	13.56	0.35	12.64	0.28	10.43	0.17	1.888 down	8.764 down	0.00	131.15	MRP-type ABC transporter
62651	12.13	0.20	11.73	0.22	9.71	0.27	1.323 down	5.377 down	0.00	81.82	long-chain fatty acid transporter.
62663	7.26	0.13	7.55	0.03	8.61	0.30	1.221 up	2.548 up	0.01	25.32	unknown protein
62693	12.91	0.13	12.96	0.04	8.73	0.29	1.031 up	18.111 down	0.00	326.74	ABC-transporter Ste6p
62704	9.85	0.03	9.60	0.00	8.73	0.07	1.191 down	2.177 down	0.00	382.90	GH18. chitinase CHI18-3
62711	13.76	0.04	13.44	0.07	12.72	0.13	1.252 down	2.050 down	0.00	67.51	amino acid transporter
62716	5.18	0.15	6.46	0.05	14.19	0.12	2.434 up	515.554 up	0.00	5318.91	Ctr copper transporter family protein
62805	10.55	0.11	11.06	0.05	9.09	0.48	1.427 up	2.758 down	0.01	21.90	C2H2 transcriptional regulator
62836	12.48	0.02	12.02	0.13	11.01	0.24	1.377 down	2.777 down	0.00	44.20	unknown protein
62872	13.50	0.04	14.52	0.00	10.24	0.17	2.024 up	9.608 down	0.00	801.65	unknown protein GPR1/FUN34/yaaH-like
62912	10.88	0.15	11.74	0.19	12.25	0.42	1.810 up	2.580 up	0.02	10.77	unknown protein. 1TM
62977	8.80	0.15	7.33	0.05	7.13	0.44	2.778 down	3.175 down	0.01	15.77	nucleoside diphosphate sugar epimerase. secreted
63007	11.14	0.03	10.48	0.02	9.22	0.27	1.582 down	3.793 down	0.00	63.31	meiotic chromosome segregation protein. putative
63152	11.92	0.01	11.75	0.02	10.65	0.16	1.122 down	2.397 down	0.00	89.81	Cut9 interacting protein Scn1. putative
63163	10.24	0.18	10.27	0.23	9.17	0.22	1.015 up	2.105 down	0.01	24.63	unknown protein
63202	11.99	0.00	11.71	0.06	9.79	0.18	1.213 down	4.578 down	0.00	213.52	alpha/beta hydrolase.
63204	13.22	0.12	12.94	0.11	12.10	0.13	1.214 down	2.179 down	0.00	68.76	flavodoxin domain containing protein
63379	9.28	0.09	10.30	0.24	10.57	0.29	2.029 up	2.439 up	0.01	17.80	RNA polymerase II transcriptional coactivator. putative
63395	11.49	0.10	12.00	0.11	10.26	0.24	1.427 up	2.347 down	0.00	60.27	Glycerophosphoryl diester phosphodiesterase
63400	12.12	0.00	11.74	0.15	11.04	0.42	1.303 down	2.106 down	0.04	7.62	mitochondrial exoribonuclease Cyt-4
63503	9.36	0.01	9.85	0.10	10.52	0.10	1.398 up	2.238 up	0.00	120.72	rrbosomal protein S2 and tRNA/rRNA methyltransferase.
63526	7.20	0.11	8.40	0.21	9.86	0.76	2.301 up	6.318 up	0.02	13.88	SSCRP
63653	8.36	0.13	11.65	0.17	10.78	0.54	9.788 up	5.352 up	0.00	32.50	ankyrin
63687	10.12	0.02	8.04	0.18	9.01	0.57	4.207 down	2.154 down	0.03	10.58	ankyrin
63692	8.79	0.06	8.36	0.14	7.34	0.12	1.343 down	2.729 down	0.00	114.60	dipeptidyl peptidase 5
63702	11.90	0.08	11.48	0.02	10.66	0.10	1.342 down	2.367 down	0.00	146.37	unknown protein
63710	3.49	0.48	3.17	0.17	4.57	0.08	1.246 down	2.107 up	0.01	28.05	MMR; HR regulation
63727	11.71	0.11	11.71	0.04	10.56	0.06	1.004 down	2.219 down	0.00	241.90	Acyl-CoA dehydrogenase. central region
63756	9.82	0.01	8.34	0.18	8.47	0.23	2.781 down	2.539 down	0.00	38.77	helicase. DEAD-box superfamily

63815	8.24	0.36	8.31	0.10	10.56	0.27	1.051 up	4.989 up	0.00	71.74	Membrane coat complex Retromer. subunit Vps26
63837	6.97	0.22	6.87	0.12	5.60	0.19	1.072 down	2.580 down	0.00	51.05	unknown protein
63868	12.27	0.17	10.83	0.29	10.74	0.23	2.728 down	2.899 down	0.00	30.82	unknown protein
63881	8.65	0.11	9.81	0.28	9.80	0.26	2.236 up	2.218 up	0.01	17.07	unknown protein
63899	7.43	0.28	8.07	0.10	9.65	0.43	1.554 up	4.639 up	0.00	29.96	unknown protein
63914	12.17	0.14	11.35	0.04	10.52	0.24	1.761 down	3.150 down	0.00	50.23	integral membrane protein. putative
63919	9.02	0.04	10.16	0.02	10.47	0.17	2.195 up	2.727 up	0.00	81.27	Cys/Met metabolism PLP-dependent enzyme
63966	4.65	0.19	4.61	0.18	6.45	0.10	1.027 down	3.494 up	0.00	172.27	high-affinity glucose transporter
63978	11.83	0.04	11.65	0.07	10.51	0.15	1.133 down	2.490 down	0.00	96.69	Zn2Cys6 transcriptional regulator
64018	13.00	0.01	13.15	0.10	11.32	0.32	1.107 up	3.214 down	0.00	48.23	GPCR. mating type pheromone G-protein coupled receptor
64029	11.06	0.14	10.75	0.02	9.77	0.22	1.233 down	2.447 down	0.00	40.91	folylpolyglutamate synthase . putative
64049	6.14	0.38	6.05	0.29	9.12	0.88	1.064 down	7.850 up	0.01	17.94	unknown protein
64066	11.95	0.08	12.21	0.02	10.50	0.15	1.196 up	2.742 down	0.00	171.56	acyltransferase 3
64172	8.33	0.03	9.00	0.11	9.77	0.32	1.590 up	2.704 up	0.01	21.85	Glutathione-dependent formaldehyde-activating.GFA
64181	6.49	0.22	7.41	0.03	7.77	0.48	1.885 up	2.427 up	0.04	7.51	SSCRP
64288	2.96	0.43	2.71	0.24	4.22	0.16	1.188 down	2.399 up	0.00	30.72	unknown protein
64295	13.49	0.01	13.17	0.06	12.03	0.09	1.242 down	2.735 down	0.00	282.73	nitrogen permease regulator Npr2. putative
64312	10.82	0.11	9.86	0.19	8.77	0.43	1.953 down	4.165 down	0.01	23.90	unknown protein. only present in fungi
64318	10.80	0.01	11.17	0.15	11.86	0.26	1.295 up	2.086 up	0.01	19.14	unknown protein
64370	11.10	0.00	9.92	0.15	8.06	0.24	2.259 down	8.211 down	0.00	165.00	calpain-like protease
64448	12.64	0.05	13.42	0.08	14.15	0.08	1.720 up	2.858 up	0.00	281.90	unknown protein
64469	12.17	0.15	11.25	0.24	11.06	0.20	1.902 down	2.170 down	0.01	22.17	Hydrolases of alpha/beta hydrolase superfamily
64545	11.63	0.04	10.44	0.03	9.74	0.15	2.286 down	3.702 down	0.00	168.21	MFS permease
64620	11.03	0.01	10.56	0.01	9.87	0.26	1.383 down	2.236 down	0.01	24.39	Alpha/beta hydrolase
64667	13.55	0.07	11.55	0.19	11.83	0.47	4.014 down	3.283 down	0.01	18.11	unknown protein
64672	9.61	0.22	9.23	0.11	7.29	0.11	1.298 down	4.986 down	0.00	235.56	Helix-turn-helix. AraC type
64676	8.24	0.07	10.19	0.25	9.52	0.26	3.856 up	2.420 up	0.00	36.17	unknown protein
64710	10.98	0.12	10.52	0.18	8.94	0.16	1.372 down	4.096 down	0.00	136.61	AAA+-type ATPase
64719	10.24	0.05	9.40	0.03	9.06	0.17	1.795 down	2.267 down	0.00	53.49	Subtilisin like protease (SUB3)
64827	11.92	0.06	11.54	0.10	10.23	0.27	1.302 down	3.227 down	0.00	49.77	GH36 raffinose synthase domain protein

64834	11.00	0.04	10.78	0.08	9.57	0.24	1.158 down	2.680 down	0.00	49.76	unknown protein
64874	13.37	0.10	13.72	0.17	12.13	0.24	1.271 up	2.365 down	0.00	48.77	MFS toxin efflux pump
64882	10.29	0.11	8.34	0.20	8.51	0.19	3.852 down	3.417 down	0.00	79.16	MFS permease
64916	11.05	0.06	11.80	0.04	9.60	0.77	1.689 up	2.720 down	0.03	10.02	unknown protein
64919	4.55	0.27	5.05	0.22	6.26	0.26	1.410 up	3.266 up	0.00	33.76	MFS permease
64925	11.35	0.10	12.38	0.07	12.56	0.17	2.051 up	2.320 up	0.00	50.56	GT32 a-glycosyltransferase
64937	7.31	0.09	7.37	0.03	8.69	0.53	1.045 up	2.616 up	0.02	10.81	unknown protein
64951	3.80	0.16	3.60	0.06	5.64	0.39	1.147 down	3.583 up	0.00	38.84	unknown protein
64956	3.21	0.29	3.66	0.18	4.89	0.21	1.370 up	3.208 up	0.00	45.55	unknown protein
64959	11.30	0.18	7.57	0.01	10.02	0.18	13.328 down	2.437 down	0.00	274.98	phosphatidyl synthase
64971	9.89	0.04	8.87	0.10	8.54	0.46	2.033 down	2.559 down	0.03	9.51	Amino acid permeases
64996	3.95	0.08	6.69	0.10	6.46	0.79	6.698 up	5.718 up	0.02	13.53	nitrilase
65021	5.65	0.13	6.06	0.20	7.25	0.41	1.331 up	3.039 up	0.01	18.30	short chain dehydrogenase/reductase
65029	12.94	0.00	12.60	0.05	11.81	0.35	1.263 down	2.182 down	0.02	13.39	2OG-Fe(II) oxygenase superfamily protein
65037	5.95	0.01	5.27	0.04	7.18	0.09	1.602 down	2.345 up	0.00	490.32	pyrophosphatase
65040	3.38	0.10	3.25	0.27	4.77	0.14	1.088 down	2.634 up	0.00	76.58	cytochrome P450. putative
65067	4.31	0.19	3.41	0.19	6.07	0.24	1.860 down	3.403 up	0.00	105.24	short chain dehydrogenase/reductase
65095	5.17	0.12	5.36	0.11	6.42	0.12	1.135 up	2.378 up	0.00	97.83	unknown protein
65096	6.81	0.24	7.26	0.14	8.09	0.42	1.360 up	2.422 up	0.03	10.00	3'-tRNA processing endoribonuclease
65098	3.78	0.06	4.25	0.01	5.88	0.11	1.379 up	4.278 up	0.00	482.28	FAD-binding domain and SignalP-predicted secretion signal. Distantly related to tre36
65102	3.65	0.38	3.66	0.36	5.02	0.18	1.007 up	2.577 up	0.01	24.56	unknown protein
65106	4.46	0.08	4.58	0.32	9.24	1.03	1.083 up	27.491 up	0.00	33.64	CN_hydrolase
65107	11.66	0.07	11.22	0.08	10.41	0.10	1.357 down	2.374 down	0.00	142.19	cytochrome P450
65116	4.04	0.56	4.29	0.02	5.36	0.25	1.187 up	2.497 up	0.02	14.62	PKS
65117	6.15	0.40	4.67	0.43	4.87	0.16	2.782 down	2.425 down	0.01	16.37	ankyrin
65133	7.83	0.10	8.29	0.13	6.33	0.54	1.370 up	2.840 down	0.01	17.26	Zn2Cys6 transcriptional regulator
65141	6.54	0.15	6.57	0.13	7.80	0.31	1.022 up	2.394 up	0.01	23.45	cytochrome P450 monooxygenase
65142	9.07	0.05	9.92	0.05	10.32	0.26	1.800 up	2.379 up	0.01	25.89	aldehyde dehydrogenase ALDH
65153	9.45	0.10	9.48	0.13	8.43	0.33	1.019 up	2.030 down	0.01	15.53	MFS permease
65156	6.32	0.19	7.24	0.10	7.74	0.31	1.887 up	2.672 up	0.01	19.81	S1/P1 nuclease

65162	10.94	0.19	12.83	0.03	13.37	0.14	3.710 up	5.381 up	0.00	210.52	GH18 endo-N-acetyl-ß-D-glucosaminidase Endo T
65164	12.52	0.16	11.75	0.02	11.22	0.19	1.711 down	2.468 down	0.00	43.30	unknown protein
65172	5.02	0.17	5.05	0.04	6.17	0.21	1.018 up	2.221 up	0.00	38.75	PKS
65223	8.89	0.14	9.58	0.04	7.79	0.13	1.609 up	2.156 down	0.00	160.57	unknown protein
65229	9.68	0.08	11.34	0.01	10.95	0.12	3.163 up	2.411 up	0.00	147.35	unknown protein
65232	9.03	0.19	9.21	0.08	7.41	0.39	1.132 up	3.068 down	0.00	29.32	short chain dehydrogenase/reductase
65286	9.49	0.02	9.79	0.16	8.15	0.13	1.227 up	2.536 down	0.00	147.30	unknown protein
65315	12.82	0.06	8.79	0.13	9.29	0.77	16.360 down	11.572 down	0.00	29.31	bZIP transcription factor
65324	8.73	0.00	9.42	0.19	11.30	0.23	1.609 up	5.921 up	0.00	135.59	metacaspase CasA
65333	12.54	0.01	11.26	0.24	10.24	0.25	2.438 down	4.942 down	0.00	72.53	GH15 alpha-glycosidase (Glucoamylase and related glycosyl hydrolases)
65380	10.51	0.12	11.99	0.04	11.93	0.26	2.788 up	2.669 up	0.00	36.52	GH47 α-1.2-mannosidase
65494	9.71	0.06	11.70	0.13	11.08	0.58	3.957 up	2.590 up	0.03	10.24	metallopeptidase
65499	5.29	0.43	5.16	0.09	6.80	0.20	1.096 down	2.838 up	0.00	39.00	Tyrosine specific protein phosphatase and dual specificity protein phosphatase
65508	13.05	0.10	12.90	0.03	11.76	0.27	1.109 down	2.452 down	0.00	31.46	fructose-bisphosphatase
65522	11.90	0.17	8.57	0.05	9.84	0.55	10.051 down	4.155 down	0.00	29.92	unknown protein
65547	8.18	0.01	8.48	0.02	6.52	0.10	1.235 up	3.156 down	0.00	559.48	mandelate racemase/muconate lactonase-like protein
65572	11.88	0.26	11.70	0.00	10.48	0.35	1.131 down	2.638 down	0.01	19.98	unknown protein
65603	5.97	0.05	6.76	0.03	7.41	0.10	1.730 up	2.704 up	0.00	218.43	unknown protein
65607	12.60	0.06	12.50	0.17	11.41	0.17	1.071 down	2.279 down	0.00	54.57	RgsC. regulator of G-protein signaling
65640	7.63	0.06	8.39	0.00	9.44	0.17	1.692 up	3.499 up	0.00	122.56	unknown protein
65711	12.41	0.09	13.00	0.02	13.87	0.17	1.504 up	2.757 up	0.00	77.00	SAM-dependent methyltransferase
65718	14.38	0.00	13.13	0.14	13.04	0.10	2.383 down	2.523 down	0.00	127.25	unknown protein
65736	5.12	0.22	6.20	0.12	7.28	0.27	2.122 up	4.485 up	0.00	57.97	catabolic 3-dehydroquinase
65739	10.32	0.10	9.69	0.05	8.08	0.36	1.548 down	4.721 down	0.00	49.11	unknown protein
65746	11.35	0.07	11.13	0.11	9.96	0.23	1.162 down	2.609 down	0.00	47.32	Zn2Cys6 transcriptional regulator
65768	12.51	0.05	12.86	0.20	11.48	0.35	1.275 up	2.053 down	0.01	18.56	unknown protein
65771	3.32	0.15	3.49	0.21	6.02	0.25	1.125 up	6.530 up	0.00	133.71	NACHT domain WD40 repeat-containing protein. related to HET
65817	9.57	0.03	7.01	0.13	7.80	0.21	5.911 down	3.410 down	0.00	115.75	GT α-1.3-mannosyltransferase CMT1
65819	7.73	0.13	7.17	0.01	6.41	0.37	1.477 down	2.505 down	0.02	14.34	Molecular chaperone Hsp70 family
65821	11.91	0.22	11.69	0.07	10.83	0.10	1.160 down	2.113 down	0.00	57.75	vacuolar targeting protein Atg18

65873	10.68	0.14	10.13	0.08	9.49	0.03	1.464 down	2.278 down	0.00	166.62	protein kinase A. catalytic subunit
65882	10.26	0.13	9.82	0.03	8.99	0.14	1.350 down	2.411 down	0.00	77.58	dihydrodipicolinate synthase . putative
65883	10.29	0.13	8.07	0.26	8.24	0.39	4.669 down	4.131 down	0.00	30.96	D-isomer-specific 2-hydroxy acid dehydrogenase
65891	10.76	0.08	10.10	0.05	9.38	0.52	1.574 down	2.603 down	0.04	7.94	PKS
65921	7.81	0.02	7.80	0.02	9.58	0.47	1.005 down	3.414 up	0.01	23.91	Acetyl/propionyl-CoA carboxylase alpha subunit
65925	6.90	0.08	7.27	0.12	8.09	0.20	1.289 up	2.282 up	0.00	39.42	cytidine and deoxycytidylate deaminase zinc-binding region
65933	6.13	0.30	7.21	0.06	7.25	0.27	2.112 up	2.174 up	0.01	14.69	Epl1/Sm1
65949	8.32	0.13	11.21	0.09	9.47	0.36	7.410 up	2.214 up	0.00	50.95	unknown protein
65950	8.29	0.04	8.54	0.06	6.74	0.14	1.188 up	2.931 down	0.00	221.30	unknown protein
65957	9.80	0.06	9.77	0.00	8.74	0.12	1.022 down	2.086 down	0.00	111.23	unknown protein
65975	9.70	0.08	9.01	0.06	8.22	0.46	1.609 down	2.796 down	0.02	11.72	unknown protein
65986	10.77	0.12	10.22	0.16	9.33	0.42	1.463 down	2.718 down	0.02	13.15	GH27 α-galactosidase
65997	9.57	0.20	10.38	0.00	11.25	0.18	1.751 up	3.188 up	0.00	68.40	Cyclophilin type peptidyl-prolyl cis-trans isomerase
66016	12.23	0.01	10.16	0.14	10.62	0.12	4.207 down	3.062 down	0.00	202.79	dienelactone hydrolase
66023	9.71	0.09	9.67	0.03	8.31	0.22	1.028 down	2.627 down	0.00	61.18	unknown protein
66034	7.76	0.27	7.61	0.06	5.19	0.58	1.104 down	5.924 down	0.00	28.44	unknown protein
66077	3.39	0.09	3.19	0.11	5.34	0.15	1.149 down	3.847 up	0.00	238.23	ooc1-related protein
66092	11.38	0.02	8.31	0.38	9.94	0.22	8.365 down	2.706 down	0.00	81.94	unique protein
66103	12.17	0.14	13.09	0.05	13.45	0.10	1.891 up	2.429 up	0.00	99.41	WbbJ Acetyltransferase (isoleucine patch superfamily)
66210	8.39	0.02	8.94	0.04	9.54	0.18	1.461 up	2.221 up	0.00	47.52	unknown protein
66228	5.97	0.03	6.21	0.05	7.75	0.55	1.186 up	3.451 up	0.01	15.54	unknown protein
66268	13.01	0.14	12.89	0.11	11.79	0.14	1.083 down	2.323 down	0.00	75.40	unknown protein
66345	11.79	0.03	11.50	0.07	14.57	0.13	1.222 down	6.910 up	0.00	773.21	Mn superoxide dismutase
66370	4.39	0.03	4.25	0.06	6.58	0.51	1.105 down	4.544 up	0.00	32.25	unknown protein
66394	11.29	0.08	11.42	0.02	9.71	0.19	1.099 up	2.982 down	0.00	120.69	DNA polymerase family X member. Most closely related to DNA polymerase mu. an er[illegible] activity.
66510	2.93	0.31	2.80	0.13	4.38	0.16	1.090 down	2.723 up	0.00	60.85	short chain dehydrognease/reductase
66521	11.51	0.19	12.70	0.07	10.42	0.23	2.283 up	2.125 down	0.00	88.69	unknown protein
66534	9.56	0.00	5.71	0.08	7.80	1.11	14.354 down	3.374 down	0.03	9.99	Cytochrome P450 CYP2 subfamily
66551	7.18	0.13	5.93	0.14	5.89	0.13	2.368 down	2.445 down	0.00	71.41	AAA ATPase

66563	10.78	0.03	11.87	0.00	12.27	0.18	2.132 up	2.812 up	0.00	73.89	unknown protein
66598	10.69	0.05	9.09	0.02	9.10	0.37	3.037 down	3.008 down	0.01	23.40	NADPH:quinone oxidoreductase
66606	9.73	0.08	10.06	0.03	8.49	0.31	1.256 up	2.361 down	0.00	33.93	Zn2Cys6 transcriptional regulator
66614	9.91	0.01	9.69	0.21	7.95	0.31	1.167 down	3.892 down	0.00	50.23	alpha/beta hydrolase
66657	9.19	0.28	8.61	0.15	7.33	0.47	1.494 down	3.626 down	0.01	16.97	MFS permease
66662	11.31	0.10	11.11	0.08	9.71	0.27	1.148 down	3.023 down	0.00	46.98	Mandelate racemase/muconate lactonizing protein
66726	8.99	0.02	5.58	0.08	7.84	1.11	10.629 down	2.210 down	0.04	8.31	FAD binding protein
66751	5.11	0.00	4.77	0.04	6.27	0.63	1.261 down	2.246 up	0.04	7.79	unknown protein
66753	6.41	0.13	6.50	0.05	8.15	0.44	1.065 up	3.341 up	0.01	23.42	unknown protein
66758	7.13	0.00	6.56	0.01	8.21	0.55	1.479 down	2.110 up	0.02	10.88	Ankyrin
66766	4.42	0.20	3.60	0.10	8.68	0.06	1.758 down	19.235 up	0.00	1788.24	Carbon-nitrogen hydrolase
66776	6.90	0.31	6.42	0.19	8.17	0.63	1.399 down	2.410 up	0.03	9.01	unknown protein
66786	12.32	0.00	10.09	0.12	10.92	0.37	4.715 down	2.649 down	0.00	30.29	unknown protein
66788	9.09	0.10	11.05	0.08	11.68	0.69	3.890 up	5.984 up	0.01	15.41	unknown protein
66804	11.01	0.08	10.55	0.13	9.65	0.29	1.376 down	2.571 down	0.01	26.01	GH69: candidate a-glycosyltransferase
66819	13.16	0.13	12.89	0.11	12.05	0.29	1.208 down	2.170 down	0.01	17.56	Amino acid transporter LysP
66827	9.66	0.03	9.34	0.04	7.57	0.34	1.251 down	4.262 down	0.00	55.20	Zinc-containing alcohol dehydrogenase
66828	13.58	0.20	14.26	0.14	12.33	0.22	1.603 up	2.380 down	0.00	69.86	Zn2Cys6 transcriptional regulator
66859	3.22	0.10	3.49	0.36	5.47	0.24	1.205 up	4.750 up	0.00	70.38	serine peptidase S28
66865	9.52	0.02	7.81	0.04	7.50	0.25	3.263 down	4.034 down	0.00	71.77	unknown protein
66877	9.95	0.05	9.82	0.00	8.95	0.12	1.094 down	2.003 down	0.00	104.02	unknown protein
66888	11.01	0.03	7.44	0.09	8.61	0.33	11.913 down	5.286 down	0.00	101.58	GT α-1.3-mannosyltransferase
66895	9.51	0.06	10.49	0.28	11.79	0.17	1.971 up	4.834 up	0.00	108.65	3' exoribonuclease
66937	12.09	0.04	11.31	0.02	10.06	0.23	1.717 down	4.094 down	0.00	95.00	FAD binding domain-containing protein
66950	9.60	0.18	9.63	0.07	8.28	0.39	1.020 up	2.504 down	0.01	17.85	serine/threonine protein kinase.
66999	5.65	0.04	5.09	0.19	8.42	1.00	1.480 down	6.807 up	0.01	15.61	AMP-dependent synthetase and ligase. putative
67024	10.50	0.16	10.01	0.03	12.38	0.24	1.411 down	3.681 up	0.00	112.75	unknown protein
67035	13.88	0.12	13.30	0.10	11.64	0.09	1.492 down	4.701 down	0.00	407.39	unknown protein
67053	12.27	0.05	12.09	0.05	11.17	0.25	1.132 down	2.145 down	0.01	27.72	Ubiquitin carboxyl-terminal hydrolase
67107	9.06	0.07	10.41	0.07	10.76	0.19	2.535 up	3.231 up	0.00	80.76	MutS-related protein involved in mismatch repair

67109	6.13	0.07	5.76	0.06	7.93	0.72	1.288 down	3.491 up	0.02	12.73	unknown protein
67133	11.34	0.20	10.12	0.26	9.95	0.40	2.323 down	2.620 down	0.02	11.39	unknown protein
67272	12.44	0.05	11.86	0.14	11.11	0.04	1.492 down	2.516 down	0.00	233.49	CRO1. required for syncytial to cellular transision. involved in sexual development
67275	11.28	0.10	12.61	0.19	12.44	0.16	2.518 up	2.241 up	0.00	46.36	RAS1
67286	10.78	0.11	10.42	0.11	9.04	0.22	1.279 down	3.345 down	0.00	72.79	unknown protein
67290	5.52	0.19	5.82	0.01	7.35	0.10	1.231 up	3.539 up	0.00	207.56	unknown protein
67418	8.16	0.04	8.64	0.23	9.75	0.14	1.399 up	3.015 up	0.00	83.32	C2H2 transcription factor
67448	10.50	0.08	10.45	0.01	8.80	0.08	1.030 down	3.245 down	0.00	529.60	AGA_beta. Aspartylglucosaminidase family
67469	13.08	0.04	11.41	0.12	11.48	0.10	3.172 down	3.022 down	0.00	221.51	MFS permease (maltose permease)
67494	14.04	0.10	13.38	0.23	11.73	0.28	1.575 down	4.957 down	0.00	70.17	Golgi GDP-mannose transporter
67538	11.44	0.03	6.73	0.08	8.64	0.24	26.140 down	6.954 down	0.00	303.94	Catalase
67579	9.78	0.27	8.67	0.02	11.32	0.96	2.166 down	2.901 up	0.03	8.79	phospholipase A2
67600	2.17	0.16	2.32	0.14	4.02	0.04	1.108 up	3.619 up	0.00	323.80	Fungal chitin synthase
67605	8.90	0.02	9.11	0.04	7.09	0.15	1.152 up	3.525 down	0.00	274.34	unknown protein
67607	4.49	0.06	4.71	0.22	6.80	0.65	1.160 up	4.956 up	0.01	18.67	unknown protein
67616	12.26	0.03	11.55	0.19	11.15	0.16	1.632 down	2.155 down	0.00	36.56	unknown protein
67627	3.25	0.03	3.30	0.06	4.66	0.09	1.034 up	2.664 up	0.00	358.06	short chain dehydrognease/reductase
67639	13.74	0.06	13.69	0.01	12.57	0.13	1.038 down	2.249 down	0.00	118.16	IlvB. Thiamine pyrophosphate-requiring enzymes
67678	4.34	0.06	4.61	0.02	6.49	0.59	1.211 up	4.457 up	0.01	19.30	chitin deacetylase
67692	7.05	0.49	5.84	0.22	5.93	0.15	2.318 down	2.175 down	0.02	13.90	MFS permease
67698	4.30	0.13	4.83	0.29	5.55	0.19	1.450 up	2.387 up	0.01	26.61	nucleotide sugar dehydrogenase (UDP-Glc?)
67699	10.94	0.14	7.00	0.09	9.04	0.37	15.411 down	3.753 down	0.00	88.88	FAD/NAD-oxidoreductase. only in Hypocreaceae
67707	4.05	0.07	4.47	0.37	5.58	0.12	1.340 up	2.879 up	0.00	48.81	unknown protein with TLC domain
67717	11.97	0.07	11.28	0.16	10.15	0.37	1.614 down	3.520 down	0.01	27.52	unknown protein. ß-lactamase HMMPfam
67761	9.80	0.19	11.09	0.19	11.77	0.26	2.447 up	3.908 up	0.00	45.72	calpain-like protease
67866	6.71	0.24	4.75	0.10	5.42	0.29	3.890 down	2.442 down	0.00	31.59	unknown protein
67958	3.71	0.22	3.50	0.33	4.90	0.19	1.153 down	2.276 up	0.00	31.06	unknown protein. only present in Magnaporthe. Chaetomium and Gibberella
67964	13.24	0.10	12.12	0.01	11.94	0.12	2.182 down	2.462 down	0.00	115.86	Cytochrome P450 CYP2 subfamily
68019	9.98	0.24	8.75	0.04	7.87	0.10	2.337 down	4.319 down	0.00	164.18	beta-lactamase superfamily
68028	8.52	0.05	8.46	0.09	7.29	0.27	1.039 down	2.340 down	0.00	32.22	unknown protein

68036	13.49	0.04	12.84	0.11	12.27	0.23	1.572 down	2.329 down	0.00	30.28	Cystathionine beta-lyases/cystathionine gamma-synthases
68068	11.60	0.10	11.38	0.00	10.53	0.27	1.157 down	2.086 down	0.01	20.00	unknown protein
68086	11.08	0.08	11.15	0.01	9.31	0.27	1.050 up	3.395 down	0.00	73.54	unknown protein
68131	8.23	0.09	7.75	0.18	6.87	0.33	1.392 down	2.566 down	0.01	18.66	heterocompatibility domain protein
68161	8.39	0.05	7.20	0.11	6.22	0.14	2.279 down	4.504 down	0.00	230.11	Arylacetamide deacetylase
68169	8.21	0.14	6.43	0.03	6.86	0.32	3.450 down	2.553 down	0.01	27.93	Calcium transporter
68204	8.67	0.13	8.95	0.01	6.92	0.25	1.209 up	3.375 down	0.00	89.00	NRPS
68279	10.54	0.02	8.52	0.10	8.02	0.26	4.046 down	5.749 down	0.00	101.46	Short-chain dehydrogenase/reductase
68336	4.58	0.03	4.81	0.18	5.90	0.10	1.171 up	2.489 up	0.00	114.61	short chain dehydrogenase/reductase
68348	8.57	0.03	10.86	0.20	12.10	0.13	4.893 up	11.521 up	0.00	466.73	SAM-dependent methyltransferase
68371	5.35	0.33	5.16	0.11	7.60	0.30	1.138 down	4.760 up	0.00	71.66	protein kinase. unusual
68401	3.11	0.06	3.37	0.01	4.86	0.10	1.199 up	3.353 up	0.00	396.73	unknown protein
68455	10.90	0.08	10.71	0.02	9.64	0.11	1.145 down	2.398 down	0.00	145.45	Zn2Cys6 transcriptional regulator
68466	4.51	0.05	4.42	0.05	5.76	0.20	1.060 down	2.389 up	0.00	66.70	glycerone kinase
68508	9.81	0.16	9.83	0.09	8.40	0.21	1.011 up	2.661 down	0.00	60.28	unknown protein
68574	9.77	0.27	6.82	0.02	8.03	0.37	7.717 down	3.343 down	0.00	45.98	unknown protein
68590	10.65	0.02	9.87	0.06	9.28	0.22	1.715 down	2.584 down	0.00	41.55	Alcohol dehydrogenase. class V
68608	14.73	0.13	14.40	0.00	13.41	0.20	1.260 down	2.507 down	0.00	50.55	Thiazole biosynthetic enzyme (Stress-inducible protein sti35)
68636	10.94	0.25	11.38	0.17	12.13	0.22	1.349 up	2.275 up	0.01	22.75	ribosomal protein S11
68640	7.38	0.20	7.79	0.10	8.43	0.40	1.331 up	2.079 up	0.04	7.47	unknown protein
68705	12.22	0.04	12.33	0.05	7.10	0.64	1.079 up	34.693 down	0.00	107.87	cytochrome P450 monooxygenase
68755	12.56	0.08	10.20	0.13	9.23	0.25	5.158 down	10.057 down	0.00	174.06	short chain dehydrogenase/reductase
68813	13.41	0.05	11.72	0.01	11.85	0.55	3.217 down	2.939 down	0.02	10.71	MFS permease
68889	11.28	0.07	10.37	0.11	9.31	0.16	1.881 down	3.905 down	0.00	143.70	PDR-type ABC transporters
68924	9.09	0.07	12.02	0.03	13.14	1.18	7.616 up	16.584 up	0.02	13.09	cyclopropane/fatty acid synthase; plant related
68925	8.24	0.13	8.16	0.01	6.94	0.08	1.051 down	2.454 down	0.00	215.72	MFS permease
68927	10.73	0.07	10.82	0.05	9.55	0.18	1.069 up	2.266 down	0.00	75.12	unknown protein
68966	9.54	0.01	9.50	0.12	8.50	0.47	1.034 down	2.063 down	0.04	7.67	flavoprotein. putative
68990	13.19	0.08	13.51	0.08	11.36	0.96	1.242 up	3.578 down	0.05	7.29	MFS permease
69026	9.36	0.19	9.05	0.16	8.30	0.38	1.237 down	2.080 down	0.04	8.71	MFS permease

69131	11.42	0.05	11.28	0.09	10.41	0.14	1.105 down	2.018 down	0.00	61.91	Unknown protein with FYVE/PHD zinc finger domain.
69171	10.08	0.14	9.29	0.10	11.73	0.46	1.728 down	3.127 up	0.00	33.51	unknown protein
69181	2.95	0.10	2.78	0.21	4.78	0.13	1.129 down	3.553 up	0.00	176.34	unknown protein
69210	3.36	0.17	3.32	0.13	5.14	0.21	1.031 down	3.440 up	0.00	92.13	FoNIIA
69228	13.23	0.02	13.10	0.11	11.83	0.14	1.094 down	2.637 down	0.00	119.53	BioA Adenosylmethionine--amino--oxononanoate aminotransferase
69245	11.63	0.00	9.06	0.20	10.44	0.21	5.937 down	2.282 down	0.00	97.99	GH2 β-mannosidase
69257	8.19	0.05	8.62	0.00	9.40	0.39	1.344 up	2.311 up	0.02	11.97	Peptidase M22. glycoprotease
69276	7.51	0.17	5.98	0.09	11.81	0.72	2.896 down	19.619 up	0.00	84.77	GH30 endo-β-1.4-xylanase
69282	10.89	0.01	9.07	0.10	8.79	0.21	3.538 down	4.297 down	0.00	109.56	monocarboxylate transporter
69287	12.44	0.09	12.84	0.12	13.83	0.13	1.318 up	2.617 up	0.00	104.55	unknown protein
69291	3.15	0.18	3.27	0.04	4.64	0.29	1.081 up	2.804 up	0.00	36.18	FoNIIA
69303	7.40	0.01	10.33	0.01	9.60	0.39	7.661 up	4.594 up	0.00	54.45	unknown protein
69362	11.97	0.06	11.86	0.19	10.34	0.30	1.078 down	3.080 down	0.00	39.09	phospholipase A2 protein family
69375	9.24	0.17	7.03	0.04	8.18	0.53	4.623 down	2.086 down	0.02	14.06	unknown protein
69384	11.42	0.00	10.75	0.01	10.04	0.17	1.593 down	2.602 down	0.00	78.94	unknown protein
69399	11.51	0.09	12.09	0.11	12.83	0.02	1.500 up	2.501 up	0.00	283.31	mitochondrial 40S ribosomal protein [Aspergillus niger]. Possible homologue of yeast
69416	11.65	0.12	11.70	0.08	10.48	0.15	1.030 up	2.247 down	0.00	85.66	unknown protein
69426	8.97	0.18	7.66	0.15	7.56	0.44	2.487 down	2.651 down	0.02	11.05	copper transporter
69479	8.65	0.09	8.88	0.02	7.57	0.30	1.180 up	2.111 down	0.01	26.18	unknown protein
69486	3.20	0.07	3.44	0.03	5.14	0.28	1.179 up	3.826 up	0.00	70.12	unknown protein
69490	4.21	0.14	4.15	0.06	6.08	0.38	1.036 down	3.654 up	0.00	40.07	CE4 chitin deacetylase
69528	8.24	0.15	9.90	0.11	9.57	0.12	3.159 up	2.513 up	0.00	107.18	unknown protein
69555	4.41	0.28	4.56	0.22	6.31	0.25	1.111 up	3.726 up	0.00	51.60	aspartyl protease. Aspergillopepsin-like
69569	4.22	0.52	5.41	0.03	5.48	0.19	2.283 up	2.392 up	0.01	15.15	unknown protein
69574	13.48	0.05	13.03	0.03	10.72	0.21	1.370 down	6.767 down	0.00	234.91	MFS permease
69648	3.99	0.43	3.45	0.17	6.15	0.54	1.449 down	4.473 up	0.01	28.16	Cytochrome P450 CYP11/CYP12/CYP24/CYP27 subfamilies
69679	3.34	0.03	3.47	0.16	5.00	0.12	1.091 up	3.165 up	0.00	183.81	ABC transporter
69688	3.08	0.14	3.24	0.10	5.09	0.17	1.121 up	4.039 up	0.00	158.03	unknown protein
69692	4.44	0.14	5.08	0.25	8.85	0.75	1.557 up	21.254 up	0.00	47.42	Quinoprotein amine dehydrogenase beta chain-like protein
69695	10.00	0.03	9.83	0.08	6.78	0.46	1.123 down	9.330 down	0.00	76.87	Zn2Cys6 transcriptional regulator

69696	5.14	0.24	5.11	0.06	7.17	0.59	1.023 down	4.071 up	0.01	19.07	coenzyme F420-dependent N5.N10-methylene tetrahydromethanopterin reductase
69712	3.16	0.15	2.75	0.29	4.24	0.14	1.328 down	2.110 up	0.00	52.12	MFS permease
69742	5.51	0.21	6.65	0.16	8.54	0.28	2.206 up	8.201 up	0.00	110.41	esterase
69751	8.28	0.00	9.16	0.14	6.72	0.59	1.838 up	2.952 down	0.01	20.92	GH16
69757	9.89	0.04	10.62	0.08	10.94	0.17	1.663 up	2.068 up	0.00	37.27	Peptidyl-tRNA hydrolase
69777	7.40	0.29	8.26	0.13	9.20	0.14	1.812 up	3.486 up	0.00	70.44	unknown protein
69805	3.91	0.04	3.84	0.12	5.16	0.13	1.046 down	2.384 up	0.00	119.57	unknown protein
69811	8.80	0.19	10.17	0.09	5.90	0.28	2.584 up	7.460 down	0.00	246.69	γ-glutamyltransferase
69816	7.98	0.01	8.20	0.02	9.29	0.11	1.168 up	2.477 up	0.00	197.99	aminoglycoside 3-N-acetyltransferase
69825	8.08	0.29	9.31	0.02	9.28	0.24	2.347 up	2.300 up	0.01	21.11	unknown protein
69841	11.97	0.06	11.96	0.01	10.38	0.22	1.006 down	3.008 down	0.00	84.58	unknown protein
69857	7.36	0.00	7.10	0.13	8.41	0.47	1.198 down	2.075 up	0.02	10.71	unknown protein
69868	5.58	0.09	6.15	0.26	7.98	0.42	1.493 up	5.306 up	0.00	39.03	GDP-mannose a-mannosyltransferases;Distant relative
69870	3.28	0.09	3.36	0.05	4.97	0.25	1.058 up	3.223 up	0.00	66.76	FAD monooxygenase
69879	10.38	0.01	11.22	0.15	11.45	0.17	1.796 up	2.098 up	0.00	36.70	unknown protein
69881	3.36	0.11	3.48	0.03	4.73	0.22	1.086 up	2.584 up	0.00	53.35	Dit1p. spore wall maturation
69883	4.12	0.33	3.48	0.40	5.28	0.21	1.557 down	2.245 up	0.00	29.84	cytochrome P450. putative
69885	10.40	0.04	8.15	0.07	9.31	0.45	4.762 down	2.134 down	0.01	20.83	initiation factor 2B
69896	3.00	0.17	2.72	0.27	4.97	0.14	1.209 down	3.938 up	0.00	143.98	alpha/beta hydrolase
69901	3.40	0.07	3.02	0.01	4.60	0.03	1.300 down	2.309 up	0.00	1125.08	unknown protein
69920	3.63	0.16	3.71	0.01	4.93	0.12	1.058 up	2.467 up	0.00	114.94	unknown protein
69928	3.47	0.08	3.63	0.13	5.25	0.11	1.113 up	3.432 up	0.00	249.27	unknown protein
69933	10.15	0.10	10.40	0.06	8.71	0.15	1.185 up	2.711 down	0.00	152.19	unknown protein
69944	6.24	0.24	4.51	0.30	7.87	0.21	3.310 down	3.091 up	0.00	136.78	GH31 α-xylosidase/α-glucosidase
69946	11.99	0.07	10.08	0.12	10.39	0.48	3.780 down	3.042 down	0.01	15.77	NRPS. siderophore synthase
69950	4.41	0.06	4.82	0.02	5.81	0.19	1.329 up	2.635 up	0.00	67.30	unknown protein
69956	8.78	0.15	5.90	0.28	7.49	0.55	7.346 down	2.442 down	0.01	20.76	Alcohol dehydrogenase. class V
69972	11.66	0.18	11.21	0.18	9.45	0.16	1.364 down	4.630 down	0.00	144.76	Zn2Cys6 transcriptional regulator
70017	10.40	0.17	10.91	0.16	12.85	0.40	1.423 up	5.487 up	0.00	46.97	mitochondrial ribosomal protein S19/S15.
70021	6.18	0.02	5.85	0.05	7.25	0.23	1.259 down	2.096 up	0.00	47.30	CE3 acetyl xylan Esterase

70025	13.16	0.04	12.65	0.04	10.78	0.19	1.429 down	5.203 down	0.00	196.82	alcohol dehydrogenase. zinc-containing. putative
70092	9.13	0.40	8.33	0.30	7.73	0.19	1.736 down	2.636 down	0.01	18.18	unknown protein
70096	9.51	0.03	10.55	0.23	10.76	0.16	2.064 up	2.380 up	0.00	40.88	Gß-WD40 domain protein
70098	11.17	0.01	10.10	0.15	7.75	0.36	2.102 down	10.727 down	0.00	109.06	amino acid permease (GABA)
70108	9.23	0.07	8.85	0.11	7.01	0.25	1.302 down	4.649 down	0.00	101.37	MFS permease
70186	5.06	0.23	4.57	0.29	6.72	0.49	1.405 down	3.155 up	0.01	21.99	GH28 polygalacturonase/xylogalacturonan hydrolase
70195	3.38	0.05	3.36	0.12	4.52	0.09	1.016 down	2.210 up	0.00	158.57	unknown protein
70197	11.15	0.01	5.60	0.18	9.84	0.19	46.788 down	2.467 down	0.00	617.37	Zn-dependent ß-lactamase
70327	5.46	0.61	5.91	0.13	7.14	0.39	1.369 up	3.210 up	0.02	13.23	unknown protein
70334	10.11	0.04	8.65	0.30	6.98	0.81	2.761 down	8.772 down	0.01	16.47	short chain dehydrogenase/reductase
70339	4.26	0.23	4.10	0.10	5.40	0.24	1.120 down	2.200 up	0.00	31.06	cytochrome P450 monoounknown proteingenase (trichothecene C-15 hydrounknown
70349	12.45	0.04	10.46	0.05	9.34	0.36	3.978 down	8.651 down	0.00	80.55	MFS permease LIZ1
70355	13.53	0.04	10.95	0.08	9.32	0.37	5.994 down	18.605 down	0.00	139.38	SAM-dependent methylransferase
70365	10.54	0.08	6.53	0.12	7.16	0.48	16.042 down	10.414 down	0.00	69.55	unknown protein
70373	12.92	0.17	12.05	0.00	10.33	0.27	1.837 down	6.019 down	0.00	98.16	unknown protein
70375	11.87	0.01	10.13	0.13	8.22	0.38	3.347 down	12.561 down	0.00	101.32	amidase
70383	11.28	0.18	10.42	0.10	9.02	0.36	1.814 down	4.805 down	0.00	43.16	succinate semialdehyde dehydrogenase. NADP
70400	4.57	0.27	4.75	0.12	6.19	0.53	1.135 up	3.069 up	0.02	12.68	unknown protein
70414	9.68	0.10	9.46	0.11	7.93	0.31	1.160 down	3.363 down	0.00	44.28	Zn2Cys6 transcriptional regulator
70439	10.54	0.06	11.54	0.13	11.87	0.25	1.997 up	2.507 up	0.00	29.12	NADH-ubiquinone oxidoreductase
70491	11.78	0.16	5.64	0.54	10.47	0.08	70.700 down	2.492 down	0.00	329.23	esterase/lipase
70570	9.59	0.10	9.65	0.07	7.89	0.22	1.047 up	3.243 down	0.00	91.82	Zn2Cys6 transcriptional regulator
70599	4.79	0.01	5.12	0.07	6.19	0.19	1.258 up	2.636 up	0.00	68.71	unknown protein
70600	7.64	0.14	3.51	0.13	4.67	0.08	17.584 down	7.834 down	0.00	814.33	catalase. large subunit type
70608	12.03	0.00	7.00	0.03	8.32	0.65	32.737 down	13.100 down	0.00	55.95	unknown protein. HHE domains
70630	13.50	0.01	13.46	0.07	12.46	0.06	1.033 down	2.060 down	0.00	358.12	homoserine acetyltransferase family protein
70631	5.02	0.30	4.79	0.05	6.21	0.19	1.175 down	2.285 up	0.00	44.04	dipeptidyl peptidase 5
70803	9.92	0.09	8.92	0.11	12.17	0.09	2.003 down	4.754 up	0.00	896.65	bifunctional catalase/peroxidase
70829	10.01	0.06	9.05	0.06	7.40	0.33	1.944 down	6.140 down	0.00	75.50	MFS multidrug transporter. putative
70830	9.31	0.01	9.67	0.10	8.10	0.20	1.280 up	2.310 down	0.00	73.93	MFS permease

70837	3.91	0.09	4.52	0.26	4.97	0.24	1.519 up	2.084 up	0.01	14.75	E3 ubiquitin ligase
70842	9.42	0.32	8.29	0.00	7.04	0.22	2.181 down	5.192 down	0.00	79.52	Cytochrome P450 CYP2 subfamily
70845	9.04	0.11	10.70	0.06	11.59	0.16	3.171 up	5.855 up	0.00	225.01	GH55 β-1.3-glucanase
70855	7.71	0.04	8.41	0.05	8.75	0.17	1.629 up	2.052 up	0.00	38.21	unknown protein
70859	12.75	0.14	10.64	0.08	10.34	0.27	4.320 down	5.344 down	0.00	82.25	amidase
70894	12.76	0.12	10.28	0.11	11.00	0.19	5.560 down	3.395 down	0.00	124.59	unknown protein
70907	10.29	0.16	12.14	0.05	12.50	0.15	3.594 up	4.621 up	0.00	169.89	Ankyrin
70910	11.55	0.25	10.54	0.12	8.04	0.39	2.008 down	11.340 down	0.00	89.78	GMC oxidoreductase
70918	4.01	0.13	3.94	0.15	7.04	0.63	1.053 down	8.118 up	0.00	37.99	unknown protein
70919	5.81	0.07	7.64	0.25	7.57	0.16	3.550 up	3.373 up	0.00	83.83	SSCRP
70921	5.99	0.14	5.56	0.06	7.92	0.40	1.347 down	3.785 up	0.00	46.34	unknown protein
70923	8.68	0.00	9.31	0.13	7.26	0.09	1.543 up	2.682 down	0.00	367.09	unknown protein. only in fungi
70927	3.16	0.10	3.31	0.00	4.89	0.14	1.116 up	3.319 up	0.00	203.65	unknown protein
70932	10.28	0.11	9.93	0.01	9.24	0.29	1.280 down	2.064 down	0.01	15.03	MFS permease
70943	10.48	0.05	10.30	0.00	9.47	0.31	1.137 down	2.022 down	0.01	14.90	Histidine kinase. part of a two component signal transduction system
70956	7.25	0.06	5.04	0.39	5.53	0.28	4.615 down	3.284 down	0.00	35.89	cytochrome P450 monooxygenase
70961	10.69	0.08	9.65	0.07	9.32	0.42	2.050 down	2.579 down	0.02	11.57	dienelactone hydrolase
70962	2.90	0.14	2.90	0.04	4.90	0.03	1.001 up	4.010 up	0.00	782.85	tripeptide peptidase
70973	3.03	0.24	2.84	0.08	4.03	0.17	1.142 down	2.000 up	0.00	39.81	4-aminobutyrate aminotransferase
70991	5.83	0.11	7.99	0.09	7.65	0.31	4.481 up	3.539 up	0.00	46.14	unknown protein. ThiJ/PfpI domain
70996	6.37	0.24	6.68	0.16	7.52	0.25	1.238 up	2.219 up	0.01	19.64	SSCRP
71010	7.98	0.08	6.89	0.16	5.82	0.14	2.126 down	4.473 down	0.00	173.68	MDR-type ABC transporters
71029	10.94	0.06	12.79	0.26	14.14	0.26	3.606 up	9.178 up	0.00	128.82	Ctr copper transporter. putative
71059	9.70	0.14	9.20	0.12	8.55	0.45	1.410 down	2.224 down	0.05	7.42	MFS permease
71072	7.07	0.36	4.76	0.11	5.14	0.20	4.958 down	3.818 down	0.00	61.33	Gluconate kinase
71076	2.98	0.31	3.11	0.04	4.72	0.24	1.093 up	3.337 up	0.00	52.85	estrase/lipase
71078	2.56	0.14	2.61	0.07	4.17	0.10	1.038 up	3.064 up	0.00	227.56	Ca/CaM-dependent kinase-1
71080	9.42	0.16	9.01	0.04	7.51	0.16	1.325 down	3.746 down	0.00	140.87	Zn2Cys6 transcriptional regulator
71092	10.34	0.02	11.78	0.18	12.80	0.22	2.724 up	5.525 up	0.00	118.89	phytase
71094	8.58	0.08	10.76	0.04	7.22	0.39	4.531 up	2.569 down	0.00	92.03	Intradiol ring-cleavage dioxygenase

71095	4.58	0.09	4.96	0.07	6.55	0.29	1.300 up	3.922 up	0.00	60.55	Aldehyde dehydrogenase
71101	5.37	0.40	5.38	0.17	7.84	0.29	1.010 up	5.575 up	0.00	67.98	enoyl-CoA hydratase/isomerase
71103	2.80	0.00	3.16	0.09	4.46	0.07	1.281 up	3.159 up	0.00	490.87	dynamin GTPase. putative
71123	7.27	0.05	8.13	0.00	10.42	0.33	1.810 up	8.852 up	0.00	121.11	unknown protein
71125	3.77	0.12	3.85	0.14	5.65	0.38	1.057 up	3.678 up	0.00	35.72	unknown protein
71146	3.35	0.04	3.51	0.60	4.94	0.38	1.114 up	3.012 up	0.02	14.48	unknown protein
71154	5.27	0.47	5.49	0.21	6.53	0.29	1.158 up	2.392 up	0.02	13.04	unknown protein
71166	3.94	0.40	4.03	0.21	6.27	0.28	1.061 up	5.009 up	0.00	59.23	unknown protein
71167	2.85	0.42	2.59	0.09	4.56	0.14	1.202 down	3.268 up	0.00	68.95	SSCRP
71173	3.53	0.14	4.15	0.04	5.26	0.12	1.536 up	3.313 up	0.00	175.88	unknown protein
71180	6.22	0.05	6.74	0.00	8.02	0.44	1.436 up	3.483 up	0.01	21.50	unknown protein
71192	3.67	0.54	3.69	0.17	6.39	0.51	1.010 up	6.576 up	0.00	32.88	unknown protein
71196	5.10	0.16	5.49	0.01	6.57	0.44	1.308 up	2.759 up	0.02	14.03	unknown protein
71212	5.47	0.23	5.54	0.23	7.16	0.34	1.050 up	3.241 up	0.00	30.06	unique protein
71245	4.32	0.20	4.74	0.04	5.50	0.43	1.341 up	2.270 up	0.04	8.63	GH18. chitinase CHI18-1
71390	5.86	0.08	5.94	0.22	7.03	0.35	1.059 up	2.255 up	0.01	15.50	MRSP1/expansin-like
71399	12.72	0.02	11.50	0.01	11.62	0.11	2.328 down	2.149 down	0.00	134.28	endo-1.3-β-glucanase
71532	12.29	0.03	8.50	0.08	10.57	0.09	13.909 down	3.294 down	0.00	1106.21	GH71 α-1 3-glucanase
71554	3.71	0.25	3.59	0.13	5.35	0.12	1.084 down	3.111 up	0.00	115.03	GH5 β-1.3-mannanase/endo-β-1.4-mannosidase
71556	7.46	0.10	5.79	0.05	5.63	0.14	3.190 down	3.546 down	0.00	168.22	unknown protein
72071	5.70	0.37	5.95	0.16	7.48	0.62	1.184 up	3.438 up	0.02	10.72	Carbohydrate-Binding Module Family 13
72072	9.59	0.17	7.82	0.04	8.56	0.28	3.402 down	2.039 down	0.00	29.57	CE1 esterase (PHB?)
72183	8.58	0.17	10.25	0.13	7.50	0.15	3.161 up	2.115 down	0.00	228.09	SSCRP
72488	9.32	0.14	9.94	0.15	8.07	0.32	1.527 up	2.387 down	0.00	37.87	GH95 α-L-fucosidase
72567	12.99	0.15	9.03	0.15	15.25	0.15	15.565 down	4.797 up	0.00	1107.20	GH6 Cellobiohydrolase CEL6A/CBH2
72581	8.82	0.34	7.71	0.09	11.40	0.21	2.158 down	5.960 up	0.00	201.13	unknown protein
72602	12.02	0.21	11.09	0.08	10.62	0.05	1.905 down	2.632 down	0.00	111.61	ribosomal protein P2.
72605	4.31	0.34	4.06	0.18	5.70	0.25	1.190 down	2.628 up	0.00	35.55	GPCR. secretin like
72611	13.77	0.12	13.30	0.08	11.31	0.40	1.386 down	5.511 down	0.00	50.81	Zn2Cys6 transcriptional regulator
72612	11.50	0.06	10.43	0.12	9.70	0.26	2.100 down	3.496 down	0.00	50.55	acetylornithine deacetylase

72627	9.44	0.11	9.83	0.07	7.01	0.32	1.309 up	5.418 down	0.00	109.17	GPCR. secretin like
72632	11.70	0.02	9.17	0.11	8.11	0.54	5.777 down	12.000 down	0.00	48.17	GH27 α-galactosidase AGL1
72896	12.26	0.03	12.61	0.14	13.46	0.09	1.277 up	2.294 up	0.00	122.74	Dolichol-phosphate (beta-D) mannosyltransferase 2
72907	7.94	0.00	7.83	0.16	6.34	0.42	1.083 down	3.047 down	0.01	21.46	unknown protein with WSC domain
72922	12.41	0.00	8.98	0.13	9.52	0.07	10.758 down	7.407 down	0.00	1110.16	amino acid permease (GABA)
73005	2.66	0.02	2.75	0.00	4.49	0.09	1.061 up	3.560 up	0.00	637.33	GH79 β-glucuronidase
73016	3.02	0.01	3.20	0.05	5.41	0.13	1.132 up	5.225 up	0.00	508.13	aryl-alcohol oxidase
73023	3.43	0.02	3.45	0.15	4.99	0.16	1.013 up	2.949 up	0.00	116.79	unknown protein
73024	4.22	0.35	4.97	0.05	6.61	0.66	1.673 up	5.217 up	0.01	15.20	unknown protein
73039	7.30	0.29	6.89	0.19	5.33	0.23	1.333 down	3.926 down	0.00	58.14	APHPhosphotransferase enzyme family aligned
73048	13.24	0.10	14.22	0.14	14.75	0.06	1.975 up	2.846 up	0.00	187.40	unknown protein
73101	4.81	0.12	4.67	0.19	6.29	0.12	1.099 down	2.796 up	0.00	131.44	glucan endo-1.3-1.4-β-D-glucosidase
73102	7.51	0.03	6.53	0.05	6.11	0.21	1.966 down	2.636 down	0.00	48.61	GH39 β-xylosidase
73103	2.42	0.18	2.56	0.21	3.90	0.03	1.100 up	2.797 up	0.00	126.63	ribosomal protein P2.
73104	10.50	0.04	9.84	0.03	9.10	0.18	1.576 down	2.634 down	0.00	67.42	Zn2Cys6 transcriptional regulator
73119	2.75	0.11	2.92	0.24	4.63	0.10	1.119 up	3.672 up	0.00	172.00	unknown protein
73173	7.12	0.07	7.70	0.24	8.87	0.55	1.500 up	3.381 up	0.02	12.09	HFB1
73179	6.99	0.07	8.66	0.06	8.78	0.43	3.182 up	3.464 up	0.01	20.78	GH71 α-1 3-glucanase
73248	7.96	0.25	6.78	0.13	6.68	0.23	2.266 down	2.413 down	0.01	24.69	GH55 exo-1.3-β-glucanase
73250	3.38	0.32	3.07	0.09	6.51	1.36	1.239 down	8.723 up	0.03	9.56	Urea transporter
73344	3.24	0.00	3.42	0.12	5.06	0.19	1.136 up	3.532 up	0.00	116.51	Cytochrome P450 CYP3/CYP5/CYP6/CYP9 subfamilies
73417	13.29	0.04	12.77	0.06	12.07	0.11	1.432 down	2.331 down	0.00	137.53	bZIP transcriptional regulator MeaB
73516	14.59	0.04	13.92	0.08	13.56	0.34	1.590 down	2.049 down	0.03	10.24	Grg1 Glucose repressible protein
73536	9.46	0.12	9.48	0.15	12.80	0.11	1.012 up	10.116 up	0.00	782.96	NADP-glutamate dehydrogenase
73594	10.58	0.11	11.36	0.23	11.80	0.16	1.712 up	2.329 up	0.00	33.93	unknown protein
73623	4.63	0.01	4.23	0.24	5.99	0.21	1.317 down	2.574 up	0.00	65.62	flavoprotein monooxygenase
73632	7.19	0.14	4.42	0.10	11.10	0.28	6.821 down	15.028 up	0.00	591.15	CE5 acetyl xylan esterase AXE1
73638	9.01	0.17	4.94	0.12	13.63	0.38	16.794 down	24.546 up	0.00	555.34	CIP1
73643	7.81	0.10	3.13	0.22	12.33	0.16	25.659 down	22.910 up	0.00	2218.87	GH61 polysaccharide monooxygenase CEL61a
73783	9.81	0.18	9.85	0.04	11.27	0.15	1.032 up	2.759 up	0.00	100.93	mitochondrial co-chaperone GrpE . putative

73792	11.63	0.02	11.83	0.06	10.43	0.05	1.145 up	2.298 down	0.00	809.35	Zn2Cys6 transcriptional regulator
73815	13.99	0.17	13.46	0.07	12.91	0.18	1.436 down	2.114 down	0.00	31.96	unknown protein
73904	9.14	0.15	11.26	0.08	10.92	0.13	4.345 up	3.426 up	0.00	181.24	unknown protein
73912	8.48	0.11	9.00	0.04	9.66	0.20	1.438 up	2.263 up	0.00	37.75	unknown protein
73924	11.14	0.11	12.47	0.07	12.48	0.09	2.517 up	2.535 up	0.00	173.36	MDR-type ABC transporters
74017	11.41	0.02	11.14	0.08	10.11	0.09	1.207 down	2.458 down	0.00	235.41	unknown protein
74054	12.28	0.03	12.31	0.04	13.38	0.13	1.020 up	2.139 up	0.00	111.43	Exocyst component Sec6
74060	7.87	1.07	8.74	0.11	11.21	0.26	1.822 up	10.096 up	0.00	32.26	SSCRP
74162	12.52	0.00	12.17	0.06	11.39	0.12	1.277 down	2.200 down	0.00	107.12	Helicase. C-terminal
74194	13.02	0.12	11.13	0.28	11.40	0.27	3.702 down	3.064 down	0.00	35.73	mannitol dehydrogenase LXR1
74214	9.95	0.20	8.46	0.00	8.51	0.27	2.813 down	2.706 down	0.00	30.94	unknown protein
74215	6.96	0.15	7.80	0.03	5.73	0.29	1.792 up	2.347 down	0.00	55.58	RTA1-like protein. 7 TM. responds to xenobiotic stimuli
74228	11.70	0.13	13.10	0.00	13.21	0.23	2.632 up	2.849 up	0.00	47.01	oleate δ-12 desaturase
74278	12.26	0.12	10.73	0.16	11.08	0.24	2.893 down	2.268 down	0.00	31.14	Pyridine nucleotide-disulphide oxidoreductase. class-II
74379	8.43	0.09	8.66	0.04	9.46	0.13	1.175 up	2.035 up	0.00	67.31	ERG2 C-8 sterol isomerase; catalyzes the isomerization of the delta-8 double bond to t
74421	10.38	0.00	10.60	0.12	11.94	0.35	1.164 up	2.954 up	0.00	28.66	unknown protein
74476	11.49	0.06	12.09	0.07	10.18	0.21	1.512 up	2.470 down	0.00	97.69	unknown protein
74486	10.91	0.02	11.51	0.02	12.03	0.13	1.513 up	2.165 up	0.00	82.02	unknown protein
74502	13.25	0.12	12.63	0.04	12.24	0.04	1.539 down	2.020 down	0.00	172.70	Aldehyde dehydrogenase
74515	13.53	0.07	12.21	0.05	12.24	0.31	2.508 down	2.449 down	0.01	21.27	unknown protein
74563	11.02	0.03	10.93	0.03	9.25	0.18	1.067 down	3.411 down	0.00	153.07	unknown protein
74576	13.56	0.11	13.52	0.07	12.22	0.03	1.033 down	2.544 down	0.00	407.74	BZIP transcriptional regulator
74622	11.27	0.03	11.37	0.08	12.49	0.20	1.068 up	2.317 up	0.00	51.00	unknown protein
74701	10.65	0.17	10.59	0.05	9.43	0.25	1.043 down	2.336 down	0.00	32.00	GTP-binding protein involved in protein synthesis
74861	9.15	0.03	9.15	0.05	7.77	0.08	1.000 up	2.614 down	0.00	457.84	unknown protein
74943	9.15	0.05	9.97	0.04	10.62	0.14	1.760 up	2.763 up	0.00	110.40	Diphthine synthase
74971	12.24	0.05	12.23	0.07	11.16	0.33	1.002 down	2.104 down	0.01	16.93	unknown protein
75027	13.85	0.10	12.58	0.05	12.52	0.07	2.399 down	2.514 down	0.00	218.64	unknown protein
75036	12.99	0.08	12.29	0.13	11.32	0.32	1.618 down	3.168 down	0.00	30.70	GH63 α-glucosidase
75156	4.13	0.04	3.53	0.17	5.94	0.19	1.519 down	3.506 up	0.00	162.97	unknown protein

75247	4.67	0.02	4.44	0.26	6.06	0.17	1.176 down	2.611 up	0.00	73.54	AAA+-type ATPase
75260	11.33	0.02	10.36	0.05	10.03	0.09	1.954 down	2.455 down	0.00	208.69	RNA polymerase H/23 kD subunit
75271	9.55	0.06	9.16	0.19	11.33	0.33	1.311 down	3.421 up	0.00	54.50	pyridoxine biosynthesis protein PDX1
75290	7.46	0.04	9.70	0.05	6.17	0.18	4.744 up	2.431 down	0.00	411.01	dynamin GTPase
75294	11.15	0.14	10.39	0.08	12.50	0.18	1.696 down	2.545 up	0.00	136.55	BCAT_beta_family
75295	13.34	0.06	12.39	0.02	11.98	0.16	1.935 down	2.580 down	0.00	79.59	Trehalose-6-phosphate synthase component TPS1 and related subunits
75361	11.71	0.07	11.14	0.05	9.61	0.19	1.483 down	4.283 down	0.00	149.15	unknown protein
75403	11.05	0.15	11.16	0.03	9.94	0.20	1.074 up	2.159 down	0.00	46.21	CsdB Selenocysteine lyase
75409	11.49	0.00	10.63	0.13	10.44	0.08	1.811 down	2.064 down	0.00	107.30	Phospholipid-translocating P-type ATPase. flippase; putative
75470	7.00	0.15	7.68	0.19	8.19	0.22	1.594 up	2.271 up	0.01	23.83	unknown protein
75609	13.14	0.03	13.37	0.10	14.26	0.12	1.178 up	2.180 up	0.00	89.17	Rpl18 gene encoding 60sRibosomal protein L18.
75704	9.58	0.10	10.48	0.16	10.64	0.09	1.867 up	2.085 up	0.00	62.22	Adenosine 5'-phosphosulfate kinase
75713	11.87	0.17	9.07	0.00	10.78	0.36	6.996 down	2.139 down	0.00	48.30	Cytochrome P450 CYP2 subfamily
75758	12.19	0.08	12.44	0.08	11.18	0.34	1.187 up	2.014 down	0.01	18.55	unknown protein
75884	10.22	0.12	8.43	0.12	7.36	0.79	3.472 down	7.271 down	0.02	14.53	unknown protein
75923	11.65	0.32	10.59	0.18	10.03	0.31	2.091 down	3.079 down	0.01	20.54	unknown protein
75985	11.16	0.10	12.43	0.16	13.10	0.24	2.407 up	3.846 up	0.00	61.91	Isochorismatase hydrolase
75995	12.90	0.26	13.24	0.29	14.04	0.26	1.266 up	2.204 up	0.02	14.42	ssDNA binding protein
76034	12.11	0.25	10.62	0.02	10.77	0.24	2.795 down	2.527 down	0.00	31.28	Iron/ascorbate family oxidoreductases
76073	12.03	0.27	12.70	0.03	13.46	0.32	1.587 up	2.692 up	0.01	18.96	U6 snRNA-associated Sm-like protein LSm6
76075	11.73	0.01	13.52	0.05	13.25	0.27	3.472 up	2.873 up	0.00	44.32	unknown protein
76136	12.16	0.21	12.02	0.18	9.83	0.28	1.097 down	4.994 down	0.00	82.07	unknown protein
76151	10.40	0.04	10.84	0.04	11.72	0.13	1.358 up	2.492 up	0.00	125.32	GT31 ß-glycosyltransferase
76200	11.85	0.07	12.04	0.06	13.12	0.27	1.138 up	2.411 up	0.00	30.42	unknown protein
76210	7.02	0.19	3.12	0.05	10.47	1.49	14.836 down	10.988 up	0.01	27.48	GH62 α-L-arabinofuranosidase ABF2
76220	13.13	0.01	12.86	0.21	12.11	0.14	1.205 down	2.030 down	0.00	38.42	Zn2Cys6 transcriptional regulator
76238	13.36	0.08	8.09	0.13	10.90	0.09	38.819 down	5.536 down	0.00	1524.02	plasma membrane H+ ATPase
76288	10.81	0.14	9.95	0.06	9.42	0.22	1.806 down	2.610 down	0.00	39.21	short chain dehydrogenase/reductase
76360	3.67	0.06	3.52	0.10	6.00	0.29	1.105 down	5.019 up	0.00	106.54	unknown protein. 1 TM domain
76366	12.29	0.10	7.39	0.04	10.51	0.16	29.853 down	3.451 down	0.00	699.15	NADH:flavin oxidoreductase/12-oxophytodienoate reductase

76421	11.00	0.06	10.53	0.10	9.77	0.31	1.382 down	2.333 down	0.01	18.06	aromatic ring-opening dioxygenase LigB subunit. putative
76453	12.40	0.13	12.63	0.09	13.52	0.34	1.175 up	2.179 up	0.02	13.71	MDR-type ABC transporters
76532	6.76	0.08	7.38	0.20	9.34	0.23	1.535 up	6.009 up	0.00	130.32	ribosomal protein (60S) P0
76543	11.28	0.10	11.20	0.05	10.27	0.08	1.052 down	2.013 down	0.00	150.60	ARF GTPase activator (Csx2). putative
76601	12.12	0.02	5.48	0.03	10.45	0.24	100.220 down	3.183 down	0.00	722.95	Sulfite oxidase. molybdopterin-binding component
76620	10.63	0.23	9.60	0.25	9.27	0.31	2.041 down	2.566 down	0.01	15.31	Glycerol-3-phosphate dehydrogenase
76633	11.50	0.02	11.62	0.15	9.29	0.49	1.083 up	4.622 down	0.00	35.25	MDR multidrug transporter
76659	7.35	0.19	7.65	0.25	9.85	0.23	1.222 up	5.631 up	0.00	108.65	unknown protein
76672	7.05	0.16	6.68	0.05	11.84	1.69	1.287 down	27.778 up	0.02	14.51	GH3 β-glucosidase BGL1/CEL3a
76677	11.37	0.10	11.19	0.12	10.05	0.21	1.132 down	2.490 down	0.00	49.36	Zn2Cys6 transcriptional regulator
76682	3.70	0.03	4.03	0.15	5.98	0.72	1.259 up	4.843 up	0.02	14.21	PDR-type ABC transporters
76705	11.75	0.11	11.87	0.00	10.54	0.18	1.082 up	2.325 down	0.00	74.16	Zn2Cys6 transcriptional regulator
76713	12.89	0.10	12.25	0.25	11.20	0.15	1.567 down	3.232 down	0.00	76.30	unknown protein
76740	10.18	0.00	10.76	0.29	11.91	0.20	1.492 up	3.307 up	0.00	53.58	zuotin
76766	12.59	0.03	11.97	0.02	11.43	0.21	1.542 down	2.240 down	0.00	35.28	xanthine/uracil permease
76822	9.21	0.06	11.86	0.09	8.00	0.29	6.287 up	2.302 down	0.00	193.82	unknown protein
76828	10.58	0.04	11.00	0.09	11.61	0.21	1.334 up	2.039 up	0.01	26.04	exosome complex exonuclease RRP40. putative
76897	14.55	0.13	14.11	0.12	13.07	0.41	1.363 down	2.796 down	0.01	15.68	MFS permease
76910	10.70	0.06	9.47	0.15	9.58	0.09	2.344 down	2.174 down	0.00	95.87	monocarboxylate transporter
76986	11.77	0.05	11.61	0.08	10.30	0.26	1.119 down	2.765 down	0.00	44.96	unknown protein
77138	12.04	0.12	12.25	0.16	13.30	0.31	1.151 up	2.395 up	0.01	20.72	unknown protein
77167	9.37	0.07	9.36	0.23	7.57	0.27	1.011 down	3.488 down	0.00	57.95	unknown protein
77202	11.22	0.03	8.48	0.12	9.03	0.11	6.662 down	4.546 down	0.00	421.43	short chain dehydrogenase/reductase
77291	9.94	0.28	10.38	0.03	8.60	0.38	1.359 up	2.532 down	0.01	24.44	Zn2Cys6 transcriptional regulator
77299	8.93	0.20	7.05	0.04	10.11	0.51	3.682 down	2.262 up	0.00	38.59	GH2 Exo-β-D-glucosaminidase GLS93
77423	12.08	0.06	10.50	0.05	9.70	0.54	2.978 down	5.195 down	0.01	21.60	Jacalin-like lectin
77495	12.71	0.15	12.95	0.25	13.83	0.12	1.182 up	2.172 up	0.00	40.03	eukaryotic translation initiation factor 3 subunit 11.
77506	13.60	0.03	13.37	0.03	12.54	0.15	1.173 down	2.078 down	0.00	66.93	GH5 β-glycosidase CEL5d
77517	10.15	0.08	10.32	0.01	11.92	0.15	1.129 up	3.423 up	0.00	195.91	MFS hexose transporter
77547	10.34	0.02	8.96	0.06	7.65	0.26	2.598 down	6.449 down	0.00	120.91	GT1 glycosyltransferase

77557	11.27	0.06	8.40	0.01	8.77	0.24	7.316 down	5.660 down	0.00	154.68	GT glycosyltransferases not yet assigned to a family. 3 TMs
77593	7.95	0.06	7.62	0.14	6.24	0.37	1.259 down	3.284 down	0.00	28.93	unknown protein
77602	14.05	0.04	13.46	0.11	12.91	0.29	1.503 down	2.212 down	0.01	16.99	A.a-trehalose-phosphate synthase
77629	8.57	0.03	9.46	0.11	10.04	0.47	1.847 up	2.756 up	0.03	10.66	unknown protein
77743	9.97	0.12	10.86	0.02	8.37	0.24	1.852 up	3.027 down	0.00	124.03	unknown protein
77878	11.32	0.04	11.19	0.00	13.11	0.14	1.096 down	3.446 up	0.00	284.36	C2H2 transcriptional regulator
77915	10.12	0.11	10.21	0.12	11.14	0.24	1.061 up	2.019 up	0.01	23.05	unknown protein
77932	10.51	0.12	11.04	0.18	12.22	0.36	1.446 up	3.282 up	0.01	25.78	Ribosomal protein L30.
77979	10.63	0.02	11.24	0.30	11.90	0.13	1.525 up	2.412 up	0.00	38.70	SmallSubunit. aps3. of the adaptor protein complex AP-3 of clathrin-coated vesicles; i
78010	12.14	0.00	12.01	0.02	10.86	0.17	1.096 down	2.423 down	0.00	89.58	ankyrin containing protein
78012	10.93	0.01	10.48	0.10	9.85	0.30	1.359 down	2.109 down	0.01	14.73	Sensory transduction histidine kinase
78037	11.34	0.01	11.05	0.03	9.73	0.25	1.220 down	3.040 down	0.00	59.78	unknown protein
78042	11.55	0.00	11.42	0.05	10.35	0.22	1.091 down	2.296 down	0.00	44.22	unknown protein
78264	10.33	0.09	11.02	0.04	11.48	0.17	1.611 up	2.211 up	0.00	45.79	Peptide methionine sulfoxide reductase
78274	8.07	0.12	7.77	0.33	9.13	0.23	1.231 down	2.084 up	0.01	27.07	phosphatidylinositol-3-phosphate 5-kinase; fab1
78301	12.93	0.03	12.63	0.25	11.75	0.14	1.228 down	2.251 down	0.00	45.19	cation diffusion facilitator family transporter
78357	13.88	0.26	13.71	0.25	12.39	0.14	1.122 down	2.796 down	0.00	50.77	NADH-quinone oxidoreductase. putative
78391	9.55	0.04	9.00	0.00	7.39	0.21	1.462 down	4.460 down	0.00	131.62	unknown protein
78465	11.54	0.01	11.95	0.04	13.06	0.17	1.324 up	2.856 up	0.00	106.76	siderophore transporter
78552	11.30	0.03	11.19	0.00	9.97	0.14	1.082 down	2.510 down	0.00	135.60	Aromatic L-amino acid decarbounknown proteinlase
78585	11.05	0.09	5.74	0.16	8.79	0.93	39.746 down	4.811 down	0.01	27.21	MFS permease
78611	13.06	0.03	12.16	0.07	11.02	0.26	1.873 down	4.125 down	0.00	70.52	phospholipase D
78639	11.45	0.01	13.71	0.19	12.98	0.13	4.778 up	2.880 up	0.00	150.16	Serine/threonine protein kinase
78679	9.74	0.03	10.17	0.21	10.78	0.15	1.345 up	2.057 up	0.00	33.79	mitochondrial carrier protein
78688	14.18	0.04	13.41	0.11	12.72	0.06	1.703 down	2.746 down	0.00	292.77	Winged helix repressor DNA-binding
78713	11.27	0.08	9.68	0.08	9.19	0.25	3.003 down	4.203 down	0.00	72.48	GH72 ß-(1-3) glucanosyltransferase
78740	9.06	0.04	9.11	0.07	10.22	0.29	1.037 up	2.234 up	0.01	24.94	v-SNARE Bet1. ER-Golgi complex
78773	13.46	0.02	13.36	0.04	12.41	0.15	1.069 down	2.061 down	0.00	69.67	long-chain-fatty-acid--CoA ligase 2
78792	10.79	0.02	9.88	0.01	8.75	0.25	1.875 down	4.111 down	0.00	81.04	unknown protein
78797	12.89	0.02	12.77	0.08	11.08	0.13	1.085 down	3.513 down	0.00	257.08	xanthine dehydrogenase

78799	11.05	0.01	10.95	0.04	9.27	0.29	1.076 down	3.434 down	0.00	60.14	Zn2Cys6 transcriptional regulator
78811	12.36	0.16	12.09	0.11	11.25	0.45	1.201 down	2.151 down	0.04	7.62	unknown protein
78828	12.00	0.13	13.51	0.12	13.57	0.47	2.840 up	2.953 up	0.02	13.12	carboxylesterase type B
78864	9.20	0.05	9.45	0.09	10.63	0.26	1.190 up	2.701 up	0.00	41.71	GcvTGlycine cleavage system T protein
78866	12.97	0.13	12.98	0.00	11.93	0.26	1.009 up	2.054 down	0.01	24.88	unknown protein
78895	11.44	0.01	11.45	0.14	9.99	0.21	1.006 up	2.724 down	0.00	68.48	Zn2Cys6 transcriptional regulator
78970	14.36	0.04	13.06	0.05	12.08	0.07	2.472 down	4.870 down	0.00	1013.94	mitochondrial (phosphate) carrier
78984	12.56	0.03	12.18	0.05	11.49	0.27	1.304 down	2.108 down	0.01	18.87	unknown protein
79034	10.12	0.05	10.67	0.00	11.60	0.04	1.463 up	2.784 up	0.00	1196.97	ABC1 family protein
79059	12.11	0.05	12.19	0.02	13.17	0.20	1.057 up	2.079 up	0.00	40.97	Myo-inositol-1-phosphate synthase. associated to cellulase signal transduction (PMID
79106	14.27	0.15	14.68	0.03	15.32	0.04	1.328 up	2.075 up	0.00	148.32	40s ribosomal protein L44e by homology to the corresponding protein of several yeas
79153	12.21	0.11	11.99	0.14	10.67	0.38	1.159 down	2.901 down	0.01	22.27	HyuA. N-methylhydantoinase A/acetone carboxylase. beta subunit
79187	10.09	0.01	11.14	0.19	11.33	0.06	2.070 up	2.371 up	0.00	111.19	DNA polymerase alpha-primase complex. polymerase-associated subunit B
79202	11.83	0.04	10.58	0.09	13.63	0.12	2.388 down	3.461 up	0.00	633.86	MFS permease. associated with cellulose signalling
79222	10.00	0.06	6.26	0.02	7.77	0.35	13.373 down	4.677 down	0.00	93.61	unknown protein
79229	11.34	0.04	11.92	0.19	12.67	0.14	1.497 up	2.508 up	0.00	65.15	elongation factor G (Tu) and the LepA membrane protein family.
79237	11.22	0.15	10.73	0.09	9.59	0.12	1.407 down	3.081 down	0.00	139.02	unknown protein
79271	13.81	0.14	13.59	0.08	12.21	0.16	1.162 down	3.028 down	0.00	110.77	malate synthase
79345	8.07	0.06	9.79	0.21	9.90	0.50	3.286 up	3.544 up	0.01	15.00	unknown protein. Mpv17/PMP22 family
79361	10.00	0.14	11.17	0.06	11.26	0.10	2.257 up	2.408 up	0.00	112.48	unknown protein
79396	14.17	0.05	12.88	0.06	13.11	0.27	2.453 down	2.093 down	0.01	22.97	GT2 ß-glycosyltransferase. related to hyaluronan synthases
79426	8.95	0.05	9.45	0.06	10.04	0.31	1.416 up	2.139 up	0.02	14.49	unknown protein
79461	12.65	0.23	11.97	0.02	11.59	0.15	1.603 down	2.087 down	0.00	30.74	unknown protein
79487	10.89	0.02	11.22	0.02	12.14	0.31	1.260 up	2.392 up	0.01	21.82	Mannose-P-dolichol utilization defect 1 protein
79565	9.72	0.15	10.62	0.08	10.92	0.28	1.870 up	2.302 up	0.01	18.15	Thioredoxin reductase TrxB
79602	10.06	0.08	10.07	0.07	7.88	0.44	1.010 up	4.513 down	0.00	40.67	GH81 endo-1.3-β-glucanase
79606	13.12	0.07	12.41	0.04	12.00	0.15	1.639 down	2.177 down	0.00	58.33	GH115 xylan-α-1.2-glucuronidase or α-(4-O-methyl)-glucuronidase
79644	9.95	0.11	9.44	0.08	11.12	0.37	1.424 down	2.247 up	0.01	24.81	metal ion transporter SMF2
79661	11.84	0.21	11.87	0.05	10.63	0.25	1.018 up	2.319 down	0.00	33.14	unknown protein
79725	10.00	0.03	9.87	0.19	8.37	0.10	1.094 down	3.115 down	0.00	179.55	Zn2Cys6 transcriptional regulator

79726	9.56	0.02	12.54	0.06	12.74	0.39	7.847 up	9.055 up	0.00	80.68	unknown protein
79741	9.16	0.12	8.30	0.09	10.30	0.36	1.807 down	2.212 up	0.00	34.55	ER-bound Farnesyl-diphosphate farnesyltransferase
79756	10.21	0.01	10.43	0.03	8.93	0.19	1.166 up	2.416 down	0.00	84.60	F-Box/WD40 repeat protein FWD-1. targets the clock component FRQ for degradation
79779	8.12	0.01	8.82	0.08	10.56	0.62	1.626 up	5.436 up	0.01	20.20	unknown protein
79807	12.64	0.03	12.51	0.12	11.58	0.38	1.096 down	2.083 down	0.02	10.82	Aspartictype endopeptidase ctsD
79816	5.84	0.06	5.57	0.28	7.72	0.46	1.212 down	3.680 up	0.01	28.23	unknown protein; secreted
79823	10.21	0.28	9.81	0.01	9.14	0.21	1.320 down	2.100 down	0.01	19.97	DCL2. Dicer-like protein. involved in quelling
79933	8.82	0.31	9.16	0.10	10.47	0.34	1.273 up	3.151 up	0.01	24.57	mitochondrial ribosomal protein MRPL6.
79947	11.67	0.13	11.34	0.00	13.01	0.14	1.257 down	2.543 up	0.00	160.60	DNA-dependent RNA polymerase
79960	10.75	0.20	12.41	0.09	12.28	0.12	3.156 up	2.894 up	0.00	107.20	GH47 α-1.2-mannosidase
80019	13.35	0.14	14.15	0.01	14.60	0.23	1.739 up	2.366 up	0.00	28.81	short-chain dehydrogenase/reductase
80026	12.50	0.20	13.64	0.02	14.16	0.39	2.194 up	3.143 up	0.01	17.98	MFS permease
80034	12.23	0.07	12.52	0.07	11.17	0.16	1.226 up	2.088 down	0.00	85.54	DadA Glycine/D-amino acid oxidases (deaminating) [Amino acid transport and metabo
80056	6.62	0.14	7.67	0.20	8.38	0.53	2.072 up	3.390 up	0.02	11.44	RNA 3'-terminal phosphate cyclase . putative
80058	11.32	0.03	7.80	0.23	9.23	0.38	11.514 down	4.257 down	0.00	64.66	MFS permease
80096	12.88	0.04	12.76	0.05	11.57	0.43	1.083 down	2.464 down	0.02	13.76	unknown protein
80105	11.80	0.04	11.81	0.10	10.68	0.23	1.011 up	2.166 down	0.00	35.59	unknown protein
80137	11.09	0.10	10.84	0.12	9.92	0.22	1.188 down	2.247 down	0.00	34.51	unknown protein
80167	12.99	0.06	13.43	0.14	14.07	0.21	1.362 up	2.118 up	0.01	27.28	histone H4 variant
80211	6.02	0.05	6.56	0.01	8.77	0.31	1.450 up	6.721 up	0.00	106.49	ribosomal protein L14.
80288	11.80	0.06	12.06	0.05	13.24	0.33	1.203 up	2.718 up	0.01	26.07	cytochrome c oxidase polypeptide V
80291	12.38	0.04	12.23	0.02	11.02	0.30	1.110 down	2.580 down	0.00	30.64	Zn2Cys6 transcriptional regulator
80340	9.94	0.11	12.27	0.04	12.59	0.16	5.037 up	6.295 up	0.00	284.96	GT α-1.6-mannosyltransferase
80365	11.98	0.02	12.13	0.15	10.84	0.19	1.112 up	2.200 down	0.00	56.01	unknown protein
80425	10.33	0.05	9.88	0.04	9.18	0.21	1.363 down	2.221 down	0.00	35.11	unknown protein
80441	11.62	0.05	12.17	0.21	12.83	0.35	1.469 up	2.322 up	0.02	12.53	unknown protein
80523	10.05	0.07	9.78	0.09	8.67	0.18	1.208 down	2.604 down	0.00	71.69	pH-response regulator protein palC
80528	10.64	0.32	12.63	0.02	11.71	0.08	3.962 up	2.091 up	0.00	80.10	Gß-WD40 protein
80553	12.91	0.04	12.80	0.11	11.73	0.12	1.083 down	2.260 down	0.00	112.98	ADP-ribosylation factor like protein
80636	12.70	0.02	12.40	0.06	11.57	0.27	1.232 down	2.196 down	0.01	22.41	unknown protein

80639	3.54	0.13	4.93	0.30	5.56	0.47	2.616 up	4.050 up	0.01	17.71	iron transporter
80645	12.86	0.02	12.85	0.05	11.01	0.48	1.004 down	3.596 down	0.01	24.23	Amidase
80654	9.34	0.00	5.79	0.09	7.39	0.12	11.701 down	3.854 down	0.00	616.24	UDP-glucuronosyl and UDP-glucosyl transferase
80659	11.38	0.03	4.77	0.32	8.84	0.29	97.770 down	5.813 down	0.00	310.61	alcohol oxidase AOX1
80668	11.31	0.12	10.49	0.06	9.62	0.19	1.769 down	3.231 down	0.00	78.97	Zn2Cys6 transcriptional regulator
80685	10.18	0.01	10.58	0.01	8.09	0.35	1.314 up	4.278 down	0.00	74.07	unknown protein
80695	11.82	0.22	11.98	0.22	10.79	0.26	1.119 up	2.036 down	0.01	20.29	half-sized ABC transporter
80714	12.33	0.16	12.56	0.15	10.94	0.35	1.174 up	2.611 down	0.01	27.37	Dolichyl-phosphate-mannose a-mannosyltransferase
80733	6.63	0.00	7.20	0.17	7.86	0.09	1.493 up	2.345 up	0.00	99.06	unknown protein
80758	12.83	0.08	12.70	0.09	11.81	0.04	1.095 down	2.026 down	0.00	235.48	serine/threonine protein kinase nrc2
80843	2.56	0.10	2.50	0.11	4.05	0.16	1.043 down	2.809 up	0.00	113.23	26S proteasome regulatory complex subunit Rpn8
80871	11.71	0.15	10.92	0.10	9.91	0.41	1.732 down	3.483 down	0.01	20.85	unknown protein
80875	10.50	0.01	11.05	0.11	8.39	0.79	1.465 up	4.312 down	0.01	15.59	MFS permease
80980	11.74	0.07	14.20	0.08	14.00	0.15	5.500 up	4.778 up	0.00	253.40	peptidyl arginine deiminase
81004	8.68	0.02	8.23	0.09	7.54	0.14	1.369 down	2.202 down	0.00	65.45	aspartyl protease
81019	11.00	0.24	10.28	0.08	8.49	0.55	1.642 down	5.665 down	0.01	25.18	Zinc-containing alcohol dehydrogenase
81022	13.01	0.06	13.08	0.10	11.24	0.18	1.047 up	3.411 down	0.00	151.70	allantoate permease. 10 TM domains
81058	9.14	0.15	8.02	0.05	7.32	0.25	2.175 down	3.544 down	0.00	53.14	peptidase family S58
81082	7.86	0.09	11.34	0.10	12.52	1.11	11.207 up	25.313 up	0.01	19.53	aquaglyceroporin
81087	10.24	0.09	6.86	0.06	11.84	0.55	10.395 down	3.035 up	0.00	88.30	aminopeptidase Y
81096	10.40	0.14	10.44	0.08	14.28	0.11	1.024 up	14.686 up	0.00	1233.48	ferric reductase
81125	13.56	0.02	12.48	0.05	12.24	0.24	2.107 down	2.495 down	0.00	33.31	Amino acid permease
81150	9.24	0.09	9.84	0.16	8.06	0.24	1.513 up	2.272 down	0.00	56.07	unknown protein
81183	11.63	0.10	11.44	0.10	10.38	0.20	1.134 down	2.366 down	0.00	48.16	unknown protein
81271	10.46	0.09	11.72	0.04	12.10	0.43	2.399 up	3.113 up	0.01	15.84	xylitol dehydrogenase XDH1
81275	12.46	0.14	11.22	0.12	10.76	0.08	2.360 down	3.247 down	0.00	179.70	phytanoyl-CoA dioxygenase family protein
81362	8.71	0.08	9.08	0.03	9.85	0.05	1.290 up	2.199 up	0.00	340.39	Lipoate synthase
81383	11.62	0.21	13.23	0.12	14.27	0.17	3.063 up	6.292 up	0.00	163.75	GPCR . contains RGS domain
81389	12.22	0.02	10.34	0.09	9.96	0.18	3.699 down	4.788 down	0.00	166.38	phosphate/H+ symporter
81420	8.72	0.07	7.19	0.09	7.31	0.44	2.875 down	2.643 down	0.02	13.01	Argonaute siRNA chaperone (ARC) complex subunit

81423	11.59	0.21	11.46	0.31	12.89	0.29	1.091 down	2.463 up	0.01	23.69	unknown protein
81454	12.15	0.10	12.12	0.14	10.70	0.31	1.024 down	2.735 down	0.00	31.87	unknown protein
81473	9.92	0.05	10.28	0.04	10.95	0.39	1.282 up	2.031 up	0.04	8.56	caffeine-induced death protein 2
81511	11.39	0.09	10.52	0.04	10.09	0.25	1.824 down	2.453 down	0.00	28.53	formamidase
81609	11.86	0.07	11.44	0.07	10.84	0.31	1.338 down	2.033 down	0.02	12.39	GH15
81623	12.73	0.04	11.84	0.11	11.43	0.08	1.859 down	2.465 down	0.00	185.24	unknown protein. only in Hypocreaceae
81646	12.64	0.16	12.04	0.04	11.47	0.04	1.513 down	2.238 down	0.00	141.24	NADPH oxidase
81659	12.31	0.04	8.98	0.08	10.44	0.17	10.047 down	3.646 down	0.00	281.55	unknown protein
81676	10.96	0.02	10.38	0.12	8.48	0.53	1.496 down	5.583 down	0.00	29.12	salicylate hydroxylase
81700	12.67	0.08	12.48	0.11	11.24	0.06	1.144 down	2.691 down	0.00	317.99	mannose-6-phosphate isomerase
81720	11.65	0.01	11.36	0.06	10.25	0.26	1.222 down	2.629 down	0.00	37.61	Ume5 gen
81730	10.84	0.02	11.13	0.16	11.91	0.23	1.224 up	2.100 up	0.01	24.99	glutaredoxin
81774	12.24	0.09	12.04	0.02	10.75	0.20	1.141 down	2.798 down	0.00	72.24	unknown protein
81843	10.80	0.03	9.34	0.12	7.49	0.46	2.746 down	9.875 down	0.00	60.40	Alkaline phosphatase
81906	12.39	0.20	12.94	0.28	13.60	0.08	1.461 up	2.310 up	0.00	36.93	unknown protein
81911	12.61	0.08	12.46	0.12	11.41	0.18	1.107 down	2.299 down	0.00	56.94	unknown protein
81920	11.55	0.07	11.72	0.05	12.78	0.26	1.128 up	2.336 up	0.00	30.11	SSCRP
81955	2.89	0.27	2.90	0.09	4.46	0.19	1.008 up	2.970 up	0.00	63.52	nitrate reductase
81979	13.00	0.03	9.76	0.07	10.67	0.13	9.476 down	5.025 down	0.00	502.20	glutathione-S-transferase
82037	12.52	0.02	12.74	0.01	11.08	0.06	1.163 up	2.719 down	0.00	1080.27	Inorganic phosphate transporter
82049	10.25	0.12	10.91	0.02	13.17	0.29	1.577 up	7.546 up	0.00	124.96	unknown protein
82095	6.16	0.06	6.23	0.01	7.49	0.51	1.055 up	2.518 up	0.02	10.85	ammonium transporter. high affinity
82105	8.91	0.26	7.45	0.09	6.81	0.40	2.755 down	4.288 down	0.01	26.34	PDR-type ABC transporters
82204	8.08	0.16	7.92	0.03	6.79	0.37	1.113 down	2.439 down	0.01	16.85	ammonium transporter. high affinity
82235	12.76	0.10	12.99	0.11	14.27	0.18	1.174 up	2.844 up	0.00	82.92	GH31 α-glucosidase
82246	11.95	0.01	12.39	0.07	13.11	0.10	1.358 up	2.245 up	0.00	134.65	GCPR. mPR-type
82286	12.36	0.02	12.93	0.02	13.39	0.29	1.478 up	2.044 up	0.02	13.92	unknown protein
82309	11.83	0.00	11.35	0.02	9.45	0.52	1.390 down	5.190 down	0.01	28.35	MFS permease
82321	11.04	0.07	4.10	0.23	9.46	0.74	122.123 down	2.981 down	0.00	81.69	Aquaporin
82327	12.55	0.05	10.27	0.00	10.04	0.28	4.846 down	5.701 down	0.00	94.75	PDR-type ABC transporters

82374	12.43	0.04	11.41	0.07	10.39	0.75	2.028 down	4.120 down	0.04	8.47	unknown protein
82403	13.55	0.08	13.28	0.07	11.61	0.14	1.198 down	3.822 down	0.00	238.93	Acyl-CoA dehydrogenase
82425	4.54	0.05	4.64	0.22	5.97	0.13	1.070 up	2.685 up	0.00	90.83	unknown protein
82523	9.32	0.16	9.12	0.13	8.22	0.17	1.146 down	2.139 down	0.00	39.93	unknown protein
82560	8.44	0.03	9.50	0.12	9.95	0.25	2.091 up	2.857 up	0.00	37.30	Ribokinase
82591	12.47	0.05	10.83	0.04	10.72	0.13	3.120 down	3.377 down	0.00	202.45	unknown protein with oxidoreductase domain
82606	11.08	0.08	11.17	0.07	12.20	0.30	1.068 up	2.174 up	0.01	20.46	Mn2+ homeostasis protein Per1
82616	12.20	0.23	9.83	0.11	10.03	0.33	5.179 down	4.507 down	0.00	48.54	GH5 membrane bound endoglucanase CEL5b
82619	12.74	0.02	12.92	0.03	9.54	0.48	1.131 up	9.196 down	0.00	78.59	arginosuccinate synthetase
82626	8.46	0.30	8.59	0.04	6.77	0.49	1.093 up	3.230 down	0.01	18.72	half-sized ABC transporter
82628	4.03	0.31	6.08	0.37	5.50	0.19	4.132 up	2.764 up	0.00	33.82	acetyltransferase SidF
82633	12.16	0.07	10.18	0.08	9.90	0.29	3.948 down	4.784 down	0.00	67.84	GH72 β-1 3-glucanosyltransferase
82662	14.11	0.09	7.87	0.06	11.87	0.20	75.475 down	4.705 down	0.00	753.52	Epl1/Sm1
102383	11.29	0.11	10.66	0.07	9.28	0.16	1.548 down	4.029 down	0.00	167.85	unknown protein
102385	2.87	0.14	2.85	0.19	4.29	0.12	1.011 down	2.671 up	0.00	105.82	unknown protein
102386	6.71	0.25	7.40	0.12	7.97	0.26	1.618 up	2.391 up	0.01	19.16	unique protein
102411	10.42	0.10	9.81	0.06	9.02	0.21	1.522 down	2.640 down	0.00	48.26	unknown protein
102416	10.58	0.12	9.48	0.13	9.46	0.10	2.132 down	2.163 down	0.00	76.63	amino acid permease PotE
102454	6.28	0.13	7.08	0.00	7.39	0.17	1.741 up	2.160 up	0.00	41.71	unknown protein
102467	4.80	0.15	4.22	0.39	6.49	0.09	1.499 down	3.218 up	0.00	101.95	unknown protein
102499	2.71	0.06	2.66	0.04	5.44	0.71	1.036 down	6.618 up	0.01	25.12	Zn2Cys6 transcriptional regulator
102500	5.32	0.02	5.12	0.17	7.26	0.27	1.148 down	3.845 up	0.00	82.90	MRSP1/expansin-like
102567	2.61	0.05	3.22	0.45	4.23	0.18	1.516 up	3.056 up	0.00	32.10	unknown protein
102593	9.10	0.21	9.73	0.10	10.44	0.18	1.550 up	2.534 up	0.00	40.50	Mitochondrial ribosomal protein L17
102637	3.06	0.09	3.95	0.08	4.92	0.11	1.847 up	3.624 up	0.00	216.12	unique protein
102680	2.42	0.01	2.34	0.11	3.67	0.02	1.058 down	2.377 up	0.00	643.98	unique protein
102738	12.18	0.00	10.17	0.11	11.09	0.52	4.027 down	2.121 down	0.02	12.30	unknown protein
102773	3.23	0.07	3.98	0.12	5.86	0.16	1.674 up	6.196 up	0.00	279.58	unknown protein
102774	4.89	0.02	5.37	0.37	7.28	0.51	1.389 up	5.248 up	0.01	25.71	unknown protein
102779	6.03	0.03	7.31	0.15	7.14	0.30	2.439 up	2.160 up	0.01	18.27	unknown protein

102780	5.79	0.19	6.21	0.21	7.84	0.55	1.340 up	4.147 up	0.01	17.45	unknown protein
102787	5.81	0.18	6.72	0.03	7.19	0.32	1.879 up	2.600 up	0.01	18.36	unique protein
102788	2.81	0.29	3.06	0.10	4.57	0.11	1.186 up	3.375 up	0.00	99.94	unique protein
102816	12.30	0.15	11.70	0.07	11.07	0.11	1.513 down	2.344 down	0.00	82.91	Zn2Cys6 transcriptional regulator
102830	12.69	0.24	10.56	0.01	11.18	0.33	4.386 down	2.858 down	0.00	31.43	unknown protein
102846	10.73	0.20	10.43	0.26	9.57	0.22	1.230 down	2.239 down	0.01	20.71	unknown protein
102850	9.00	0.04	9.04	0.03	7.85	0.05	1.029 up	2.222 down	0.00	696.54	unique protein
102851	9.23	0.12	11.30	0.17	12.54	1.01	4.207 up	9.908 up	0.02	11.70	SSCRP
102863	10.44	0.01	10.29	0.01	8.92	0.24	1.103 down	2.858 down	0.00	58.72	unknown protein
102876	6.34	0.19	6.39	0.02	5.30	0.37	1.040 up	2.045 down	0.02	12.39	unique protein
102887	4.50	0.04	5.04	0.11	6.19	0.38	1.447 up	3.225 up	0.01	24.29	unique protein
102892	13.39	0.10	13.17	0.10	12.01	0.26	1.165 down	2.607 down	0.00	36.21	unknown protein
102906	10.74	0.05	9.19	0.13	8.62	0.27	2.927 down	4.337 down	0.00	60.77	unique protein
102920	12.11	0.00	9.90	0.07	9.47	0.25	4.625 down	6.239 down	0.00	121.53	C2H2 transcriptional regulator
102927	3.52	0.04	4.01	0.33	4.89	0.08	1.412 up	2.592 up	0.00	53.72	Serine/threonine protein kinase
102933	8.32	0.02	9.19	0.08	6.35	0.23	1.831 up	3.914 down	0.00	189.43	unknown protein. only in Gibberella
102935	3.11	0.07	3.28	0.22	4.68	0.09	1.124 up	2.963 up	0.00	146.45	unknown protein
102936	5.65	0.07	5.95	0.11	7.48	0.20	1.234 up	3.549 up	0.00	104.24	unknown protein
102953	9.59	0.01	7.98	0.07	7.35	0.15	3.052 down	4.708 down	0.00	218.57	dityrosine transporter. required for spore wall synthesis
102960	10.84	0.04	11.73	0.09	12.04	0.25	1.842 up	2.297 up	0.01	25.15	unique protein. 2 TM
102976	7.64	0.16	8.31	0.24	8.91	0.25	1.588 up	2.411 up	0.01	20.09	unknown protein
102985	12.37	0.07	11.48	0.08	10.68	0.15	1.861 down	3.234 down	0.00	126.08	unknown protein
102999	6.79	0.09	9.41	0.12	10.31	0.39	6.126 up	11.491 up	0.00	85.82	unknown protein
103015	10.32	0.11	7.17	0.03	6.94	0.26	8.856 down	10.365 down	0.00	194.28	unknown protein
103016	2.73	0.10	2.65	0.12	4.16	0.13	1.057 down	2.695 up	0.00	144.94	unique protein
103032	3.25	0.11	3.53	0.14	5.02	0.16	1.208 up	3.405 up	0.00	125.26	unknown protein
103034	8.45	0.31	6.64	0.06	5.17	0.12	3.507 down	9.746 down	0.00	251.03	Zn2Cys6 transcriptional regulator
103041	5.10	0.69	2.83	0.26	7.73	0.63	4.814 down	6.212 up	0.00	48.24	acetamidase
103043	2.20	0.01	2.34	0.10	3.79	0.10	1.105 up	3.003 up	0.00	293.51	unknown protein
103044	3.35	0.08	3.55	0.38	4.83	0.32	1.148 up	2.795 up	0.01	21.05	unique protein

103049	5.31	0.24	4.06	0.41	6.62	0.24	2.382 down	2.468 up	0.00	56.32	GH28 endo-polygalacturonase
103059	6.74	0.05	5.70	0.06	7.76	0.43	2.053 down	2.031 up	0.01	25.68	unknown protein
103061	4.04	0.42	4.41	0.01	6.10	0.64	1.298 up	4.189 up	0.02	12.75	unknown protein
103063	6.61	0.16	5.82	0.40	8.55	0.27	1.738 down	3.827 up	0.00	70.38	unknown protein
103064	8.77	0.04	6.06	0.00	10.08	0.24	6.552 down	2.463 up	0.00	297.95	unknown protein
103065	6.67	0.06	7.14	0.23	8.29	0.32	1.378 up	3.061 up	0.01	27.28	unknown protein
103073	5.68	0.00	5.96	0.13	6.71	0.22	1.205 up	2.041 up	0.01	25.16	unknown protein
103112	6.88	0.01	8.23	0.09	8.92	0.27	2.549 up	4.117 up	0.00	63.11	unknown protein
103114	3.75	0.00	3.86	0.24	5.39	0.29	1.079 up	3.106 up	0.00	39.63	unknown protein
103119	4.08	0.49	4.24	0.28	6.09	0.16	1.115 up	4.041 up	0.00	47.34	membrane dipeptidase GliJ
103121	3.26	0.21	3.52	0.00	5.27	0.34	1.200 up	4.041 up	0.00	45.63	unique protein
103122	2.80	0.14	2.79	0.04	4.66	0.14	1.000 down	3.653 up	0.00	213.92	Zn2Cys6 transcriptional regulator
103129	4.98	0.24	5.13	0.43	6.21	0.20	1.111 up	2.343 up	0.01	18.78	unknown protein
103130	4.19	0.10	4.59	0.03	5.60	0.19	1.315 up	2.650 up	0.00	65.57	unique protein
103135	4.57	0.31	4.67	0.21	8.59	1.65	1.070 up	16.190 up	0.03	9.45	SSCRP
103136	3.54	0.10	4.23	0.10	4.83	0.11	1.614 up	2.455 up	0.00	107.96	Glycolate oxidase
103138	10.95	0.08	10.91	0.02	9.65	0.24	1.029 down	2.460 down	0.00	44.63	Zn2Cys6 transcriptional regulator
103145	10.29	0.18	8.54	0.05	8.48	0.31	3.357 down	3.491 down	0.00	36.17	unique protein
103147	14.36	0.03	13.55	0.10	11.78	0.35	1.749 down	5.977 down	0.00	66.35	unknown protein
103149	10.91	0.07	6.74	0.01	8.20	0.18	18.085 down	6.549 down	0.00	460.98	AAA ATPase
103155	3.21	0.06	3.42	0.30	5.07	0.22	1.153 up	3.630 up	0.00	62.78	unknown protein
103156	8.28	0.05	3.98	0.21	6.60	0.18	19.725 down	3.204 down	0.00	341.30	unknown protein
103157	11.16	0.00	11.19	0.18	8.88	0.51	1.024 up	4.853 down	0.00	32.63	unknown protein
103158	9.69	0.07	8.31	0.07	6.75	0.35	2.610 down	7.664 down	0.00	78.58	Zn2Cys6 transcriptional regulator
103172	7.16	0.15	4.60	0.01	6.14	0.20	5.905 down	2.021 down	0.00	113.95	unique protein
103174	3.05	0.09	3.06	0.11	5.05	0.15	1.007 up	4.014 up	0.00	226.43	SSCRP
103175	3.14	0.12	3.70	0.22	4.99	0.17	1.476 up	3.592 up	0.00	89.00	epoxide hydrolase. putative
103176	4.38	0.33	4.39	0.18	5.73	0.24	1.004 up	2.534 up	0.00	29.14	unknown protein
103177	4.36	0.33	5.41	0.11	5.62	0.24	2.072 up	2.395 up	0.01	18.51	glutathionine S-transferase
103186	4.26	0.17	3.81	0.29	5.34	0.17	1.367 down	2.113 up	0.00	45.58	unknown protein

103189	6.82	0.20	5.20	0.05	5.45	0.32	3.073 down	2.586 down	0.01	23.09	HET protein
103193	8.14	0.16	4.80	0.55	5.30	0.18	10.151 down	7.186 down	0.00	81.59	ankyrin
103205	6.72	0.05	9.04	0.38	8.97	0.68	5.009 up	4.748 up	0.02	12.54	unknown protein
103215	11.54	0.20	11.06	0.05	9.92	0.09	1.392 down	3.073 down	0.00	159.13	Cytochrome P450 CYP4/CYP19/CYP26 subfamilies
103222	12.94	0.12	13.54	0.10	14.12	0.16	1.518 up	2.257 up	0.00	45.27	Ribosomal protein S29 (S14 family) by homology to the corresponding protein of N. cr
103230	11.42	0.05	11.25	0.08	9.16	0.22	1.127 down	4.801 down	0.00	154.68	Zn2Cys6 transcriptional regulator
103236	9.66	0.21	9.01	0.01	11.13	0.12	1.562 down	2.773 up	0.00	195.76	unknown protein
103286	2.54	0.08	2.92	0.43	4.77	0.23	1.300 up	4.691 up	0.00	60.77	unique protein
103335	7.60	0.31	7.95	0.15	9.98	0.57	1.274 up	5.229 up	0.01	22.59	unknown protein
103356	13.29	0.01	13.12	0.07	12.17	0.16	1.124 down	2.175 down	0.00	67.08	Vacuolar transporter chaperone
103372	12.47	0.09	11.75	0.01	10.97	0.24	1.646 down	2.811 down	0.00	44.07	BZIP transcriptional regulator
103394	3.38	0.24	3.27	0.10	5.06	0.16	1.079 down	3.209 up	0.00	103.46	unknown protein
103395	3.14	0.27	3.15	0.05	4.77	0.26	1.010 up	3.104 up	0.00	47.50	unknown protein
103403	3.53	0.11	3.90	0.45	5.19	0.19	1.289 up	3.154 up	0.00	34.59	unknown protein
103423	5.98	0.19	5.85	0.09	7.37	0.44	1.092 down	2.619 up	0.01	17.14	SSCRP
103447	9.47	0.12	9.61	0.04	8.26	0.30	1.104 up	2.307 down	0.00	29.21	unknown protein
103455	9.41	0.01	11.00	0.01	11.16	0.24	3.006 up	3.367 up	0.00	63.20	unknown protein
103482	11.47	0.00	10.68	0.07	9.27	0.38	1.723 down	4.604 down	0.00	40.21	transcriptional activator with ariadne RING finger
103506	12.73	0.01	11.92	0.24	11.59	0.23	1.748 down	2.197 down	0.01	20.11	unknown protein
103537	6.21	0.19	10.65	0.01	10.18	0.33	21.752 up	15.724 up	0.00	182.26	GCN5-N-acetyltransferase
103651	3.33	0.24	3.73	0.23	4.86	0.12	1.319 up	2.880 up	0.00	58.96	unknown protein
103653	2.15	0.10	2.09	0.02	3.47	0.07	1.046 down	2.489 up	0.00	379.26	unique protein
103655	8.44	0.06	9.17	0.04	9.83	0.14	1.654 up	2.620 up	0.00	109.18	rad55
103668	9.95	0.07	9.52	0.02	8.63	0.56	1.348 down	2.508 down	0.05	7.11	unique protein
103671	11.33	0.08	10.65	0.03	9.40	0.33	1.602 down	3.810 down	0.00	42.11	unknown protein
103683	6.18	0.35	6.91	0.04	5.13	0.11	1.654 up	2.075 down	0.00	73.27	unique protein
103694	11.90	0.14	11.72	0.14	9.26	0.46	1.135 down	6.223 down	0.00	48.04	PTH11 GPCR
103715	5.65	0.21	6.10	0.17	7.62	0.41	1.364 up	3.923 up	0.01	27.50	unique protein
103778	3.65	0.07	3.59	0.07	6.40	0.40	1.045 down	6.708 up	0.00	79.82	unique protein
103798	11.55	0.16	10.22	0.02	9.72	0.32	2.520 down	3.567 down	0.00	33.46	SSCRP

103799	10.91	0.01	10.32	0.15	9.40	0.17	1.497 down	2.834 down	0.00	76.07	unique protein
103804	12.60	0.17	12.22	0.15	11.32	0.22	1.300 down	2.422 down	0.00	31.56	SWI-SNF chromatin-remodeling complex protein
103852	11.98	0.04	12.06	0.03	10.78	0.23	1.057 up	2.304 down	0.00	49.70	unknown protein
103863	4.35	0.08	4.64	0.02	5.84	0.32	1.224 up	2.825 up	0.00	30.71	unknown protein
103886	14.40	0.24	14.27	0.16	12.95	0.14	1.097 down	2.746 down	0.00	66.66	unknown protein
103898	8.04	0.16	9.09	0.30	9.25	0.31	2.071 up	2.304 up	0.02	12.59	unknown protein
103900	3.16	0.15	3.22	0.19	4.96	0.09	1.039 up	3.474 up	0.00	191.33	unknown protein
103907	6.95	0.30	7.42	0.00	5.06	0.61	1.380 up	3.721 down	0.01	19.15	unknown protein
103930	12.39	0.05	12.06	0.00	10.94	0.39	1.251 down	2.725 down	0.01	18.13	unknown protein
103949	7.71	0.11	7.94	0.15	8.96	0.35	1.170 up	2.376 up	0.01	16.06	unknown protein
103959	3.26	0.21	3.16	0.08	4.72	0.12	1.069 down	2.748 up	0.00	123.91	unique protein
103960	8.69	0.12	7.45	0.08	7.05	0.25	2.353 down	3.107 down	0.00	43.86	nitrilase
103973	5.61	0.50	6.10	0.21	7.84	0.59	1.398 up	4.665 up	0.01	15.14	unknown protein
104000	10.69	0.14	11.63	0.12	11.80	0.12	1.918 up	2.158 up	0.00	53.59	DNA repair protein rad18
104016	11.64	0.10	11.81	0.05	10.59	0.12	1.120 up	2.070 down	0.00	120.11	unknown protein
104046	3.28	0.14	3.67	0.43	4.79	0.11	1.311 up	2.853 up	0.00	37.43	unique protein
104050	4.20	0.35	3.56	0.41	8.09	0.98	1.559 down	14.834 up	0.01	28.16	SSCRP
104054	3.82	0.03	3.88	0.14	5.72	0.24	1.043 up	3.720 up	0.00	88.32	unknown protein
104059	3.45	0.37	3.36	0.28	5.30	0.15	1.067 down	3.602 up	0.00	65.02	short chain dehydrognease/reductase
104060	3.25	0.38	2.99	0.20	4.97	0.32	1.197 down	3.301 up	0.00	35.54	short chain dehydrognease/reductase
104064	3.52	0.29	3.44	0.13	5.39	0.42	1.057 down	3.660 up	0.00	29.44	unknown protein
104067	3.70	0.01	3.48	0.11	6.35	0.61	1.162 down	6.266 up	0.00	33.90	unknown protein
104071	4.47	0.05	4.44	0.39	5.83	0.28	1.019 down	2.566 up	0.01	23.71	unknown protein
104075	10.93	0.22	10.65	0.22	9.52	0.41	1.213 down	2.653 down	0.02	13.92	Zn2Cys6 transcriptional regulator
104077	10.78	0.02	10.41	0.06	7.17	0.92	1.291 down	12.210 down	0.01	23.05	Amino acid transporter PotE
104079	4.72	0.22	4.56	0.09	6.57	0.32	1.112 down	3.604 up	0.00	51.52	MRSP1/expansin-like
104081	5.55	0.13	5.51	0.20	7.12	0.73	1.026 down	2.959 up	0.04	7.62	coenzyme F420-dependent N5.N10-methylene tetrahydromethanopterin reductase
104106	4.16	0.11	4.05	0.14	5.47	0.16	1.073 down	2.488 up	0.00	90.42	ADP/ATP carrier protein
104109	4.44	0.13	4.60	0.02	5.66	0.20	1.121 up	2.331 up	0.00	47.52	unique protein
104118	3.27	0.25	3.48	0.27	5.81	0.44	1.156 up	5.813 up	0.00	41.37	unique protein

104140	9.55	0.01	10.93	0.18	10.91	0.27	2.606 up	2.557 up	0.01	26.90	3-hydroxi-isobutyrate dehydrogenase
104157	3.30	0.00	3.27	0.36	4.49	0.05	1.015 down	2.282 up	0.00	53.68	unique protein
104168	6.42	0.07	7.75	0.00	7.55	0.35	2.519 up	2.197 up	0.01	14.76	unknown protein
104173	5.53	0.02	4.90	0.05	6.84	0.21	1.554 down	2.475 up	0.00	103.48	unique protein
104175	4.33	0.13	4.61	0.13	5.71	0.15	1.211 up	2.594 up	0.00	79.90	unique protein
104180	9.55	0.14	6.27	0.46	8.29	0.13	9.686 down	2.395 down	0.00	97.31	Ankyrin
104181	4.71	0.11	6.34	0.14	6.93	0.26	3.106 up	4.664 up	0.00	69.97	SSCRP
104182	11.47	0.06	10.30	0.04	8.47	0.34	2.242 down	7.991 down	0.00	94.62	Zn2Cys6 transcriptional regulator
104200	6.26	0.08	8.24	0.32	8.67	0.47	3.939 up	5.335 up	0.01	26.30	unknown protein. F-box
104201	2.98	0.09	2.90	0.04	4.36	0.20	1.064 down	2.585 up	0.00	80.18	unknown protein
104206	3.67	0.03	3.95	0.31	4.69	0.21	1.211 up	2.035 up	0.01	18.00	SSCRP
104209	3.76	0.11	3.96	0.16	5.57	0.32	1.146 up	3.511 up	0.00	43.07	unknown protein
104211	8.37	0.09	4.41	0.21	12.97	0.28	15.509 down	24.344 up	0.00	883.18	FKBP-type peptidyl-prolyl cis-trans isomerase
104215	8.09	0.05	7.55	0.04	6.38	0.07	1.454 down	3.289 down	0.00	537.69	unknown protein
104227	4.10	0.27	3.94	0.25	8.49	1.38	1.120 down	21.005 up	0.01	17.09	SSCRP
104228	2.54	0.26	2.48	0.08	3.98	0.16	1.048 down	2.701 up	0.00	71.49	unique protein
104231	12.70	0.11	12.57	0.08	11.63	0.10	1.087 down	2.094 down	0.00	109.37	unknown protein
104260	5.98	0.21	6.37	0.06	7.87	0.28	1.303 up	3.683 up	0.00	51.47	unknown protein
104261	6.25	0.13	6.65	0.23	8.83	0.41	1.323 up	6.012 up	0.00	50.30	GCN5-related N-acetyltransferase
104276	9.57	0.08	9.91	0.22	8.16	0.20	1.269 up	2.652 down	0.00	72.16	unknown protein
104277	13.33	0.32	12.37	0.05	11.80	0.47	1.942 down	2.882 down	0.03	10.27	cell wall protein. distantly related to A. niger CwpA.
104286	3.33	0.16	3.42	0.10	4.84	0.13	1.065 up	2.845 up	0.00	121.82	unique protein
104289	4.34	0.06	4.27	0.07	5.67	0.14	1.048 down	2.528 up	0.00	145.74	unique protein
104292	7.50	0.09	8.35	0.01	8.77	0.42	1.803 up	2.411 up	0.03	10.22	mating type pheromone precursor. alpha type
104293	5.98	0.54	6.49	0.48	8.14	0.21	1.430 up	4.472 up	0.01	28.34	HFB5
104294	4.48	0.47	4.76	0.24	6.93	0.20	1.215 up	5.465 up	0.00	67.34	unknown protein
104295	7.24	0.16	4.88	0.32	9.76	0.67	5.144 down	5.720 up	0.00	56.23	unknown protein
104304	10.28	0.15	9.01	0.06	8.34	0.25	2.406 down	3.822 down	0.00	57.79	unknown protein
104334	13.09	0.07	12.88	0.07	12.04	0.32	1.156 down	2.076 down	0.02	14.20	unknown protein
104335	5.40	0.42	5.73	0.00	6.56	0.15	1.256 up	2.224 up	0.01	21.62	unknown protein

104336	3.54	0.33	3.13	0.09	5.14	0.15	1.324 down	3.040 up	0.00	91.88	unique protein
104351	6.35	0.01	7.63	0.05	7.87	0.29	2.431 up	2.871 up	0.00	30.81	unique protein
104353	4.64	0.02	5.36	0.33	5.78	0.29	1.651 up	2.210 up	0.02	11.91	unknown protein. contains F-box
104354	3.27	0.05	3.34	0.31	4.70	0.27	1.047 up	2.685 up	0.00	30.58	SSCRP
104368	5.69	0.50	5.89	0.28	7.64	0.40	1.153 up	3.884 up	0.01	21.47	unknown protein
104369	3.78	0.12	3.70	0.22	5.23	0.15	1.052 down	2.744 up	0.00	85.25	unknown protein
104373	2.93	0.12	3.22	0.09	4.96	0.20	1.217 up	4.060 up	0.00	124.78	MRSP1/expansin-like
104377	11.82	0.02	11.56	0.05	9.53	0.31	1.198 down	4.890 down	0.00	79.65	unknown protein
104380	8.36	0.13	7.27	0.00	5.84	0.30	2.130 down	5.726 down	0.00	79.63	Zn2Cys6 transcriptional regulator
104390	9.31	0.04	11.22	0.31	7.95	0.26	3.773 up	2.563 down	0.00	121.61	glutathione S-transferase
104395	10.21	0.00	10.35	0.03	8.77	0.27	1.098 up	2.721 down	0.00	51.16	unknown protein
104399	2.50	0.00	2.70	0.05	3.93	0.06	1.149 up	2.696 up	0.00	634.28	unique protein
104419	3.89	0.25	3.77	0.37	5.94	0.42	1.090 down	4.138 up	0.00	30.44	unique protein. secreted
104421	2.93	0.07	3.22	0.17	4.97	0.10	1.220 up	4.106 up	0.00	270.16	unknown protein
104422	3.22	0.13	2.73	0.10	6.41	0.62	1.407 down	9.137 up	0.00	50.18	unknown protein
104423	5.10	0.35	4.38	0.09	7.12	0.35	1.648 down	4.053 up	0.00	58.58	MRSP1/expansin-like
104437	11.49	0.15	13.25	0.05	12.81	0.11	3.382 up	2.494 up	0.00	140.96	unknown protein
104490	9.80	0.14	10.21	0.24	11.54	0.32	1.325 up	3.345 up	0.00	31.37	v-SNARE Gos1. Golgi transport
104501	2.41	0.11	2.56	0.06	4.10	0.14	1.112 up	3.236 up	0.00	166.92	unique protein
104511	8.20	0.03	8.12	0.06	9.36	0.23	1.060 down	2.225 up	0.00	43.91	unique protein
104513	10.62	0.07	10.37	0.12	9.52	0.17	1.188 down	2.135 down	0.00	46.75	Zn2Cys6 transcriptional regulator
104533	2.45	0.10	2.41	0.07	4.12	0.11	1.025 down	3.190 up	0.00	293.98	unique protein
104556	5.79	0.15	6.08	0.09	9.39	0.54	1.223 up	12.129 up	0.00	67.35	unknown protein
104557	10.32	0.08	7.52	0.14	8.49	0.06	6.959 down	3.565 down	0.00	553.15	short-chain dehydrogenase/reductase
104576	2.20	0.07	2.23	0.08	3.67	0.03	1.023 up	2.780 up	0.00	676.14	unique protein
104577	3.77	0.20	3.91	0.06	5.19	0.07	1.095 up	2.664 up	0.00	155.81	unique protein
104585	6.26	0.11	6.14	0.02	7.45	0.38	1.082 down	2.283 up	0.01	17.25	unknown protein
104592	3.89	0.30	3.76	0.34	5.36	0.15	1.093 down	2.779 up	0.00	43.21	unknown protein
104593	6.60	0.13	5.05	0.25	5.06	0.11	2.936 down	2.909 down	0.00	77.80	unknown protein with ankyrin. Leu-zipper and WD40
104595	10.68	0.08	10.86	0.06	11.81	0.36	1.134 up	2.185 up	0.02	14.04	Ribosomal protein L7Ae/L30e/S12e/Gadd45. Homologue of yeast SNU13.

104599	10.45	0.08	8.79	0.13	8.00	0.22	3.147 down	5.473 down	0.00	117.20	Mandelate racemase/muconate lactonizing enzyme
104601	8.23	0.13	8.36	0.08	7.09	0.38	1.094 up	2.201 down	0.01	15.95	unknown protein
104617	12.45	0.04	12.17	0.13	11.03	0.14	1.218 down	2.670 down	0.00	105.00	
104695	4.37	0.05	3.59	0.32	5.53	0.36	1.708 down	2.243 up	0.01	27.90	unique protein
104715	2.47	0.16	2.46	0.15	4.01	0.07	1.005 down	2.905 up	0.00	192.79	unique protein
104716	3.01	0.11	3.12	0.38	5.01	0.10	1.076 up	3.999 up	0.00	100.42	unique protein
104741	11.06	0.09	10.13	0.04	8.93	0.25	1.902 down	4.389 down	0.00	78.30	unknown protein
104744	11.34	0.11	11.49	0.15	12.53	0.41	1.111 up	2.282 up	0.02	11.59	cytochrome c oxidase assembly protein COX19. putative
104762	7.57	0.09	8.36	0.14	8.60	0.11	1.727 up	2.041 up	0.00	53.53	unknown protein
104795	7.58	0.18	8.07	0.10	9.28	0.12	1.407 up	3.245 up	0.00	128.94	unique protein
104820	9.48	0.04	9.02	0.03	8.43	0.41	1.371 down	2.073 down	0.04	7.86	unknown protein
104842	11.37	0.23	11.36	0.21	10.20	0.28	1.005 down	2.241 down	0.01	19.95	transthyretin-like protein
104911	11.79	0.01	11.65	0.00	10.72	0.14	1.099 down	2.097 down	0.00	87.85	unknown protein
104923	3.28	0.18	3.38	0.00	4.91	0.22	1.072 up	3.090 up	0.00	69.98	unique protein
104925	8.63	0.00	9.43	0.03	7.14	0.35	1.743 up	2.802 down	0.00	52.00	unknown protein
104927	7.68	0.16	9.46	0.11	9.73	0.39	3.427 up	4.123 up	0.00	29.55	unique protein
104968	12.02	0.11	11.73	0.07	10.55	0.10	1.229 down	2.768 down	0.00	188.37	unknown protein
104972	13.65	0.12	13.24	0.16	12.59	0.29	1.330 down	2.083 down	0.02	13.85	glutathione synthase
105003	11.25	0.04	8.94	0.16	9.21	0.28	4.968 down	4.113 down	0.00	66.87	unknown protein
105027	5.23	0.27	5.36	0.11	6.39	0.15	1.093 up	2.231 up	0.00	38.47	unknown protein
105106	12.96	0.02	13.28	0.03	10.36	0.42	1.255 up	6.057 down	0.00	73.35	unique protein
105133	5.90	0.02	6.45	0.08	7.06	0.23	1.460 up	2.238 up	0.01	28.04	unknown protein
105143	9.41	0.20	9.81	0.08	11.95	0.49	1.323 up	5.834 up	0.00	36.53	unique protein
105154	4.59	0.03	4.59	0.02	6.05	0.53	1.002 down	2.745 up	0.02	12.46	unknown protein
105155	4.21	0.24	4.63	0.33	6.28	0.16	1.334 up	4.191 up	0.00	72.04	unknown protein
105156	3.52	0.40	3.57	0.11	6.94	0.73	1.034 up	10.673 up	0.00	32.41	unique protein
105157	3.07	0.02	3.09	0.08	5.23	0.10	1.011 up	4.445 up	0.00	651.12	unknown protein
105158	7.00	0.06	7.48	0.20	5.78	0.10	1.390 up	2.330 down	0.00	155.15	unknown protein
105167	3.43	0.21	3.59	0.28	4.93	0.09	1.123 up	2.845 up	0.00	68.27	unique protein
105171	3.65	0.23	3.68	0.15	5.81	0.16	1.023 up	4.463 up	0.00	145.59	unknown protein

105173	2.62	0.15	2.73	0.24	4.38	0.21	1.076 up	3.365 up	0.00	69.68	unknown protein
105187	6.56	0.15	6.38	0.02	8.52	0.36	1.130 down	3.887 up	0.00	50.55	unknown protein
105206	4.55	0.31	5.03	0.00	6.34	0.36	1.399 up	3.470 up	0.01	25.91	unique protein
105220	6.40	0.39	3.96	0.35	4.59	0.09	5.424 down	3.525 down	0.00	55.86	unknown protein
105221	3.70	0.31	2.98	0.41	4.72	0.05	1.650 down	2.028 up	0.00	40.61	unknown protein
105222	3.84	0.09	3.95	0.15	5.71	0.34	1.081 up	3.648 up	0.00	43.69	unique protein
105223	8.62	0.06	8.38	0.26	6.42	0.24	1.179 down	4.591 down	0.00	87.86	unknown protein
105233	2.86	0.01	2.69	0.06	4.34	0.03	1.129 down	2.785 up	0.00	2054.93	unique protein
105239	11.56	0.15	11.34	0.13	9.98	0.14	1.161 down	2.977 down	0.00	110.89	Zn2Cys6 transcriptional regulator
105246	3.77	0.13	4.35	0.31	5.70	0.18	1.488 up	3.805 up	0.00	67.49	GH23 exo-ß-1.3-glucanase. distantly related
105247	3.19	0.23	3.44	0.17	5.19	0.06	1.196 up	4.006 up	0.00	188.44	dipeptidyl peptidase 5
105251	9.70	0.07	9.46	0.06	8.26	0.30	1.185 down	2.724 down	0.00	31.24	unknown protein
105255	9.46	0.15	8.77	0.14	7.05	0.34	1.609 down	5.298 down	0.00	57.77	Zn2Cys6 transcriptional regulator
105260	11.56	0.08	10.04	0.26	7.90	0.47	2.886 down	12.720 down	0.00	64.07	MFS permease
105263	11.69	0.19	11.24	0.00	10.00	0.12	1.364 down	3.222 down	0.00	146.52	Zn2Cys6 transcriptional regulator
105269	9.61	0.10	8.88	0.04	8.60	0.19	1.661 down	2.011 down	0.00	28.97	Zn2Cys6 transcriptional regulator
105279	4.80	0.34	3.90	0.10	5.98	0.71	1.861 down	2.277 up	0.03	9.49	Leucine aminopeptidase 1
105286	4.55	0.12	4.22	0.23	5.90	0.14	1.261 down	2.546 up	0.00	90.36	unknown protein
105290	5.58	0.15	4.88	0.43	6.81	0.53	1.631 down	2.336 up	0.02	13.16	unknown protein
105307	9.30	0.01	10.03	0.25	10.90	0.59	1.649 up	3.013 up	0.04	7.90	40s mitochodrial ribosome subunit protein Mrp17
105311	10.60	0.13	11.26	0.21	12.63	0.30	1.576 up	4.079 up	0.00	47.62	SSCRP
105330	5.23	0.38	4.48	0.06	7.23	0.47	1.687 down	3.985 up	0.00	36.96	unknown protein
105336	5.04	0.35	5.32	0.04	6.47	0.33	1.210 up	2.696 up	0.01	19.01	unknown protein
105338	3.00	0.24	3.26	0.21	5.23	0.28	1.199 up	4.693 up	0.00	66.45	unknown protein
105363	11.53	0.05	9.03	0.19	8.69	0.56	5.675 down	7.165 down	0.00	29.16	aldolase/citrate lyase family protein
105381	10.01	0.22	10.41	0.22	11.22	0.32	1.324 up	2.320 up	0.02	13.97	unknown protein
105383	3.58	0.00	3.80	0.44	5.58	0.26	1.162 up	3.994 up	0.00	44.75	unknown protein
105385	4.14	0.04	4.10	0.34	5.44	0.18	1.030 down	2.463 up	0.00	40.31	unknown protein
105386	12.27	0.12	12.15	0.11	10.81	0.23	1.081 down	2.736 down	0.00	54.56	unknown protein
105391	2.84	0.02	3.09	0.03	4.98	0.12	1.189 up	4.408 up	0.00	435.83	unknown protein

105393	6.37	0.06	5.78	0.08	7.87	0.48	1.500 down	2.832 up	0.01	23.89	unknown protein
105405	3.12	0.07	2.89	0.09	4.70	0.29	1.174 down	2.990 up	0.00	55.05	unique protein
105444	5.47	0.21	4.84	0.21	6.88	0.30	1.549 down	2.658 up	0.00	44.05	SSCRP
105445	2.81	0.05	2.88	0.02	4.45	0.13	1.048 up	3.096 up	0.00	250.64	unknown protein. only in Neurospora and A. fumigatus
105447	4.99	0.20	4.89	0.19	7.02	0.33	1.074 down	4.082 up	0.00	53.72	unknown protein
105448	3.29	0.22	3.57	0.27	5.80	0.40	1.215 up	5.686 up	0.00	47.65	CBM 13
105449	3.27	0.16	3.30	0.11	5.16	0.29	1.020 up	3.699 up	0.00	61.32	Cyclin C-dependent kinase CDK8
105455	8.79	0.04	7.04	0.24	7.48	0.37	3.385 down	2.493 down	0.01	18.56	unknown protein
105457	3.13	0.02	3.38	0.12	4.56	0.11	1.195 up	2.697 up	0.00	175.60	unknown protein
105467	3.40	0.29	3.47	0.16	5.23	0.10	1.053 up	3.546 up	0.00	115.97	unknown protein
105488	6.17	0.07	6.39	0.23	7.24	0.05	1.162 up	2.099 up	0.00	75.24	unknown protein
105514	11.01	0.12	6.45	0.03	9.52	0.66	23.554 down	2.801 down	0.00	41.87	unique protein
105515	11.50	0.17	13.05	0.11	13.90	0.13	2.915 up	5.259 up	0.00	216.53	unique protein
105518	11.91	0.04	10.56	0.10	9.27	0.16	2.552 down	6.245 down	0.00	260.59	K+ channel
105532	6.28	0.04	5.52	0.13	7.50	0.59	1.695 down	2.322 up	0.02	13.17	unique protein
105537	7.11	0.10	7.18	0.02	6.03	0.16	1.051 up	2.108 down	0.00	72.40	unknown protein
105540	5.25	0.28	5.20	0.24	6.50	0.40	1.033 down	2.368 up	0.02	12.82	unique protein
105553	6.67	0.57	7.12	0.27	9.76	0.69	1.363 up	8.518 up	0.01	22.60	unique protein
105556	3.59	0.01	4.13	0.23	5.67	0.14	1.445 up	4.205 up	0.00	157.22	unique protein
105597	11.82	0.04	11.96	0.12	10.43	0.26	1.101 up	2.615 down	0.00	50.62	unique protein
105643	12.01	0.07	11.83	0.01	10.59	0.20	1.135 down	2.688 down	0.00	70.93	myb transcriptional regulator
105652	3.74	0.15	4.09	0.06	5.51	0.06	1.273 up	3.408 up	0.00	339.50	unknown protein
105676	8.69	0.02	9.86	0.11	9.89	0.18	2.252 up	2.303 up	0.00	50.14	DNA replication licensing factor mcm7
105707	2.78	0.54	2.88	0.38	4.44	0.20	1.072 up	3.169 up	0.01	23.67	unknown protein
105718	11.10	0.22	10.12	0.09	8.21	0.29	1.979 down	7.431 down	0.00	104.61	unknown protein
105722	10.76	0.00	6.64	0.22	8.75	0.51	17.360 down	4.027 down	0.00	51.85	unique protein
105729	3.13	0.38	3.15	0.31	4.60	0.06	1.012 up	2.767 up	0.00	41.75	unknown protein
105752	2.80	0.14	2.82	0.04	6.30	1.06	1.018 up	11.330 up	0.01	18.05	C4-dicarboxylate transporter/malic acid transport protein
105763	10.20	0.24	10.93	0.14	8.12	0.26	1.649 up	4.233 down	0.00	114.04	HFBs
105765	11.39	0.11	11.41	0.13	9.36	0.36	1.009 up	4.102 down	0.00	51.05	unknown protein

105768	4.61	0.12	4.54	0.18	5.67	0.08	1.049 down	2.085 up	0.00	88.18	Cytochrome P450
105775	9.52	0.10	8.18	0.05	8.39	0.39	2.536 down	2.195 down	0.02	11.84	unknown protein
105784	8.10	0.13	7.24	0.19	6.61	0.27	1.815 down	2.811 down	0.01	28.15	Zn2Cys6 transcriptional regulator
105785	4.58	0.09	5.09	0.04	6.72	0.19	1.423 up	4.402 up	0.00	151.49	unknown protein
105798	4.02	0.15	4.18	0.38	5.43	0.10	1.120 up	2.659 up	0.00	46.35	MFS permease
105804	3.59	0.31	3.33	0.43	5.34	0.20	1.193 down	3.352 up	0.00	44.52	PKS
105805	3.32	0.07	3.67	0.42	5.53	0.17	1.272 up	4.632 up	0.00	78.10	Zn2Cys6 transcriptional regulator
105806	3.11	0.22	2.58	0.08	5.03	0.13	1.436 down	3.782 up	0.00	229.23	unknown protein
105820	2.83	0.06	3.14	0.26	4.24	0.16	1.237 up	2.642 up	0.00	54.42	unknown protein
105823	9.62	0.02	4.85	0.30	6.76	0.45	27.139 down	7.263 down	0.00	81.61	FAD binding domain protein
105832	12.66	0.10	12.18	0.10	11.38	0.23	1.403 down	2.434 down	0.00	32.27	phosphoribosylaminoimidazole carbounknown proteinlase
105834	9.00	0.02	9.15	0.28	7.39	0.24	1.110 up	3.049 down	0.00	56.63	Zn2Cys6 transcriptional regulator
105838	4.36	0.28	4.15	0.23	5.51	0.08	1.160 down	2.210 up	0.00	52.87	glyoxalase
105840	9.19	0.00	5.93	0.11	10.24	0.33	9.559 down	2.071 up	0.00	187.59	unknown protein. 2 TMs
105849	9.65	0.05	9.14	0.13	7.46	0.21	1.424 down	4.587 down	0.00	129.79	Zn2Cys6 transcriptional regulator
105851	7.73	0.00	7.43	0.17	6.49	0.48	1.232 down	2.375 down	0.03	8.81	GMC oxidoreductase family protein
105863	10.84	0.07	11.32	0.14	9.63	0.09	1.395 up	2.300 down	0.00	216.55	unknown protein
105866	11.94	0.03	11.76	0.06	10.55	0.12	1.135 down	2.615 down	0.00	177.20	unique protein
105867	3.25	0.38	3.33	0.09	5.25	0.30	1.058 up	3.986 up	0.00	44.82	unique protein
105869	5.76	0.03	6.38	0.17	7.64	0.27	1.536 up	3.676 up	0.00	52.64	HFB6
105874	7.15	0.06	7.22	0.04	5.75	0.18	1.051 up	2.636 down	0.00	105.88	FAD binding domain-containing protein
105884	3.51	0.07	3.67	0.03	5.19	0.19	1.120 up	3.214 up	0.00	109.51	short chain dehydrogenase/reductase
105888	5.67	0.03	6.55	0.30	7.75	0.30	1.847 up	4.222 up	0.00	42.05	short chain dehydrogenase/reductase
105894	5.39	0.45	5.59	0.22	8.56	0.58	1.149 up	9.027 up	0.00	37.04	unknown protein
105912	4.14	0.08	4.02	0.23	5.37	0.24	1.082 down	2.348 up	0.00	35.79	unknown protein
105977	4.17	0.04	4.64	0.09	5.27	0.19	1.379 up	2.139 up	0.00	35.64	unknown secreted protein. 3 TM
105978	9.14	0.04	9.88	0.14	8.14	0.24	1.676 up	2.003 down	0.00	57.74	ARO8. Transcriptional regulators containing a DNA-binding HTH domain and an amino
105983	3.44	0.09	3.56	0.39	4.87	0.11	1.085 up	2.706 up	0.00	48.02	unknown protein
105989	4.03	0.11	5.44	0.29	5.40	0.17	2.653 up	2.578 up	0.00	40.65	Zn2Cys6 transcriptional regulator
106009	11.46	0.12	11.28	0.18	10.42	0.23	1.133 down	2.063 down	0.01	23.03	Zn2Cys6 transcriptional regulator

106018	10.86	0.17	11.93	0.00	12.07	0.05	2.107 up	2.312 up	0.00	133.36	unknown protein
106024	7.73	0.25	8.24	0.16	9.68	0.63	1.429 up	3.875 up	0.02	11.80	unknown protein
106029	11.58	0.04	10.58	0.07	9.37	0.16	1.994 down	4.616 down	0.00	203.95	MFS permease
106041	3.37	0.00	3.65	0.14	5.00	0.16	1.216 up	3.105 up	0.00	120.31	unique protein
106043	10.09	0.06	8.15	0.07	8.00	0.25	3.842 down	4.265 down	0.00	79.56	unknown protein. GFA-domain
106067	9.56	0.72	8.94	0.11	11.16	0.22	1.540 down	3.028 up	0.01	28.11	Chaperonin Cpn10
106082	11.93	0.00	11.49	0.06	10.44	0.15	1.358 down	2.813 down	0.00	126.56	Epl1/Sm1
106089	8.48	0.01	7.61	0.14	6.13	0.18	1.823 down	5.089 down	0.00	169.85	unique protein
106107	5.31	0.09	5.83	0.23	6.65	0.10	1.429 up	2.523 up	0.00	68.42	unknown protein
106116	5.08	0.17	6.23	0.01	6.81	0.67	2.227 up	3.324 up	0.05	7.36	unknown protein
106118	10.89	0.04	10.61	0.16	9.58	0.13	1.221 down	2.479 down	0.00	92.34	Inorganic phosphate transporter
106120	8.56	0.06	12.87	0.03	12.32	0.33	19.812 up	13.457 up	0.00	181.01	esterase/lipase
106129	11.55	0.05	10.92	0.11	9.73	0.38	1.554 down	3.547 down	0.01	28.27	unknown protein. only present in ascomycota
106130	13.95	0.02	13.52	0.08	12.44	0.12	1.346 down	2.843 down	0.00	187.86	unknown protein
106138	8.36	0.00	7.30	0.01	6.61	0.32	2.081 down	3.340 down	0.00	32.81	SNF2 family DNA-dependent ATPase
106151	3.53	0.30	3.71	0.06	6.47	0.27	1.136 up	7.677 up	0.00	127.89	unknown protein
106152	5.09	0.02	5.67	0.16	6.15	0.19	1.488 up	2.079 up	0.01	27.98	unknown protein
106154	6.09	0.24	6.55	0.11	4.66	0.04	1.368 up	2.699 down	0.00	189.85	unknown protein
106164	10.81	0.00	8.06	0.07	12.21	0.13	6.740 down	2.623 up	0.00	962.65	short chain dehydrogenase/reductase
106168	4.76	0.12	5.17	0.04	6.53	0.33	1.325 up	3.414 up	0.00	37.64	SSCRP
106171	10.23	0.14	8.73	0.26	11.92	1.06	2.821 down	3.232 up	0.03	10.31	HET protein
106181	11.39	0.07	11.73	0.04	7.85	0.72	1.265 up	11.629 down	0.00	44.19	unique protein
106219	7.71	0.26	8.41	0.06	9.30	0.21	1.617 up	3.010 up	0.00	42.88	unknown protein
106223	12.86	0.05	11.45	0.20	10.26	0.26	2.658 down	6.040 down	0.00	92.59	unique protein
106234	12.55	0.08	11.75	0.16	11.53	0.18	1.739 down	2.030 down	0.01	28.00	integral membrane protein comprising an SPX and an EXS domain. therefore most pro
106242	9.79	0.12	9.28	0.11	8.52	0.19	1.424 down	2.404 down	0.00	43.38	unique protein
106245	7.80	0.08	7.06	0.04	5.75	0.36	1.665 down	4.117 down	0.00	38.52	catalase
106248	11.09	0.04	11.85	0.01	12.91	0.14	1.696 up	3.534 up	0.00	192.30	monosaccharide transporter (galactose permease ?)
106267	4.25	0.03	4.36	0.24	5.85	0.21	1.081 up	3.031 up	0.00	61.55	unique protein
106270	10.85	0.14	5.32	0.53	8.08	1.82	46.214 down	6.817 down	0.05	7.44	unknown protein

106272	8.37	0.12	7.82	0.01	7.00	0.30	1.455 down	2.574 down	0.01	24.28	PKS
106276	12.71	0.11	12.59	0.06	11.06	0.20	1.087 down	3.132 down	0.00	93.44	initiator tRNA phosphoribosyl transferase.
106297	10.92	0.07	11.40	0.02	9.89	0.26	1.393 up	2.036 down	0.00	39.86	Amino acid transporters
106314	8.84	0.01	9.22	0.03	6.11	0.20	1.306 up	6.595 down	0.00	351.56	Ankyrin
106315	7.19	0.33	8.37	0.04	9.20	0.31	2.271 up	4.033 up	0.00	33.69	serine protease
106356	12.26	0.12	11.94	0.05	11.12	0.33	1.240 down	2.196 down	0.01	14.88	unknown protein
106371	6.69	0.10	3.58	0.04	5.47	0.06	8.617 down	2.322 down	0.00	1022.21	SSCRP
106391	12.95	0.01	12.86	0.01	11.14	0.36	1.062 down	3.506 down	0.00	40.27	unknown protein
106405	5.90	0.06	6.88	0.01	7.56	0.42	1.978 up	3.158 up	0.01	17.68	extracellular lipase-like protein
106437	11.06	0.11	10.62	0.15	9.69	0.24	1.356 down	2.581 down	0.00	34.56	unknown protein
106445	5.01	0.40	4.84	0.38	7.67	0.40	1.126 down	6.337 up	0.00	48.88	unique protein
106452	11.33	0.21	9.63	0.00	9.12	0.25	3.258 down	4.639 down	0.00	74.10	unknown protein
106453	6.35	0.59	6.53	0.57	8.25	0.59	1.138 up	3.756 up	0.03	9.80	SSCRP
106470	2.42	0.11	2.36	0.22	3.61	0.14	1.047 down	2.278 up	0.00	62.53	unique protein
106479	2.63	0.15	2.84	0.27	4.45	0.10	1.156 up	3.527 up	0.00	118.28	unknown protein
106480	4.55	0.06	5.77	0.29	7.32	0.74	2.327 up	6.820 up	0.01	15.66	flavoprotein monooxygenases
106486	2.87	0.02	2.98	0.14	4.67	0.05	1.074 up	3.471 up	0.00	545.63	unique protein
106488	8.71	0.12	8.15	0.31	6.86	0.51	1.475 down	3.615 down	0.01	14.77	unknown protein
106490	4.39	0.13	4.64	0.21	5.94	0.31	1.188 up	2.925 up	0.00	29.69	unknown protein. 3 TM. only in Neurospora and Chaetomium
106491	8.56	0.06	6.98	0.10	6.22	0.28	2.986 down	5.048 down	0.00	74.76	unknown protein
106492	5.08	0.27	5.19	0.30	6.83	0.45	1.077 up	3.370 up	0.01	19.04	SSCRP
106493	2.28	0.02	2.40	0.05	3.86	0.16	1.085 up	2.974 up	0.00	152.76	unknown protein
106499	3.27	0.08	2.81	0.24	4.55	0.08	1.377 down	2.433 up	0.00	141.90	unknown protein
106538	9.51	0.02	12.40	0.21	12.85	0.42	7.440 up	10.140 up	0.00	67.85	HFB4
106556	7.18	0.20	6.64	0.04	8.85	0.90	1.458 down	3.185 up	0.04	8.00	unknown protein
106564	6.10	0.32	6.15	0.34	7.41	0.21	1.032 up	2.475 up	0.01	23.69	unique protein
106583	2.60	0.02	2.44	0.10	4.29	0.04	1.121 down	3.225 up	0.00	996.83	unique protein
106604	6.83	0.55	6.96	0.16	8.33	0.41	1.092 up	2.819 up	0.02	12.55	unique protein
106654	8.93	0.26	8.49	0.11	6.84	0.35	1.352 down	4.265 down	0.00	40.21	Zn2Cys6 transcriptional regulator
106657	11.17	0.24	10.54	0.00	9.79	0.10	1.546 down	2.603 down	0.00	74.92	Zn2Cys6 transcriptional regulator

106659	8.97	0.12	8.56	0.01	6.60	0.58	1.329 down	5.170 down	0.01	23.05	unknown protein
106661	4.37	0.17	3.58	0.03	5.90	0.21	1.727 down	2.889 up	0.00	118.76	aspartyl protease
106662	5.03	0.07	5.09	0.64	6.24	0.11	1.038 up	2.307 up	0.01	15.36	SSCRP
106677	9.36	0.11	7.74	0.13	7.44	0.07	3.067 down	3.773 down	0.00	287.76	Zn2Cys6 transcriptional regulator
106684	2.94	0.32	3.62	0.02	4.76	0.17	1.611 up	3.536 up	0.00	63.47	unknown protein
106686	9.25	0.02	8.06	0.14	11.51	0.74	2.277 down	4.800 up	0.01	26.98	unknown protein
106695	10.79	0.12	7.71	0.18	9.37	0.10	8.454 down	2.670 down	0.00	311.43	Sulfite oxidase. molybdopterin-binding component
106697	7.14	0.15	6.03	0.13	5.51	0.43	2.150 down	3.088 down	0.01	14.93	esterase/lipase. HGT
106706	11.27	0.05	10.77	0.01	8.28	0.42	1.414 down	7.936 down	0.00	72.47	Zn2Cys6 transcriptional regulator
106798	9.82	0.05	11.74	0.29	12.06	0.27	3.762 up	4.706 up	0.00	56.50	GCN5-N-acetyltransferase
106801	10.74	0.03	10.75	0.05	9.74	0.22	1.007 up	2.007 down	0.00	34.47	unknown protein
106812	2.68	0.18	2.82	0.03	4.99	0.27	1.106 up	4.957 up	0.00	97.91	unique protein
106828	10.98	0.11	11.35	0.01	9.89	0.13	1.297 up	2.124 down	0.00	136.12	unknown protein
106869	4.56	0.06	5.35	0.22	6.38	0.50	1.737 up	3.537 up	0.01	14.82	unknown protein
106897	4.65	0.01	5.10	0.19	5.90	0.31	1.367 up	2.386 up	0.01	17.74	unique protein
106928	4.13	0.07	4.17	0.04	6.26	0.35	1.027 up	4.367 up	0.00	60.65	Na/K ATPase alpha 1 subunit.
106939	5.26	0.21	7.45	0.22	6.60	0.28	4.556 up	2.534 up	0.00	36.92	protein kinase
106947	3.33	0.30	4.20	0.18	5.20	0.13	1.833 up	3.657 up	0.00	70.42	unique protein
106958	11.60	0.12	11.76	0.09	10.14	0.25	1.115 up	2.764 down	0.00	57.33	unique protein
106960	3.53	0.51	3.38	0.29	5.62	0.28	1.109 down	4.269 up	0.00	41.17	unknown protein
106998	4.85	0.09	4.72	0.14	6.17	0.54	1.098 down	2.488 up	0.02	10.78	unknown protein
107002	10.27	0.01	9.74	0.07	8.48	0.18	1.448 down	3.478 down	0.00	124.97	unknown protein
107003	8.80	0.08	7.99	0.06	10.86	0.59	1.757 down	4.165 up	0.00	30.54	unknown protein
107007	3.42	0.11	3.28	0.26	4.61	0.18	1.101 down	2.283 up	0.00	44.89	SSCRP
107008	2.95	0.12	3.23	0.14	5.25	0.08	1.208 up	4.930 up	0.00	448.46	unknown protein
107031	8.06	0.13	7.73	0.17	9.41	0.48	1.255 down	2.560 up	0.01	15.79	unique protein
107037	10.10	0.11	11.49	0.15	12.07	0.42	2.634 up	3.940 up	0.01	22.97	unknown protein
107042	6.99	0.12	8.06	0.08	8.07	0.37	2.094 up	2.118 up	0.03	10.08	PTH11 GPCR
107055	11.41	0.05	10.75	0.02	8.69	0.51	1.572 down	6.580 down	0.00	37.76	unknown protein
107070	2.78	0.08	2.73	0.06	4.19	0.06	1.036 down	2.653 up	0.00	465.97	unique protein

107071	3.19	0.01	3.69	0.18	5.10	0.29	1.412 up	3.767 up	0.00	52.01	HET-domain protein. unknown unknown
107107	5.89	0.12	5.64	0.00	7.06	0.32	1.193 down	2.243 up	0.01	26.35	unknown protein
107111	11.88	0.10	11.55	0.12	10.26	0.14	1.258 down	3.064 down	0.00	135.66	unknown protein
107112	5.79	0.14	6.68	0.26	7.11	0.12	1.851 up	2.498 up	0.00	43.82	unique protein
107123	8.55	0.08	9.49	0.05	9.72	0.13	1.913 up	2.239 up	0.00	76.03	mitochondrial import inner membrane translocase subunit tim16
107131	9.64	0.01	10.36	0.31	11.14	0.19	1.657 up	2.838 up	0.00	39.53	unknown protein
107137	13.80	0.10	13.60	0.03	12.66	0.16	1.151 down	2.205 down	0.00	65.25	sterol desaturase family
107142	4.26	0.05	5.25	0.25	5.70	0.16	1.980 up	2.709 up	0.00	48.62	neutral protease 2
107146	9.24	0.11	9.25	0.10	10.48	0.45	1.009 up	2.363 up	0.02	12.33	unknown protein
107194	3.92	0.30	4.96	0.23	5.50	0.24	2.057 up	2.995 up	0.01	26.73	unique protein
107202	10.12	0.10	8.17	0.02	8.85	0.13	3.857 down	2.407 down	0.00	161.99	unknown protein
107207	4.91	0.07	5.33	0.10	6.09	0.09	1.338 up	2.270 up	0.00	130.08	HMG box-containing protein
107208	2.41	0.02	2.49	0.04	3.82	0.03	1.057 up	2.665 up	0.00	2343.52	unique protein
107217	6.96	0.02	4.25	0.00	5.50	0.19	6.515 down	2.752 down	0.00	166.57	unknown protein
107239	9.94	0.05	11.34	0.14	11.05	0.13	2.637 up	2.160 up	0.00	76.09	microtubule protein BIM1. possibly involved in mating. karyogamy or meiosis
107258	12.08	0.11	12.20	0.04	10.86	0.19	1.087 up	2.331 down	0.00	66.64	1-acyl-sn-gylcerol-3-phosphate acyltransferase (Lysophosphatidic acid acyltransferase
107260	12.14	0.07	12.12	0.06	11.04	0.12	1.017 down	2.150 down	0.00	119.87	unknown protein
107268	12.06	0.12	11.68	0.04	10.96	0.09	1.302 down	2.148 down	0.00	120.56	Esterase/lipase/thioesterase
107294	12.26	0.01	12.02	0.21	10.90	0.34	1.177 down	2.568 down	0.01	20.22	RTA1-like protein. 7 TM. responds to xenobiotic stimuli
107297	10.92	0.10	9.33	0.12	7.45	0.17	3.011 down	11.076 down	0.00	368.20	unique protein
107306	6.29	0.07	6.55	0.02	5.27	0.18	1.201 up	2.031 down	0.00	68.00	unique protein
107329	4.80	0.15	4.36	0.54	6.80	0.22	1.361 down	3.984 up	0.00	55.51	methionine synthase
107340	14.37	0.03	13.30	0.11	11.84	0.48	2.102 down	5.791 down	0.00	32.32	unknown protein
107347	4.80	0.32	6.38	0.43	6.08	0.50	2.995 up	2.418 up	0.05	7.26	SSCRP
107349	3.43	0.11	3.75	0.04	4.75	0.21	1.248 up	2.496 up	0.00	47.81	unknown protein
107358	5.33	0.13	5.12	0.30	6.48	0.22	1.150 down	2.227 up	0.00	32.21	unique protein
107369	12.41	0.10	10.99	0.08	10.70	0.16	2.688 down	3.284 down	0.00	103.53	guanine nucleotide exchange factor-like protein
107386	11.23	0.09	11.28	0.16	12.71	0.40	1.032 up	2.794 up	0.01	20.43	unknown protein. only in Gibberella and Neurospora
107445	3.37	0.00	3.49	0.05	5.22	0.16	1.087 up	3.595 up	0.00	199.16	unknown protein
107463	10.53	0.13	9.45	0.12	9.49	0.24	2.112 down	2.059 down	0.01	20.27	exoribonuclease Dhp1

107464	7.23	0.00	7.47	0.09	10.75	0.31	1.179 up	11.473 up	0.00	194.77	unknown protein
107475	11.38	0.06	10.64	0.12	9.45	0.50	1.673 down	3.812 down	0.01	17.32	unknown protein
107483	9.31	0.13	9.36	0.04	7.83	0.14	1.037 up	2.775 down	0.00	145.80	unknown protein
107488	3.68	0.52	3.83	0.19	5.22	0.19	1.111 up	2.913 up	0.01	26.13	unknown protein
107494	12.60	0.02	9.59	0.10	10.48	0.14	8.068 down	4.350 down	0.00	371.71	unique protein
107495	11.95	0.05	7.25	0.16	10.94	0.16	25.930 down	2.009 down	0.00	603.83	unknown protein
107507	9.92	0.03	13.02	0.03	11.70	0.54	8.541 up	3.427 up	0.01	27.52	unknown protein
107513	5.11	0.16	5.96	0.11	6.14	0.32	1.801 up	2.031 up	0.03	10.26	unique protein
107524	9.58	0.03	9.51	0.02	8.08	0.33	1.048 down	2.835 down	0.00	33.04	unique protein
107526	5.17	0.11	5.15	0.48	6.39	0.11	1.014 down	2.315 up	0.01	26.89	short chain dehydrognease/reductase
107554	11.58	0.02	10.72	0.06	10.56	0.03	1.815 down	2.036 down	0.00	544.19	glutathione transferase
107564	9.34	0.04	10.36	0.05	10.44	0.29	2.034 up	2.146 up	0.01	16.56	Sin3-associated polypeptide Sap18
107595	3.84	0.14	3.94	0.04	5.32	0.29	1.072 up	2.779 up	0.00	37.87	unique protein
107601	11.55	0.24	11.33	0.09	12.74	0.34	1.169 down	2.275 up	0.01	20.73	unknown Zn-finger protein. Tim10/DPP type
107639	11.41	0.03	9.20	0.10	9.93	0.10	4.604 down	2.784 down	0.00	308.31	unknown protein
107641	5.52	0.02	5.94	0.19	11.06	0.08	1.342 up	46.831 up	0.00	2546.06	unique protein
107657	3.82	0.04	3.89	0.04	5.45	0.17	1.044 up	3.085 up	0.00	138.73	unique protein
107669	10.70	0.08	10.57	0.06	9.10	0.30	1.094 down	3.048 down	0.00	42.95	Dipeptidyl aminopeptidase
107670	10.98	0.02	10.99	0.13	8.80	0.21	1.009 up	4.515 down	0.00	154.43	unknown protein
107674	5.95	0.02	6.31	0.12	11.19	0.49	1.279 up	37.788 up	0.00	173.78	unique protein
107685	5.31	0.33	5.53	0.42	6.81	0.41	1.171 up	2.829 up	0.02	12.56	unique protein
107704	9.43	0.17	10.29	0.01	11.44	0.10	1.814 up	4.051 up	0.00	252.69	unknown protein. only in A. fumigatus
107717	3.27	0.21	3.26	0.02	5.12	0.28	1.004 down	3.605 up	0.00	61.26	SSCRP
107742	5.14	0.07	5.81	0.23	6.18	0.17	1.583 up	2.044 up	0.01	23.78	unique protein
107743	6.39	0.47	5.86	0.37	9.17	0.43	1.447 down	6.870 up	0.00	52.38	unique protein
107811	4.88	0.09	4.34	0.31	6.04	0.14	1.458 down	2.235 up	0.00	70.12	unknown protein
107814	3.07	0.13	3.65	0.03	4.67	0.14	1.489 up	3.026 up	0.00	129.78	unknown protein
107835	6.99	0.20	7.56	0.25	10.42	0.23	1.480 up	10.793 up	0.00	194.17	Arylacetamide deacetylase
107843	4.75	0.03	5.22	0.04	6.24	0.14	1.380 up	2.810 up	0.00	143.78	unique protein
107844	4.60	0.29	4.38	0.26	6.53	0.41	1.161 down	3.817 up	0.00	32.37	unknown protein. 3 TM. only in Gibberella and Chaetomium

107846	3.18	0.09	3.52	0.07	4.92	0.12	1.262 up	3.339 up	0.00	231.90	unknown protein
107848	9.85	0.08	11.61	0.23	11.92	0.42	3.382 up	4.186 up	0.01	25.48	multicopper oxidase type 1. secreted
107850	9.71	0.12	7.79	0.16	11.15	0.35	3.764 down	2.720 up	0.00	93.69	CE1 poly(3-hydroxybutyrate) depolymerase
107853	10.62	0.19	7.18	0.13	8.42	0.44	10.904 down	4.618 down	0.00	48.45	unknown protein
107857	9.52	0.06	8.77	0.06	8.40	0.21	1.684 down	2.167 down	0.00	30.75	unknown protein
107867	4.28	0.20	4.15	0.07	6.08	0.27	1.095 down	3.495 up	0.00	68.25	unique protein
107868	2.71	0.38	2.44	0.07	3.93	0.05	1.211 down	2.333 up	0.00	59.72	unknown protein
107869	5.12	0.13	3.59	0.11	6.35	0.20	2.888 down	2.341 up	0.00	178.26	unknown protein
107871	13.11	0.04	13.06	0.06	11.30	0.22	1.034 down	3.499 down	0.00	102.81	unknown protein
107888	11.77	0.19	11.35	0.22	9.91	0.40	1.333 down	3.619 down	0.01	24.63	unknown protein
107896	6.60	0.22	7.04	0.01	8.03	0.39	1.353 up	2.696 up	0.01	15.68	Isoflavone reductase
107914	9.63	0.10	9.37	0.10	7.58	0.19	1.197 down	4.142 down	0.00	150.08	unique protein
107935	6.88	0.01	6.30	0.07	5.85	0.22	1.500 down	2.048 down	0.01	23.64	Nucleolar GTPase/ATPase p130
107938	10.43	0.07	10.19	0.01	9.33	0.18	1.175 down	2.139 down	0.00	46.35	esterase or lipase
107947	12.36	0.13	12.35	0.05	10.52	0.26	1.010 down	3.591 down	0.00	75.29	Acyl-CoA synthetase
107949	3.64	0.22	3.37	0.17	4.88	0.10	1.208 down	2.364 up	0.00	87.52	unknown protein with ThiJ/PfpI domain
107961	5.57	0.24	5.45	0.29	7.18	0.35	1.088 down	3.059 up	0.01	27.76	unique protein
107974	4.73	0.37	4.83	0.45	7.11	0.43	1.077 up	5.221 up	0.00	30.65	BZIP transcriptional regulator transcription factor JlbA/IDI-4
107998	3.07	0.27	3.05	0.10	4.92	0.23	1.017 down	3.595 up	0.00	70.63	unique protein
108007	3.83	0.15	3.92	0.18	5.21	0.09	1.070 up	2.616 up	0.00	110.97	α/ß hydrolase lipase
108010	9.27	0.05	8.07	0.01	7.05	0.45	2.292 down	4.642 down	0.01	27.72	ABC transporter
108011	2.79	0.00	2.69	0.07	4.60	0.32	1.073 down	3.505 up	0.00	56.73	unknown protein
108012	3.10	0.12	3.08	0.00	4.61	0.14	1.013 down	2.836 up	0.00	161.05	serine peptidase S28
108013	4.28	0.23	3.91	0.10	5.85	0.09	1.292 down	2.980 up	0.00	177.36	Zn2Cys6 transcriptional regulator
108025	3.87	0.07	3.98	0.01	5.99	0.22	1.081 up	4.348 up	0.00	141.03	unknown protein
108075	5.26	0.05	6.74	0.03	6.91	0.19	2.786 up	3.138 up	0.00	82.53	unique protein
108081	12.80	0.21	13.62	0.06	13.82	0.10	1.766 up	2.025 up	0.00	45.10	kinetochore protein spc24. putative
108143	6.13	0.07	11.03	0.06	12.41	0.12	29.783 up	77.549 up	0.00	2605.29	unique protein
108144	4.07	0.30	7.99	0.09	9.49	0.24	15.129 up	42.756 up	0.00	368.68	unknown protein
108145	5.88	0.09	11.77	0.22	11.48	0.22	59.226 up	48.437 up	0.00	590.83	unique protein

108155	7.78	0.44	7.98	0.30	9.93	0.79	1.148 up	4.428 up	0.03	9.71	unknown protein. unique in fungi
108158	12.79	0.03	12.37	0.09	11.59	0.07	1.341 down	2.295 down	0.00	218.59	unknown protein
108185	4.12	0.23	4.27	0.21	6.09	0.25	1.108 up	3.932 up	0.00	62.49	unknown protein. unique in fungi
108188	3.28	0.19	3.28	0.00	5.35	0.48	1.002 down	4.188 up	0.00	29.87	unique protein
108201	12.53	0.14	12.04	0.07	10.60	0.14	1.405 down	3.825 down	0.00	177.30	Unknown protein with reductase domain
108208	12.34	0.02	11.70	0.16	10.66	0.46	1.553 down	3.192 down	0.01	15.49	unknown protein
108233	7.69	0.01	8.07	0.00	5.71	0.71	1.293 up	3.966 down	0.01	15.85	unknown protein. secreted. only in fungi
108251	2.69	0.06	2.87	0.06	4.33	0.06	1.130 up	3.113 up	0.00	649.20	SSCRP
108261	10.97	0.12	13.23	0.05	12.84	0.11	4.792 up	3.644 up	0.00	310.19	SSCRP
108263	11.91	0.07	11.88	0.11	10.75	0.27	1.021 down	2.224 down	0.01	27.03	unknown protein
108287	11.57	0.17	11.36	0.09	10.33	0.08	1.155 down	2.372 down	0.00	118.23	unknown protein
108333	9.27	0.32	8.98	0.05	7.77	0.24	1.223 down	2.829 down	0.00	34.40	unknown protein
108335	4.12	0.07	5.20	0.27	5.30	0.19	2.116 up	2.272 up	0.01	27.59	unknown protein
108339	6.47	0.39	6.89	0.27	8.22	0.35	1.337 up	3.360 up	0.01	20.42	unique protein
108340	7.64	0.02	6.75	0.06	10.08	0.19	1.860 down	5.397 up	0.00	406.14	unique protein
108341	6.90	0.55	7.50	0.09	8.89	0.59	1.519 up	3.967 up	0.02	11.28	unique protein
108342	3.05	0.16	3.56	0.44	5.20	0.08	1.424 up	4.452 up	0.00	79.38	unique protein
108343	5.67	0.17	6.74	0.20	6.96	0.29	2.097 up	2.438 up	0.01	17.71	unique protein
108344	5.04	0.08	5.15	0.12	6.32	0.30	1.076 up	2.424 up	0.01	26.47	unique protein
108346	3.46	0.08	3.59	0.18	5.28	0.26	1.099 up	3.550 up	0.00	65.72	GH18. chitinase CHI18-8
108348	5.19	0.27	5.61	0.03	6.46	0.38	1.335 up	2.410 up	0.02	11.86	unknown protein
108367	4.60	0.47	5.13	0.15	8.15	0.67	1.444 up	11.732 up	0.00	34.53	unique protein
108383	3.63	0.37	4.05	0.28	5.68	0.38	1.339 up	4.132 up	0.01	27.20	unique protein
108384	4.80	0.30	5.15	0.32	7.85	1.07	1.269 up	8.260 up	0.02	11.47	unique protein
108412	2.33	0.02	2.30	0.16	3.67	0.09	1.023 down	2.526 up	0.00	191.57	SSCRP
108418	6.25	0.06	7.39	0.01	7.81	0.28	2.204 up	2.947 up	0.00	34.58	SSCRP
108431	9.94	0.23	9.89	0.06	11.39	0.13	1.038 down	2.735 up	0.00	103.00	unknown protein
108449	10.53	0.13	11.14	0.02	9.44	0.14	1.525 up	2.132 down	0.00	134.86	unknown protein
108457	4.12	0.30	4.55	0.00	5.21	0.26	1.349 up	2.131 up	0.02	14.32	unique protein
108482	8.76	0.06	8.85	0.11	11.16	0.29	1.064 up	5.286 up	0.00	102.80	fumarylacetoacetate hydrolase

108542	10.56	0.07	11.56	0.21	11.80	0.19	2.003 up	2.357 up	0.00	32.11	unknown protein
108559	13.24	0.08	13.32	0.02	12.02	0.27	1.051 up	2.328 down	0.00	34.99	unknown protein
108566	5.95	0.24	6.34	0.09	7.56	0.27	1.313 up	3.061 up	0.00	36.07	unknown protein
108585	6.82	0.10	6.41	0.16	11.23	0.73	1.330 down	21.213 up	0.00	65.35	unknown protein with CHAP domain
108586	8.03	0.06	10.60	0.04	13.34	0.33	5.954 up	39.802 up	0.00	296.23	unknown protein
108604	4.70	0.06	4.67	0.59	6.30	0.37	1.020 down	3.048 up	0.01	17.22	unique protein
108605	7.11	0.06	7.84	0.12	8.67	0.21	1.661 up	2.953 up	0.00	54.22	unique protein
108635	6.16	0.11	6.94	0.25	7.59	0.12	1.715 up	2.706 up	0.00	57.99	unknown protein
108655	5.91	0.06	6.04	0.12	7.06	0.09	1.090 up	2.217 up	0.00	146.02	unknown protein. secreted
108663	7.53	0.22	8.83	0.05	12.65	0.16	2.454 up	34.752 up	0.00	789.27	SSCRP
108665	4.19	0.21	3.89	0.06	5.22	0.33	1.229 down	2.034 up	0.01	19.51	unknown protein
108681	4.07	0.21	4.23	0.30	5.90	0.20	1.117 up	3.541 up	0.00	61.22	unknown protein
108683	6.49	0.04	6.06	0.05	7.60	0.12	1.342 down	2.152 up	0.00	192.75	unknown protein. contains 5 TM
108686	9.02	0.01	7.62	0.17	6.79	0.32	2.650 down	4.702 down	0.00	49.70	Aspartyl protease
108721	3.11	0.14	3.03	0.09	6.78	1.12	1.053 down	12.803 up	0.01	18.11	unknown protein. unique in fungi
108749	10.60	0.05	10.57	0.05	13.86	0.09	1.017 down	9.597 up	0.00	1792.15	Copper transporter
108776	5.42	0.14	5.50	0.11	6.77	0.23	1.054 up	2.537 up	0.00	44.04	GH55 β-1.3-glucanase
108778	12.10	0.09	12.68	0.21	13.15	0.12	1.494 up	2.071 up	0.00	40.24	unknown protein
108802	5.40	0.43	7.23	0.16	6.47	0.50	3.568 up	2.104 up	0.03	8.78	unique protein
108806	13.41	0.26	13.31	0.20	12.04	0.12	1.073 down	2.590 down	0.00	60.39	unknown protein
108832	4.67	0.03	5.36	0.05	6.43	0.40	1.613 up	3.379 up	0.01	22.87	unknown protein
108866	8.15	0.03	9.44	0.08	9.99	0.19	2.443 up	3.576 up	0.00	93.55	unknown protein
108885	12.48	0.05	11.32	0.02	10.31	0.11	2.243 down	4.499 down	0.00	435.96	amidase
108893	10.09	0.02	8.02	0.18	8.29	0.21	4.190 down	3.480 down	0.00	80.26	MFS permease
108899	11.98	0.11	11.29	0.03	10.38	0.11	1.612 down	3.030 down	0.00	197.34	unknown protein
108914	11.09	0.02	10.39	0.00	13.20	0.73	1.630 down	4.303 up	0.01	19.69	methyltransferase type 11
108920	11.89	0.11	11.81	0.03	10.60	0.19	1.058 down	2.443 down	0.00	66.13	zinc-binding dehydrogenase
108928	3.79	0.20	4.10	0.14	4.92	0.19	1.235 up	2.186 up	0.00	28.83	unique protein
108940	12.64	0.10	11.93	0.26	10.68	0.16	1.633 down	3.881 down	0.00	92.71	Zn2Cys6 transcriptional regulator
108953	7.04	0.05	7.98	0.00	8.23	0.32	1.913 up	2.273 up	0.01	15.58	unknown protein

108996	9.41	0.22	10.37	0.26	12.84	0.67	1.946 up	10.748 up	0.00	30.91	unique protein
109030	2.61	0.09	3.29	0.04	4.54	0.14	1.610 up	3.826 up	0.00	195.92	unique protein
109044	3.59	0.12	3.69	0.36	5.01	0.19	1.071 up	2.676 up	0.00	37.24	unique protein
109071	4.63	0.45	4.22	0.23	7.66	0.42	1.333 down	8.135 up	0.00	66.01	unknown protein
109075	5.85	0.19	5.96	0.08	7.07	0.54	1.076 up	2.320 up	0.04	7.50	unknown protein
109102	3.64	0.13	3.96	0.14	5.27	0.27	1.240 up	3.093 up	0.00	42.10	unknown protein
109122	10.96	0.15	9.07	0.13	9.78	0.09	3.690 down	2.256 down	0.00	142.60	Amino acid transporters
109146	9.87	0.10	5.49	0.08	4.27	0.07	20.774 down	48.526 down	0.00	3317.18	PTH11 GPCR
109147	2.36	0.22	2.37	0.18	3.66	0.06	1.004 up	2.450 up	0.00	90.13	unknown protein
109231	4.17	0.29	3.84	0.02	6.01	0.25	1.265 down	3.577 up	0.00	76.81	SSCRP
109234	11.81	0.29	10.98	0.18	12.95	0.33	1.780 down	2.210 up	0.00	30.69	D-aminopeptidase
109237	4.75	0.40	4.77	0.12	6.45	0.25	1.014 up	3.251 up	0.00	40.54	Cytochrome P450
109239	3.60	0.42	3.18	0.39	5.80	0.53	1.331 down	4.596 up	0.01	24.74	unknown protein. only in Aspergilli
109243	4.67	0.31	5.14	0.27	7.99	0.60	1.386 up	9.969 up	0.00	38.89	unique protein
109244	10.65	0.08	9.82	0.17	9.58	0.24	1.775 down	2.097 down	0.01	18.07	unique protein
109249	10.73	0.05	9.00	0.08	8.78	0.33	3.319 down	3.862 down	0.00	40.37	unknown protein
109255	6.96	0.00	6.56	0.02	8.07	0.57	1.311 down	2.170 up	0.03	9.21	SSCRP
109264	3.44	0.45	3.93	0.01	4.61	0.19	1.409 up	2.256 up	0.01	16.10	unique protein
109267	5.75	0.25	6.81	0.08	6.83	0.37	2.083 up	2.106 up	0.03	9.11	unique protein
109278	7.01	0.15	8.24	0.07	9.85	0.82	2.351 up	7.186 up	0.02	13.89	GH24 lysozyme
109284	4.41	0.23	4.47	0.05	6.74	0.08	1.044 up	5.045 up	0.00	350.93	unknown protein
109304	3.87	0.24	4.23	0.05	5.65	0.26	1.284 up	3.443 up	0.00	50.11	unknown protein
109305	4.03	0.58	4.23	0.43	6.06	0.20	1.144 up	4.070 up	0.00	29.21	unique protein
109307	9.01	0.08	7.80	0.18	7.62	0.44	2.326 down	2.633 down	0.02	11.18	unknown protein
109319	5.26	0.22	5.58	0.10	7.79	0.22	1.253 up	5.783 up	0.00	136.60	unknown protein
109320	4.60	0.02	5.08	0.09	6.84	0.28	1.393 up	4.709 up	0.00	81.05	unique protein
109331	3.99	0.31	4.61	0.03	5.55	0.12	1.533 up	2.944 up	0.00	61.47	unknown protein. secreted. 5 TM
109334	4.07	0.15	4.41	0.14	5.89	0.37	1.270 up	3.527 up	0.00	30.22	unknown protein
109335	4.63	0.01	6.04	0.09	6.06	0.24	2.651 up	2.695 up	0.00	40.87	SSCRP
109338	3.88	0.12	3.47	0.43	5.40	0.08	1.325 down	2.868 up	0.00	68.86	unknown protein. in A. oryzae and G. zeae

109341	7.81	0.07	6.31	0.11	6.63	0.15	2.831 down	2.260 down	0.00	78.13	ankyrin
109345	3.44	0.04	3.41	0.21	5.12	0.44	1.019 down	3.225 up	0.01	22.77	unique protein
109349	2.70	0.14	2.76	0.06	4.07	0.10	1.041 up	2.587 up	0.00	172.41	short chain dehydrogenase/reductase
109351	2.75	0.04	3.06	0.04	4.64	0.19	1.237 up	3.722 up	0.00	136.67	unique protein
109353	3.78	0.09	4.55	0.16	5.49	0.12	1.696 up	3.270 up	0.00	139.14	unknown protein
109357	4.32	0.25	4.91	0.16	6.08	0.24	1.502 up	3.389 up	0.00	44.36	short chain dehydrognease/reductase
109359	5.56	0.14	5.72	0.25	6.64	0.45	1.115 up	2.116 up	0.04	7.48	unknown protein
109361	4.51	0.14	6.27	0.15	5.92	0.17	3.399 up	2.671 up	0.00	69.42	GT32 α-glycosyltransferase
109364	4.35	0.51	4.48	0.04	6.32	0.40	1.098 up	3.925 up	0.01	25.02	unknown protein
109367	9.99	0.02	4.94	0.10	6.36	0.56	33.158 down	12.434 down	0.00	74.15	unique protein
109370	3.19	0.27	3.42	0.00	4.72	0.12	1.169 up	2.885 up	0.00	90.48	SSCRP
109376	2.67	0.00	2.80	0.16	4.78	0.13	1.098 up	4.328 up	0.00	261.80	glutamate decarbounknown proteinlase
109379	12.45	0.04	10.85	0.12	10.79	0.26	3.043 down	3.165 down	0.00	46.07	short chain dehydrogenase/reductase
109388	3.92	0.33	3.74	0.04	5.08	0.14	1.137 down	2.230 up	0.00	47.87	unknown protein
109392	5.34	0.21	6.06	0.10	7.85	0.54	1.649 up	5.707 up	0.01	26.05	unknown protein
109410	10.70	0.05	10.31	0.20	9.09	0.25	1.307 down	3.048 down	0.00	43.94	unknown protein
109415	3.21	0.13	3.39	0.10	5.66	0.48	1.134 up	5.453 up	0.00	38.36	unknown protein
109416	12.14	0.08	11.94	0.05	11.14	0.41	1.147 down	2.005 down	0.04	8.14	unknown protein
109435	9.96	0.00	10.61	0.07	11.23	0.13	1.562 up	2.412 up	0.00	96.21	unknown protein. 1 TM
109475	4.68	0.07	4.87	0.04	6.85	0.47	1.147 up	4.516 up	0.00	32.23	unique protein
109486	9.07	0.09	9.49	0.08	8.04	0.52	1.343 up	2.030 down	0.03	9.64	unique protein
109489	3.88	0.31	4.14	0.10	5.48	0.30	1.200 up	3.025 up	0.00	29.73	unknown protein
109526	8.28	0.07	2.60	0.26	9.46	0.28	51.119 down	2.258 up	0.00	525.54	SNF2 family helicase/ATPase
109538	12.62	0.22	8.46	0.04	9.66	0.25	17.920 down	7.807 down	0.00	197.72	BZIP transcriptional regulator
109562	5.56	0.39	7.79	0.03	7.69	0.26	4.685 up	4.390 up	0.00	49.41	unique protein
109590	3.19	0.17	3.44	0.05	4.69	0.09	1.189 up	2.828 up	0.00	186.79	unknown protein
109607	2.27	0.17	2.19	0.04	3.58	0.04	1.056 down	2.491 up	0.00	261.83	unique protein
109608	3.13	0.13	3.33	0.10	4.72	0.19	1.148 up	3.013 up	0.00	85.18	unique protein
109668	5.00	0.11	4.82	0.03	6.60	0.36	1.135 down	3.029 up	0.00	35.17	unique protein
109677	10.46	0.11	10.96	0.20	8.52	0.25	1.422 up	3.833 down	0.00	102.97	MFS permease

109721	7.52	0.19	7.82	0.02	8.62	0.15	1.234 up	2.147 up	0.00	46.43	unknown protein
109725	4.14	0.26	4.08	0.02	6.24	0.11	1.040 down	4.279 up	0.00	213.87	unknown protein
109726	4.53	0.07	5.37	0.06	5.54	0.19	1.795 up	2.016 up	0.00	30.87	unique protein
109748	12.04	0.01	11.35	0.18	9.99	0.35	1.616 down	4.129 down	0.00	38.19	Zn2Cys6 transcriptional regulator
109756	10.96	0.06	11.00	0.09	9.81	0.15	1.026 up	2.215 down	0.00	81.77	unknown protein
109787	13.60	0.05	12.68	0.05	12.51	0.16	1.891 down	2.121 down	0.00	47.32	SSCRP
109790	11.91	0.05	12.12	0.19	12.99	0.21	1.158 up	2.102 up	0.01	27.08	pyridoxamine 5'-phosphate oxidase
109801	6.34	0.33	5.77	0.22	8.73	0.31	1.490 down	5.221 up	0.00	81.05	unknown protein
109815	5.02	0.06	4.72	0.00	6.14	0.21	1.229 down	2.176 up	0.00	59.92	unknown protein
109835	10.61	0.04	10.87	0.04	8.57	0.17	1.204 up	4.092 down	0.00	255.60	unknown protein. thaumatin family
109837	4.07	0.21	4.55	0.85	5.93	0.12	1.391 up	3.619 up	0.01	16.77	MFS permease
109850	12.58	0.02	10.77	0.00	9.99	0.12	3.502 down	6.017 down	0.00	550.87	unknown protein
109872	12.98	0.06	12.65	0.04	11.79	0.17	1.258 down	2.277 down	0.00	57.97	unknown protein
109883	12.05	0.06	11.99	0.20	10.89	0.35	1.039 down	2.228 down	0.01	15.63	unknown protein
109895	11.89	0.11	11.77	0.20	10.47	0.05	1.084 down	2.668 down	0.00	150.37	ATP-dependent Clp protease
109908	5.18	0.06	5.14	0.01	6.59	0.22	1.024 down	2.665 up	0.00	70.37	C2H2 transcriptional regulator
109910	5.26	0.11	5.25	0.17	6.61	0.29	1.006 down	2.551 up	0.00	32.21	unique protein. secreted
109911	3.93	0.18	4.17	0.12	6.27	0.54	1.179 up	5.082 up	0.01	27.13	SSCRP
109925	12.11	0.02	6.26	0.43	10.15	0.60	57.532 down	3.871 down	0.00	70.89	unknown protein
109929	11.89	0.12	11.56	0.17	10.27	0.16	1.256 down	3.070 down	0.00	89.64	unknown protein
109934	6.88	0.45	7.14	0.10	8.70	0.37	1.194 up	3.526 up	0.01	22.96	unknown protein
109943	3.07	0.03	3.05	0.03	4.75	0.10	1.015 down	3.202 up	0.00	460.78	unknown protein
109946	3.22	0.18	3.14	0.13	4.53	0.19	1.057 down	2.469 up	0.00	60.19	unknown protein
109949	5.83	0.20	5.65	0.23	7.88	0.42	1.132 down	4.140 up	0.00	37.51	unknown protein
109952	3.84	0.21	4.23	0.29	5.86	0.33	1.302 up	4.036 up	0.00	37.12	unknown protein. containing ankyrin repeats
109960	9.73	0.00	9.32	0.01	6.89	0.90	1.329 down	7.162 down	0.02	14.60	unknown protein in Sordariomycetes
109967	4.41	0.11	4.45	0.16	5.98	0.29	1.023 up	2.968 up	0.00	41.68	unique protein. 4 TM
109998	13.32	0.05	12.91	0.05	12.03	0.15	1.333 down	2.444 down	0.00	90.40	glutathione S-transferase
110007	10.98	0.02	10.55	0.08	9.28	0.22	1.345 down	3.235 down	0.00	73.32	unique protein
110025	5.20	0.21	5.87	0.08	7.34	0.14	1.590 up	4.411 up	0.00	157.65	SSCRP

110035	4.36	0.39	4.06	0.13	7.34	0.98	1.229 down	7.891 up	0.01	16.12	unknown protein. in Sordariomycetes
110056	6.31	0.07	6.53	0.01	7.83	0.45	1.163 up	2.870 up	0.01	16.65	unknown protein
110058	4.66	0.16	4.93	0.21	5.93	0.16	1.211 up	2.413 up	0.00	45.36	SSCRP
110078	6.74	0.24	6.49	0.25	8.69	0.57	1.188 down	3.866 up	0.01	19.59	SSCRP
110096	8.03	0.15	8.75	0.19	9.31	0.37	1.655 up	2.439 up	0.02	12.17	unknown protein
110117	6.81	0.44	7.39	0.26	9.11	0.62	1.491 up	4.906 up	0.01	14.86	SSCRP
110127	6.92	0.22	8.06	0.07	8.44	0.54	2.193 up	2.856 up	0.04	8.34	unique protein
110128	4.28	0.77	4.46	0.13	8.80	0.61	1.136 up	23.050 up	0.00	56.24	unique protein
110131	2.74	0.17	2.86	0.00	4.54	0.14	1.089 up	3.501 up	0.00	170.19	unknown protein
110133	11.52	0.12	11.06	0.08	10.32	0.14	1.377 down	2.299 down	0.00	63.62	Mitochondrial carrier protein
110151	4.59	0.06	5.24	0.21	10.46	0.22	1.560 up	58.240 up	0.00	814.50	unique secreted protein
110152	10.75	0.04	12.02	0.06	12.00	0.17	2.400 up	2.376 up	0.00	62.60	BZIP transcriptional regulator
110153	3.08	0.12	3.41	0.01	4.53	0.11	1.254 up	2.726 up	0.00	171.26	unique protein
110173	6.96	0.08	9.37	0.16	8.29	0.27	5.323 up	2.512 up	0.00	60.04	unknown protein
110197	4.79	0.22	5.00	0.08	6.02	0.26	1.161 up	2.347 up	0.01	24.97	unknown protein
110207	3.11	0.07	3.36	0.06	5.24	0.25	1.186 up	4.385 up	0.00	105.06	unique protein
110224	3.69	0.12	3.72	0.12	5.90	0.26	1.019 up	4.625 up	0.00	103.99	unknown protein
110225	6.20	0.17	6.64	0.15	7.49	0.41	1.358 up	2.437 up	0.02	10.94	unknown protein
110259	3.42	0.12	3.43	0.18	4.78	0.12	1.004 up	2.553 up	0.00	107.31	alginate lyases
110261	10.49	0.19	8.66	0.16	11.81	0.25	3.567 down	2.491 up	0.00	132.52	unique protein
110262	9.63	0.07	8.65	0.01	7.98	0.13	1.965 down	3.144 down	0.00	163.53	Zinc-binding oxidoreductase
110263	8.99	0.04	8.67	0.05	7.58	0.23	1.245 down	2.653 down	0.00	51.50	unknown protein
110265	8.11	0.16	4.43	0.10	7.02	0.37	12.882 down	2.138 down	0.00	81.62	unknown protein
110276	6.95	0.18	7.61	0.01	5.87	0.53	1.577 up	2.120 down	0.02	12.56	isoflavone reductase
110281	6.25	0.28	6.39	0.05	7.81	0.40	1.098 up	2.952 up	0.01	20.07	unknown protein
110295	9.88	0.18	10.34	0.14	11.73	0.37	1.372 up	3.610 up	0.00	30.44	ribosomal protein S14
110317	3.11	0.09	3.00	0.07	4.82	0.12	1.083 down	3.254 up	0.00	285.38	GH18. chitinase CHI18-17
110329	3.76	0.15	3.61	0.19	5.51	0.27	1.112 down	3.372 up	0.00	61.29	unknown protein. only present in ascomycota
110337	4.89	0.05	5.04	0.47	6.53	0.46	1.111 up	3.133 up	0.02	14.18	unknown protein with thij/pfpi domain
110343	2.89	0.13	3.23	0.01	5.09	0.35	1.257 up	4.573 up	0.00	54.07	unique protein

110345	4.53	0.05	4.74	0.07	5.65	0.17	1.156 up	2.179 up	0.00	57.77	unique protein
110363	5.58	0.10	6.92	0.12	8.76	0.21	2.528 up	9.052 up	0.00	230.09	unique protein
110366	9.35	0.04	8.65	0.09	7.54	0.28	1.620 down	3.498 down	0.00	46.95	unique protein
110396	11.49	0.03	11.34	0.16	13.17	0.22	1.116 down	3.199 up	0.00	88.22	unknown protein with Tim10/DDP family zinc finger
110403	8.85	0.00	8.93	0.26	7.67	0.37	1.058 up	2.273 down	0.01	15.58	unknown protein
110414	13.22	0.05	12.37	0.08	11.27	0.31	1.803 down	3.884 down	0.00	44.33	Aminotran_Aminotransferase class-III
110415	11.32	0.01	10.91	0.12	10.23	0.10	1.328 down	2.131 down	0.00	102.24	Serine/threonine protein kinase
110430	11.59	0.05	11.04	0.13	10.17	0.23	1.466 down	2.673 down	0.00	42.64	MFS permease
110433	2.40	0.10	2.59	0.11	3.92	0.10	1.141 up	2.874 up	0.00	204.88	unique protein
110440	9.72	0.13	8.62	0.02	7.46	0.09	2.145 down	4.792 down	0.00	446.17	unique protein
110455	8.68	0.09	8.21	0.16	6.06	0.53	1.381 down	6.166 down	0.00	33.03	short chain dehydrogenase/reductase
110457	6.36	0.21	6.19	0.09	7.94	0.37	1.125 down	2.982 up	0.00	29.54	SSCRP
110471	12.46	0.14	12.09	0.09	10.29	0.26	1.289 down	4.504 down	0.00	84.38	Cu2+/Zn2+ superoxide dismutase SOD1
110490	6.52	0.16	7.31	0.12	7.75	0.26	1.721 up	2.343 up	0.01	20.93	unknown protein
110569	6.00	0.40	6.23	0.18	7.36	0.15	1.168 up	2.568 up	0.00	31.05	SSCRP
110595	11.60	0.06	10.68	0.21	8.95	0.52	1.896 down	6.313 down	0.00	30.75	unknown protein
110600	9.36	0.11	10.06	0.09	10.37	0.08	1.629 up	2.023 up	0.00	86.96	unknown protein
110615	11.24	0.11	10.59	0.23	10.21	0.38	1.573 down	2.040 down	0.05	6.99	unknown protein
110638	12.85	0.13	12.92	0.07	11.69	0.12	1.045 up	2.235 down	0.00	110.36	mitochondrial carrier protein
110660	6.30	0.04	5.61	0.06	7.33	0.64	1.611 down	2.045 up	0.04	8.69	unknown protein
110663	4.80	0.26	5.60	0.26	6.38	0.21	1.743 up	3.007 up	0.00	32.23	AMP-dependent synthetase and ligase. putative
110665	4.64	0.14	4.99	0.22	7.82	0.71	1.277 up	9.084 up	0.00	29.02	unique protein
110666	4.02	0.18	3.42	0.33	8.15	0.80	1.512 down	17.592 up	0.00	47.69	ferric reductase
110684	3.59	0.09	3.89	0.42	5.25	0.40	1.232 up	3.155 up	0.01	17.18	unique protein
110686	8.95	0.14	8.81	0.11	7.68	0.27	1.099 down	2.404 down	0.00	29.61	unknown protein
110688	3.29	0.08	3.27	0.28	6.35	0.82	1.016 down	8.358 up	0.01	22.52	unique protein
110689	10.66	0.00	9.07	0.08	8.75	0.27	3.007 down	3.777 down	0.00	55.40	Zn2Cys6 transcriptional regulator
110691	5.68	0.16	5.70	0.02	7.58	0.30	1.009 up	3.730 up	0.00	60.28	unique protein
110712	4.29	0.42	4.08	0.25	5.42	0.11	1.161 down	2.177 up	0.01	27.98	unique protein
110729	3.76	0.05	4.13	0.04	5.42	0.08	1.294 up	3.166 up	0.00	486.94	unique protein

110732	4.31	0.25	3.44	0.21	5.35	0.10	1.826 down	2.061 up	0.00	96.54	unique protein
110735	2.98	0.22	3.13	0.01	4.95	0.16	1.106 up	3.912 up	0.00	149.63	unique protein
110740	4.39	0.07	4.79	0.31	6.15	0.25	1.315 up	3.374 up	0.00	43.87	unknown protein
110749	3.67	0.11	3.54	0.04	4.68	0.13	1.088 down	2.018 up	0.00	88.44	unknown protein
110752	3.08	0.14	3.01	0.04	4.61	0.13	1.050 down	2.880 up	0.00	170.13	unique protein
110753	2.94	0.29	3.22	0.23	4.81	0.16	1.208 up	3.642 up	0.00	72.73	Na+/proline symporter PutP
110757	9.79	0.04	7.40	0.03	7.76	0.13	5.229 down	4.072 down	0.00	341.34	imidazole proprionase-related amidohydrolase
110762	3.24	0.19	5.30	0.11	4.59	0.40	4.159 up	2.539 up	0.01	21.17	unknown protein
110763	3.35	0.25	3.47	0.08	5.16	0.26	1.086 up	3.513 up	0.00	56.98	unknown protein
110767	10.66	0.09	9.06	0.12	9.20	0.16	3.035 down	2.745 down	0.00	83.51	unique protein
110768	13.16	0.22	11.78	0.07	11.43	0.17	2.600 down	3.324 down	0.00	75.87	unknown protein
110791	2.61	0.07	2.51	0.13	4.45	0.16	1.074 down	3.579 up	0.00	179.94	unknown protein
110797	5.21	0.06	4.26	0.28	7.13	0.30	1.931 down	3.776 up	0.00	88.78	unknown protein. 1 TM. only in Gibberella
110811	13.72	0.03	13.59	0.01	12.31	0.14	1.088 down	2.654 down	0.00	143.60	unknown protein
110813	11.61	0.10	12.25	0.01	12.74	0.06	1.560 up	2.185 up	0.00	217.13	Amino acid transporter LysP
110825	6.98	0.07	5.97	0.18	5.98	0.32	2.009 down	2.000 down	0.02	10.77	unique protein
110831	9.34	0.05	9.07	0.04	8.10	0.35	1.207 down	2.371 down	0.01	17.31	unknown protein
110848	3.32	0.16	3.45	0.16	5.57	0.52	1.096 up	4.761 up	0.01	27.31	GH23 exo-ß-1.3-glucanase. distantly related
110850	7.33	0.08	5.89	0.11	6.02	0.32	2.702 down	2.474 down	0.01	21.30	Zn2Cys6 transcriptional regulator
110855	5.72	0.36	8.21	0.14	4.68	0.15	5.605 up	2.058 down	0.00	197.03	unique protein
110860	3.30	0.33	3.81	0.12	4.91	0.24	1.422 up	3.051 up	0.00	33.03	Na+-independent Cl/HCO3 exchanger AE1 and related transporters (SLC4 family)
110866	12.04	0.02	11.71	0.21	9.75	0.09	1.254 down	4.888 down	0.00	318.23	metal-dependent amidase/aminoacylase/carbounknown proteinpeptidase
110868	4.98	0.07	4.40	0.29	6.23	0.46	1.491 down	2.392 up	0.01	17.93	unknown protein
110890	11.15	0.04	11.81	0.16	12.15	0.05	1.586 up	2.003 up	0.00	100.27	porphobilinogen deaminase-like protein
110891	10.04	0.18	6.19	0.20	8.36	0.43	14.392 down	3.207 down	0.00	59.30	unique protein
110894	7.01	0.12	6.23	0.11	9.29	0.91	1.711 down	4.867 up	0.01	14.92	GH30 endo-β-1 6-galactanase
110901	8.71	0.03	8.00	0.06	7.37	0.30	1.634 down	2.520 down	0.01	21.87	Zn2Cys6 transcriptional regulator
110902	6.72	0.44	6.98	0.56	8.70	0.47	1.201 up	3.948 up	0.01	14.66	unique protein
110966	3.00	0.42	3.31	0.11	5.05	0.14	1.240 up	4.159 up	0.00	73.12	unknown protein
111005	3.31	0.04	3.81	0.35	4.99	0.14	1.407 up	3.203 up	0.00	59.76	unique protein

111012	7.76	0.50	7.79	0.23	9.71	0.80	1.020 up	3.849 up	0.04	8.30	unique protein
111027	6.00	0.26	6.68	0.08	8.43	0.17	1.596 up	5.366 up	0.00	144.57	unknown protein
111038	10.59	0.09	10.60	0.09	9.30	0.12	1.002 up	2.448 down	0.00	139.33	unique protein
111048	5.33	0.29	5.54	0.08	6.38	0.11	1.155 up	2.059 up	0.00	35.58	UbiA prenyltransferase. putative
111049	4.28	0.21	4.21	0.18	6.47	0.19	1.047 down	4.564 up	0.00	131.45	cytochrome P450 protein. class IV
111050	7.98	0.12	8.28	0.01	6.90	0.25	1.228 up	2.126 down	0.00	38.80	unique protein
111053	12.41	0.13	8.69	0.10	10.54	0.23	13.178 down	3.633 down	0.00	183.75	retrograde regulation protein 2
111059	9.53	0.10	9.46	0.00	8.18	0.34	1.050 down	2.545 down	0.01	23.87	unknown protein
111082	11.76	0.12	10.93	0.08	13.19	0.10	1.768 down	2.695 up	0.00	364.00	glutathione S transferase. 2 TM
111093	4.25	0.12	3.80	0.37	6.00	0.20	1.361 down	3.370 up	0.00	73.09	3-beta hydroxysteroid dehydrogenase/isomerase. putative
111095	7.57	0.03	10.01	0.10	9.96	0.56	5.410 up	5.226 up	0.01	22.68	unknown protein
111108	3.90	0.07	3.88	0.01	5.86	0.19	1.011 down	3.895 up	0.00	177.71	unknown protein
111109	4.13	0.12	4.62	0.16	5.44	0.24	1.405 up	2.480 up	0.00	30.31	unique protein
111110	7.89	0.02	9.39	0.13	10.24	0.43	2.815 up	5.076 up	0.00	32.79	SAM-dependent methyltransferases
111119	7.37	0.02	6.79	0.00	6.20	0.28	1.487 down	2.244 down	0.01	20.03	bZI) transcription factor. possibly related to AP1
111121	4.77	0.07	5.11	0.00	6.19	0.49	1.267 up	2.663 up	0.02	10.84	metallopeptidase
111124	9.66	0.04	9.78	0.02	8.26	0.30	1.089 up	2.641 down	0.00	40.86	unknown protein
111129	13.52	0.01	13.39	0.11	12.10	0.26	1.091 down	2.666 down	0.00	42.25	unknown protein
111135	11.96	0.08	10.78	0.21	10.25	0.12	2.277 down	3.293 down	0.00	106.10	unknown protein
111138	10.06	0.07	9.40	0.26	8.49	0.24	1.573 down	2.970 down	0.00	36.18	unknown protein. secreted
111144	3.53	0.38	3.71	0.20	5.39	0.20	1.139 up	3.648 up	0.00	52.26	unique protein
111170	6.77	0.06	7.54	0.12	8.76	0.57	1.712 up	3.986 up	0.02	14.29	unknown protein
111171	3.10	0.08	3.47	0.03	4.97	0.12	1.288 up	3.651 up	0.00	276.74	unique protein
111192	10.46	0.11	10.31	0.15	11.59	0.11	1.110 down	2.199 up	0.00	99.58	unknown protein
111194	14.46	0.04	14.35	0.04	13.27	0.25	1.078 down	2.276 down	0.00	33.36	unknown protein
111204	5.44	0.24	5.66	0.17	7.32	0.55	1.163 up	3.676 up	0.01	15.94	unknown protein
111205	7.76	0.24	8.22	0.06	9.35	0.45	1.381 up	3.027 up	0.01	14.93	SSCRP
111236	11.54	0.11	11.37	0.30	12.71	0.11	1.124 down	2.264 up	0.00	56.14	N-acetyltransferase of bacterial origin
111245	10.37	0.03	8.88	0.08	8.27	0.24	2.789 down	4.278 down	0.00	79.09	PL8 polysaccharide lyase; distantly related to chondroitin lyases
111252	4.70	0.70	4.78	0.62	7.54	0.37	1.054 up	7.154 up	0.00	30.49	unique protein

111272	4.56	0.14	4.91	0.40	6.15	0.36	1.274 up	3.013 up	0.01	18.40	unknown protein
111289	3.04	0.25	3.12	0.25	4.37	0.06	1.051 up	2.506 up	0.00	63.08	unique protein
111304	9.31	0.17	7.60	0.12	7.04	0.19	3.260 down	4.816 down	0.00	112.84	unknown protein
111326	4.46	0.12	4.62	0.02	5.53	0.33	1.112 up	2.090 up	0.02	14.26	unknown protein
111345	8.74	0.06	8.12	0.20	10.77	0.37	1.540 down	4.093 up	0.00	62.33	unknown protein. Sordariomycetes. 1 TM
111351	12.67	0.01	12.28	0.11	11.51	0.16	1.311 down	2.230 down	0.00	55.13	DHBP_synthase.-dihydroxy--butanone -phosphate synthase
111357	12.79	0.04	12.25	0.03	10.55	0.15	1.449 down	4.725 down	0.00	298.03	FAD-dependent oxidoreductase
111362	12.83	0.16	10.07	0.14	11.54	0.56	6.778 down	2.442 down	0.01	19.41	unknown protein
111372	5.73	0.40	6.56	0.12	7.11	0.18	1.779 up	2.619 up	0.01	23.35	unique protein
111382	12.26	0.05	10.90	0.26	9.46	0.22	2.571 down	6.966 down	0.00	130.67	ankyrin
111393	2.80	0.24	2.83	0.14	5.04	0.18	1.019 up	4.733 up	0.00	138.09	unique protein
111405	3.94	0.77	2.71	0.01	6.73	0.53	2.346 down	6.898 up	0.00	42.97	unique protein
111442	10.76	0.09	7.60	0.06	7.76	0.23	8.953 down	8.042 down	0.00	202.36	Epl1/Sm1
111443	8.50	0.06	6.38	0.06	10.22	0.19	4.343 down	3.299 up	0.00	444.33	unique protein
111446	12.13	0.08	12.11	0.03	9.66	0.46	1.017 down	5.552 down	0.00	46.77	Zn2Cys6 transcriptional regulator
111449	2.35	0.02	2.50	0.13	3.82	0.10	1.113 up	2.773 up	0.00	216.42	Epl1-like protein
111451	6.40	0.06	6.65	0.23	7.93	0.50	1.188 up	2.894 up	0.02	12.59	peptidase S41
111465	2.96	0.33	5.62	0.50	4.85	0.25	6.317 up	3.691 up	0.00	34.88	unique protein
111466	4.90	0.17	4.90	0.12	6.56	0.18	1.001 up	3.149 up	0.00	97.39	Zn2Cys6 transcriptional regulator
111476	10.24	0.03	9.74	0.05	8.63	0.11	1.416 down	3.062 down	0.00	263.15	unknown protein
111495	9.92	0.09	6.34	0.28	11.58	0.56	11.978 down	3.161 up	0.00	90.27	SSCRP
111499	5.59	0.17	5.96	0.31	6.95	0.23	1.291 up	2.560 up	0.01	25.10	MRSP1/expansin-like
111502	11.44	0.24	12.32	0.27	12.94	0.19	1.838 up	2.827 up	0.00	32.00	NADH:ubiquinone oxidoreductase. NDUFB7/B18 subunit
111515	8.59	0.16	5.53	0.19	6.67	0.22	8.323 down	3.766 down	0.00	116.51	Zn2Cys6 transcriptional regulator
111527	11.39	0.03	10.52	0.10	10.33	0.31	1.836 down	2.084 down	0.02	13.25	unique protein
111564	9.65	0.01	10.65	0.11	10.87	0.18	2.001 up	2.324 up	0.00	48.42	D-tyrosyl-tRNA deacylase
111566	8.25	0.08	4.45	0.02	7.15	0.26	13.910 down	2.134 down	0.00	183.82	unique protein
111567	11.27	0.02	7.00	0.00	8.72	0.24	19.198 down	5.836 down	0.00	258.98	C2H2 transcription factor
111570	10.18	0.06	9.19	0.02	8.62	0.10	1.982 down	2.951 down	0.00	220.01	short chain dehydrogenase/reductase
111584	3.80	0.07	3.99	0.25	5.22	0.11	1.145 up	2.679 up	0.00	83.23	unknown protein

111591	8.86	0.10	8.55	0.20	6.76	0.43	1.242 down	4.288 down	0.00	31.12	unknown protein
111593	3.12	0.34	3.22	0.02	5.24	0.11	1.069 up	4.337 up	0.00	141.78	unique protein
111632	8.71	0.07	9.33	0.01	10.71	0.28	1.541 up	3.999 up	0.00	64.18	unknown protein
111638	10.42	0.03	11.79	0.00	9.41	0.16	2.574 up	2.018 down	0.00	239.32	unknown protein
111645	4.89	0.25	5.95	0.13	6.21	0.14	2.085 up	2.497 up	0.00	43.65	unique protein
111663	9.81	0.17	10.30	0.10	10.86	0.12	1.400 up	2.074 up	0.00	46.38	unknown protein
111674	2.72	0.11	2.73	0.05	4.38	0.09	1.007 up	3.142 up	0.00	356.92	unique protein
111682	8.86	0.18	8.00	0.07	7.78	0.12	1.813 down	2.116 down	0.00	49.24	unknown protein
111729	3.08	0.11	3.15	0.12	4.40	0.07	1.049 up	2.506 up	0.00	212.13	HFBs
111731	6.08	0.08	6.30	0.10	4.83	0.23	1.164 up	2.379 down	0.00	54.77	unique protein
111733	3.39	0.32	3.12	0.21	4.96	0.10	1.207 down	2.955 up	0.00	80.91	GH92 α-1.2-mannosidase
111735	5.70	0.51	5.55	0.10	6.91	0.19	1.106 down	2.314 up	0.01	22.54	unique protein
111736	8.34	0.17	9.60	0.22	9.44	0.29	2.401 up	2.150 up	0.01	15.57	unique protein
111750	10.50	0.01	10.85	0.06	14.86	0.08	1.277 up	20.527 up	0.00	3531.03	ferric reductase
111755	10.29	0.07	9.92	0.06	8.83	0.10	1.291 down	2.742 down	0.00	221.86	transcriptional regulator. unknown
111758	9.61	0.08	9.06	0.11	10.91	0.39	1.464 down	2.467 up	0.01	27.98	unknown protein
111776	9.98	0.05	10.51	0.05	8.06	0.48	1.452 up	3.775 down	0.00	35.58	Zn2Cys6 transcriptional regulator
111778	7.80	0.06	10.81	0.05	8.96	0.36	8.063 up	2.238 up	0.00	59.93	unknown protein
111788	11.50	0.09	10.82	0.07	10.41	0.06	1.608 down	2.132 down	0.00	169.70	Synaptobrevin/VAMP-like protein (VAMP71 family)
111807	6.56	0.26	7.78	0.14	5.39	0.41	2.321 up	2.247 down	0.00	32.25	unknown protein
111808	3.83	0.22	4.23	0.04	5.42	0.21	1.312 up	3.002 up	0.00	55.41	unknown protein
111811	4.13	0.03	6.53	0.16	5.70	0.28	5.260 up	2.968 up	0.00	58.70	unique protein
111827	3.82	0.03	3.77	0.04	4.92	0.18	1.035 down	2.137 up	0.00	65.08	unknown protein
111832	11.53	0.06	10.63	0.00	10.36	0.14	1.854 down	2.241 down	0.00	71.31	Isoflavone reductase superfamily protein
111833	2.31	0.11	2.28	0.06	4.09	0.11	1.015 down	3.442 up	0.00	297.59	MFS permease
111838	8.80	0.18	8.15	0.12	6.74	0.32	1.572 down	4.166 down	0.00	44.24	serin endopeptidase
111849	9.08	0.03	7.99	0.15	10.12	0.08	2.121 down	2.055 up	0.00	362.84	GH30 endo-β-1.4-xylanase XYN4
111858	3.64	0.14	3.54	0.10	5.42	0.10	1.078 down	3.423 up	0.00	278.70	methyltransferase
111861	3.37	0.15	4.22	0.40	5.21	0.31	1.803 up	3.599 up	0.01	24.81	PTH11 GPCR
111874	7.22	0.04	4.60	0.29	5.63	0.24	6.170 down	3.012 down	0.00	67.63	swollenin 2

111875	11.89	0.05	10.96	0.02	10.24	0.35	1.909 down	3.131 down	0.01	24.95	unknown protein
111884	4.89	0.03	5.37	0.29	6.49	0.26	1.391 up	3.018 up	0.00	34.10	unknown protein
111888	10.13	0.10	10.10	0.14	8.98	0.21	1.025 down	2.228 down	0.00	39.98	MFS permease
111901	6.63	0.00	6.17	0.06	5.50	0.09	1.368 down	2.178 down	0.00	180.00	unknown proteins
111915	10.02	0.20	13.43	0.08	13.79	0.23	10.684 up	13.684 up	0.00	250.57	SSCRP
111923	6.77	0.02	8.73	0.21	11.07	0.42	3.901 up	19.749 up	0.00	114.50	unknown protein
111937	10.64	0.03	11.04	0.09	11.70	0.32	1.316 up	2.075 up	0.02	13.18	unique protein
111948	5.81	0.30	5.37	0.33	7.14	0.32	1.356 down	2.520 up	0.01	25.21	unique protein
111951	13.55	0.27	9.15	0.07	12.22	0.40	21.223 down	2.518 down	0.00	95.89	unique protein
111953	9.58	0.02	9.04	0.15	8.48	0.14	1.454 down	2.142 down	0.00	49.90	GH47 α-1.2-mannosidase
111955	8.19	0.10	7.53	0.12	9.58	0.63	1.589 down	2.613 up	0.02	13.23	unknown protein. 1 TM
111956	3.59	0.33	3.61	0.12	4.98	0.37	1.014 up	2.628 up	0.01	17.72	unknown protein
111958	3.71	0.02	3.87	0.00	5.47	0.23	1.111 up	3.370 up	0.00	85.79	unknown protein
111966	4.11	0.13	3.86	0.19	6.06	0.11	1.186 down	3.869 up	0.00	241.15	short chain dehydrogenase/reductase
111968	9.32	0.16	9.30	0.04	5.66	0.61	1.011 down	12.623 down	0.00	57.43	unknown protein. only in ascomycetes
112001	11.29	0.13	10.12	0.04	9.51	0.18	2.252 down	3.446 down	0.00	90.10	unknown protein
112002	8.54	0.08	7.98	0.04	9.93	0.12	1.473 down	2.624 up	0.00	275.86	SSCRP
112028	2.97	0.13	3.47	0.11	5.19	0.12	1.416 up	4.666 up	0.00	276.85	terpene synthase
112030	3.93	0.10	3.86	0.23	5.74	0.41	1.052 down	3.490 up	0.00	30.25	unique protein
112034	2.98	0.06	2.92	0.24	4.59	0.15	1.036 down	3.052 up	0.00	106.76	unknown protein
112037	3.28	0.00	3.36	0.16	5.03	0.06	1.059 up	3.377 up	0.00	402.53	SSCRP
112049	6.57	0.18	3.30	0.57	5.20	0.23	9.679 down	2.578 down	0.00	52.54	unique protein
112055	3.33	0.43	3.42	0.16	4.50	0.12	1.064 up	2.249 up	0.01	24.94	unique protein
112064	9.97	0.29	10.06	0.12	8.92	0.20	1.064 up	2.073 down	0.01	27.56	unique protein
112080	9.62	0.04	9.86	0.14	8.44	0.08	1.180 up	2.264 down	0.00	216.08	unknown protein
112081	12.34	0.23	11.55	0.07	10.27	0.18	1.738 down	4.201 down	0.00	99.15	Methionine synthase. vitamin-B12 independent. putative
112083	6.96	0.10	5.66	0.16	8.40	0.60	2.453 down	2.723 up	0.01	23.60	HET protein
112086	3.65	0.14	5.00	0.35	6.04	0.16	2.542 up	5.252 up	0.00	87.50	unknown protein
112094	8.35	0.05	7.61	0.02	6.88	0.23	1.676 down	2.767 down	0.00	44.70	unique protein
112113	3.19	0.09	3.40	0.39	4.58	0.11	1.152 up	2.611 up	0.00	42.13	unknown protein

112124	5.10	0.35	4.82	0.27	7.39	0.40	1.219 down	4.884 up	0.00	43.13	HFBs
112125	10.30	0.11	8.50	0.08	8.58	0.43	3.475 down	3.278 down	0.01	19.58	macrophomate synthase
112126	12.76	0.00	12.29	0.09	11.62	0.01	1.388 down	2.204 down	0.00	486.22	Methylmalonate semialdehyde dehydrogenase
112129	4.67	0.11	5.00	0.61	5.71	0.13	1.254 up	2.060 up	0.03	9.43	Zn2Cys6 transcriptional regulator
112133	2.98	0.12	3.07	0.14	5.09	0.24	1.064 up	4.317 up	0.00	102.06	unknown protein
112134	9.69	0.19	9.12	0.02	7.52	0.35	1.483 down	4.514 down	0.00	45.80	Zn2Cys6 transcriptional regulator
112140	4.08	0.33	3.64	0.23	8.33	0.38	1.359 down	18.991 up	0.00	169.30	GH28 exo-polygalacturonase PGX1
112163	7.40	0.07	6.74	0.16	8.85	0.19	1.586 down	2.729 up	0.00	126.20	unknown protein in Sordariomycetes
112180	11.93	0.09	11.27	0.00	10.06	0.06	1.573 down	3.638 down	0.00	672.74	unknown protein
112193	12.23	0.12	12.27	0.10	10.93	0.51	1.031 up	2.465 down	0.02	11.03	unknown protein
112211	12.37	0.06	12.19	0.05	11.34	0.15	1.131 down	2.038 down	0.00	62.12	exonuclease. putative
112215	4.97	0.22	6.19	0.22	6.32	0.12	2.337 up	2.548 up	0.00	47.00	unique protein
112222	6.75	0.26	5.94	0.36	5.40	0.26	1.752 down	2.550 down	0.01	15.32	unknown protein
112232	3.95	0.31	4.91	0.28	5.20	0.29	1.944 up	2.375 up	0.02	12.47	unique protein
112238	3.19	0.18	3.38	0.05	4.60	0.09	1.142 up	2.660 up	0.00	151.97	unique protein
112239	13.14	0.07	5.45	0.08	6.68	0.25	206.465 down	87.701 down	0.00	888.55	unknown protein
112247	11.69	0.04	8.42	0.04	8.14	0.48	9.643 down	11.725 down	0.00	64.34	3-isopropylmalate dehydrogenase
112255	2.50	0.04	2.78	0.15	4.02	0.08	1.219 up	2.863 up	0.00	231.25	unique protein
112267	11.17	0.15	10.28	0.08	9.55	0.32	1.846 down	3.066 down	0.01	26.34	unknown protein
112275	9.78	0.01	9.65	0.04	7.91	0.16	1.093 down	3.655 down	0.00	201.85	unique protein
112276	11.70	0.15	11.19	0.22	9.19	0.15	1.427 down	5.681 down	0.00	186.68	unique protein
112281	9.64	0.02	5.55	0.26	7.48	0.26	16.952 down	4.461 down	0.00	153.11	unique protein
112285	8.59	0.20	7.09	0.08	7.20	0.30	2.821 down	2.624 down	0.01	23.83	unknown protein
112288	5.03	0.06	4.84	0.16	6.85	0.42	1.141 down	3.536 up	0.00	33.14	unknown protein
112328	12.78	0.04	12.09	0.02	10.86	0.27	1.612 down	3.779 down	0.00	60.30	peptidase M14
112330	12.52	0.08	12.27	0.09	11.04	0.42	1.190 down	2.780 down	0.01	17.11	transcriptional regulator. putative
112338	4.14	0.56	4.33	0.15	7.00	0.86	1.140 up	7.254 up	0.01	14.93	unique protein
112369	2.52	0.07	2.83	0.21	4.62	0.35	1.242 up	4.281 up	0.00	45.88	unique protein
112370	3.56	0.06	3.65	0.22	4.93	0.15	1.069 up	2.586 up	0.00	74.64	unique protein
112386	3.25	0.22	3.27	0.41	5.38	0.23	1.011 up	4.348 up	0.00	59.21	unknown protein

112387	4.89	0.28	4.43	0.30	6.19	0.57	1.376 down	2.463 up	0.02	10.83	unknown protein with WD repeats
112390	14.26	0.08	13.69	0.05	12.74	0.24	1.487 down	2.876 down	0.00	48.53	unknown protein
112392	4.90	0.08	4.95	0.22	6.93	0.37	1.037 up	4.085 up	0.00	43.65	GH11 endo-ß-1.4-xylanase XYN5
112396	4.41	0.33	4.98	0.32	7.15	0.16	1.484 up	6.680 up	0.00	104.95	unique secreted protein
112398	8.75	0.22	7.77	0.02	7.14	0.17	1.968 down	3.059 down	0.00	65.58	unique protein
112400	10.88	0.11	10.43	0.18	9.66	0.18	1.362 down	2.321 down	0.00	38.43	unique protein
112401	11.01	0.04	10.72	0.23	9.46	0.24	1.224 down	2.921 down	0.00	45.13	Zn2Cys6 transcriptional regulator
112427	2.29	0.12	2.40	0.03	3.95	0.09	1.075 up	3.158 up	0.00	319.84	unique protein
112439	14.58	0.08	13.73	0.05	12.04	0.20	1.813 down	5.854 down	0.00	188.11	unknown protein
112441	9.99	0.14	10.78	0.01	8.93	0.18	1.728 up	2.077 down	0.00	101.71	Vacuolar sorting protein Vps52
112459	7.67	0.13	7.43	0.28	6.10	0.48	1.180 down	2.970 down	0.02	13.73	unique protein
112477	4.05	0.45	4.35	0.13	5.81	0.36	1.228 up	3.370 up	0.01	21.45	unknown protein
112478	6.50	0.07	6.10	0.03	10.14	0.85	1.325 down	12.441 up	0.00	34.31	unknown protein. only in A. oryzae
112490	7.17	0.22	6.82	0.14	5.71	0.39	1.276 down	2.755 down	0.01	16.31	unknown protein
112494	3.81	0.17	3.90	0.36	5.18	0.14	1.066 up	2.581 up	0.00	39.68	unknown protein
112496	9.60	0.06	10.00	0.15	7.73	0.22	1.319 up	3.655 down	0.00	131.96	heterocompatibility domain protein
112502	11.83	0.01	10.86	0.04	10.06	0.20	1.955 down	3.398 down	0.00	90.68	unknown protein
112514	6.13	0.40	6.65	0.08	7.33	0.20	1.440 up	2.308 up	0.01	17.69	unique protein
112518	3.54	0.12	3.73	0.42	5.68	0.18	1.138 up	4.409 up	0.00	72.78	unique protein
112519	10.33	0.08	9.99	0.13	8.90	0.32	1.264 down	2.695 down	0.01	24.53	vacuolar endopolyphosphatase
112520	8.73	0.18	9.13	0.11	10.19	0.15	1.314 up	2.734 up	0.00	73.31	unique protein
112523	6.38	0.15	6.27	0.22	7.86	0.65	1.075 down	2.781 up	0.03	8.80	unknown protein
112532	8.32	0.03	8.36	0.06	7.01	0.40	1.029 up	2.471 down	0.01	18.22	bHLH transcriptional regulator
112535	4.79	0.13	6.23	0.21	6.01	0.31	2.712 up	2.327 up	0.01	18.14	unique protein
112536	11.57	0.01	10.77	0.25	10.19	0.30	1.749 down	2.604 down	0.01	19.50	unknown protein
112538	6.90	0.09	5.41	0.02	7.95	0.16	2.815 down	2.072 up	0.00	247.82	C2H2 transcription factor
112539	6.70	0.02	5.29	0.14	5.51	0.27	2.661 down	2.286 down	0.01	25.71	Zn2Cys6 transcriptional regulator
112551	10.57	0.05	9.67	0.07	8.97	0.15	1.870 down	3.024 down	0.00	110.40	unknown protein
112559	2.56	0.11	2.73	0.01	4.30	0.15	1.126 up	3.351 up	0.00	172.72	unknown protein
112560	10.09	0.28	9.71	0.05	8.35	0.20	1.298 down	3.333 down	0.00	60.94	Zn2Cys6 transcriptional regulator

112563	3.53	0.05	3.64	0.03	5.96	0.28	1.078 up	5.371 up	0.00	114.40	α-ketoglutarate dependent (FeII) dioxygenase
112578	4.55	0.30	4.19	0.22	5.65	0.21	1.287 down	2.136 up	0.00	30.96	unknown protein
112580	2.76	0.04	2.78	0.05	4.54	0.19	1.015 up	3.443 up	0.00	143.16	unknown protein
112596	11.18	0.15	10.64	0.08	8.98	0.11	1.448 down	4.583 down	0.00	291.51	flavoprotein monooxygenase
112602	2.47	0.06	2.36	0.01	3.85	0.03	1.078 down	2.595 up	0.00	1398.90	unknown protein
112603	2.26	0.20	2.42	0.10	3.71	0.06	1.121 up	2.732 up	0.00	153.62	unique protein
112604	2.29	0.17	2.36	0.06	3.80	0.10	1.049 up	2.849 up	0.00	171.94	unique protein
112605	2.16	0.17	2.21	0.09	3.54	0.09	1.033 up	2.608 up	0.00	154.51	unique protein
112629	5.06	0.64	5.42	0.08	6.60	0.49	1.284 up	2.916 up	0.04	8.46	unique protein
112631	5.90	0.15	6.80	0.25	7.58	0.20	1.875 up	3.216 up	0.00	46.98	unique protein
112638	3.49	0.27	3.57	0.24	5.04	0.34	1.056 up	2.926 up	0.01	23.73	unknown protein
112649	2.20	0.08	2.44	0.23	3.94	0.06	1.184 up	3.356 up	0.00	194.92	unknown protein
112651	2.58	0.08	2.55	0.05	3.70	0.03	1.020 down	2.173 up	0.00	513.28	unknown protein
112656	8.57	0.03	7.53	0.25	7.27	0.18	2.052 down	2.474 down	0.00	36.25	unique protein
112665	9.82	0.02	7.25	0.16	7.18	0.28	5.943 down	6.267 down	0.00	99.23	GCN5-related N-acetyltransferase
112675	2.43	0.02	2.45	0.24	4.53	0.25	1.007 up	4.269 up	0.00	90.23	unknown protein
112676	1.98	0.18	2.08	0.14	3.48	0.10	1.074 up	2.829 up	0.00	134.89	unique protein
112678	2.42	0.07	2.46	0.07	3.65	0.15	1.030 up	2.347 up	0.00	90.78	unique protein
112679	2.45	0.08	2.47	0.13	4.09	0.10	1.014 up	3.120 up	0.00	247.52	unique protein
112683	2.21	0.14	2.28	0.19	3.59	0.07	1.047 up	2.594 up	0.00	124.38	unknown protein
112685	2.53	0.02	2.72	0.18	4.30	0.10	1.141 up	3.407 up	0.00	214.57	unknown protein
112688	2.72	0.11	2.84	0.13	4.16	0.41	1.086 up	2.713 up	0.01	17.91	unique protein
112689	2.52	0.26	2.59	0.35	4.34	0.15	1.054 up	3.548 up	0.00	62.28	unknown protein
112695	3.71	0.40	3.73	0.24	5.01	0.13	1.011 up	2.453 up	0.00	30.54	SSCRP
119534	13.98	0.01	13.69	0.06	12.64	0.17	1.220 down	2.526 down	0.00	79.21	unique protein
119552	8.94	0.05	9.19	0.08	10.38	0.07	1.189 up	2.725 up	0.00	383.62	unique protein
119556	11.42	0.03	9.96	0.01	10.03	0.55	2.752 down	2.620 down	0.04	8.39	unknown protein. secreted
119568	11.23	0.17	10.96	0.04	9.39	0.28	1.206 down	3.590 down	0.00	55.13	unknown protein
119607	12.48	0.04	11.67	0.09	10.95	0.10	1.762 down	2.907 down	0.00	191.47	unknown protein
119619	10.33	0.13	8.28	0.15	8.86	0.44	4.165 down	2.780 down	0.01	18.34	unknown protein

119620	11.04	0.02	10.62	0.11	9.84	0.30	1.343 down	2.295 down	0.01	18.47	Zn2Cys6 transcriptional regulator
119710	12.91	0.01	10.98	0.08	10.81	0.13	3.821 down	4.284 down	0.00	257.88	MFS multidrug transporter
119723	12.63	0.01	12.57	0.29	11.60	0.28	1.044 down	2.050 down	0.01	15.65	NSF attachment protein Sec17/alpha-SNAP
119789	13.09	0.12	12.64	0.18	11.62	0.49	1.369 down	2.762 down	0.02	10.73	MFS permease
119800	11.17	0.04	12.14	0.04	12.17	0.10	1.953 up	2.000 up	0.00	113.19	unknown protein
119805	13.14	0.20	13.44	0.16	14.20	0.29	1.232 up	2.096 up	0.02	14.34	HFBs
119806	12.18	0.05	12.97	0.00	13.60	0.10	1.723 up	2.677 up	0.00	200.54	unknown protein. only in Gibberella. Magnaporthe and Chaetomium
119816	11.20	0.12	11.84	0.10	12.45	0.36	1.562 up	2.392 up	0.02	12.97	unknown protein
119823	13.22	0.04	12.51	0.15	11.79	0.19	1.635 down	2.686 down	0.00	54.82	SAM-dependent methyltransferases
119825	3.61	0.19	3.93	0.00	5.31	0.49	1.242 up	3.237 up	0.01	15.96	unknown protein
119826	11.37	0.25	10.07	0.03	7.13	0.62	2.461 down	18.872 down	0.00	55.71	C2H2 transcriptional regulator
119839	3.87	0.60	3.02	0.17	5.44	0.35	1.805 down	2.966 up	0.00	29.04	cyanamide hydratase
119856	12.24	0.22	12.23	0.02	11.22	0.23	1.007 down	2.034 down	0.01	25.56	E3 ubiquitin-protein ligase/Putative upstream regulatory element binding protein
119857	7.15	0.06	11.56	0.00	10.25	0.26	21.231 up	8.567 up	0.00	258.13	unique protein
119860	10.86	0.08	11.37	0.02	9.71	0.35	1.423 up	2.222 down	0.01	27.66	unknown protein
119864	9.25	0.02	6.08	0.25	6.95	0.32	9.027 down	4.933 down	0.00	74.84	unknown protein
119876	11.86	0.20	12.00	0.07	12.88	0.12	1.103 up	2.027 up	0.00	50.03	aspartyl protease
119895	11.86	0.07	11.91	0.13	10.61	0.32	1.040 up	2.374 down	0.01	24.19	unknown protein
119896	11.85	0.01	11.47	0.04	9.97	0.28	1.306 down	3.683 down	0.00	61.71	malic enzyme
119902	12.69	0.11	10.43	0.05	11.41	0.25	4.795 down	2.418 down	0.00	65.44	unique protein
119960	9.28	0.14	9.61	0.23	10.48	0.28	1.254 up	2.295 up	0.01	18.07	unique protein
119963	12.77	0.05	13.04	0.09	13.80	0.05	1.202 up	2.031 up	0.00	244.10	HFBs
119989	13.80	0.03	13.75	0.12	14.81	0.14	1.034 down	2.015 up	0.00	74.14	HFB2
120008	12.11	0.28	13.17	0.14	13.12	0.27	2.084 up	2.017 up	0.02	12.86	GMC oxidoreductase
120031	12.53	0.11	13.82	0.02	14.12	0.30	2.450 up	3.009 up	0.00	31.30	unknown protein
120060	12.38	0.08	12.22	0.10	11.37	0.21	1.112 down	2.008 down	0.00	29.67	unknown protein with pleckstrin like domain
120079	11.02	0.19	10.35	0.16	9.64	0.26	1.595 down	2.608 down	0.01	25.40	mitochondrial acetoacetyl-CoA thiolase-like protein
120088	10.22	0.12	10.60	0.10	12.10	0.31	1.302 up	3.685 up	0.00	46.15	cytosolic asparaginyl-tRNA synthetase. required for protein synthesis. catalyzes the sp
120106	11.44	0.10	12.44	0.28	12.85	0.26	1.999 up	2.648 up	0.01	22.23	Isopentenyl diphosphate:dimethylallyl diphosphate isomerase
120113	11.19	0.00	10.66	0.12	10.15	0.18	1.444 down	2.062 down	0.00	34.96	unknown protein

120125	12.13	0.12	11.78	0.20	10.85	0.14	1.277 down	2.433 down	0.00	56.09	unknown protein
120160	12.49	0.09	13.00	0.37	13.74	0.10	1.428 up	2.378 up	0.00	31.86	unknown protein
120172	12.14	0.06	11.77	0.16	11.04	0.41	1.285 down	2.139 down	0.04	8.31	flavodoxin and radical SAM domain protein
120198	13.70	0.17	13.30	0.01	12.32	0.18	1.323 down	2.604 down	0.00	57.56	glycosyl transferase. family 35. glycogen phosphorylase 1
120229	7.18	0.12	5.04	0.56	13.25	0.80	4.392 down	67.461 up	0.00	117.81	GH10 endo-ß-1.4-xylanase XYN3
120248	10.10	0.11	8.90	0.03	9.06	0.44	2.296 down	2.051 down	0.04	7.61	unknown protein
120260	8.99	0.01	9.28	0.06	10.18	0.39	1.219 up	2.267 up	0.02	12.22	ribosomal protein L24e. putative
120272	11.02	0.12	10.84	0.07	9.96	0.14	1.138 down	2.083 down	0.00	59.65	64 kDa mitochondrial NADH dehydrogenase. putative
120294	5.83	0.19	6.52	0.41	6.98	0.07	1.606 up	2.216 up	0.01	20.21	unknown protein
120311	10.57	0.15	8.77	0.14	12.83	0.22	3.486 down	4.803 up	0.00	321.21	unique protein
120312	10.86	0.21	6.74	0.10	14.81	0.26	17.338 down	15.462 up	0.00	846.37	GH5 endo-β-1.4-glucanase EGL2/CEL5a
120326	10.77	0.15	10.21	0.05	8.72	0.29	1.472 down	4.129 down	0.00	59.77	unknown protein
120351	9.69	0.17	10.45	0.11	10.71	0.14	1.691 up	2.020 up	0.00	36.34	unknown protein
120363	7.04	0.14	7.25	0.16	5.93	0.56	1.151 up	2.172 down	0.04	7.72	C2H2 transcriptional regulator
120365	11.07	0.12	10.04	0.11	9.78	0.33	2.044 down	2.434 down	0.01	15.71	BZIP transcriptional regulator
120370	12.72	0.05	13.33	0.05	13.74	0.02	1.519 up	2.024 up	0.00	501.09	unknown protein
120371	13.55	0.12	6.22	0.16	10.49	0.85	161.771 down	8.375 down	0.00	61.55	Catalase
120381	11.41	0.09	12.85	0.06	8.69	0.45	2.719 up	6.564 down	0.00	104.43	unique protein
120415	10.57	0.02	12.80	0.11	11.67	0.11	4.687 up	2.154 up	0.00	273.94	unknown protein
120451	6.67	0.10	7.57	0.07	8.38	0.30	1.872 up	3.275 up	0.00	34.67	unknown protein
120473	8.00	0.01	8.22	0.08	9.09	0.28	1.170 up	2.137 up	0.01	21.12	Dihydrolipoamide transacylase (alpha-keto acid dehydrogenase E2 subunit)
120475	13.16	0.02	12.41	0.02	11.91	0.16	1.685 down	2.386 down	0.00	65.47	C2H2 transcriptional regulator
120486	9.09	0.09	9.79	0.01	11.21	0.24	1.624 up	4.346 up	0.00	91.20	auxiliary protein of DNA polymerase delta
120489	12.66	0.06	11.43	0.06	11.08	0.04	2.331 down	2.972 down	0.00	698.58	unknown protein
120504	9.93	0.06	8.58	0.07	13.18	0.28	2.557 down	9.472 up	0.00	323.72	unique protein
120571	9.68	0.17	10.36	0.12	12.03	0.55	1.596 up	5.082 up	0.01	22.57	pre-rRNA-processing protein PNO1
120597	14.15	0.09	12.62	0.07	11.06	0.18	2.887 down	8.540 down	0.00	286.58	C2H2 transcriptional regulator
120623	14.26	0.09	13.16	0.07	13.05	0.30	2.154 down	2.316 down	0.01	18.21	unknown protein
120688	5.26	0.28	5.84	0.19	6.66	0.28	1.495 up	2.635 up	0.01	20.20	unique protein
120697	11.88	0.15	13.65	0.12	14.05	0.18	3.428 up	4.518 up	0.00	125.05	SSCRP

120702	9.03	0.30	9.29	0.03	11.27	0.08	1.202 up	4.743 up	0.00	206.28	unknown protein
120715	11.33	0.17	10.27	0.17	10.31	0.23	2.085 down	2.028 down	0.01	18.08	Zn2Cys6 transcriptional regulator
120733	10.99	0.09	11.55	0.01	12.86	0.21	1.470 up	3.649 up	0.00	98.31	mitochondrial import inner membrane translocase subunit TIM13 / Zn-finger. Tim10/[illegible]
120749	13.19	0.01	11.91	0.08	14.50	0.06	2.428 down	2.475 up	0.00	1411.91	GH1 ß-glucosidase BGL2/CEL1a
120751	12.33	0.09	11.32	0.08	11.18	0.13	2.015 down	2.226 down	0.00	75.50	unknown protein
120752	11.71	0.08	11.23	0.02	9.96	0.17	1.396 down	3.362 down	0.00	129.33	K+ transporter Trk1
120784	12.80	0.12	12.12	0.09	11.33	0.12	1.608 down	2.766 down	0.00	108.24	cell wall mannoprotein
120791	11.95	0.07	12.45	0.25	13.03	0.35	1.410 up	2.105 up	0.03	9.32	unknown protein
120823	13.71	0.13	14.20	0.12	14.79	0.03	1.409 up	2.117 up	0.00	113.10	cell wall mannoprotein
120828	11.92	0.11	12.23	0.43	13.21	0.36	1.240 up	2.442 up	0.02	11.44	unknown protein
120872	11.24	0.11	9.96	0.01	9.47	0.44	2.434 down	3.420 down	0.01	17.82	GCN5 N-acetyltransferase
120873	4.82	0.29	4.73	0.18	8.28	0.26	1.069 down	11.000 up	0.00	191.27	GH71 α-1 3-glucanase
120889	11.83	0.28	12.17	0.07	10.75	0.38	1.271 up	2.110 down	0.01	15.46	cytochrome P450. putative
120911	12.54	0.03	10.88	0.12	9.44	0.18	3.158 down	8.588 down	0.00	300.30	short chain dehydrogenase/reductase
120926	6.16	0.00	4.14	0.41	7.47	0.56	4.050 down	2.468 up	0.00	33.19	unique protein
120927	5.16	0.18	6.13	0.16	7.84	0.76	1.963 up	6.440 up	0.01	14.82	ankyrin containing protein
120931	7.20	0.15	10.08	0.13	10.84	0.20	7.346 up	12.502 up	0.00	275.08	unknown protein
120961	8.65	0.10	3.31	0.15	13.06	0.69	40.524 down	21.244 up	0.00	218.45	GH61 polysaccharide monooxygenase CEL61b
120968	6.19	0.65	4.64	0.42	9.96	0.23	2.928 down	13.631 up	0.00	143.64	Copper chaperone for superoxide dismutase
120969	10.53	0.00	11.16	0.08	11.64	0.04	1.548 up	2.148 up	0.00	354.23	NADPH oxidase regulator NoxR
120978	13.23	0.03	13.39	0.29	14.24	0.14	1.121 up	2.011 up	0.00	29.96	large ribosomal subunit (protein L34e).
120981	11.07	0.07	10.66	0.03	9.43	0.29	1.324 down	3.117 down	0.00	42.51	magnesium and cobalt transporter CorA
120993	4.37	0.44	3.99	0.15	6.23	0.22	1.306 down	3.637 up	0.00	58.22	unknown protein
121042	8.57	0.50	9.87	0.05	11.49	0.47	2.457 up	7.562 up	0.00	32.25	unknown protein
121058	3.75	0.31	3.92	0.01	4.92	0.09	1.119 up	2.244 up	0.00	49.28	unknown protein
121095	11.22	0.04	10.98	0.10	9.89	0.26	1.186 down	2.528 down	0.00	36.27	cell cycle control protein (Cwf19). putative
121107	11.66	0.05	11.94	0.13	10.55	0.20	1.213 up	2.152 down	0.00	56.60	Zn2Cys6 transcriptional regulator
121121	9.74	0.05	10.22	0.04	10.88	0.38	1.399 up	2.196 up	0.03	10.51	Zn2Cys6 transcriptional regulator
121127	10.11	0.01	6.03	0.25	12.36	0.46	17.003 down	4.756 up	0.00	189.98	GH3 β-xylosidase BXL1
121130	8.65	0.16	8.68	0.12	7.54	0.44	1.021 up	2.166 down	0.03	10.48	Zn2Cys6 transcriptional regulator

121135	2.40	0.04	3.51	0.56	3.83	0.10	2.155 up	2.695 up	0.01	20.25	SSCRP
121171	8.76	0.21	9.82	0.07	9.89	0.37	2.084 up	2.196 up	0.03	9.86	replication fork protection component Swi3
121187	11.13	0.12	9.56	0.17	9.66	0.12	2.968 down	2.772 down	0.00	98.83	unknown protein
121203	11.80	0.02	11.19	0.14	10.77	0.24	1.527 down	2.032 down	0.01	18.13	unknown protein
121226	10.37	0.15	11.73	0.03	11.46	0.47	2.555 up	2.130 up	0.04	7.90	unknown protein
121230	12.08	0.04	12.21	0.10	13.33	0.32	1.100 up	2.390 up	0.01	22.86	unique protein
121294	11.82	0.19	11.08	0.13	10.59	0.29	1.670 down	2.337 down	0.01	16.22	glucan endo-1.3(4)-β-D-glucosidase
121295	13.13	0.04	12.85	0.24	11.98	0.16	1.210 down	2.214 down	0.00	37.63	Dolichol kinase Sec59
121308	12.31	0.04	13.04	0.22	13.37	0.13	1.666 up	2.084 up	0.00	37.60	PutA delta-1-pyrroline-5-carboxylate dehydrogenase
121315	4.02	0.48	4.44	0.05	5.78	0.25	1.335 up	3.384 up	0.00	29.29	unknown protein
121337	5.24	0.27	4.61	0.24	6.26	0.34	1.552 down	2.022 up	0.01	20.66	unknown protein
121372	12.83	0.02	12.95	0.04	14.24	0.04	1.084 up	2.660 up	0.00	1177.99	60S ribosomal protein L38 by homology with corresponding proteins in other fungi an
121397	12.35	0.37	12.30	0.06	13.39	0.19	1.036 down	2.050 up	0.01	22.62	Sec61alpha subunit
121405	12.11	0.10	10.11	0.02	8.67	0.26	4.002 down	10.879 down	0.00	190.87	4-aminobutyrate aminotransferase
121412	10.15	0.00	12.32	0.08	11.64	0.14	4.497 up	2.811 up	0.00	204.64	Zn2Cys6 transcriptional regulator
121415	8.47	0.06	12.02	0.13	13.35	0.16	11.737 up	29.400 up	0.00	834.80	Zn2Cys6 transcriptional regulator
121417	4.74	0.18	4.32	0.56	5.95	0.17	1.340 down	2.317 up	0.01	24.46	unknown protein with fasciclin domain
121439	12.27	0.22	10.67	0.07	10.88	0.30	3.040 down	2.622 down	0.01	25.39	unknown protein
121441	12.83	0.05	13.11	0.36	11.10	0.22	1.208 up	3.323 down	0.00	63.12	carbounknown proteinlic acid transporter
121464	12.11	0.01	12.35	0.07	13.80	0.14	1.181 up	3.226 up	0.00	186.05	GcvHGlycine cleavage system H protein (lipoate-binding)
121474	12.36	0.07	11.85	0.09	10.33	0.39	1.420 down	4.098 down	0.00	35.59	Zn2Cys6 transcriptional regulator
121486	11.63	0.07	7.94	0.18	8.78	1.35	12.952 down	7.206 down	0.05	7.13	unknown protein
121495	4.55	0.24	3.80	0.05	7.79	0.18	1.677 down	9.463 up	0.00	430.05	Vacuolar proteinase B (yscB). a serine protease of the subtilisin family
121498	12.43	0.09	8.82	0.01	10.99	0.27	12.234 down	2.704 down	0.00	152.30	Phosphatidylserine decarboxylase
121516	13.61	0.04	13.96	0.04	14.70	0.09	1.279 up	2.128 up	0.00	148.78	histone H2B
121579	11.15	0.04	11.75	0.13	12.18	0.15	1.519 up	2.047 up	0.00	43.56	unknown protein
121594	6.92	0.04	4.35	0.39	5.42	0.14	5.975 down	2.831 down	0.00	78.88	unique protein
121602	12.22	0.03	11.99	0.07	10.88	0.09	1.168 down	2.527 down	0.00	249.24	Zn2Cys6 transcriptional regulator
121608	11.32	0.18	10.10	0.03	8.39	0.28	2.331 down	7.628 down	0.00	111.92	MFS permease
121638	11.27	0.14	9.76	0.04	10.16	0.38	2.859 down	2.162 down	0.02	13.96	unique protein

121664	7.66	0.12	7.63	0.20	8.93	0.51	1.022 down	2.411 up	0.03	9.97	Glutamate decarboxylase and related proteins
121693	12.49	0.18	11.28	0.00	10.92	0.14	2.324 down	2.982 down	0.00	91.88	Glutathione-dependent formaldehyde-activating. GFA
121735	10.35	0.09	12.11	0.07	12.74	0.46	3.392 up	5.256 up	0.00	30.06	GH3 β-glucosidase CEL3b
121741	12.65	0.02	13.06	0.27	13.67	0.16	1.323 up	2.025 up	0.01	25.48	Ribosomal protein S7
121743	12.98	0.20	13.10	0.04	11.76	0.59	1.088 up	2.331 down	0.04	7.69	Mitochondrial succinate/fumarate antiporter
121746	10.83	0.07	11.79	0.14	12.79	0.48	1.952 up	3.896 up	0.01	18.19	GH55 exo-1 3-β-glucanase GLUC78
121773	10.87	0.03	11.79	0.11	9.81	0.57	1.896 up	2.074 down	0.02	13.67	unknown protein
121785	10.15	0.02	5.84	0.13	9.01	0.21	19.861 down	2.194 down	0.00	357.38	ATP-dependent RNA helicase
121800	13.18	0.10	12.38	0.13	11.86	0.17	1.748 down	2.510 down	0.00	52.17	stomatin-like protein
121804	10.73	0.07	11.02	0.01	12.03	0.23	1.225 up	2.470 up	0.00	42.84	Ribosomal protein S15.
121824	11.53	0.16	11.37	0.18	12.87	0.30	1.118 down	2.540 up	0.00	31.83	ATP citrate lyase. alpha subunit
121864	11.79	0.12	11.70	0.10	10.16	0.30	1.059 down	3.088 down	0.00	42.20	unknown protein
121870	10.48	0.16	8.58	0.04	9.20	0.13	3.727 down	2.435 down	0.00	128.32	unknown protein
121877	10.63	0.07	8.40	0.32	8.74	0.09	4.705 down	3.710 down	0.00	113.70	epoxide hydrolase
121883	10.62	0.25	13.39	0.20	12.84	0.23	6.827 up	4.673 up	0.00	89.26	unique secreted protein
121906	12.31	0.11	12.81	0.11	13.33	0.18	1.414 up	2.027 up	0.00	28.94	40s ribosomal protein S14 (S11 family).
121944	13.08	0.04	12.90	0.02	11.98	0.23	1.128 down	2.144 down	0.00	32.29	serine/threonine protein kinase
121948	11.13	0.07	11.98	0.33	12.74	0.18	1.810 up	3.070 up	0.00	42.02	signal peptidase spc12
121968	11.79	0.07	11.02	0.01	10.64	0.20	1.714 down	2.219 down	0.00	35.18	serine peptidase S28
121989	12.21	0.01	12.04	0.12	11.05	0.22	1.126 down	2.243 down	0.00	38.75	oxalate decarboxylase
122007	9.81	0.02	11.27	0.00	12.07	0.12	2.737 up	4.789 up	0.00	366.67	unique protein
122040	12.31	0.12	12.55	0.09	13.34	0.08	1.178 up	2.036 up	0.00	102.68	ribosomal protein L31e.
122048	11.56	0.05	12.03	0.37	13.40	0.28	1.382 up	3.580 up	0.00	36.35	Sec61 beta subunit
122081	8.69	0.23	7.09	0.06	13.76	0.40	3.019 down	33.524 up	0.00	332.44	GH7 Endo-β-1.4-glucanase EGL1/CEL7b
122089	10.66	0.22	7.76	0.24	9.05	0.47	7.444 down	3.053 down	0.01	26.94	unknown protein
122091	12.42	0.00	11.60	0.00	11.04	0.04	1.767 down	2.592 down	0.00	1206.45	phospholipase of papatin-family
122096	5.80	0.30	6.49	0.16	8.68	0.50	1.613 up	7.382 up	0.00	38.73	unknown protein
122102	9.89	0.02	9.79	0.03	8.85	0.20	1.067 down	2.049 down	0.00	42.05	unknown protein
122104	10.48	0.21	9.95	0.23	9.24	0.29	1.447 down	2.368 down	0.01	16.05	Xanthine/uracil permease family
122113	13.45	0.10	13.78	0.01	14.53	0.08	1.257 up	2.126 up	0.00	145.86	unknown protein

122127	13.76	0.11	14.42	0.06	14.78	0.25	1.574 up	2.024 up	0.01	16.94	SSCRP
122140	12.07	0.21	11.95	0.26	10.69	0.49	1.085 down	2.592 down	0.03	10.38	unique protein
122160	9.13	0.18	10.01	0.12	6.87	0.29	1.845 up	4.787 down	0.00	128.41	Heat shock proteinwith DnaJ domain
122169	11.58	0.16	11.30	0.08	10.57	0.20	1.214 down	2.011 down	0.01	27.05	Histidine kinase. part of a two component signal transduction system
122212	12.50	0.13	12.15	0.11	11.37	0.25	1.276 down	2.188 down	0.01	22.84	vacuolar protein-sorting protein BRO1
122240	13.30	0.03	13.09	0.07	12.16	0.24	1.159 down	2.210 down	0.00	29.68	Carnitine o-acyltransferase
122278	9.28	0.10	9.14	0.06	10.33	0.18	1.099 down	2.065 up	0.00	54.12	U3 small nucleolar RNA associated protein (SOF1)
122284	14.05	0.07	13.54	0.05	12.66	0.09	1.420 down	2.615 down	0.00	220.23	vel1. velvet protein
122293	11.06	0.08	11.01	0.09	9.58	0.16	1.035 down	2.797 down	0.00	118.68	unknown protein
122350	12.43	0.05	11.98	0.04	10.61	0.21	1.366 down	3.530 down	0.00	94.48	Glutamate decarboxylase
122363	8.91	0.06	7.44	0.07	10.23	0.31	2.768 down	2.497 up	0.00	89.98	Hsp26/Hsp42
122366	10.77	0.13	10.55	0.04	9.69	0.19	1.161 down	2.117 down	0.00	38.05	unknown protein
122374	11.12	0.03	13.66	0.05	13.94	0.43	5.802 up	7.071 up	0.00	49.66	MRSP1/expansin-like
122422	9.27	0.13	12.00	0.07	11.46	0.21	6.607 up	4.563 up	0.00	143.26	SSCRP
122448	7.53	0.06	7.88	0.04	10.44	0.07	1.273 up	7.482 up	0.00	1764.05	C2H2 transcription factor
122499	8.17	0.03	7.31	0.51	9.71	0.16	1.816 down	2.904 up	0.00	63.03	unknown protein
122505	12.45	0.04	10.91	0.08	10.41	0.09	2.914 down	4.134 down	0.00	473.05	aryl-alcohol dehydrogenase
122526	6.72	0.12	6.77	0.17	9.27	0.26	1.039 up	5.874 up	0.00	126.86	unknown protein
122529	10.64	0.12	11.90	0.23	11.96	0.50	2.395 up	2.493 up	0.04	7.62	S1/P1 Nuclease
122541	12.54	0.01	12.30	0.07	10.99	0.21	1.175 down	2.915 down	0.00	76.12	C2H2 transcriptional regulator
122556	7.37	0.20	10.21	0.23	11.18	0.90	7.167 up	13.985 up	0.01	19.08	Short-chain dehydrogenase/reductase
122567	13.64	0.27	12.79	0.04	12.57	0.21	1.795 down	2.102 down	0.01	19.33	unknown protein
122572	13.14	0.01	13.00	0.18	14.35	0.10	1.105 down	2.299 up	0.00	129.62	hsp70 family protein
122579	9.39	0.06	11.87	0.05	11.69	0.44	5.546 up	4.905 up	0.00	36.15	unknown protein. 4TM
122614	11.35	0.16	10.20	0.07	9.58	0.31	2.211 down	3.394 down	0.00	31.82	unique protein
122629	7.92	0.12	9.97	0.01	9.72	0.49	4.133 up	3.481 up	0.01	18.37	unique protein
122745	8.89	0.07	9.77	0.22	10.58	0.07	1.845 up	3.234 up	0.00	142.63	2-oxoisovalerate dehydrogenase subunit beta. putative
122778	12.17	0.04	9.23	0.02	10.73	0.07	7.686 down	2.730 down	0.00	1319.23	short chain dehydrogenase/reductase
122780	11.31	0.04	13.46	0.10	13.91	0.27	4.450 up	6.086 up	0.00	99.32	GH28 exo-rhamnogalacturonase RGX1
122795	9.60	0.26	8.99	0.10	8.15	0.31	1.517 down	2.720 down	0.01	19.98	PTH11 GPCR

122813	10.24	0.11	11.33	0.01	12.23	0.44	2.128 up	3.983 up	0.01	22.96	unique protein
122819	10.36	0.14	11.24	0.18	12.30	0.27	1.837 up	3.820 up	0.00	49.29	DNA replication complex GINS protein PSF3
122820	8.77	0.14	4.27	0.15	7.06	0.88	22.692 down	3.281 down	0.01	22.01	Kynurenine aminotransferase. glutamine transaminase K
122824	6.85	0.25	3.43	0.13	11.99	0.62	10.764 down	35.206 up	0.00	212.69	PTH11 GPCR
122825	10.74	0.09	9.70	0.06	8.79	0.32	2.051 down	3.847 down	0.00	40.84	unknown protein
122870	10.96	0.12	12.03	0.03	12.80	0.38	2.089 up	3.578 up	0.01	24.87	cell wall protein. CwpA
122889	8.01	0.14	2.83	0.17	6.59	0.33	36.228 down	2.689 down	0.00	195.06	unknown protein
122941	9.63	0.11	8.78	0.01	11.38	0.25	1.806 down	3.349 up	0.00	129.57	unique secreted protein with CFEM domain
122974	11.25	0.14	10.95	0.01	10.07	0.24	1.233 down	2.272 down	0.00	29.73	unknown protein
122992	9.74	0.08	8.52	0.10	11.09	0.23	2.333 down	2.545 up	0.00	126.36	GT 31 ß-glycosyltransferase
122994	12.28	0.17	11.93	0.00	11.25	0.11	1.279 down	2.043 down	0.00	61.14	unknown protein
122995	8.00	0.04	8.16	0.17	6.75	0.55	1.115 up	2.386 down	0.03	9.42	unknown protein
123009	12.53	0.02	14.10	0.06	13.90	0.23	2.980 up	2.582 up	0.00	48.38	glutamine synthetase
123019	11.07	0.05	10.70	0.09	9.89	0.20	1.298 down	2.272 down	0.00	41.46	unknown protein
123029	13.53	0.10	13.11	0.20	10.84	0.35	1.339 down	6.480 down	0.00	75.95	Cu2+/Zn2+ superoxide dismutase SOD1
123039	12.74	0.09	12.70	0.20	13.93	0.06	1.031 down	2.278 up	0.00	130.26	HFBs
123079	11.06	0.07	13.99	0.02	14.34	0.22	7.591 up	9.716 up	0.00	244.91	short chain dehydrogenase/reductase
123084	9.38	0.02	8.38	0.11	8.34	0.27	1.995 down	2.052 down	0.01	16.78	chloroperoxidase
123086	6.32	0.11	6.77	0.12	13.89	0.15	1.367 up	190.175 up	0.00	2760.75	SSCRP
123095	6.80	0.30	8.33	0.03	5.43	0.42	2.880 up	2.588 down	0.00	46.56	unique protein
123120	10.85	0.28	11.11	0.07	12.01	0.18	1.200 up	2.241 up	0.00	30.89	unknown protein. 8 TM
123149	9.34	0.07	7.99	0.34	7.72	0.21	2.559 down	3.075 down	0.00	36.14	unknown protein
123180	10.82	0.10	11.15	0.06	11.94	0.33	1.257 up	2.173 up	0.02	14.43	Seryl-tRNASynthetase. class IIa.
123191	12.70	0.05	12.66	0.11	14.06	0.12	1.029 down	2.562 up	0.00	165.16	unknown protein
123199	9.46	0.08	11.23	0.02	12.00	0.56	3.404 up	5.813 up	0.01	22.62	SSCRP
123204	12.33	0.08	13.99	0.03	13.74	0.05	3.159 up	2.647 up	0.00	646.36	lipase/esterase
123205	5.51	0.02	5.47	0.26	6.97	0.23	1.029 down	2.750 up	0.00	47.08	unknown protein
123207	12.43	0.09	14.15	0.05	13.93	0.11	3.296 up	2.828 up	0.00	206.34	unknown protein
123226	12.64	0.12	10.99	0.08	9.60	0.27	3.145 down	8.238 down	0.00	130.71	GH37 α.α-trehalase
123227	9.49	0.03	9.24	0.13	8.12	0.15	1.187 down	2.591 down	0.00	92.98	unknown protein

123232	10.75	0.20	6.77	0.05	14.31	0.26	15.789 down	11.782 up	0.00	784.21	GH12 endo-ß-1.4-glucanase
123236	7.67	0.06	7.96	0.16	9.26	0.41	1.222 up	3.011 up	0.01	19.97	SSCRP
123256	7.44	0.13	8.56	0.08	8.61	0.24	2.168 up	2.251 up	0.01	25.23	unknown protein
123262	10.49	0.17	9.91	0.03	8.48	0.23	1.494 down	4.007 down	0.00	82.06	unique protein. HTG. amidase domain. 1 TM
123265	10.92	0.08	11.77	0.01	12.14	0.40	1.801 up	2.330 up	0.03	10.28	unknown protein
123274	12.85	0.29	10.35	0.08	10.70	0.04	5.664 down	4.439 down	0.00	201.71	Aldehyde dehydrogenase
123278	4.56	0.35	5.46	0.34	6.17	0.35	1.875 up	3.054 up	0.01	14.69	unknown protein
123282	8.45	0.13	7.38	0.30	6.72	0.27	2.097 down	3.305 down	0.00	30.89	unknown protein
123325	9.39	0.19	10.46	0.03	10.74	0.23	2.108 up	2.548 up	0.00	31.56	zinc transporter
123396	7.30	0.23	7.27	0.26	8.97	0.53	1.023 down	3.179 up	0.01	14.81	unknown protein
123441	9.28	0.01	10.54	0.33	10.49	0.33	2.384 up	2.308 up	0.02	12.78	unknown protein
123455	9.11	0.08	9.06	0.03	7.76	0.37	1.035 down	2.549 down	0.01	21.40	unknown protein
123473	11.89	0.04	9.96	0.02	8.04	0.15	3.810 down	14.364 down	0.00	761.02	MFS permease
123475	14.92	0.06	14.31	0.13	13.02	0.55	1.527 down	3.709 down	0.02	14.47	cell wall Thr-rich mannoprotein. Distantly related to S. cerevisiae Dan4p.
123476	8.74	0.04	8.61	0.06	11.31	0.34	1.094 down	5.959 up	0.00	98.04	unknown protein
123493	12.52	0.11	12.42	0.10	10.81	0.28	1.073 down	3.276 down	0.00	55.30	Ubiquitin-protein ligase (E3)
123509	9.77	0.07	10.37	0.05	7.79	0.36	1.506 up	3.955 down	0.00	68.32	Zn2Cys6 transcriptional regulator
123510	10.55	0.21	10.14	0.11	7.46	0.53	1.330 down	8.529 down	0.00	46.60	Zn2Cys6 transcriptional regulator
123553	8.78	0.09	10.25	0.15	9.88	0.40	2.779 up	2.147 up	0.02	11.79	short chain dehydrogenase/reductase
123572	10.71	0.04	9.31	0.05	9.52	0.25	2.634 down	2.278 down	0.00	32.50	Phospholipase A2
123604	12.96	0.07	13.26	0.28	13.99	0.23	1.224 up	2.042 up	0.01	16.69	Rps24 (family S16) by homology with similar proteins in other fungi.
123608	7.21	0.05	7.67	0.04	8.59	0.19	1.373 up	2.603 up	0.00	65.47	unique protein
123616	13.85	0.08	14.84	0.18	15.10	0.12	1.984 up	2.365 up	0.00	60.77	short unique protein
123636	12.27	0.10	11.48	0.07	10.86	0.25	1.722 down	2.658 down	0.00	33.73	unknown protein
123639	3.68	0.23	6.03	0.38	5.07	0.25	5.100 up	2.625 up	0.00	37.05	GH64 endo-1.3-β-glucanase
123649	9.84	0.06	9.04	0.11	7.87	0.35	1.749 down	3.910 down	0.00	36.97	unique protein
123659	13.76	0.02	9.39	0.02	11.31	0.07	20.748 down	5.487 down	0.00	3163.03	cell wall protein. istantly related to S. cerevisiae Pir3p.
123695	9.22	0.06	9.99	0.26	10.71	0.11	1.705 up	2.807 up	0.00	68.68	unknown protein
123697	13.70	0.00	12.30	0.03	11.36	0.10	2.644 down	5.059 down	0.00	611.63	Unknown protein
123723	12.27	0.20	12.16	0.10	10.87	0.62	1.085 down	2.645 down	0.04	7.59	Arylacetamide deacetylase

123732	10.92	0.04	9.08	0.12	8.72	0.16	3.583 down	4.606 down	0.00	181.52	unique protein
123740	13.80	0.18	13.29	0.15	12.60	0.20	1.430 down	2.305 down	0.00	29.28	unknown protein
123779	9.12	0.04	9.77	0.05	7.70	0.25	1.577 up	2.671 down	0.00	88.00	unique protein
123805	11.77	0.08	11.32	0.04	10.43	0.12	1.360 down	2.531 down	0.00	138.39	DHBP_synthase.-dihydroxy--butanone -phosphate synthase
123806	11.98	0.00	10.38	0.10	10.07	0.43	3.020 down	3.750 down	0.01	21.98	GPCR. secretin like
123818	11.69	0.07	7.14	0.01	13.01	0.61	23.487 down	2.491 up	0.00	104.73	GH11 endo-ß-1.4-xylanase XYN2
123827	14.33	0.04	13.73	0.03	12.88	0.31	1.521 down	2.737 down	0.01	26.72	bifunctional catalase/peroxidase
123850	13.38	0.04	13.73	0.12	14.50	0.10	1.270 up	2.171 up	0.00	107.42	60s acid ribosomal protein P1 based on homology to corresponding proteins in fungi a
123865	11.33	0.22	13.67	0.03	12.34	0.45	5.058 up	2.021 up	0.01	20.81	Peptidase S8 and S53. subtilisin. kexin. sedolisin
123888	12.51	0.07	11.04	0.13	10.04	0.27	2.767 down	5.528 down	0.00	83.91	unknown protein
123940	4.84	0.37	3.14	0.19	10.18	1.69	3.232 down	40.577 up	0.01	22.82	GH115 methylglurunoyl esterase CIP2
123955	9.64	0.23	13.88	0.14	12.72	0.16	18.904 up	8.468 up	0.00	330.87	Epl1/Sm1
123967	13.79	0.07	14.14	0.06	14.96	0.16	1.272 up	2.252 up	0.00	60.58	HFB3
123968	12.70	0.20	14.32	0.04	14.05	0.07	3.090 up	2.561 up	0.00	140.23	unknown protein
123989	14.28	0.04	11.50	0.12	15.59	0.09	6.841 down	2.485 up	0.00	1418.75	GH7 Cellobiohydrolase CBH1/CEL7a
123992	11.22	0.10	9.33	0.06	13.13	0.65	3.714 down	3.757 up	0.00	38.57	swollenin
123999	7.03	0.26	8.21	0.10	8.27	0.21	2.266 up	2.361 up	0.01	26.76	NADH:flavin oxidoreductase/NADH oxidase
124022	9.18	0.01	6.09	0.20	10.20	0.22	8.500 down	2.036 up	0.00	297.26	Zn2Cys6 transcriptional regulator
124030	10.88	0.02	10.08	0.01	9.77	0.38	1.742 down	2.166 down	0.03	9.47	unknown protein with TIM barrel
124043	3.51	0.39	3.82	0.23	6.63	0.23	1.241 up	8.695 up	0.00	122.71	GH18. chitinase CHI18-14
124059	12.76	0.03	10.05	0.12	13.82	0.05	6.566 down	2.088 up	0.00	2235.49	SSCRP
124065	9.38	0.17	8.53	0.23	12.11	0.39	1.804 down	6.623 up	0.00	98.19	unknown protein. only in bacteria; contains large nuclear transport factor 2 domain
124079	3.15	0.17	3.60	0.27	4.60	0.31	1.365 up	2.734 up	0.01	21.12	Multicopper oxidases
124104	12.47	0.05	12.16	0.09	11.17	0.29	1.247 down	2.473 down	0.01	25.86	unknown protein
124115	13.54	0.15	12.82	0.13	11.26	0.08	1.640 down	4.849 down	0.00	350.81	phosphoenolpyruvate carboxykinase AcuF
124116	10.52	0.02	10.85	0.29	9.46	0.18	1.254 up	2.082 down	0.00	42.80	unknown protein
124119	11.53	0.12	11.32	0.07	9.61	0.26	1.157 down	3.788 down	0.00	73.41	unique protein
124157	13.20	0.03	13.27	0.05	11.47	0.13	1.051 up	3.312 down	0.00	309.69	unknown protein
124170	10.02	0.05	9.59	0.04	8.34	0.25	1.352 down	3.206 down	0.00	59.19	arsenite methyltransferase
124177	13.23	0.08	12.48	0.11	11.86	0.31	1.683 down	2.586 down	0.01	21.10	unknown protein

124187	9.90	0.07	10.12	0.15	11.25	0.14	1.166 up	2.551 up	0.00	90.56	ER-associated protein degradation
124210	13.23	0.01	13.42	0.11	14.65	0.21	1.144 up	2.673 up	0.00	62.30	histone H3
124222	13.50	0.18	13.08	0.11	11.42	0.24	1.336 down	4.232 down	0.00	82.95	CaaX-protease. related to E. nidulans rce1. involved in signal transduction
124228	12.36	0.05	11.82	0.03	10.59	0.11	1.455 down	3.406 down	0.00	300.48	GT2 chitin synthase
124246	9.39	0.13	9.85	0.08	10.41	0.28	1.375 up	2.022 up	0.02	14.02	unknown protein
124249	10.65	0.17	11.83	0.09	12.52	0.38	2.271 up	3.665 up	0.01	24.77	unknown protein
124256	10.29	0.12	11.18	0.14	12.41	0.41	1.855 up	4.359 up	0.00	30.22	phospholipase-like protein
124260	9.65	0.04	9.48	0.07	8.35	0.20	1.123 down	2.464 down	0.00	61.37	Zn2Cys6 transcriptional regulator
124278	11.40	0.01	12.33	0.04	12.90	0.28	1.907 up	2.834 up	0.00	32.90	unknown protein with EXS domain
124283	8.92	0.11	10.62	0.00	11.80	0.27	3.244 up	7.355 up	0.00	123.52	unknown protein. SET and MYND domains
124286	12.19	0.09	11.84	0.07	10.97	0.21	1.274 down	2.340 down	0.00	40.53	Heteromeric CCAAT factors
124295	11.87	0.00	10.23	0.20	13.46	0.18	3.114 down	3.010 up	0.00	262.78	SSCRP
124296	9.17	0.05	7.68	0.10	10.38	1.04	2.810 down	2.316 up	0.04	7.65	unique protein
124299	3.13	0.08	3.23	0.35	5.31	0.33	1.069 up	4.537 up	0.00	50.27	HET-domain protein with WD40 repeats
124302	12.96	0.02	12.54	0.02	11.69	0.10	1.335 down	2.406 down	0.00	203.45	response regulator receiver. distantly related to S. cerevisiae Ssk1p and S. pombe Mcs
124310	3.05	0.45	3.63	0.04	4.94	0.36	1.497 up	3.722 up	0.01	23.04	unknown protein
124338	11.12	0.01	8.97	0.02	8.17	0.19	4.453 down	7.751 down	0.00	270.02	unique protein
124339	5.73	0.16	6.59	0.08	7.43	0.24	1.818 up	3.250 up	0.00	48.41	unique protein
124341	11.80	0.08	12.45	0.01	9.85	0.77	1.561 up	3.879 down	0.01	15.16	mating protein MAT1-2-1

* Abbreviations: SEQ-ID, Trire2:number; S.D. standard deviation; "ratio" specifies the expression ratio vs the parent strain. Note that expression levels (but not the ratios) are given as log2 by the ArrayStar evaluation package
