# Supplementary material for: Functional Analyses of Trichoderma reesei LAE1 Reveal Conserved and Contrasting Roles of This Regulator
Source: G3 (Bethesda). 2013 Feb 1;3(2):369–78. doi: 10.1534/g3.112.005140 (PMC3564997; doi:10.1534/g3.112.005140)
Supplement: Supporting Information [file supp_3.2.369_TableS3.pdf]

Table S3 Comparison of expression results from microarrays and qPCR for selected genes*

Trire2:	gene category	array results		p-value	qPCR results			
		Δlae1	*lae1OE*		*Δlae1*		*lae1OE*	
						S.D.		S.D.
5647	PTH	3.364 down	1.567 down	0.00	0.73	± 0.11	0.55	± 0.14
39587	PTH	155.090 down	2.883 down	0.05	0.03	± 0.02	0.11	± 0.04
62462	PTH	1.475 down	2.438 up	0.00	1.9	± 0.87	1.8	± 0.47
69904	PTH	4.888 down	1.125 up	0.00	1.3	± 0.67	1.3	± 0.17
76763	PTH	3.952 down	1.267 up	0.05	0.3	± 0.12	0.22	± 0.18
82041	PTH	4.287 down	1.305 down	0.04	< 0.1	ND	0.45	± 0.11
105224	PTH	2.763 down	4.166 down	0.07	0.15	± 0.08	0.18	± 0.09
110339	PTH	1.078 down	11.197 up	0.07	3.4	± 2.3	268	± 78.4
110744	PTH	5.100 down	1.514 down	0.00	0.15	± 0.11	1.4	± 0.12
121990	PTH	4.330 down	1.236 down	0.04	0.35	± 0.23	2.2	± 1.2
122824	PTH	10.764 down	35.206 up	0.00	0.22	± 0.04	81.3	± 33.4
112083	HET	2.453 down	2.723 up	0.01	< 0.1	ND	0.3	± 0.08
69187	HET	2.647 down	1.827 down	0.02	0.85	± 0.35	3	± 1.6
107071	HET	1.412 up	3.767 up	0.00	0.83	± 0.42	4.6	± 1.1
111494	HET	2.414 down	1.882 down	0.01	0.81	± 0.28	2.6	± 1.4
23171	NRPS	3.634 up	9.894 up	0.00	1.44	± 0.33	4.14	± 0.51

* values are means of at least 3 replica, S.D. standard deviation
