# Supplementary material for: Functional Analyses of Trichoderma reesei LAE1 Reveal Conserved and Contrasting Roles of This Regulator
Source: G3 (Bethesda). 2013 Feb 1;3(2):369–78. doi: 10.1534/g3.112.005140 (PMC3564997; doi:10.1534/g3.112.005140)
Supplement: Supporting Information [file supp_3.2.369_TableS5.pdf]

Table S5 A few genes are associated with H3K4 methylation, which would be consistent with a positive role of LAE1 in H3K4 methylation*

protein ID	H3K4me2			H3K4me3			ratio *[Δlae1]*	ratio *[lae1OE]*	Annotation
	WT	*Δlae1*	*lae1OE*	WT	*Δlae1*	*lae1OE*			
47926	+	+	+	+			1,723 up	4,383 up	ABC1 family protein
72612			+				2,100 down	3,496 down	acetylornithine deacetylase
53372	+		+	+		+	2,753 down	2,231 down	acyltransferase 3
70859	+		+	+		+	4,320 down	5,344 down	amidase
81420	+	+	+	+			2,875 down	2,643 down	Argonaute siRNA chaperone (ARC) complex subunit
121785	+						19,861 down	2,194 down	ATP-dependent RNA helicase
65315	+		+				16,360 down	11,572 down	bZIP transcription factor
54703	+	+	+	+		+	2,647 down	2,513 down	C2H2 transcriptional regulator
102920	+	+	+	+		+	4,625 down	6,239 down	C2H2 transcriptional regulator
4876	+	+	+	+			2,534 down	5,033 down	catechol dioxygenase
123659	+		+	+		+	20,748 down	5,487 down	cell wall protein, istantly related to S. cerevisiae Pir3p.
75713	+	+	+	+		+	6,996 down	2,139 down	Cytochrome P450 CYP2 subfamily
120088	+	+	+	+		+	1,302 up	3,685 up	cytosolic asparaginyl-tRNA synthetase, required for protein synthesis, catalyzes the spe tRNA.
121877	+	+	+			+	4,705 down	3,710 down	epoxide hydrolase
79741	+	+	+	+		+	1,807 down	2,212 up	ER-bound Farnesyl-diphosphate farnesyltransferase
54144			+				1,097 down	3,141 up	ferric reductase
104211			+	+		+	15,509 down	24,344 up	FKBP-type peptidyl-prolyl cis-trans isomerase
2185			+				1,764 up	7,251 up	GCN5-related N-acetyltransferase
61703	+	+	+	+		+	1,316 up	2,241 up	germinal center kinase, related to S. cerevisiae Kic1
50215	+		+			+	6,380 down	2,374 up	GH16 endo-1,3-β-D-glucosidase/1,3-glucan binding protein
45717			+				4,496 down	4,485 down	GH47 α-1,2-mannosidase
82616	+		+				5,179 down	4,507 down	GH5 membrane bound endoglucanase CEL5b
82633			+				3,948 down	4,784 down	GH72 β-1 3-glucanosyltransferase
76620	+		+				2,041 down	2,566 down	Glycerol-3-phosphate dehydrogenase
123806	+						3,020 down	3,750 down	GPCR, secretin like

77557	+		+		+	7,316 down	5,660 down	GT glycosyltransferases not yet assigned to a family, 3 TMs
66888	+					11,913 down	5,286 down	GT α-1,3-mannosyltransferase
65817	+	+	+	+		5,911 down	3,410 down	GT α-1,3-mannosyltransferase CMT1
4561	+	+	+	+	+	2,718 down	2,569 down	GT α-1,6-mannosyltransferase
119963	+		+	+	+	1,202 up	2,031 up	HFBs
119805	+	+	+	+	+	1,232 up	2,096 up	HFBs
122363	+		+			2,768 down	2,497 up	Hsp26/Hsp42
105260	+	+	+	+	+	2,886 down	12,720 down	MFS permease
53475			+			2,083 down	2,851 down	MFS permease
68813			+			3,217 down	2,939 down	MFS permease
106248			+			1,696 up	3,534 up	monosaccharide transporter (galactose permease ?)
44476	+		+			4,980 down	2,986 down	MRP-type ABC transporter
73536			+			1,012 up	10,116 up	NADP-glutamate dehydrogenase
82105	+					2,755 down	4,288 down	PDR-type ABC transporters
64959	+	+		+	+	13,328 down	2,437 down	phosphatidyl synthase
3568	+	+	+	+	+	5,337 down	3,682 down	phospholipase A2
123572	+	+	+		+	2,634 down	2,278 down	Phospholipase A2
44278	+	+	+	+	+	2,647 down	4,813 down	Rab geranyl transferase escort protein
70355	+		+	+	+	5,994 down	18,605 down	SAM-dependent methylransferase
60758	+		+	+	+	12,719 down	5,079 down	SAM-dependent methyltransferase
4442	+					2,342 down	3,378 down	SAM-dependent methyltransferase
3055	+			+	+	22,757 down	2,485 down	short chain dehydrogenase/reductase
123616			+			1,984 up	2,365 up	short unique protein
71167	+		+			1,202 down	3,268 up	SSCRP
53366	+	+	+	+	+	1,353 up	2,151 up	translation initiation protein Sua5p.
106223	+	+	+	+	+	2,658 down	6,040 down	unique protein
119902	+	+	+	+	+	4,795 down	2,418 down	unique protein
43392	+		+		+	1,651 up	5,859 up	unique protein
104695	+	+	+	+	+	1,708 down	2,243 up	unique protein
105167			+			1,123 up	2,845 up	unique protein

107112			+			1,851 up	2,498 up	unique protein
4851	+		+	+	+	194,650 down	3,832 down	unknown protein
122089	+		+	+	+	7,444 down	3,053 down	unknown protein
65522	+		+			10,051 down	4,155 down	unknown protein
59940	+	+	+	+	+	3,732 down	3,082 down	unknown protein
60616	+	+	+	+	+	4,708 down	3,985 down	unknown protein
3488	+	+	+	+		2,876 down	2,578 down	unknown protein
107202			+			3,857 down	2,407 down	unknown protein
54622	+		+			2,553 down	4,686 up	unknown protein
43199	+		+			1,442 up	3,792 up	unknown protein
43302	+		+			1,007 down	5,446 up	unknown protein
71146	+		+			1,114 up	3,012 up	unknown protein
71154	+		+			1,158 up	2,392 up	unknown protein
120993	+					1,306 down	3,637 up	unknown protein
43083	+	+	+	+		1,236 down	3,216 up	unknown protein
105707			+			1,072 up	3,169 up	unknown protein
50996	+	+		+	+	4,528 down	7,482 down	unknown protein, C2 domain
49928			+			1,144 down	7,578 up	unknown protein, only in Sordariomycetes
102499			+			1,036 down	6,618 up	Zn2Cys6 transcriptional regulator

* genes are sorted alphabetically according to gene anniotation; other abbreviations and the color code are used as in Supplementary Table S3
